# Supplementary material for: Divergent rhodium-catalyzed electrochemical vinylic C–H annulation of acrylamides with alkynes
Source: Nat Commun. 2021 Feb 10;12:930. doi: 10.1038/s41467-021-21190-8 (PMC7876044; doi:10.1038/s41467-021-21190-8)
Supplement: Supplementary file 1 — Supplementary information. [file 41467_2021_21190_MOESM1_ESM.pdf]

Supplementary Information

Divergent Rhodium-Catalyzed Electrochemical Vinylic C–H Annulation of Acrylamides with Alkynes

Yi-Kang Xing,[a] Xin-Ran Chen,[b] Qi-Liang Yang,[c] Shuo-Qing Zhang,[b] Hai-Ming Guo,[c] Xin Hong,[b] Tian-Sheng Mei[a]

[a]State Key Laboratory of Organometallic Chemistry, Center for Excellence in Molecular Synthesis, Shanghai Institute of Organic Chemistry, University of Chinese Academy of Sciences, Chinese Academy of Sciences, 345 Lingling Road, Shanghai, 200032, China

[b] Department of Chemistry, Zhejiang University, Hangzhou, 310027, China

[c]Henan Key Laboratory of Organic Functional Molecules and Drug Innovation, Collaborative Innovation Center of Henan Province for Green Manufacturing of Fine Chemicals, School of Chemistry and Chemical Engineering, Henan Normal University, Xinxiang, Henan, 453007, China

Table of Contents

1. General Information..S3

2. Synthesis and Characterization Data for Alkynes..S4

3. Synthesis and Characterization Data for Acrylic Amides...........................S10

4. Optimization of the Reaction Conditions...S29

5. General Procedure for Rh(III)-Catalyzed Annulation of Acrylic Amides with Alkynes……………………………..…………...............................S36

6. Graphical Guide for Rhodium-Catalyzed Oxidative Annulation of Acrylic Amides with Alkynes...S38

7. Table of Failed Examples...S39

8. Characterization Data for the Products…………………………………....S39

9. Synthesis of Rhodium (I) Sandwich Complex **10** and **11**……………..……..S91

10. Synthesis Lactone **3aa** from Cyclic Imidate **3a** …..…………….……..…..S93

11. Preliminary Mechanistic Studies ………………………………...…......S94

12. Deuterium-Labeled Experiment ……………………………….....….......S96

13. Kinetic Isotope Effect Studies …………………………………...…..........S98

14. General Procedure for Kinetic Experiment …………………………....S101

15. Visual Kinetic Analysis ………………………………………………......S107

16. Effect of Current On Annulation Reaction ……………………………..S109

17. Electrochemical Procedure for Cyclic Voltammetry…..……….…..……..S111

18. X-ray Crystal Structure Data...S115

19. Computational Result...S123

20. Spectral Data..S135

21. Supplementary References..S336

1. General Information

All the electrochemical oxidations were performed in ***IKA ElectraSyn 2.0*** equipped with two platinum electrodes (each 0.8 × 3.0 cm^2) or in an undivided cell equipped with two platinum electrodes (1.0 × 1.0 cm^2) unless otherwise noted. $(Cp^*RhCl_2)_2$ was purchased form aladdin chemical company. And other commercial reagents were purchased from TCI, Sigma-Aldrich, Adamas-beta and Energy Chemical of the highest purity grade. They were used without further purification unless specified. Column chromatography was performed using either 100–200 Mesh or 300–400 Mesh silica gel. Visualization of spots on TLC plate was accomplished with UV light (254 nm).

^1H and ^{13}C NMR spectra were recorded at room temperature in $CDCl_3$ (containing 0.03% TMS) solutions on Varian Inova 400 spectrometer or Agilent AV 400 spectrometer. ^1H NMR spectra was recorded at 400 MHz, ^{13}C NMR spectra was recorded at 100 MHz. ^1H NMR spectra was recorded with tetramethylsilane (δ = 0.00 ppm) as internal reference in $CDCl_3$ and ^{13}C NMR spectra was recorded with $CDCl_3$ (δ = 77.00 ppm). The following abbreviations were used to explain multiplicities: s = singlet, d = doublet, t = triplet, q = quartet, m = multiplet, and br = broad. Infrared spectra were obtained on a Bio-Rad FTS-185 instrument. High-resolution mass spectra was obtained by using Agilent Technologies 6224 TOF LC/MS mass spectrometer.

2. Synthesis and Characterization Data for Alkynes:

Alkynes **2a**, **2n, 2o, 2p, 2t**, **2u**, **2v**, **2w, 2x** and **2y** are commercially available and were used as supplied.

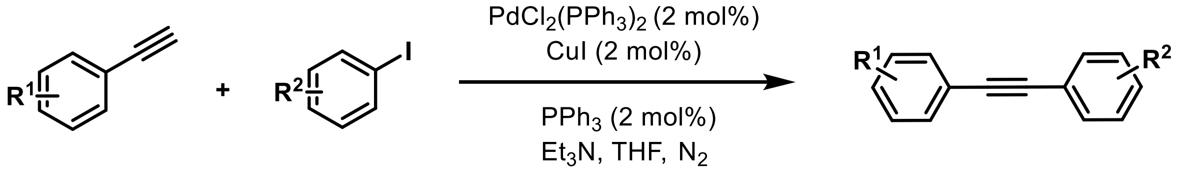

The corresponding aryl iodide (10 mmol, 1 equiv), $PdCl_2(PPh_3)_2$ (1–2 mol%), CuI (1–2 mol%), PPh_3 (1–2 mol%) and phenylacetylene (1–1.1 equiv) were added to a 50 mL Schlenk flask with a stir bar under an atmosphere of nitrogen. Then tetrahydrofuran (20 mL) and triethylamine (20 mL) were added sequentially. The reaction mixture was then stirred at room temperature overnight. Afterwards 15 mL of water were added and the reaction mixture was extracted with EtOAc (3 × 25 mL). The combined organic fractions were washed with brine and dried over Na_2SO_4. After filtration, the solvent was removed under reduced pressure. The residue was purified by silica gel column chromatography using petroleum ether and ethyl acetate as the eluent. The yields were not optimized for the synthesis of alkynes.

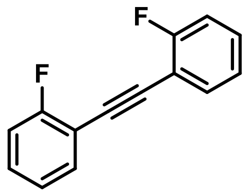

2b

1,2-Bis(2-fluorophenyl)ethyne (2b)

^{1}H NMR (400 MHz, $CDCl_3$): δ 7.61 – 7.51 (m, 2 H), 7.38 – 7.29 (m, 2 H), 7.18 – 7.07 (m, 4 H). The spectral data are in accordance with those reported in the

literature.[1]

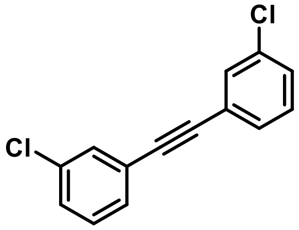

2c

1,2-Bis(3-chlorophenyl)ethyne (2c)

^{1}H NMR (400 MHz, $CDCl_3$): δ 7.52 (t, *J* = 1.6 Hz, 2 H), 7.40 (dt, *J* = 7.6, 1.2 Hz, 2 H), 7.36 – 7.26 (m, 4 H). The spectral data are in accordance with those reported in the literature.[1]

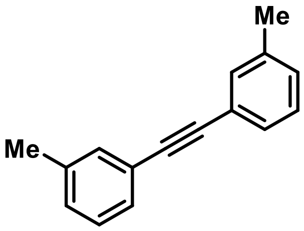

2d

1,2-Di-m-tolylethyne (2d)

^{1}H NMR (400 MHz, $CDCl_3$): δ 7.41 – 7.35 (m, 4 H), 7.26 (t, *J* = 7.6 Hz, 2 H), 7.16 (d, *J* = 7.6 Hz, 2 H), 2.38 (s, 6 H). The spectral data are in accordance with those reported in the literature.[1]

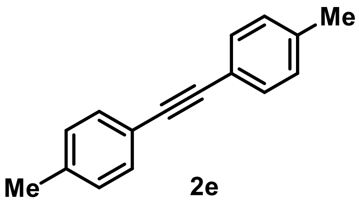

2e

1,2-Di-p-tolylethyne (2e)

^{1}H NMR (400 MHz, CDCl$_3$): δ 7.42 (d, *J* = 8.0 Hz, 4 H), 7.15 (d, *J* = 8.0 Hz, 4 H), 2.37 (s, 6 H). The spectral data are in accordance with those reported in the literature.[2]

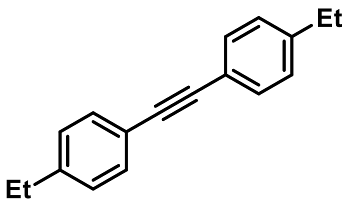

2f

1,2-Bis(4-ethylphenyl)ethyne (2f)

^{1}H NMR (400 MHz, CDCl$_3$): δ 7.45 (d, *J* = 8.4 Hz, 4 H), 7.18 (d, *J* = 8.4 Hz, 4 H), 2.67 (q, *J* = 7.6 Hz, 4 H), 1.25 (t, *J* = 7.6 Hz, 6 H). The spectral data are in accordance with those reported in the literature.[3]

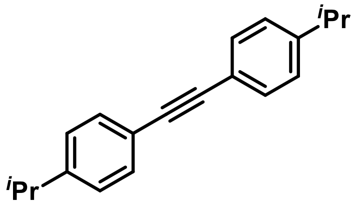

2g

1,2-Bis(4-isopropylphenyl)ethyne (2g)

^{1}H NMR (400 MHz, CDCl$_3$): δ 7.46 (d, *J* = 8.0 Hz, 4 H), 7.21 (d, *J* = 8.0 Hz, 4 H), 2.96 – 2.80 (m, 2 H), 1.26 (d, *J* = 6.8 Hz, 12 H). The spectral data are in accordance with those reported in the literature.[4]

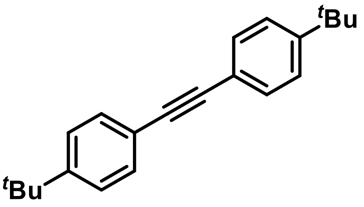

2h

1,2-Bis(4-(tert-butyl)phenyl)ethyne (2h)

^{1}**H NMR (400 MHz, CDCl$_3$):** δ 7.49 (d, *J* = 8.6 Hz, 4 H), 7.42 – 7.34 (m, 4 H), 1.35 (s, 18 H). The spectral data are in accordance with those reported in the literature.[5]

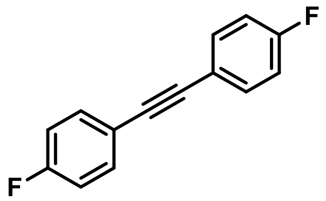

2i

1,2-Bis(4-fluorophenyl)ethyne (2i)

^{1}**H NMR (400 MHz, CDCl$_3$):** δ 7.59 – 7.41 (m, 4 H), 7.14 – 6.97 (m, 4 H). The spectral data are in accordance with those reported in the literature.[6]

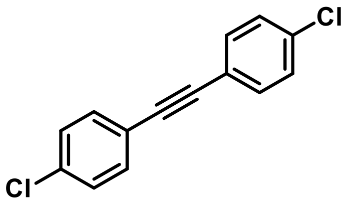

2j

1,2-Bis(4-chlorophenyl)ethyne (2j)

^{1}**H NMR (400 MHz, CDCl$_3$):** δ 7.47 – 7.41 (m, 4 H), 7.36 – 7.29 (m, 4 H). The spectral data are in accordance with those reported in the literature.[2]

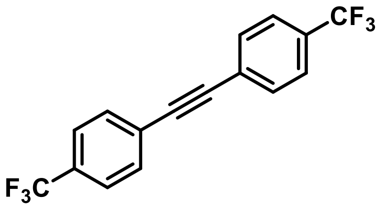

2k

1,2-Bis(4-(trifluoromethyl)phenyl)ethyne (2k)

^{1}**H NMR (400 MHz, CDCl$_3$):** δ 7.73 – 7.54 (m, 8 H). The spectral data are in accordance with those reported in the literature.[5]

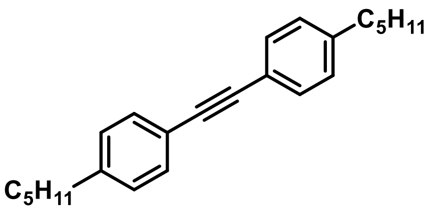

2l

1,2-Bis(4-pentylphenyl)ethyne (2l)

^{1}H NMR (400 MHz, CDCl$_3$): δ 7.44 (d, *J* = 7.2 Hz, 4 H), 7.16 (d, *J* = 7.2 Hz, 4 H), 2.61 (t, *J* = 7.2 Hz, 4 H), 1.70 – 1.58 (m, 4 H), 1.43 – 1.25 (m, 8 H), 0.90 (t, *J* = 6.3 Hz, 6 H). The spectral data are in accordance with those reported in the literature.[4]

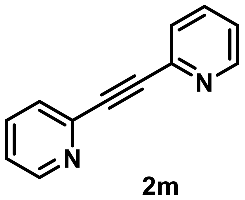

2m

1,2-Di(pyridin-2-yl)ethyne (2m)

^{1}H NMR (400 MHz, CDCl$_3$): δ 8.77 – 8.52 (m, 2 H), 7.85 – 7.54 (m, 4 H), 7.36 – 7.18 (m, 2 H). The spectral data are in accordance with those reported in the literature.[7]

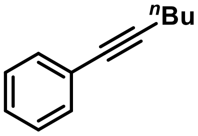

2q

Hex-1-yn-1-ylbenzene (2q)

^{1}H NMR (400 MHz, CDCl$_3$): δ 7.49 – 7.39 (m, 2 H), 7.38 – 7.08 (m, 3 H), 2.46 (t, *J* = 7.2 Hz, 2 H), 1.70 – 1.60 (m, 2 H), 1.57 – 1.47 (m, 2 H), 1.01 (t, *J* = 7.2

Hz, 3 H). The spectral data are in accordance with those reported in the literature.[2]

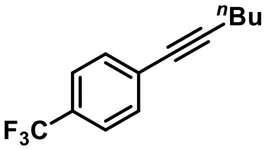

2r

1-(Hex-1-yn-1-yl)-4-(trifluoromethyl)benzene (2r)

^{1}H NMR (400 MHz, $CDCl_3$): δ 7.53 (d, *J* = 8.4 Hz, 2 H), 7.48 (d, *J* = 8.0 Hz, 2 H), 2.43 (t, *J* = 7.2 Hz, 2 H), 1.65 – 1.57 (m, 2 H), 1.53 – 1.45 (m, 2 H), 0.96 (t, *J* = 7.6 Hz, 3 H). The spectral data are in accordance with those reported in the literature.[8]

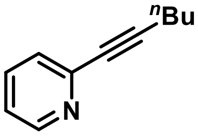

2s

2-(hex-1-yn-1-yl)pyridine (2s)

^{1}H NMR (400 MHz, $CDCl_3$): δ 8.55 – 8.48 (m, 1 H), 7.62 – 7.55 (m, 1 H), 7.39 – 7.30 (m, 1 H), 7.21 – 7.09 (m, 1 H), 2.43 (t, *J* = 7.2 Hz, 2 H), 1.66 – 1.55 (m, 2 H), 1.52 – 1.43 (m, 2 H), 0.93 (t, *J* = 7.2 Hz, 3 H). The spectral data are in accordance with those reported in the literature.[8]

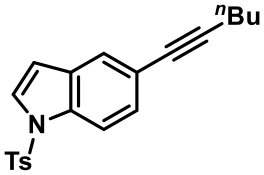

2z

5-(Hex-1-yn-1-yl)-1-tosyl-1H-indole (2z)

^{1}H NMR (400 MHz, CDCl$_3$): δ 7.91 (d, *J* = 8.4 Hz, 1 H), 7.73 (d, *J* = 8.4 Hz, 2 H), 7.59 – 7.52 (m, 2 H), 7.35 (dd, *J* = 8.8, 1.2 Hz, 1 H), 7.19 (d, *J* = 8.0 Hz, 2 H), 6.59 (d, *J* = 3.6 Hz, 1 H), 2.41 (t, *J* = 7.2 Hz, 2 H), 2.31 (s, 3 H), 1.63 – 1.54 (m, 2 H), 1.54 – 1.44 (m, 2 H), 0.95 (t, *J* = 7.2 Hz, 3 H). The spectral data are in accordance with those reported in the literature.[9]

3. Synthesis and Characterization Data for Acrylic amide

Reaction scheme for 1a-1s synthesis

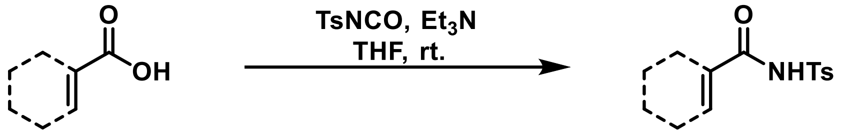

Synthetic procedure A[10] :

To a solution of acrylic acid (1 equiv.) in THF (0.5 M) was added p-tosyl isocyanate (1 equiv.). After stirring the resulting clear solution at rt for 10 min, triethyl amine (1 equiv.) was added dropwise, with release of gas. The progress of the reaction was monitored using TLC. Once the acrylic acids disappeared, the mixture was diluted with EtOAc and washed with 2 M HCl . The organic layer was dried over MgSO4, filtered and concentrated under reduced pressure. The residue was subjected to column chromatography on silica gel to deliver substrates **1a, 5a – 5u**.

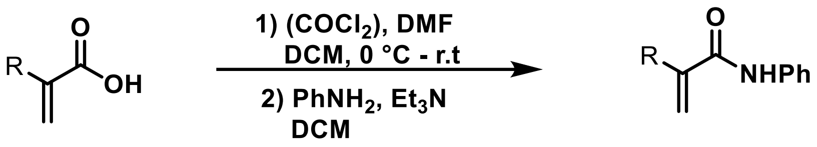

Synthetic procedure B[11] :

To a solution of the carboxylic acid (10.0 mmol, 1.0 equiv.) in dry CH_2Cl_2 (60 mL) at 0 °C was added dropwise $(COCl)_2$ (10 mmol, 1 equiv.) followed by a catalytic amount of dry DMF (2 drops). The reaction was allowed to stir at rt for 3 h. The solvent was then removed under reduce pressure to afford the corresponding crude acid chloride.

Amine (10 mmol) and Et_3N (10 mmol, 1 equiv.) are dissolved in 100 ml of CH_2Cl_2. Acid chloride in 20 mL CH_2Cl_2 are added slowly to the ice-cold solution and are stirred for 1 h at rt. The reaction mixture was washed with saturated $NaHCO_3$ (aq) and saturated NaCl (aq). The organic phase was dried over Na_2SO_4, filtered and removed under reduced pressure. The crude reaction mixture was purified by flash column chromatography to afford the substrates **1b, 8a – 8f.**

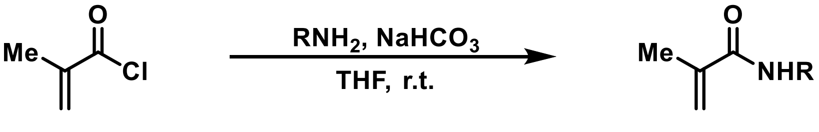

Synthetic procedure C[12] :

To a single flask was added RNH_2 (11 mmol, 1.1 equiv), $NaHCO_3$ (35 mmol, 3.5 equiv) and THF (40 mL). To this suspension was introduced methacryloyl chloride (10 mmol, 1.0 equiv) slowly at r.t. The mixture was allowed to stir at r.t for 3h to reach completion which was determined TLC

monitoring. To the mixture was treated with water (~15 mL) and EtOAc (~30 mL). The phases were separated and the aqueous phase was extracted with EtOAc (~20 mL) again. The organic phase was combined and washed with 1 M HCl (~15 mL) once followed by brine (~15 mL) once. The organic phase was dried over Na_2SO_4, filtered and removed under reduced pressure. The crude reaction mixture was purified by flash column chromatography to afford the substrates **1c – 1f**.

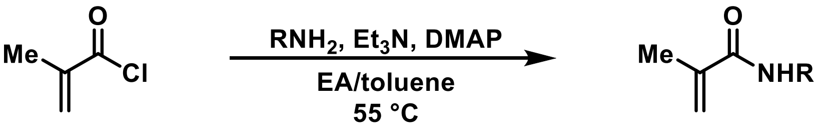

Synthetic procedure D[13] :

To a solution of RNH_2 (10 mmol, 1.0 equiv), DMAP (0.02 mmol, 0.5 mol%), NEt_3 (25 mmol, 2.5 equiv) in EtOAc (5 mL, 2.0 M) was added a solution of methacryloyl chloride (11 mmol, 1.1 equiv) in toluene (12.5 mL, 0.8 M) slowly under argon atmosphere. After being stirred at 55 ℃ for 3~5 hours, the reaction mixture was cooled to room temperature and quenched with 1.0 N HCl. The resulting mixture was extracted with EtOAc (three times), dried over MgSO4, and concentrated under reduced pressure. The crude reaction mixture was purified by flash column chromatography to afford the substrates **1g – 1k**.

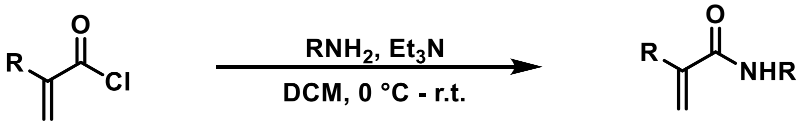

Synthetic procedure E[14] :

Amine (10 mmol) and Et_3N (10 mmol, 1 equiv.) are dissolved in 100 ml of CH_2Cl_2. Acid chloride in 20 mL CH_2Cl_2 are added slowly to the ice-cold solution and are stirred for 1 h at rt. The reaction mixture was washed with saturated $NaHCO_3$ (aq) and saturated NaCl (aq). The organic phase was dried over Na_2SO_4, filtered and removed under reduced pressure. The crude reaction mixture was purified by flash column chromatography to afford the substrates **1i**, **1j**.

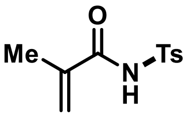

1a

***N*-tosylmethacrylamide (1a)**

^{1}H NMR (400 MHz, CDCl$_3$): δ 9.53 (s, 1 H), 7.97 (d, *J* = 8.0 Hz, 2 H), 7.31 (d, *J* = 8.0 Hz, 2 H), 5.89 (s, 1 H), 5.59 – 5.48 (m, 1 H), 2.39 (s, 3H), 1.84 (s, 3 H). The spectral data are in accordance with those reported in the literature.[10]

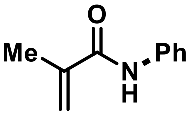

1b

***N*-phenylmethacrylamide (1b)**

^{1}H NMR (400 MHz, CDCl$_3$): δ 7.83 – 7.63 (m, 1 H), 7.60 – 7.52 (m, 2 H), 7.37 – 7.28 (m, 2 H), 7.11 (t, *J* = 7.4 Hz, 1 H), 5.78 (s, 1 H), 5.47 – 5.40 (m, 1 H), 2.05 (s, 3 H). The spectral data are in accordance with those reported in the

literature.[15]

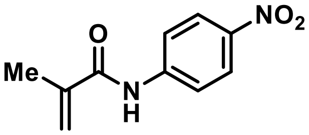

1c

***N*-(4-nitrophenyl)methacrylamide (1c)**

^{1}H NMR (400 MHz, $CDCl_3$): δ 8.26 – 8.18 (m, 2 H), 7.80 – 7.72 (m, 2 H), 5.85 (m, 1 H), 5.56 (m, 1 H), 2.08 (s, 3 H). The spectral data are in accordance with those reported in the literature.[16]

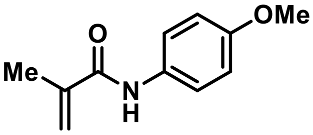

1d

***N*-(4-methoxyphenyl)methacrylamide (1d)**

^{1}H NMR (400 MHz, $CDCl_3$): δ 7.55 (s, 1 H), 7.45 (d, J = 8.8 Hz, 2 H), 6.85 (d, J = 9.2 Hz, 2 H), 5.76 (s, 1 H), 5.41 (s, 1 H), 3.78 (s, 3 H), 2.04 (s, 3 H). The spectral data are in accordance with those reported in the literature.[16]

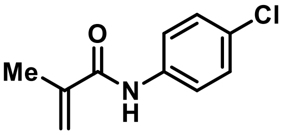

1e

***N*-(4-chlorophenyl)methacrylamide (1e)**

^{1}H NMR (400 MHz, $CDCl_3$): δ 7.56 – 7.46 (m, 2 H), 7.34 – 7.24 (m, 2 H), 5.79 (m, 1 H), 5.48 (m, 1 H), 2.10 – 2.00 (m, 3 H). The spectral data are in

accordance with those reported in the literature.[17]

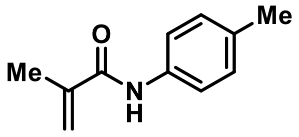

1f

***N*-(p-tolyl)methacrylamide (1f)**

^{1}H NMR (400 MHz, $CDCl_3$): δ 7.44 (d, *J* = 8.4 Hz, 2 H), 7.13 (d, *J* = 7.6 Hz, 2 H), 5.79 – 5.76 (m, 1 H), 5.44 (m, 1 H), 2.32 (s, 3 H), 2.06 (m, 3 H). The spectral data are in accordance with those reported in the literature.[18]

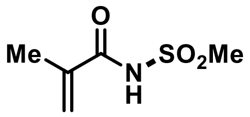

1g

***N*-(methylsulfonyl)methacrylamide (1g)**

^{1}H NMR (400 MHz, $CDCl_3$): δ 5.90 (d, *J* = 1.2 Hz, 1 H), 5.68 (d, *J* = 1.6 Hz, 1 H), 3.36 (s, 3 H), 1.99 (s, 3 H). The spectral data are in accordance with those reported in the literature.[13]

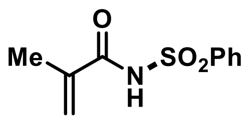

1h

***N*-(phenylsulfonyl)methacrylamide (1h)**

^{1}H NMR (400 MHz, $CDCl_3$): δ 8.55 (s, 1 H), 8.15 – 8.08 (m, 2 H), 7.71 – 7.62 (m, 1 H), 7.57 (dd, *J* = 8.4, 7.2 Hz, 2 H), 5.82 (d, *J* = 1.6 Hz, 1 H), 5.60 (d, *J* =

1.6 Hz, 1 H), 1.90 (s, 3 H). The spectral data are in accordance with those reported in the literature.[13]

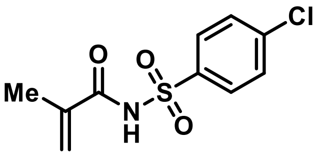

1i

***N*-((4-chlorophenyl)sulfonyl)methacrylamide (1i)**

^{1}H NMR (400 MHz, CDCl$_3$): δ 8.50 (s, 1 H), 8.05 (d, *J* = 8.4 Hz, 1 H), 7.53 (d, *J* = 8.8 Hz, 2 H), 5.82 (d, *J* = 1.3 Hz, 1 H), 5.62 (d, *J* = 1.7 Hz, 1 H), 1.90 (s, 3 H). The spectral data are in accordance with those reported in the literature.[19]

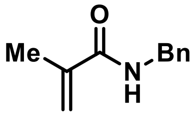

1j

***N*-benzylmethacrylamide (1j)**

^{1}H NMR (400 MHz, CDCl$_3$): δ 7.40 – 7.23 (m, 5 H), 5.71 (s, 1 H), 5.34 (s, 1 H), 4.49 (d, *J* = 6.0 Hz, 2 H), 1.97 (s, 3 H). The spectral data are in accordance with those reported in the literature.[14]

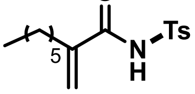

5a

2-Methylene-*N*-tosylbutanamide (5a)

^{1}H NMR (400 MHz, CDCl$_3$): δ 7.96 (d, *J* = 8.0 Hz, 2 H), 7.30 (d, *J* = 8.0 Hz, 2

H), 5.85 (s, 1 H), 5.47 (s, 1 H), 2.40 (s, 3H), 2.18 (t, *J* = 7.6 Hz, 2 H), 1.30 (q, *J* = 7.6 Hz, 2 H), 1.23 – 1.09 (m, 6 H), 0.86 – 0.73 (m, 3 H). The spectral data are in accordance with those reported in the literature.[10]

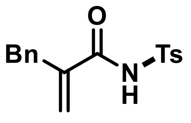

5b

2-Benzyl-*N*-tosylacrylamide (5b)

^{1}H NMR (400 MHz, CDCl$_3$): δ 9.29 (s, 1 H), 7.87 (d, *J* = 8.4 Hz, 2 H), 7.26 (d, *J* = 8.0 Hz, 2 H), 7.23 – 7.14 (m, 3 H), 7.09 – 7.01 (m, 2 H), 5.94 (s, 1 H), 5.42 – 5.31 (m, 1 H), 3.54 (s, 2 H), 2.40 (s, 3 H). The spectral data are in accordance with those reported in the literature.[10]

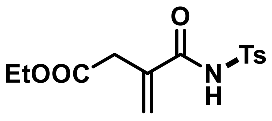

5c

Ethyl 3-(tosylcarbamoyl)but-3-enoate (5c)

The representative synthetic procedure A was followed. Purification by column chromatography (*n*-hexane/EtOAc: 5/1) yielded **5c** as a white solid. **M. p.: 120.2 – 145.2 ℃. ^{1}H NMR (400 MHz, CDCl$_3$):** δ 9.56 (s, 1 H), 7.97 (d, *J* = 8.0 Hz, 2 H), 7.33 (d, *J* = 8.0 Hz, 2 H), 6.03 (s, 1 H), 5.67 (s, 1 H), 4.09 (q, *J* = 7.2 Hz, 2 H), 3.31 (s, 2 H), 2.42 (s, 3 H), 1.18 (t, *J* = 7.2 Hz, 3 H). **^{13}C NMR (100 MHz, CDCl$_3$)**: δ 171.0, 164.5, 145.0, 135.9, 135.5, 129.5, 128.5, 126.6, 61.6, 37.6, 21.6, 13.9. **IR** (neat): 3147, 1697, 1458, 1341, 1300, 1208, 1162, 1123,

1081, 1027, 819, 753, 656, 539 cm^{-1}. **HRMS** (ESI-TOF) m/z Calcd for $C_{14}H_{17}NO_5NaS$ $[M+Na]^+$, 334.0720 found 334.0717.

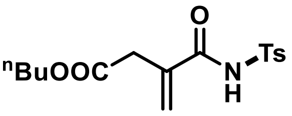

5d

Butyl 3-(tosylcarbamoyl)but-3-enoate (5d)

The representative synthetic procedure A was followed. Purification by column chromatography (*n*-hexane/EtOAc: 5/1) yielded **5d** as a white solid. **M. p.: 105.8 – 108.6 ℃.** **^{1}H NMR (400 MHz, $CDCl_3$):** δ 9.91 (s, 1 H), 7.97 (d, *J* = 8.0 Hz, 2 H), 7.32 (d, *J* = 8.0 Hz, 2 H), 6.09 (s, 1H), 5.71 (s, 1 H), 4.02 (t, *J* = 6.8 Hz, 2 H), 3.33 (s, 2 H), 2.41 (s, 3 H), 1.58 – 1.47 (m, 2 H), 1.37 – 1.24(m, 2 H), 0.89 (t, *J* = 6.8 Hz, 3 H). **^{13}C NMR (100 MHz, $CDCl_3$)**: δ 170.9, 164.6, 144.7, 135.6, 135.4, 129.3, 128.2, 126.4, 65.0, 37.3, 30.1, 21.4, 18.7, 13.4. **IR** (neat): 3163, 2964, 1695, 1461, 1348, 1301, 1210, 1161, 1120, 1081, 839, 655, 545 cm^{-1}. **HRMS** (ESI-TOF) m/z Calcd for $C_{16}H_{21}NO_5NaS$ $[M+Na]^+$, 362.1033 found 362.1037.

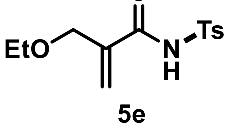

5e

2-(Ethoxymethyl)-*N*-tosylacrylamide (5e)

^{1}H NMR (400 MHz, $CDCl_3$): δ 10.19 – 9.88 (m, 1 H), 7.98 (d, *J* = 8.4 Hz, 2 H), 7.33 (d, *J* = 8.0 Hz, 2 H), 6.22 (s, 1H), 5.70 (s, 1 H), 4.18 (s, 2 H), 3.53 (q, *J* =

7.0 Hz, 2 H), 2.40 (s, 3 H), 1.24 (t, *J* = 7.0 Hz, 3 H). The spectral data are in accordance with those reported in the literature.[10]

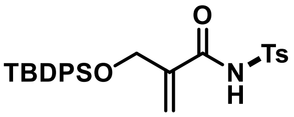

5f

2-(((Tert-butyldiphenylsilyl)oxy)methyl)-*N*-tosylacrylamide (5f)

The representative synthetic procedure A was followed. Purification by column chromatography (*n*-hexane/EtOAc: 5/1) yielded **5f** as a colorless semisolid. **^{1}H NMR (400 MHz, $CDCl_3$):** δ 8.06 – 7.98 (m, 2 H), 7.69 – 7.61 (m, 4 H), 7.50 – 7.44 (m, 2 H), 7.44 – 7.38 (m, 4 H), 7.35 (d, *J* = 8.4 Hz, 2 H), 6.15 (s, 1 H), 5.45 (s, 1 H), 4.37 (s, 2 H), 2.44 (s, 3 H), 1.12 (s, 9 H). **^{13}C NMR (100 MHz, $CDCl_3$)**: δ 163.3, 144.9, 138.5, 135.7, 135.4, 131.8, 130.2, 129.5, 128.5, 127.9, 126.8, 63.9, 26.7, 21.6, 19.0. **IR** (neat): 3237, 2932, 2858, 1697, 1427, 1346, 1169, 1070, 812, 699, 661, 554, 496 cm^{-1}. **HRMS** (ESI-TOF) m/z Calcd for $C_{27}H_{31}NO_4NaSiS$ $[M+Na]^+$, 516.1635 found 516.1632**.**

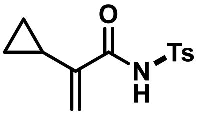

5g

2-Cyclopropyl-*N*-tosylacrylamide (5g)

^{1}H NMR (400 MHz, $CDCl_3$): δ 7.98 (d, *J* = 8.0 Hz, 2 H), 7.33 (d, *J* = 8.0 Hz, 2 H), 5.97 (s, 1 H), 5.32 (s, 1 H), 2.41 (s, 3 H), 1.62 – 1.38 (m, 1 H), 0.88 – 0.72

(m, 2 H), 0.53 – 0.40 (m, 2 H) The spectral data are in accordance with those reported in the literature.[10]

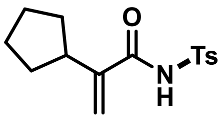

5h

2-Cyclopentyl-*N*-tosylacrylamide (5h)

The representative synthetic procedure A was followed. Purification by column chromatography (*n*-hexane/EtOAc: 5/1) yielded **5h** as a white solid. **M. p.: 100.3 – 101.8 ℃. ^{1}H NMR (400 MHz, $CDCl_3$):** δ 9.01 – 8.78 (m, 1 H), 7.98 (d, *J* = 8.4 Hz, 2 H), 7.34 (d, *J* = 8.4 Hz, 2 H), 5.74 (s, 1 H), 5.48 (s, 1 H), 2.82 – 2.66 (m, 1 H), 2.43 (s, 3 H), 1.86 – 1.73 (m, 2 H), 1.66 – 1.46 (m, 4 H), 1.37 – 1.18 (m, 2 H). **^{13}C NMR (100 MHz, $CDCl_3$)**: δ 165.9, 146.8, 145.0, 135.5, 129.5, 128.4, 119.4, 119.4, 41.1, 31.6, 24.6, 21.6. **IR** (neat): 3249, 2949, 2865, 1702, 1633, 1593, 1411, 1325, 1160, 1077, 927, 864, 805, 534 cm^{-1}. **HRMS** (ESI-TOF) m/z Calcd for $C_{15}H_{19}NO_3NaS$ $[M+Na]^+$, 316.0978 found 316.0974.

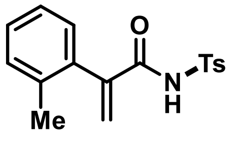

5i

2-(O-tolyl)-*N*-tosylacrylamide (5i)

^{1}H NMR (400 MHz, $CDCl_3$): δ 7.94 (d, *J* = 8.0 Hz, 3 H), 7.38 – 7.29 (m, 3 H), 7.28 – 7.18 (m, 2 H), 7.17 – 7.07 (m, 1 H), 6.47 (s, 1 H), 5.59 (s, 1 H), 2.45 (s, 3

H), 2.09 (s, 3 H). The spectral data are in accordance with those reported in the literature.[20]

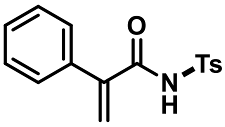

5j

2-Phenyl-*N*-tosylacrylamide (5j)

^{1}H NMR (400 MHz, CDCl$_3$): δ 8.88 (s, 1 H), 7.95 (d, *J* = 8.4 Hz, 2 H), 7.36 – 7.28 (m, 5 H), 7.26 – 7.20 (m, 2 H), 6.08 (s, 1 H), 5.72 (s, 1 H), 2.39 (s, 3 H). The spectral data are in accordance with those reported in the literature.[10]

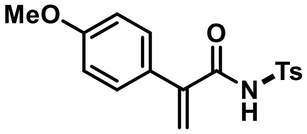

5k

2-(4-Methoxyphenyl)-*N*-tosylacrylamide (5k)

^{1}H NMR (400 MHz, CDCl$_3$): δ 8.45 (s, 1 H), 7.96 (d, *J* = 8.4 Hz, 2 H), 7.34 (d, *J* = 8.4 Hz, 2 H), 7.18 (d, *J* = 8.8 Hz, 2 H), 6.88 (d, *J* = 8.8 Hz, 2 H), 6.08 (s, 1 H), 5.66 (s, 1 H), 3.80 (s, 3 H), 2.44 (s, 3 H). The spectral data are in accordance with those reported in the literature.[10]

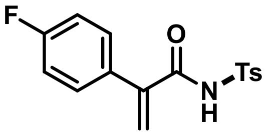

5l

2-(4-Fluorophenyl)-*N*-tosylacrylamide (5l)

^{1}H NMR (400 MHz, CDCl$_3$): δ 8.41 (s, 1 H), 7.97 (d, *J* = 8.4 Hz, 2 H), 7.36 (d, *J* = 8.4 Hz, 2 H), 7.30 – 7.21 (m, 2 H), 7.10 – 7.02 (m, 2 H), 6.15 (s, 1 H), 5.74 (s, 1 H), 2.46 (s, 3 H). The spectral data are in accordance with those reported in the literature.[10]

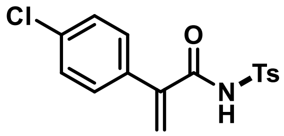

5m

2-(4-Chlorophenyl)-*N*-tosylacrylamide (5m)

^{1}H NMR (400 MHz, CDCl$_3$): δ 7.95 (d, *J* = 8.4 Hz, 2 H), 7.39 – 7.29 (m, 4 H), 7.23 – 7.16 (m, 2 H), 6.14 (s, 1 H), 5.76 (s, 1 H), 2.45 (s, 3 H). The spectral data are in accordance with those reported in the literature.[10]

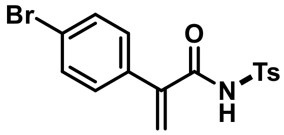

5n

2-(4-Bromophenyl)-*N*-tosylacrylamide (5n)

^{1}H NMR (400 MHz, CDCl$_3$): δ 7.96 (d, *J* = 8.4 Hz, 2 H), 7.49 (d, *J* = 8.4 Hz, 2 H), 7.35 (d, *J* = 8.0 Hz, 2 H), 7.14 (d, *J* = 8.4 Hz, 2 H), 6.16 (s, 1 H), 5.77 (s, 1 H), 2.45 (s, 3 H). The spectral data are in accordance with those reported in the literature.[10]

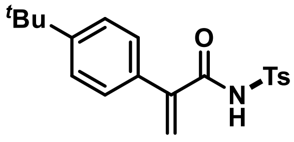

5o

2-(4-(Tert-butyl)phenyl)-N-tosylacrylamide (5o)

^{1}H NMR (400 MHz, DMSO-*d*$_6$): δ 8.51 (s, 1 H), 7.98 (d, *J* = 8.0 Hz, 2 H), 7.40 (d, *J* = 8.4 Hz, 2 H), 7.35 (d, *J* = 8.0 Hz, 2 H), 7.20 (d, *J* = 8.0 Hz, 2 H), 6.16 (s, 1 H), 5.71 (s, 1 H), 2.44 (s, 3 H), 1.32 (s, 9 H). The spectral data are in accordance with those reported in the literature.[10]

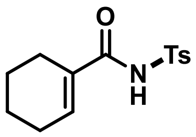

5p

***N*-Tosylcyclohex-1-ene-1-carboxamide (5p)**

^{1}H NMR (400 MHz, CDCl$_3$): δ 7.98 (d, *J* = 8.4 Hz, 2 H), 7.33 (d, *J* = 8.0 Hz, 2 H), 6.83 – 6.72 (m, 1 H), 2.43 (s, 3 H), 2.21 – 2.10 (m, 4 H), 1.65 – 1.49 (m, 4 H). The spectral data are in accordance with those reported in the literature.[10]

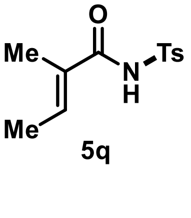

5q

(*E*)-2-methyl-*N*-tosylbut-2-enamide (5q)

^{1}H NMR (400 MHz, CDCl$_3$): δ 9.40 (s, 1 H), 7.97 (d, *J* = 8.0 Hz, 2 H), 7.30 (d, *J* = 8.0 Hz, 2 H), 6.60 (q, *J* = 6.8 Hz, 1 H), 2.39 (s, 3 H), 1.81 – 1.64 (m, 6 H). The spectral data are in accordance with those reported in the literature.[10]

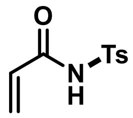

5r

***N*-tosylacrylamide (5r)**

^{1}H NMR (400 MHz, CDCl$_3$): δ 7.95 (d, *J* = 8.0 Hz, 2 H), 7.29 (d, *J* = 8.0 Hz, 2 H), 6.39 (d, *J* = 17.2 Hz, 1 H), 6.26 – 6.12 (m, 1 H), 5.77 (d, *J* = 10.4 Hz, 1 H), 2.38 (s, 3 H). The spectral data are in accordance with those reported in the literature.[21]

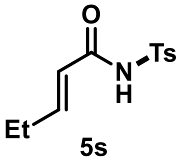

5s

***(E)*-*N*-tosylpent-2-enamide (5s)**

^{1}H NMR (400 MHz, CDCl$_3$): δ 8.01 – 7.92 (m, 2 H), 7.33 (d, *J* = 7.6 Hz, 2 H), 7.09 – 6.94 (m, 1 H), 5.77 – 5.86 (m, 1 H), 2.43 (s, 3 H), 2.25 – 2.13 (m, 2 H), 1.06 – 0.96 (m, 3 H). **^{13}C NMR (100 MHz, CDCl$_3$)** δ 163.2, 152.4, 145.1, 135.6, 129.6, 128.4, 120.2, 25.4, 21.7, 11.9. **IR** (neat): 3233, 2321, 1715, 1646, 1595, 1427, 1336, 1189, 1124, 1081, 1031, 853, 660, 566, 543 cm^{-1}. **HRMS** (ESI-TOF) m/z Calcd for $C_{12}H_{16}O_3NS$ [M]$^+$, 254.0845 found 254.0847**.**

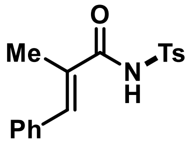

5t

(*E*)-2-methyl-3-phenyl-*N*-tosylacrylamide (5t)

^{1}H NMR (400 MHz, $CDCl_3$): δ 8.06 – 8.00 (m, 2 H), 7.42 – 7.28 (m, 8 H), 2.44 (s, 3 H), 2.06 (d, *J* = 1.4 Hz, 3 H). The spectral data are in accordance with those reported in the literature.[10]

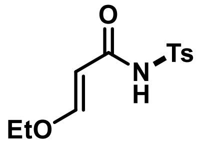

5u

(*E*)-3-ethoxy-*N*-tosylacrylamide (5u)

^{1}H NMR (400 MHz, $CDCl_3$): δ 8.54 (s, 1H), 7.97 – 7.90 (m, 2H), 7.60 (d, *J* = 12.0 Hz, 1H), 7.33 (d, *J* = 8.0 Hz, 2H), 5.28 (d, *J* = 12.0 Hz, 1H), 3.90 (q, *J* = 7.4 Hz, 2H), 2.43 (s, 3H), 1.30 (t, *J* = 7.4 Hz, 3H). The spectral data are in accordance with those reported in the literature.[26]

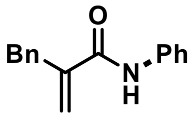

8a

2-Benzyl-N-phenylacrylamide (8a)

^{1}H NMR (400 MHz, $CDCl_3$): δ 7.44 (d, *J* = 7.6 Hz, 2 H), 7.36 – 7.22 (m, 6 H), 7.14 – 7.03 (m, 1 H), 5.89 (s, 1 H), 5.39 (s, 1 H), 3.75 (s, 2 H). The spectral data are in accordance with those reported in the literature.[22]

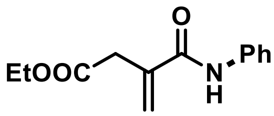

8b

Ethyl 3-(phenylcarbamoyl)but-3-enoate (8b)

The representative synthetic procedure B was followed. Purification by column chromatography (*n*-hexane/EtOAc: 3/1) yielded **4c** as a white solid. **M. p.: 178.4 – 184.7 ℃. ^{1}H NMR (400 MHz, CDCl$_3$):** δ 8.32 (s, 1 H), 7.56 (d, *J* = 7.2 Hz, 2 H), 7.34 – 7.27 (m, 2 H), 7.14 – 7.04 (m, 1 H), 5.94 (s, 1 H), 5.55 (s, 1 H), 4.17 (q, *J* = 7.2 Hz, 2 H), 3.53 – 3.33 (m, 2 H), 1.26 (t, *J* = 7.2 Hz, 3 H). **^{13}C NMR (100 MHz, CDCl$_3$)**: δ 171.4, 165.8, 138.8, 137.8, 128.9, 124.3, 122.5, 120.0, 61.3, 38.4, 14.0. **IR** (neat): 3324, 2981, 1715, 1663, 1628, 1597, 1532, 1495, 1440, 1321, 1243, 1182, 1029, 938, 753, 692 cm^{-1}. **HRMS** (ESI-TOF) m/z Calcd for $C_{13}H_{15}NO_3Na$ [M+Na]$^+$, 256.0944 found 256.0945.

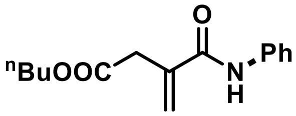

8c

Butyl 3-(phenylcarbamoyl)but-3-enoate (8c)

The representative synthetic procedure B was followed. Purification by column chromatography (*n*-hexane/EtOAc: 3/1) yielded **4d** as a colorless oil. **^{1}H NMR (400 MHz, CDCl$_3$):** δ 8.47 – 8.35 (m, 1 H), 7.56 (d, *J* = 8.0 Hz, 2 H), 7.30 (t, *J* = 8.0 Hz, 2 H), 7.09 (t, *J* = 7.6 Hz, 1 H), 5.93 (s, 1 H), 5.54 (s, 1 H), 4.10 (t, *J* = 6.4 Hz, 2 H), 3.44 (s, 2 H), 1.67 – 1.53 (m, 2 H), 1.40 – 1.27 (m, 2 H), 0.89 (t, *J*

= 7.2 Hz, 3 H). **^{13}C NMR (100 MHz, $CDCl_3$)**: δ 171.5, 165.9, 138.8, 137.8, 128.8, 124.3, 122.4, 120.0, 65.2, 38.3, 30.4, 18.9, 13.5. **IR** (neat): 3325, 2958, 1723, 1668, 1598, 1532, 1438, 1321, 1244, 1180, 940, 750, 654 cm^{-1}. **HRMS** (ESI-TOF) m/z Calcd for $C_{15}H_{19}NO_3Na$ $[M+Na]^+$, 284.1257 found 284.1258.

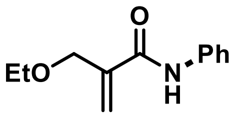

8d

2-(Ethoxymethyl)-N-phenylacrylamide (8d)

The representative synthetic procedure B was followed. Purification by column chromatography (*n*-hexane/EtOAc: 3/1) yielded **4e** as a colorless oil. **^{1}H NMR (400 MHz, $CDCl_3$):** δ 9.15 (s, 1 H), 7.58 (d, *J* = 8.0 Hz, 2 H), 7.33 (t, *J* = 7.8 Hz, 2 H), 7.11 (t, *J* = 7.6 Hz, 1 H), 6.35 (s, 1 H), 5.62 (s, 1 H), 4.32 (s, 2 H), 3.61 (q, *J* = 7.0 Hz, 2 H), 1.31 (t, *J* = 7.0 Hz, 3 H). **^{13}C NMR (100 MHz, $CDCl_3$)**: δ 164.3, 138.8, 138.0, 129.0, 126.4, 124.2, 119.9, 71.1, 65.7, 15.1. **IR** (neat): 3305, 2974, 2867, 1670, 1598, 1534, 1495, 1439, 1319, 1246, 1084, 949, 751, 690 cm^{-1}. **HRMS** (ESI-TOF) m/z Calcd for $C_{12}H_{15}NO_2Na$ $[M+Na]^+$, 228.0995 found 228.0994.

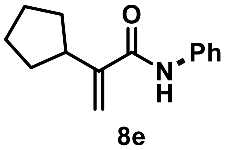

8e

2-Cyclopentyl-N-phenylacrylamide (8e)

The representative synthetic procedure B was followed. Purification by column

chromatography (*n*-hexane/EtOAc: 3/1) yielded **4f** as a white solid. **M. p.: 93.7 – 94.7 ℃. ^{1}H NMR (400 MHz, $CDCl_3$):** δ 7.61 (s, 1 H), 7.59 – 7.53 (m, 2 H), 7.37 – 7.28 (m, 2 H), 7.15 – 7.06 (m, 1 H), 5.61 (s, 1 H), 5.35 (d, *J* = 1.6 Hz, 1 H), 3.00 – 2.89 (m, 1 H), 2.01 – 1.89 (m, 2 H), 1.77 – 1.58 (m, 4 H), 1.51 – 1.41 (m, 2 H). **^{13}C NMR (100 MHz, $CDCl_3$)**: δ 168.0, 150.4, 137.9, 128.9, 124.3, 119.9, 114.8, 41.9, 31.5, 24.8. **IR** (neat): 3237, 2949, 2867, 1650, 1594, 1533, 1493, 1437, 1318, 1254, 920, 751, 692 cm^{-1}. **HRMS** (ESI-TOF) m/z Calcd for $C_{14}H_{18}NO$ $[M+H]^+$, 216.1383 found 216.1377.

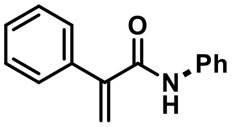

8f

***N*,2-diphenylacrylamide (8f)**

^{1}H NMR (400 MHz, $CDCl_3$): δ 7.52 (d, *J* = 8.0 Hz, 2 H), 7.49 – 7.38 (m, 5 H), 7.32 (t, *J* = 8.0 Hz, 2 H), 7.16 – 7.09 (m, 1 H), 6.37 – 6.25 (s, 1 H), 5.73 (s, 1 H). The spectral data are in accordance with those reported in the literature.[23]

4. Optimization of the Reaction Conditions

Supplementary Table 1: Optimization of Time[a]

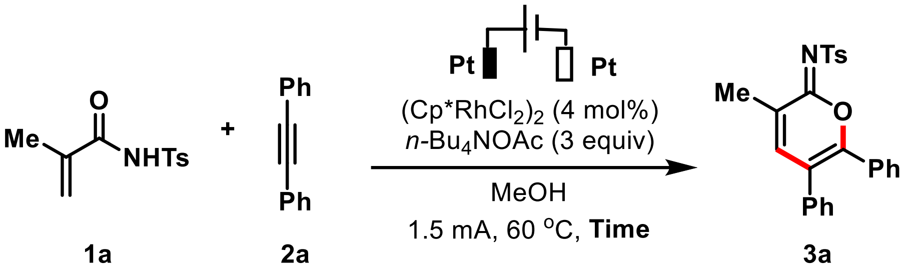

Entry	Time(h)	Yield (%)[b]
1	2	36
2	4	54
3	6	79
4	7	99
5	8	95

[a]Reaction conditions: **1a** (0.30 mmol), **2a** (0.20 mmol), $(Cp^*RhCl_2)_2$ (4 mol%), *n*-Bu_4NOAc (3.0 equiv) and solvent (3 mL), in an undivided cell with two platinum electrodes (each 1.0 × 1.0 cm^2), 60 °C, 1.5 mA. [b]Yield was determined by ^1H NMR with 1,4-Dimethoxybenzene as the internal standard.

Supplementary Table 2: Optimization of Solvents[a]

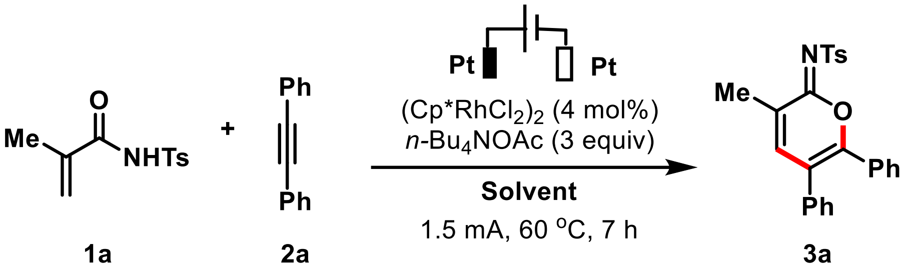

Entry	Solvent	Yield (%)[b]
1	MeOH	99 (91)[c]
2	EtOH	99
3	HFIP	9
4	CF_3CH_2OH	17
5	CH_3CN	98
6	DMF	20

[a]Reaction conditions: **1a** (0.30 mmol), **2a** (0.20 mmol), $(Cp^*RhCl_2)_2$ (4 mol%), *n*-Bu_4NOAc (3.0 equiv) and solvent (3 mL), in an undivided cell with two platinum electrodes (each 1.0 × 1.0 cm^2), 60 °C, 1.5 mA, 7 h. [b]Yield was determined by ^1H NMR with 1,4-Dimethoxybenzene as the internal standard. [c]Isolated yield

Supplementary Table 3: Optimization of Bases [a]

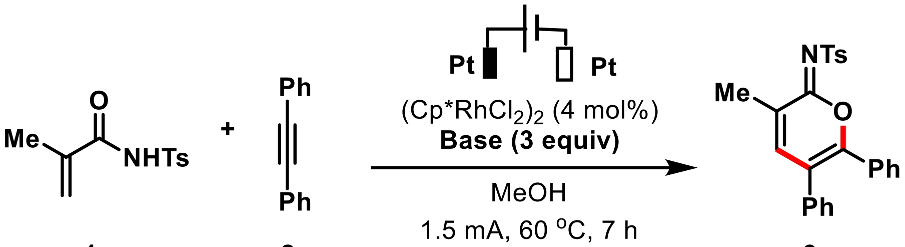

Entry	Base	Yield (%)[b]
1	KOAc	94
2	KOPiv	80
3	NaOAc	86
4	NaOPiv·H_2O	82
5	NaOTf	27
6	K_2CO_3	N.D.[d]
7	*n*-Bu_4NOAc	99(91)[c]
8	*n*-Bu_4NBF_4	7
9	*n*-Bu_4NClO_4	24
10	*n*-Bu_4NI	N.D.[d]
11	*n*-Bu_4NPF_6	17
12	KPF_6	28

[a]Reaction conditions: **1a** (0.30 mmol), **2a** (0.20 mmol), $(Cp^*RhCl_2)_2$ (4 mol%), *n*-Bu_4NOAc (3.0 equiv) and solvent (3 mL), in an undivided cell with two platinum electrodes (each 1.0 × 1.0 cm^2), 60 °C, 1.5 mA, 7 h. [b]Yield was determined by ^1H NMR with 1,4-Dimethoxybenzene as the internal standard.

[c]Isolated yield. [d]No detected.

Supplementary Table 4: Optimization of Current[a]

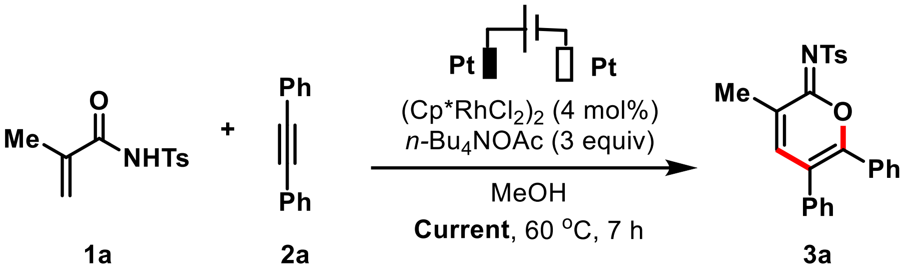

1a 2a 3a

Entry	Current (mA)	Time (h)	Yield(%) [c]
1	0	7	trace
1	1.0	2	20
2	1.5	2	28
3	1.5 [b]	7	NR
4	3.0	2	57
5	5.0	2	94
6	10.0	2	63

[a]Reaction conditions: **1a** (0.30 mmol), **2a** (0.20 mmol), $(Cp^*RhCl_2)_2$ (4 mol%), *n*-Bu_4NOAc (3.0 equiv) and MeOH (3 mL), in an undivided cell with two platinum electrodes (each 1.0×1.0 cm^2), 60 °C. [b]No $(Cp^*RhCl_2)_2$, [c]Yield was determined by ^1H NMR with 1,4-Dimethoxybenzene as the internal standard.

Supplementary Table 5: Deviation of protecting group[a]

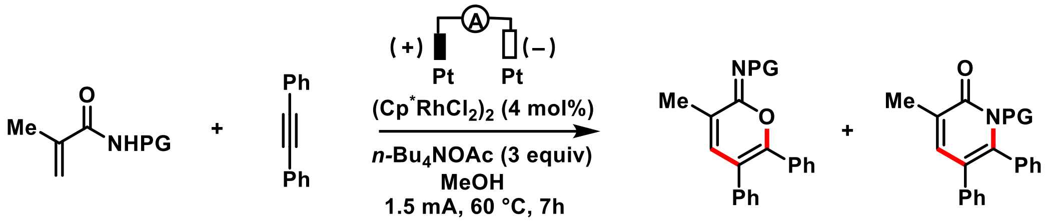

1a-1j 2a 3 4

Entry	protection group	iminoester yield(%)[b]	pyridone yield(%)[b]
1	NO PG	-	-
2	(1a) Ts	91	-
3	(1b) Ph	-	95
4	(1c) *p*-NO_2-C_6H_4	< 5%	86
5	(1d) *p*-OMe-C_6H_4	< 5%	42
6	(1e) *p*-Cl-C_6H_4	< 5%	83
7	(1f) *p*-Me-C_6H_4	< 5%	49
8	(1g) Ms	65	-
9	(1h) $C_6H_5SO_2$	88	-
10	(1i) *p*-Cl-$C_6H_4SO_2$	46	-
11	(1j) Bn	-	-

[a]Reaction conditions: Acryclic amide **(1a–1j)** (0.30 mmol), alkyne **2a** (0.20 mmol), $(Cp^*RhCl_2)_2$ (4 mol%), *n*-Bu_4NOAc (3.0 equiv) and MeOH (3 mL), in an undivided cell with two platinum electrodes (each 1.0 × 1.0 cm^2), 60 °C, 1.5 mA, 7h. [b]Isolated Yield

Supplementary Table 6: Optimization of temperature[a]

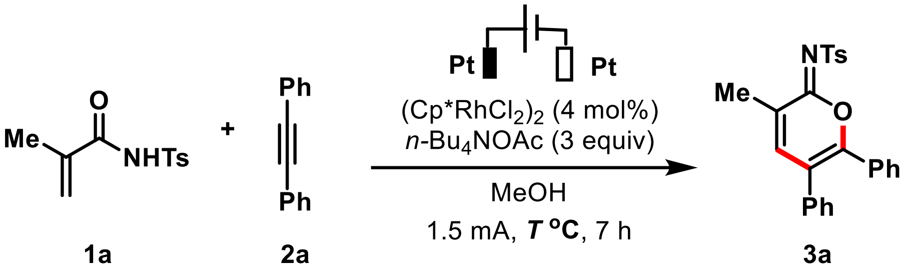

1a 2a 3a

Entry	T/℃	Yield (%)[b]
1	60	91
2	25 (room temperature)	93
3	IKA equipment[c]	92

[a]Reaction conditions: **1a** (0.30 mmol), **2a** (0.20 mmol), $(Cp^*RhCl_2)_2$ (4 mol%), *n*-Bu_4NOAc (3.0 equiv) and MeOH (3 mL), in an undivided cell with two platinum electrodes (each 1.0 × 1.0 cm^2). [b]Isolated Yield. [c]**1a** (0.30 mmol), **2a** (0.20 mmol), $(Cp^*RhCl_2)_2$ (4 mol%), *n*-Bu_4NOAc (3.0 equiv) and MeOH (6 mL), in an undivided cell with two platinum electrodes (each 0.8 × 3.0 cm^2).

Supplementary Table 7: Optimization of electrode[a]

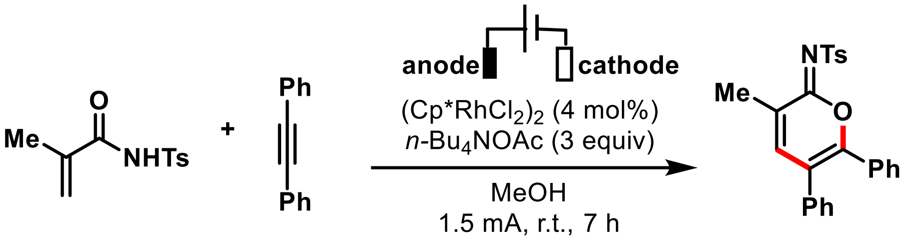

1a 2a 3a

Entry	electrode	Yield (%)[b]
1	Pt(+)‖Pt(-)	99(92)[c]
2	Graphite(+)‖Pt(-)	91
3	Glassy Carbon(+)‖Pt(-)	96
4	RVC(+)‖Pt(-)	84
5	BDD(+) ‖ Pt(-)	99
6	Graphite (+)‖Graphite (-)	95

[a]Reaction conditions: **1a** (0.30 mmol), **2a** (0.20 mmol), $(Cp*RhCl_2)_2$ (4 mol%), *n*-Bu_4NOAc (3.0 equiv) and MeOH (3 mL), in an undivided cell with two electrodes (each 0.8 × 3.0 cm^2). [b]Yield was determined by ^1H NMR with 1,4-Dimethoxybenzene as the internal standard. [c]Isolated Yield.

5. General Procedure for Rh(III) Catalyzed Annulation of Acrylic Amide with Alkyne

Representative Procedure A: Rhodium-catalyzed oxidative annulation

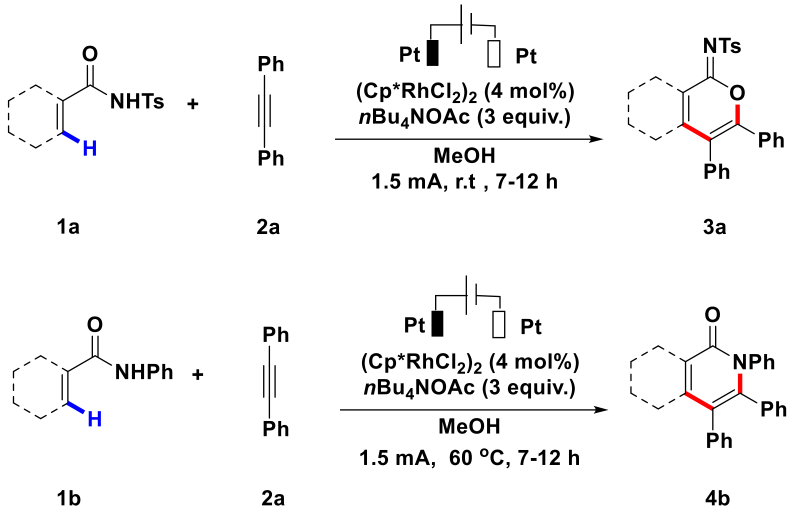

The electrocatalysis can carry out in ***IKA ElectraSyn 2.0*** equipped with two platinum electrodes (each 0.8 × 3.0 cm^2). Acrylic amide **1a** (0.30 mmol, 1.5 equiv), alkyne **2a** (0.2 mmol, 1.0 equiv), *n*-Bu_4NOAc (180.9 mg, 0.6 mmol, 3.0 equiv) and $(Cp^*RhCl_2)_2$ (5.0 mg, 4.0 mol %, 99 wt.%) were dissolved in MeOH (6.0 mL). Electrocatalysis was performed at room temperature with a constant current of 1.5 mA maintained for 7 – 12 h (2.0 – 3.4 F mol^{-1}). After the reaction, the reaction mixture was concentrated in vacuo. The resulting residue was purified by silica gel flash chromatography to give the product **3a**.

Also, the electrocatalysis was carried out in an undivided cell equipped with two platinum electrodes (each 1.0 × 1.0 cm^2). Acrylic amide **1b** (0.30 mmol, 1.5 equiv), alkyne **2a** (0.2 mmol, 1.0 equiv), *n*-Bu_4NOAc (180.9 mg, 0.6 mmol, 3.0 equiv) and $(Cp^*RhCl_2)_2$ (5.0 mg, 4.0 mol %, 99 wt.%) were dissolved in MeOH (3.0 mL). Electrocatalysis was performed 60 ℃ with a

constant current of 1.5 mA maintained for 7 – 12 h (2.0 – 3.4 F mol^{-1}). After the reaction, the reaction mixture was concentrated in vacuo. The resulting residue was purified by silica gel flash chromatography to give the product **4b**.

Representative Procedure for a Gram-Scale Experiment

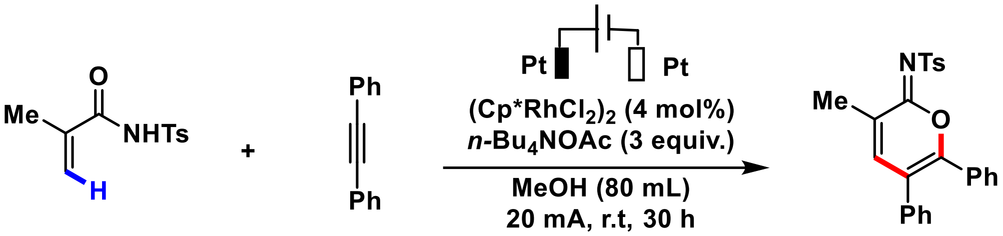

1a (1.5 equiv.) 2a (4 mmol, 0.71g) 3a (1.42 g, 86%)

The gram scale reaction was conducted in a 100 mL beaker-type cell equipped with two platinum electrodes (4.0 × 4.0 cm^2). N-tosylmethacrylamide **1a** (1.5 equiv), 1,2-diphenylethyne **2a** (0.71g, 4 mmol, 1.0 equiv), n-Bu_4NOAc (3.62 g, 12 mmol, 3.0 equiv) and $(Cp^*RhCl_2)_2$ (98.0 mg, 4 mol %, 99 wt.%) and MeOH (80 mL) were added to the electrochemical cell. Electrocatalysis was proformed at room temperature with a constant current of 20 mA maintained for 30 h (5.6 F mol^{-1}). After the reaction, the reaction mixture was concentrated in vacuo. The resulting residue was purified by silica gel flash chromatography to give the product **3a** as yellow solid with 86% yield.

6. Graphical Guide for Rhodium-Catalyzed Oxidative Annulation of Acrylic Amides with Alkynes

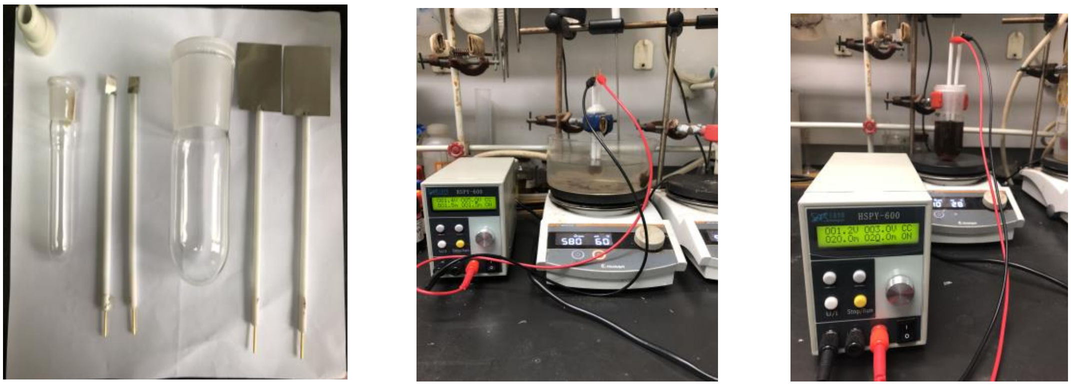

Supplementary Figure 1. **General reaction equipment. *(Left)* General equipment for electrolysis *(Middle)*: General reaction setup. *(Right)*: Gram Scale reaction.**

Alternatively, IKA ElectraSyn 2.0 can be used to conduct this reaction at room temperature. The cell assembly process is depicted below:

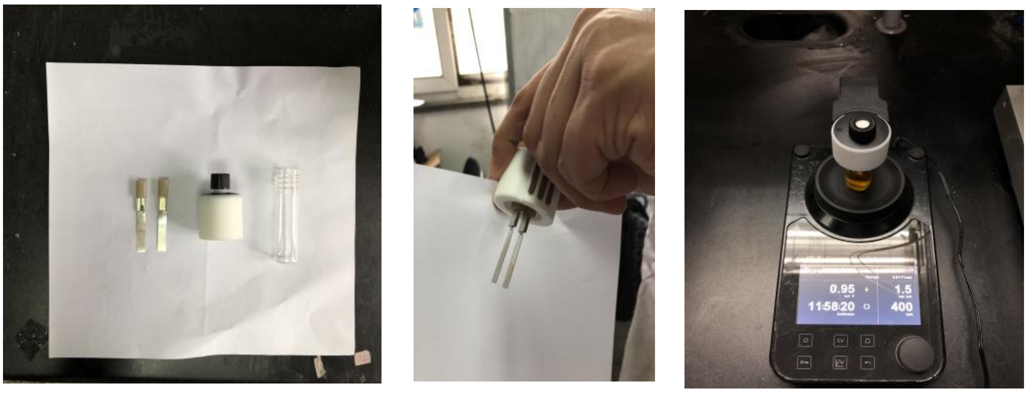

Supplementary Figure 2. IKA ElectraSyn 2.0. (Left) **General equipment for *IKA ElectraSyn 2.0 (Middle)*: Platinum electrodes setup. *(Right)*: The reaction mixture was subjected to constant current electrolysis (I = 1.5mA) using an *IKA ElectraSyn* 2.0**

7. Table of Failed Examples

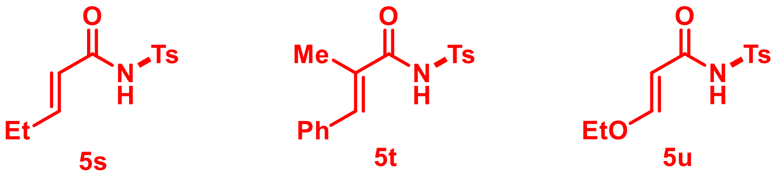

unsuccessful result

8. Characterization Data for the Products

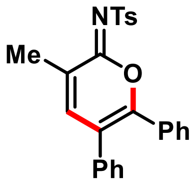

3a

(*Z*)-4-methyl-*N*-(3-methyl-5,6-diphenyl-2*H*-pyran-2-ylidene)benzenesulfonamide (3a)

The representative procedure A was followed using acrylic amide **1a** (71.7 mg, 0.3 mmol) and diphenylacetylene **2a** (35.6 mg, 0.2 mmol). Purification by column chromatography (*n*-hexane/EtOAc: 10/1) yielded **3a** (76.5 mg, 92%) as a yellow solid. **M. p.**: 179.8 – 244.7 ℃. **^{1}H NMR (400 MHz, CDCl$_3$)**: δ 7.89 (d, *J* = 8.0 Hz, 2 H), 7.46 – 7.43 (m, 2 H), 7.38 – 7.28 (m, 7 H), 7.17 – 7.12 (m, 4 H), 2.34 (s, 3 H), 2.19 (s, 3 H). **^{13}C NMR (100 MHz, CDCl$_3$)**: δ 160.1, 155.8, 143.6, 142.7, 139.1, 135.6, 130.7, 130.2, 129.5, 129.1, 129.0, 128.9, 128.3, 128.3, 127.1, 125.9, 120.1, 21.5, 17.0. **IR** (neat): 1640, 1571, 1524, 1484, 1441, 1298, 1151, 1079, 875, 772, 694, 663, 602, 553 cm^{-1}. **HRMS** (ESI-TOF) m/z Calcd for $C_{25}H_{21}NO_3NaS$ [M+Na]$^+$ 438.1134, found 438.1142

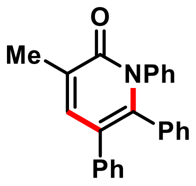

4b

3-Methyl-1,5,6-triphenylpyridin-2(1*H*)-one (4b)

The representative procedure A was followed using phenylacrylic amide **1b** (48.4 mg, 0.3 mmol) and 1,2-diphenylethyne **2a** (35.7 mg, 0.2 mmol) at 60 ℃. Purification by column chromatography (*n*-hexane/EtOAc: 10/1) yielded **4b** (64.0 mg, 95%) as a white solid. **^{1}H NMR (400 MHz, CDCl$_3$)**: δ 7.50 – 7.43 (m, 1 H), 7.25 – 7.18 (m, 2 H), 7.17 – 7.09 (m, 4 H), 7.18 – 6.99 (m, 3 H), 6.99 – 6.91 (m, 2 H), 6.89 – 6.83 (m, 2 H), 2.29 (s, 3 H). **^{13}C NMR (100 MHz, CDCl$_3$)**: δ 163.0, 143.7, 140.0, 139.3, 138.6, 134.2, 131.0, 129.6, 129.2, 129.1, 128.5, 127.9, 127.6, 127.4, 126.3, 119.7, 17.2. The spectral data are in accordance with those reported in the literature.[24]

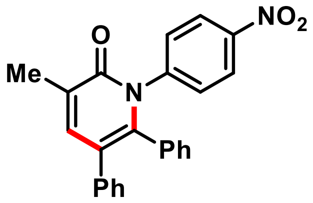

4c

3-Methyl-1-(4-nitrophenyl)-5,6-diphenylpyridin-2(1*H*)-one (4c)

The representative procedure A was followed using acrylic amide **1c** (61.9 mg, 0.3 mmol) and diphenylacetylene **2a** (35.7 mg, 0.2 mmol) at 60 ℃. Purification by column chromatography (*n*-hexane/EtOAc: 10/1) yielded **4c** (66.1 mg, 86%)

as a yellow oil. **^{1}H NMR (400 MHz, CDCl$_3$)**: δ 8.13 – 8.02 (m, 2 H), 7.51 – 7.45(m, 1 H), 7.28 – 7.20 (m, 2 H), 7.17 – 7.10 (m, 3 H), 7.03 – 6.94 (m, 5 H), 6.88 – 6.81 (m, 2 H), 2.26 (d, *J* = 1.2 Hz, 3 H). **^{13}C NMR (100 MHz, CDCl$_3$)**: δ 162.5, 146.6, 145.2, 142.7, 140.6, 140.5, 137.9, 133.4, 130.8, 130.5, 129.4, 129.3, 128.3, 128.0, 127.9, 126.6, 123.8, 123.8, 120.4, 17.1. The spectral data are in accordance with those reported in the literature.[24]

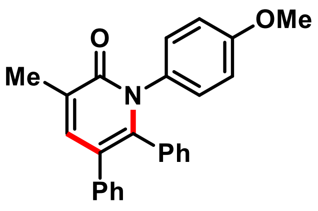

4d

1-(4-Methoxyphenyl)-3-methyl-5,6-diphenylpyridin-2(1*H*)-one (4d)

The representative procedure A was followed using acrylic amide **1d** (57.4 mg, 0.3 mmol) and diphenylacetylene **2a** (35.7 mg, 0.2 mmol) at 60 ℃. Purification by column chromatography (*n*-hexane/EtOAc: 10/1) yielded **4d** (31.2 mg, 42%) as a brown oil. **^{1}H NMR (400 MHz, CDCl$_3$)**: δ 7.46 – 7.43(m, 1 H), 7.17 – 7.07 (m, 3 H), 7.02 – 6.93 (m, 7 H), 6.88 – 6.83 (m, 2 H), 6.75 – 6.69 (m, 2 H), 3.71 (s, 3 H), 2.27 (d, *J* = 1.1 Hz, 3 H). **^{13}C NMR (100 MHz, CDCl$_3$)**: δ 163.3, 158.5, 144.1, 139.9, 138.7, 134.3, 132.1, 131.0, 130.0, 129.5, 128.9, 127.8, 127.5, 127.4, 126.3, 119.7, 113.8, 55.2, 17.2. The spectral data are in accordance with those reported in the literature.[24]

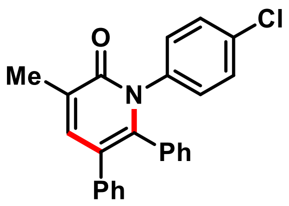

4e

1-(4-Chlorophenyl)-3-methyl-5,6-diphenylpyridin-2(1*H*)-one (4e)

The representative procedure A was followed using acrylic amide **1e** (58.7 mg, 0.3 mmol) and diphenylacetylene **2a** (35.7 mg, 0.2 mmol) at 60 ℃. Purification by column chromatography (*n*-hexane/EtOAc: 10/1) yielded **4e** (61.6 mg, 83%) as a white solid. **^{1}H NMR (400 MHz, CDCl$_3$)**: δ 7.48 – 7.45 (m, 1 H), 7.21 – 7.16 (m, 2 H), 7.16 – 7.09 (m, 3 H), 7.03 – 6.96 (m, 7 H), 6.87 – 6.83 (m, 2 H), 2.27 (d, *J* = 1.2 Hz, 3 H). **^{13}C NMR (100 MHz, CDCl$_3$)**: δ 162.8, 143.4, 140.1, 140.1, 138.3, 137.8, 133.8, 133.4, 130.8, 130.4, 129.5, 129.0, 128.7, 127.9, 127.8, 127.6, 126.4, 119.9, 17.1. The spectral data are in accordance with those reported in the literature.[24]

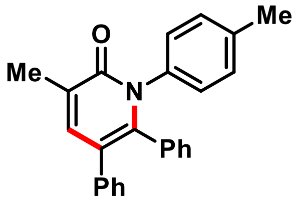

4f

3-Methyl-5,6-diphenyl-1-(p-tolyl)pyridin-2(1*H*)-one (4f)

The representative procedure A was followed using acrylic amide **1f** (52.6 mg, 0.3 mmol) and diphenylacetylene **2a** (35.7 mg, 0.2 mmol) at 60 ℃. Purification by column chromatography (*n*-hexane/EtOAc: 10/1) yielded **4f** (34.6 mg, 49%)

as a white solid. **^{1}H NMR (400 MHz, CDCl$_3$)**: δ 7.47 – 7.44 (m, 1 H), 7.14 – 7.09 (m, 3 H), 7.02 – 6.95 (m, 7 H), 6.94 – 6.90 (m, 2 H), 6.88 – 6.84 (m, 2 H), 2.27 (d, *J* = 1.2 Hz, 3 H), 2.23 (s, 3 H). **^{13}C NMR (100 MHz, CDCl$_3$)**: δ 163.2, 143.9, 140.0, 138.6, 137.3, 136.6, 134.3, 131.0, 129.6, 129.2, 128.9, 128.7, 127.8, 127.5, 127.4, 126.3 119.8, 21.0, 17.2. The spectral data are in accordance with those reported in the literature.[24]

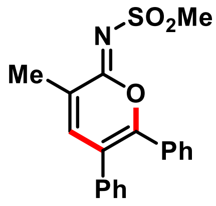

4g

(*Z*)-*N*-(3-methyl-5,6-diphenyl-2*H*-pyran-2-ylidene)methanesulfonamide (4g)

The representative procedure A was followed using acrylic amide **1g** (48.9 mg, 0.3 mmol) and diphenylacetylene **2a** (35.7 mg, 0.2 mmol) at 60 ℃. Purification by column chromatography (*n*-hexane/EtOAc: 10/1) yielded **4g** (44.6 mg, 65%) as a white solid. **M. p.**: 174.5 – 178.3 ℃. **^{1}H NMR (400 MHz, CDCl$_3$)**: δ 7.53 – 7.47 (m, 2 H), 7.38 – 7.33 (m, 3 H), 7.32 – 7.17 (m, 6 H), 3.14 (s, 3 H), 2.18 (s, 3 H). **^{13}C NMR (100 MHz, CDCl$_3$)**: δ 160.2, 155.3, 143.8, 135.6, 130.6, 130.2, 129.1, 128.8, 128.3, 128.3, 125.2, 119.8, 42.1, 16.7. **IR** (neat): 2916, 1640, 1576, 1530, 1284, 1112, 968, 877, 769, 691, 591, 522 cm^{-1}. **HRMS** (ESI-TOF) m/z Calcd for $C_{19}H_{18}NO_3S$ [M+H]$^+$ 340.1002, found 340.0995

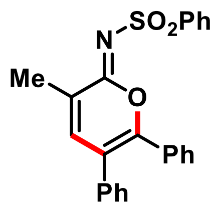

4h

(*Z*)-*N*-(3-methyl-5,6-diphenyl-2*H*-pyran-2-ylidene)benzenesulfonamide (4h)

The representative procedure A was followed using acrylic amide **1h** (67.5 mg, 0.3 mmol) and diphenylacetylene **2a** (35.7 mg, 0.2 mmol) at 60 ℃. Purification by column chromatography (*n*-hexane/EtOAc: 10/1) yielded **4h** (70.5 mg, 88%) as a yellow solid. **M. p.**: 161.7 – 164.1 ℃. **^{1}H NMR (400 MHz, $CDCl_3$)**: δ 8.00 (d, *J* = 7.6 Hz, 2 H), 7.47 – 7.39 (m, 3 H), 7.38 – 7.24 (m, 10 H), 7.15 (d, *J* = 4.4 Hz, 2 H), 2.20 (s, 3 H). **^{13}C NMR (100 MHz, $CDCl_3$)**: δ 160.3, 155.7, 143.8, 141.8, 135.4, 132.0, 130.5, 130.2, 129.3, 129.0, 128.9, 128.3, 128.3, 128.2, 126.9, 125.7, 120.2, 16.9. **IR** (neat): 1637, 1568, 1519, 1483, 1439, 1299, 1145, 1081, 878, 777, 693, 595 565 cm^{-1}. **HRMS** (ESI-TOF) m/z Calcd for $C_{24}H_{20}NO_3S$ $[M+H]^+$ 402.1158, found 402.1160

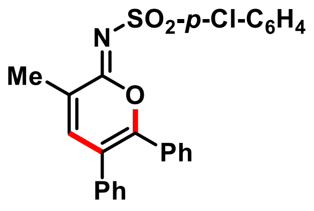

4i

(*Z*)-4-chloro-*N*-(3-methyl-5,6-diphenyl-2*H*-pyran-2-ylidene)benzenesulfona mide (4i)

The representative procedure A was followed using acrylic amide **1i** (77.7 mg,

0.3 mmol) and diphenylacetylene **2a** (35.7 mg, 0.2 mmol) at 60 ℃. Purification by column chromatography (*n*-hexane/EtOAc: 10/1) yielded **4i** (40.5 mg, 46%) as a yellow semisolid. **^{1}H NMR (400 MHz, $CDCl_3$)**: δ 7.93 (d, *J* = 9.2 Hz, 2 H), 7.44 (d, *J* = 7.6 Hz, 2 H), 7.40 – 7.25 (m, 10 H), 7.18 – 7.12 (m, 2 H), 2.19 (s, 3 H). **^{13}C NMR (100 MHz, $CDCl_3$)**: δ 160.4, 155.9, 144.1, 140.4, 138.4, 135.3, 130.5, 130.4, 129.4, 129.1, 128.9, 128.6, 128.5, 128.4, 128.3, 125.7, 120.4, 16.9. **IR** (neat): 1639, 1572, 1525, 1482, 1298, 1147, 1081, 875, 761, 694, 638, 581 cm^{-1}. **HRMS** (ESI-TOF) m/z Calcd for $C_{24}H_{19}NO_3SCl$ $[M+H]^+$ 436.0769, found 436.0759

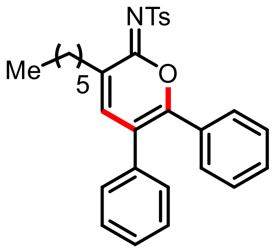

6a

(*Z*)-N-(3-hexyl-5,6-diphenyl-2*H*-pyran-2-ylidene)-4-methylbenzenesulfonamide (6a)

The representative procedure A was followed using acrylic amide **5a** (92.5 mg, 0.3 mmol) and diphenylacetylene **2a** (35.6 mg, 0.2 mmol). Purification by column chromatography (*n*-hexane/EtOAc: 10/1) yielded **6a** (95.3 mg, 99%) as a yellow solid. **M. p.**: 131.0 – 132.0 ℃. **^{1}H NMR (400 MHz, $CDCl_3$)**: δ 7.88 (d, *J* = 8.4 Hz, 2 H), 7.47 – 7.44 (m, 2 H), 7.37 – 7.32 (m, 4 H), 7.30 – 7.25 (m, 3 H), 7.17 – 7.15 (m, 2 H), 7.12 (d, *J* = 8.4 Hz, 2 H), 2.55 (t, *J* = 7.6 Hz, 2 H),

2.33 (s, 3 H), 1.66 – 1.58 (m, 2 H), 1.38 – 1.25 (m, 6 H), 0.88 (t, *J* = 8.0 Hz, 2 H). **^{13}C NMR (100 MHz, CDCl$_3$)**: δ 159.7, 155.4, 142.6, 142.5, 139.2, 135.6, 130.6, 130.1, 129.9, 129.4, 129.0, 128.9, 128.2, 128.2, 126.9, 120.0, 31.5, 30.0, 28.8, 27.4, 22.4, 21.4, 14.0. **IR** (neat): 2922, 1640, 1578, 1533, 1486, 1451, 1289, 1081, 879, 782, 681, 546 cm^{-1}. **HRMS** (ESI-TOF) m/z Calcd for $C_{30}H_{31}NO_3NaS$ [M+Na]$^+$ 508.1917, found 508.1909

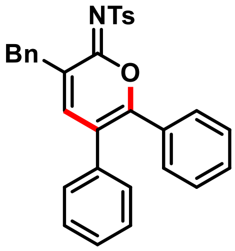

6b

(*Z*)-*N*-(3-benzyl-5,6-diphenyl-2*H*-pyran-2-ylidene)-4-methylbenzenesulfonamide (6b)

The representative procedure A was followed using acrylic amide **5b** (94.5 mg, 0.3 mmol) and diphenylacetylene **2a** (35.6 mg, 0.2 mmol). Purification by column chromatography (*n*-hexane/EtOAc: 10/1) yielded **6b** (82.9 mg, 84%) as a yellow solid. **M**. **p**.: 219.7 – 220.6 ℃. **^{1}H NMR (400 MHz, CDCl$_3$)**: δ 7.85 (d, *J* = 8.0 Hz, 2 H), 7.44 (d, *J* = 7.6 Hz, 2 H), 7.37 – 7.20 (m, 11 H), 7.13 (d, *J* = 8.0 Hz, 2 H), 7.10 – 7.05 (m, 3 H), 3.86 (s, 2 H), 2.33 (s, 3 H) **^{13}C NMR (100 MHz, CDCl$_3$)**: δ 159.3, 155.8, 143.2, 142.7, 139.1, 137.1, 135.4, 130.5, 130.3, 129.4, 129.4, 129.1, 129.0, 128.9, 128.9, 128.6, 128.2, 128.2, 126.9, 126.8, 120.0, 36.0, 21.4. **IR** (neat): 1637, 1570, 1524, 1483, 1314, 1150, 1081, 878,

799, 695, 648, 550 cm^{-1}. **HRMS** (ESI-TOF) m/z Calcd for $C_{31}H_{25}NO_3NaS$ $[M+Na]^+$ 514.1447, found 514.1452

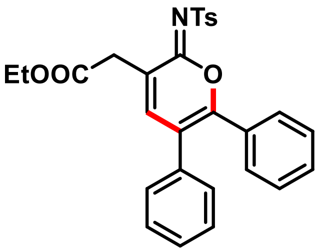

6c

Ethyl (*Z*)-2-(5,6-diphenyl-2-(tosylimino)-2*H*-pyran-3-yl)acetate (6c)

The representative procedure A was followed using acrylic amide **5c** (93.4 mg, 0.3 mmol) and diphenylacetylene **2a** (35.6 mg, 0.2 mmol). Purification by column chromatography (*n*-hexane/EtOAc: 10/1) yielded **6c** (91.2 mg, 93%) as a yellow solid. **M. p.**: 171.1 – 172.1 ℃. **^{1}H NMR (400 MHz, $CDCl_3$)**: δ 7.85 (d, *J* = 8.0 Hz, 2 H), 7.49 – 7.44 (m, 3 H), 7.39 – 7.27 (m, 6 H), 7.21 – 7.16 (m, 2 H), 7.14 (d, *J* = 8.0 Hz, 2 H), 4.15 (q, *J* = 7.2 Hz, 2 H), 3.57 (s, 2 H), 2.34 (s, 3 H), 1.25 (t, *J* = 7.2 Hz, 3 H). **^{13}C NMR (100 MHz, $CDCl_3$)**: δ 169.6, 159.0, 156.8, 145.4, 142.8, 139.1, 135.4, 130.5, 130.5, 129.6, 129.1, 129.0, 128.4, 128.3, 126.9, 122.2, 119.9, 61.3, 35.5, 21.5, 14.1. **IR** (neat): 1726, 1526, 1292, 1143, 869, 668, 586 cm^{-1}. **HRMS** (ESI-TOF) m/z Calcd for $C_{28}H_{25}NO_5NaS$ $[M+Na]^+$ 510.1346, found 510.1344

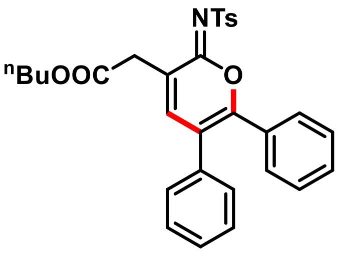

6d

Butyl (*Z*)-2-(5,6-diphenyl-2-(tosylimino)-2*H*-pyran-3-yl)acetate (6d)

The representative procedure A was followed using acrylic amide **5d** (101.8 mg, 0.3 mmol) and diphenylacetylene **2a** (35.6 mg, 0.2 mmol). Purification by column chromatography (*n*-hexane/EtOAc: 10/1) yielded **6d** (96.9 mg, 94%) as a yellow solid. **M. p.**: 119.9 – 125.5 ℃. **^{1}H NMR (400 MHz, CDCl$_3$)**: δ 7.85 (d, *J* = 8.0 Hz, 2 H), 7.50 -7.44 (m, 3 H), 7.40 – 7.28 (m, 6 H), 7.21 – 7.11 (m, 4 H), 4.09 (t, *J* = 6.4 Hz, 2 H), 3.58 (s, 2 H), 2.34 (s, 3 H), 1.64 – 1.55 (m, 2 H), 1.41 – 1.29 (m, 2 H), 0.92 (t, *J* = 7.2 Hz, 3 H). **^{13}C NMR (100 MHz, CDCl$_3$)**: δ 169.7, 159.1, 156.8, 145.4, 142.7, 139.0, 135.4, 130.5, 130.4, 129.5, 129.1, 129.0, 128.4, 128.3, 126.9, 122.2, 119.9, 65.2, 35.4, 30.5, 21.5, 19.1, 13.7. **IR** (neat): 1729, 1577, 1529, 1297, 1146, 1079, 876, 770, 668 cm^{-1}. **HRMS** (ESI-TOF) m/z Calcd for $C_{30}H_{29}NO_5NaS$ [M+Na]$^+$ 538.1659, found 538.1666

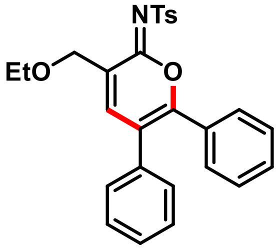

6e

(*Z*)-*N*-(3-(ethoxymethyl)-5,6-diphenyl-2*H*-pyran-2-ylidene)-4-methylbenzen

esulfonamide (6e)

The representative procedure A was followed using acrylic amide **5e** (85.0 mg, 0.3 mmol) and diphenylacetylene **2a** (35.6 mg, 0.2 mmol). Purification by column chromatography (*n*-hexane/EtOAc: 10/1) yielded **6e** (87.2 mg, 95%) as a yellow solid. **M**. **p**.: 186.8 – 217.2 ℃. **^{1}H NMR (400 MHz, CDCl$_3$)**: δ 7.87 (d, *J* = 8.0 Hz, 2 H); 7.62 – 7.61 (m, 1 H), 7.46 – 7.43 (m, 2 H); 7.38 – 7.27 (m, 6 H), 7.18 – 7.16 (m, 2 H), 7.12 (d, *J* = 8.0 Hz, 2 H), 4.43 (d, *J* = 1.6 Hz, 2 H), 3.63 (q, *J* = 8.0 Hz, 2 H), 2.32 (s, 3 H), 1.24 (t, *J* = 8.0 Hz, 3 H). **^{13}C NMR (100 MHz, CDCl$_3$)**: δ 157.5, 156.0, 142.8, 141.5, 138.8, 135.5, 130.5, 130.3, 129.4, 129.0, 128.9, 128.2, 128.2, 127.0, 126.4, 120.2, 66.8, 66.1, 21.4, 15.0. **IR** (neat): 1640, 1574, 1527, 1485, 1305, 1147, 1087, 878, 778, 694, 653, 547 cm^{-1}. **HRMS** (ESI-TOF) m/z Calcd for $C_{27}H_{25}NO_4NaS$ $[M+Na]^+$ 482.1397, found 482.1392

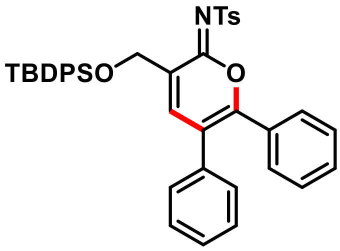

6f

(*Z*)-*N*-(3-(((tert-butyldiphenylsilyl)oxy)methyl)-5,6-diphenyl-2*H*-pyran-2-ylidene)-4-methylbenzenesulfonamide (6f)

The representative procedure A was followed using acrylic amide **5f** (148.1 mg, 0.3 mmol) and diphenylacetylene **2a** (35.6 mg, 0.2 mmol). Purification by

column chromatography (*n*-hexane/EtOAc: 10/1) yielded **6f** (126.0 mg, 94%) as a yellow solid. **M. p.**: 64.7 – 99.7 ℃. **^{1}H NMR (400 MHz, CDCl$_3$)**: δ 7.84 – 7.80 (m, 3 H), 7.69 – 7.66 (m, 4 H), 7.48 – 7.43 (m, 4 H), 7.41 – 7.29 (m, 10 H), 7.20 – 7.18 (m, 2 H), 7.08 (d, *J* = 8.0 Hz, 2 H), 4.73 (s, 2 H), 2.32 (s, 3 H), 1.11 (s, 9 H) **^{13}C NMR (100 MHz, CDCl$_3$)**: δ 157.3, 155.9, 142.7, 141.1, 138.7, 135.6, 135.3, 132.5, 130.5, 130.3, 129.9, 129.5, 129.0, 128.8, 128.8, 128.3, 128.3, 128.2, 127.8, 127.0, 120.1, 60.3, 26.8, 21.3, 19.2. **IR** (neat): 1640, 1575, 1527, 1484, 1307, 1147, 1079, 882, 815, 776, 694 cm^{-1}. **HRMS** (ESI-TOF) m/z Calcd for $C_{41}H_{39}NO_4NaSiS$ [M+Na]$^+$ 692.2261, found 692.2252

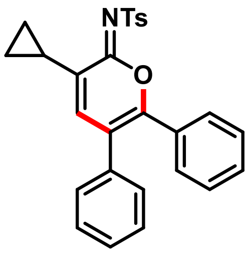

6g

(*Z*)-*N*-(3-cyclopropyl-5,6-diphenyl-2*H*-pyran-2-ylidene)-4-methylbenzenesulfonamide (6g)

The representative procedure A was followed using acrylic amide **5g** (79.6 mg, 0.3 mmol) and diphenylacetylene **2a** (35.6 mg, 0.2 mmol). Purification by column chromatography (*n*-hexane/EtOAc: 10/1) yielded **6g** (84.6 mg, 96%) as a yellow solid. **M. p.**: 193.7 – 194.7 ℃. **^{1}H NMR (400 MHz, CDCl$_3$)**: δ 7.90 (d, *J* = 8.0 Hz, 2 H); 7.45 – 7.43 (m, 2 H), 7.36 – 7.31 (m, 4 H); 7.29 – 7.25 (m, 2 H), 7.14 – 7.11 (m, 4 H), 6.91(s, 1 H), 2.33 (s, 3 H), 2.18 – 2.11 (m, 1 H), 1.04 –

0.99 (m, 2 H), 0.72 – 0.68 (m, 2 H). **^{13}C NMR (100 MHz, CDCl$_3$)**: δ 160.0, 154.7, 142.6, 139.1, 137.9, 135.7, 131.6, 130.5, 130.1, 129.4, 129.0, 128.9, 128.9, 128.3, 128.2, 127.0, 119.9, 21.4, 10.5, 8.5. **IR** (neat): 1635, 1565, 1515, 1484, 1299, 1147, 1082, 840, 798, 698, 555 cm^{-1}. **HRMS** (ESI-TOF) m/z Calcd for $C_{30}H_{29}NO_5NaS$ $[M+Na]^+$ 464.1291, found 464.1290

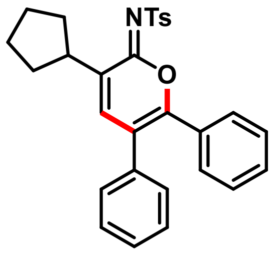

6h

(*Z*)-*N*-(3-cyclopentyl-5,6-diphenyl-2*H*-pyran-2-ylidene)-4-methylbenzenesul fonamide (6h)

The representative procedure A was followed using acrylic amide **5h** (88.0 mg, 0.3 mmol) and diphenylacetylene **2a** (35.6 mg, 0.2 mmol). Purification by column chromatography (*n*-hexane/EtOAc: 10/1) yielded **6h** (82.3 mg, 87%) as a yellow solid. **M. p.**: 182.9 – 247.9 ℃. **^{1}H NMR (400 MHz, CDCl$_3$)**: δ 7.88 (d, *J* = 8.4 Hz, 2 H), 7.47 – 7.45 (m, 2 H), 7.37 – 7.33 (m, 4 H), 7.30 – 7.28 (m, 3 H), 7.18 – 7.15 (m, 2H), 7.12 (d, *J* = 8.0 Hz, 2H) 3.26 – 3.17 (m, 1H), 2.33 (s, 3H), 2.13 – 2.06 (m, 2H), 1.77 – 1.63 (m, 4H), 1.57 – 1.47 (m, 2H) **^{13}C NMR (100 MHz, CDCl$_3$)**: δ 159.7, 155.1, 142.6, 140.2, 139.2, 135.8, 133.4, 130.6, 130.1, 129.4, 129.0, 128.9, 128.9, 128.2, 128.2, 126.9, 120.0, 39.5, 31.8, 25.0, 21.4. **IR** (neat): 1635, 1568, 1526, 1484, 1444, 1298, 1152, 1080, 909, 780, 677

cm^{-1}. **HRMS** (ESI-TOF) m/z Calcd for $C_{29}H_{27}NO_3NaS$ $[M+Na]^+$ 492.1604, found 492.1616

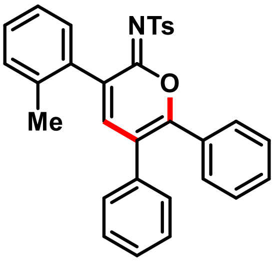

6i

(*Z*)-*N*-(5,6-diphenyl-3-(o-tolyl)-2*H*-pyran-2-ylidene)-4-methylbenzenesulfonamide (6i)

The representative procedure A was followed using acrylic amide **5i** (94.6 mg, 0.3 mmol) and diphenylacetylene **2a** (35.6 mg, 0.2 mmol). Purification by column chromatography (*n*-hexane/EtOAc: 10/1) yielded **6i** (92.8 mg, 95%) as a yellow solid. **M. p.**: 233.7 – 238.1 ℃. **^{1}H NMR (400 MHz, $CDCl_3$)**: δ 7.78 (d, *J* = 8.0 Hz, 2 H), 7.60 – 7.56 (m, 2 H), 7.42 – 7.36 (m, 2 H), 7.36 – 7.31 (m, 5 H), 7.31 – 7.25 (m, 1 H), 7.24 – 7.17 (m, 5 H), 7.09 (d, *J* = 8.0 Hz, 2 H), 2.33 – 2.29 (m, 6 H). **^{13}C NMR (100 MHz, $CDCl_3$)**: δ 158.3, 157.1, 145.9, 142.5, 139.2, 136.8, 135.3, 133.9, 130.6, 130.5, 130.3, 129.8, 129.7, 129.5, 129.1, 128.9, 128.9, 128.8, 128.4, 128.3, 126.8, 125.8, 120.0, 21.4, 20.2. **IR** (neat): 1632, 1567, 1519, 1483, 1442, 1306, 1150, 1084, 761, 548 cm^{-1}. **HRMS** (ESI-TOF) m/z Calcd for $C_{31}H_{25}NO_3NaS$ $[M+Na]^+$ 514.1447, found 514.1442

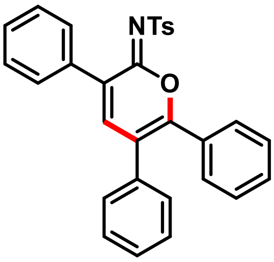

6j

(*Z*)-4-methyl-*N*-(3,5,6-triphenyl-2*H*-pyran-2-ylidene)benzenesulfonamide (6j)

The representative procedure A was followed using acrylic amide **5j** (90.3 mg, 0.3 mmol) and diphenylacetylene **2a** (35.6 mg, 0.2 mmol). Purification by column chromatography (*n*-hexane/EtOAc: 10/1) yielded **6j** (78.8 mg, 82%) as a yellow solid. **M. p.**: 235.4 – 236.5 ℃. **^{1}H NMR (400 MHz, CDCl$_3$)**: δ 7.85 (d, *J* = 8.0 Hz, 2H), 7.69 – 7.63 (m, 2 H), 7.56 (d, *J* = 7.6 Hz, 2 H), 7.52 (s, 1 H), 7.40 – 7.33 (m, 7 H), 7.33 – 7.27 (m, 2 H), 7.26 – 7.19 (m, 2 H), 7.13 (d, *J* = 8.0 Hz, 2 H), 2.33 (s, 3 H). **^{13}C NMR (100 MHz, CDCl$_3$)**: δ 158.4, 156.7, 144.4, 142.6, 139.4, 135.5, 133.8, 130.6, 130.4, 129.6, 129.2, 129.0, 129.0, 128.9, 128.8, 128.5, 128.4, 128.3, 128.0, 126.8, 120.5, 21.4. **IR** (neat): 1630, 1569, 1523, 1484, 1442, 1285, 1153, 1081, 845, 774, 684, 545 cm^{-1}. **HRMS** (ESI-TOF) m/z Calcd for $C_{30}H_{23}NO_3NaS$ [M+Na]$^+$ 500.1291, found 500.1297

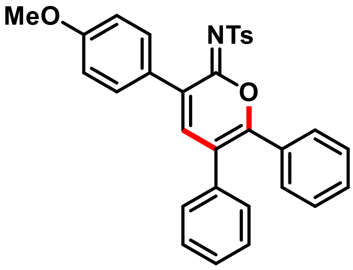

6k

(*Z*)-*N*-(3-(4-methoxyphenyl)-5,6-diphenyl-2*H*-pyran-2-ylidene)-4-methylbenzenesulfonamide (6k)

The representative procedure A was followed using acrylic amide **5k** (99.4 mg, 0.3 mmol), diphenylacetylene **2a** (35.6 mg, 0.2 mmol) and methanol (6 mL). Purification by column chromatography (*n*-hexane/EtOAc: 10/1) yielded **6k** (81.9 mg, 81%) as a yellow solid. **M. p.**: 223.7 – 227.3 ℃. **^{1}H NMR (400 MHz, $CDCl_3$)**: δ 7.87 (d, *J* = 8.0 Hz, 2 H), 7.65 (d, *J* = 8.4 Hz, 2 H), 7.55 (d, *J* = 7.6 Hz, 2 H), 7.49 (s, 1 H), 7.40 – 7.28 (m, 6 H), 7.25 – 7.21 (m, 2 H), 7.14 (d, *J* = 8.0 Hz, 2 H), 6.92 (d, *J* = 8.4 Hz, 2 H), 3.83 (s, 3 H), 2.34 (s, 3 H). **^{13}C NMR (100 MHz, $CDCl_3$)**: δ 160.2, 158.6, 156.2, 143.3, 142.6, 139.4, 135.7, 130.5, 130.4, 130.2, 129.5, 129.2, 129.0, 128.4, 128.3, 127.6, 126.8, 126.1, 120.6, 113.7, 55.3, 21.4. **IR** (neat): 1614, 1564, 1518, 1492, 1449, 1293, 1250, 1150, 1080, 1028, 842, 684, 667, 548 cm^{-1}. **HRMS** (ESI-TOF) m/z Calcd for $C_{31}H_{25}NO_4NaS$ $[M+Na]^+$ 530.1397, found 530.1394

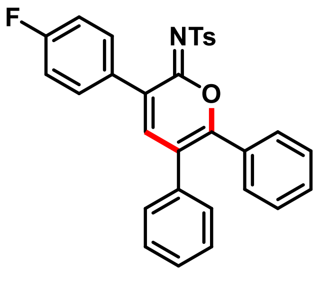

6l

(*Z*)-*N*-(3-(4-fluorophenyl)-5,6-diphenyl-2*H*-pyran-2-ylidene)-4-methylbenzenesulfonamide (6l)

The representative procedure A was followed using acrylic amide **5l** (95.8 mg,

0.3 mmol) and diphenylacetylene **2a** (35.6 mg, 0.2 mmol). Purification by column chromatography (*n*-hexane/EtOAc: 10/1) yielded **6l** (69.7 mg, 70%) as a yellow solid. **M. p.**: 227.6 – 228.5 ℃. **^{1}H NMR (400 MHz, $CDCl_3$)**: δ 7.85 (d, *J* = 8.4 Hz, 2 H), 7.69 – 7.62 (m, 2 H), 7.59 – 7.53 (m, 2 H), 7.50 (s, 1 H), 7.41 – 7.29 (m, 6 H), 7.24-7.22 (m, 2 H), 7.15 (d, *J* = 8.0 Hz, 2 H), 7.10-7.05 (m, 2 H), 2.35 (s, 3 H). **^{13}C NMR (100 MHz, $CDCl_3$)**: δ 163.0 (d, *J* = 248.1 Hz), 158.3, 156.8, 144.2, 142.7, 139.2, 135.4, 130.7 (d, *J* = 8.2 Hz), 130.6, 130.3, 129.8, 129.7, 129.6, 129.2, 129.0, 128.9, 128.5, 128.4, 126.9, 126.8, 120.4, 115.3 (d, *J* = 21.6 Hz), 21.4. **^{19}F NMR (376 MHz, $CDCl_3$)** δ -111.92. **IR** (neat): 1629, 1561, 1513, 1307, 1150, 1080, 843, 810, 768, 695, 656, 619, 546 cm^{-1}. **HRMS** (ESI-TOF) m/z Calcd for $C_{30}H_{22}NO_3FNaS$ $[M+Na]^+$ 518.1197, found 518.1204

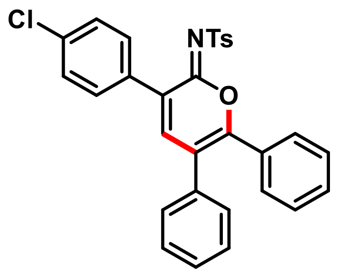

6m

(*Z*)-*N*-(3-(4-chlorophenyl)-5,6-diphenyl-2*H*-pyran-2-ylidene)-4-methylbenzenesulfonamide (6m)

The representative procedure A was followed using acrylic amide **5m** (100.7 mg, 0.3 mmol), diphenylacetylene **2a** (35.6 mg, 0.2 mmol) and methanol (6 mL). Purification by column chromatography (*n*-hexane/EtOAc: 10/1) yielded **6m**

(72.7 mg, 71%) as a yellow solid. **M. p.**: 227.6 – 228.5 ℃. **^{1}H NMR (400 MHz, CDCl$_3$)**: δ 7.86 (d, *J* = 8.0 Hz, 2 H); 7.62 (d, *J* = 8.4 Hz, 2 H), 7.59 – 7.53 (m, 2 H); 7.51 (s, 1 H), 7.41 – 7.28 (m, 8 H), 7.25 – 7.20 (m, 2 H), 7.15 (d, *J* = 8.0 Hz, 2 H), 2.35 (s, 3 H). **^{13}C NMR (100 MHz, CDCl$_3$)**: δ 158.0, 157.0, 144.4, 142.7, 139.2, 135.3, 134.9, 132.2, 130.7, 130.3, 130.1, 129.6, 129.2, 129.0, 128.9, 128.5, 128.5, 128.4, 126.8, 126.7, 120.4, 21.4. **IR** (neat): 1624, 1574, 1518, 1484, 1285, 1082, 847, 767, 677, 543 cm^{-1}. **HRMS** (ESI-TOF) m/z Calcd for $C_{30}H_{22}NO_3NaSCl$ [M+Na]$^+$ 534.0901, found 534.0898

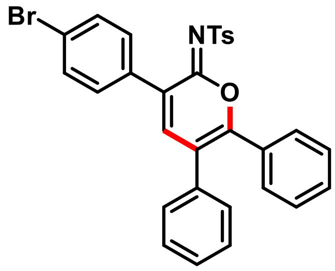

6n

(*Z*)-*N*-(3-(4-bromophenyl)-5,6-diphenyl-2*H*-pyran-2-ylidene)-4-methylbenzenesulfonamide (6n)

The representative procedure A was followed using acrylic amide **5n** (114.1 mg, 0.3 mmol) and diphenylacetylene **2a** (35.6 mg, 0.2 mmol). Purification by column chromatography (*n*-hexane/EtOAc: 10/1) yielded **6n** (92.6 mg, 83%) as a yellow solid. **M. p.**: 225.7 – 238.9 ℃. **^{1}H NMR (400 MHz, CDCl$_3$)**: δ 7.85 (d, *J* = 8.0 Hz, 2H); 7.58 – 7.53 (m, 4 H), 7.52 – 7.48 (m 3 H); 7.41 – 7.34 (m, 4 H), 7.33 – 7.28 (m, 2 H), 7.24 – 7.20 (m, 2 H), 7.15 (d, *J* = 8.0 Hz, 2 H), 2.34 (s, 3 H). **^{13}C NMR (100 MHz, CDCl$_3$)**: δ 158.0, 157.0, 144.4, 142.7, 139.1, 135.2,

132.6, 131.4, 130.7, 130.4, 130.2, 129.5, 129.2, 129.0, 128.9, 128.5, 128.4, 126.7, 126.7, 123.2, 120.4, 21.4. **IR** (neat): 1624, 1575, 1519, 1484, 1285, 1079, 847, 677, 543 cm^{-1}. **HRMS** (ESI-TOF) m/z Calcd for $C_{30}H_{22}NO_3NaSBr$ $[M+Na]^+$ 578.0396, found 578.0397

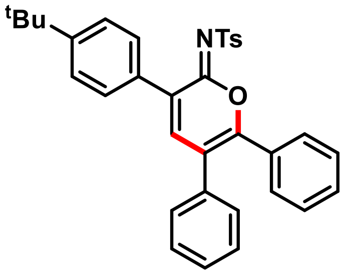

6o

(*Z*)-*N*-(3-(4-(tert-butyl)phenyl)-5,6-diphenyl-2*H*-pyran-2-ylidene)-4-methyl benzenesulfonamide (6o)

The representative procedure A was followed using acrylic amide **5o** (107.1 mg, 0.3 mmol) and diphenylacetylene **2a** (35.6 mg, 0.2 mmol). Purification by column chromatography (*n*-hexane/EtOAc: 10/1) yielded **6o** (88.0 mg, 82%) as a yellow solid. **M**. **p**.: 202.0 – 212.3 ℃. **^{1}H NMR (400 MHz, $CDCl_3$)**: δ 7.88 (d, *J* = 8.0 Hz, 2 H), 7.63 (d, *J* = 8.4 Hz, 2 H), 7.57 – 7.52 (m, 3 H), 7.44 – 7.39 (m, 2 H), 7.39 – 7.28 (m, 6 H), 7.24 – 7.19 (m, 2 H), 7.13 (d, *J* = 8.0 Hz, 2 H), 2.34 (s, 3 H), 1.34 (s, 9 H). **^{13}C NMR (100 MHz, $CDCl_3$)**: δ 158.6, 156.5, 152.2, 144.0, 142.6, 139.3, 135.6, 130.8, 130.5, 130.5, 129.6, 129.2, 129.0, 128.5, 128.4, 128.3, 127.9, 126.9, 125.3, 120.6, 34.7, 31.2, 21.4. **IR** (neat): 2956, 1628, 1567, 1521, 1483, 1289, 1083, 1022, 844, 800, 680, 553 cm^{-1}. **HRMS** (ESI-TOF) m/z Calcd for $C_{34}H_{31}NO_3NaS$ $[M+Na]^+$ 556.1917, found 556.1928

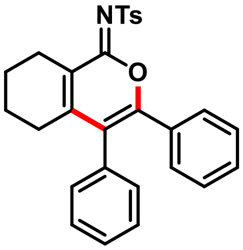

6p

(*Z*)-*N*-(3,4-diphenyl-5,6,7,8-tetrahydro-1*H*-isochromen-1-ylidene)-4-methyl benzenesulfonamide (6p)

The representative procedure A was followed using acrylic amide **5p** (83.8 mg, 0.3 mmol) and diphenylacetylene **2a** (35.6 mg, 0.2 mmol). Purification by column chromatography (*n*-hexane/EtOAc: 10/1) yielded **6p** (84.7 mg, 93%) as a yellow solid. **M**. **p**.: 215.8 – 217.3 ℃. **^{1}H NMR (400 MHz, CDCl$_3$)**: δ 7.88 (d, *J* = 8.0 Hz, 2 H); 7.38 – 7.29 (m, 5 H), 7.28 – 7.24 (m, 1 H); 7.23 – 7.17 (m, 2 H), 7.13 – 7.04 (m, 4 H), 2.64 – 2.56 (m, 2 H), 2.32 (s, 3H), 2.21 – 2.13, (m, 2H), 1.75 – 1.67 (m, 2H), 1.67 – 1.58 (m, 2H) **^{13}C NMR (100 MHz, CDCl$_3$)**: δ 159.6, 154.0, 152.6, 142.4, 139.4, 133.6, 131.1, 130.2, 129.6, 129.3, 129.0, 128.8, 128.3, 127.9, 127.0, 123.5, 121.7, 29.0, 24.2, 21.5, 21.4, 21.0. **IR** (neat): 1620, 1550, 1510, 1481, 1280, 1145, 1084, 868, 820, 688, 556 cm^{-1}. **HRMS** (ESI-TOF) m/z Calcd for $C_{28}H_{25}NO_3NaS$ [M+Na]$^+$ 478.1447, found 478.1454

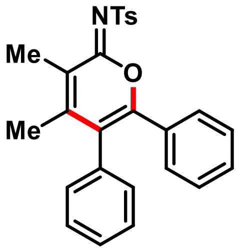

6q

(*Z*)-*N*-(3,4-dimethyl-5,6-diphenyl-2*H*-pyran-2-ylidene)-4-methylbenzenesulfonamide (6q)

The representative procedure A was followed using acrylic amide **5q** (76.0 mg, 0.3 mmol) and diphenylacetylene **2a** (35.6 mg, 0.2 mmol). Purification by column chromatography (*n*-hexane/EtOAc: 10/1) yielded **6q** (79.0 mg, 92%) as a yellow solid. **M. p.**: 176.3 – 181.9 ℃. **^{1}H NMR (400 MHz, CDCl$_3$)**: δ 7.87 (d, *J* = 8.2 Hz, 2 H), 7.35 (h, *J* = 2.4 Hz, 3 H), 7.32 – 7.24 (m, 3 H), 7.19 (dd, *J* = 8.2, 6.6 Hz, 2 H), 7.11 – 7.04 (m, 4 H), 2.30 (s, 3 H), 2.20 (s, 3 H), 1.95 (s, 3 H). **^{13}C NMR (100 MHz, CDCl$_3$)**: δ 160.1, 154.4, 151.7, 142.3, 139.3, 134.2, 131.0, 130.1, 129.6, 129.2, 128.9, 128.7, 128.2, 127.8, 126.8, 122.3, 122.2, 21.3, 18.4, 13.6. **IR** (neat): 2922, 1634, 1505, 1292, 1147, 1082, 829, 786, 698, 662, 554 cm^{-1}. **HRMS** (ESI-TOF) m/z Calcd for $C_{26}H_{23}NO_3NaS$ $[M+Na]^+$ 452.1291, found 452.1297

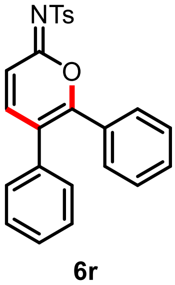

6r

5'-(4-Methoxyphenyl)-[1,1':2',1''-terphenyl]-4'(3'*H*)-one (6r)

The representative procedure A was followed using acrylic amide **5r** (67.6 mg, 0.3 mmol) and diphenylacetylene **2a** (35.6 mg, 0.2 mmol). Purification by column chromatography (*n*-hexane/EtOAc: 10/1) yielded **6r** (66.7 mg, 83%) as a yellow solid. **M. p.**: 90.2 – 163.7 ℃. **^{1}H NMR (400 MHz, $CDCl_3$)**: δ 7.89 (d, *J* = 8.0 Hz, 2 H), 7.48 (d, *J* = 8.0 Hz, 1 H), 7.43 (d, *J* = 8.0 Hz, 2 H), 7.39 – 7.32 (m, 4 H), 7.30 – 7.26 (m, 3 H), 7.19 – 7.14(m, 4 H), 2.35 (s, 3 H). **^{13}C NMR (100 MHz, $CDCl_3$)**: δ 157.9, 146.6, 142.9, 138.8, 135.1, 130.6, 130.5, 129.5, 129.1, 128.8, 128.4, 128.3, 126.9, 120.0, 21.3. **IR** (neat): 2918, 1637, 1560, 1519, 1479, 1443, 1287, 1144, 1082, 804, 668, 550 cm^{-1}. **HRMS** (ESI-TOF) m/z Calcd for $C_{24}H_{19}NO_3NaS$ $[M+Na]^+$ 424.0978, found 424.0967

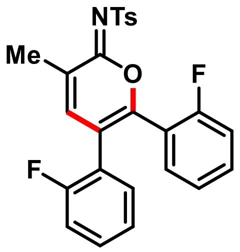

7a

(*Z*)-*N*-(5,6-bis(2-fluorophenyl)-3-methyl-2*H*-pyran-2-ylidene)-4-methylbenzenesulfonamide (7a)

The representative procedure A was followed using methacrylic amide **1a** (71.7 mg, 0.3 mmol) and 1,2-bis(2-fluorophenyl)ethyne **2b** (42.8 mg, 0.2 mmol). Purification by column chromatography (*n*-hexane/EtOAc: 10/1) yielded **7a**

(59.6 mg, 66%) as a white semisolid. **^{1}H NMR (400 MHz, $CDCl_3$)**: δ 7.81 (d, *J* = 8.4 Hz, 2 H), 7.65 (m, 1 H), 7.42 (m, 1 H), 7.32 – 7.24 (m, 3 H), 7.12 – 6.85 (m, 6 H), 2.34 (s, 3 H), 2.20 (s, 3 H). **^{13}C NMR (100 MHz, $CDCl_3$)**: δ 159.7, 159.6 (d, *J* = 247.0 Hz), 159.0 (d, *J* = 252.7 Hz), 151.5 (d, *J* = 2.4 Hz), 142.8, 142.2 (d, *J* = 2.2 Hz), 138.7, 132.6 (d, *J* = 8.4 Hz), 131.6 (d, *J* = 1.7 Hz), 130.6 (d, *J* = 8.3 Hz), 130.5 (d, *J* = 1.9 Hz), 128.9, 127.3, 126.8, 124.5 (d, *J* = 3.6 Hz), 124.3 (d, *J* = 3.7 Hz), 122.6 (dd, *J* = 14.6, 2.5 Hz), 119.1 (d, *J* = 13.5 Hz), 117.3 (d, *J* = 1.5 Hz), 116.1 (d, *J* = 7.6 Hz), 115.9 (d, *J* = 7.7 Hz), 21.4, 17.1. **^{19}F NMR (376 MHz, $CDCl_3$)** δ -110.27, -114.97. **IR** (neat): 1649, 1576, 1532, 1485, 1446, 1300, 1150, 1080, 881, 814, 763, 667, 606, 562 cm^{-1}. **HRMS** (ESI-TOF) m/z Calcd for $C_{25}H_{20}NO_3F_2S$ $[M+H]^+$ 452.1127, found 452.1115

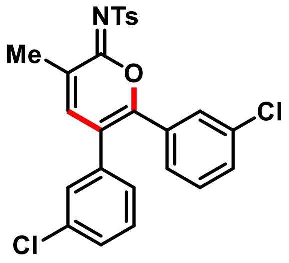

7b

(*Z*)-*N*-(5,6-bis(3-chlorophenyl)-3-methyl-2*H*-pyran-2-ylidene)-4-methylbenzenesulfonamide (7b)

The representative procedure A was followed using methacrylic amide **1a** (71.7 mg, 0.3 mmol) and 1,2-bis(3-chlorophenyl)ethyne **2c** (49.4 mg, 0.2 mmol). Purification by column chromatography (*n*-hexane/EtOAc: 10/1) yielded **7b** (89.5 mg, 93%) as a yellow semisolid. **^{1}H NMR (400 MHz, $CDCl_3$)**: δ 7.88 (d,

J = 8.4 Hz, 2 H), 7.38 – 7.37 (m, 1 H), 7.36 – 7.31 (m, 2 H), 7.31 – 7.27 (m, 1 H), 7.27 – 7.22 (m, 3 H), 7.22 – 7.16 (m, 3 H), 7.00 – 6.98 (m, 1 H), 2.35 (s, 3 H), 2.18 (s, 3 H). **^{13}C NMR (100 MHz, CDCl$_3$)**: δ 159.4, 154.1, 142.9, 142.4, 138.9, 136.8, 135.1, 134.4, 131.9, 130.5, 130.4, 129.7, 129.2, 129.1, 128.8, 128.7, 127.5, 127.2, 127.0, 126.9, 119.2, 21.5, 17.0. **IR** (neat): 1640, 1570, 1525, 1468, 1414, 1297, 1147, 1083, 889, 802, 666, 560 cm^{-1}. **HRMS** (ESI-TOF) m/z Calcd for $C_{25}H_{19}NO_3NaSCl_2$ [M+Na]$^+$ 506.0355, found 506.0352

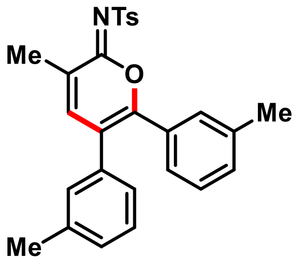

7c

(*Z*)-4-methyl-*N*-(3-methyl-5,6-di-m-tolyl-2*H*-pyran-2-ylidene)benzenesulfonamide (7c)

The representative procedure A was followed using methacrylic amide **1a** (71.7 mg, 0.3 mmol) and 1,2-di-*p*-tolylethyne **2d** (41.3 mg, 0.2 mmol). Purification by column chromatography (*n*-hexane/EtOAc: 10/1) yielded **7c** (78.2 mg, 88%) as a yellow solid. **M**. **p**.: 179.5 – 180.8 ℃ **^{1}H NMR (400 MHz, CDCl$_3$)**: δ 7.89 (d, J = 8.0 Hz, 2 H), 7.32 (s, 1 H), 7.29-7.27 (m, 1 H), 7.20-7.12 (m, 7 H), 6.98 (s, 1 H), 6.90 (d, J = 7.6 Hz, 1 H), 2.33 (s, 3 H), 2.30 (s, 3 H), 2.28 (s, 3 H), 2.18 (s, 3 H). **^{13}C NMR (100 MHz, CDCl$_3$)**: δ 160.2, 155.9, 143.8, 142.6, 139.2, 138.8, 137.8, 135.5, 130.9, 130.6, 129.9, 129.3, 128.9, 128.8, 128.0, 127.0, 126.6,

126.0, 125.5, 120.1, 21.4, 16.9. **IR** (neat): 1565, 1510, 1298, 1145, 1082, 904, 804, 660, 612, 557 cm^{-1}. **HRMS** (ESI-TOF) m/z Calcd for $C_{27}H_{25}NO_3NaS$ $[M+Na]^+$ 466.1447, found 466.1446

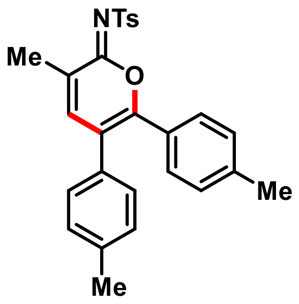

7d

(*Z*)-4-methyl-*N*-(3-methyl-5,6-di-p-tolyl-2*H*-pyran-2-ylidene)benzenesulfonamide (7d)

The representative procedure A was followed using methacrylic amide **1a** (71.7 mg, 0.3 mmol) and 1,2-di-*p*-tolylethyne **2e** (41.3 mg, 0.2 mmol). Purification by column chromatography (*n*-hexane/EtOAc: 10/1) yielded **7d** (66.7 mg, 75%) as a yellow semisolid. **^{1}H NMR (400 MHz, $CDCl_3$)**: δ 7.89 (d, *J* = 8.4 Hz, 2 H), 7.35 (d, *J* = 8.0 Hz, 2 H), 7.27 – 7.24 (m, 1 H), 7.16 – 7.06 (m, 6 H), 7.03 (d, *J* = 7.9 Hz, 2 H), 2.34 (s, 6 H), 2.33 (s, 3 H), 2.17 (s, 3 H). **^{13}C NMR (100 MHz, $CDCl_3$)**: δ 160.2, 155.9, 144.1, 142.6, 140.5, 139.2, 138.1, 132.7, 129.7, 129.27, 129.0, 128.9, 128.7, 127.9, 127.0, 125.2, 119.6, 21.4, 21.1, 16.9. **IR** (neat): 1638, 1568, 1527, 1495, 1302, 1152, 1084, 921, 816, 748, 665, 597, 554 cm^{-1}. **HRMS** (ESI-TOF) m/z Calcd for $C_{27}H_{25}NO_3NaS$ $[M+Na]^+$ 466.1447, found 466.1447

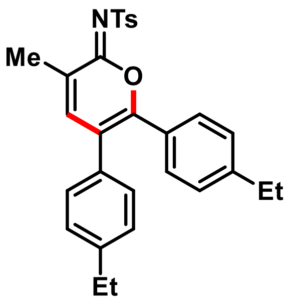

7e

(*Z*)-*N*-(5,6-bis(4-ethylphenyl)-3-methyl-2*H*-pyran-2-ylidene)-4-methylbenzenesulfonamide (7e)

The representative procedure A was followed using methacrylic amide **1a** (71.7 mg, 0.3 mmol) and 1,2-bis(4-ethylphenyl)ethyne **2f** (46.9 mg, 0.2 mmol). Purification by column chromatography (*n*-hexane/EtOAc: 10/1) yielded **7e** (81.1 mg, 86%) as a yellow solid. **M**. **p**.: 201.4 – 204.4 ℃. **^{1}H NMR (400 MHz, $CDCl_3$)**: δ 7.89 (d, *J* = 8.0 Hz, 2 H), 7.38 (d, *J* = 8.4 Hz, 2 H), 7.27 (d, *J* = 1.6 Hz, 1 H), 7.17 – 7.09 (m, 6 H), 7.09 – 7.04 (m, 2 H), 2.68 – 2.61 (m, 4 H), 2.33 (s, 3 H), 2.16 (s, 3 H), 1.25 – 1.21 (m, 6 H). **^{13}C NMR (100 MHz, $CDCl_3$)**: δ 160.2, 155.9, 146.7, 144.4, 144.1, 142.6, 139.2, 132.9, 129.4, 128.9, 128.8, 128.5, 128.1, 127.7, 127.0, 125.2, 119.7, 28.7, 28.5, 21.4, 16.9, 15.3, 15.1. **IR** (neat): 1639, 1569, 1525, 1497, 1298, 1141, 1079, 881, 837, 652, 599 cm^{-1}. **HRMS** (ESI-TOF) m/z Calcd for $C_{29}H_{29}NO_3NaS$ $[M+Na]^+$ 494.1760, found 494.1761

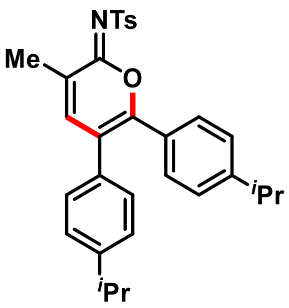

7f

(*Z*)-*N*-(5,6-bis(4-isopropylphenyl)-3-methyl-2*H*-pyran-2-ylidene)-4-methylbenzenesulfonamide (7f)

The representative procedure A was followed using methacrylic amide **1a** (71.7 mg, 0.3 mmol) and 1,2-bis(4-isopropylphenyl)ethyne **2g** (52.5 mg, 0.2 mmol). Purification by column chromatography (*n*-hexane/EtOAc: 10/1) yielded **7f** (82.9 mg, 83%) as a yellow oil. **^{1}H NMR (400 MHz, $CDCl_3$)**: δ 7.89 (d, *J* = 8.0 Hz, 2 H), 7.40 (d, *J* = 8.0 Hz, 2 H), 7.30 – 7.26 (m, 1 H), 7.18 (d, *J* = 8.0 Hz, 2 H), 7.15 – 7.13 (m, 3 H), 7.15 – 7.08 (m, 2 H), 7.07 (s, 1 H), 2.98 – 2.85 (m, 2H), 2.33 (s, 3H), 2.16 (s, 3H), 1.28 – 1.22 (m, 12H). **^{13}C NMR (100 MHz, $CDCl_3$)**: δ 160.3, 155.9, 151.3, 149.0, 144.2, 142.5, 139.3, 133.0, 129.3, 128.88, 128.8, 128.2, 127.1, 127.0, 126.3, 125.1, 119.6, 33.9, 33.7, 23.8, 23.7, 21.4, 16.9. **IR** (neat): 2956, 1638, 1569, 1523, 1497, 1299, 1140, 1076, 877, 841, 654, 594 cm^{-1}. **HRMS** (ESI-TOF) m/z Calcd for $C_{31}H_{33}NO_3NaS$ $[M+Na]^+$ 522.2073, found 522.2083

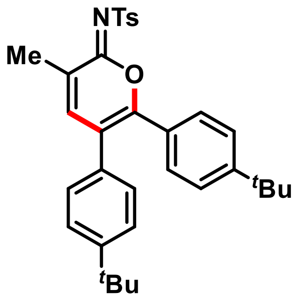

7g

(*Z*)-*N*-(5,6-bis(4-(tert-butyl)phenyl)-3-methyl-2*H*-pyran-2-ylidene)-4-methylbenzenesulfonamide (7g)

The representative procedure A was followed using methacrylic amide **1a** (71.7 mg, 0.3 mmol) and 1,2-bis(4-(tert-butyl)phenyl)ethyne **2h** (58.1 mg, 0.2 mmol). Purification by column chromatography (*n*-hexane/EtOAc: 10/1) yielded **7g** (96.3 mg, 91%) as a yellow solid. **M. p.**: 151.8 – 153.7 ℃. **^{1}H NMR (400 MHz, $CDCl_3$)**: δ 7.94 – 7.87 (m, 2 H), 7.46 – 7.40 (m, 2 H), 7.38 – 7.33 (m, 2 H), 7.32 – 7.28 (m, 2 H), 7.27 – 7.26 (m, 1 H), 7.14 – 7.10 (m, 4 H), 2.33 (s, 3 H), 2.16 (s, 3 H), 1.32 (s, 9 H), 1.31 (s, 9 H). **^{13}C NMR (100 MHz, $CDCl_3$)**: δ 160.3, 155.8, 153.6, 151.3, 144.3, 142.5, 139.3, 132.7, 129.0, 128.9, 128.5, 127.9, 127.0, 125.9, 125.2, 125.1, 119.6, 34.8, 34.6, 31.2, 31.0, 21.4, 16.9. **IR** (neat): 2958, 1493, 1296, 1145, 1082, 729, 663 cm^{-1}. **HRMS** (ESI-TOF) m/z Calcd for $C_{33}H_{37}NO_3NaS$ $[M+Na]^+$ 550.2386, found 550.2386

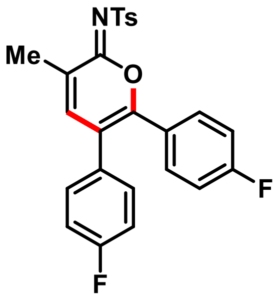

7h

(*Z*)-*N*-(5,6-bis(4-fluorophenyl)-3-methyl-2*H*-pyran-2-ylidene)-4-methylbenzenesulfonamide (7h)

The representative procedure A was followed using methacrylic amide **1a** (71.7 mg, 0.3 mmol) and 1,2-bis(4-fluorophenyl)ethyne **2i** (42.8 mg, 0.2 mmol). Purification by column chromatography (*n*-hexane/EtOAc: 10/1) yielded **7h** (78.9 mg, 87%) as a yellow solid. **M. p.**: 215.2 – 217.2 ℃. **^{1}H NMR (400 MHz, $CDCl_3$)**: 7.86 (d, *J* = 8.4 Hz, 2 H), 7.48 – 7.41 (m, 2 H), 7.24 (d, *J* = 1.2 Hz, 1 H), 7.18 – 7.09 (m, 4 H), 7.06 - 6.96 (m, 4 H), 2.34 (s, 3 H), 2.16 (s, 3 H). **^{13}C NMR (100 MHz, $CDCl_3$)**: δ 163.6 (d, *J* = 251.1 Hz), 162.6 (d, *J* = 247.8 Hz), 159.8, 154.6, 143.2, 142.8, 139.0, 131.5 (d, *J* = 8.7 Hz), 131.3 (d, *J* = 3.4 Hz), 130.7 (d, *J* = 8.2 Hz), 129.0, 126.9, 126.7 (d, *J* = 3.4 Hz), 126.0, 118.8, 116.3 (d, *J* = 21.7 Hz), 115.64 (d, *J* = 21.9 Hz), 21.4, 16.9. **^{19}F NMR (376 MHz, $CDCl_3$)** δ -108.44, -112.41. **IR** (neat): 1640, 1576, 1530, 1493, 1283, 1222, 1153, 1082, 881, 834, 674, 582, 533 cm^{-1}. **HRMS** (ESI-TOF) m/z Calcd for $C_{25}H_{19}NO_3F_2NaS$ $[M+Na]^+$ 474.0946, found 474.0957

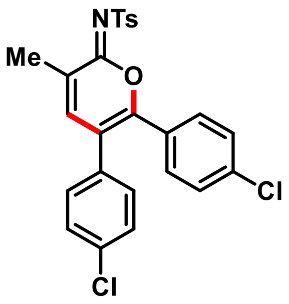

7i

(*Z*)-*N*-(5,6-bis(4-chlorophenyl)-3-methyl-2*H*-pyran-2-ylidene)-4-methylbenzenesulfonamide (7i)

The representative procedure A was followed using methacrylic amide **1a** (71.7 mg, 0.3 mmol) and 1,2-bis(4-chlorophenyl)ethyne **2j** (49.4 mg, 0.2 mmol). Purification by column chromatography (*n*-hexane/EtOAc: 10/1) yielded **7i** (67.3 mg, 70%) as a yellow solid. **M. p.**: 244.2 – 248.4 ℃. **^{1}H NMR (400 MHz, $CDCl_3$)**: δ 7.87 (d, *J* = 8.0 Hz, 2 H), 7.39 (d, *J* = 8.4 Hz, 2 H), 7.30 (dd, *J* = 16.4, 8.0 Hz, 4 H), 7.24 (s, 1 H), 7.18 (d, *J* = 8.0 Hz, 2 H), 7.10 (d, *J* = 8.0 Hz, 2 H), 2.36 (s, 3 H), 2.17 (s, 3 H). **^{13}C NMR (100 MHz, $CDCl_3$)**: δ 159.6, 154.3, 142.9, 142.8, 139.0, 136.5, 134.6, 133.6, 130.6, 130.2, 129.5, 129.0, 128.8, 128.7, 126.8, 126.4, 119.0, 21.4, 16.9. **IR** (neat): 1633, 1567, 1521, 1480, 1296, 1148, 1084, 871, 829, 670 cm^{-1}. **HRMS** (ESI-TOF) m/z Calcd for $C_{25}H_{19}NO_3NaSCl_2$ $[M+Na]^+$ 506.0355, found 506.0359

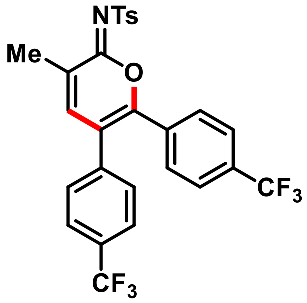

7j

(*Z*)-4-methyl-*N*-(3-methyl-5,6-bis(4-(trifluoromethyl)phenyl)-2*H*-pyran-2-ylidene)benzenesulfonamide (7j)

The representative procedure A was followed using methacrylic amide **1a** (71.7 mg, 0.3 mmol) and 1,2-bis(4-(trifluoromethyl)phenyl)ethyne **2k** (62.9 mg, 0.2 mmol). Purification by column chromatography (*n*-hexane/EtOAc: 10/1) yielded **7j** (104.3 mg, 95%) as a yellow solid. **M. p.**: 236.1 – 237.1 ℃. **^{1}H NMR (400 MHz, $CDCl_3$)**: δ 7.87 (d, *J* = 8.4 Hz, 2H), 7.63 (d, *J* = 8.0 Hz, 2H), 7.58 (s, 4 H), 7.32 (d, *J* = 8.0 Hz, 2 H), 7.27 (s, 1 H), 7.19 (d, *J* = 8.0 Hz, 2 H), 2.37 (s, 3 H), 2.19 (s, 3 H). **^{13}C NMR (100 MHz, $CDCl_3$)**: δ 159.2, 153.9, 143.1, 142.1, 139.0, 138.8, 133.6, 132.0 (q, *J* = 33.0 Hz), 130.8 (q, *J* = 32.7 Hz), 129.7, 129.4, 129.1, 127.5, 126.8, 126.3, 125.5, 123.6 (q, *J* = 271.0 Hz), 123.4 (q, *J* = 271.4Hz), 119.6, 21.4, 17.0. **^{19}F NMR (376 MHz, $CDCl_3$)** δ -62.79, -63.09. **IR** (neat): 1634, 1570, 1531, 1411, 1317, 1159, 1115, 1074, 861, 667 cm^{-1}. **HRMS** (ESI-TOF) m/z Calcd for $C_{27}H_{19}NO_3F_6NaS$ $[M+Na]^+$ 574.0882, found 574.0873

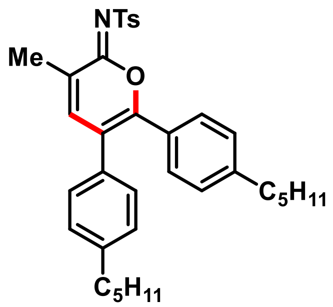

7k

(*Z*)-4-methyl-*N*-(3-methyl-5,6-bis(4-pentylphenyl)-2*H*-pyran-2-ylidene)benzenesulfonamide (7k)

The representative procedure A was followed using methacrylic amide **1a** (71.7 mg, 0.3 mmol) and 1,2-bis(4-pentylphenyl)ethyne **2l** (63.7 mg, 0.2 mmol). Purification by column chromatography (*n*-hexane/EtOAc: 10/1) yielded **7k** (101.0 mg, 91%) as a yellow oil. **^{1}H NMR (400 MHz, $CDCl_3$)**: δ 7.92 – 7.87 (m, 2 H), 7.39 – 7.33 (m, 2 H), 7.29 – 7.27 (m, 1 H), 7.15 – 7.09 (m, 5 H), 7.08 – 7.04 (m, 3 H), 2.62 – 2.57 (m, 4 H), 2.33 (s, 3 H), 2.17 (d, *J* = 1.2 Hz, 3 H), 1.65 – 1.57 (m, 4 H), 1.38 – 1.25 (m, 8 H), 0.89 (t, *J* = 7.2 Hz, 6 H). **^{13}C NMR (100 MHz, $CDCl_3$)**: δ 160.3, 156.0, 145.4, 144.1, 143.1, 142.5, 139.2, 132.9, 129.3, 129.0, 128.9, 128.7, 128.2, 128.1, 127.0, 125.1, 119.7, 35.7, 35.5, 31.4, 30.9, 30.6, 22.4, 22.4, 21.4, 16.9, 14.0, 14.0. **IR** (neat): 2923, 2857, 1638, 1571, 1528, 1497, 1300, 1150, 1084, 877, 812, 733, 670 cm^{-1}. **HRMS** (ESI-TOF) m/z Calcd for $C_{33}H_{41}NO_3NaS$ $[M+Na]^+$ 578.2699, found 578.2703

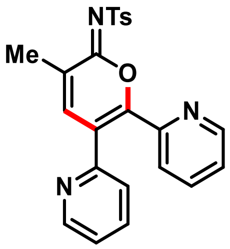

7l

(*Z*)-4-methyl-*N*-(3-methyl-5,6-di(pyridin-2-yl)-2*H*-pyran-2-ylidene)benzene sulfonamide (7l)

The representative procedure A was followed using methacrylic amide **1a** (71.7 mg, 0.3 mmol) and 1,2-di(pyridin-2-yl)ethyne **2m** (36.4 mg, 0.2 mmol). Purification by column chromatography (*n*-hexane/EtOAc: 3/1) yielded **7l** (42.3

mg, 51%) as a brown solid. **M. p.**: 193.6 – 199.3 ℃. **^{1}H NMR (400 MHz, CDCl$_3$)**: δ 8.71 (d, *J* = 4.8 Hz, 1 H), 8.45 – 8.32 (m, 1 H), 8.22 (d, *J* = 8.0 Hz, 1 H), 8.02 – 7.96 (m, 2 H), 7.93 (td, *J* = 8.0, 2.0 Hz, 1 H), 7.68 (d, *J* = 1.2 Hz, 1H), 7.61 (td, *J* = 7.6, 2.0 Hz, 1 H), 7.39 – 7.33 (m, 1H), 7.33 – 7.27 (m, 1 H), 7.25 (d, *J* = 8.0 Hz, 2 H), 7.13 (d, *J* = 8.0 Hz, 1 H), 2.42 (s, 3 H), 2.27 (d, *J* = 1.2 Hz, 3 H). **^{13}C NMR (100 MHz, CDCl$_3$)**: δ 159.3, 154.3, 153.9, 149.6, 149.3, 148.8, 142.8, 142.8, 139.0, 137.0, 135.6, 129.0, 127.1, 126.8, 124.8, 124.8, 124.3, 122.5, 120.9, 21.4, 17.0. **IR** (neat): 1644, 1567, 1529, 1465, 1424, 1286, 1146, 1082, 871, 790, 670, 589 cm^{-1}. **HRMS** (ESI-TOF) m/z Calcd for $C_{23}H_{19}N_3O_3NaS$ [M+Na]$^+$ 440.1039, found 440.1039

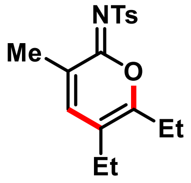

7m

(*Z*)-*N*-(5,6-diethyl-3-methyl-2*H*-pyran-2-ylidene)-4-methylbenzenesulfonamide (7m)

The representative procedure A was followed using methacrylic amide **1a** (71.7 mg, 0.3 mmol) and hex-3-yne **2n** (16.5 mg, 0.2 mmol). Purification by column chromatography (*n*-hexane/EtOAc: 10/1) yielded **7m** (57.8 mg, 91%) as a white solid. **M. p.**: 75.7 – 78.5 ℃. **^{1}H NMR (400 MHz, CDCl$_3$)**: δ 7.90 (d, *J* = 8.0 Hz, 2 H), 7.25 (d, *J* = 8.0 Hz, 2 H), 7.02 (d, *J* = 1.6 Hz, 1 H), 2.48 (q, *J* = 7.6 Hz, 2 H), 2.38 (s, 3 H), 2.27 (q, *J* = 7.6 Hz, 2 H), 2.05 (s, 3 H), 1.15 (t, *J* = 7.6 Hz, 3 H), 1.05 (t, *J* = 7.6 Hz, 3 H). **^{13}C NMR (100 MHz, CDCl$_3$)**: δ 161.0, 160.3,

142.8, 142.5, 139.6, 129.0, 127.0, 125.0, 118.9, 23.3, 22.5, 21.4, 16.9, 14.5, 12.4. **IR** (neat): 1510, 1383, 1297, 1145, 1079, 894, 811, 685, 651, 594, 546 cm^{-1}. **HRMS** (ESI-TOF) m/z Calcd for $C_{17}H_{21}NO_3NaS$ $[M+Na]^+$ 342.1134, found 342.1131

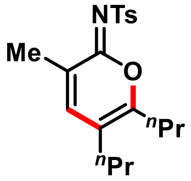

7n

(*Z*)-4-methyl-*N*-(3-methyl-5,6-dipropyl-2*H*-pyran-2-ylidene)benzenesulfonamide (7n)

The representative procedure A was followed using methacrylic amide **1a** (71.7 mg, 0.3 mmol) and oct-4-yne **2o** (22.0 mg, 0.2 mmol). Purification by column chromatography (*n*-hexane/EtOAc: 10/1) yielded **7n** (65.7 mg, 95%) as a white solid. **M**. **p**.: 75.9 – 82.4 ℃. **^{1}H NMR (400 MHz, $CDCl_3$)**: δ 7.92 (d, *J* = 8.4 Hz, 2 H), 7.27 (d, *J* = 8.4 Hz, 2 H), 7.03 (d, *J* = 1.6 Hz, 1 H), 2.45-2.41 (m, 5 H), 2.28 – 2.20 (m, 2 H), 2.08 (s, 3 H), 1.59 (h, *J* = 7.6 Hz, 2 H), 1.48 (h, *J* = 7.6 Hz, 2 H), 0.93 (m, 6 H). **^{13}C NMR (100 MHz, $CDCl_3$)**: δ 161.0, 159.5, 143.0, 142.4, 139.7, 129.0, 127.0, 124.8, 117.9, 31.8, 31.1, 23.1, 21.4, 21.2, 16.8, 13.7, 13.5. **IR** (neat): 1653, 1522, 1295, 1149, 1079, 818, 672, 602, 557 cm^{-1}. **HRMS** (ESI-TOF) m/z Calcd for $C_{19}H_{25}NO_3NaS$ $[M+Na]^+$ 370.1447, found 370.1452

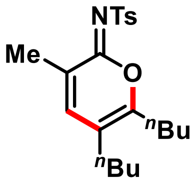

7o

(*Z*)-*N*-(5,6-dibutyl-3-methyl-2*H*-pyran-2-ylidene)-4-methylbenzenesulfonamide (7o)

The representative procedure A was followed using methacrylic amide **1a** (71.7 mg, 0.3 mmol) and dec-5-yne **2p** (27.6 mg, 0.2 mmol). Purification by column chromatography (*n*-hexane/EtOAc: 10/1) yielded **7o** (70.2 mg, 94%) as a white solid. **M. p.**: 80.7 – 82.5 ℃. **^{1}H NMR (400 MHz, $CDCl_3$)**: δ 7.92 (d, *J* = 8.0 Hz, 2 H), 7.27 (d, *J* = 8.0 Hz, 2 H), 7.04 (d, *J* = 1.6 Hz, 1 H), 2.45 (t, *J* = 8.0 Hz, 2 H), 2.40 (s, 3 H), 2.25 (t, *J* = 8.0 Hz, 2 H), 2.08 (s, 3 H), 1.55 – 1.47 (m, 2 H), 1.45 – 1.38 (m, 2 H), 1.37 - 1.26 (m, 4 H), 0.95-0.89 (m, 6 H). **^{13}C NMR (100 MHz, $CDCl_3$)**: δ 161.0, 159.6, 143.1, 142.4, 139.7, 129.0, 127.0, 124.7, 118.0, 32.0, 29.8, 29.6, 28.8, 22.3, 22.1, 21.4, 16.8, 13.7, 13.6. **IR** (neat): 2922, 2864, 1645, 1514, 1299, 1153, 1083, 816, 709, 599, 550 cm^{-1}. **HRMS** (ESI-TOF) m/z Calcd for $C_{21}H_{29}NO_3NaS$ [M+Na]$^+$ 398.1760, found 398.1769

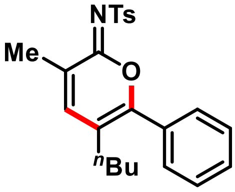

7p

(*Z*)-*N*-(5-butyl-3-methyl-6-phenyl-2*H*-pyran-2-ylidene)-4-methylbenzenesulfonamide (7p)

The representative procedure A was followed using methacrylic amide **1a** (71.7 mg, 0.3 mmol) and hex-1-yn-1-ylbenzene **2q** (31.7 mg, 0.2 mmol). Purification by column chromatography (*n*-hexane/EtOAc: 10/1) yielded **7p** (56.4 mg, 70%) as a yellow solid. **M. p.**: 119.9 – 122.5 ℃. **^{1}H NMR (400 MHz, $CDCl_3$)**: δ 7.77 (d, *J* = 8.4 Hz, 2 H), 7.56 – 7.44 (m, 5 H), 7.18 (d, *J* = 1.2 Hz, 1 H), 7.04 (d, *J* = 8.0 Hz, 2 H), 2.45 – 2.38 (m, 2 H), 2.30 (s, 3 H), 2.15 (s, 3 H), 1.54 – 1.42 (m, 2 H), 1.34 -1.22 (m, 2 H), 0.85 (t, *J* = 7.2 Hz, 3 H). **^{13}C NMR (100 MHz, $CDCl_3$)**: δ 160.3, 155.7, 143.0, 142.5, 139.0, 130.9, 130.1, 129.0, 128.8, 128.5, 127.2, 126.2, 119.0, 32.0, 29.1, 22.2, 21.4, 17.0, 13.6. **IR** (neat): 1641, 1576, 1524, 1279, 1147, 1077, 920, 785, 666, 586, 558 cm^{-1}. **HRMS** (ESI-TOF) m/z Calcd for $C_{23}H_{25}NO_3NaS$ $[M+Na]^+$ 418.1447, found 418.1448

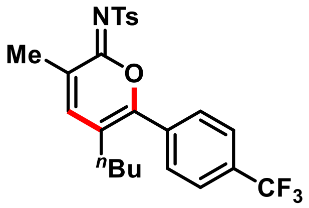

7q

(*Z*)-*N*-(5-butyl-3-methyl-6-(4-(trifluoromethyl)phenyl)-2*H*-pyran-2-ylidene)-4-methylbenzenesulfonamide (7q)

The representative procedure A was followed using methacrylic amide **1a** (71.7 mg, 0.3 mmol) and 1-(hex-1-yn-1-yl)-4-(trifluoromethyl)benzene **2r** (45.3 mg, 0.2 mmol). Purification by column chromatography (*n*-hexane/EtOAc: 10/1) yielded **7q** (78.7 mg, 85%) as a white solid. **M. p.**: 147.7 – 148.2 ℃. **^{1}H NMR (400 MHz, $CDCl_3$)**: δ 7.80 – 7.67 (m, 6 H), 7.19 (d, *J* = 1.6 Hz, 1 H), 7.09 (d, *J*

= 8.0 Hz, 2 H), 2.46 – 2.39 (m, 2 H), 2.33 (s, 3 H), 2.15 (s, 3 H), 1.55 – 1.46 (m, 2 H), 1.36 - 1.25 (m, 2 H), 0.87 (t, *J* = 7.2 Hz, 3 H) **^{13}C NMR (100 MHz, $CDCl_3$)**: δ 159.9, 153.5, 142.7, 142.5, 139.1, 134.4, 134.4, 131.8 (q, *J* = 32.7 Hz), 129.3, 128.9, 127.3, 127.0, 125.5 (q, *J* = 3.7 Hz), 125.0 (q, *J* = 270.9 Hz), 119.8, 31.9, 29.1, 22.2, 21.4, 17.0, 13.6. **^{19}F NMR (376 MHz, $CDCl_3$)** δ -62.92. **IR** (neat): 1536, 1315, 1293, 1159, 1122, 1074, 911, 855, 803, 666, 593 cm^{-1}. **HRMS** (ESI-TOF) m/z Calcd for $C_{24}H_{24}NO_3F_3NaS$ $[M+Na]^+$ 486.1321, found 486.1328

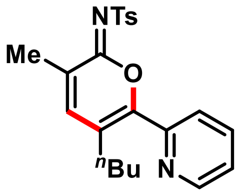

7r

(*Z*)-*N*-(5-butyl-3-methyl-6-(pyridin-2-yl)-2*H*-pyran-2-ylidene)-4-methylben zenesulfonamide (7r)

The representative procedure A was followed using methacrylic amide **1a** (71.7 mg, 0.3 mmol) and hex-1-yn-1-ylbenzene **2s** (31.9 mg, 0.2 mmol). Purification by column chromatography (*n*-hexane/EtOAc: 4/1) yielded **7r** (52.0 mg, 65%) as a yellow solid. **M**. **p**.: 122.3 – 127.6 ℃. **^{1}H NMR (400 MHz, $CDCl_3$)**: δ 8.68 (d, *J* = 4.3 Hz, 1 H), 8.37 (d, *J* = 8.0 Hz, 1 H), 7.96 – 7.92 (m, 3 H), 7.40 – 7.32 (m, 1 H), 7.28 – 7.21 (m, 3 H), 3.05 – 2.98 (m, 2 H), 2.41 (s, 3 H), 2.20 (s, 3 H), 1.64 – 1.53(m, 2 H), 1.45 – 1.34 (m, 2 H), 0.95 (t, *J* = 7.2 Hz, 3 H) **^{13}C NMR (100 MHz, $CDCl_3$)**: δ 159.8, 152.3, 150.7, 148.7, 144.9, 142.6, 139.4, 137.1, 129.0, 127.2, 126.6, 123.8, 123.8, 122.3, 31.9, 29.5, 22.3, 21.4, 16.9, 13.7. **IR**

(neat): 1564, 1514, 1437, 1294, 1142, 1081, 916, 808, 657, 602 cm^{-1}. **HRMS** (ESI-TOF) m/z Calcd for $C_{22}H_{24}N_2O_3NaS$ $[M+Na]^+$ 419.1400, found 419.1397

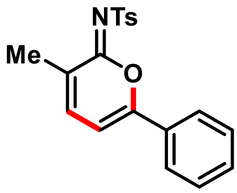

7s

(*Z*)-4-methyl-*N*-(3-methyl-6-phenyl-2*H*-pyran-2-ylidene)benzenesulfonamide (7s)

The representative procedure A was followed using methacrylic amide **1a** (35.9 mg, 0.15 mmol) and ethynylbenzene **2t** (30.6 mg, 0.30 mmol). Purification by column chromatography (*n*-hexane/EtOAc: 10/1) yielded **7s** (41.2 mg, 81%) as a white solid. **M. p.**: 144.8 – 187.0 ℃. **^{1}H NMR (400 MHz, $CDCl_3$)**: δ 8.00 – 7.89 (m, 4 H), 7.54 – 7.43 (m, 3 H), 7.27 – 7.21 (m, 3 H), 6.74 (d, *J* = 7.2 Hz, 1 H), 2.38 (s, 3 H), 2.11 (s, 3 H). **^{13}C NMR (100 MHz, $CDCl_3$)**: δ 160.5, 158.8, 142.7, 139.7, 139.7, 131.0, 130.4, 129.2, 129.1, 126.7, 125.9, 125.7, 103.2, 21.5, 17.2. **IR** (neat): 1635, 1567, 1504, 1290, 1145, 1087, 829, 764, 694, 658, 561 cm^{-1}. **HRMS** (ESI-TOF) m/z Calcd for $C_{19}H_{17}NO_3NaS$ $[M+Na]^+$ 362.0821, found 362.0825

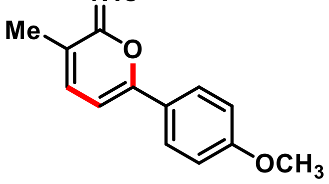

7t

(*Z*)-*N*-(6-(4-methoxyphenyl)-3-methyl-2*H*-pyran-2-ylidene)-4-methylbenzen

esulfonamide (7t)

The representative procedure A was followed using methacrylic amide **1a** (35.9 mg, 0.15 mmol) and 1-ethynyl-4-methoxybenzene **2u** (39.7 mg, 0.30 mmol). Purification by column chromatography (*n*-hexane/EtOAc: 10/1) yielded **7t** (22.2 mg, 40%) as a yellow semisolid. **^{1}H NMR (400 MHz, $CDCl_3$)**: δ 8.02 – 7.92 (m, 2 H), 7.92 – 7.83 (m, 2 H), 7.30 – 7.17 (m, 3 H), 7.03 – 6.93 (m, 2 H), 6.63 (d, J = 7.2 Hz, 1 H), 3.86 (s, 3 H), 2.38 (s, 3 H), 2.09 (d, J = 1.2 Hz, 3 H). **^{13}C NMR (100 MHz, $CDCl_3$)**: δ 161.9, 160.6, 159.0, 142.6, 140.3, 139.8, 129.1, 127.5, 126.6, 124.3, 122.9, 114.5, 101.9, 55.4, 21.5, 17.0. **IR** (neat): 1638, 1498, 1285, 1253, 1178, 1083, 1021, 959, 832, 667, 572, 517 cm^{-1}. **HRMS** (ESI-TOF) m/z Calcd for $C_{20}H_{19}NO_4NaS$ $[M+Na]^+$ 392.0927, found 392.0928

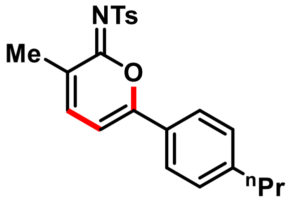

7u

(*Z*)-4-methyl-*N*-(3-methyl-6-(4-propylphenyl)-2*H*-pyran-2-ylidene)benzenesulfonamide (7u)

The representative procedure A was followed using methacrylic amide **1a** (35.9 mg, 0.15 mmol) and 1-ethynyl-4-propylbenzene **2v** (43.3 mg, 0.30 mmol). Purification by column chromatography (*n*-hexane/EtOAc: 10/1) yielded **7u** (35.5 mg, 62%) as a yellow solid. **M. p.**: 99.9 – 101.8 ℃. **^{1}H NMR (400 MHz, $CDCl_3$)**: δ 7.95 (d, J = 7.9 Hz, 2 H), 7.82 (d, J = 8.0 Hz, 2 H), 7.25 (m, 5 H),

6.70 (d, *J* = 7.2 Hz, 1 H), 2.63 (t, *J* = 7.6 Hz, 2 H), 2.37 (s, 3H), 2.08 (s, 3 H), 1.65 (m, 2 H), 0.95 (t, *J* = 7.2 Hz, 3 H). **^{13}C NMR (100 MHz, $CDCl_3$)**: δ 160.6, 159.1, 146.2, 142.6, 140.1, 139.7, 129.2, 129.1, 127.8, 126.6, 125.6, 125.1, 102.8, 37.8, 24.2, 21.4, 17.1, 13.7. **IR** (neat): 1636, 1592, 1491, 1295, 1142, 1084, 827, 744, 666, 562 cm^{-1}. **HRMS** (ESI-TOF) m/z Calcd for $C_{22}H_{23}NO_3NaS$ $[M+Na]^+$ 404.1291, found 404.1289

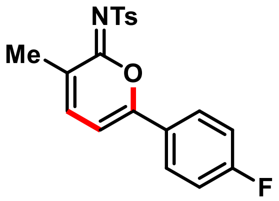

7v

(*Z*)-*N*-(6-(4-fluorophenyl)-3-methyl-2*H*-pyran-2-ylidene)-4-methylbenzenes ulfonamide (7v)

The representative procedure A was followed using methacrylic amide **1a** (35.9 mg, 0.15 mmol) and 1-ethynyl-4-fluorobenzene **2w** (36.0 mg, 0.30 mmol). Purification by column chromatography (*n*-hexane/EtOAc: 10/1) yielded **7v** (46.1 mg, 86%) as a yellow solid. **M. p.**: 185.1 – 188.7 ℃. **^{1}H NMR (400 MHz, $CDCl_3$)**: δ 7.92 (m, 4 H), 7.24 (m, 3 H), 7.15 (t, *J* = 8.4 Hz, 2 H), 6.68 (d, *J* = 7.2 Hz, 1 H), 2.38 (s, 3 H), 2.08 (s, 3 H). **^{13}C NMR (100 MHz, $CDCl_3$)**: δ 164.3 (d, *J* = 254.0. Hz), 160.3, 157.7, 142.8, 139.8, 139.5, 129.2, 127.8 (d, *J* = 8.0 Hz), 126.6, 125.6, 116.3 (d, *J* = 8.0 Hz), 102.9, 21.4, 17.1. **^{19}F NMR (376 MHz, $CDCl_3$)** δ -108.12. **IR** (neat): 1639, 1589, 1492, 1284, 1230, 1141, 1079, 813, 738, 660, 592, 559 cm^{-1}. **HRMS** (ESI-TOF) m/z Calcd for $C_{19}H_{16}NO_3FNaS$ $[M+Na]^+$ 380.0727, found 380.0723

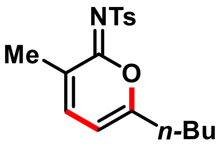

7w

(*Z*)-*N*-(6-butyl-3-methyl-2*H*-pyran-2-ylidene)-4-methylbenzenesulfonamide (7w)

The representative procedure A was followed using methacrylic amide **1a** (35.9 mg, 0.15 mmol) and hex-1-yne **2x** (24.7 mg, 0.30 mmol). Purification by column chromatography (*n*-hexane/EtOAc: 10/1) yielded **7w** (31.9 mg, 67%) as a white solid. **M. p.**: 110.3 – 117.2 ℃. **^{1}H NMR (400 MHz, $CDCl_3$)**: δ 7.90 (d, *J* = 8.0 Hz, 2 H), 7.30 – 7.23 (m, 3 H), 7.08 (dd, *J* = 6.8, 1.2 Hz, 1 H), 6.06 (d, *J* = 6.8 Hz, 1 H), 2.44 (t, *J* = 7.7 Hz, 2 H), 2.39 (s, 3 H), 2.06 (s, 3 H), 1.59 – 1.48 (m, 2 H), 1.37 – 1.28 (m, 2 H), 0.91 (t, *J* = 7.2 Hz, 3 H). **^{13}C NMR (100 MHz, $CDCl_3$)**: δ 163.8, 161.3, 142.6, 139.6, 139.5, 129.0, 127.1, 124.8, 105.1, 32.5, 28.9, 22.0, 21.5, 17.0, 13.6. **IR** (neat): 2921, 1652, 1519, 1295, 1144, 1083, 853, 701, 560 cm^{-1}. **HRMS** (ESI-TOF) m/z Calcd for $C_{17}H_{21}NO_3NaS$ $[M+Na]^+$ 342.1134, found 3342.1138

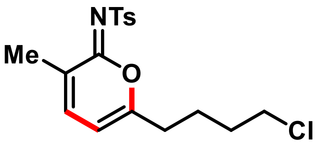

7x

(*Z*)-*N*-(6-(4-chlorobutyl)-3-methyl-2*H*-pyran-2-ylidene)-4-methylbenzenesul fonamide (7x)

The representative procedure A was followed using methacrylic amide **1a** (35.9 mg, 0.15 mmol) and 6-chlorohex-1-yne **2y** (35.0 mg, 0.30 mmol). Purification by column chromatography (*n*-hexane/EtOAc: 10/1) yielded **7x** (42.0 mg, 79%) as a brown semisolid. **^{1}H NMR (400 MHz, CDCl$_3$)**: δ 7.83 (d, *J* = 8.4 Hz, 2 H), 7.21 (d, *J* = 8.0 Hz, 2 H), 7.03 (dd, *J* = 6.8, 1.2 Hz, 1 H), 6.03 (d, *J* = 7.2 Hz, 1 H), 3.52 – 3.43 (m, 2 H), 2.49 – 2.41 (m, 2 H), 2.33 (s, 3 H), 1.99 (s, 3 H), 1.82 – 1.66 (m, 4 H). **^{13}C NMR (100 MHz, CDCl$_3$)**: δ 162.6, 161.1, 142.7, 139.5, 139.4, 129.1, 127.1, 127.0, 125.2, 105.4, 44.3, 32.0, 31.5, 24.1, 21.5, 17.0. **IR** (neat): 1652, 1527, 1297, 1147, 1081, 851, 827, 827, 680, 585 cm^{-1}. **HRMS** (ESI-TOF) m/z Calcd for $C_{20}H_{20}NO_3NaSCl$ [M+Na]$^+$ 376.0745, found 376.0741

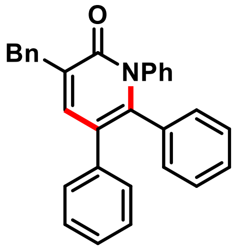

9a

3-Benzyl-1,5,6-triphenylpyridin-2(1*H*)-one (9a)

The representative procedure A was followed using phenylacrylic amide **8a** (71.2 mg, 0.3 mmol) and 1,2-diphenylethyne **2a** (35.7 mg, 0.2 mmol). Purification by column chromatography (*n*-hexane/EtOAc: 10/1) yielded **9a** (60.9 mg, 74%) as a brown solid. **M. p.**: 168.1 – 206.0 ℃. **^{1}H NMR (400 MHz, CDCl$_3$)**: δ 7.45 – 7.40 (m, 2 H), 7.38 – 7.30 (m, 3 H), 7.28 – 7.18 (m, 3 H), 7.18 – 7.03 (m, 6 H), 6.99 – 6.92 (m, 5 H), 6.89 – 6.82 (m, 2 H), 4.01 (s, 2 H). **^{13}C**

NMR (100 MHz, CDCl$_3$): δ 162.5, 144.1, 139.7, 139.4, 139.3, 138.5, 134.1, 132.4, 130.9, 129.6, 129.5, 129.1, 128.5, 128.5, 127.8, 127.6, 127.6, 127.4, 126.3, 126.2, 119.8, 36.8. **IR** (neat): 1649, 1604, 1537, 1486, 1446, 1129, 1070, 763, 695 cm^{-1}. **HRMS** (ESI-TOF) m/z Calcd for $C_{30}H_{24}NO$ $[M+H]^+$414.1852, found 414.1859

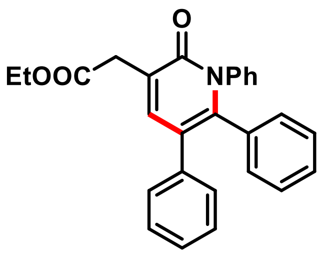

9b

Ethyl 3-(phenylcarbamoyl)but-3-enoate (9b)

The representative procedure A was followed using phenylacrylic amide **8b** (70.0 mg, 0.3 mmol) and 1,2-diphenylethyne **2a** (35.7 mg, 0.2 mmol). Purification by column chromatography (*n*-hexane/EtOAc: 10/1) yielded **9b** (56.5 mg, 69%) as a yellow solid. **M. p.**: 154.2 – 158.0 ℃. **^{1}H NMR (400 MHz, CDCl$_3$)**: δ 7.58 (s, 1 H), 7.24 – 7.17 (m, 2 H), 7.16 – 7.08 (m, 4 H), 7.07 – 6.99 (m, 4 H), 6.99 – 6.92 (m, 3 H), 6.89 – 6.84 (m, 2 H), 4.21 (q, *J* = 7.2 Hz, 2 H), 3.67 (s, 2 H), 1.28 (t, *J* = 7.2 Hz, 3 H). **^{13}C NMR (100 MHz, CDCl$_3$)**: δ 171.2, 162.2, 145.2, 141.6, 139.0, 138.3, 133.9, 130.9, 129.6, 129.1, 128.5, 127.9, 127.7, 127.7, 127.4, 126.4, 125.5, 119.8, 60.8, 36.1, 14.1. **IR** (neat): 1722, 1643, 1600, 1538, 1485, 1209, 1156, 1018, 747, 693 cm^{-1}. **HRMS** (ESI-TOF) m/z Calcd for $C_{27}H_{24}NO_3$ $[M+H]^+$ 410.1751, found 410.1744

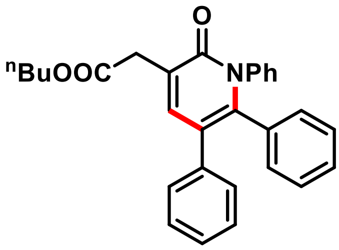

9c

Butyl 2-(2-oxo-1,5,6-triphenyl-1,2-dihydropyridin-3-yl)acetate (9c)

The representative procedure A was followed using phenylacrylic amide **8c** (78.4 mg, 0.3 mmol) and 1,2-diphenylethyne **2a** (35.7 mg, 0.2 mmol). Purification by column chromatography (*n*-hexane/EtOAc: 10/1) yielded **9c** (45.0 mg, 51%) as a yellow solid. **M. p.**: 101.7 – 117.9 ℃. **^{1}H NMR (400 MHz, $CDCl_3$)**: δ 7.57 (s, 1 H), 7.23 – 7.17 (m, 2 H), 7.16 – 7.08 (m, 4 H), 7.07 – 7.00 (m, 4 H), 6.99 – 6.92 (m, 3 H), 6.89 – 6.84(m, 2 H), 4.16 (t, *J* = 6.8 Hz, 2 H), 3.67 (s, 2 H), 1.70 – 1.58 (m, 2 H), 1.47 – 1.33 (m, 2 H), 0.93 (t, *J* = 7.2 Hz, 3 H). **^{13}C NMR (100 MHz, $CDCl_3$)**: δ 171.2, 162.2, 145.2, 141.6, 139.0, 138.3, 134.0, 130.9, 129.6, 129.1, 128.5, 127.9, 127.7, 127.7, 127.4, 126.4, 125.6, 119.8, 64.8, 36.1, 30.6, 19.1, 13.7. **IR** (neat): 1737, 1649, 1602, 1488, 1164, 760, 696 cm^{-1}. **HRMS** (ESI-TOF) m/z Calcd for $C_{29}H_{28}NO_3$ $[M+H]^+$ 438.2064, found 438.2069

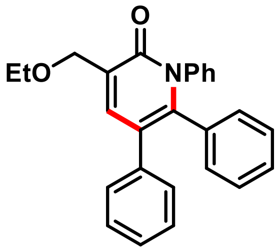

9d

3-(Ethoxymethyl)-1,5,6-triphenylpyridin-2(1*H*)-one (9d)

The representative procedure A was followed using phenylacrylic amide **8d** (61.6 mg, 0.3 mmol) and 1,2-diphenylethyne **2a** (35.7 mg, 0.2 mmol). Purification by column chromatography (*n*-hexane/EtOAc: 10/1) yielded **9d** (49.9 mg, 65%) as a yellow solid. **M. p.**: 144.1 – 149.4 ℃. **^{1}H NMR (400 MHz, $CDCl_3$)**: δ 7.72 (s, 1 H), 7.25 – 7.18 (m, 2 H), 7.17 – 7.09 (m, 4 H), 7.07 – 7.01 (m, 4 H), 7.00 – 6.92 (m, 3 H), 6.89 – 6.82 (m, 2 H), 4.60 – 4.56 (m, 2 H), 3.71 (q, *J* = 7.2 Hz, 2 H), 1.29 (t, *J* = 7.2 Hz, 3 H). **^{13}C NMR (100 MHz, $CDCl_3$)**: δ 161.4, 144.7, 139.0, 138.7, 138.6, 134.1, 130.9, 129.7, 129.3, 129.2, 128.5, 127.9, 127.7, 127.4, 126.4, 120.0, 67.6, 66.7, 15.2. **IR** (neat): 1737, 1649, 1602, 1488, 1164, 760, 696 cm^{-1}. **HRMS** (ESI-TOF) m/z Calcd for $C_{26}H_{23}NO_2Na$ $[M+Na]^+$404.1621, found 404.1613

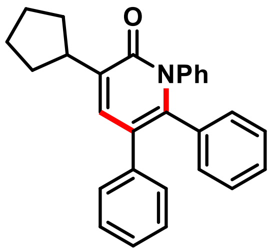

9e

3-Cyclopentyl-1,5,6-triphenylpyridin-2(1*H*)-one (9e)

The representative procedure A was followed using phenylacrylic amide **8e** (64.6 mg, 0.3 mmol) and 1,2-diphenylethyne **2a** (35.7 mg, 0.2 mmol). Purification by column chromatography (*n*-hexane/EtOAc: 10/1) yielded **9e** (36.5 mg, 47%) as a yellow solid. **M. p.**: 171.5 – 173.0 ℃. **^{1}H NMR (400 MHz, $CDCl_3$)**: δ 7.45 (s, 1 H), 7.24 – 7.18 (m, 2 H), 7.17 – 7.10 (m, 4 H), 7.09 – 7.00 (m, 4 H), 6.99 – 6.92 (m, 3 H), 6.90 – 6.84 (m, 2 H), 3.39 – 3.27 (m, 1 H), 2.16 – 2.07 (m, 2 H), 1.86 – 1.75 (m, 2 H), 1.75 – 1.62 (m, 4 H). **^{13}C NMR (100 MHz, $CDCl_3$)**: δ 162.6, 143.4, 139.5, 138.9, 137.0, 136.5, 134.3, 131.0, 129.6, 129.3, 128.5, 127.9, 127.5, 127.5, 127.4, 126.3, 119.6, 40.6, 32.0, 25.4. **IR** (neat): 1647, 1602, 1537, 1485, 1444, 760, 696 cm^{-1}. **HRMS** (ESI-TOF) m/z Calcd for $C_{28}H_{26}NO$ $[M+H]^+$ 392.2009, found 392.1999

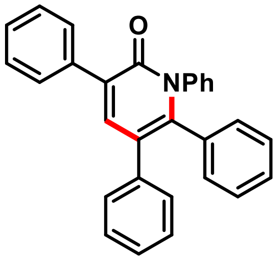

9f

1,3,5,6-Tetraphenylpyridin-2(1*H*)-one (9f)

The representative procedure A was followed using phenylacrylic amide **8f** (67.0 mg, 0.3 mmol) and 1,2-diphenylethyne **2a** (35.7 mg, 0.2 mmol). Purification by column chromatography (*n*-hexane/EtOAc: 10/1) yielded **9f** (60.9 mg, 76%) as a white solid. **M. p.**: 214.2 – 231.2 ℃. **^{1}H NMR (400 MHz, $CDCl_3$)**: δ 7.87 – 7.82 (m, 2 H), 7.78 (s, 1 H), 7.42 – 7.36 (m, 2 H), 7.35 – 7.28

(m, 1 H), 7.24 – 7.19 (m, 2 H), 7.17 – 7.08 (m, 6 H), 7.08 – 7.03 (m, 2 H), 7.00 – 6.94 (m, 3 H), 6.93 – 6.88 (m, 2 H). **^{13}C NMR (100 MHz, $CDCl_3$)**: δ 161.6, 145.6, 140.6, 139.4, 138.4, 136.4, 134.0, 130.8, 130.5, 129.6, 129.2, 128.7, 128.5, 128.0, 128.0, 127.8, 127.7, 127.5, 126.5, 120.3. **IR** (neat): 1650, 1590, 1527, 1484, 1441, 747, 695, 592, 535 cm^{-1}. **HRMS** (ESI-TOF) m/z Calcd for $C_{29}H_{22}NO$ $[M+H]^+$ 400.1696, found 400.1704

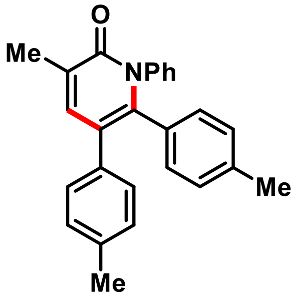

9g

3-Methyl-1-phenyl-5,6-di-p-tolylpyridin-2(1*H*)-one (9g)

The representative procedure A was followed using phenylacrylic amide **1b** (48.4 mg, 0.3 mmol) and 1,2-di-*p*-tolylethyne **2e** (41.3 mg, 0.2 mmol). Purification by column chromatography (*n*-hexane/EtOAc: 10/1) yielded **9g** (45.6 mg, 62%) as a yellow solid. **^{1}H NMR (400 MHz, $CDCl_3$)**: δ 7.44 (s, 1 H), 7.25 – 7.18 (m, 2 H), 7.18 – 7.11 (m, 1 H), 7.06 – 7.00 (m, 2 H), 6.94 (d, *J* = 8.0 Hz, 2 H), 6.89 (d, *J* = 8.0 Hz, 2 H), 6.79 – 6.69 (m, 4 H), 2.26 (s, 3H), 2.25 (s, 3 H), 2.13 (s, 3 H). **^{13}C NMR (100 MHz, $CDCl_3$)**: δ 163.1, 143.7, 140.3, 139.5, 137.2, 135.8, 131.3, 130.8, 129.4, 129.1, 128.7, 128.6, 128.5, 128.1, 127.5, 119.7, 21.1, 21.0, 17.2. The spectral data are in accordance with those reported in the literature.[17]

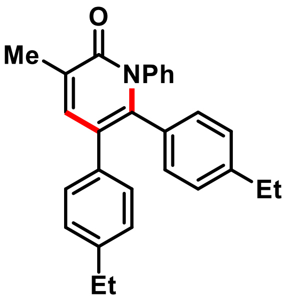

9h

5,6-Bis(4-ethylphenyl)-3-methyl-1-phenylpyridin-2(1*H*)-one (9h)

The representative procedure A was followed using phenylacrylic amide **1b** (48.4 mg, 0.3 mmol) and 1,2-bis(4-ethylphenyl)ethyne **2f** (46.9 mg, 0.2 mmol). Purification by column chromatography (*n*-hexane/EtOAc: 10/1) yielded **9h** (58.4 mg, 74%) as a yellow semisolid. **^{1}H NMR (400 MHz, $CDCl_3$)**: δ 7.46 (s, 1 H), 7.24 – 7.17 (m, 2 H), 7.16 – 7.09 (m, 1 H), 7.07 – 7.00 (m, 2 H), 6.99 – 6.89 (m, 4 H), 6.81 – 6.72 (m, 4 H), 2.55 (q, *J* = 7.6 Hz, 2 H), 2.43 (q, *J* = 7.6 Hz, 2 H), 2.27 (s, 3 H), 1.17 (t, *J* = 7.6 Hz, 3 H), 1.05 (t, *J* = 7.6 Hz, 3 H). **^{13}C NMR (100 MHz, $CDCl_3$)**: δ 163.3, 143.8, 143.6, 142.1, 140.2, 139.5, 136.0, 131.6, 130.9, 129.4, 129.1, 128.6, 128.4, 127.4, 127.3, 126.8, 119.7, 28.3, 28.3, 17.1, 15.2, 15.0. **IR** (neat): 1649, 1610, 1542, 1496, 1449, 1289, 1195, 834, 689 cm^{-1}. **HRMS** (ESI-TOF) m/z Calcd for $C_{28}H_{28}NO$ $[M+H]^+$394.2165, found 394.2158

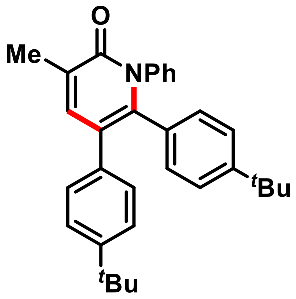

9i

5,6-Bis(4-(tert-butyl)phenyl)-3-methyl-1-phenylpyridin-2(1*H*)-one (9i)

The representative procedure A was followed using phenylacrylic amide **1b** (48.4 mg, 0.3 mmol) and 1,2-bis(4-(tert-butyl)phenyl)ethyne **2h** (58.1 mg, 0.2 mmol). Purification by column chromatography (*n*-hexane/EtOAc: 10/1) yielded **9i** (64.2 mg, 71%) as a white solid. **M. p.**: 178.4 – 184.7 ℃. **^{1}H NMR (400 MHz, $CDCl_3$)**: δ 7.47 (s, 1 H), 7.24 – 7.17 (m, 2 H), 7.16 – 7.09 (m, 3 H), 7.07 – 7.01 (m, 2 H), 6.97 – 6.88 (m, 4 H), 6.74 (d, *J* = 8.4 Hz, 2 H), 2.27 (s, 3 H), 1.23 (s, 9 H), 1.13 (s, 9 H). **^{13}C NMR (100 MHz, $CDCl_3$)**: δ 163.0, 150.5, 148.9, 143.9, 140.1, 139.5, 135.7, 131.3, 130.7, 129.1, 128.6, 128.4, 127.4, 124.6, 124.1, 119.7, 34.3, 34.3, 31.2, 31.0, 17.2. **IR** (neat): 2955, 1649, 1609, 1499, 1366, 834, 757, 697, 562 cm^{-1}. **HRMS** (ESI-TOF) m/z Calcd for $C_{32}H_{36}NO$ $[M+H]^{+}$450.2791, found 450.2784

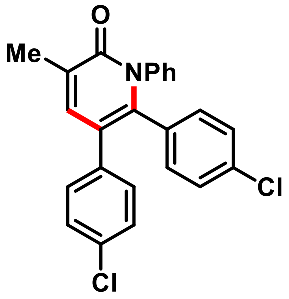

9j

5,6-Bis(4-chlorophenyl)-3-methyl-1-phenylpyridin-2(1*H*)-one (9j)

The representative procedure A was followed using phenylacrylic amide **1b** (48.4 mg, 0.3 mmol) and 1,2-bis(4-chlorophenyl)ethyne **2j** (49.4 mg, 0.2 mmol). Purification by column chromatography (*n*-hexane/EtOAc: 10/1) yielded **9j** (49.7 mg, 61%) as a white solid. **^{1}H NMR (400 MHz, $CDCl_3$)**: δ 7.39 (s, 1 H), 7.27 – 7.16 (m, 3 H), 7.13 (d, *J* = 8.4 Hz, 2 H), 7.05 – 6.99 (m, 2 H), 6.90 – 6.88 (m, 4 H), 6.78 (d, *J* = 8.4 Hz, 2 H), 2.26 (s, 3 H). **^{13}C NMR (100 MHz, $CDCl_3$)**: δ 162.8, 142.5, 139.4, 138.9, 136.7, 133.9, 132.6, 132.4, 132.1, 130.8, 129.8, 129.0, 128.8, 128.3, 127.9, 127.9, 118.7, 17.2. The spectral data are in accordance with those reported in the literature.[17]

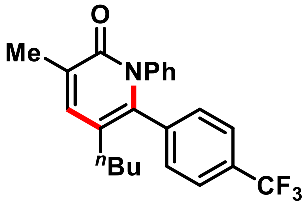

9k

5-Butyl-3-methyl-1-phenyl-6-(4-(trifluoromethyl)phenyl)pyridin-2(1*H*)-one (9k)

The representative procedure A was followed using phenylacrylic amide **1b** (48.4 mg, 0.3 mmol) and 1-(hex-1-yn-1-yl)-4-(trifluoromethyl)benzene **2r** (45.3 mg, 0.2 mmol). Purification by column chromatography (*n*-hexane/EtOAc: 10/1) yielded **9k** (35.1 mg, 46%) as a yellow solid. **M. p.**: 90.1 – 98.3 ℃ **^{1}H NMR (400 MHz, $CDCl_3$)**: δ 7.41 (d, *J* = 8.0 Hz, 2 H), 7.30 – 7.27 (m, 1 H), 7.22 – 7.16 (m, 2 H), 7.15 – 7.09 (m, 3 H), 6.97 (d, *J* = 7.2 Hz, 2 H), 2.23 (s, 3 H), 2.11

– 2.04 (m, 2 H), 1.44 – 1.34 (m, 2 H), 1.24 – 1.12 (m, 2 H), 0.78 (t, *J* = 7.2 Hz, 3 H). **^{13}C NMR (100 MHz, CDCl$_3$)**: δ 162.7, 141.4, 139.3, 139.2, 137.9, 130.70, 129.9 (q, *J* = 32.7 Hz), 129.9, 129.0, 128.7, 127.8, 124.7 (q, *J* = 3.8 Hz), 123.7 (q, *J* = 270.3 Hz), 118.1, 33.0, 30.8, 22.2, 17.2, 13.7. **^{19}F NMR (376 MHz, CDCl$_3$)** δ -62.84. **IR** (neat): 2922, 1646, 1604, 1549, 1316, 1164, 1114, 836, 758, 697 cm^{-1}. **HRMS** (ESI-TOF) m/z Calcd for $C_{23}H_{23}NOF_3$ [M+H]$^+$ 386.1726, found 386.1716

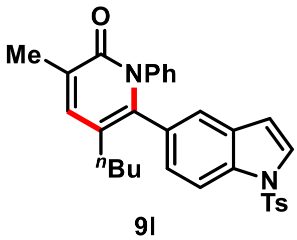

9l

5-Butyl-3-methyl-1-phenyl-6-(1-tosyl-1H-indol-5-yl)pyridin-2(1*H*)-one (9l)

The representative procedure A was followed using phenylacrylic amide **1b** (48.4 mg, 0.3 mmol) and 5-(Hex-1-yn-1-yl)-1-tosyl-1H-indole **2z** (70.3 mg, 0.2 mmol). Purification by column chromatography (*n*-hexane/EtOAc: 10/1) yielded **9l** (68.8 mg, 67%) as a yellow semisolid. **^{1}H NMR (400 MHz, CDCl$_3$)**: δ 7.75 (d, *J* = 8.4 Hz, 1 H), 7.60 (d, *J* = 8.4 Hz, 2 H), 7.45 (d, *J* = 3.6 Hz, 1 H), 7.27 (s, 1 H), 7.18 (d, *J* = 8.0 Hz, 2 H), 7.13 (d, *J* = 1.6 Hz, 1 H), 7.11 – 7.02 (m, 1 H), 7.02 – 6.84 (m, 5 H), 6.51 (d, *J* = 3.6 Hz, 1 H), 2.36 (s, 3 H), 2.22 (s, 3 H), 2.09 – 2.01 (m, 2 H), 1.39 – 1.29 (m, 2 H), 1.15 – 1.04 (m, 2 H), 0.66 (t, *J* = 7.2 Hz, 3 H). **^{13}C NMR (100 MHz, CDCl$_3$)**: δ 162.8, 144.8, 143.0, 139.6, 139.4, 134.9, 133.9, 129.9, 129.6, 129.4, 129.0, 128.9, 128.8, 128.3, 127.0, 126.7,

126.5, 123.1, 118.3, 112.8, 109.1, 32.9, 30.7, 22.1, 21.5, 17.1, 13.6. **IR** (neat): 2919, 1648, 1602, 1546, 1453, 1366, 1125, 727, 664, 580 cm^{-1}. **HRMS** (ESI-TOF) m/z Calcd for $C_{31}H_{31}N_2O_3F_3S$ $[M+H]^+$ 511.2050, found 511.2056

9. Synthesis of Rhodium(I) Sandwich Complex 10, 11

Representative Procedure B:

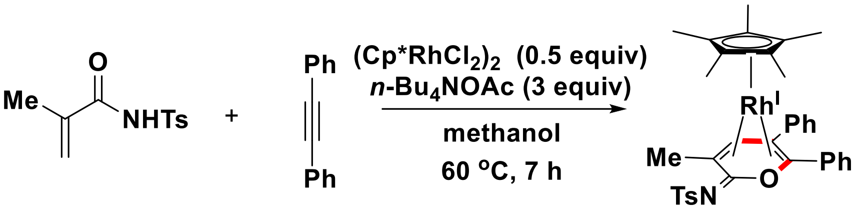

1a **2a** **10**

10: acrylic amide **1a** (28.7 mg, 0.12 mmol, 1.2 equiv), diphenylacetylene **2a** (17.8 mg, 0.1 mmol, 1.0 equiv.), *n*-Bu_4NOAc (90.5 mg, 0.3 mmol, 3.0 equiv.) and $(Cp^*RhCl_2)_2$ (30.9 mg, 0.05 mmol, 0.5 equiv.) were placed in a storage tube with 3 mL of methanol. After stirring for 7 h at 60 ℃, the solvent was removed in *vacuo*. And the residue was purified by chromatography on silica gel (*n*-hexane/EtOAc = 10:1) yielded the complex **10** (42.3 mg, 65%) as a yellow solid. **M. p.**: 165.9 – 168.0 ℃. **^{1}H NMR (400 MHz, $CDCl_3$)**: δ 7.86 – 7.79 (m, 2 H), 7.30 – 7.22 (m, 3 H), 7.21 – 7.16 (m, 4 H), 7.07 – 6.96 (m, 3 H), 6.92 – 6.87 (m, 2 H), 5.58 (s, 1H), 2.33 (s, 3 H), 1.44 (s, 15 H), 1.42 (s, 3 H). **^{13}C NMR (100 MHz, $CDCl_3$)**: δ 142.2, 140.0, 137.4, 135.2, 129.5, 129.4, 129.0, 128.7, 127.9, 127.5, 127.0, 125.5, 125.3, 96.5, 89.9, 86.5, 56.9, 21.5, 170, 9.1. **IR** (neat): 1544, 1297, 1149, 1081, 843, 770, 689, 574, 557 cm^{-1}. **HRMS** (ESI-TOF) m/z Calcd for $C_{35}H_{37}NO_3SRh$ $[M+H]^+$ 654.1544, found 654.1529.

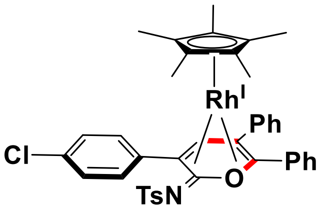

11

11: The representative procedure B was followed using acrylic amide **5m** (40.3 mg, 0.12 mmol, 1.2 equiv) and diphenylacetylene **2a** (17.8 mg, 0.1 mmol, 1.0 equiv.) for 7 h. Purification by chromatography on silica gel (*n*-hexane/EtOAc = 10:1) yielded the complex **11** (53.8 mg, 72%) as a yellow solid. **M. p.**: 169.1 – 170.5 ℃. **^{1}H NMR (400 MHz, CDCl$_3$)**: δ 7.85 – 7.80 (m, 2 H), 7.53 – 7.47 (m, 2 H), 7.41 – 7.35 (m, 3 H), 7.34 – 7.30 (m, 2 H), 7.29 – 7.25 (m, 2 H), 7.24 – 7.15 (m, 4 H), 7.15 – 7.08 (m, 3 H), 6.33 – 6.29 (m, 1 H), 2.38 (s, 3 H), 1.24 (s, 15 H). **^{13}C NMR (100 MHz, CDCl$_3$)**: δ 142.3, 139.8, 136.7, 134.6, 134.1, 131.7, 129.5, 129.0, 128.9, 128.5, 128.3, 128.0, 127.8, 126.8, 125.8, 125.3, 99.0, 97.4, 86.2, 84.3, 62.4, 21.5, 8.3. **IR** (neat): 1542, 1491, 1292, 1267, 1080, 1021, 817, 683, 545cm^{-1}. **HRMS** (ESI-TOF) m/z Calcd for $C_{40}H_{38}NO_3SClRh$ $[M+H]^+$ 750.1311, found 750.1293.

10. Synthesis of Lactone 3aa From Cyclic Imidate 3a[25]

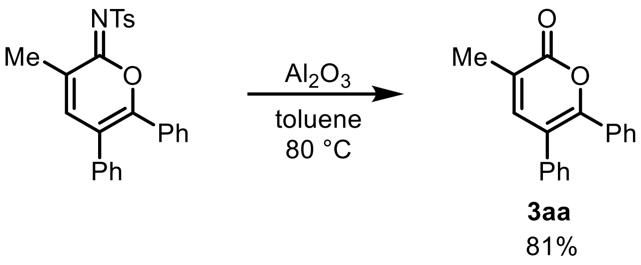

To a open flask was added basic aluminium oxide (34.0 gram) and iminoester (1.42 g, 3.40 mmol, 1 equiv), and then added toluene. The mixture was heated in oil bath at 80 ℃ for 12 h, then filtrated and washed with DCM three times (3 x 25 ml), The organic layer was dried over Na_2SO4, concentrated in vacuo. The residue was subjected to column chromatography on silica gel to deliver corresponding lactone **3aa** (0.72 mg, 81%).

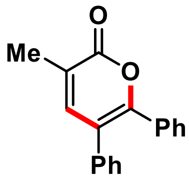

3aa

3-Methyl-5,6-diphenyl-2*H*-pyran-2-one (3aa)

^{1}H NMR (400 MHz, $CDCl_3$): δ 7.35 – 7.30 (m, 2 H), 7.29 – 7.26 (m, 3 H), 7.26 – 7.22 (m, 2 H), 7.17 – 7.12 (m, 4 H), 2.17 (s, 3 H). **^{13}C NMR (100 MHz, $CDCl_3$)**: δ 163.2, 155.3, 144.1, 136.5, 132.2, 129.5, 129.2, 129.0, 128.9, 128.1, 127.8, 123.7, 118.0, 16.5. The spectral data are in accordance with those reported in the literature.[5]

11. Preliminary Mechanistic Studies

(1) Catalytic Reactivity of 10

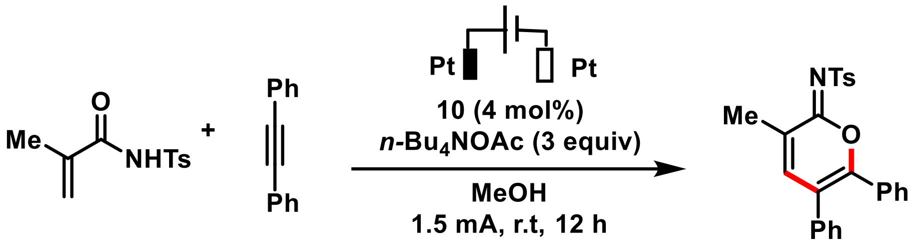

1a 2a 3a, 93%

The electrocatalysis was carried out in an undivided cell equipped with two platinum electrodes (1.0×1.0 cm^2). methacrylic amide **1a** (71.7 mg, 0.3 mmol, 1.5 equiv), diphenylacetylene **2a** (35.7mg, 0.2 mmol, 1.0 equiv), *n*-Bu_4NOAc (180.9 mg, 0.6 mmol, 3.0 equiv) and **10** (5.2 mg, 4.0 mol %) were dissolved in MeOH (3.0 mL). Electrocatalysis was performed at room temperature with a constant current of 1.5 mA maintained for 12 h. After the reaction, the reaction mixture was concentrated in vacuo. The resulting residue was purified on silica gel (*n*-hexane/EtOAc = 10:1) yielded **3a** (77.0 mg, 93%) as a yellow solid.

(2) Stoichiometric Reactions of Rhodacycle 10 with anodic oxidation

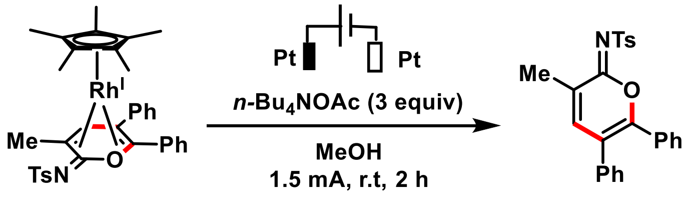

10 3a, 83%

The electrocatalysis was carried out in an undivided cell equipped with two platinum electrodes (1.0×1.0 cm^2). Rhodium cycle **10** (130.7 mg, 0.2 mmol, 1.0 equiv) and *n*-Bu_4NOAc (180.9 mg, 0.6 mmol, 3.0 equiv) were dissolved in MeOH (3.0 mL). Electrocatalysis was performed at room temperature with a

constant current of 1.5 mA maintained for 2 h. After the reaction, the reaction mixture was concentrated in vacuo. The resulting residue was purified on silica gel (*n*-hexane/EtOAc = 10:1) yielded **3a** (65.3 mg, 83%) as a yellow solid.

12. Deuterium-Labeled Experiments

H/D exchange in 1a

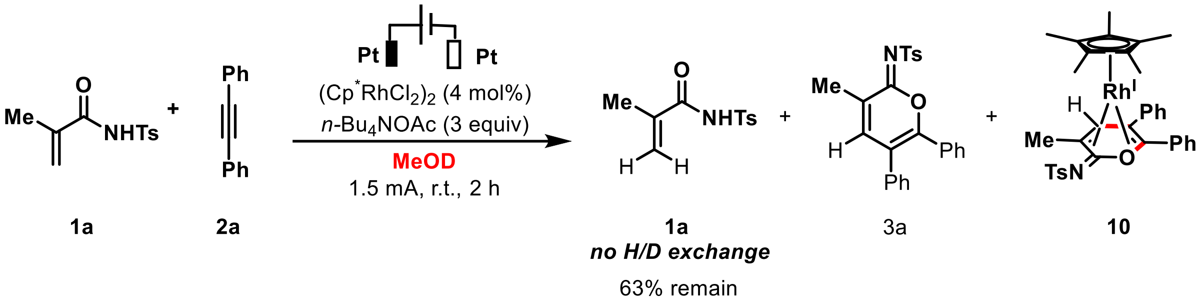

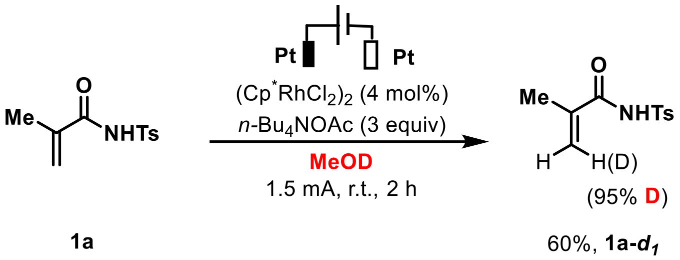

The electrocatalysis was carried out in an undivided cell equipped with two platinum electrodes (1.0×1.0 cm^2). methacrylic amide **1a** (71.7 mg, 0.3 mmol, 1.5 equiv) with diphenylacetylene **2a** (35.7mg, 0.2 mmol, 1.0 equiv), *n*-Bu_4NOAc (180.9 mg, 0.6 mmol, 3.0 equiv) and $(Cp^*RhCl_2)_2$ (5.0 mg, 4.0 mol%) were dissolved in MeOD (3.0 mL). Electrocatalysis was performed at room temperature with a constant current of 1.5 mA maintained for 2 h. After the reaction, the reaction mixture was concentrated in vacuo. The resulting residue was purified on silica gel (*n*-hexane/EtOAc = 10:1) recovered **1a** (44.5 mg, 63% remain).

The electrocatalysis was carried out in an undivided cell equipped with two platinum electrodes (1.0×1.0 cm^2). methacrylic amide **1a** (71.7 mg, 0.3 mmol, 1.5 equiv), *n*-Bu_4NOAc (180.9 mg, 0.6 mmol, 3.0 equiv) and $(Cp^*RhCl_2)_2$ (5.0 mg, 4.0 mol%) were dissolved in MeOD (3.0 mL).

Electrocatalysis was performed at room temperature with a constant current of 1.5 mA maintained for 2 h. After the reaction, the reaction mixture was concentrated in vacuo. The resulting residue was purified on silica gel (*n*-hexane/EtOAc = 10:1) yielded **1a-*d*$_1$** (43.2 mg, 60%). The D% incorporation of recovered starting material was determined by ^{1}H NMR.

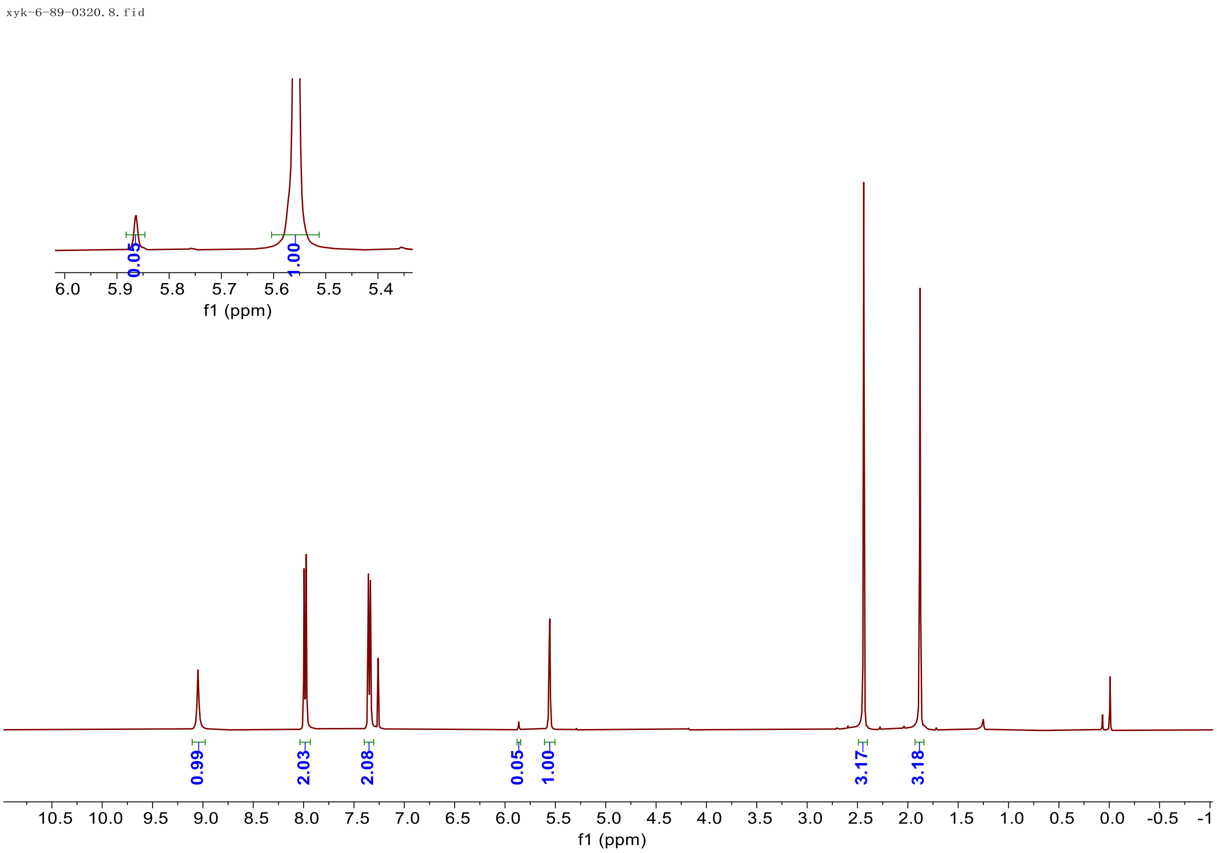

13. Kinetic Isotope Effect Studies

a) Intermolecular competition experiment:

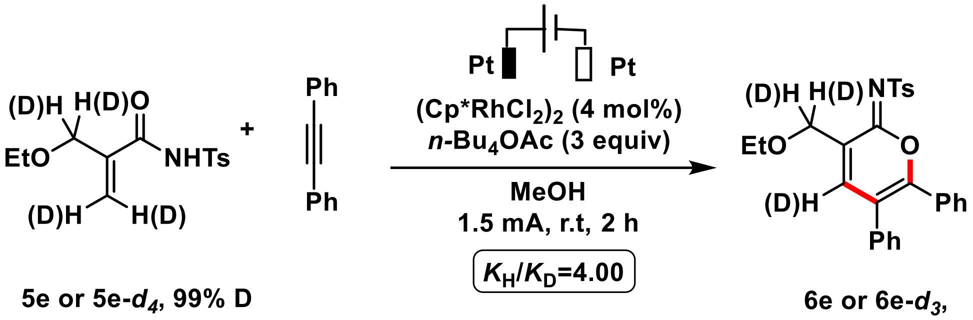

5e or 5e-d_4, 99% D

6e or 6e-d_3, yield 28.5%

According to general procedure, in an electrochemical undivided cell, **5e** (42.5 mg, 0.15 mmol), **5e-*d*4** (99% D, 43.1 mg, 0.15 mmol) with diphenylacetylene **2a** (35.7 mg, 0.2 mmol, 1.0 equiv), $(Cp^*RhCl_2)_2$ (5.0 mg, 4.0 mol %) and *n*-Bu_4NOAc (180.9 mg, 0.6 mmol, 3.0 equiv) were dissolved in MeOH (6 mL). Electrolysis was conducted at a constant current of 1.5 mA at room temperature for 2 h. The reaction mixture was concentrated in vacuo. The resulting residue was purified by silica gel flash chromatography to give the mixture products 6e and 6e-d_3. The ratio of **6e/6e-d3** was determined by ^{1}H NMR analysis to give a KIE value of **4.00**.

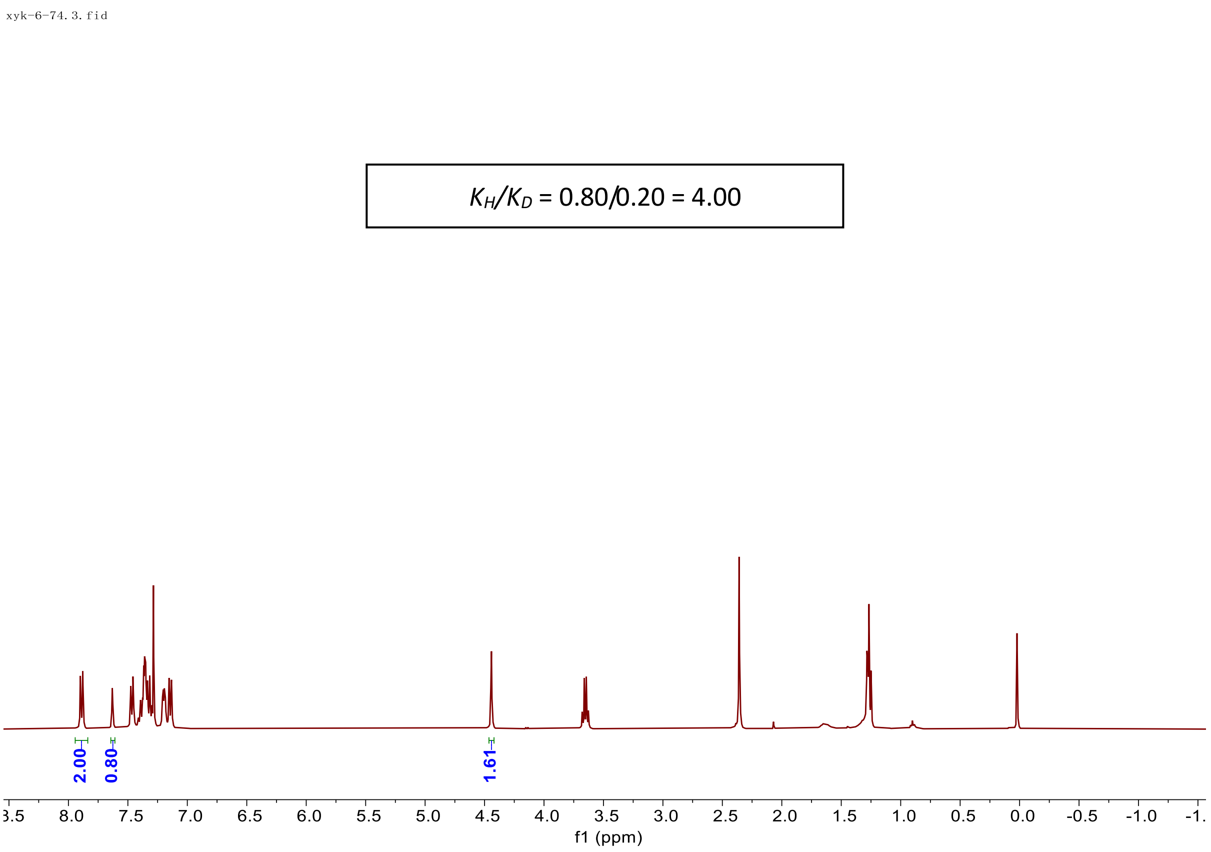

b) Parallel experiments:

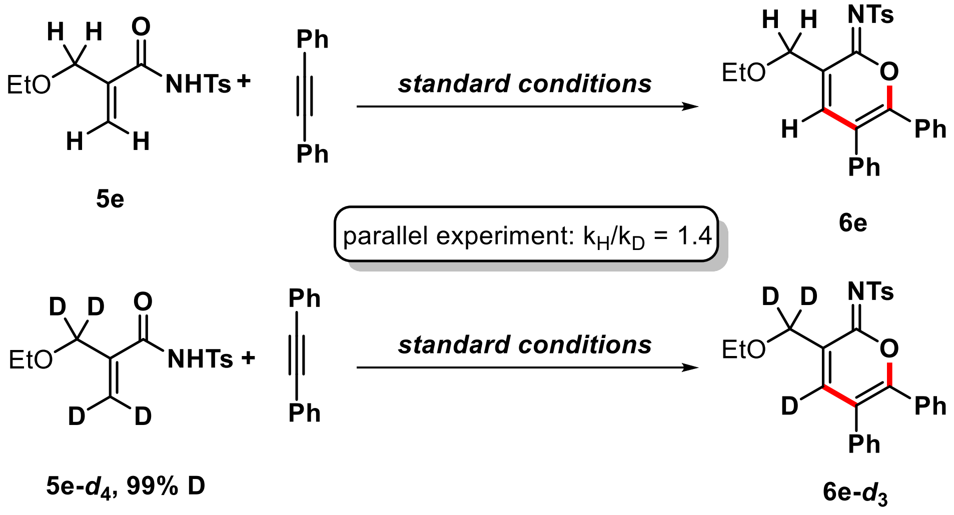

According to general procedure, an undivided cell was charged with **5e** (85.0 mg, 0.3 mmol) with diphenylacetylene **2a** (35.7mg, 0.2 mmol, 1.0 equiv), $(Cp^*RhCl_2)_2$ (5.0 mg, 4.0 mol %) and *n*-Bu_4NOAc (180.9 mg, 0.6 mmol, 3.0

equiv) were dissolved in MeOH (6 mL). Electrolysis was conducted at a constant current of 1.5 mA at room temperature and stopped respectively at 10 min, 20 min, 30 min, 40 min and 50 min. In similar, substrate **5e-*d*$_4$** (99% D, 86.2 mg, 0.3 mmol) was used instead of **5e** for the reaction. The yield of products was determined by ^{1}H NMR with 1,4-Dimethoxybenzene as internal standard and the reaction rate was obtained by plotting the percentage yield of the product versus time. The kinetic isotope effect (k_H/k_D) was determined to be 1.4.

Time (min)	10	20	30	40	50
Yield **6e** (%)	3.3	6.5	9.7	14.2	15.9
Yield **[d$_3$]-6e** (%)	2.7	6.3	8.2	10.6	12.0

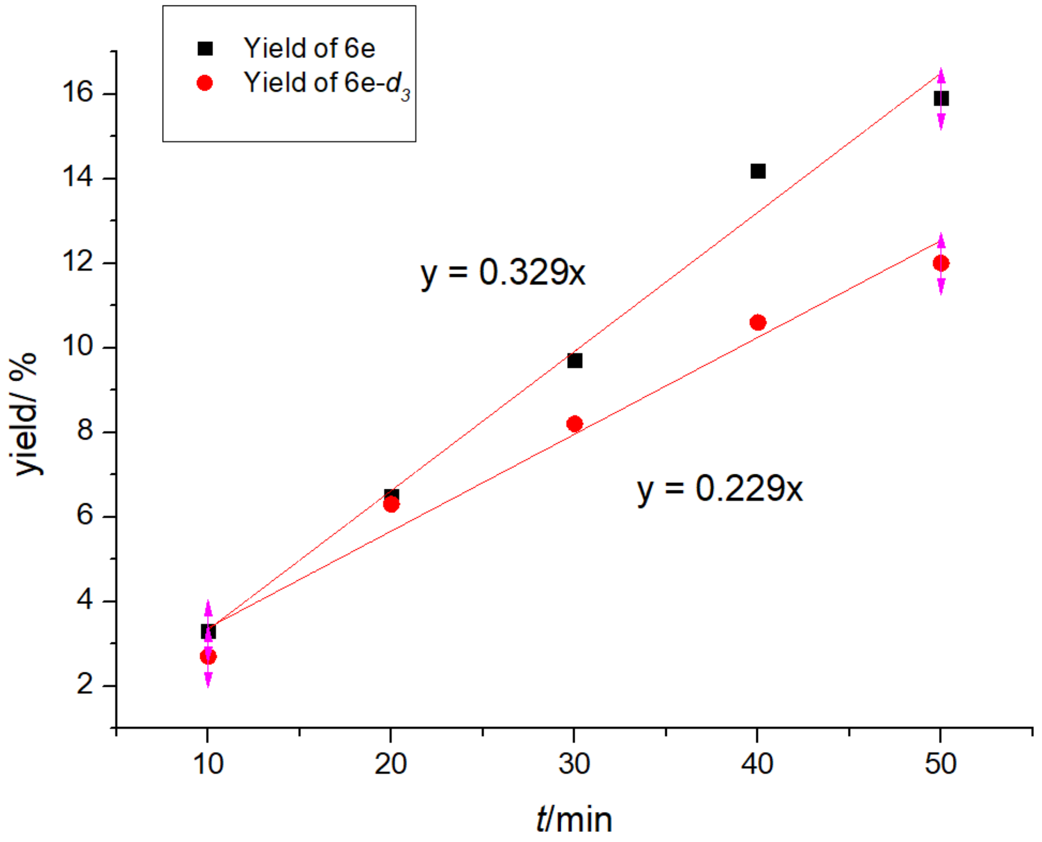

Supplementary Figure 3. **Kinetic Isotope Effect Studies: Parallel experiments**

14. General Procedure for Kinetic Experiments

Kinetics experiments were run in an electrochemical undivided cell, Reactions were run up to about 3–20% conversion, and the data ([product] versus time) was analyzed using the initial rates method. The reported value of initial rate is the average of the two kinetic experiments, and the reported error in the initial rate is the standard deviation of those two kinetic experiments.

1) Order in acrylamide

The order in **1a** was determined by studying the initial rate of reactions with different **[1a]**. The electrocatalysis was carried out in an undivided cell equipped

with two platinum electrodes (1.0×1.0 cm^2). methacrylic amide **1a** (0.15 – 0.75 mmol) with diphenylacetylene 2a (35.7mg, 0.2 mmol), *n*-Bu_4NOAc (180.9 mg, 0.6 mmol) and $(Cp^*RhCl_2)_2$ (5.0 mg, 4.0 mol%) were dissolved in MeOH (6.0 mL). Electrolysis was conducted at a constant current of 1.5 mA at room temperature for one hour. The yield of products was determined by ^{1}H NMR with 1,4-Dimethoxybenzene as internal standard and each reported initial rate represents an average of two experiments.

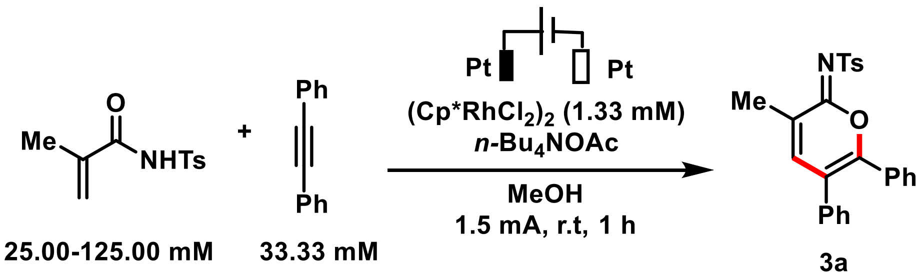

Entry	**1a** (mmol)	**[1a]** mM	Yield 3a (%)	Initial rate [mM/h]
1	0.15	25.0	13.4	4.5
2	0.30	50.0	13.7	4.6
3	0.45	75.0	13.9	4.6
4	0.60	100.0	14.2	4.7
5	0.75	125.0	14.7	4.9

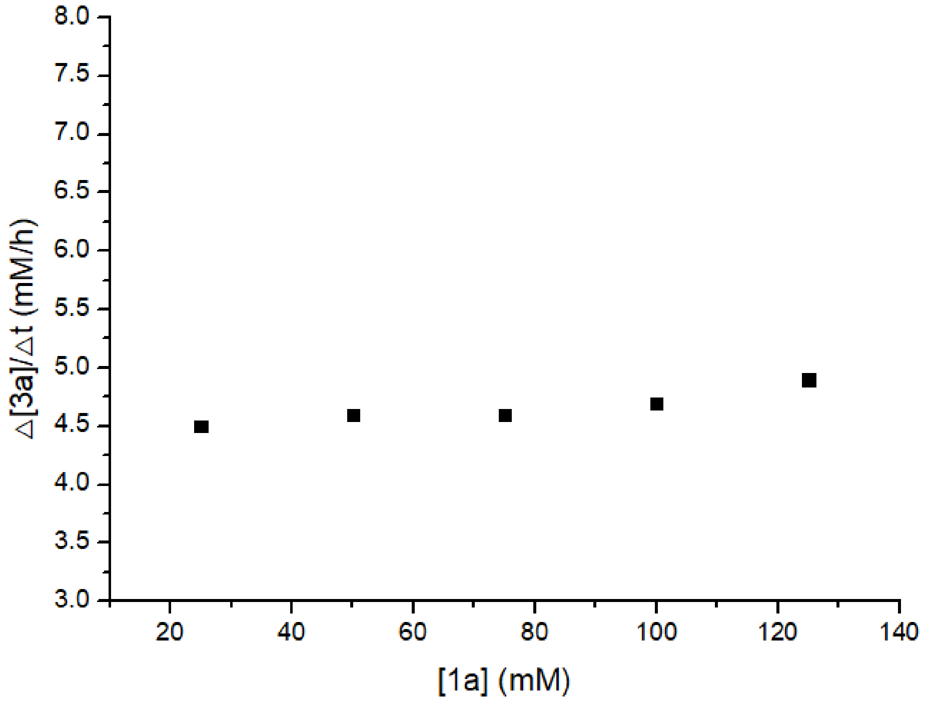

Supplementary Figure 4. **Kinetic experiment of acrylamide 1a**

Note: The zero order of acrylamide 1a was observed.

2) Order in alkyne

The order in **2a** was detemined by studying the initial rate of reactions with different **[2a]**. The electrocatalysis was carried out in an undivided cell equipped with two platinum electrodes (1.0×1.0 cm^2). methacrylic amide **1a** (71.7 mg, 0.3 mmol) with diphenylacetylene 2a (16.7 – 50 mmol), *n*-Bu_4NOAc (180.9 mg, 0.6 mmol) and $(Cp^*RhCl_2)_2$ (5.0 mg, 4.0 mol%) were dissolved in MeOH (6.0 mL). Electrolysis was conducted at a constant current of 1.5 mA at room temperature for one hour. The yield of products was determined by ^1H NMR with 1,4-Dimethoxybenzene as internal standard and each reported initial rate represents an average of two experiments.

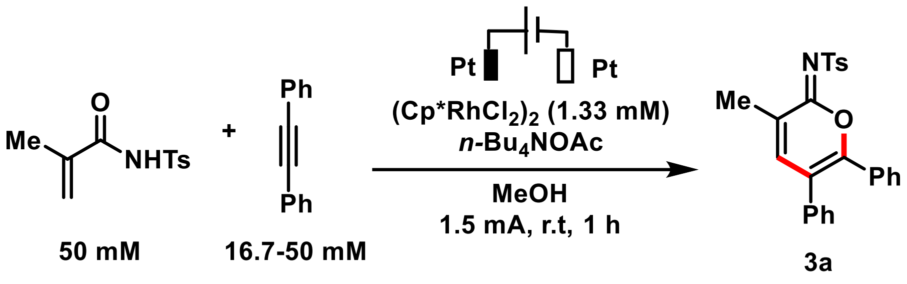

Entry	**2a** (mmol)	**[2a]** mM	Yield 3a (%)	Initial rate [mM/h]
1	0.10	16.7	12.9	4.3
2	0.15	25.0	12.3	4.1
3	0.20	33.3	13.4	4.5
4	0.25	41.7	14.0	4.7
5	0.30	50.0	13.2	4.4

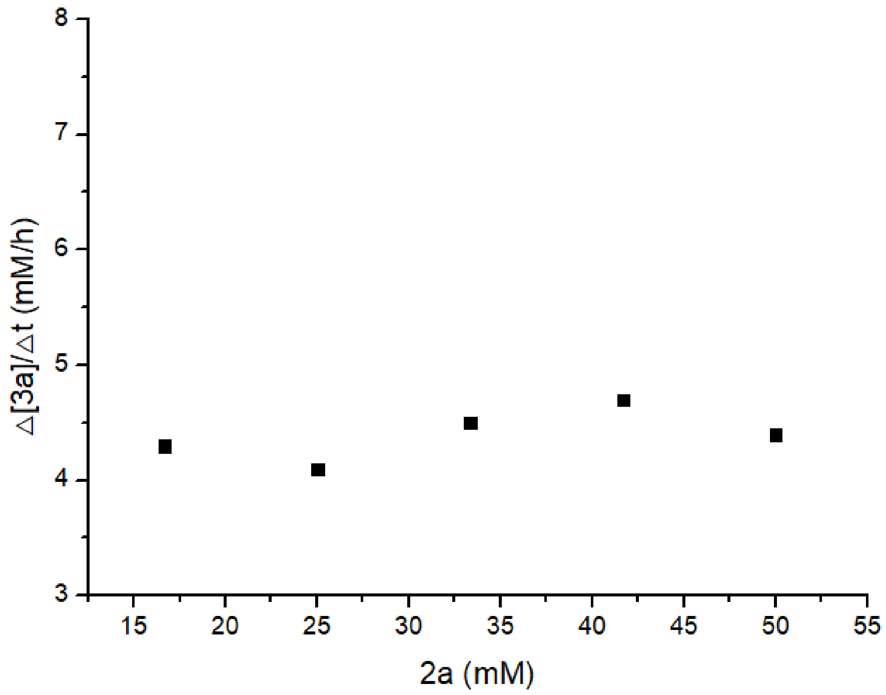

***Supplementary Figure 5*. Kinetic experiment of alkyne 2a**

Note: The zero order of acrylamide 2a was observed.

3) Order in $(Cp^*RhCl_2)_2$

The order in **$(Cp^*RhCl_2)_2$** was detemined by studying the initial rate of reactions with different **$(Cp^*RhCl_2)_2$**. The electrocatalysis was carried out in an undivided cell equipped with two platinum electrodes (1.0×1.0 cm^2). methacrylic amide **1a** (71.7 mg, 0.3 mmol) with diphenylacetylene 2a (35.7 mg, 0.2 mmol), *n*-Bu_4NOAc (180.9 mg, 0.6 mmol) and $(Cp^*RhCl_2)_2$ (0.004 – 0.032 mmol) were dissolved in MeOH (6.0 mL). Electrolysis was conducted at a constant current of 1.5 mA at room temperature for one hour. The yield of products was determined by ^1H NMR with 1,4-Dimethoxybenzene as internal standard and each reported initial rate represents an average of two experiments.

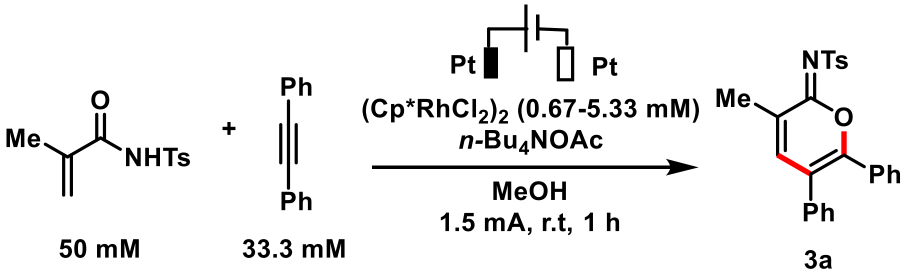

Entry	**[Rh]** (mmol)	**[Rh]** mM	Yield 3a (%)	Initial rate [mM/h]
1	0.004	0.67	8.5	2.8
2	0.008	1.33	14.1	4.7
3	0.012	2.00	17.9	6.0
4	0.016	2.67	20.9	7.0

5	0.020	3.33	20.2	6.7
6	0.024	4.00	21.3	7.1
7	0.028	4.67	20.7	6.9
8	0.032	5.33	21.9	7.3

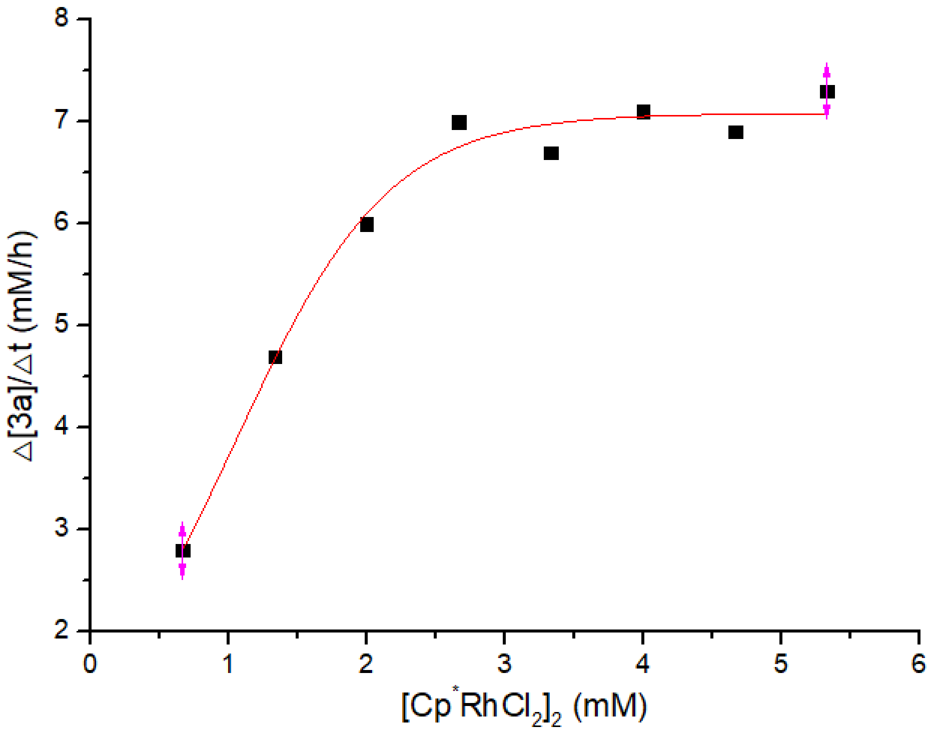

Supplementary Figure 6. **Kinetic experiment of catalyst ($(Cp^*RhCl_2)_2$)**

Note: Saturation kinetics was observed. The kinetic order of **$(Cp^*RhCl_2)_2$** was measured to be first-order only at very low concentration (< 2.0 mM).

15. Visual Kinetic Analysis

According to general procedure, an undivided cell was charged with **1a** (71.7 mg, 0.3 mmol) with diphenylacetylene **2a** (35.7mg, 0.2 mmol), $(Cp^*RhCl_2)_2$ (2.5 mg, 2.0 mol %) or (5.0 mg, 4.0 mol %) and *n*-Bu_4NOAc (180.9 mg, 0.6 mmol, 3.0 equiv) were dissolved in MeOH (6 mL). Electrolysis was conducted at a constant current of 1.5 mA at room temperature and stopped respectively at different time. The data obtained is shown in below.

First, the time scale was normalized assuming a first order in catalyst, an excellent overlay could be achieved. In contrast, no overlay could be achieved for an assumed second order dependence on $(Cp^*RhCl_2)_2$ as is shown in below.

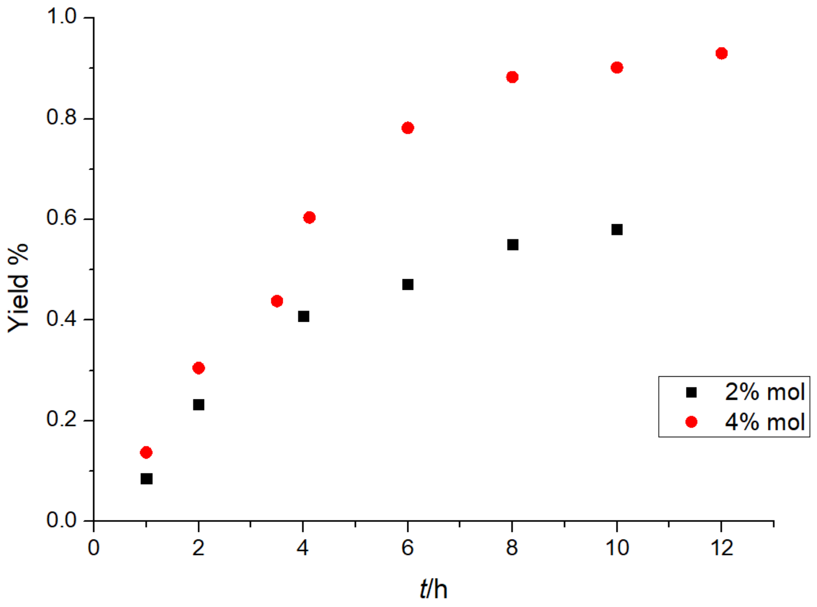

Supplementary Figure 7. **Data obtained without correction for the catalyst loading**

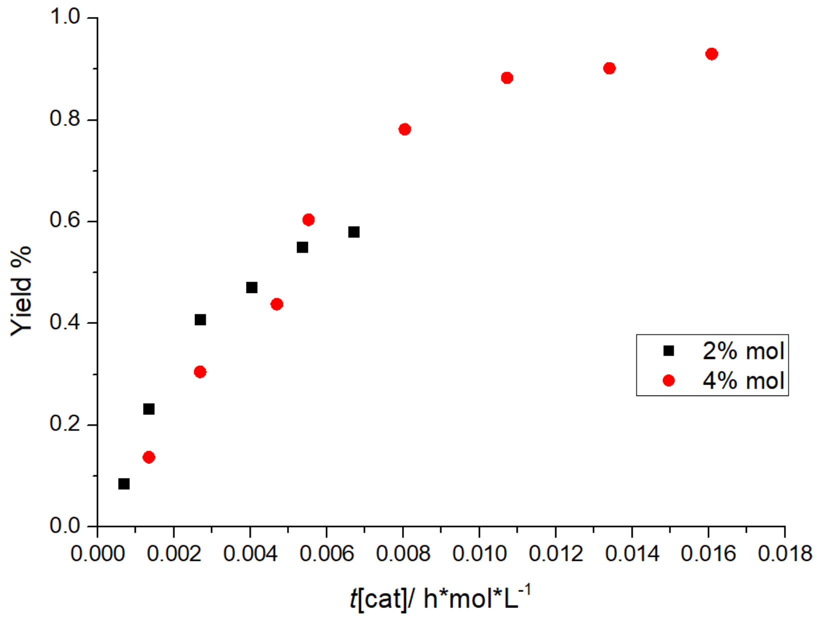

Supplementary Figure 8. **Excellent overlay is achieved for a first order in catalyst**

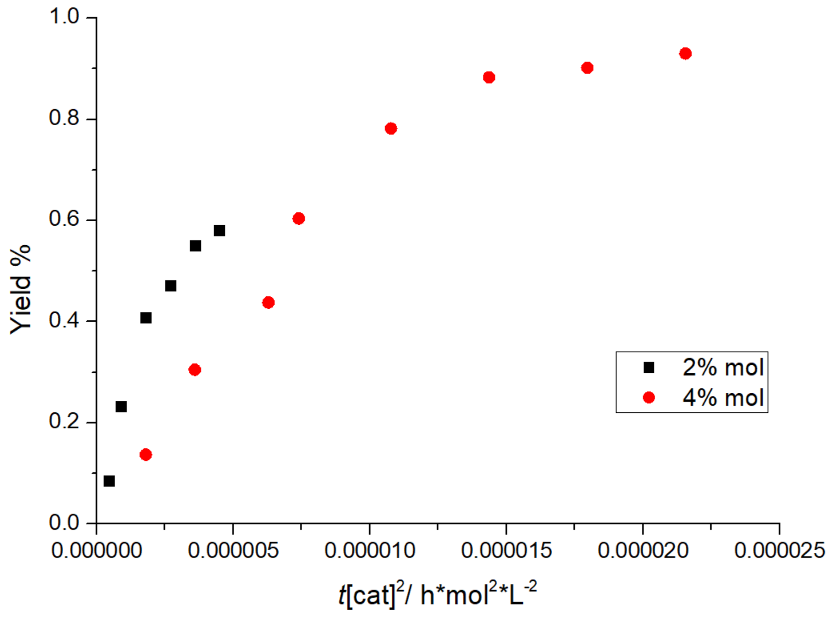

Supplementary Figure 9. **No overlay was achieved for a second order in catalyst**

16. Effect of current on annulation reaction.

According to general procedure, an undivided cell was charged with **1a** (71.7 mg, 0.3 mmol) with diphenylacetylene **2a** (35.7mg, 0.2 mmol), $(Cp^*RhCl_2)_2$ (5.0 mg, 4.0 mol %) and *n*-Bu_4NOAc (180.9 mg, 0.6 mmol, 3.0 equiv) were dissolved in MeOH (6 mL). Electrolysis was conducted respectively at a constant current of 0.5 mA, 1 mA, 1.5 mA, 2 mA, 2.5 mA at room temperature for different hours. The yield of products was determined by ^1H NMR with 1,4-Dimethoxybenzene as internal standard and the reaction rate was obtained by plotting the percentage yield of the product versus current.

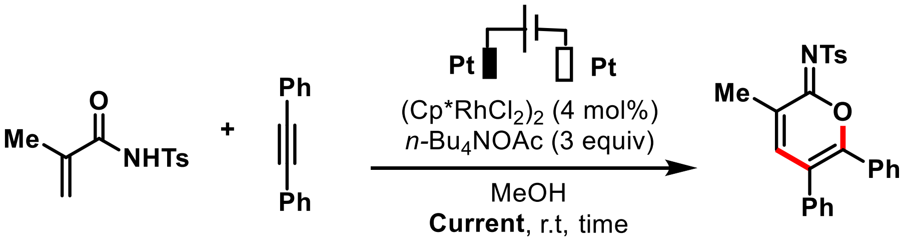

1a 2a 3a

Current (0.5 mA)

Time(h)	2	4	6	8	10
Yield of 3a (%)	9.8	15.0	25.2	30.9	38.4

Current (0.8 mA)

Time(h)	2	4	6	8	10
Yield of 3a (%)	17.6	32.9	47.6	59.9	64.3

Current (1.0 mA)

Time(h)	1	2	3	4	6
Yield of 3a (%)	8.8	19.5	26.5	34.7	49.9

Current (1.5 mA)

Time(h)	1	2	3	4	6
Yield of 3a (%)	13.7	30.5	38.4	49.0	78.2

Current (2.0 mA)

Time(h)	1	2	3	4	6
Yield of 3a (%)	17.3	37.3	41.4	53.8	89.9

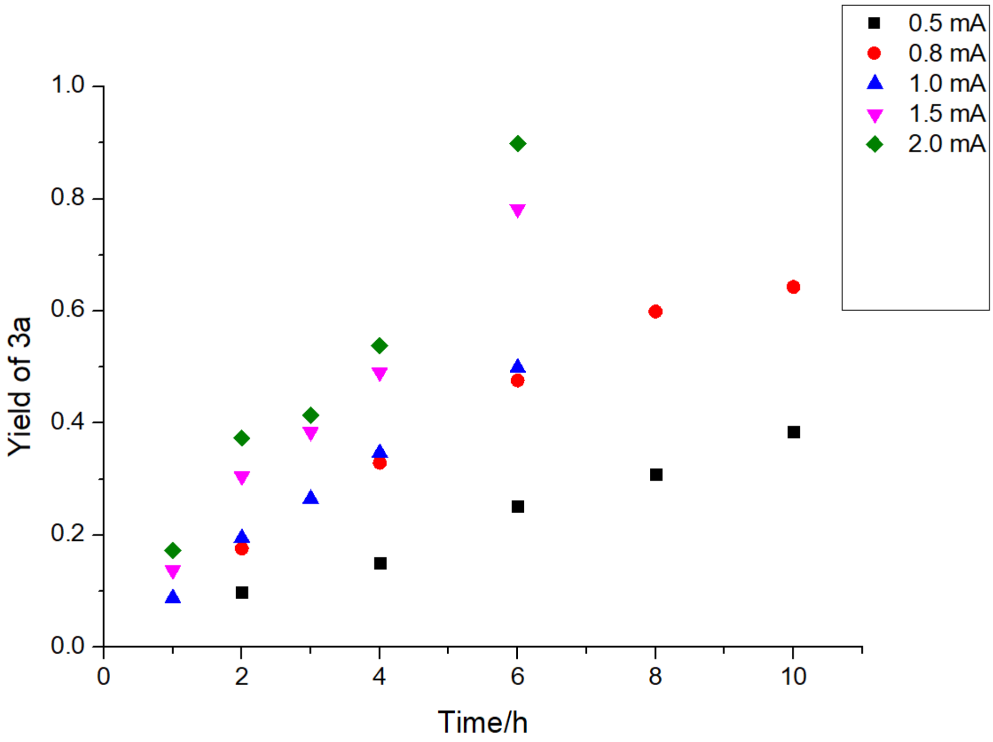

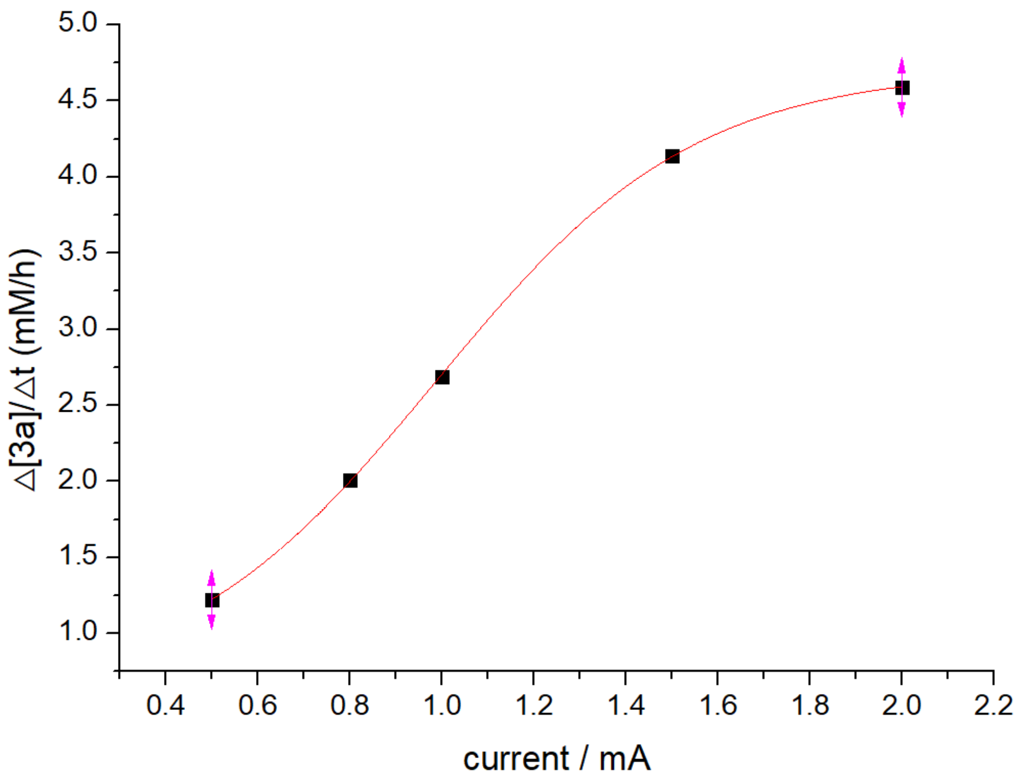

Supplementary Figure 10. **Effect of current on annulation reaction**

17. Electrochemical Procedure for Cyclic Voltammetry

Cyclic voltammograms were recorded with a CHI660E potentiostat at room temperature in MeCN. *n*-Bu_4NPF_6 (0.1 M) was used as the supporting electrolyte, and a Pt electrode (area = 0.03 cm^2) was used as the working electrode. The auxiliary electrode was a Pt sheet. A saturated calomel electrode (SCE) was used as reference electrode and all potentials are expressed vs. this reference system.

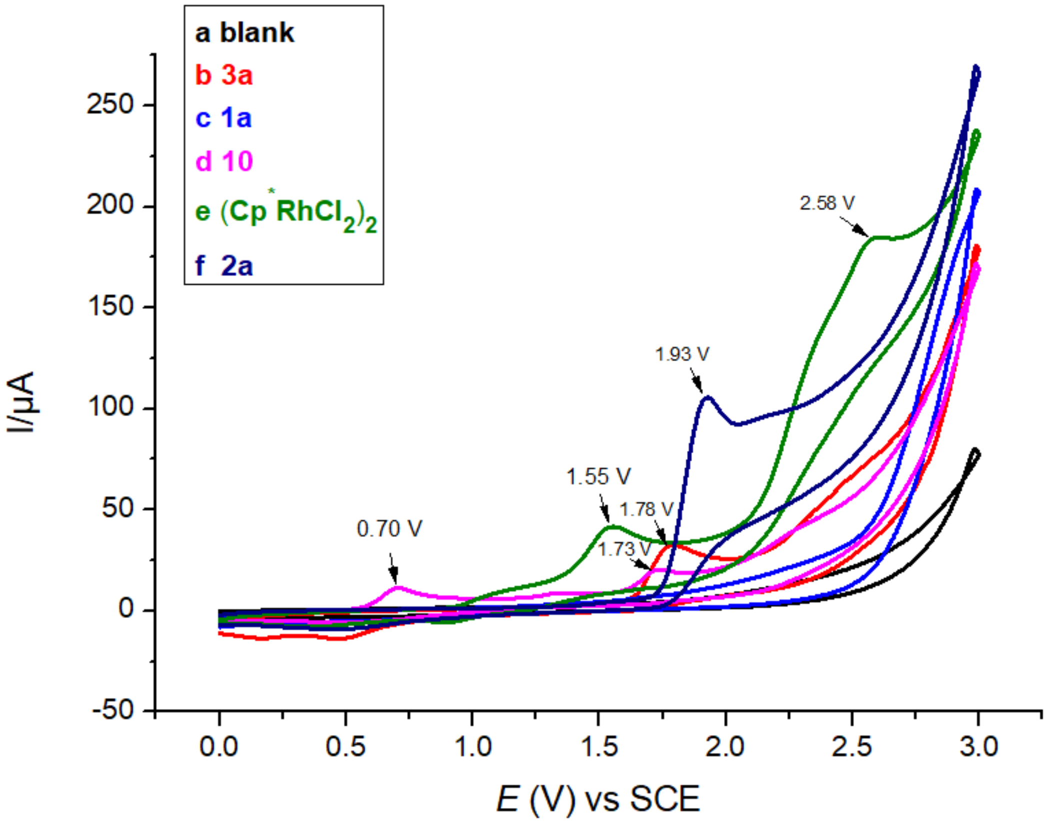

***Supplementary Figure 11*. Cyclic voltametric study.** Cyclic voltammograms recorded on a Pt electrode (area = 0.03 cm^2), The scan rate was 100 mV s^{-1}: (a) MeCN containing 0.1 M *n*-Bu_4NPF_6; (b) MeCN containing 0.1 M *n*-Bu_4NPF_6, after addition of 4 mM 3a; (c) MeCN containing 0.1 M *n*-Bu_4NPF_6, after addition of 4 mM 1a; (d) MeCN containing 0.1 M *n*-Bu_4NPF_6, after addition of 4 mM 10; (e) MeCN containing 0.1 M *n*-Bu_4NPF_6, after addition of 4 mM complex ($Cp^*RhCl_2)_2$; (f) MeCN containing 0.1 M *n*-Bu_4NPF_6, after addition of 4 mM **2a**.

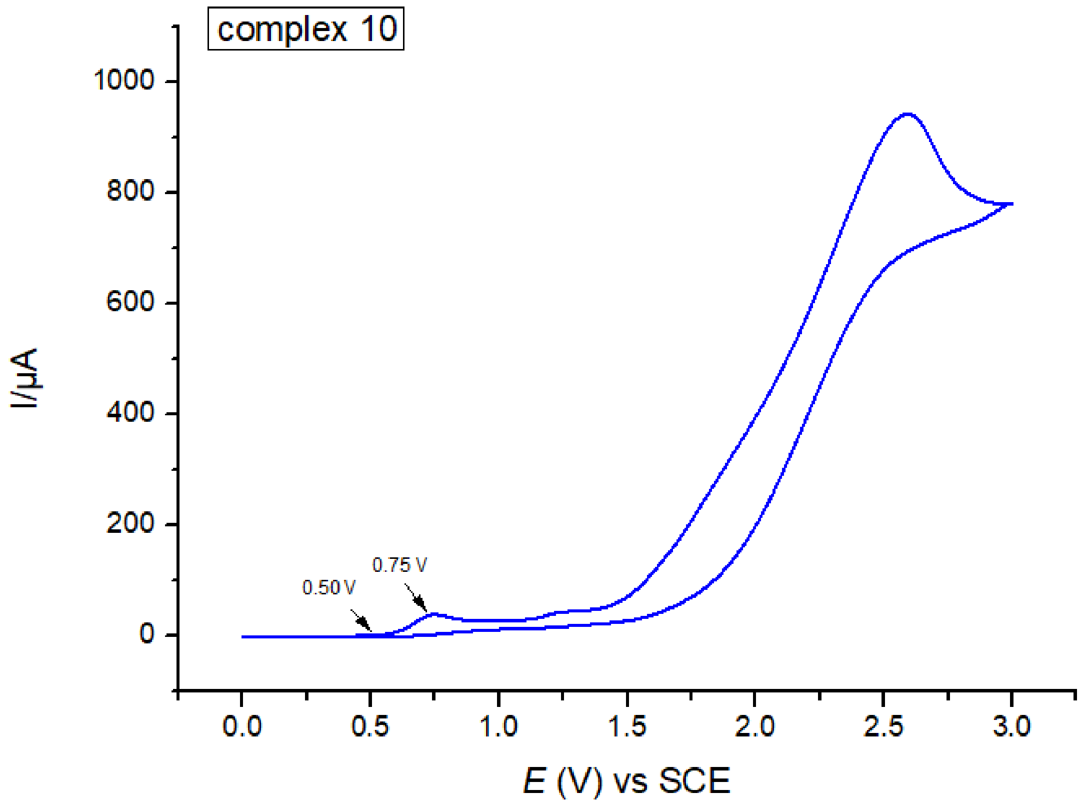

Supplementary Figure 12. **Cyclic voltametric study with *n*-Bu$_4$NOAc as electrolyte.** Cyclic voltammograms recorded on a Pt electrode (area = 0.03 cm^2), The scan rate was 100 mV s^{-1}: MeCN containing 0.1 M *n*-Bu$_4$NOAc, after addition of 4 mM **10**

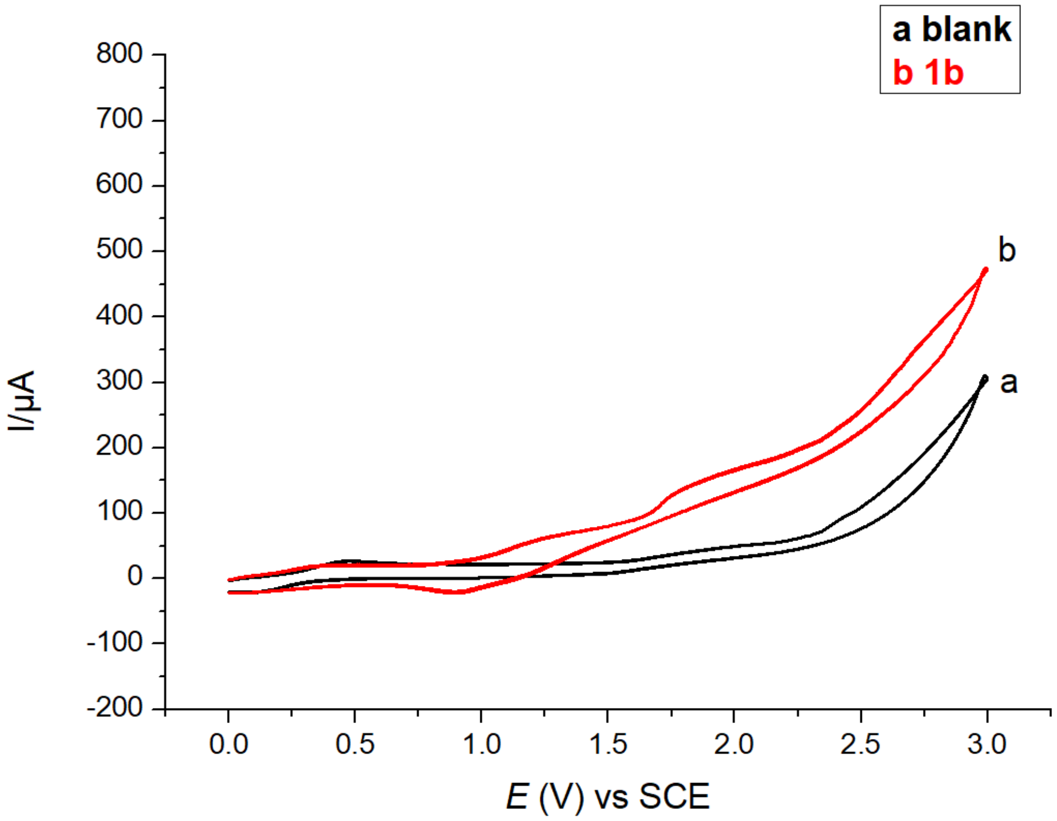

***Supplementary Figure 13*. Cyclic voltametric study of substrate 1b.** Cyclic voltammograms recorded on a Pt electrode (area = 0.03 cm^2), The scan rate was 100 mV s^{-1}: MeCN containing 0.1 M *n*-Bu_4NPF_6, after addition of 4 mM **1b**.

18. X-ray crystal structure data

Compound 3a (CCDC 1967781)

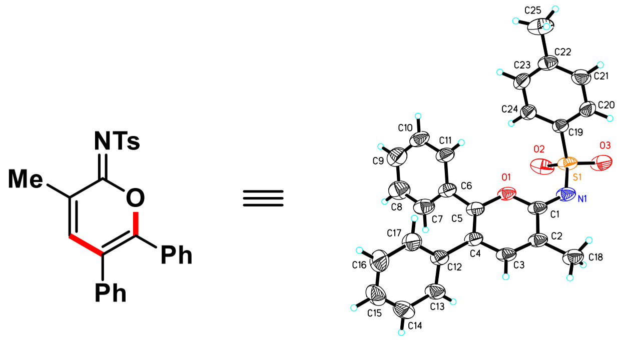

3a

Empirical formula	$C_{25}H_{21}NO_3S$
Formula weight	415.49
Temperature	293(2) K
Wavelength	0.71073 Å
Crystal system	Monoclinic
Space group	P 21/c
Unit cell dimensions	a = 13.2203(5) Å, α = 90 °.<br>b = 12.3690(5) Å, β = 117.039(2) °.<br>c = 14.4410(7) Å, γ = 90 °.
Volume	2103.31(16) $Å^3$
Z	4
Density (calculated)	1.312 Mg/m^3
Absorption coefficient	0.181 mm^{-1}
F(000)	872
Crystal size	0.200 x 0.170 x 0.130 mm^3
Theta range for data collection	3.272 to 25.997 °.
Index ranges	-16<=h<=14, -13<=k<=15, -16<=l<=17
Reflections collected	10161
Independent reflections	4088 [R(int) = 0.0264]
Completeness to theta = 25.242 °	98.9 %
Absorption correction	Semi-empirical from equivalents
Max. and min. transmission	0.7456 and 0.6484
Refinement method	Full-matrix least-squares on F^2
Data / restraints / parameters	4088 / 1 / 274

Goodness-of-fit on F^2	1.031
Final R indices [I>2sigma(I)]	R1 = 0.0479, wR2 = 0.1073
R indices (all data)	R1 = 0.0790, wR2 = 0.1281
Extinction coefficient	0.023(4)
Largest diff. peak and hole	0.172 and -0.224 e.Å^{-3}

Compound 6c (CCDC 1967779)

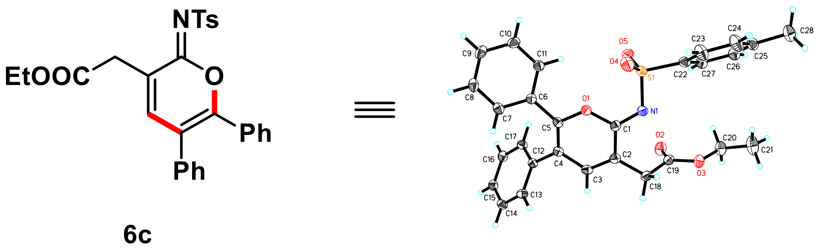

6c

Empirical formula	$C_{28}H_{25}NO_5S$
Formula weight	487.55
Temperature	193(2) K
Wavelength	0.71073 Å
Crystal system	Monoclinic
Space group	C 2/c
Unit cell dimensions	a = 16.1340(4) Å, α = 90 °.<br>b = 8.5734(2) Å, β = 98.3730(10) °.<br>c = 35.0069(8) Å, γ = 90 °.
Volume	4790.7(2) Å^3
Z	8
Density (calculated)	1.352 Mg/m^3
Absorption coefficient	0.176 mm^{-1}
F(000)	2048
Crystal size	0.200 x 0.160 x 0.120 mm^3
Theta range for data collection	2.552 to 25.999 °.
Index ranges	-19<=h<=19, -10<=k<=10, -41<=l<=43
Reflections collected	23691
Independent reflections	4706 [R(int) = 0.0508]
Completeness to theta = 25.242 °	99.7 %

Absorption correction	Semi-empirical from equivalents
Max. and min. transmission	0.7456 and 0.5733
Refinement method	Full-matrix least-squares on F^2
Data / restraints / parameters	4706 / 0 / 319
Goodness-of-fit on F^2	1.057
Final R indices [I>2sigma(I)]	R1 = 0.0395, wR2 = 0.0915
R indices (all data)	R1 = 0.0530, wR2 = 0.1016
Extinction coefficient	0.0024(3)
Largest diff. peak and hole	0.238 and -0.313 e.Å^{-3}

Compound 6o (CCDC 1967782)

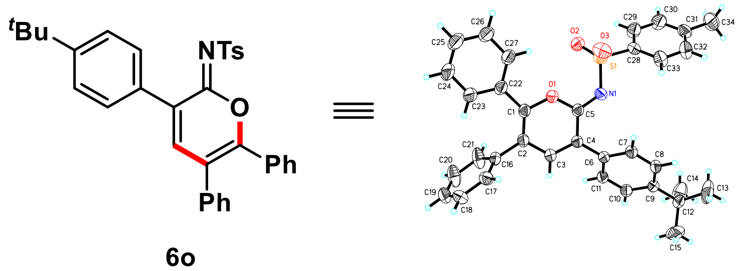

6o

Empirical formula	$C_{34}H_{31}NO_3S$
Formula weight	533.66
Temperature	293(2) K
Wavelength	0.71073 Å
Crystal system	Monoclinic
Space group	P 21/n
Unit cell dimensions	a = 10.7212(10) Å, α = 90 °.<br>b = 19.633(2) Å, β = 108.654(4) °.<br>c = 14.7063(18) Å, γ = 90 °.
Volume	1717.82(6) Å^3
Z	4
Density (calculated)	1.209 Mg/m^3
Absorption coefficient	0.144 mm^{-1}

F(000)	1128
Crystal size	0.200 x 0.160 x 0.130 mm^3
Theta range for data collection	2.538 to 25.993 °.
Index ranges	-13<=h<=13, -24<=k<=23, -15<=l<=18
Reflections collected	14543
Independent reflections	5742 [R(int) = 0.0333]
Completeness to theta = 25.242 °	99.5 %
Absorption correction	Semi-empirical from equivalents
Max. and min. transmission	0.7456 and 0.6777
Refinement method	Full-matrix least-squares on F^2
Data / restraints / parameters	5742 / 18 / 357
Goodness-of-fit on F^2	1.023
Final R indices [I>2sigma(I)]	R1 = 0.0557, wR2 = 0.1400
R indices (all data)	R1 = 0.0854, wR2 = 0.1657
Extinction coefficient	0.016(3)
Largest diff. peak and hole	0.314 and -0.215 e.$Å^{-3}$

Compound 7r (CCDC 1967777)

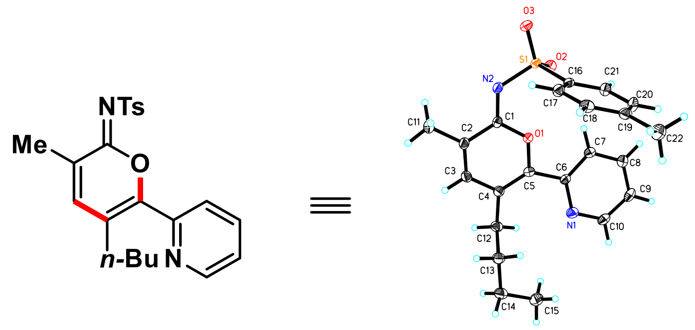

7r

Empirical formula	$C_{22}H_{24}N_2O_3S$
Formula weight	396.49
Temperature	193(2) K
Wavelength	0.71073 Å
Crystal system	Monoclinic
Space group	P 21/c

Unit cell dimensions	a = 8.0987(4) Å, α = 90 °.<br>b = 22.8424(11) Å, β = 97.959(2) °.<br>c = 10.9963(6) Å, γ = 90 °.
Volume	2014.65(18) $Å^3$
Z	4
Density (calculated)	1.307 Mg/m^3
Absorption coefficient	0.186 mm^{-1}
F(000)	840
Crystal size	0.180 x 0.150 x 0.110 mm^3
Theta range for data collection	2.584 to 25.992 °.
Index ranges	-8<=h<=9, -26<=k<=28, -13<=l<=13
Reflections collected	9683
Independent reflections	3883 [R(int) = 0.0468]
Completeness to theta = 25.242 °	98.1 %
Absorption correction	Semi-empirical from equivalents
Max. and min. transmission	0.7456 and 0.5500
Refinement method	Full-matrix least-squares on F^2
Data / restraints / parameters	3883 / 0 / 256
Goodness-of-fit on F^2	1.090
Final R indices [I>2sigma(I)]	R1 = 0.0566, wR2 = 0.1580
R indices (all data)	R1 = 0.0618, wR2 = 0.1626
Extinction coefficient	n/a
Largest diff. peak and hole	0.579 and -0.475 e.$Å^{-3}$

Compound 7s (CCDC 1967783)

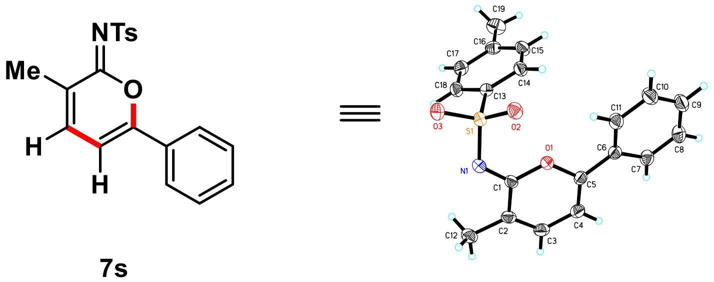

7s

Empirical formula	$C_{19}H_{17}NO_3S$
Formula weight	339.39
Temperature	293(2) K
Wavelength	0.71073 Å
Crystal system	Monoclinic
Space group	P 21/n
Unit cell dimensions	a = 13.6589(5) Å, α = 90 °.<br>b = 8.1701(3) Å, β = 104.6090(10) °.<br>c = 15.4766(6) Å, γ = 90 °.
Volume	1671.27(11) $Å^3$
Z	4
Density (calculated)	1.349 Mg/m^3
Absorption coefficient	0.210 mm^{-1}
F(000)	712
Crystal size	0.200 x 0.170 x 0.120 mm^3
Theta range for data collection	2.840 to 25.994 °.
Index ranges	-16<=h<=16, -10<=k<=10, -19<=l<=18
Reflections collected	16418
Independent reflections	3264 [R(int) = 0.0319]
Completeness to theta = 25.242 °	99.4 %
Absorption correction	Semi-empirical from equivalents
Max. and min. transmission	0.7456 and 0.6345
Refinement method	Full-matrix least-squares on F^2
Data / restraints / parameters	3264 / 0 / 219
Goodness-of-fit on F^2	1.034
Final R indices [I>2sigma(I)]	R1 = 0.0359, wR2 = 0.0942
R indices (all data)	R1 = 0.0428, wR2 = 0.1001
Extinction coefficient	n/a
Largest diff. peak and hole	0.251 and -0.306 e.$Å^{-3}$

Compound 10 (CCDC 1967780)

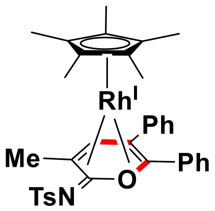

8a

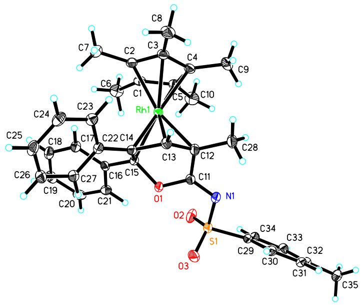

Empirical formula	$C_{35}H_{36}NO_3RhS$
Formula weight	653.62
Temperature	193(2) K
Wavelength	0.71073 Å
Crystal system	Monoclinic
Space group	C 2/c
Unit cell dimensions	a = 22.6277(6) Å, a= 90 °.<br>b = 15.9256(4) Å, b= 118.5590(10) °.<br>c = 19.0532(9) Å, g = 90 °.
Volume	6030.6(4) $Å^3$
Z	8
Density (calculated)	1.440 Mg/m^3
Absorption coefficient	0.672 mm^{-1}
F(000)	2704
Crystal size	0.150 x 0.130 x 0.100 mm^3
Theta range for data collection	2.558 to 25.999 °.
Index ranges	-27<=h<=26, -19<=k<=19, -23<=l<=23
Reflections collected	30502
Independent reflections	5899 [R(int) = 0.0299]
Completeness to theta = 25.242 °	99.7 %
Absorption correction	Semi-empirical from equivalents
Max. and min. transmission	0.7456 and 0.6414
Refinement method	Full-matrix least-squares on F^2
Data / restraints / parameters	5899 / 0 / 377
Goodness-of-fit on F^2	1.078

Final R indices [I>2sigma(I)]	R1 = 0.0242, wR2 = 0.0551
R indices (all data)	R1 = 0.0295, wR2 = 0.0580
Extinction coefficient	n/a
Largest diff. peak and hole	0.288 and -0.270 e.$Å^{-3}$

Compound 11 (CCDC 1967778)

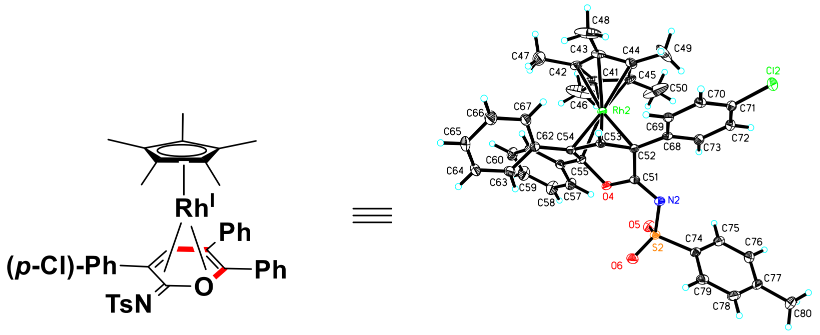

8b

Empirical formula	$C_{40}H_{37}ClNO_3RhS$
Formula weight	750.16
Temperature	192(2) K
Wavelength	0.71073 Å
Crystal system	Triclinic
Space group	P -1
Unit cell dimensions	a = 12.3006(4) Å, a= 77.8540(10) °.<br>b = 12.6515(4) Å, b= 85.6210(10) °.<br>c = 24.5054(8) Å, g = 85.0170(10) °..
Volume	3707.5(2) $Å^3$
Z	2
Density (calculated)	1.423 Mg/m^3
Absorption coefficient	0.632 mm^{-1}
F(000)	1640
Crystal size	0.170 x 0.150 x 0.120 mm^3
Theta range for data collection	2.310 to 26.000 °.
Index ranges	-15<=h<=15, -15<=k<=15, -29<=l<=30
Reflections collected	74435

Independent reflections	14546 [R(int) = 0.0467]
Completeness to theta = 25.242 °	99.8 %
Absorption correction	Semi-empirical from equivalents
Max. and min. transmission	0.7456 and 0.6559
Refinement method	Full-matrix least-squares on F^2
Data / restraints / parameters	14546 / 31 / 916
Goodness-of-fit on F^2	1.001
Final R indices [I>2sigma(I)]	R1 = 0.0301, wR2 = 0.0859
R indices (all data)	R1 = 0.0369, wR2 = 0.0927
Extinction coefficient	0.0016(3)
Largest diff. peak and hole	0.445 and -0.475 e.$Å^{-3}$

19. Computational Result

Computational Methods

All DFT calculations were carried out with Gaussian 09 program.[27] The geometry optimizations were conducted using B3LYP functional[28,29] including Grimme's D3 dispersion corrections[30] with Becke-Johnson damping, LANL2DZ basis set[31-33] for cobalt and 6-31G(d) basis set for other atoms. To confirm whether each optimized stationary point is an energy minimum or a transition state as well as evaluate the zero-point vibrational energy and thermal corrections at 298 K, the vibrational frequencies were computed at the same level of theory as for the geometry optimizations. On the basis of the gas-phase optimized structures, the single-point energies were evaluated with the B3LYP

functional def2-TZVP basis set[34,35], including Grimme's D3 (BJ-damping) dispersion corrections. The solvation energies were calculated using the self-consistent reaction field with the SMD implicit solvent model.[36] Fragment distortion and interaction energies were calculated at the B3LYP level of theory with def2-TZVPP basis set, including Grimme's D3 (BJ-damping) dispersion corrections without the inclusion of solvation energy corrections.

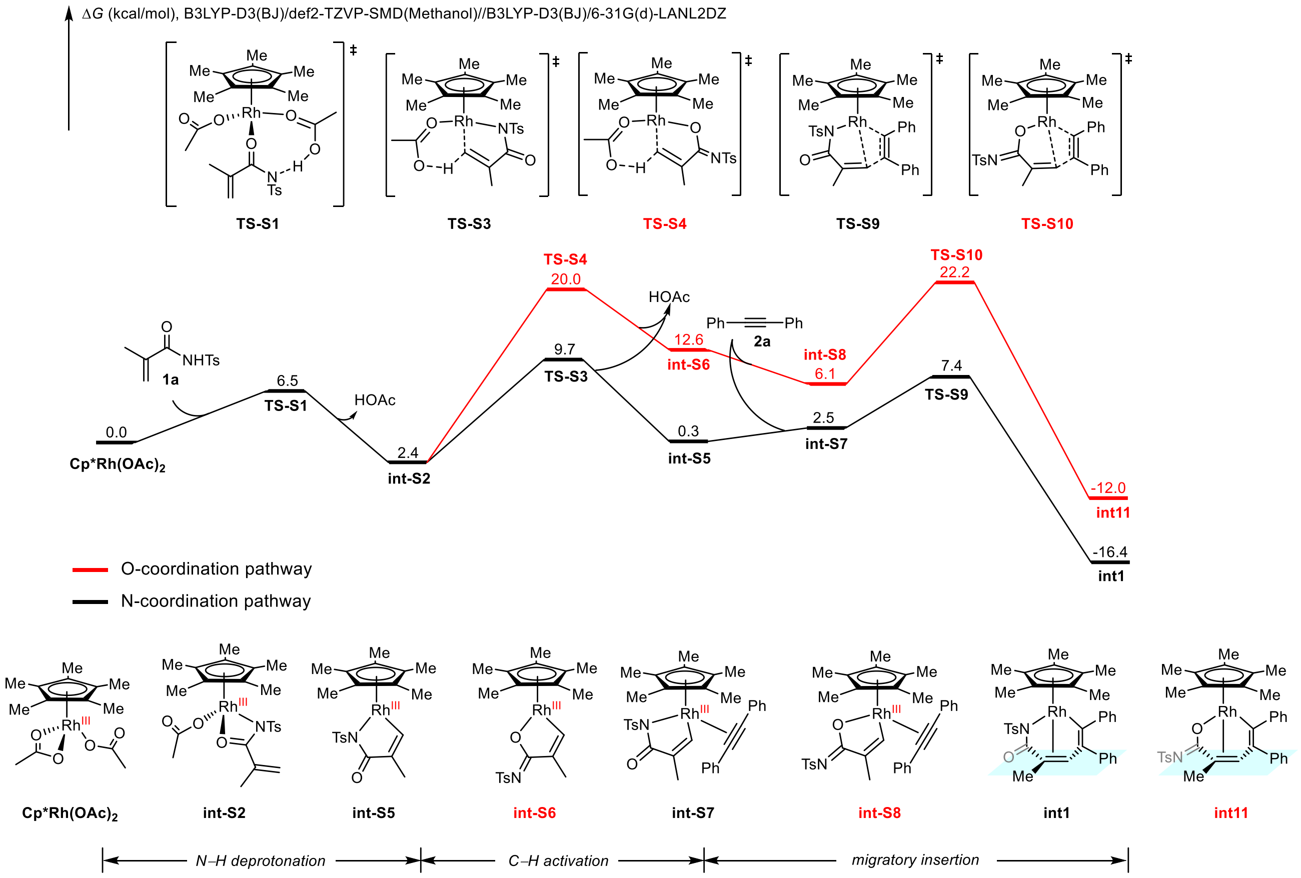

Supplementary Figure 14. **DFT-computed free energy changes of Rh-catalyzed vinylic C–H annulation when *N*-tosyl acrylamide 1a was employed as substrate.**

From the active catalyst Cp*Rh(OAc)$_2$, N–H deprotonation and vinyl C–H activation of *N*-tosyl acrylamide **1a** and subsequent diphenylacetylene insertion

generate the seven-membered ring vinyl-rhodium intermediate. The DFT-computed free energy changes of the seven-membered ring vinyl-rhodium intermediate formation are shown in Figure S14. The *N*-tosyl acrylamide **1a** first undergoes a N–H deprotonation via **TS-S1** to generate the intermediate **int-S2**. Subsequent vinyl C–H activation can occur through a CMD-type mechanism either with the N-coordination (**TS-S3**) or the O-coordination (**TS-S4**). The five-membered ring vinyl-rhodium species (**int-S5** and **int-S6**) undergo the corresponding alkyne insertion via **TS-S9** or **TS-S10** to produce the seven-membered ring vinyl-rhodium intermediate **int1** and **int11**. Comparing the two pathways, the N-coordination pathway (black pathway) is significantly more favorable than the O-coordination pathway (red pathway). This suggests that rhodium catalyst selectively generates the seven membered ring vinyl-rhodium species with N-coordination, and subsequent reductive elimination determines the chemoselectivity of product formation.

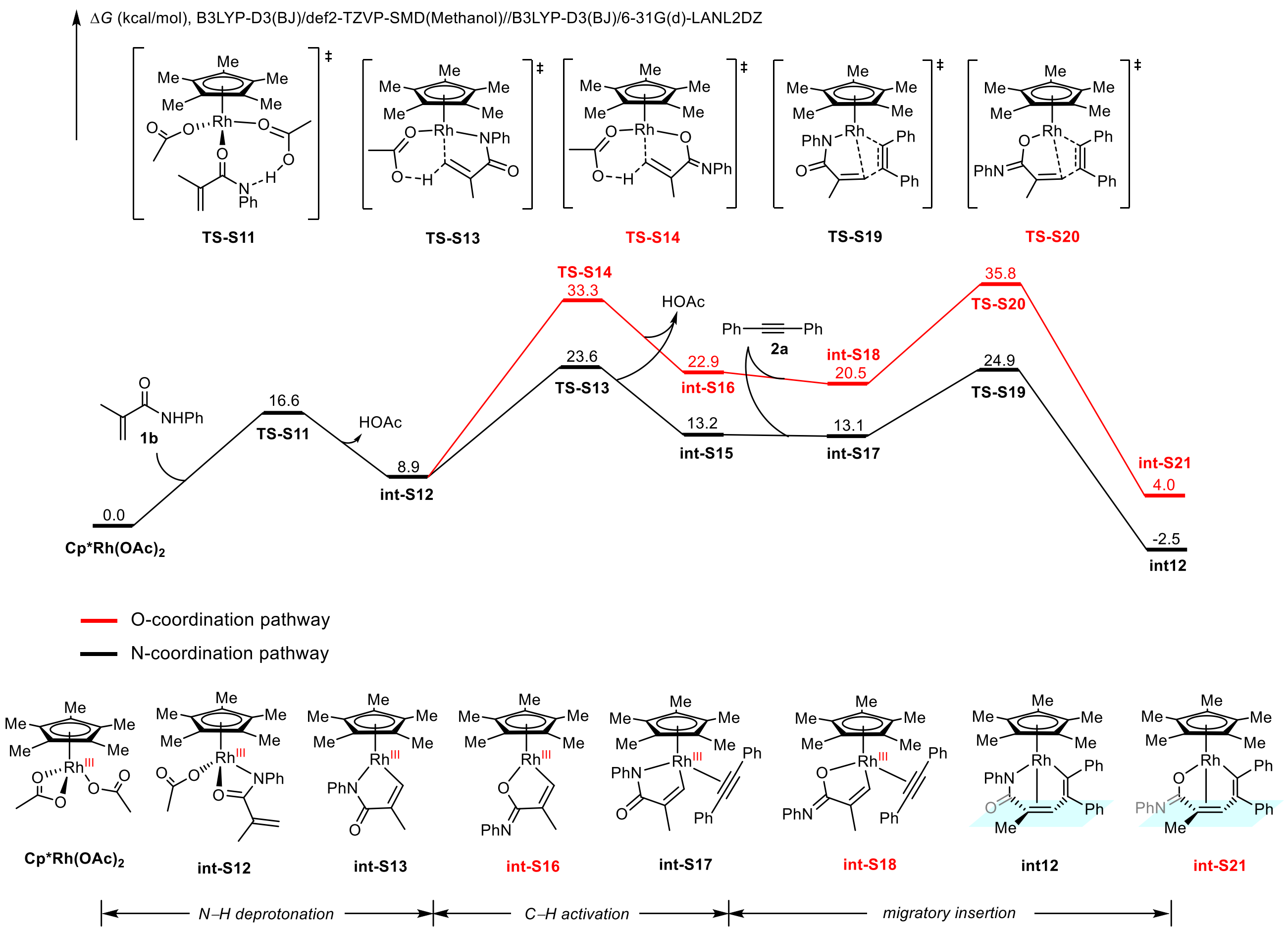

Supplementary Figure 15. DFT-computed free energy changes of Rh-catalyzed vinylic C–H annulation when _N_-phenyl acrylamide 1b was employed as substrate.

Similar to N-tosyl acrylamide, *N*-phenyl acrylamide **1b** undergoes the same mechanism to generate the seven-membered ring vinyl-rhodium intermediate **int12** with N-coordination or **int-S21** with O-coordination. The N-coordination pathway (black pathway) is more favorable than the O-coordination pathway (red pathway). Thus, the seven-membered ring vinyl-rhodium intermediate with N-coordination **int12** would be selectively generated under the rhodium catalysis. Subsequent reductive elimination of **int12** determines the

chemoselectivity of product formation.

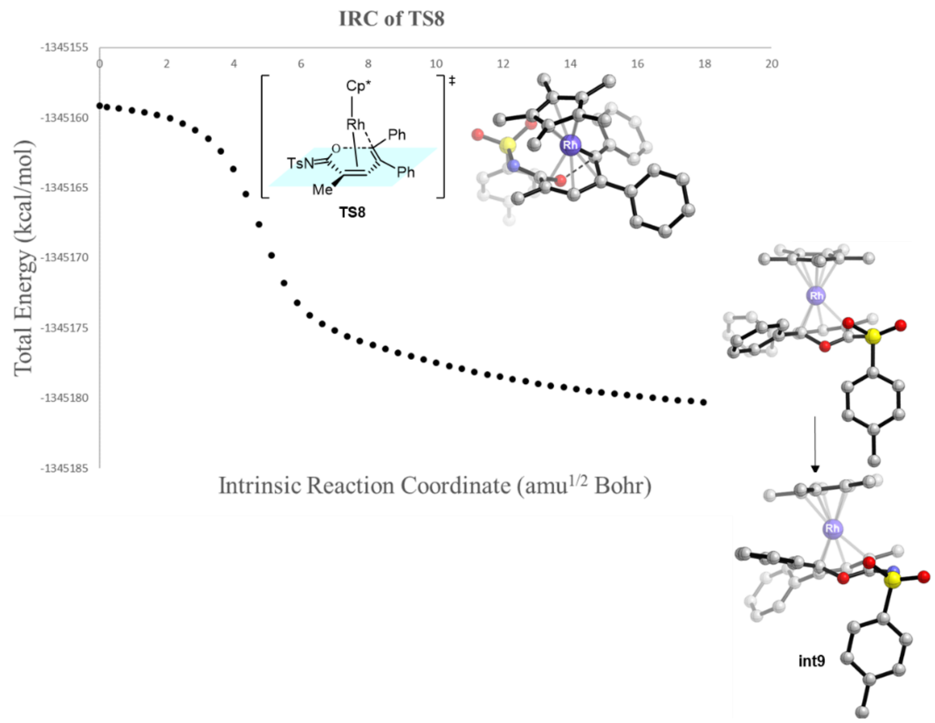

Supplementary Figure 16. **IRC of TS8.** IRC of **TS8** that confirms its connectivity to **int9**.

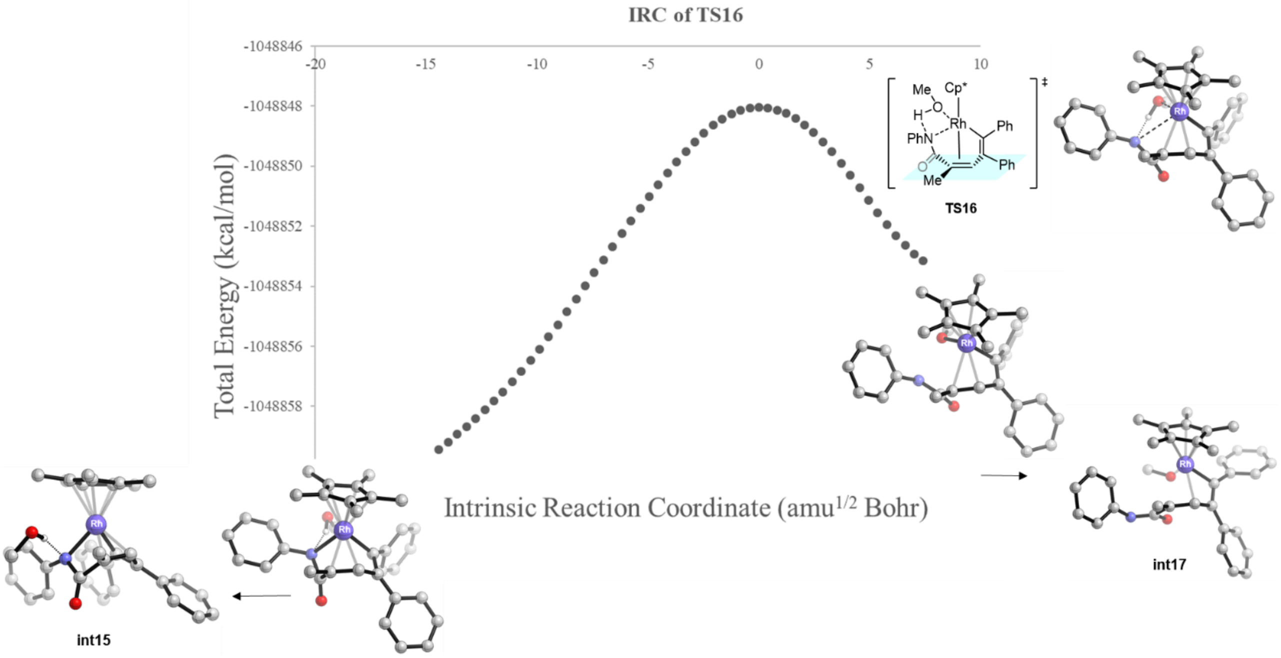

Supplementary Figure 17. **IRC of TS16.** IRC of **TS16** that confirms its

connectivity to **int15** and **int17**.

A. Ionic stepwise reductive elimination barriers

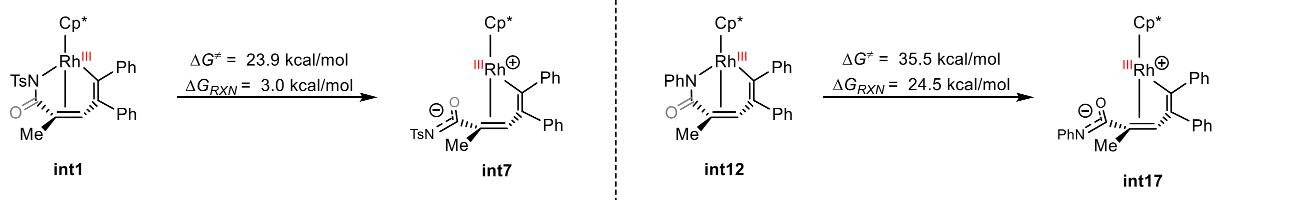

B. Heterolytic bond dissociation energy (HBDE) of Rh–N bond

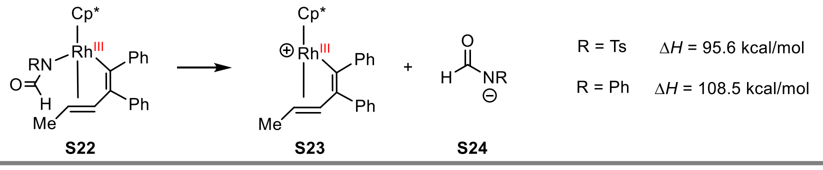

C. Hammett plot

R	HBDE (kcal/mol)	σ_p
Me	111.8	-0.88
Ph	108.5	1.34
p-OMe-C_6H_4	103.8	0.50
Ms	96.9	7.79
Ts	95.6	6.89
p-Cl-$C_6H_4SO_2$	92.2	8.29

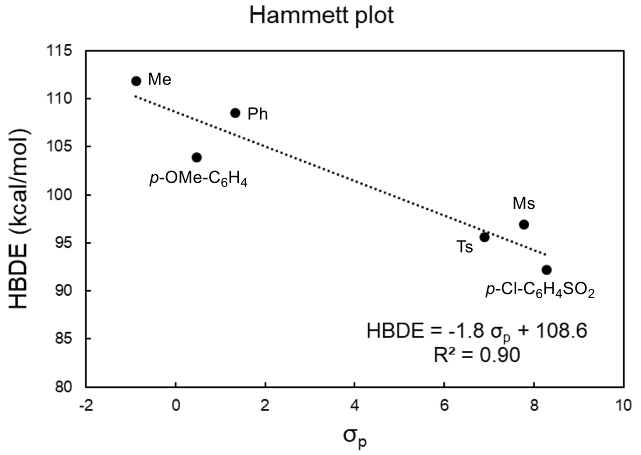

Supplementary Figure 18. **Analysis of the N-substituent effect on the ionic stepwise reductive elimination. A. Ionic stepwise reductive elimination barriers. B. Heterolytic bond dissociation energy (HBDE) of *Rh-N* bond. C. Hammett plot.**

For the ionic stepwise reductive elimination, the generation of the zwitterionic intermediate significantly favors the tosyl-substituted case. It requires 3.0 kcal/mol free energy to generate the tosyl-substituted zwitterionic intermediate **int7** from the seven-membered ring intermediate **int1**, while the same type of process requires 24.5 kcal/mol to generate the phenyl-substituted intermediate

int17 (Figure S18A). To verify the strength change of the rhodium-nitrogen bond, we computed the heterolytic bond dissociation energies on the model rhodium(III) complex **S22** (Figure S18B). The tosyl substitution stabilizes the dissociating amide anion, leading to a lower HBDE as expected (95.6 kcal/mol for tosyl vs. 108.5 kcal/mol for phenyl). To further corroborate the proposed electronic effect, the HBDEs of a series of substituted rhodium complexes were computed, and a linear relationship was identified with the corresponding Hammett constants (Figure S18C). These analyses provide additional supports for the proposed electronic origins of the *N*-substituent effect that electronic withdrawing substituent would promote the ionic stepwise reductive elimination pathway.

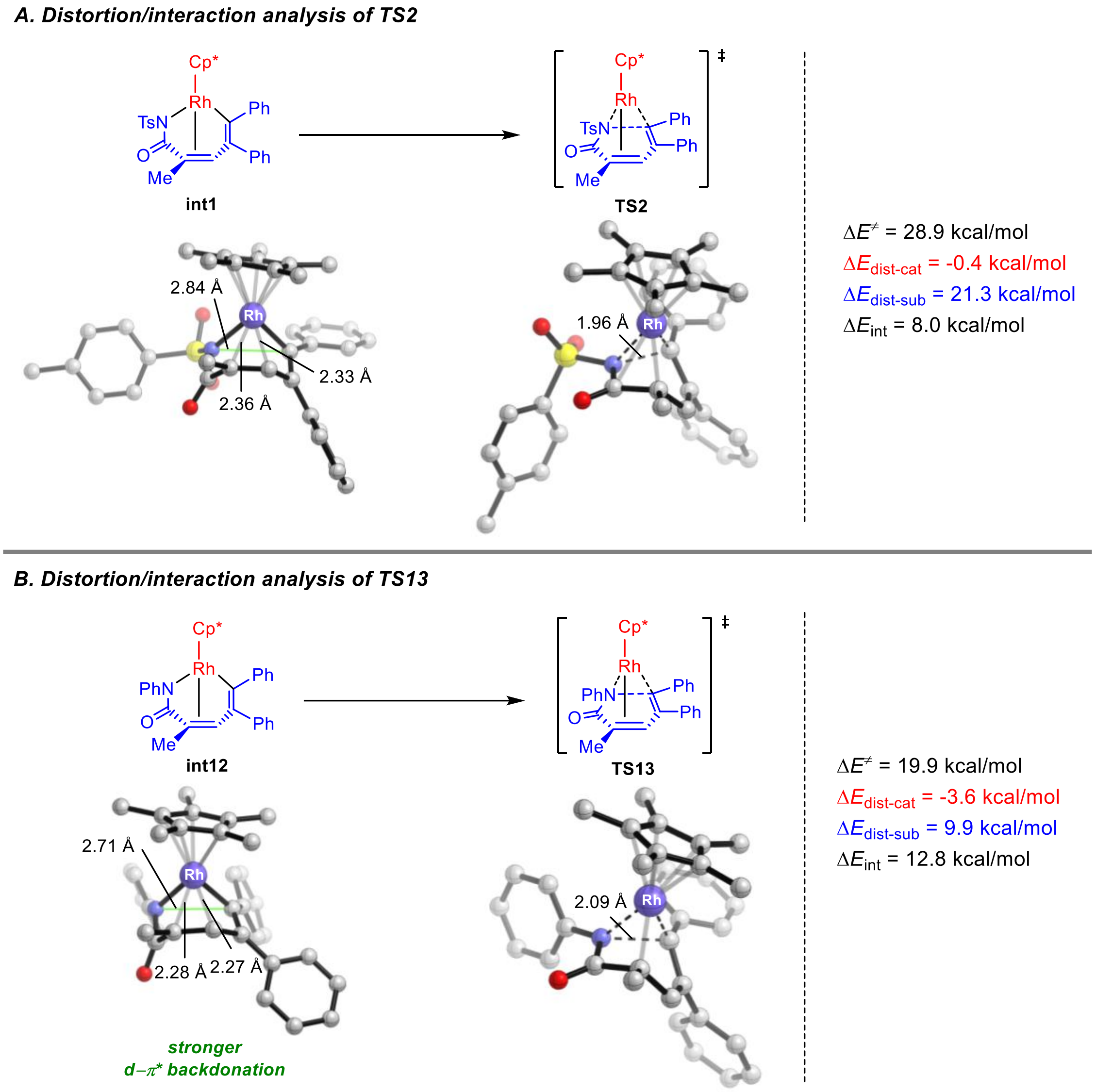

Supplementary Figure 19. **Analysis of the N-substituent effect on the neutral concerte reductive elimination. A. Distortion/interaction analysis of TS2. B. Distortion/interaction analysis of TS13.**

For the neutral concerted reductive elimination, the distortion/interaction analysis revealed the distortion-controlled origins of the N-substitution (Figure S19). The C–N reductive elimination transition states were separated to the rhodium catalyst part (labeled in red) and the amide substrate part (labeled in blue). The energy required to distort the selected fragment from the geometry in

the pre-intermediate to the corresponding geometry in the transition state is the distortion energy, $\Delta E_{\text{dist-cat}}$ and $\Delta E_{\text{dist-sub}}$. The difference between the electronic energy barrier ΔE and the total distortion energy ΔE_{dist} is the stabilizing interaction energy between the two distorted fragments in the transition state, $\Delta E_{\text{int}} = \Delta E - (\Delta E_{\text{dist}} + \Delta E_{\text{dist-sub}})$.[37,38]

Table of Energies

Table S1. Energies in Figure 4, Figure 5, Figure S1 and Figure S2. Zero-point correction (*ZPE*) thermal correction to enthalpy (*TCH*) thermal correction to Gibbs free energy (*TCG*) energies (*E*) enthalpies (*H*) and Gibbs free energy (*G*) (in Hartree) of the structures calculated at the B3LYP-D3(BJ)/def2TZVP-SMD(Methanol)//B3LYP-D3(BJ)/6-31G(d)-LANL2DZ level of theory.

Structures	*ZPE*	*TCH*	*TCG*	*E*	*H*	*G*	Imaginary Frequency
**$Cp^*Rh(OAc)_2$**	0.327676	0.352746	0.273207	−958.210671	−957.857925	−957.937464	
**1a**	0.226389	0.243925	0.179440	−1105.977167	−1105.733242	−1105.79773	
**1b**	0.189642	0.201671	0.151925	−517.922789	−517.721118	−517.770864	
**2a**	0.191989	0.204023	0.152886	−539.720496	−539.516473	−539.567610	
**int1**	0.625692	0.667983	0.550252	−2145.535962	−2144.867979	−2144.98571	
**TS2**	0.623919	0.665330	0.550563	−2145.485939	−2144.820609	−2144.93538	300.97*i*
**int3**	0.628175	0.669130	0.557289	−2145.565605	−2144.896475	−2145.00832	
**int4**	0.681073	0.726673	0.604073	−2261.329576	−2260.602903	−2260.725503	
**TS5**	0.680722	0.725752	0.604968	−2261.307328	−2260.581576	−2260.70236	82.03*i*
**int6**	0.679829	0.725242	0.603676	−2261.319668	−2260.594426	−2260.715992	
**int7**	0.625494	0.667614	0.551456	−2145.527664	−2144.860324	−2144.98100	
**TS8**	0.625560	0.666717	0.551884	−2145.519849	−2144.853132	−2144.967965	28.94*i*

**int9**	0.627667	0.668822	0.554508	−2145.545228	−2144.876406	−2144.99072	
**TS10**	0.624056	0.666017	0.547191	−2145.504267	−2144.83825	−2144.957076	95.04*i*
**int11**	0.625780	0.667751	0.551918	−2145.530535	−2144.862784	−2144.978617	
**int12**	0.587076	0.624328	0.519433	−1557.456009	−1556.831681	−1556.936576	
**TS13**	0.586132	0.622441	0.519353	−1557.421046	−1556.798605	−1556.901693	207.13*i*
**int14**	0.589794	0.625870	0.524544	−1557.491721	−1556.865851	−1556.967177	
**int15**	0.642723	0.683610	0.571151	−1673.252145	−1672.568535	−1672.568753	
**TS16**	0.640746	0.680879	0.570877	−1673.205945	−1672.525066	−1672.635068	107.67*i*
**int17**	0.640263	0.680819	0.569120	−1673.221355	−1672.540536	−1672.652235	
**int18**	0.588013	0.624520	0.522882	−1557.416024	−1556.791504	−1556.893142	
**TS-S1**	0.551625	0.593925	0.475437	−2064.19729	−2063.603365	−2063.721853	1013.48*i*
**int-S2**	0.492885	0.529564	0.425205	−1834.990551	−1834.460987	−1834.565346	
**TS-S3**	0.487659	0.523711	0.420697	−1834.968736	−1834.445025	−1834.548039	897.63*i*
**TS-S4**	0.487254	0.523393	0.417958	−1834.94959	−1834.426197	−1834.531632	894.28*i*
**int-S5**	0.428352	0.459751	0.363992	−1605.758435	−1605.298684	−1605.394443	
**int-S6**	0.428598	0.459546	0.366216	−1605.741091	−1605.281545	−1605.374875	
**int-S7**	0.623917	0.666818	0.548860	−2145.504443	−2144.837625	−2144.955583	
**int-S8**	0.623120	0.666262	0.544801	−2145.494673	−2144.828411	−2144.94987	
**TS-S9**	0.622657	0.665131	0.546970	−2145.494673	−2144.829542	−2144.94770	397.48*i*
**TS-S10**	0.622469	0.664945	0.544304	−2145.468393	−2144.803448	−2144.92409	411.11*i*

**TS-S11**	0.513169	0.550416	0.442937	−1476.12174	−1475.571324	−1475.678803	905.54*i*
**int-S12**	0.454598	0.486426	0.392271	−1246.91481	−1246.428384	−1246.522539	
**TS-S13**	0.449366	0.480313	0.389183	−1246.888247	−1246.407934	−1246.499064	967.85*i*
**TS-S14**	0.448872	0.479911	0.386503	−1246.870139	−1246.390228	−1246.483636	897.79*i*
**int-S15**	0.390304	0.416311	0.335997	−1017.682955	−1017.266644	−1017.346958	
**int-S16**	0.390293	0.416397	0.333838	−1017.6654	−1017.249003	−1017.331562	
**int-S17**	0.585046	0.623149	0.515554	−1557.427274	−1556.804125	−1556.91172	
**int-S18**	0.584355	0.622655	0.512519	−1557.412426	−1556.789771	−1556.89991	
**TS-S19**	0.584314	0.621808	0.514750	−1557.407768	−1556.78596	−1556.893018	348.27*i*
**TS-S20**	0.583554	0.621234	0.511720	−1557.387277	−1556.766043	−1556.87556	407.83*i*
**int-S21**	0.587095	0.624274	0.516786	−1557.443031	−1556.818757	−1556.926245	
**MeOH**	0.051481	0.055716	0.028760	−115.786546	−115.73083	−115.757786	
**HOAc**	0.062046	0.067569	0.034724	−229.206367	−229.138798	−229.171643	

Table S2. Energies in Figure S5. Zero-point correction (*ZPE*) thermal correction to enthalpy (*TCH*) thermal correction to Gibbs free energy (*TCG*) energies (*E*) enthalpies (*H*) and Gibbs free energy (*G*) (in Hartree) of the structures calculated at the B3LYP-D3(BJ)/def2TZVP//B3LYP-D3(BJ)/6-31G(d)-LANL2DZ level of theory.

Structures	*ZPE*	*TCH*	*TCG*	*E*	*H*	*G*	Imaginary Frequency
**S22 (Me)**	0.555574	0.591147	0.490506	−1366.761716	−1366.170569	−1366.27121	
**S22 (Ph)**	0.608327	0.646968	0.538561	−1558.598178	−1557.95121	−1558.059617	
**S22 (*p*-OMe-C_6H_4)**	0.641199	0.682346	0.568297	−1673.173688	−1672.491342	−1672.605391	
**S22 (Ms)**	0.566078	0.604784	0.497346	−1915.510998	−1914.906214	−1915.013652	

**S22 (Ts)**	0.646764	0.690468	0.571364	−1558.598178	−1557.95121	−1558.059617
**S22 (*p*-Cl-$C_6H_4SO_2$)**	0.609537	0.652573	0.534137	−2566.959958	−2566.307385	−2566.425821
**S23**	0.491983	0.522080	0.432813	−1157.871219	−1157.349139	−1157.438406
**S24 (Me)**	0.060179	0.065556	0.033454	−208.70876	−208.643204	−208.675306
**S24 (Ph)**	0.113731	0.121756	0.081741	−400.550975	−400.429219	−400.469234
**S24 (*p*-OMe-C_6H_4)**	0.146049	0.156857	0.109796	−515.133576	−514.976719	−515.02378
**S24 (Ms)**	0.071489	0.079770	0.039401	−757.482461	−757.402691	−757.44306
**S24 (Ts)**	0.152245	0.165372	0.112161	-988.646715	-988.481343	-988.534554
**S24 (*p*-Cl-$C_6H_4SO_2$)**	0.114700	0.127429	0.074393	−1408.93873	−1408.811301	−1408.864337

20. Spectral Data

^{1}H NMR Spectrum of 2b at 25 ^{o}C ($CDCl_3$)

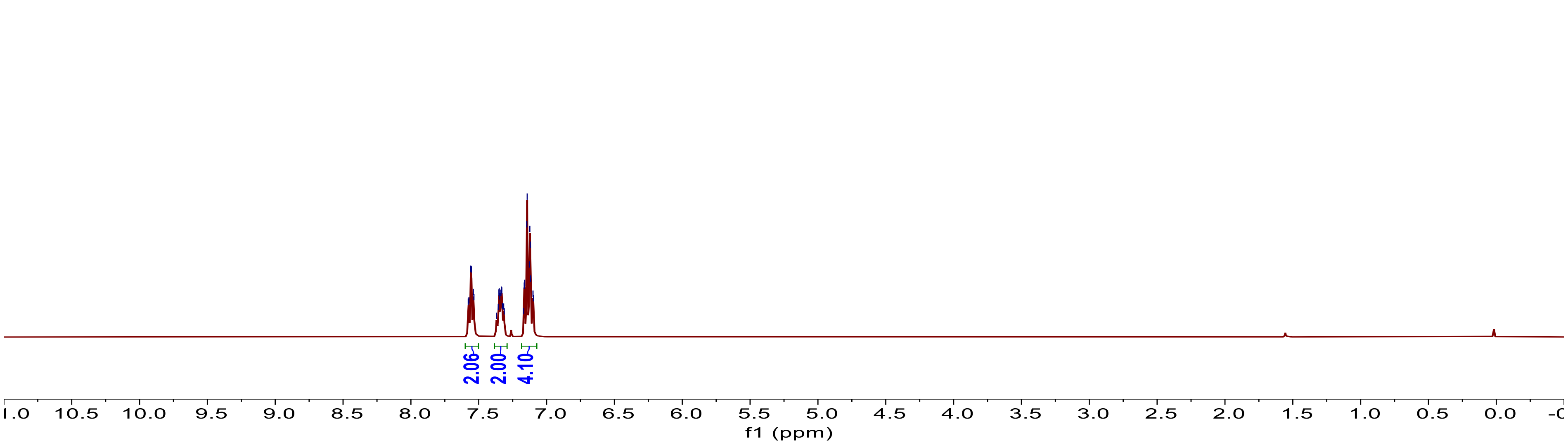

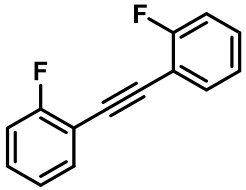

2b

^{1}H NMR Spectrum of 2c at 25 ºC ($CDCl_3$)

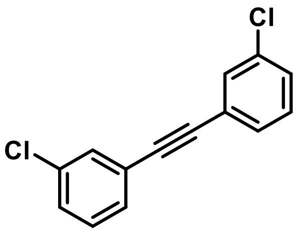

2c

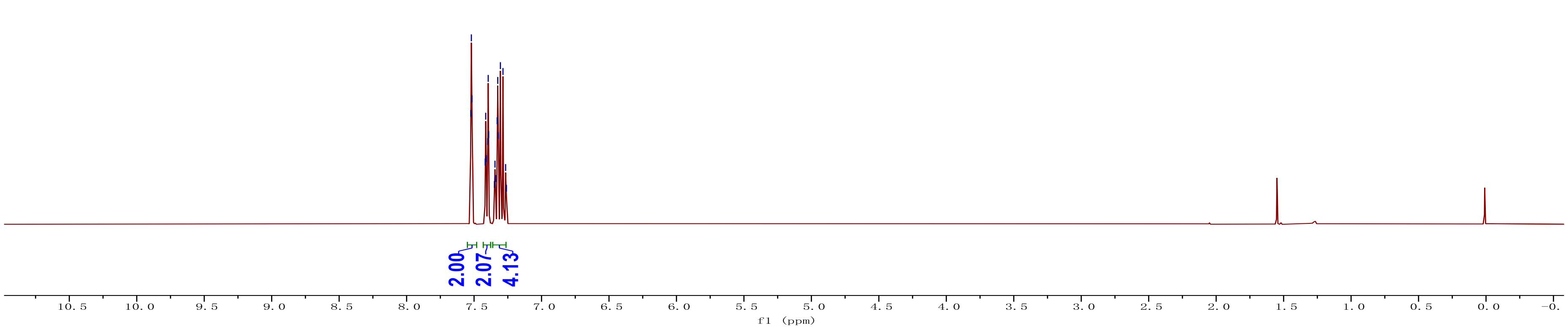

^{1}H NMR Spectrum of 2d at 25 ºC ($CDCl_3$)

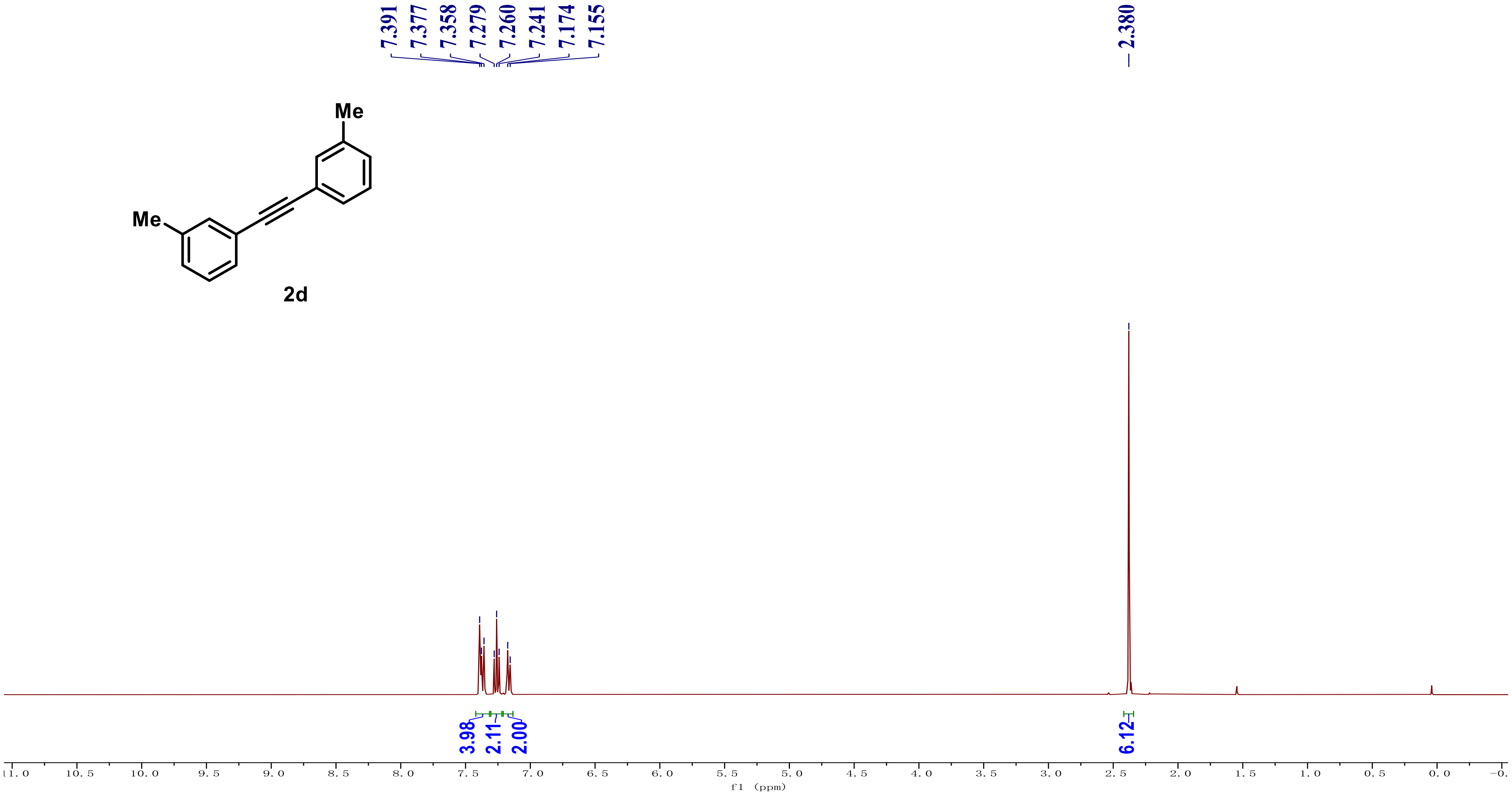

2d

^{1}H NMR Spectrum of 2e at 25 ºC ($CDCl_3$)

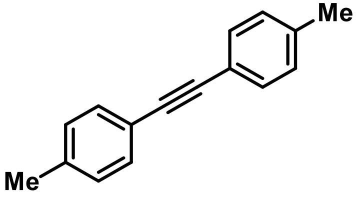

2e

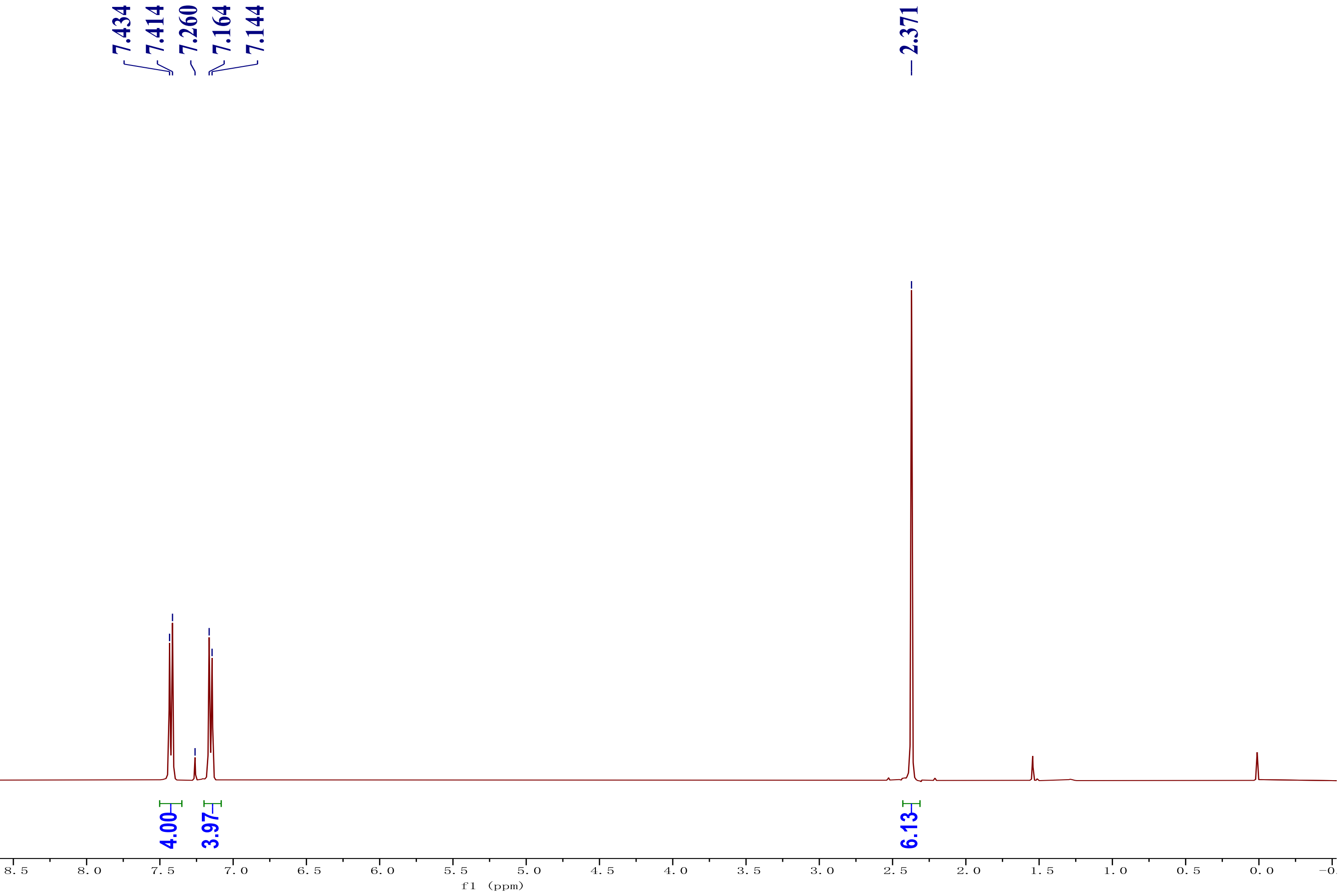

^{1}H NMR Spectrum of 2f at 25 ºC ($CDCl_3$)

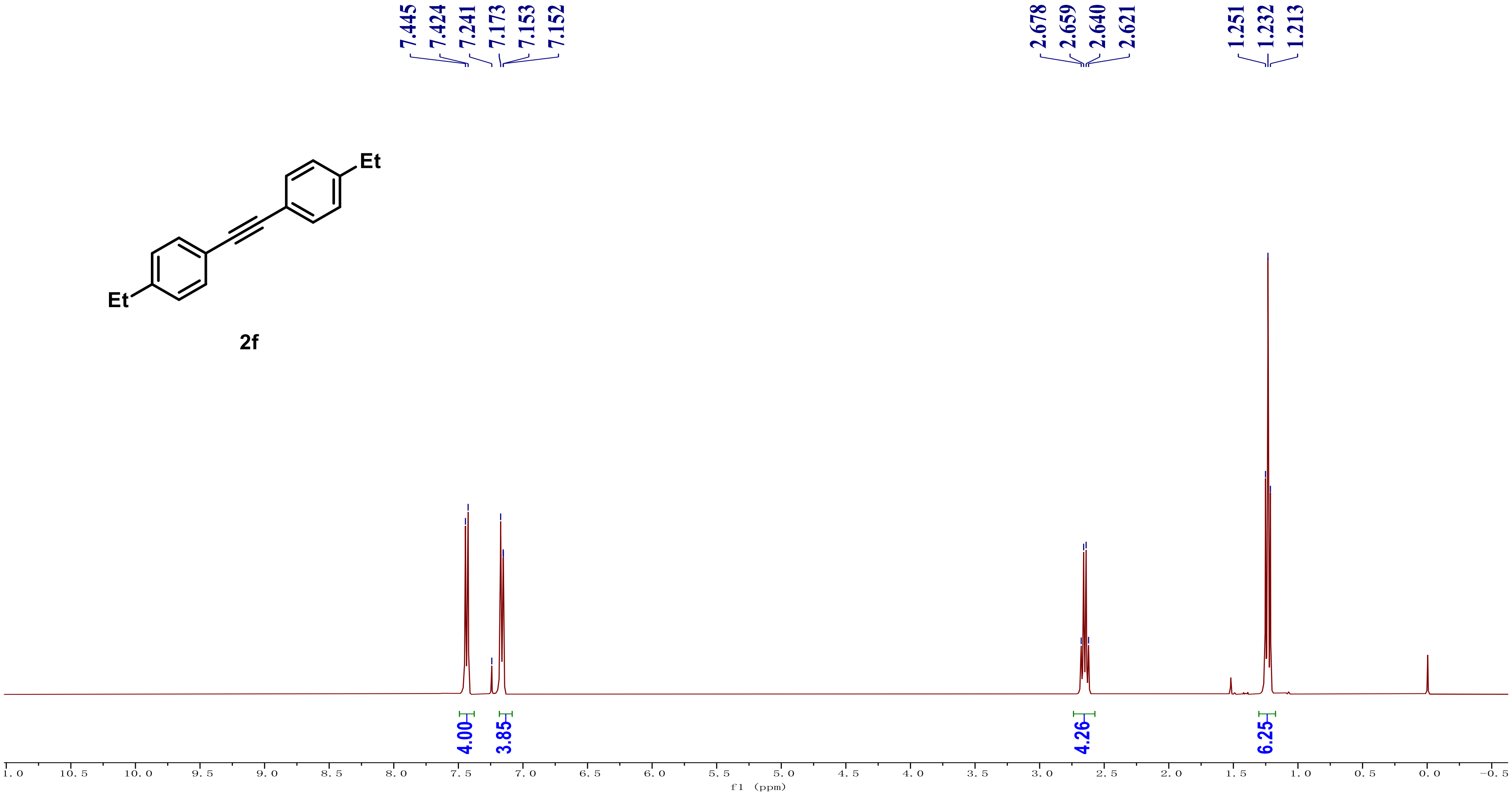

^{1}H NMR Spectrum of 2g at 25 ºC ($CDCl_3$)

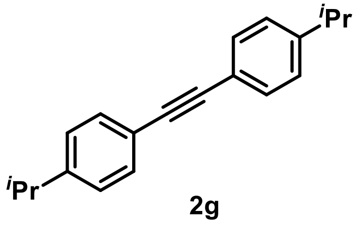

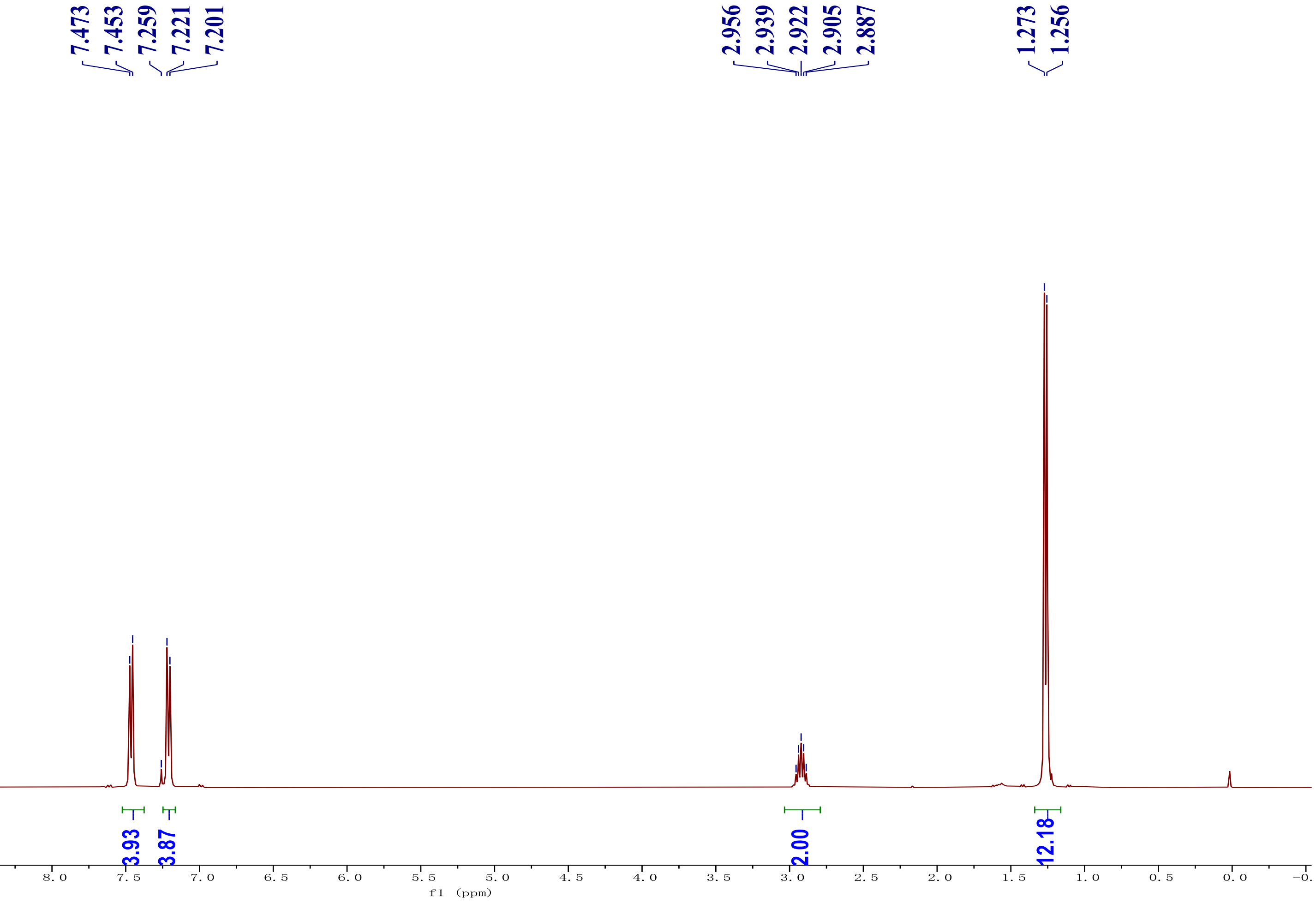

^{1}H NMR Spectrum of 2h at 25 °C ($CDCl_3$)

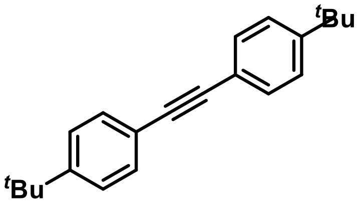

2h

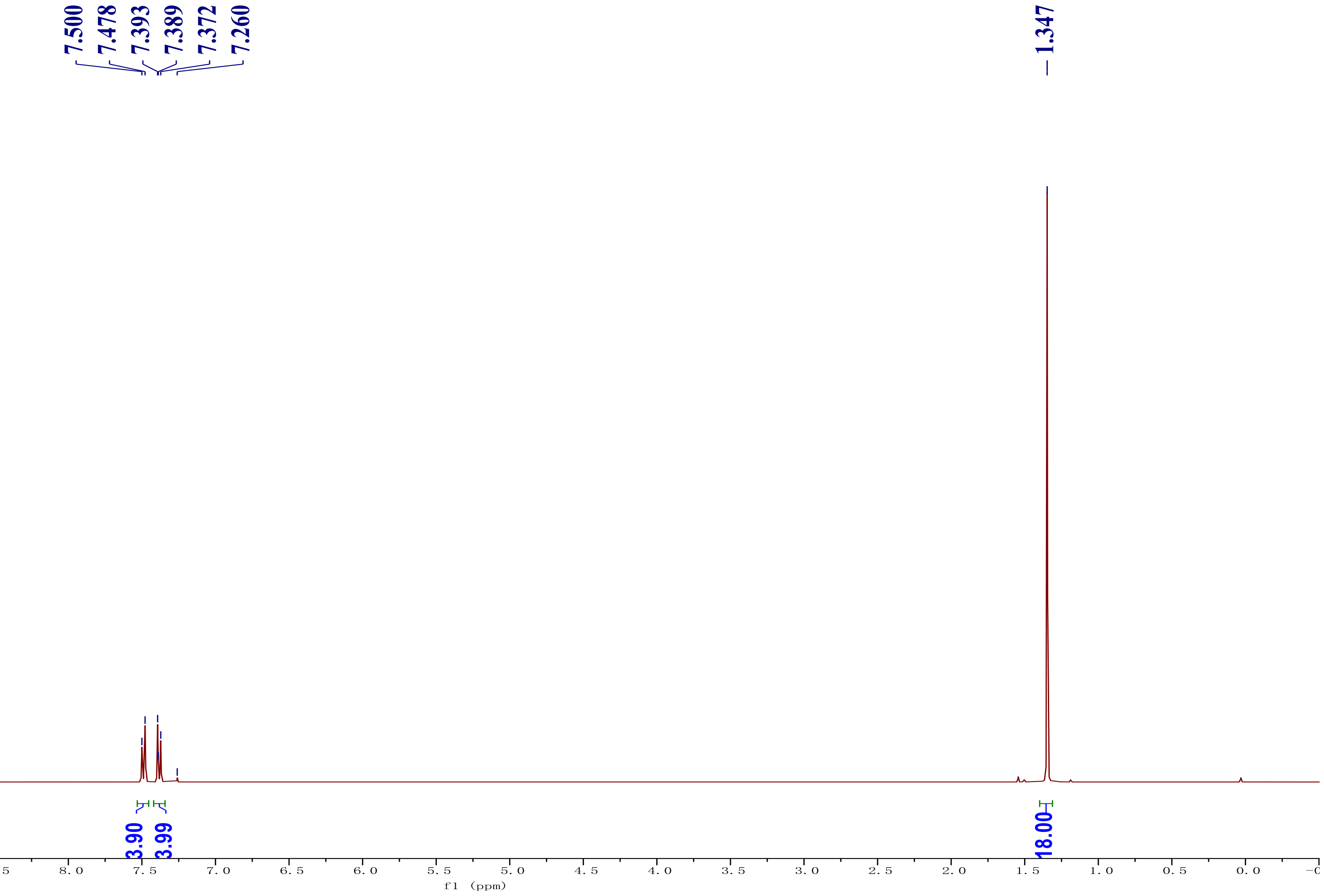

^{1}H NMR Spectrum of 2i at 25 ºC ($CDCl_3$)

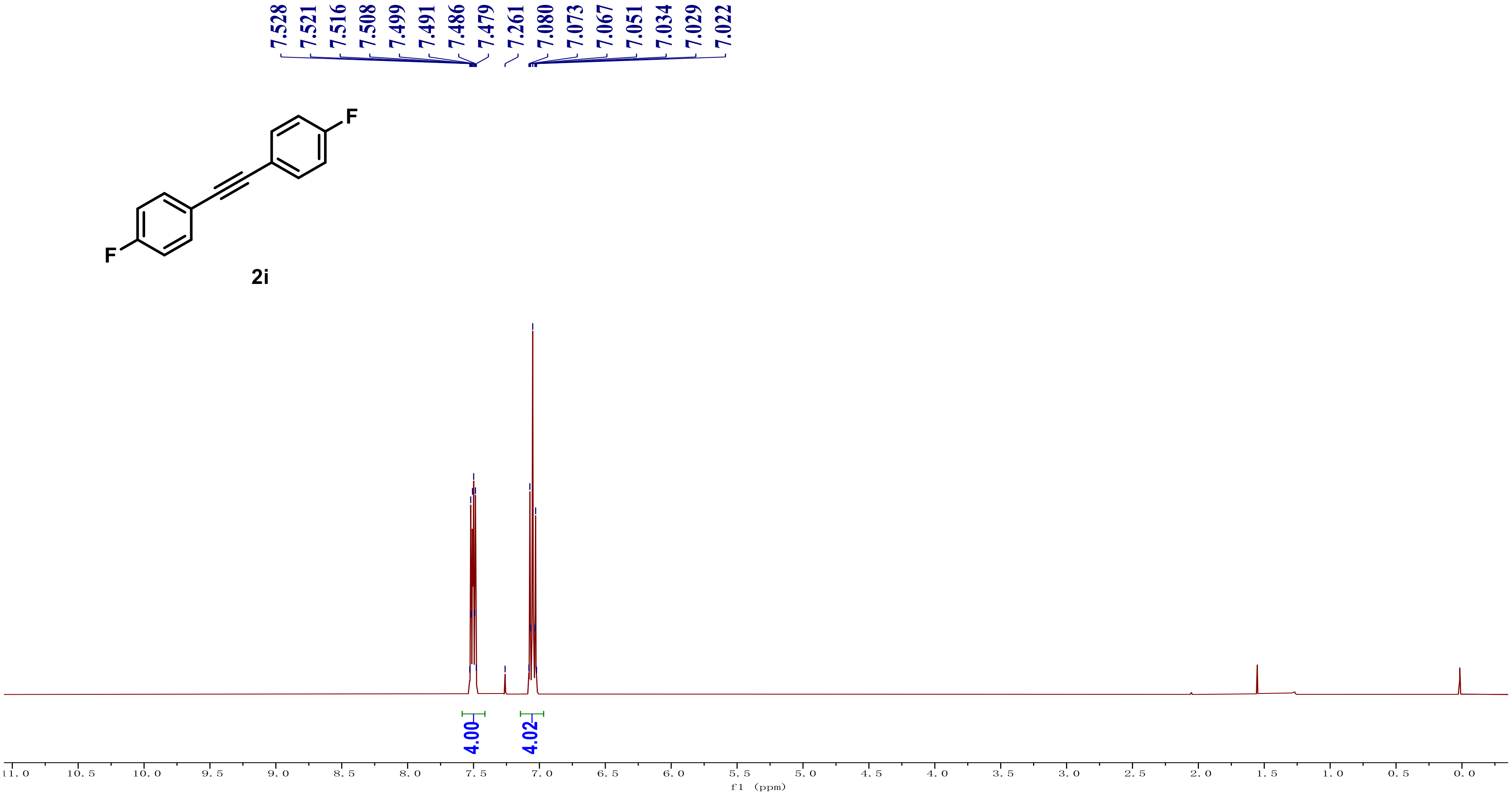

^{1}H NMR Spectrum of 2j at 25 ºC ($CDCl_3$)

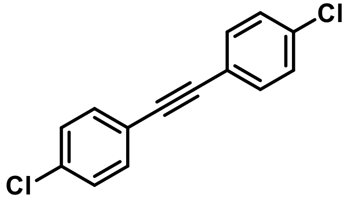

2j

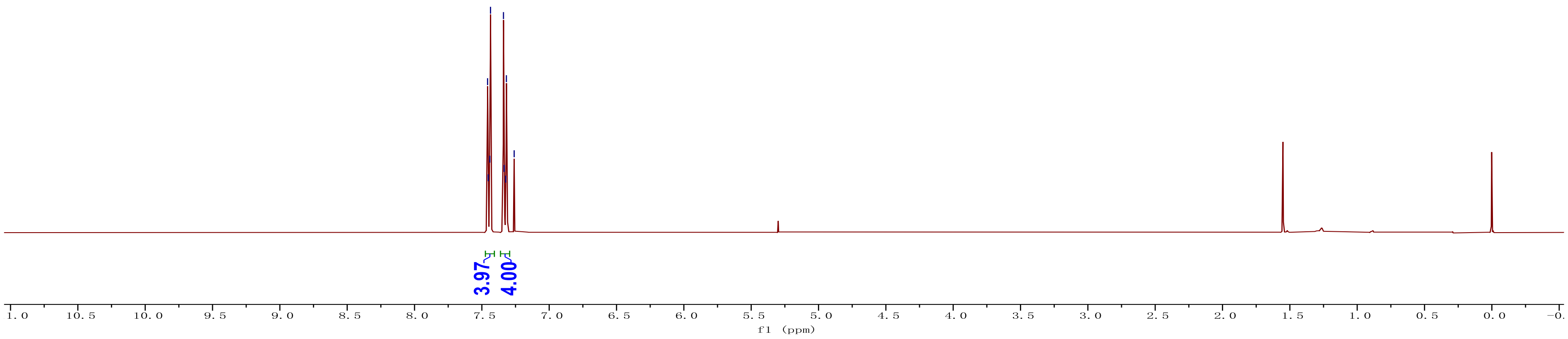

^{1}H NMR Spectrum of 2k at 25 °C ($CDCl_3$)

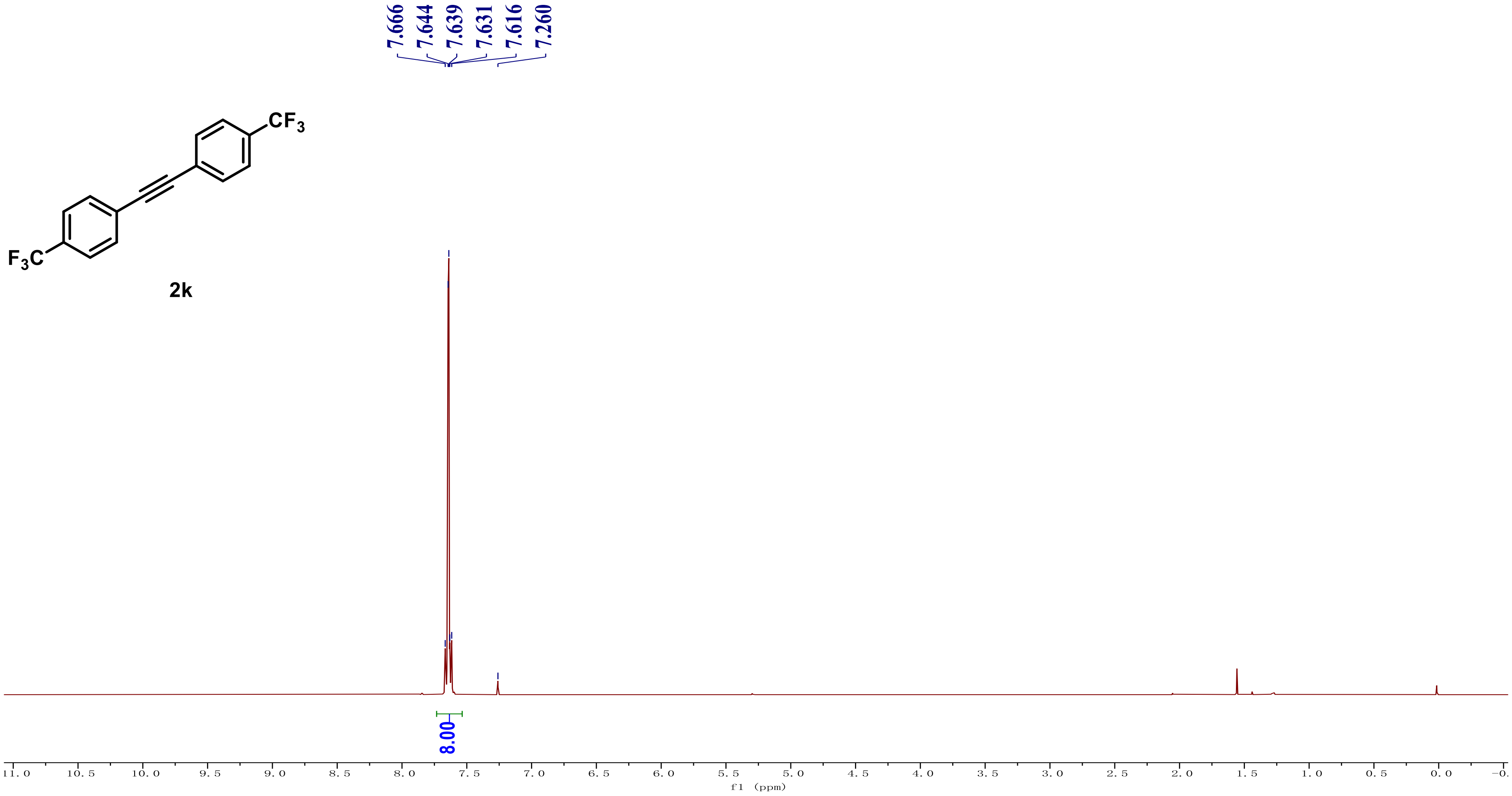

^{1}H NMR Spectrum of 2l at 25 ºC ($CDCl_3$)

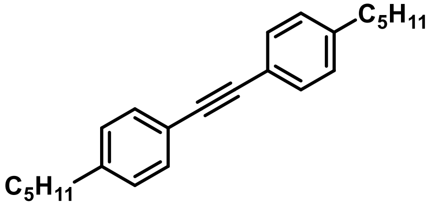

2l

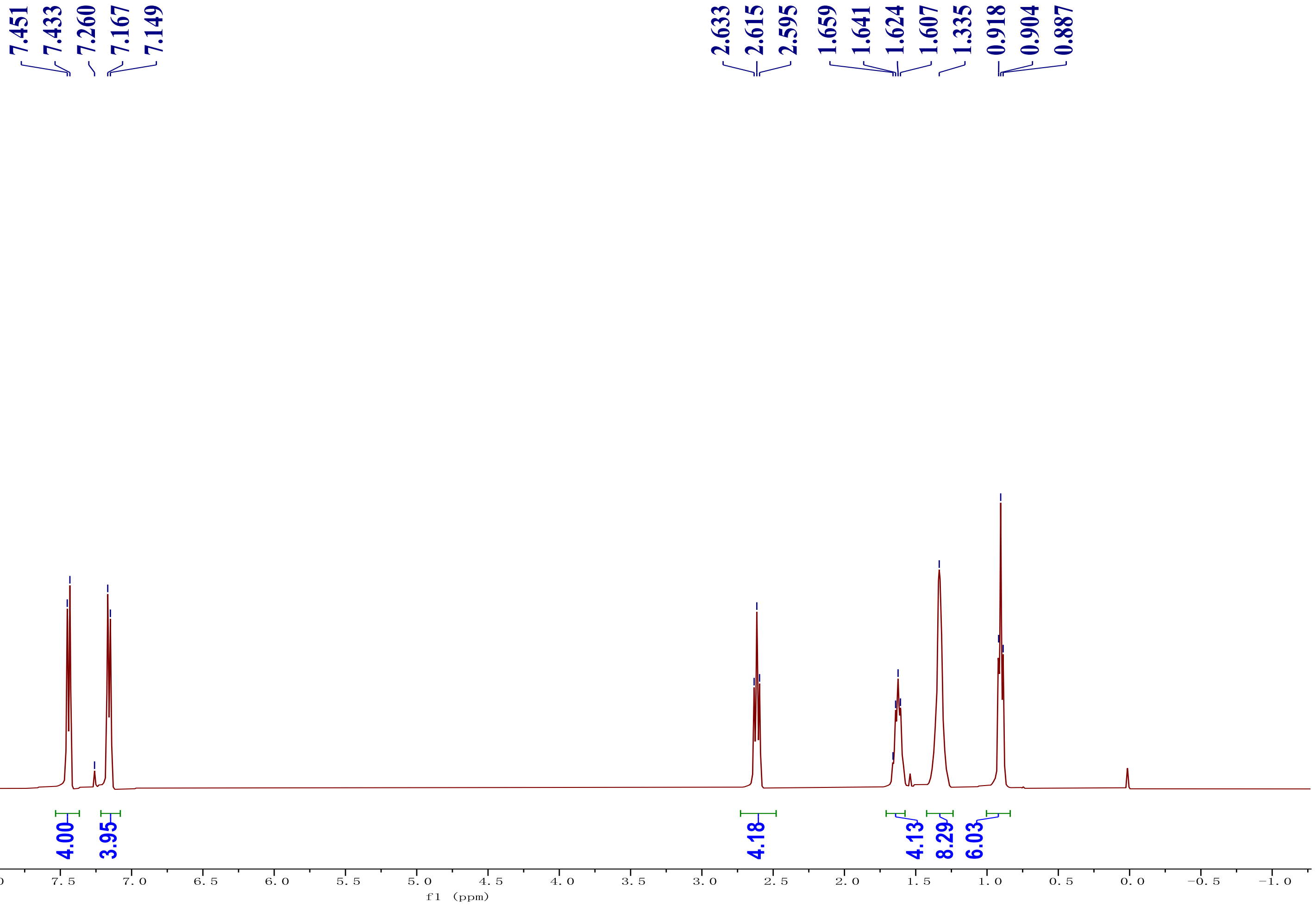

^{1}H NMR Spectrum of 2m at 25 °C ($CDCl_3$)

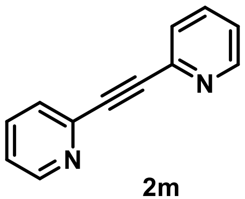

2m

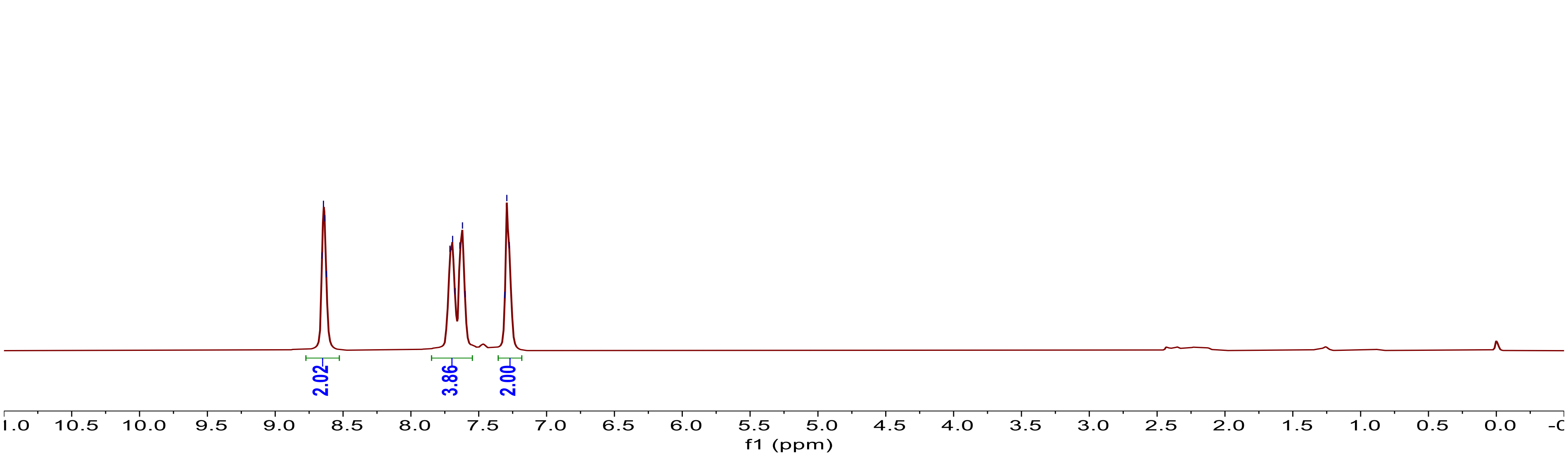

^{1}H NMR Spectrum of 2q at 25 ºC ($CDCl_3$)

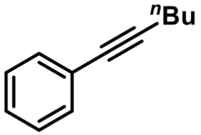

2q

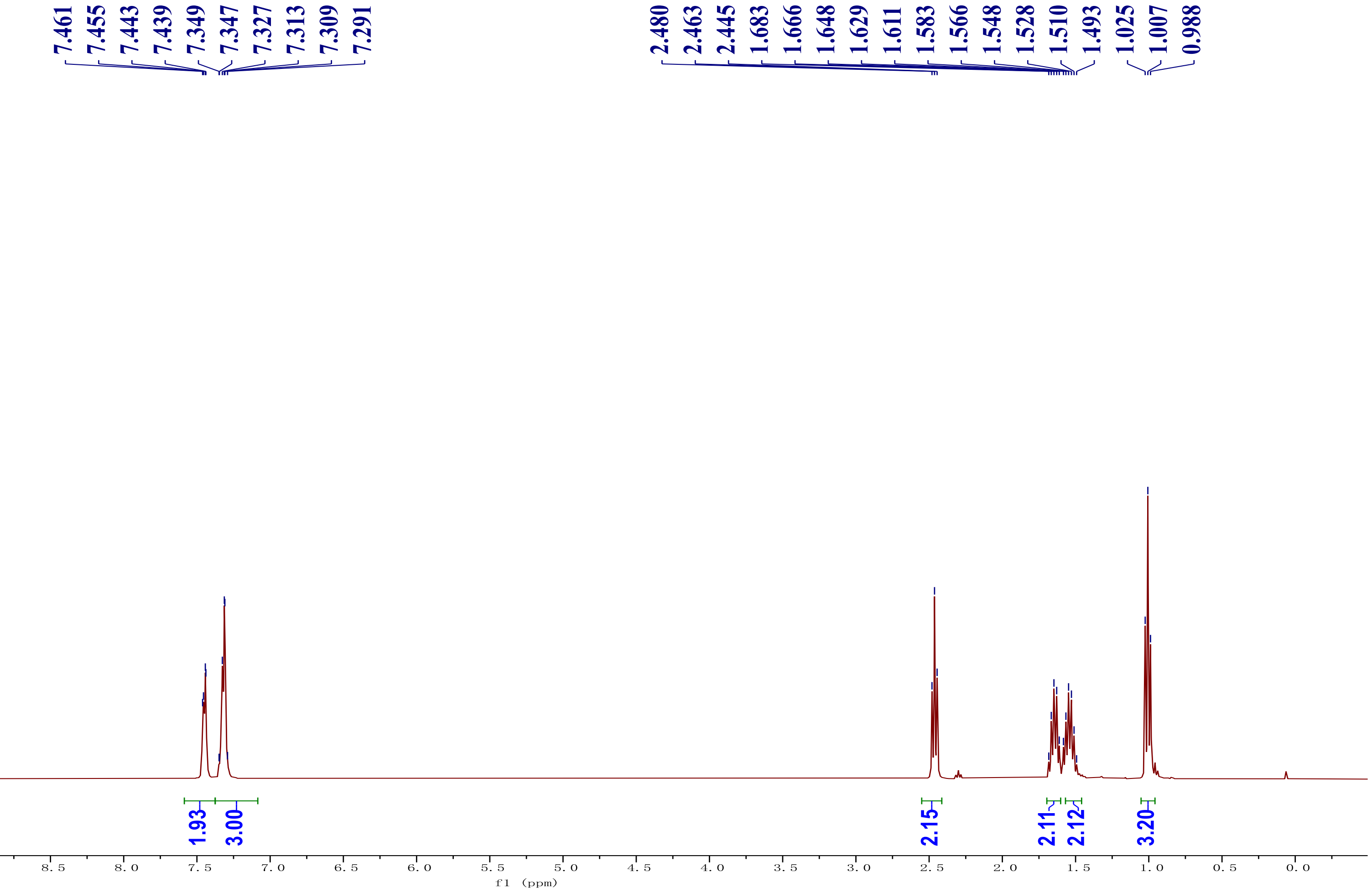

^{1}H NMR Spectrum of 2r at 25 °C ($CDCl_3$)

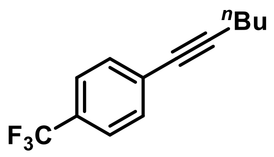

2r

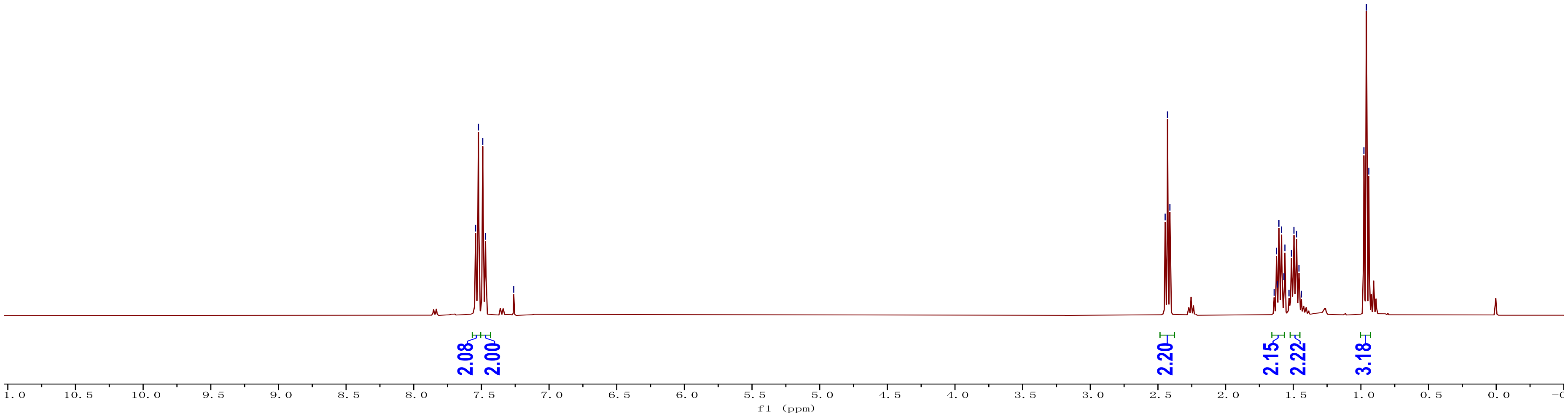

^{1}H NMR Spectrum of 2s at 25 °C ($CDCl_3$)

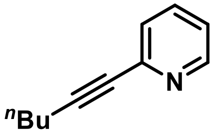

2s

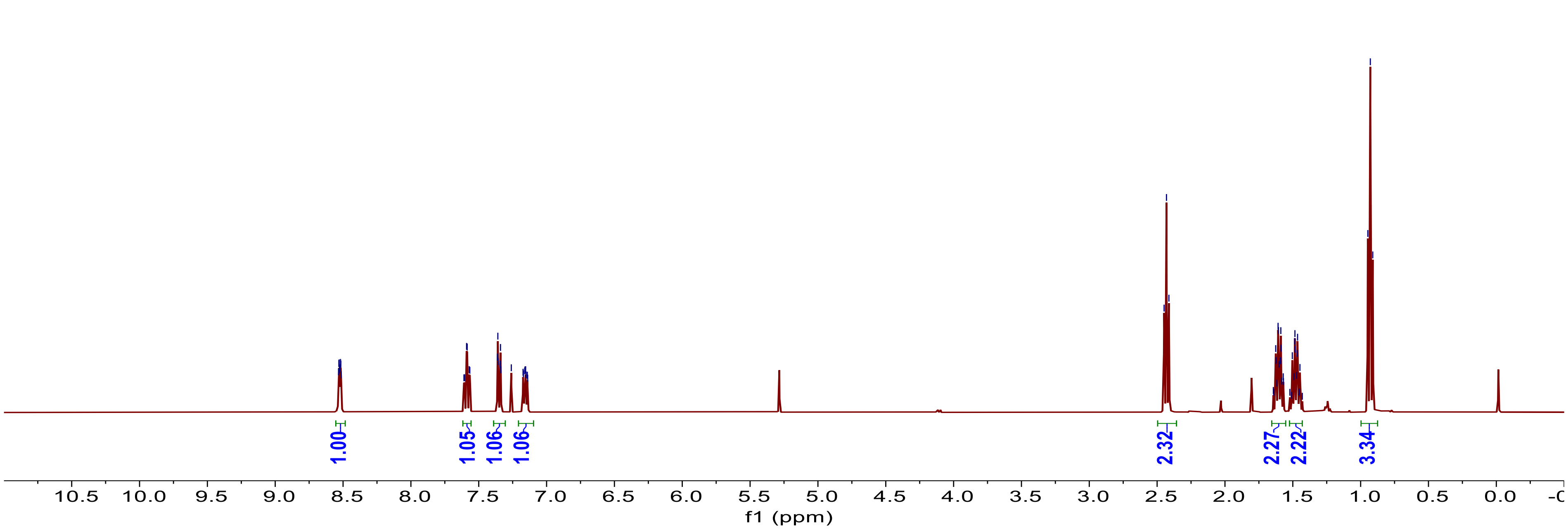

^{1}H NMR Spectrum of 2z at 25 °C ($CDCl_3$)

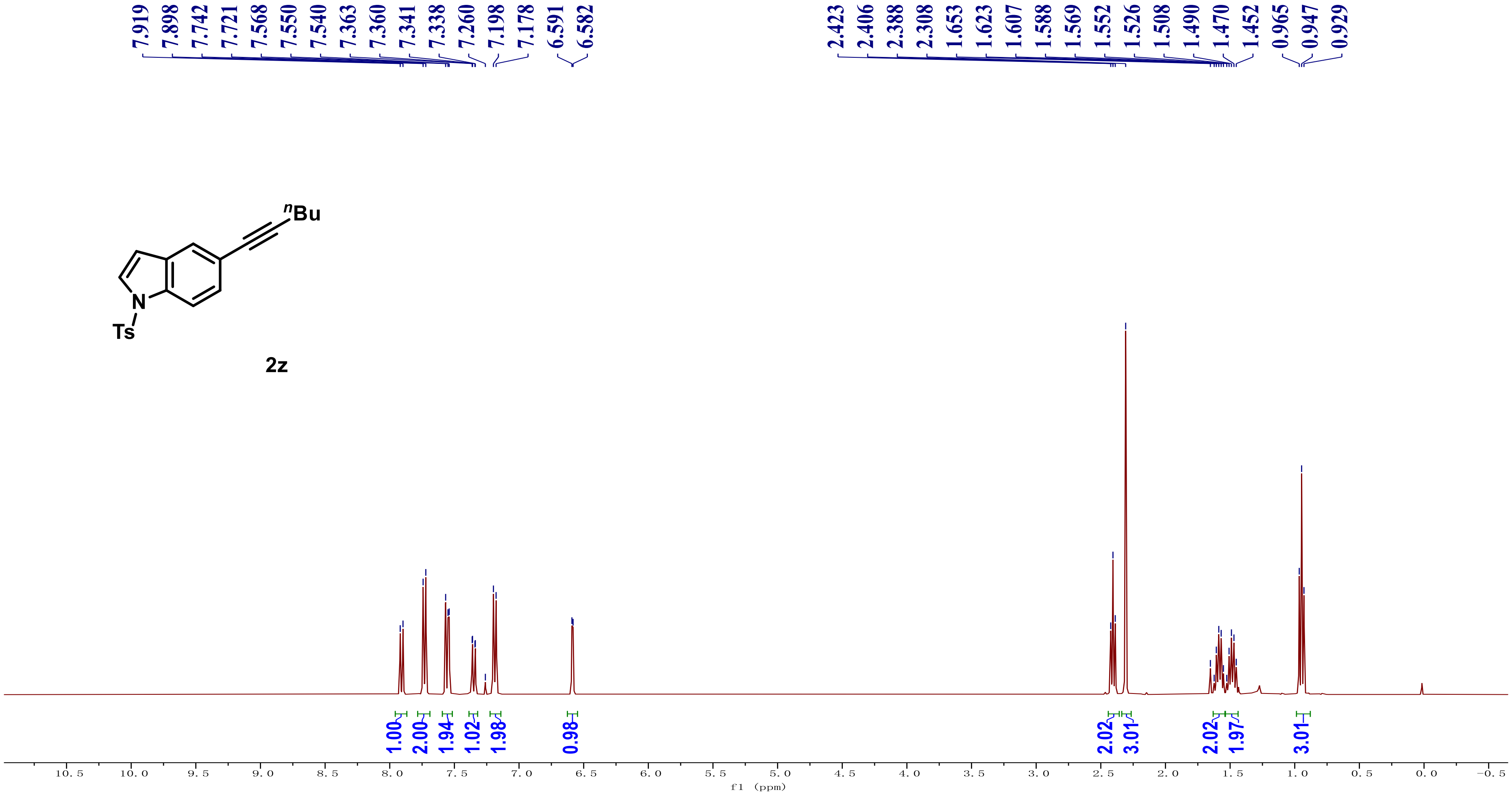

^{1}H NMR Spectrum of 1a at 25 °C ($CDCl_3$)

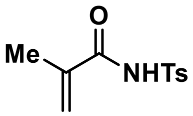

1a

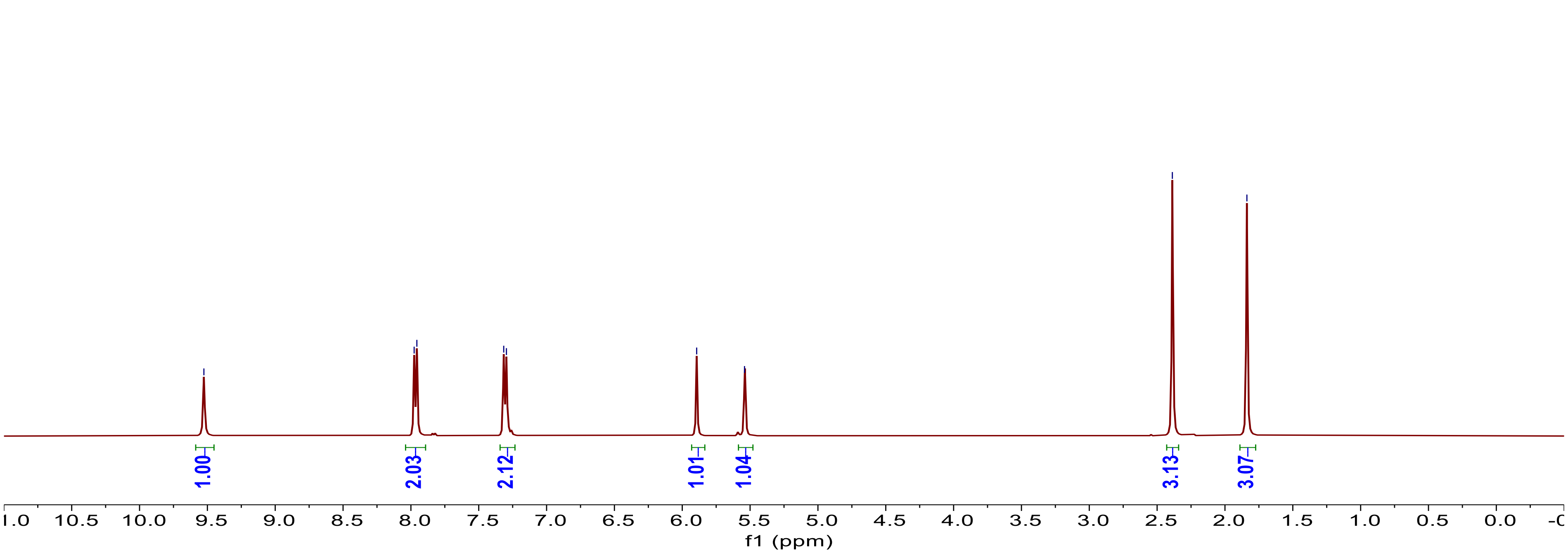

^{1}H NMR Spectrum of 1b at 25 °C ($CDCl_3$)

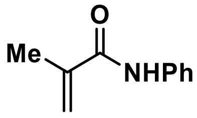

1b

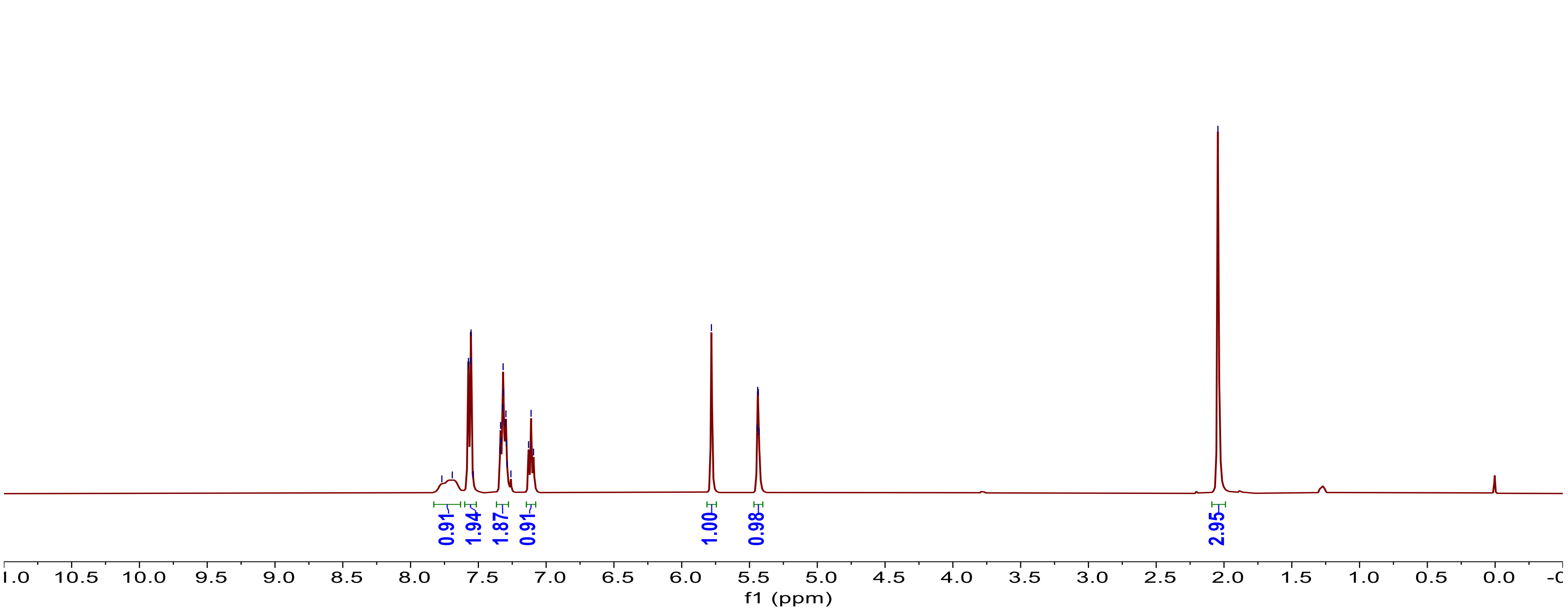

^{1}H NMR Spectrum of 1c at 25 ^{o}C ($CDCl_3$)

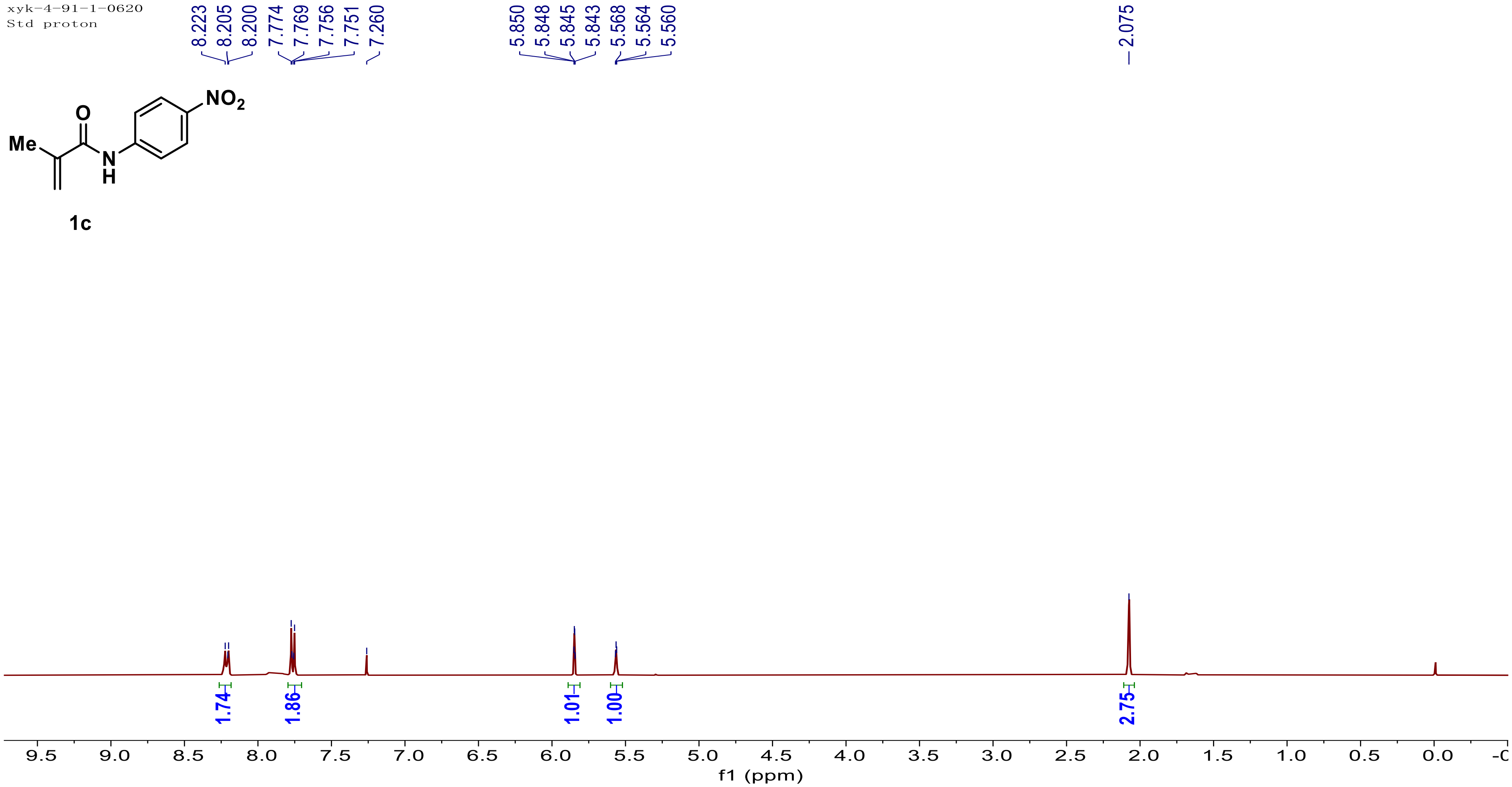

^{1}H NMR Spectrum of 1d at 25 °C ($CDCl_3$)

xyk-4-91-4-H-1022
Std proton

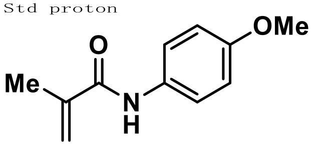

1d

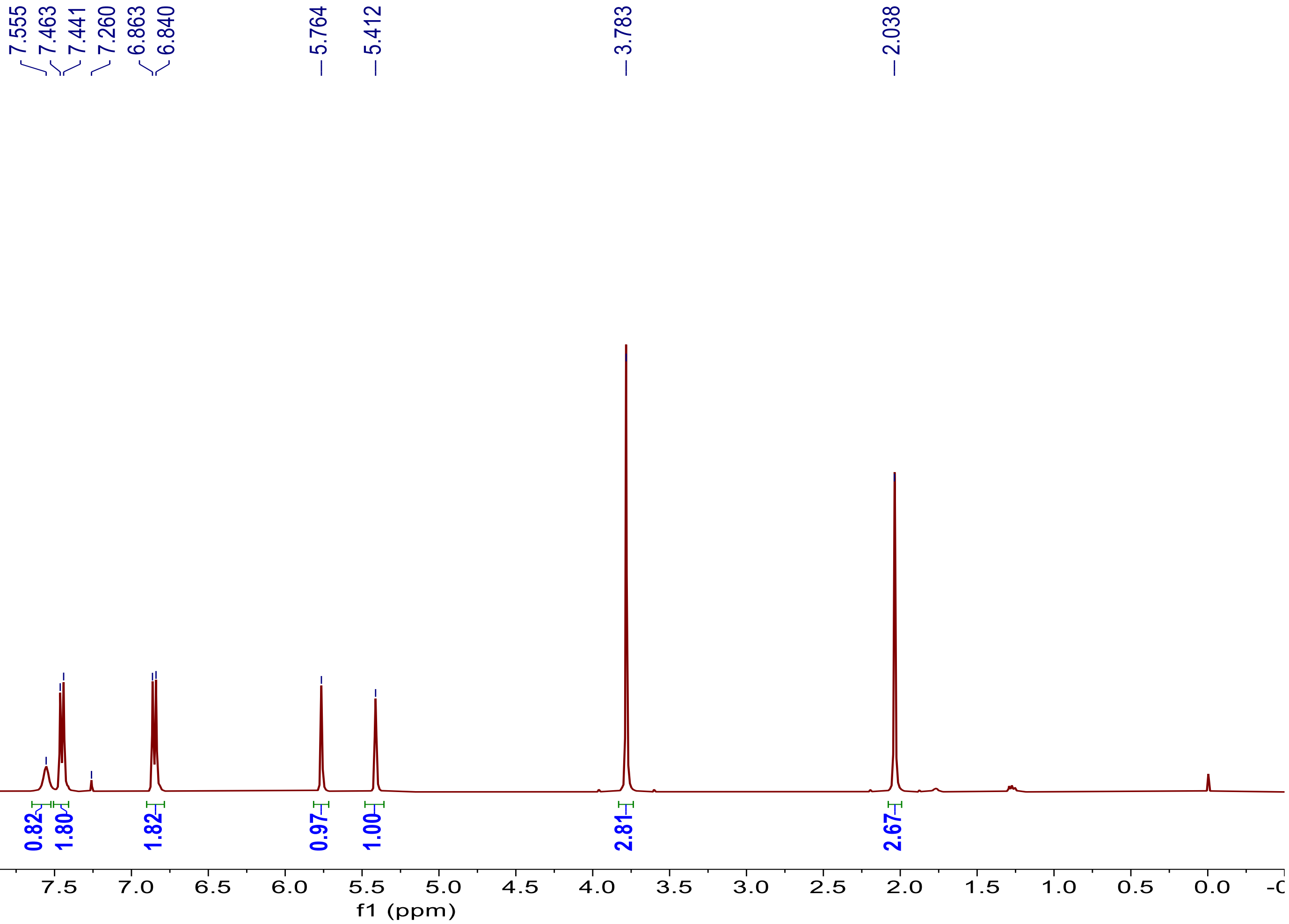

^{1}H NMR Spectrum of 1e at 25 ºC ($CDCl_3$)

xyk-4-91-2-0620
Std proton

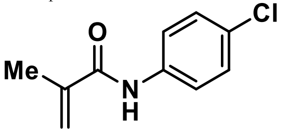

1e

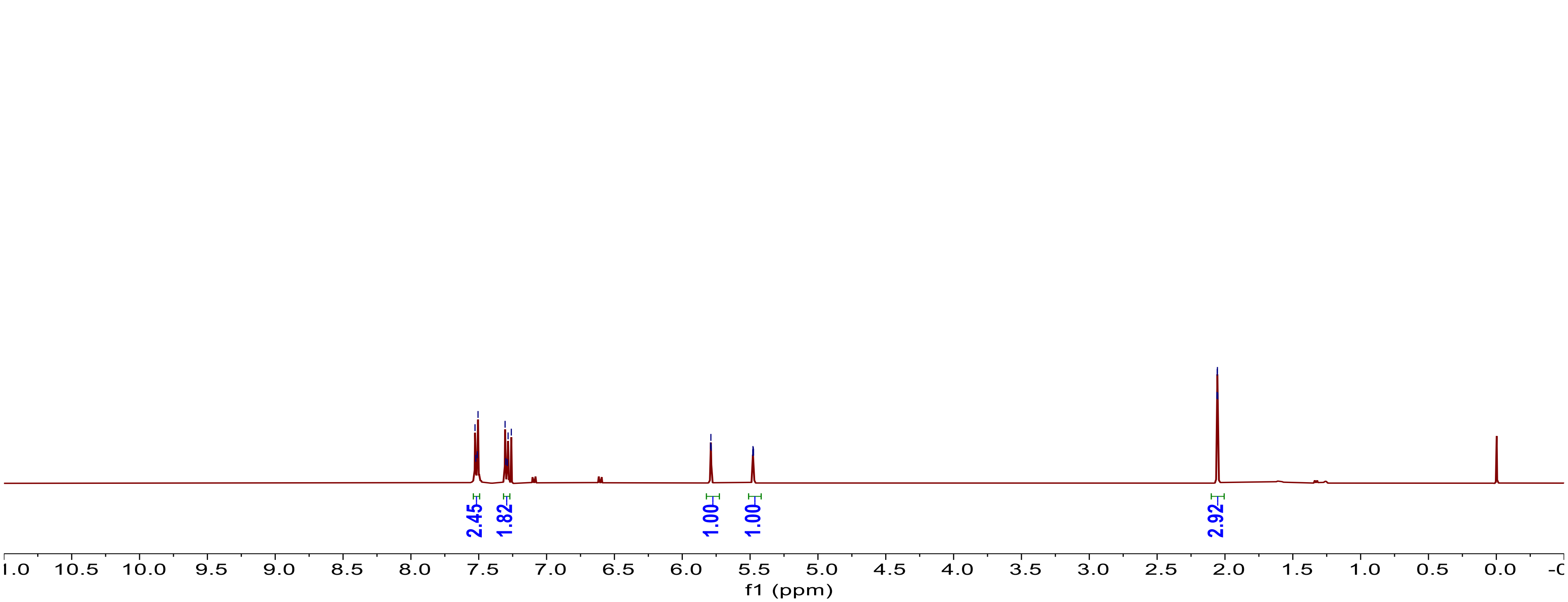

^{1}H NMR Spectrum of 1f at 25 ºC ($CDCl_3$)

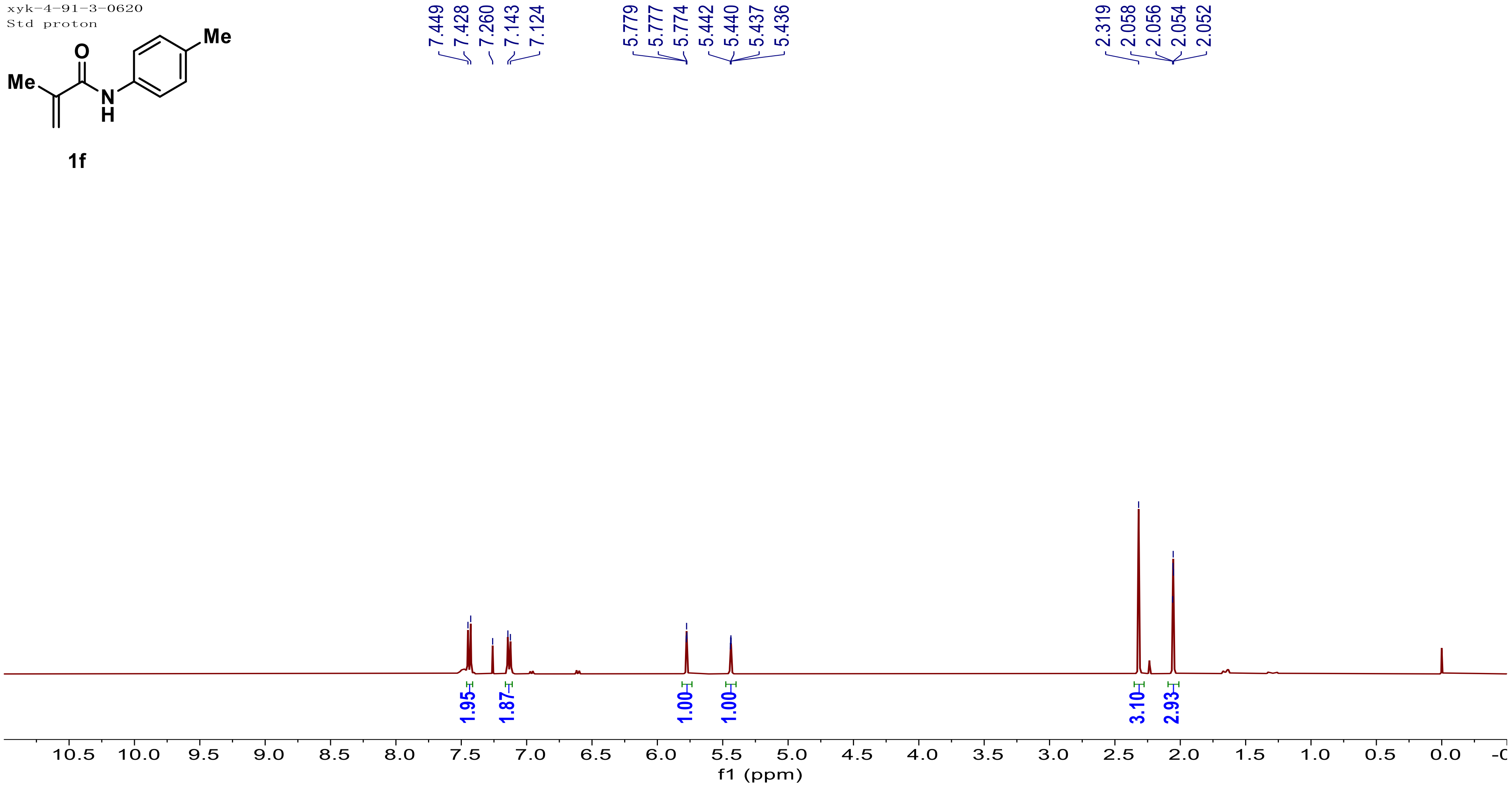

^{1}H NMR Spectrum of 1g at 25 °C ($CDCl_3$)

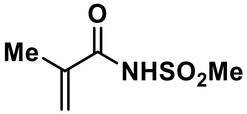

1g

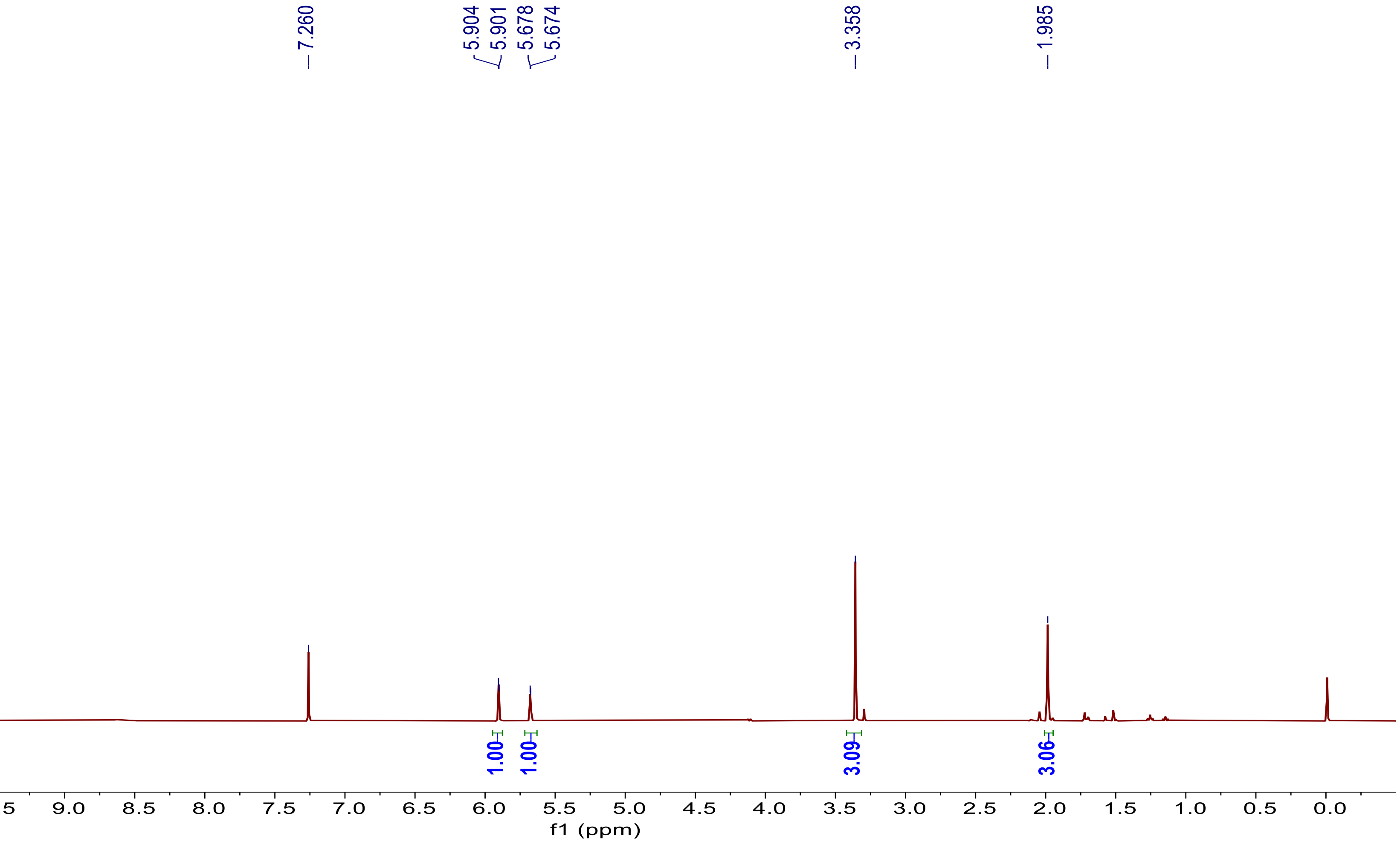

^{1}H NMR Spectrum of 1h at 25 °C ($CDCl_3$)

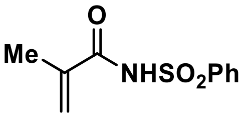

1h

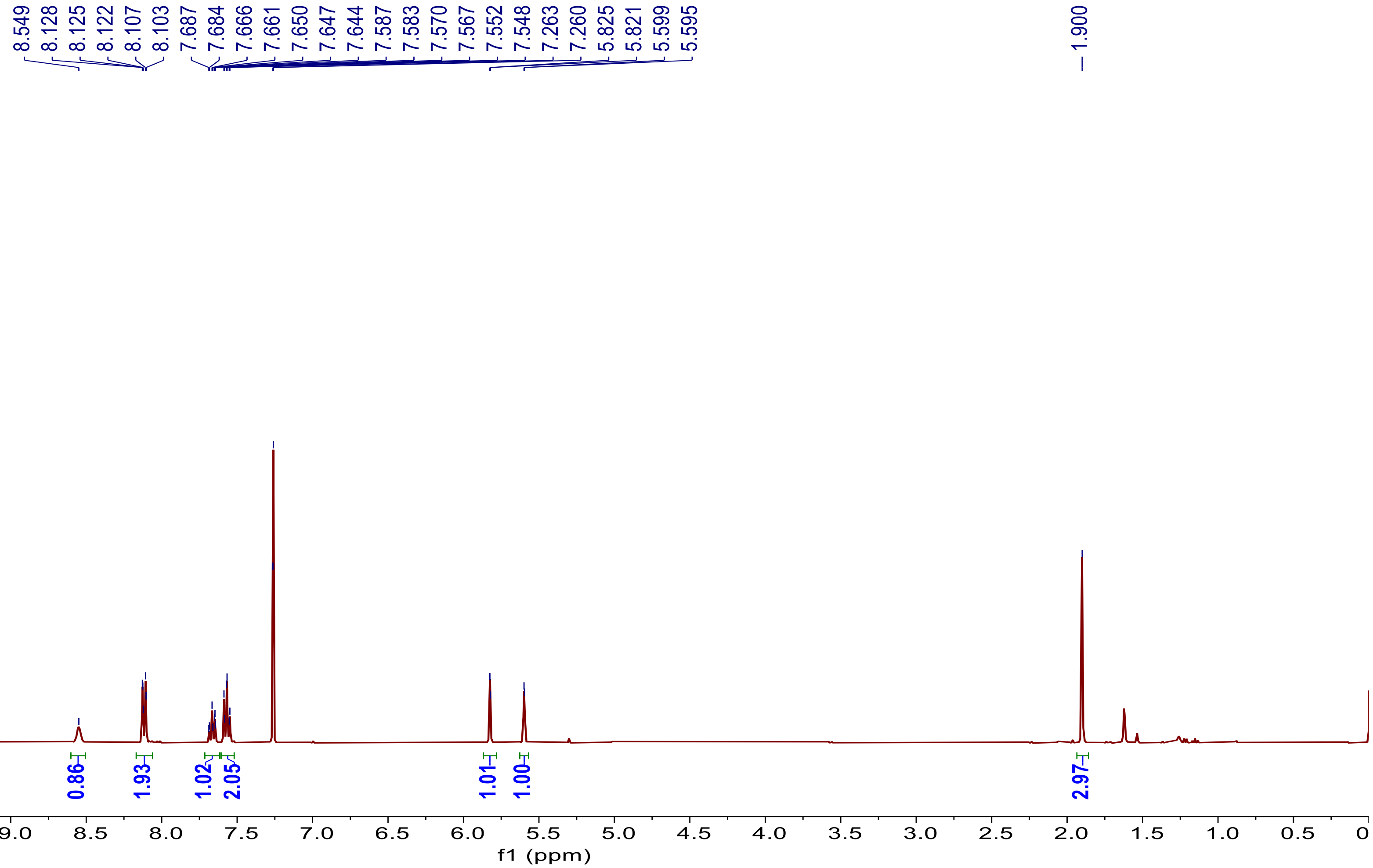

^{1}H NMR Spectrum of 1i at 25 ºC ($CDCl_3$)

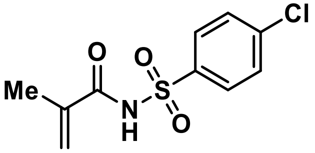

1i

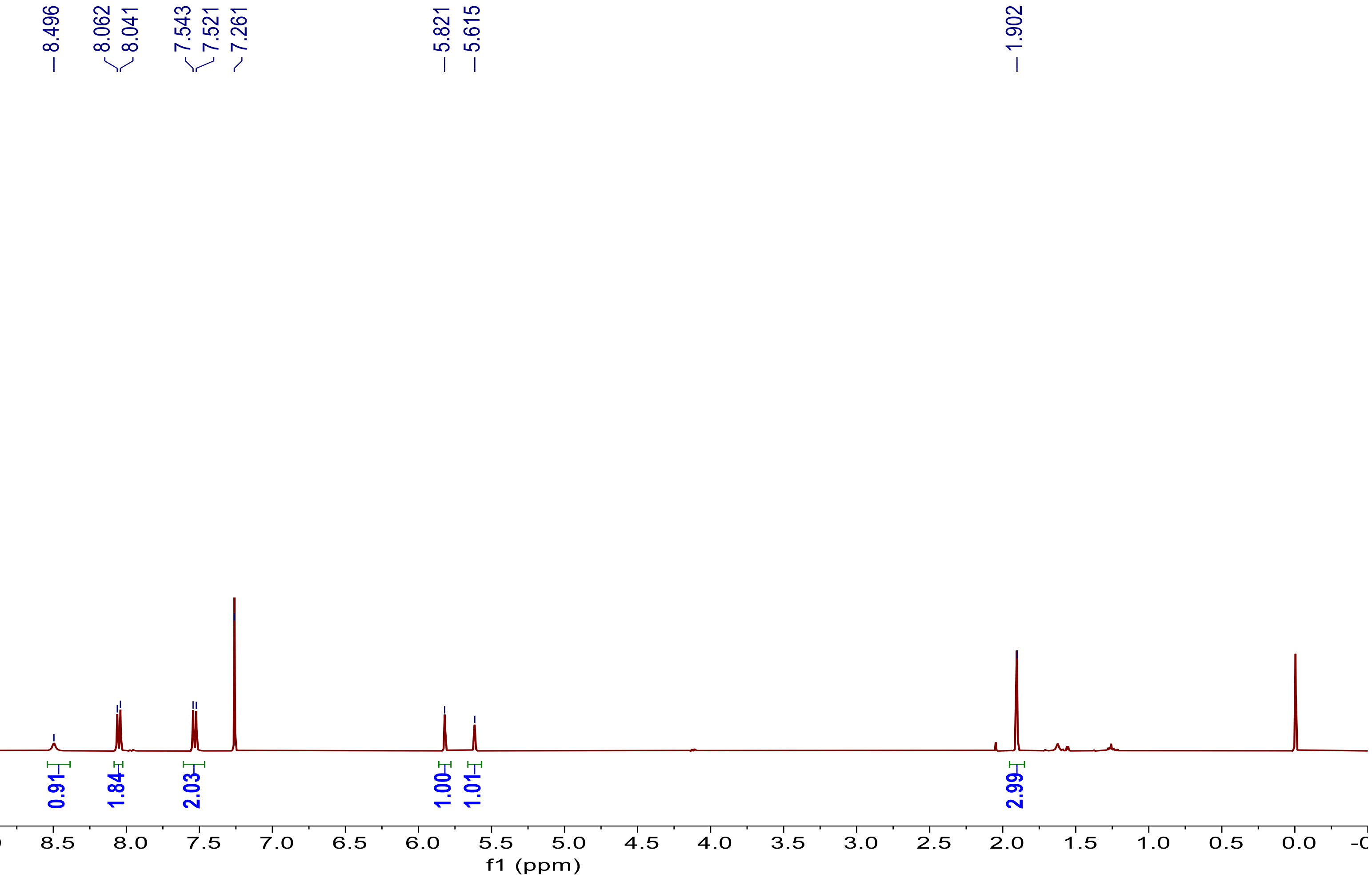

^{1}H NMR Spectrum of 1j at 25 ºC ($CDCl_3$)

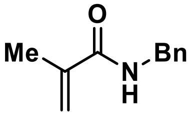

1j

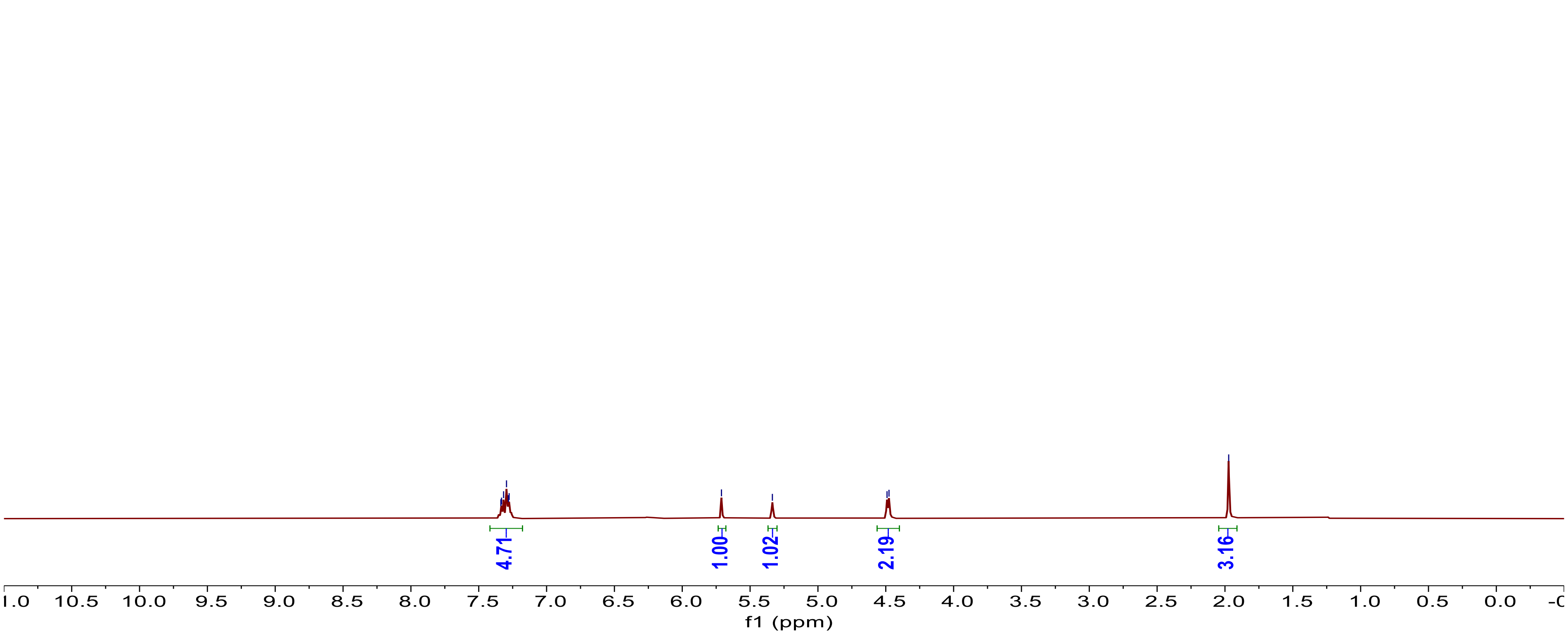

^{1}H NMR Spectrum of 3a at 25 ºC ($CDCl_3$)

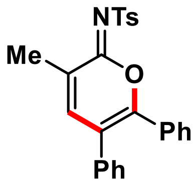

3a

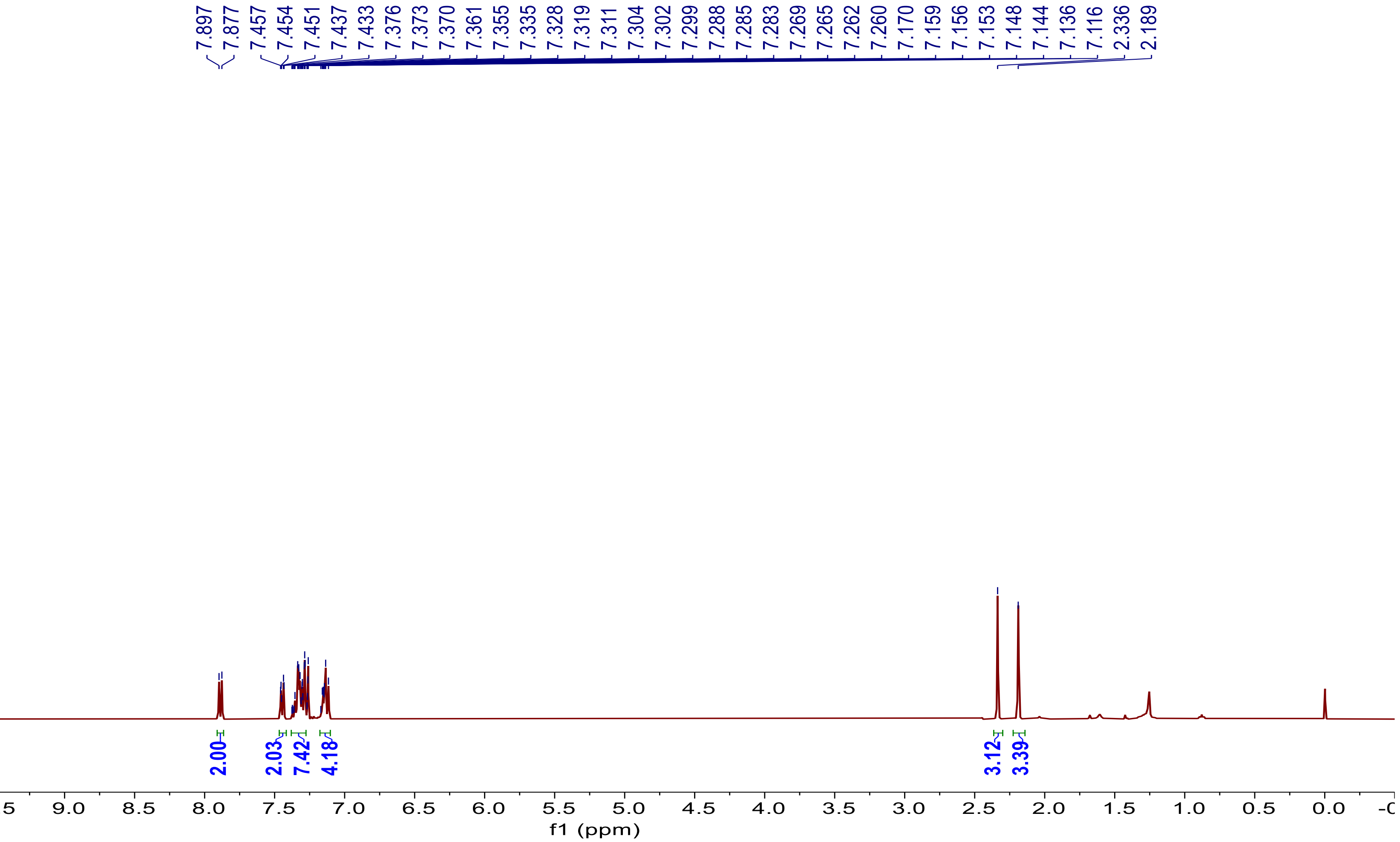

^{13}C NMR Spectrum of 3a at 25 ºC ($CDCl_3$)

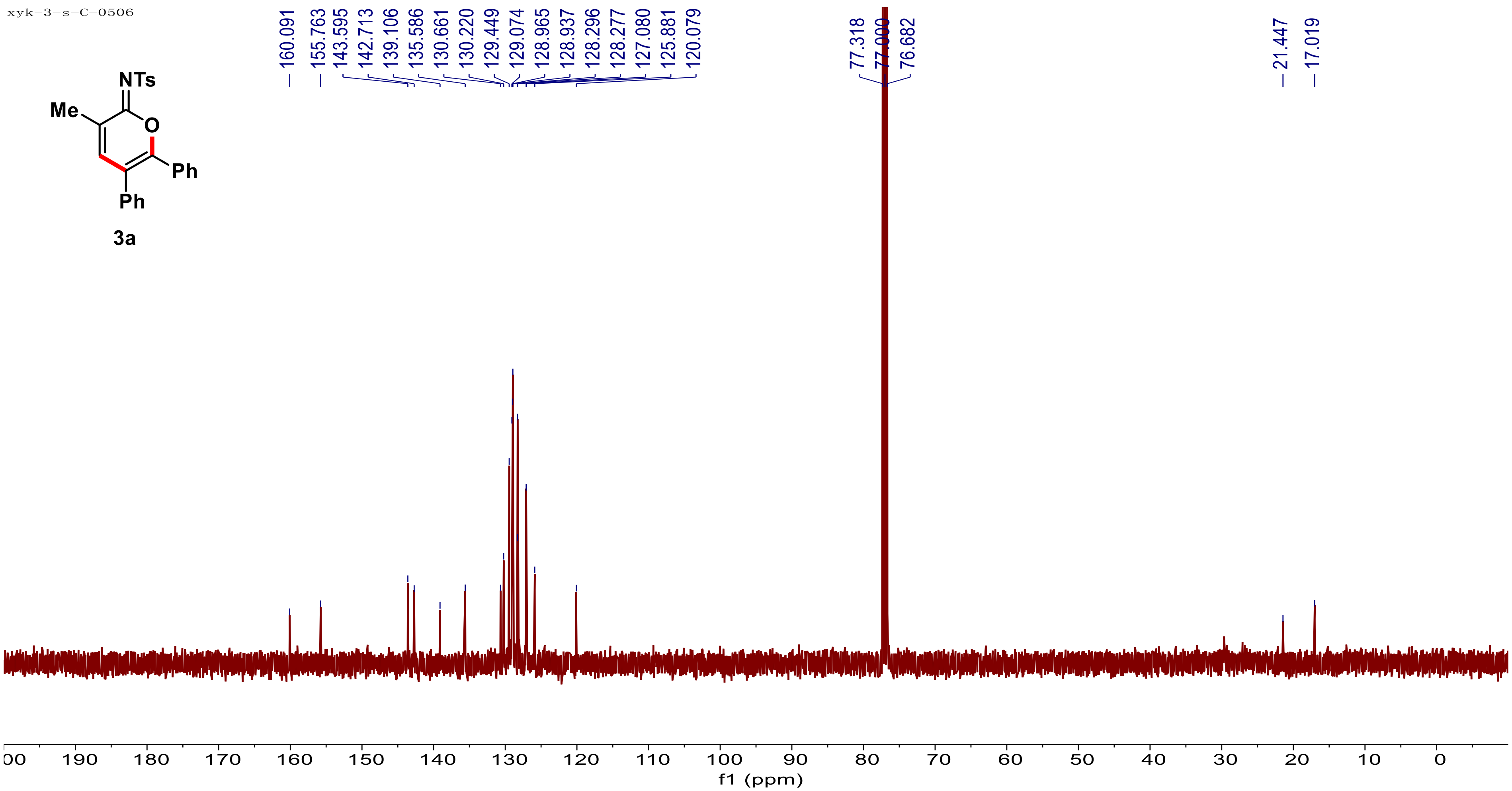

^{1}H NMR Spectrum of 4b at 25 °C ($CDCl_3$)

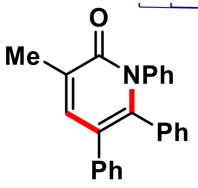

4b

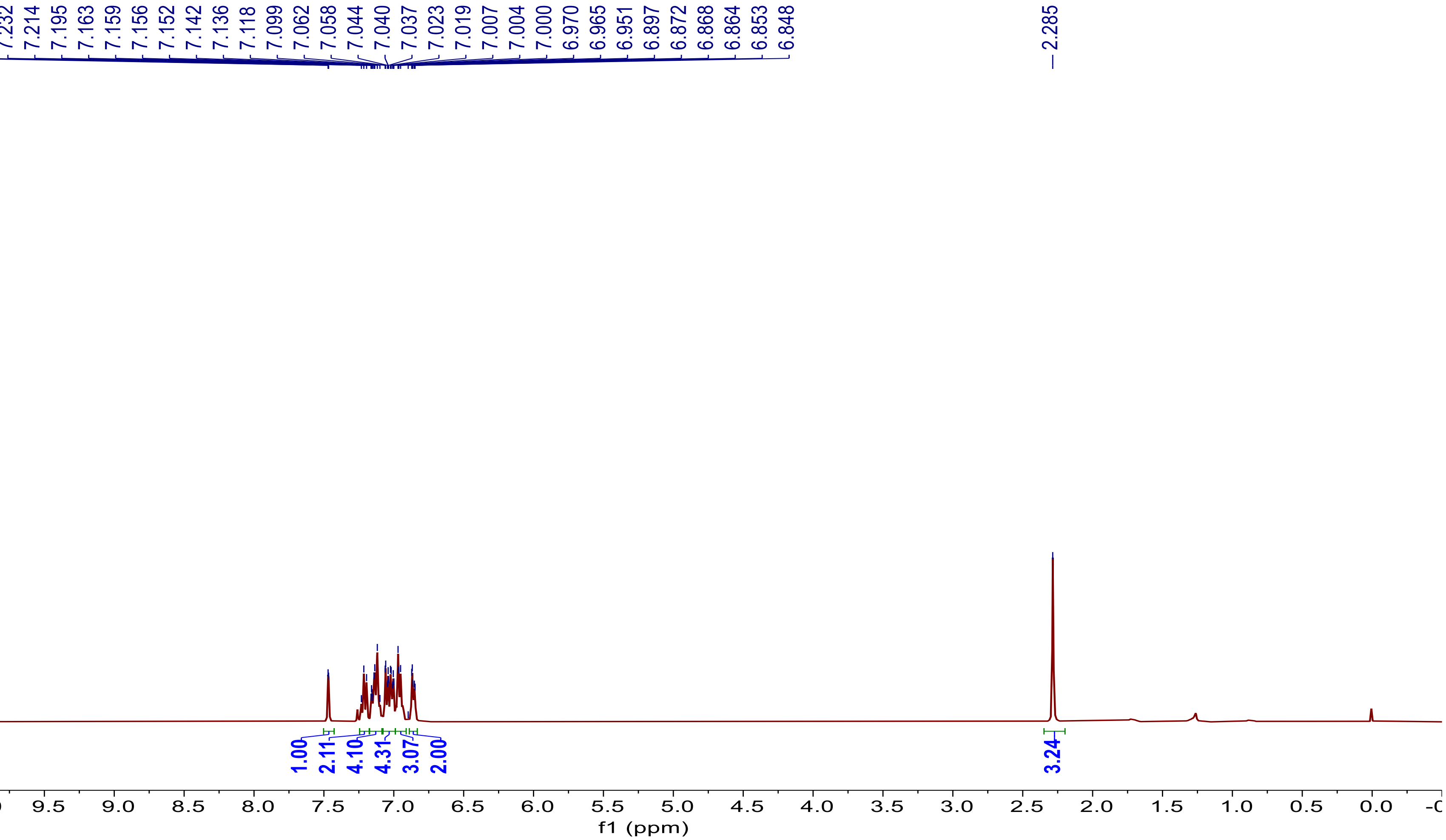

^{13}C NMR Spectrum of 4b at 25 ºC ($CDCl_3$)

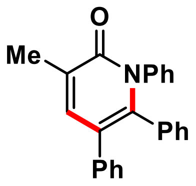

4b

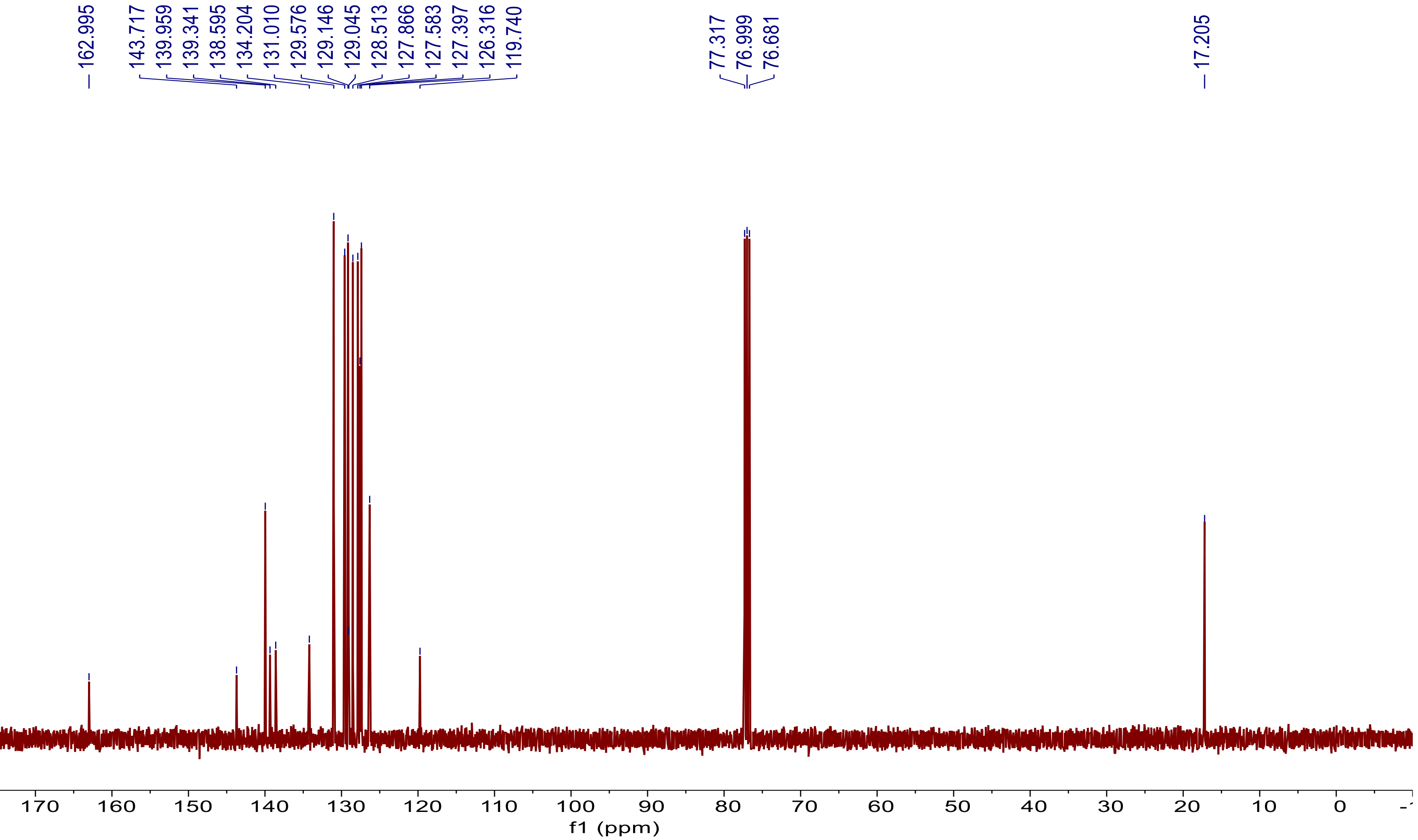

^{1}H NMR Spectrum of 4c at 25 °C ($CDCl_3$)

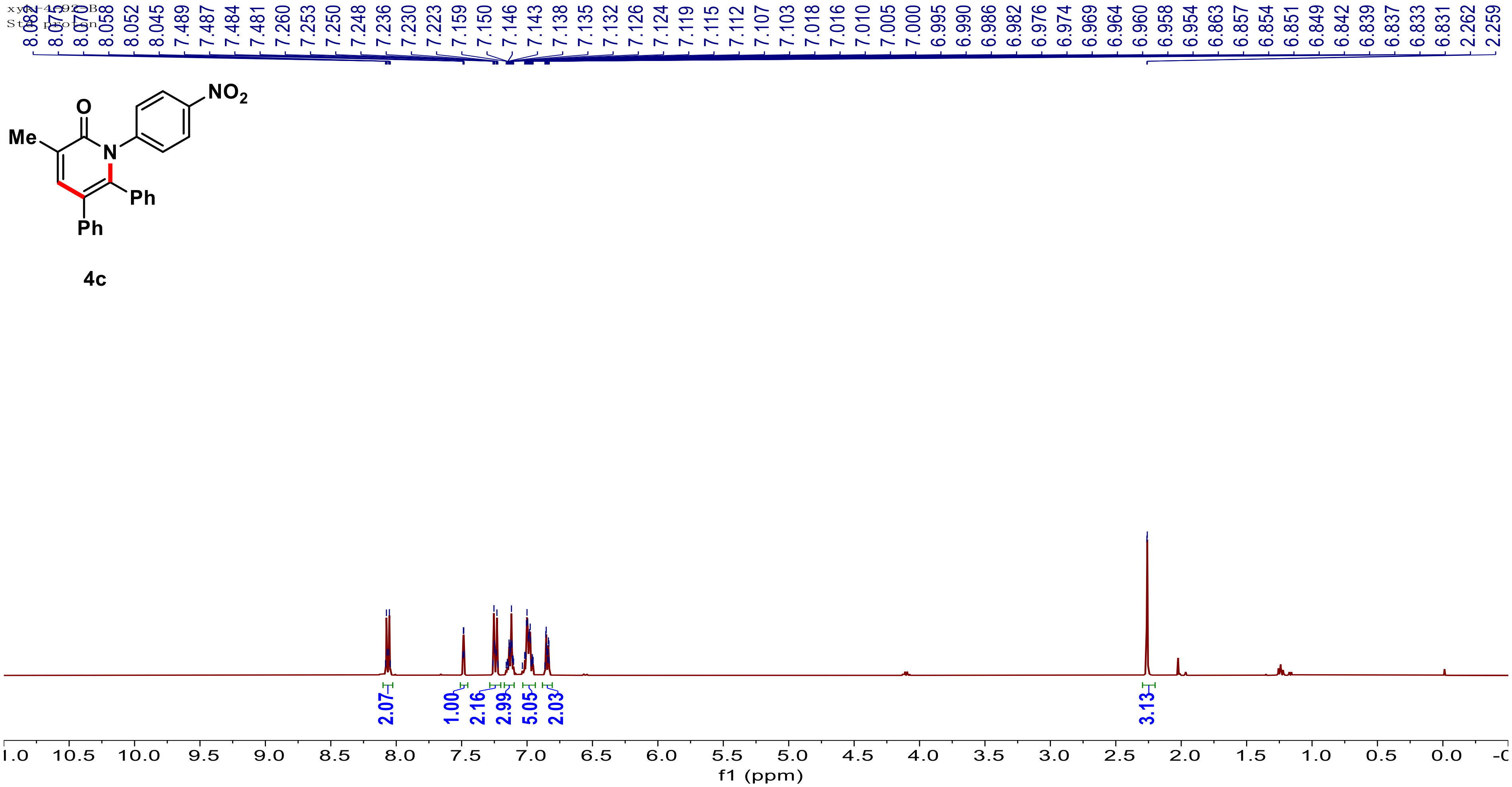

^{13}C NMR Spectrum of 4c at 25 ºC ($CDCl_3$)

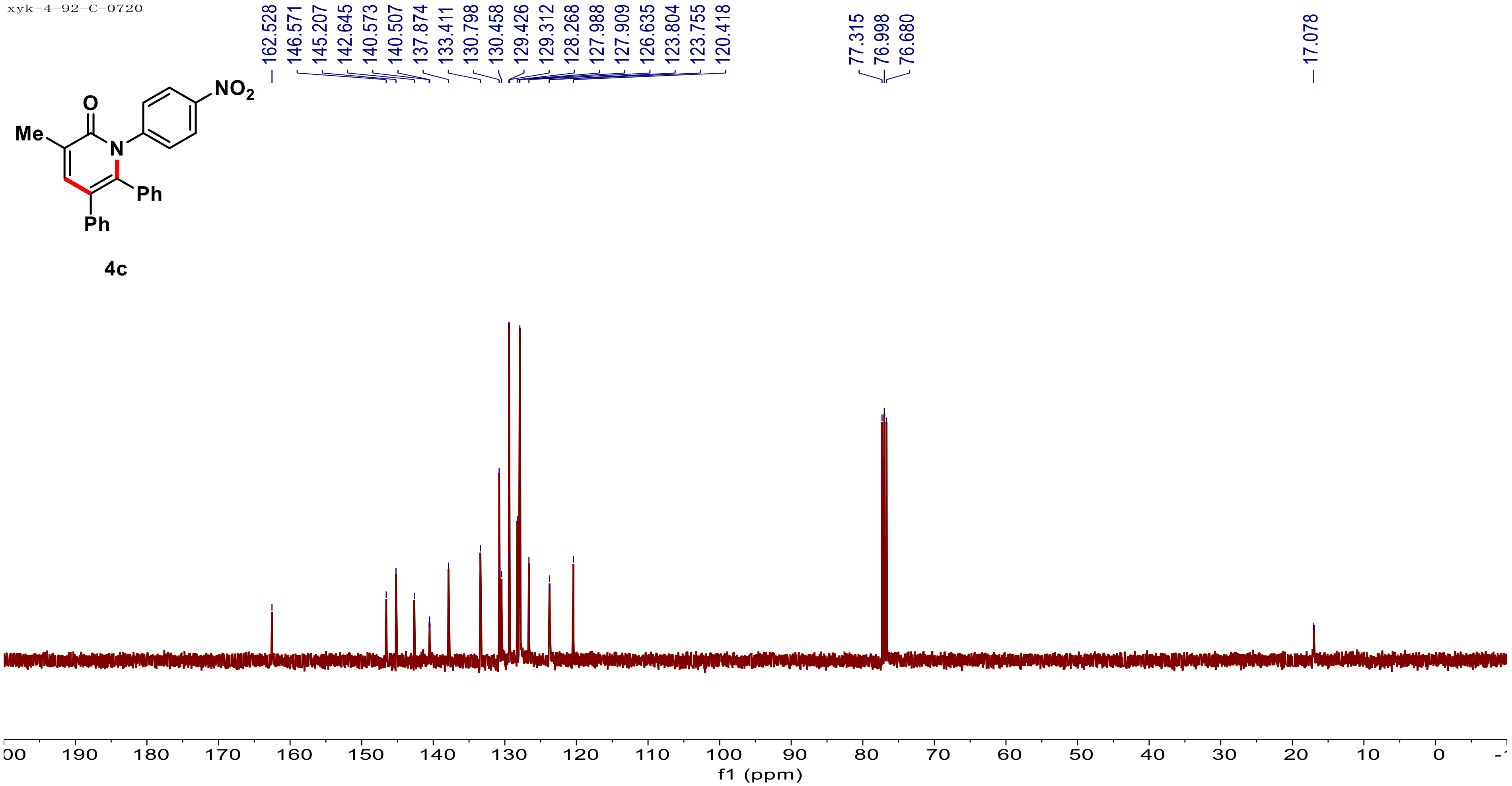

^{1}H NMR Spectrum of 4d at 25 °C ($CDCl_3$)

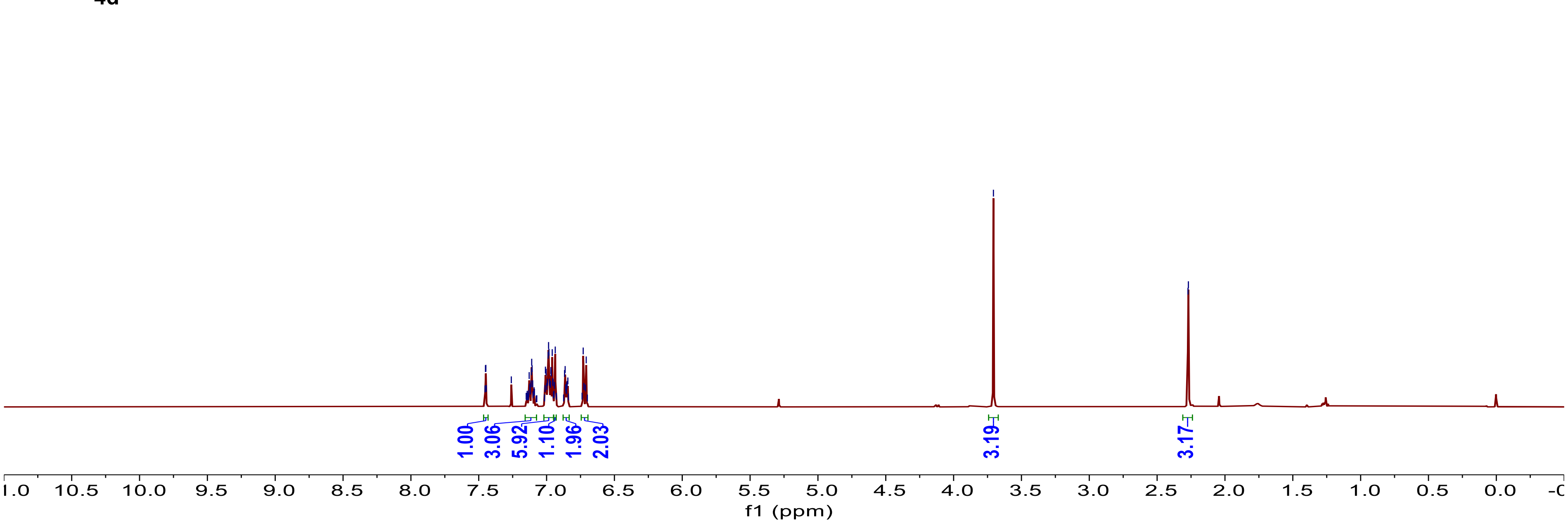

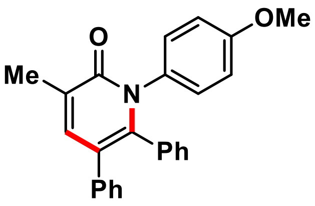

4d

^{13}C NMR Spectrum of 4d at 25 ºC ($CDCl_3$)

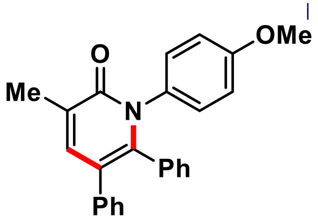

4d

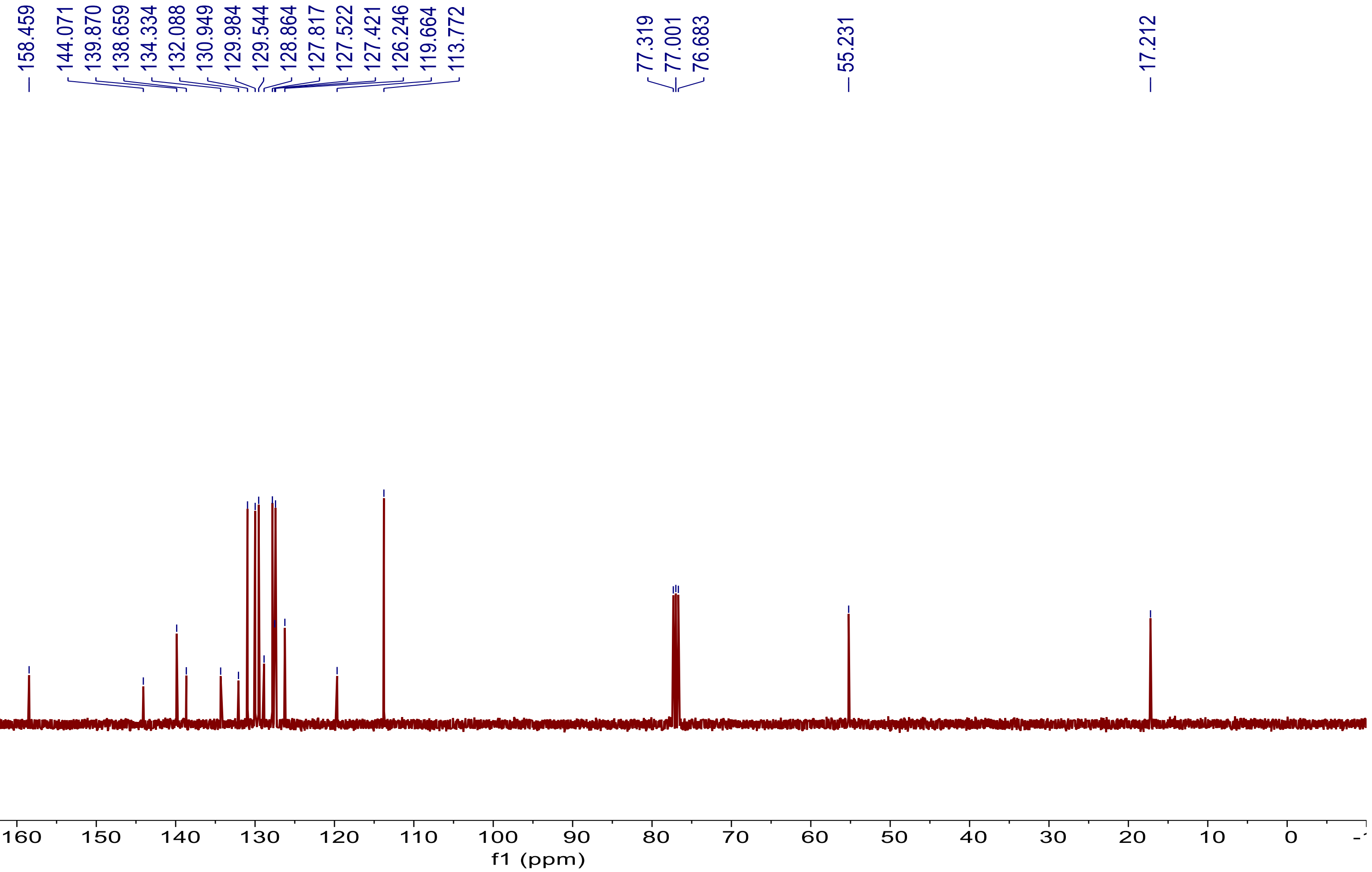

^{1}H NMR Spectrum of 4e at 25 ^{o}C ($CDCl_3$)

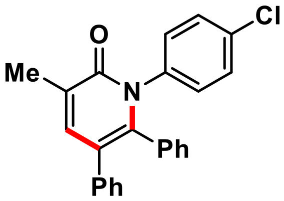

4e

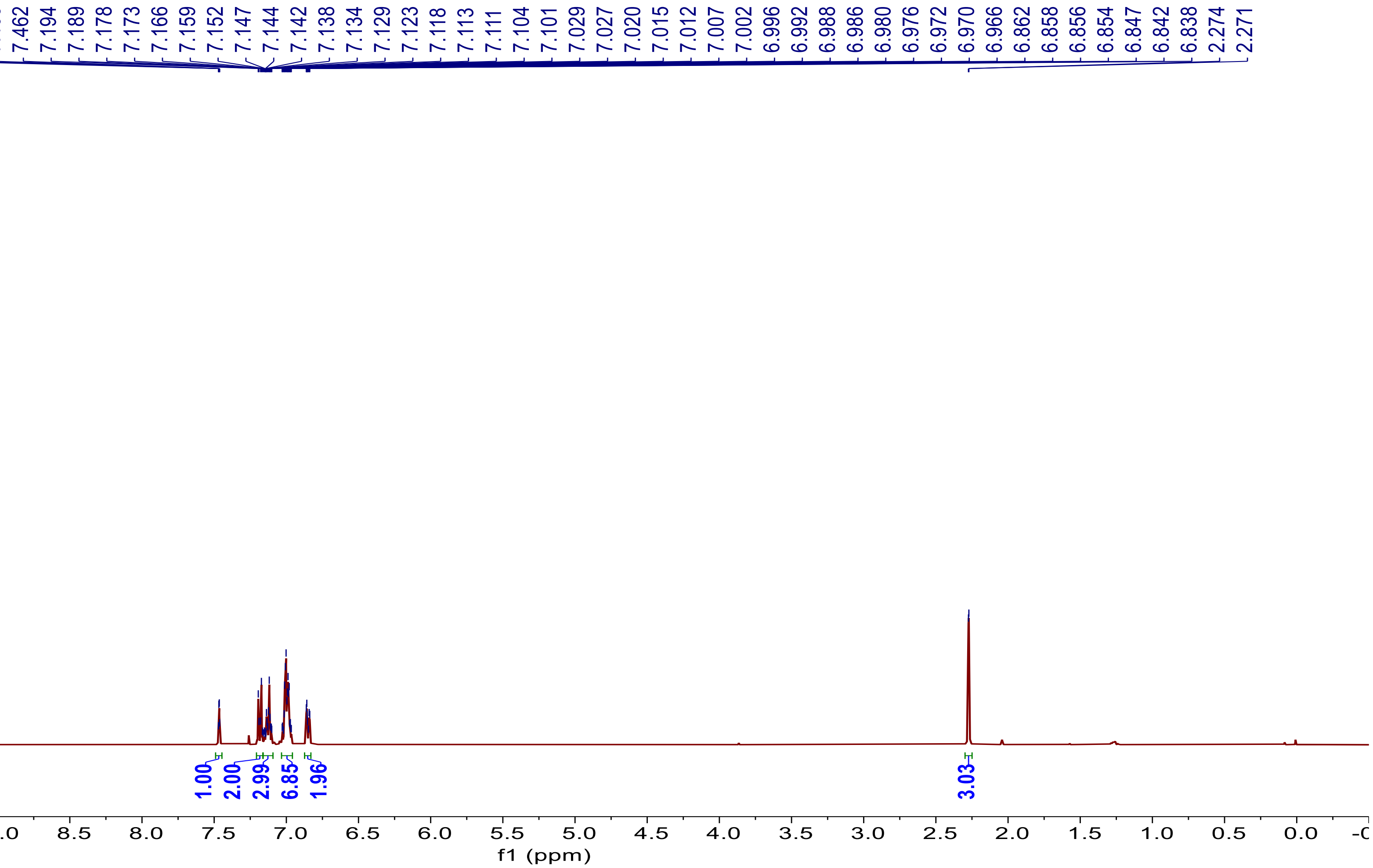

^{13}C NMR Spectrum of 4e at 25 ºC ($CDCl_3$)

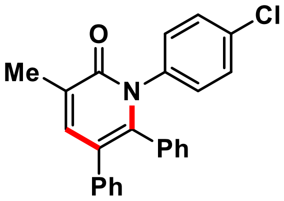

4e

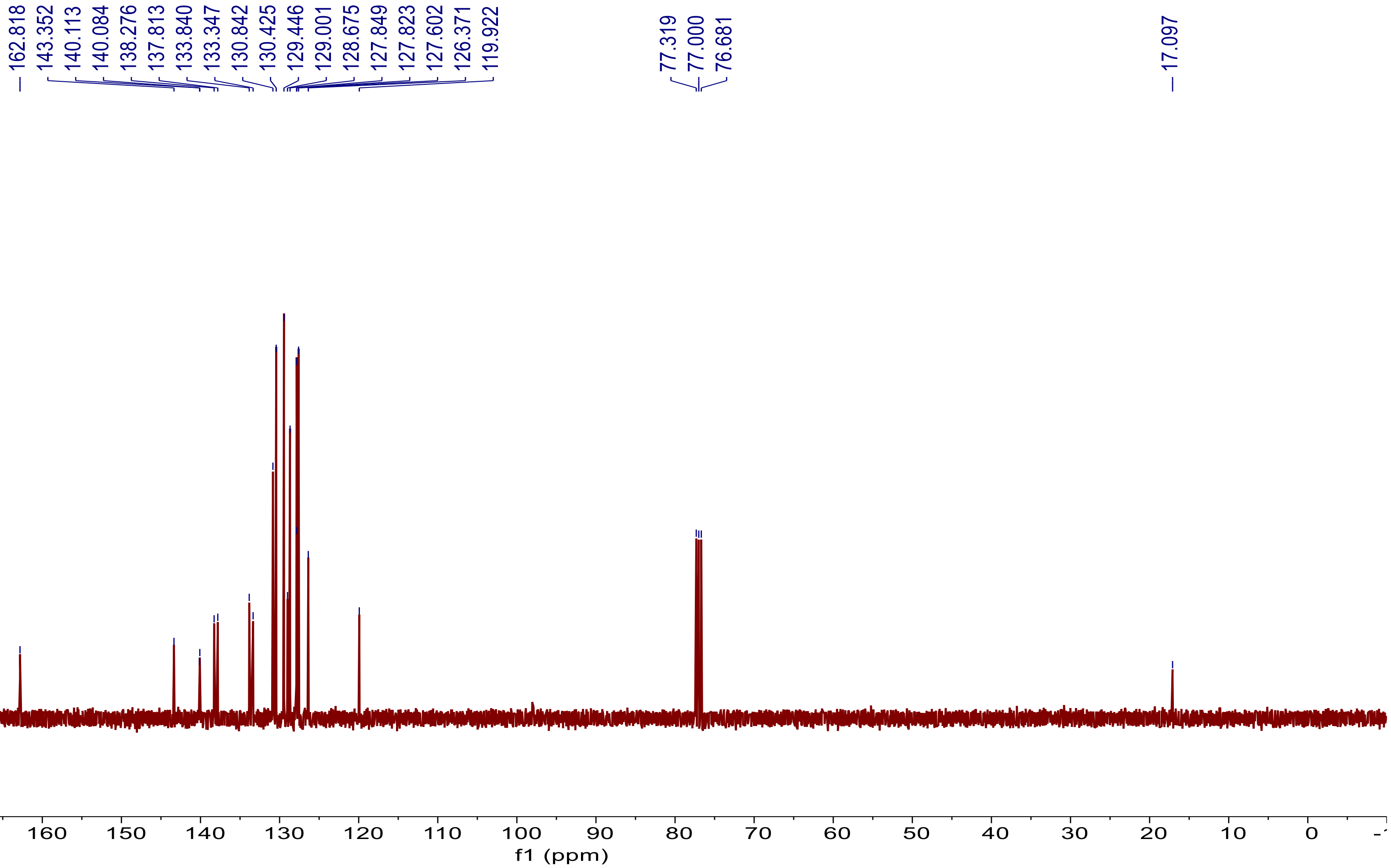

^{1}H NMR Spectrum of 4f at 25 ºC ($CDCl_3$)

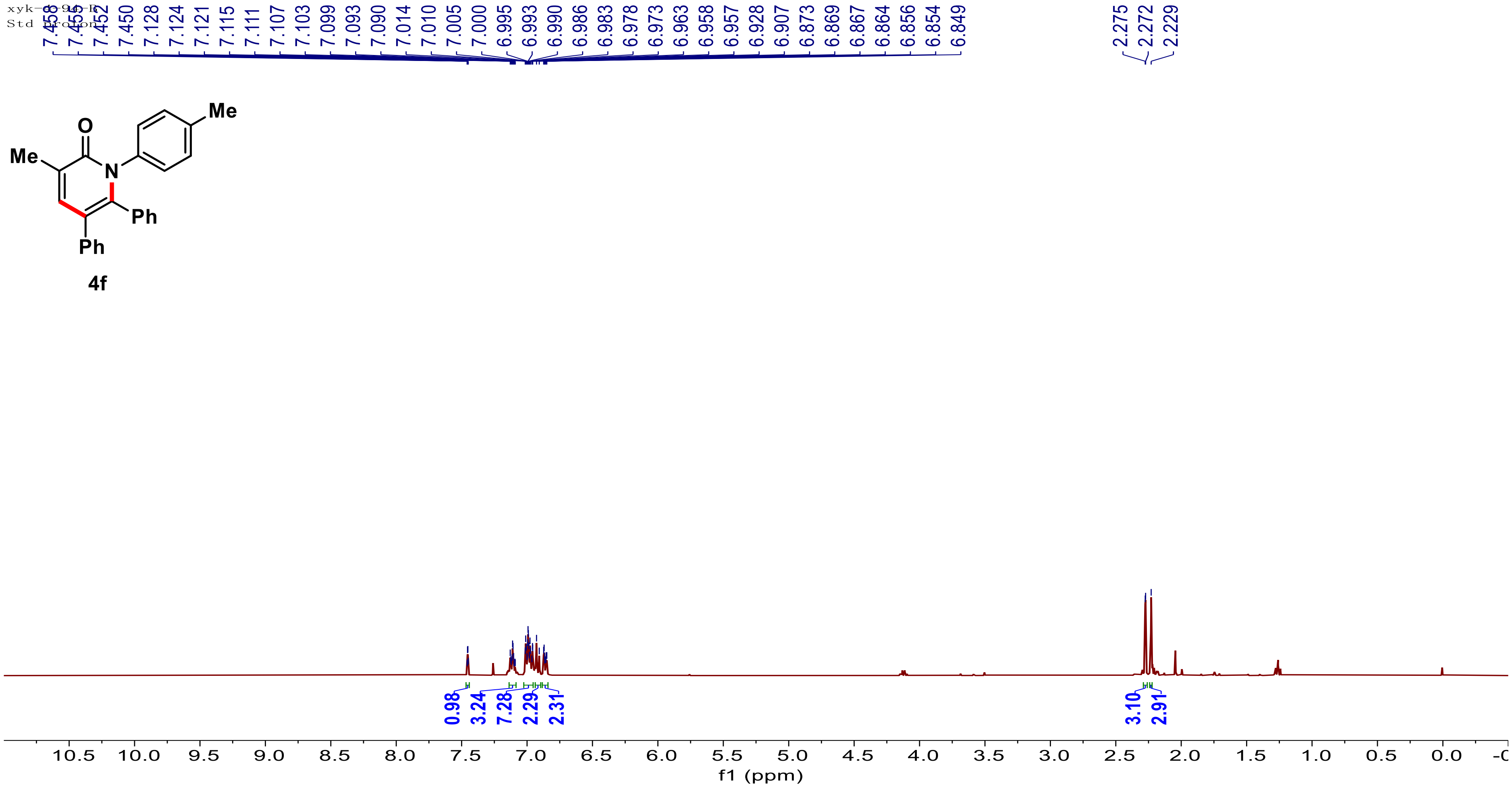

^{13}C NMR Spectrum of 4f at 25 °C ($CDCl_3$)

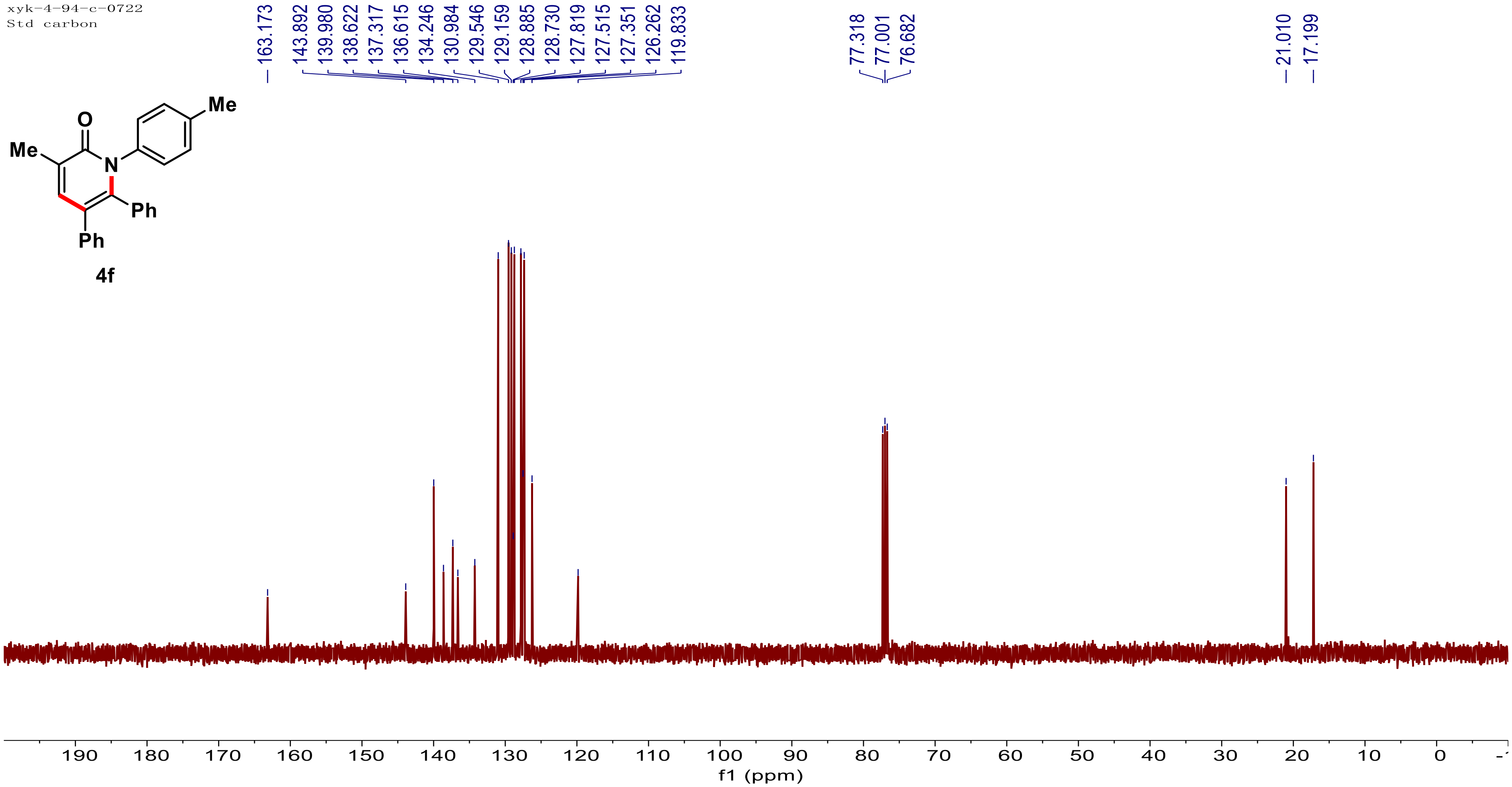

^{1}H NMR Spectrum of 4g at 25 °C ($CDCl_3$)

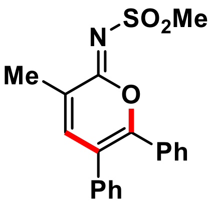

4g

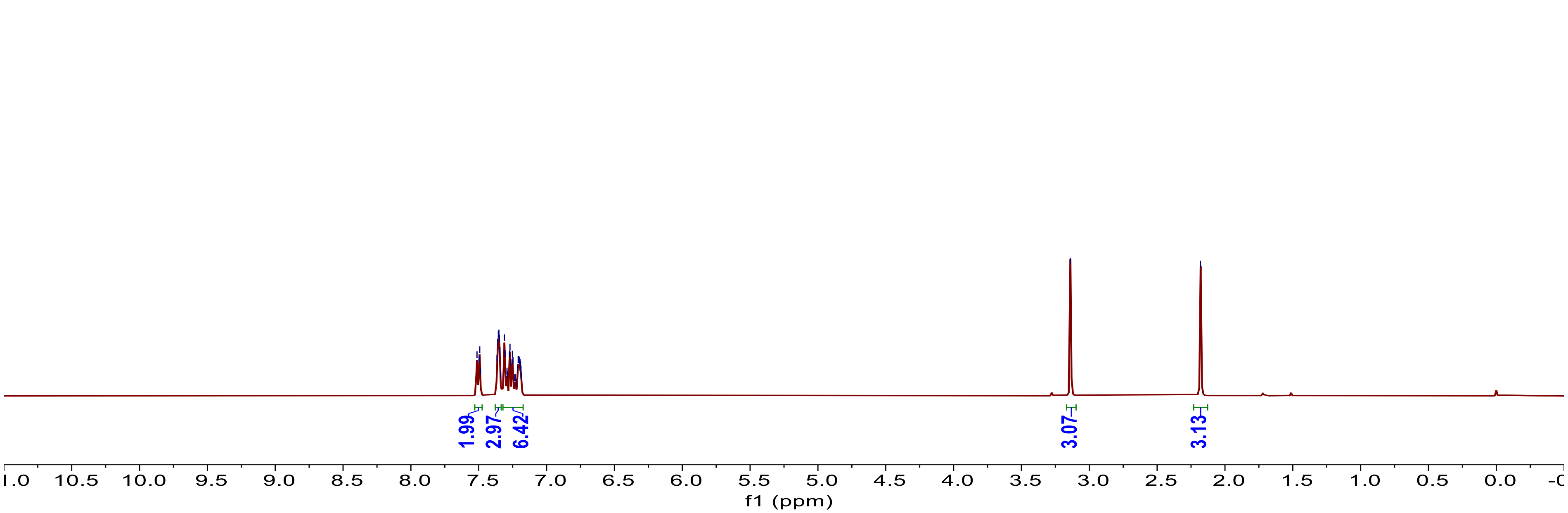

^{13}C NMR Spectrum of 4g at 25 ºC ($CDCl_3$)

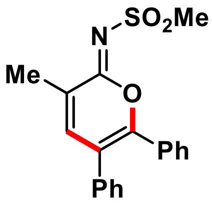

4g

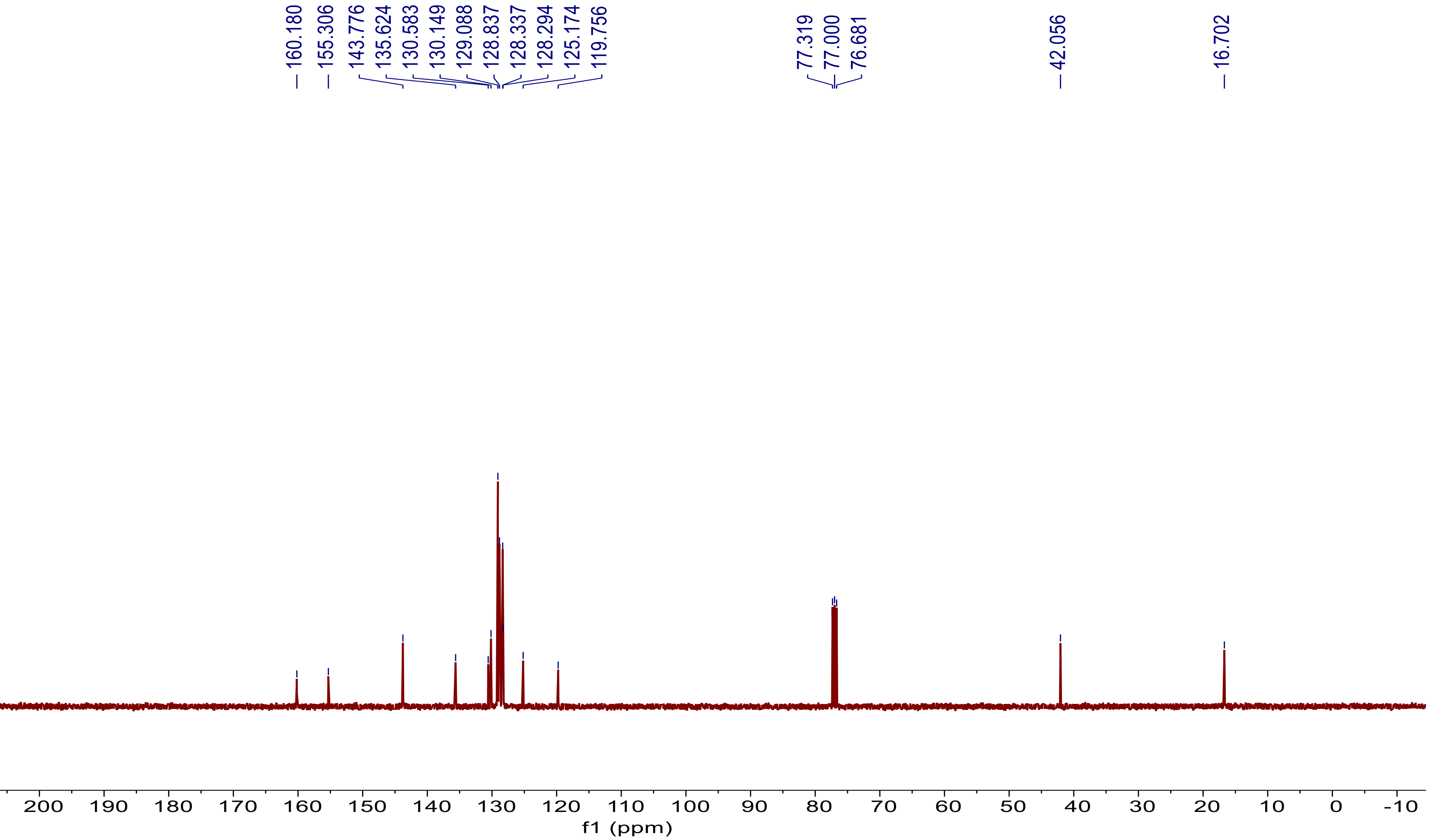

^{1}H NMR Spectrum of 4h at 25 °C ($CDCl_3$)

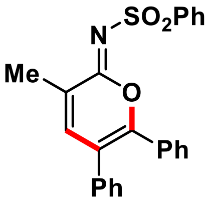

4h

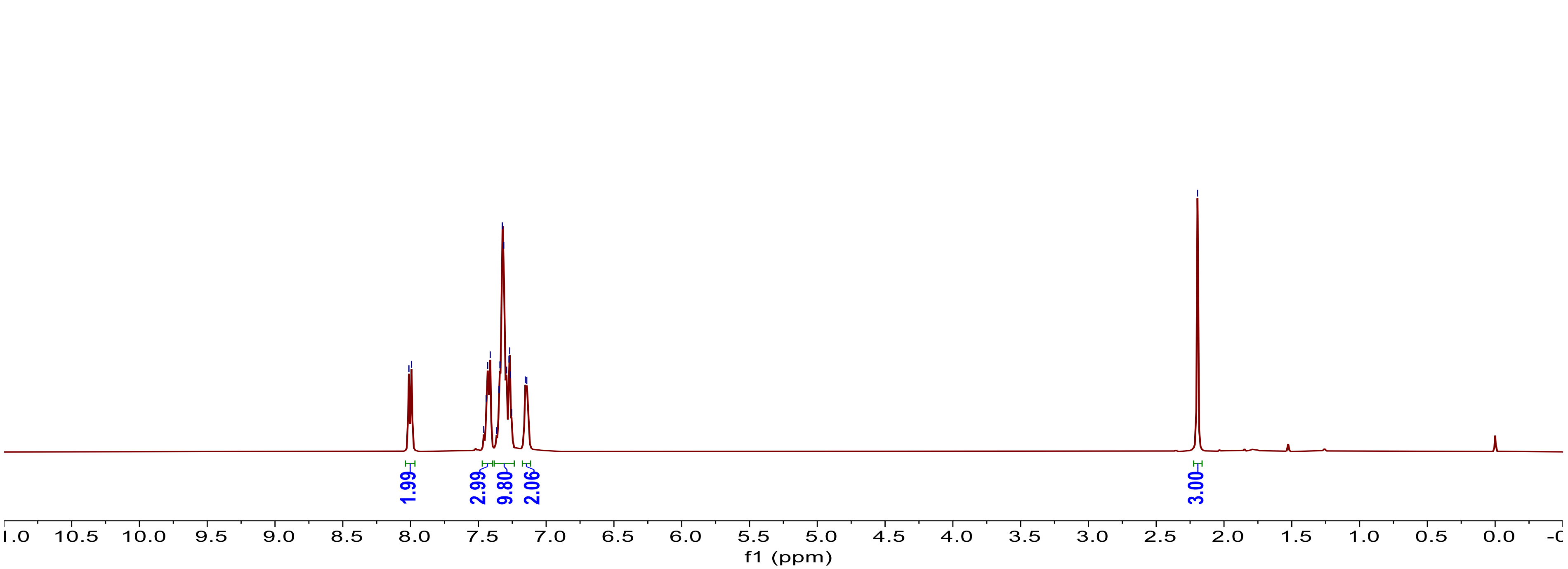

^{13}C NMR Spectrum of 4h at 25 °C ($CDCl_3$)

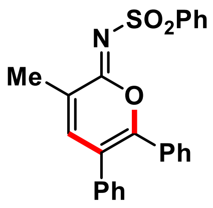

4h

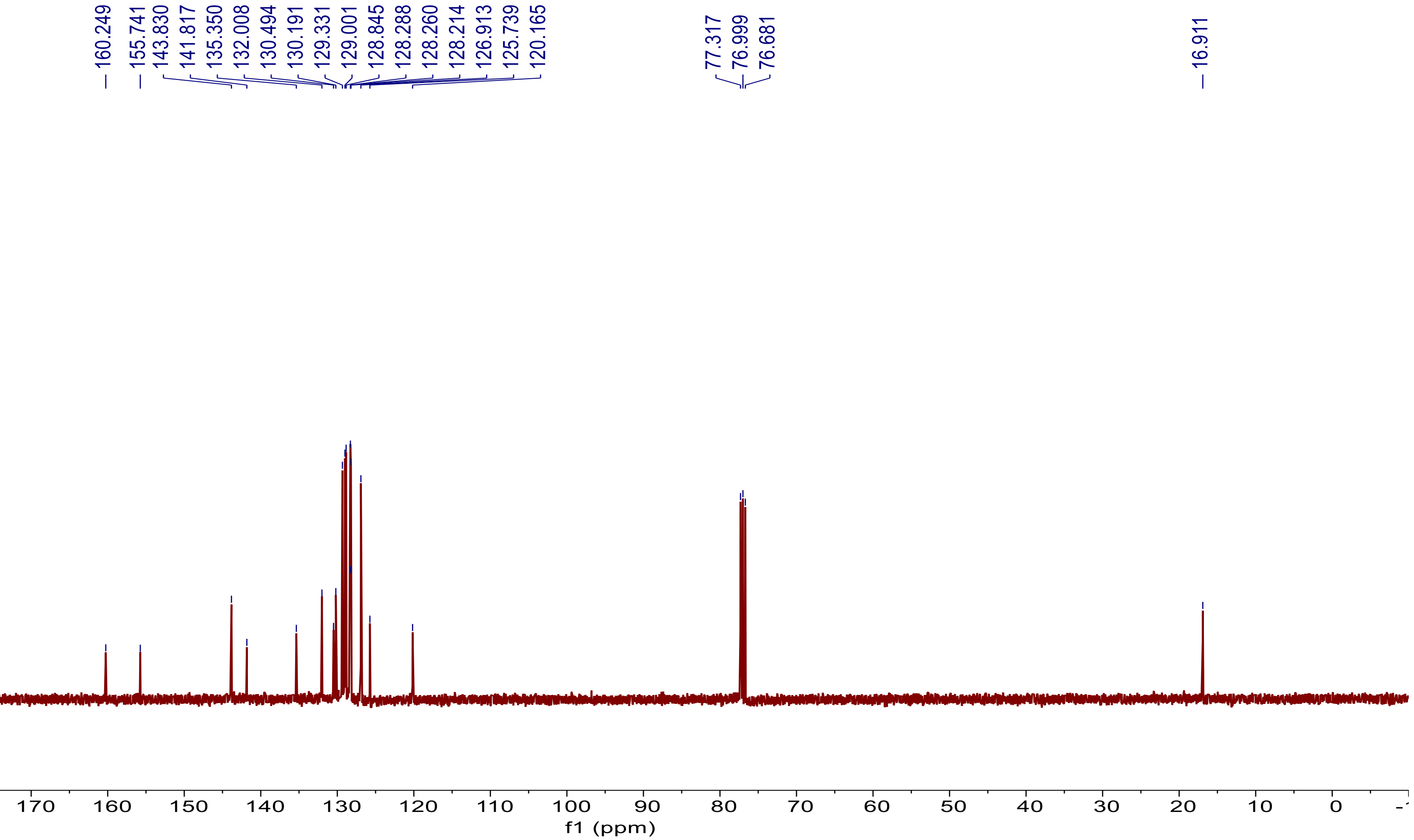

^{1}H NMR Spectrum of 4i at 25 ºC ($CDCl_3$)

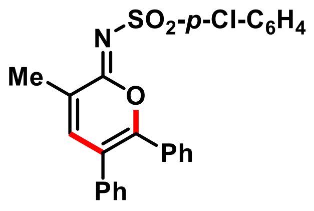

4i

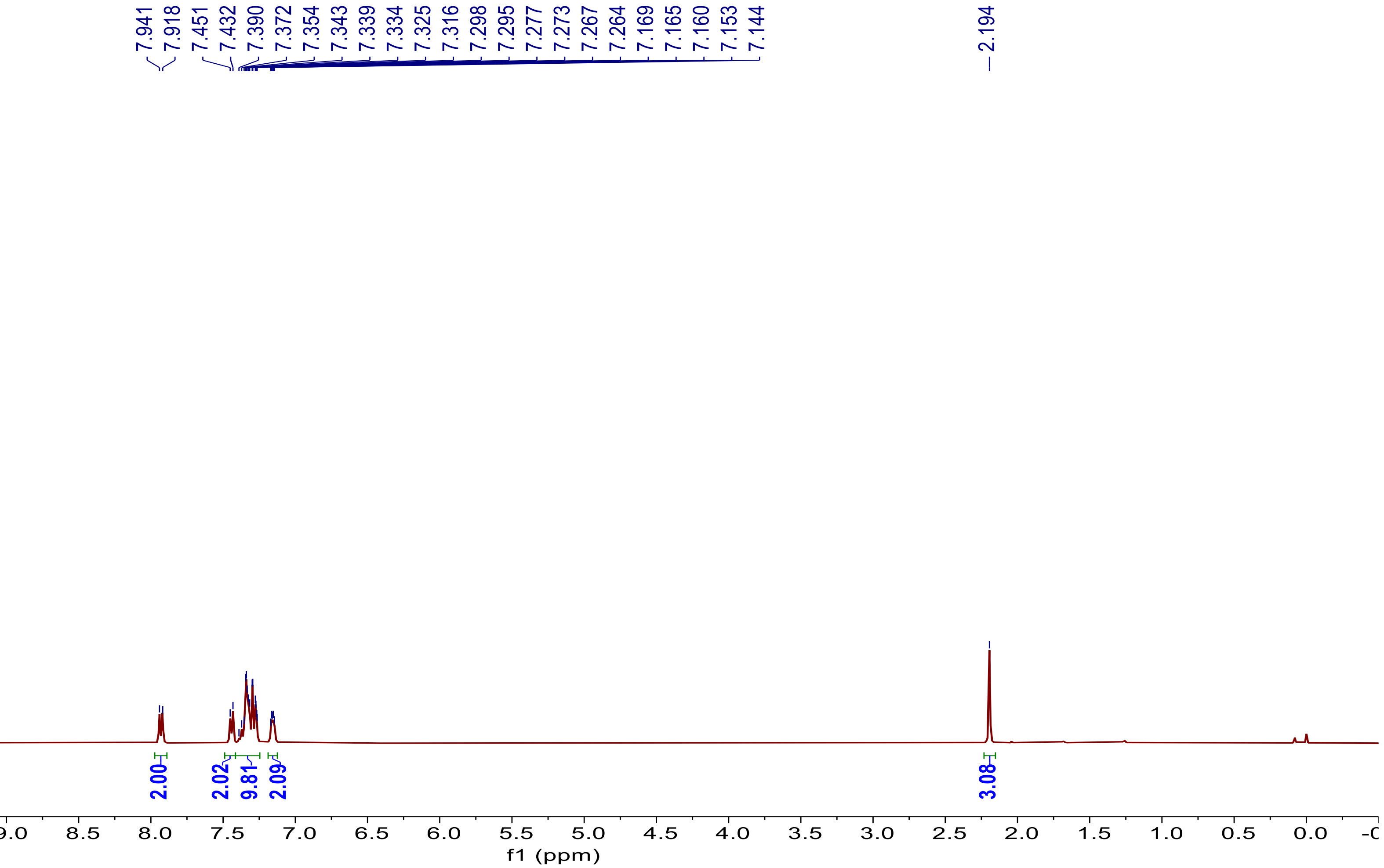

^{13}C NMR Spectrum of 4i at 25 °C ($CDCl_3$)

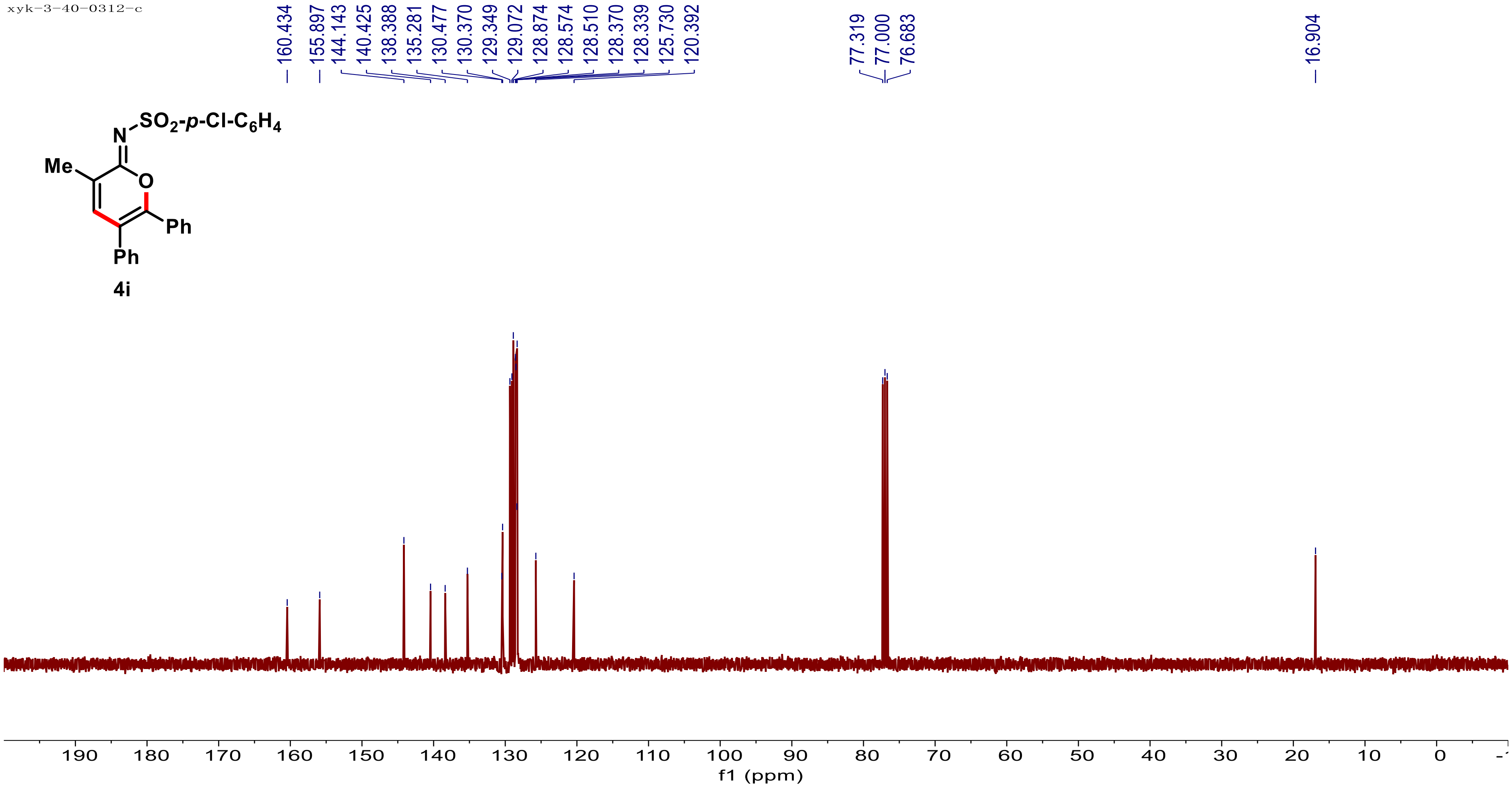

4i

^{1}H NMR Spectrum of 5a at 25 °C (CDCl$_3$)

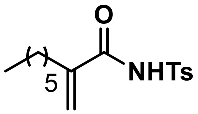

5a

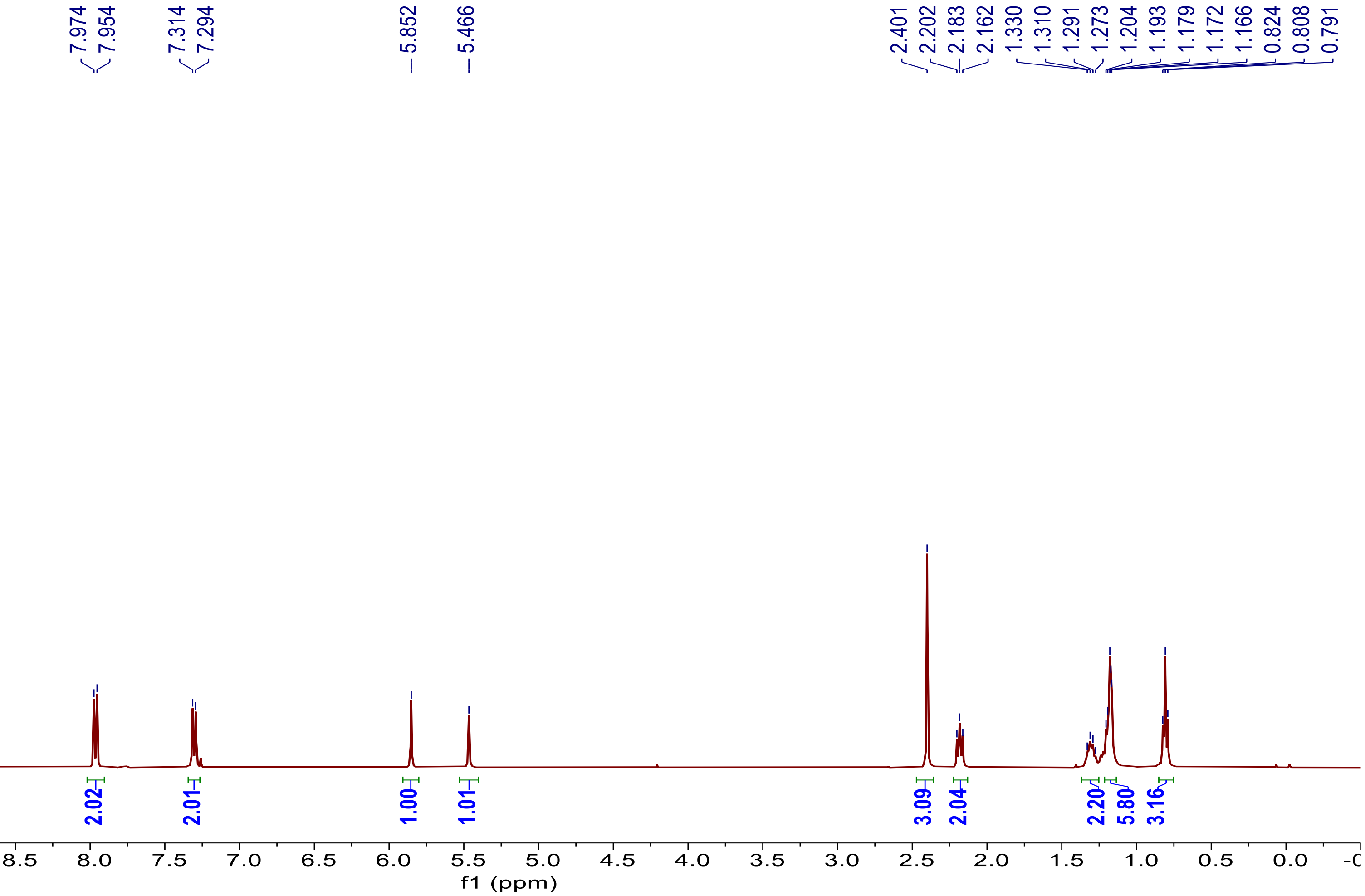

^{1}H NMR Spectrum of 5b at 25 °C ($CDCl_3$)

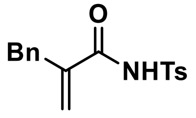

5b

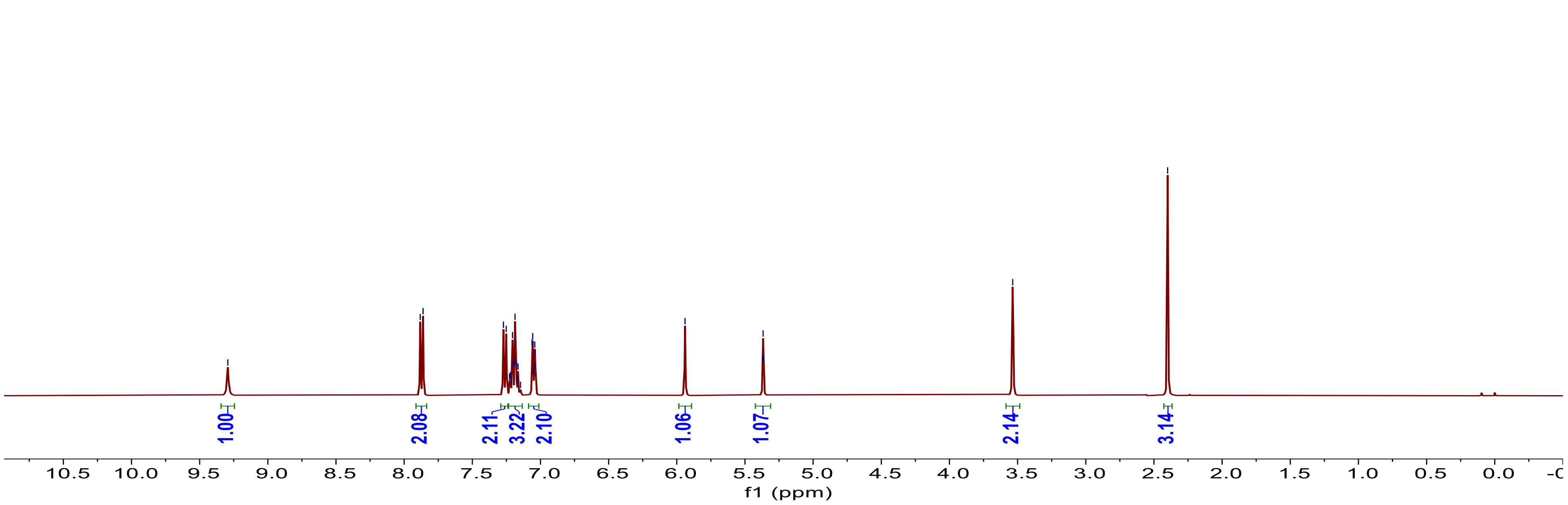

^{1}H NMR Spectrum of 5c at 25 ºC ($CDCl_3$)

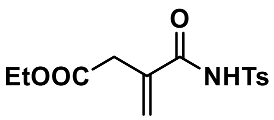

5c

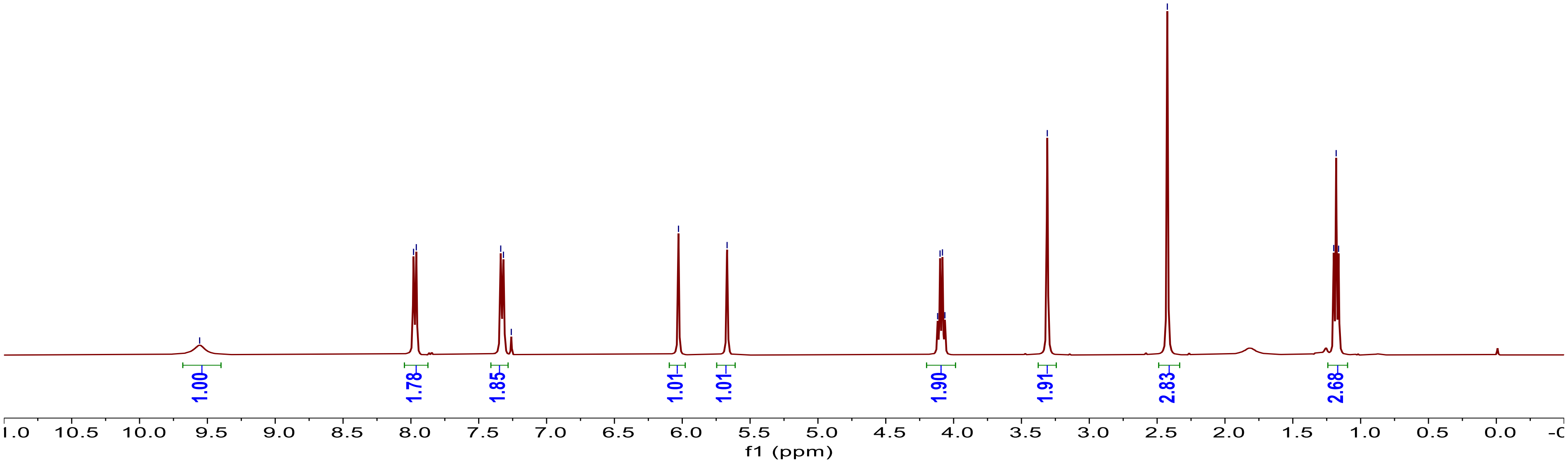

^{13}C NMR Spectrum of 5c at 25 ºC ($CDCl_3$)

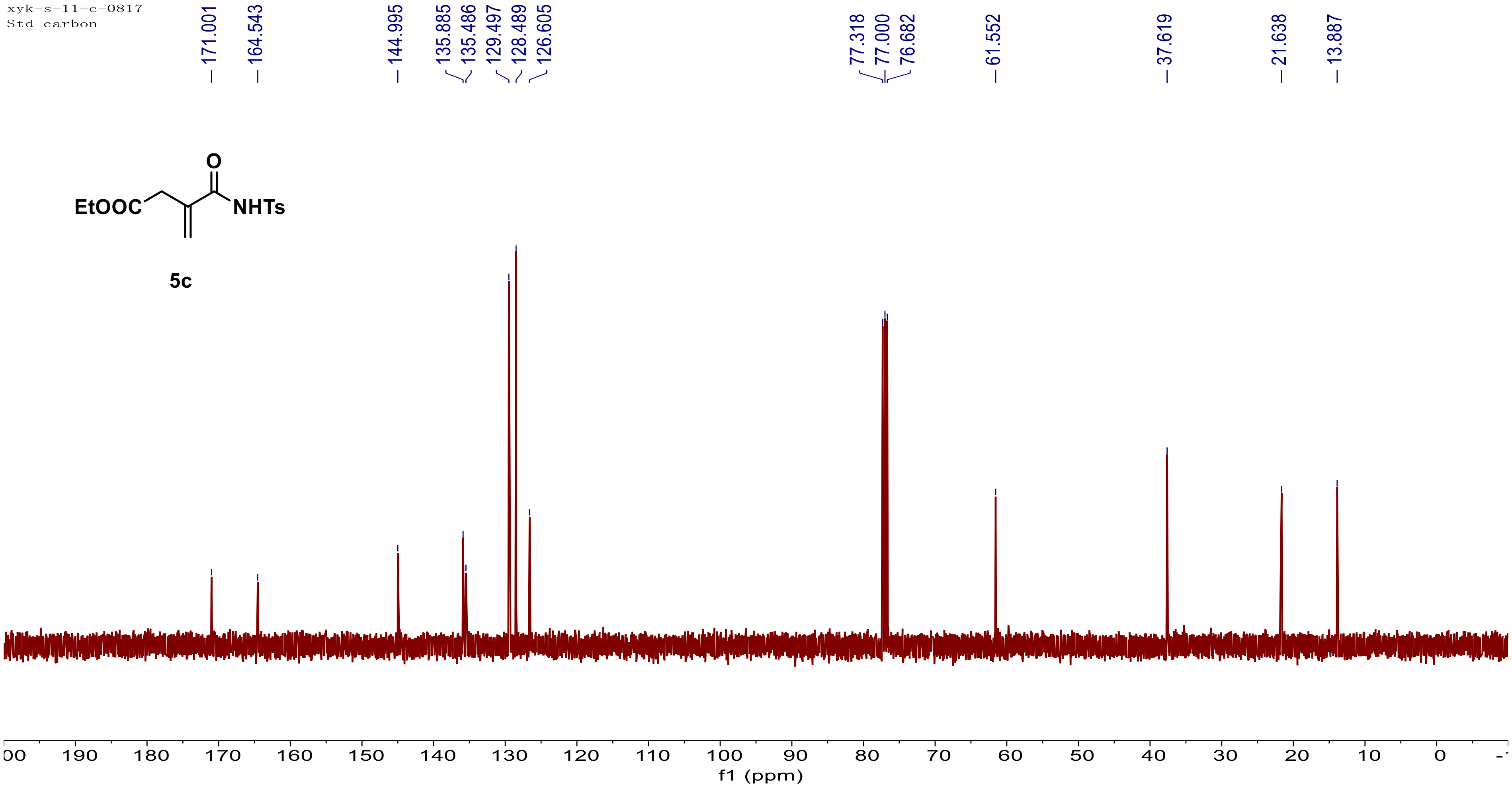

^{1}H NMR Spectrum of 5d at 25 °C ($CDCl_3$)

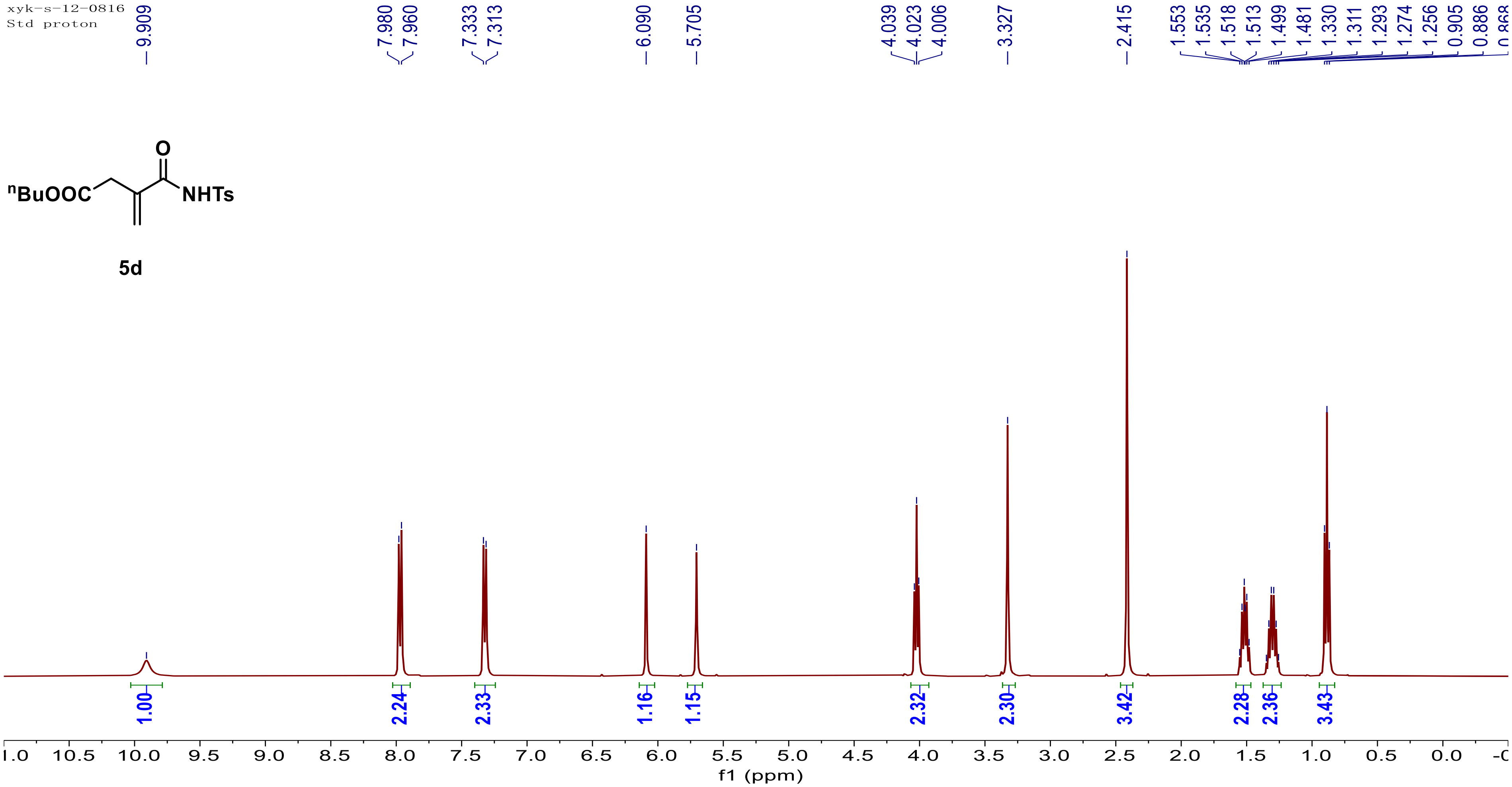

^{13}C NMR Spectrum of 5d at 25 ºC ($CDCl_3$)

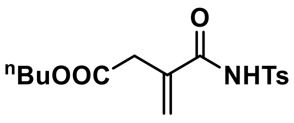

5d

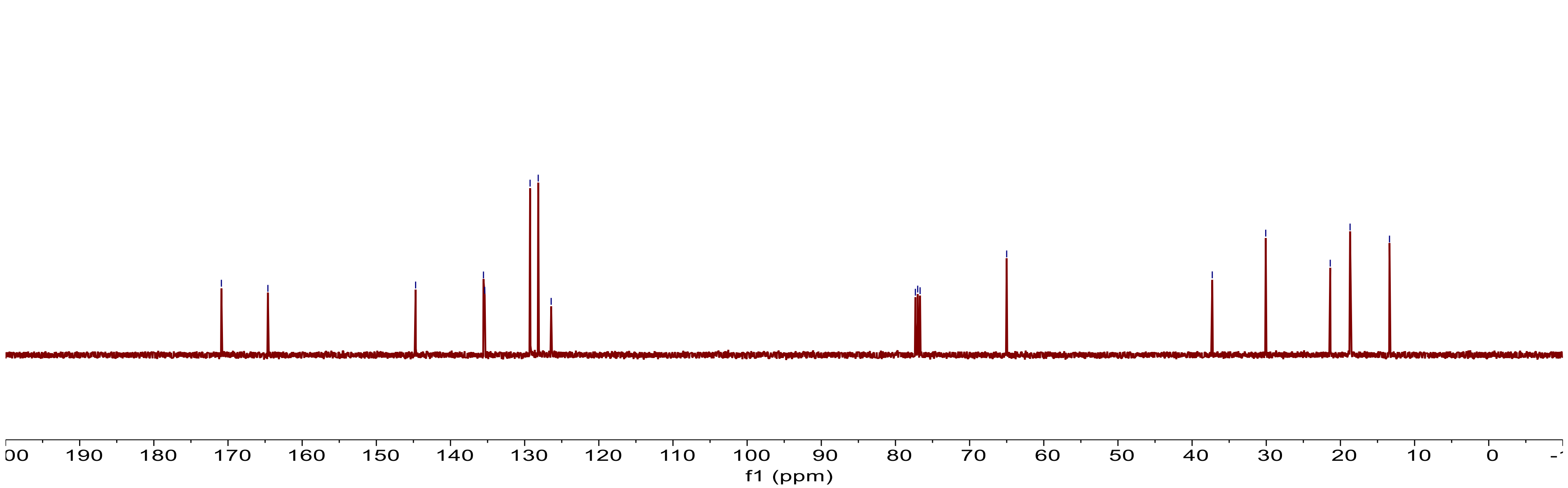

^{1}H NMR Spectrum of 5e at 25 ºC (CDCl₃)

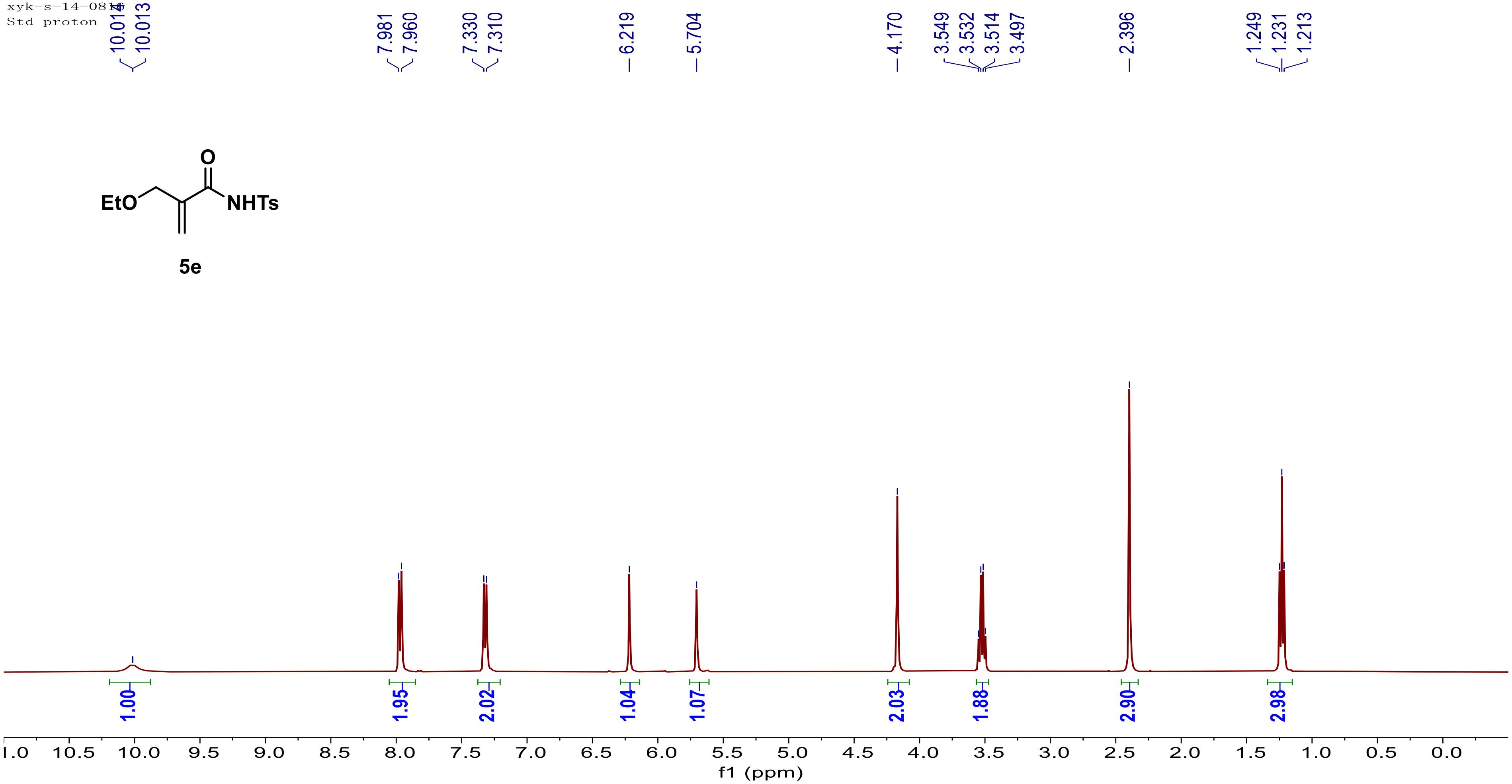

^{1}H NMR Spectrum of 5f at 25 ºC ($CDCl_3$)

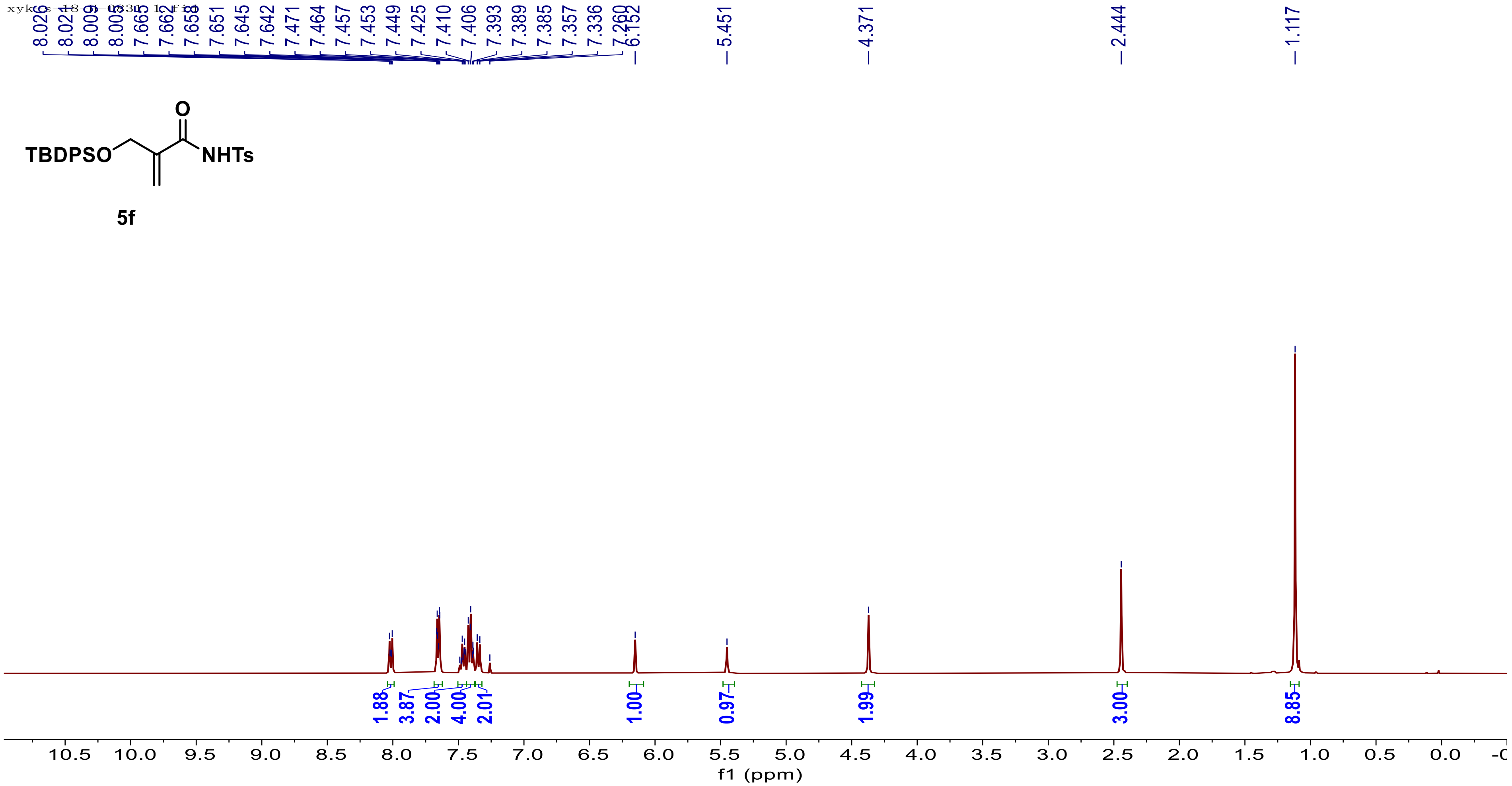

^{13}C NMR Spectrum of 5f at 25 ºC ($CDCl_3$)

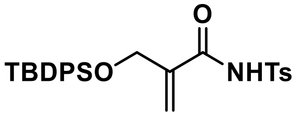

5f

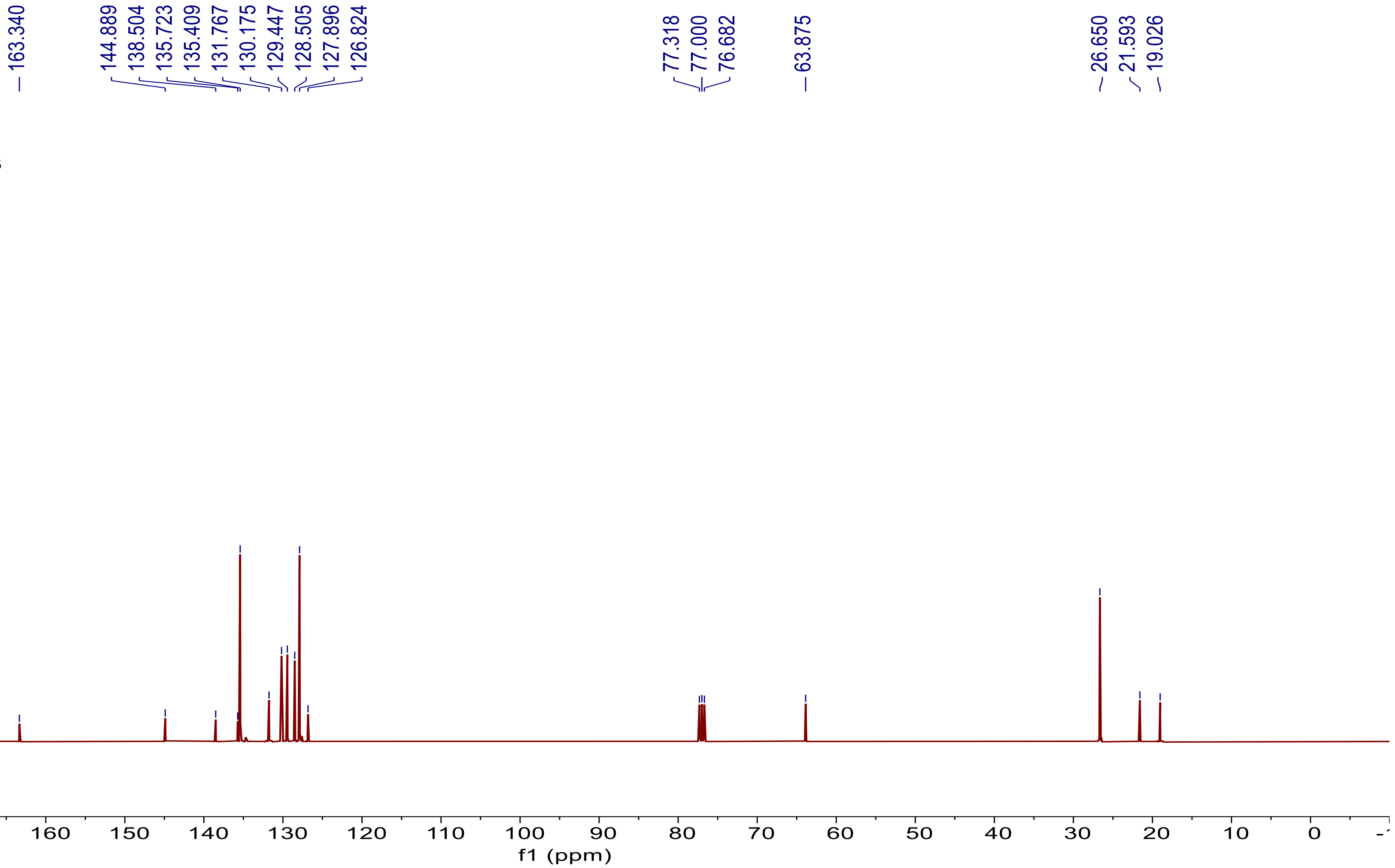

^{1}H NMR Spectrum of 5g at 25 °C ($CDCl_3$)

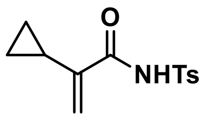

5g

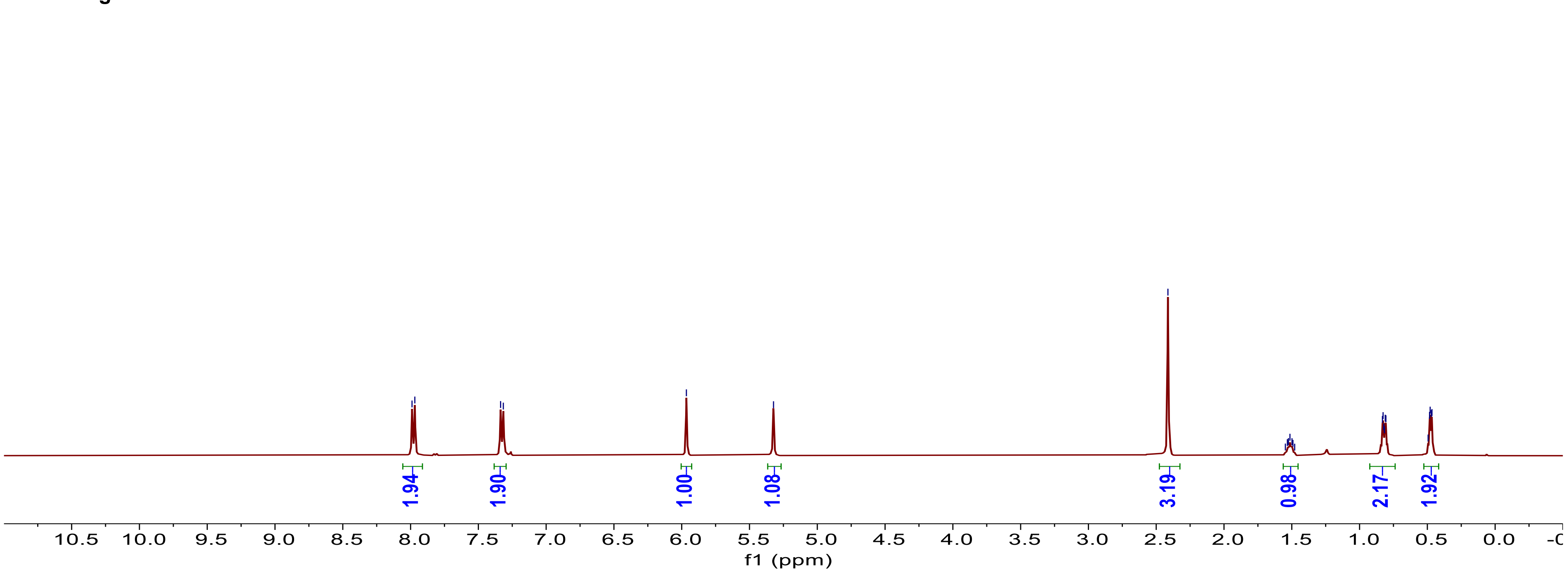

^{1}H NMR Spectrum of 5h at 25 ºC ($CDCl_3$)

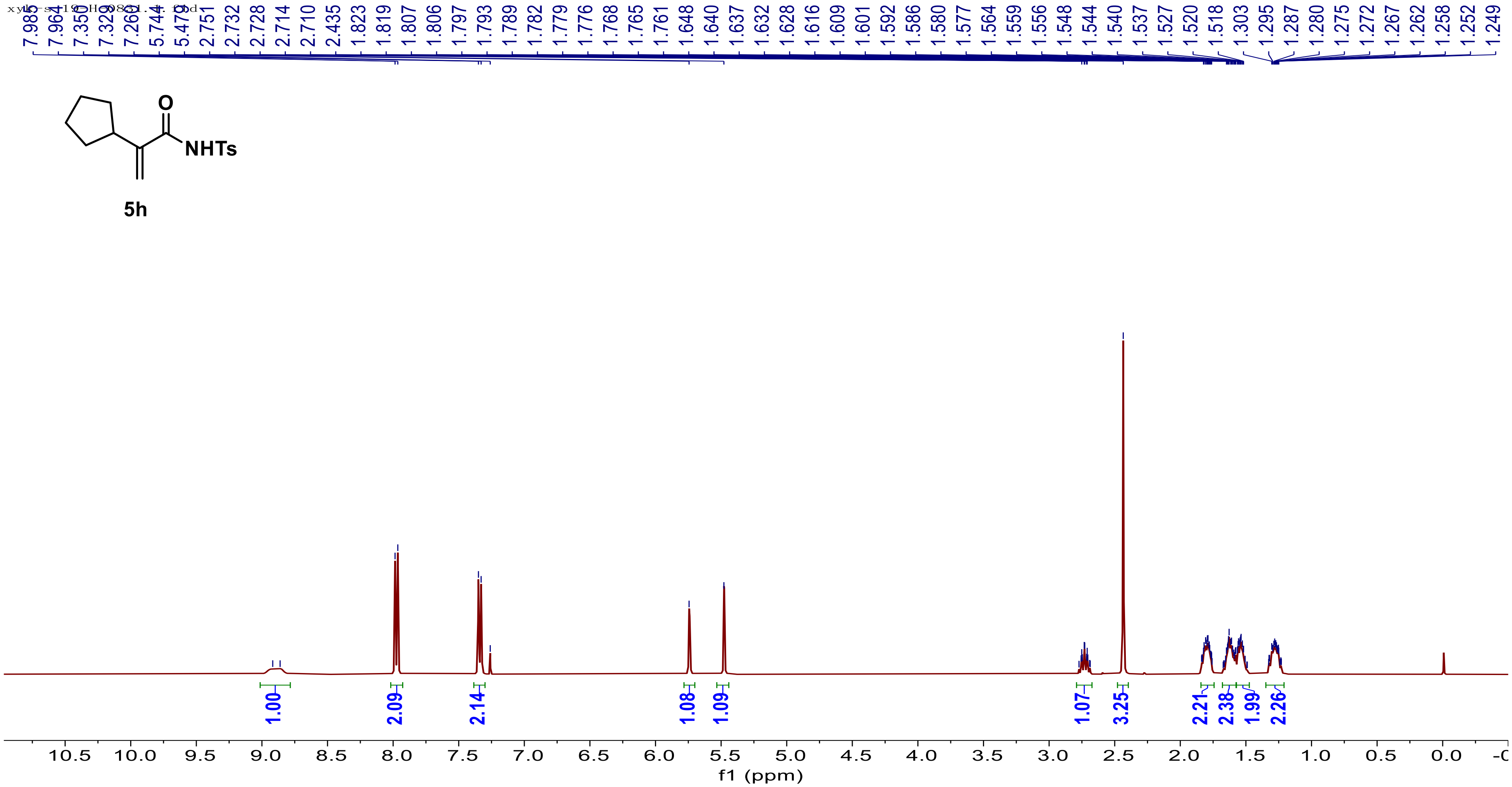

5h

^{13}C NMR Spectrum of 5h at 25 ºC ($CDCl_3$)

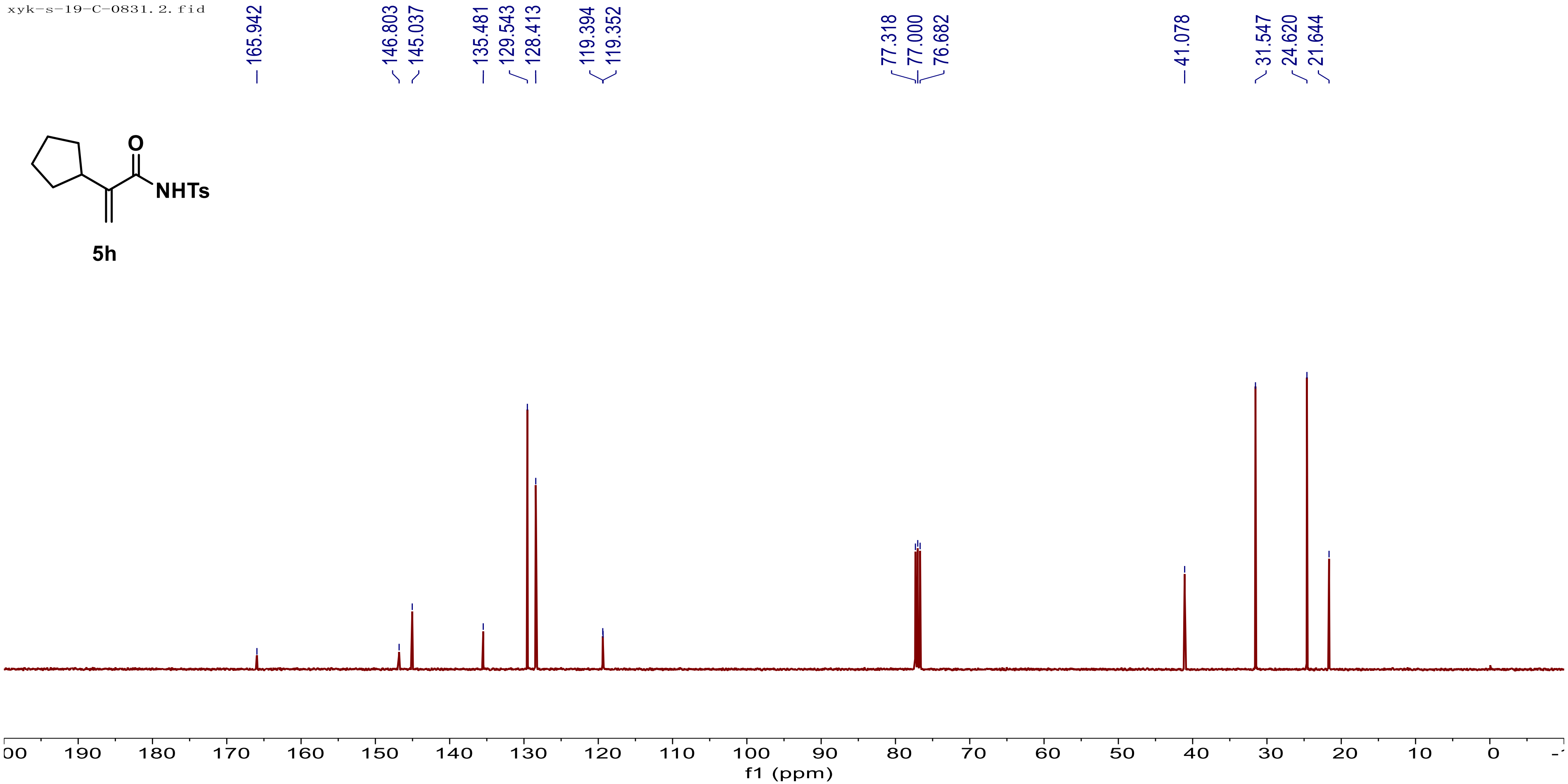

^{1}H NMR Spectrum of 5i at 25 °C ($CDCl_3$)

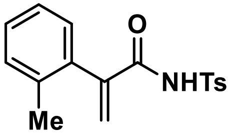

5i

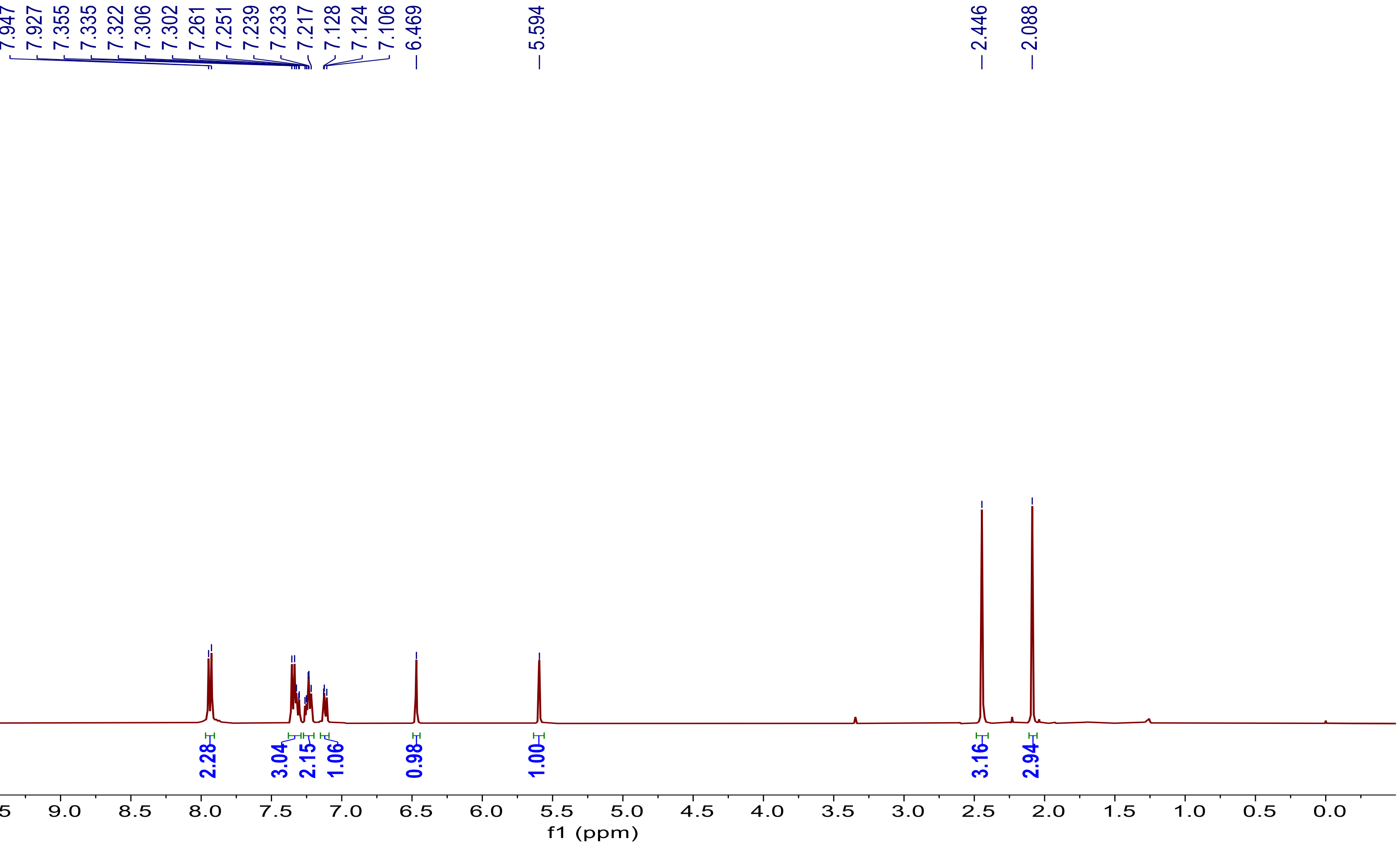

^{1}H NMR Spectrum of 5j at 25 ºC (CDCl$_3$)

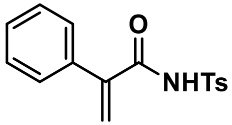

5j

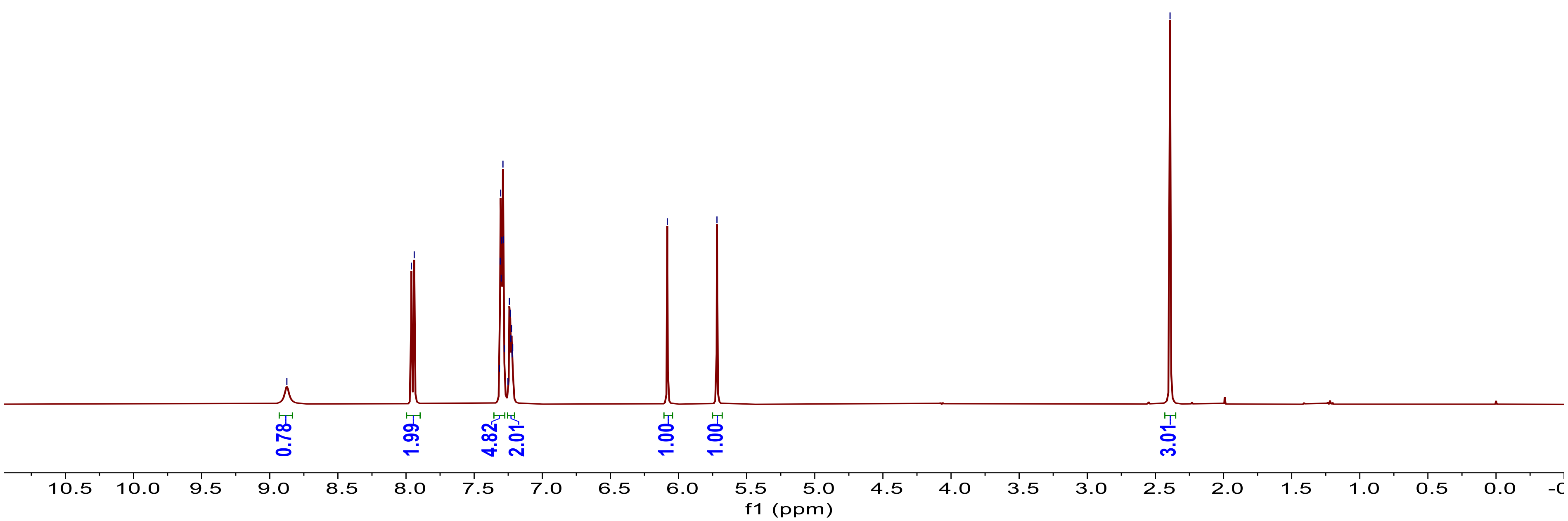

^{1}H NMR Spectrum of 5k at 25 ºC ($CDCl_3$)

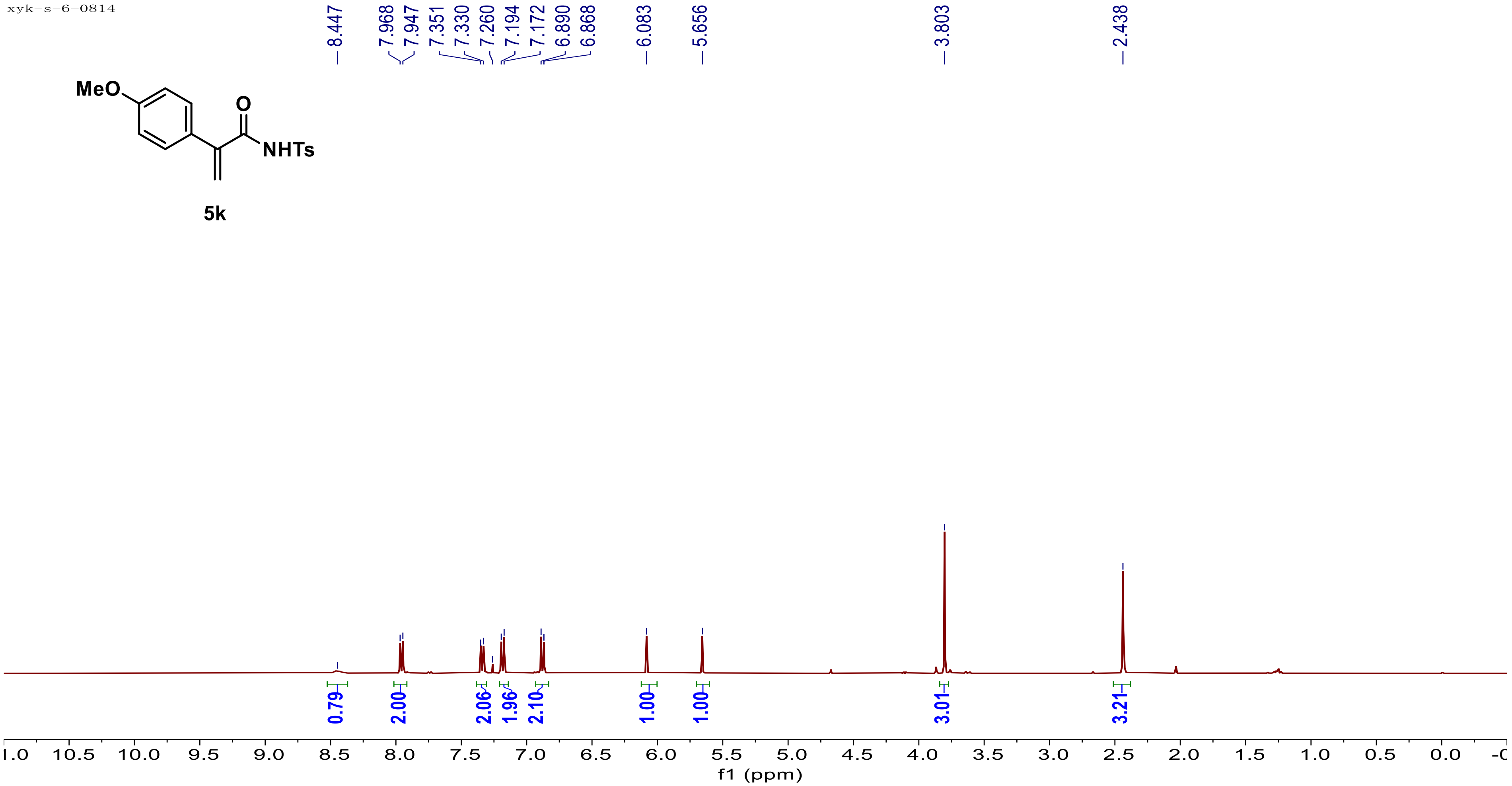

^{1}H NMR Spectrum of 5l at 25 ºC ($CDCl_3$)

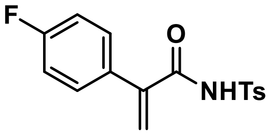

5l

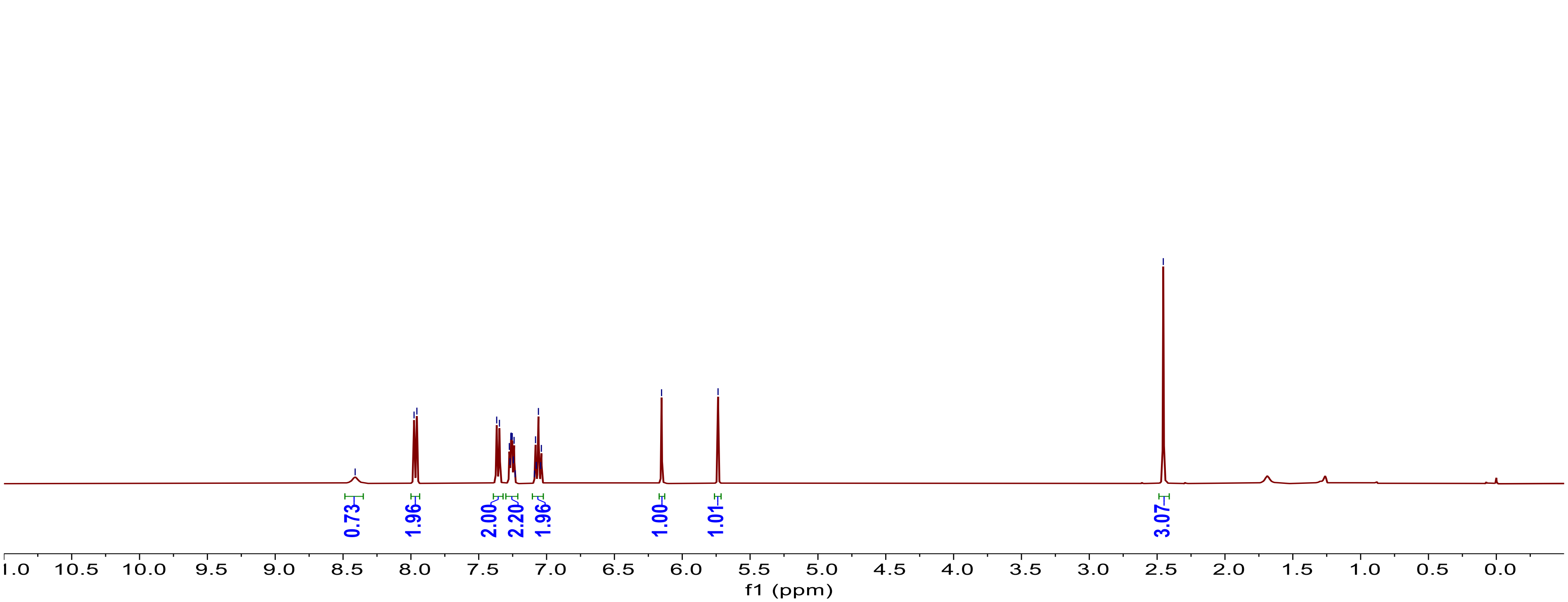

^{1}H NMR Spectrum of 5m at 25 ºC (CDCl$_3$)

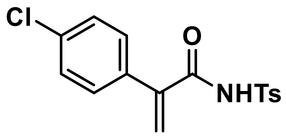

5m

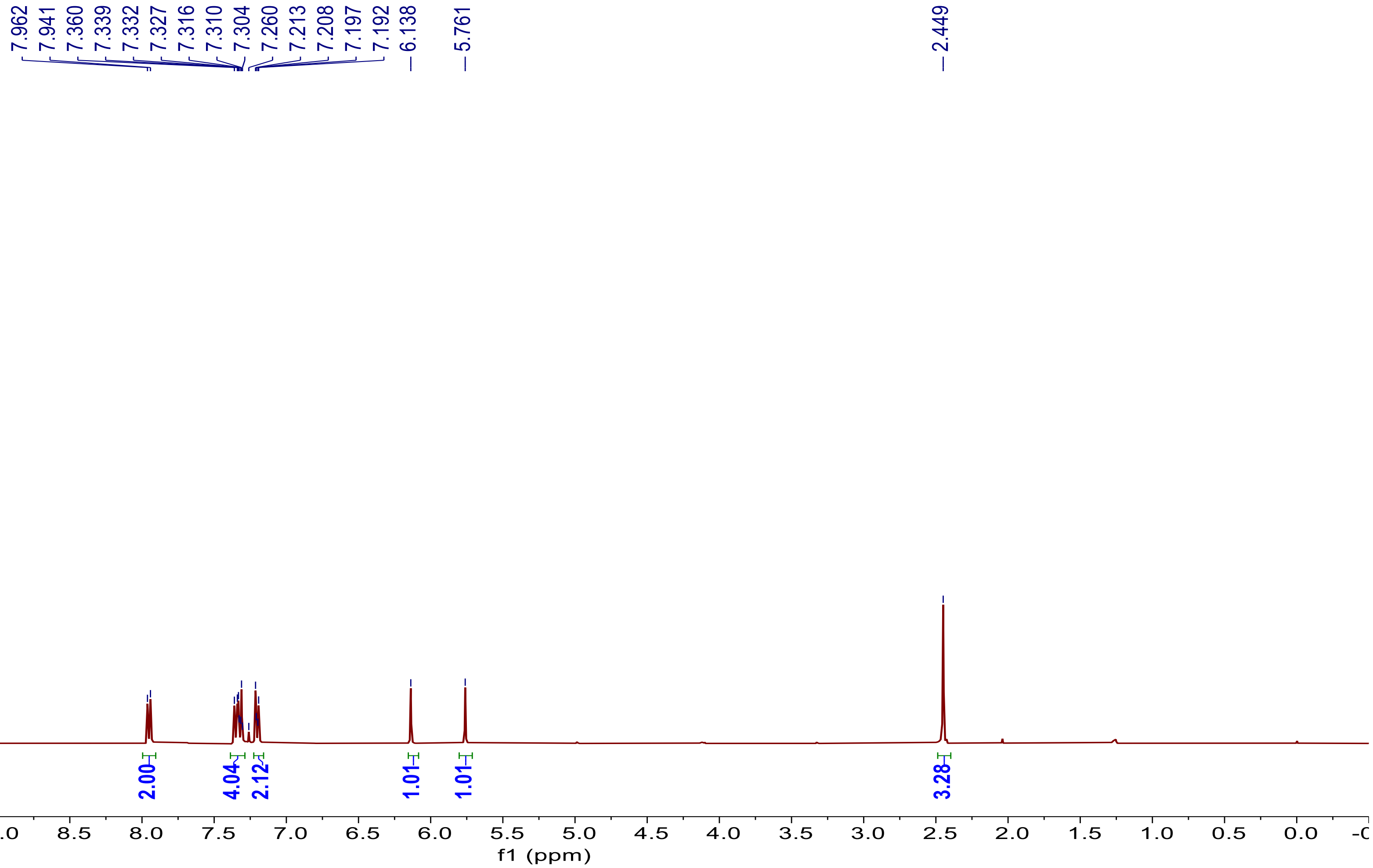

¹H NMR Spectrum of 5n at 25 °C ($CDCl_3$)

xyk-s-10-0814
Std proton

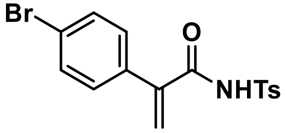

5n

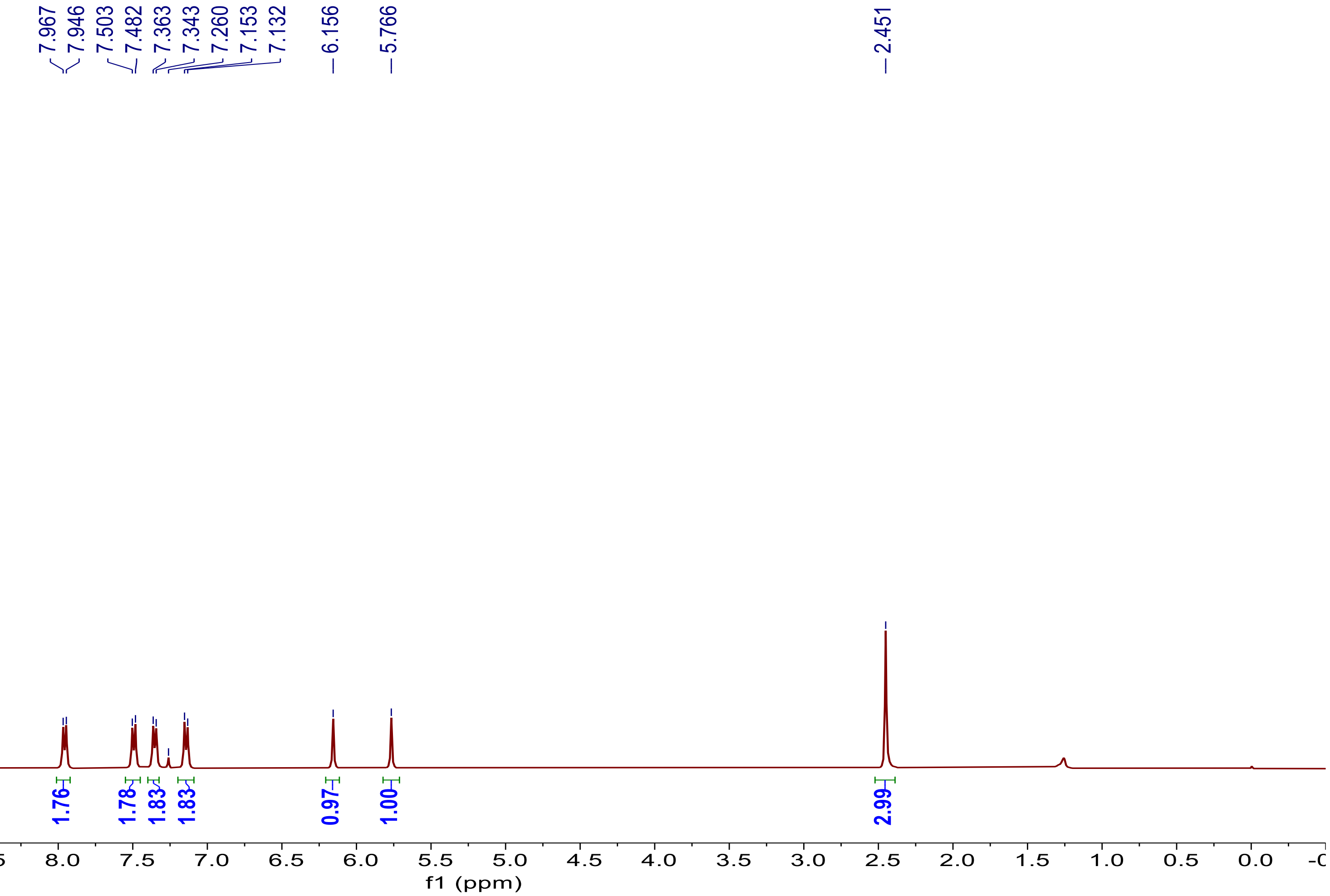

^{1}H NMR Spectrum of 5o at 25 ºC ($CDCl_3$)

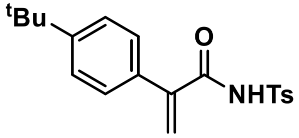

5o

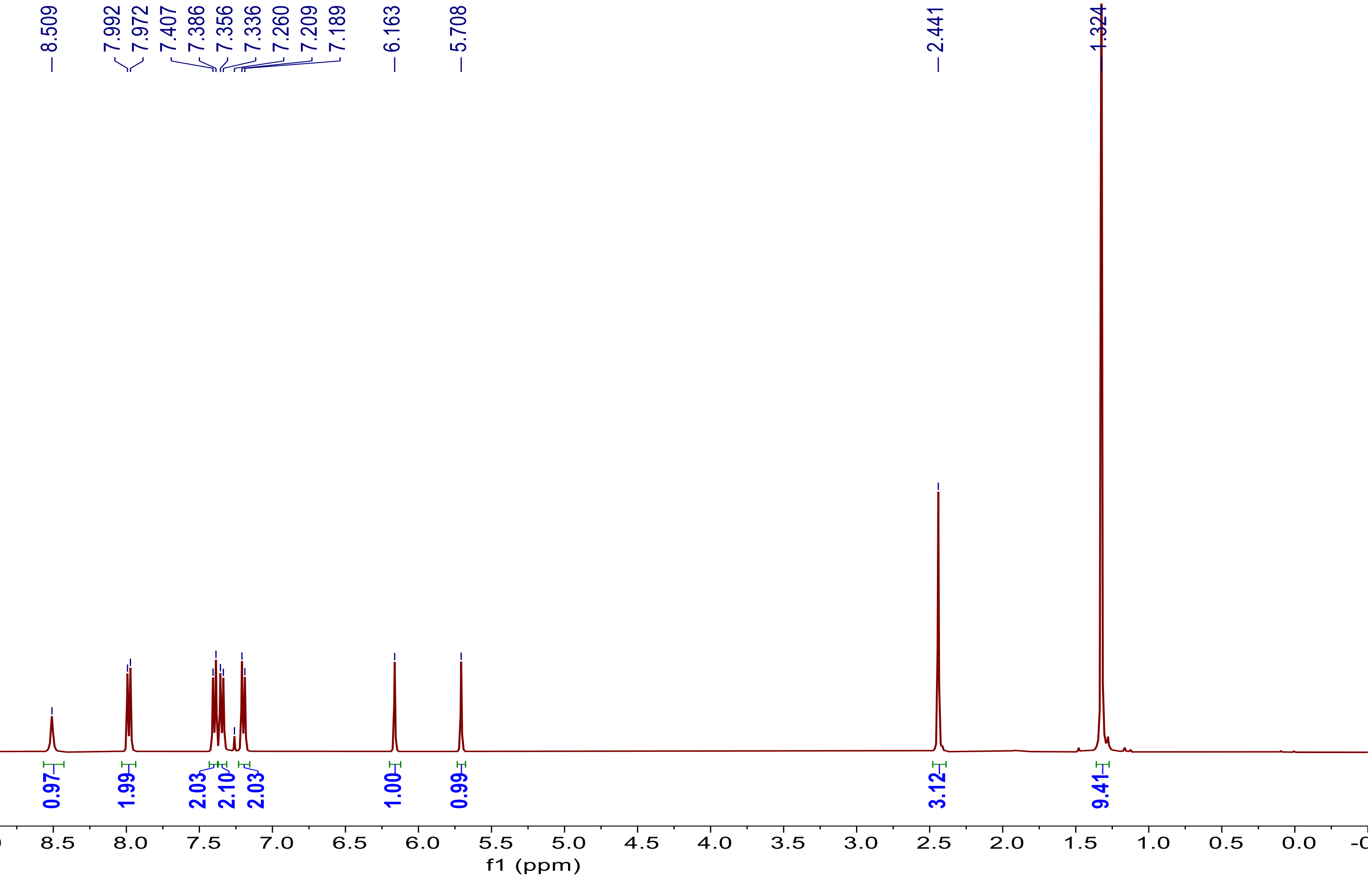

^{1}H NMR Spectrum of 5p at 25 ºC ($CDCl_3$)

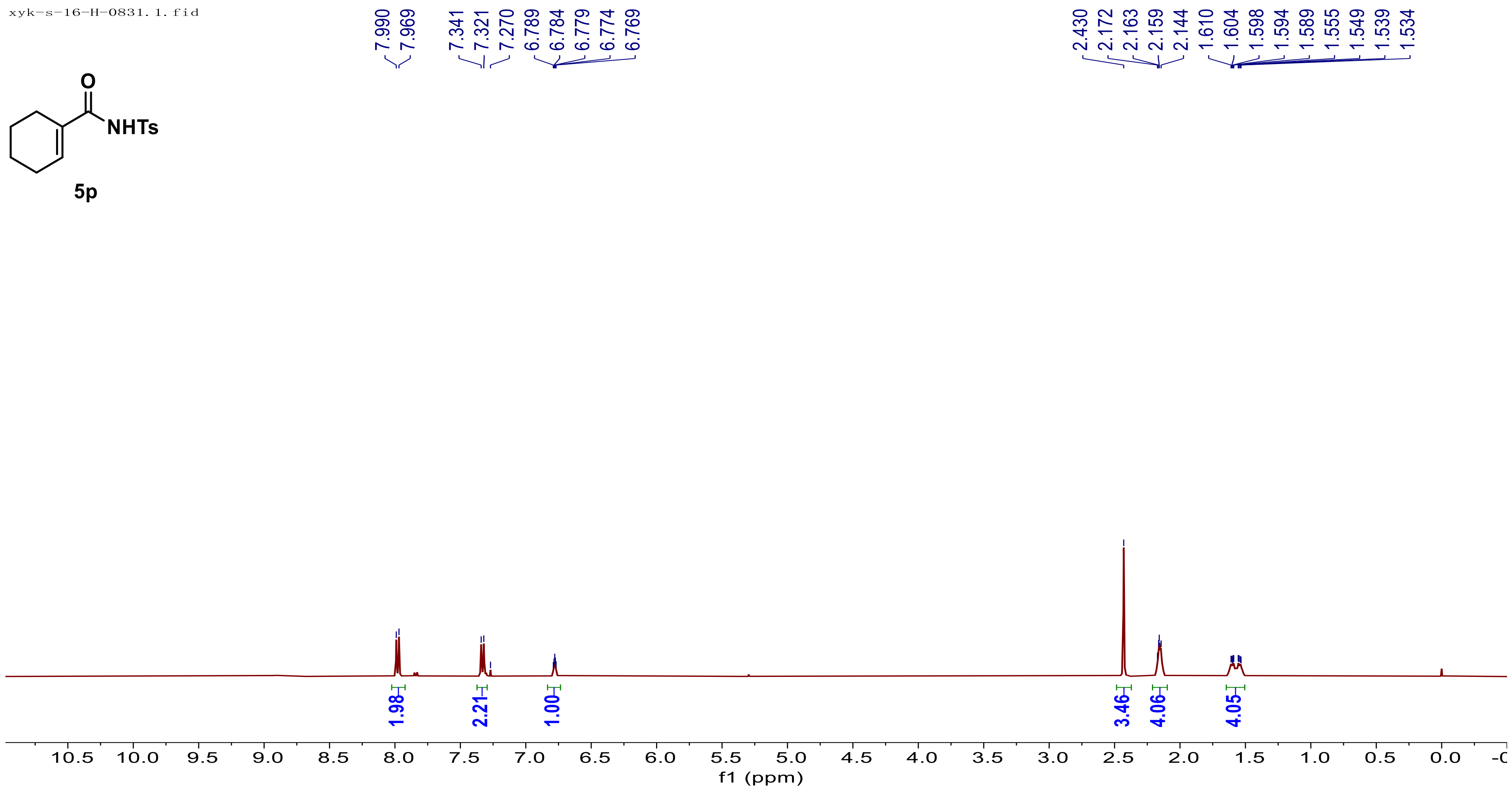

^{1}H NMR Spectrum of 5q at 25 ºC ($CDCl_3$)

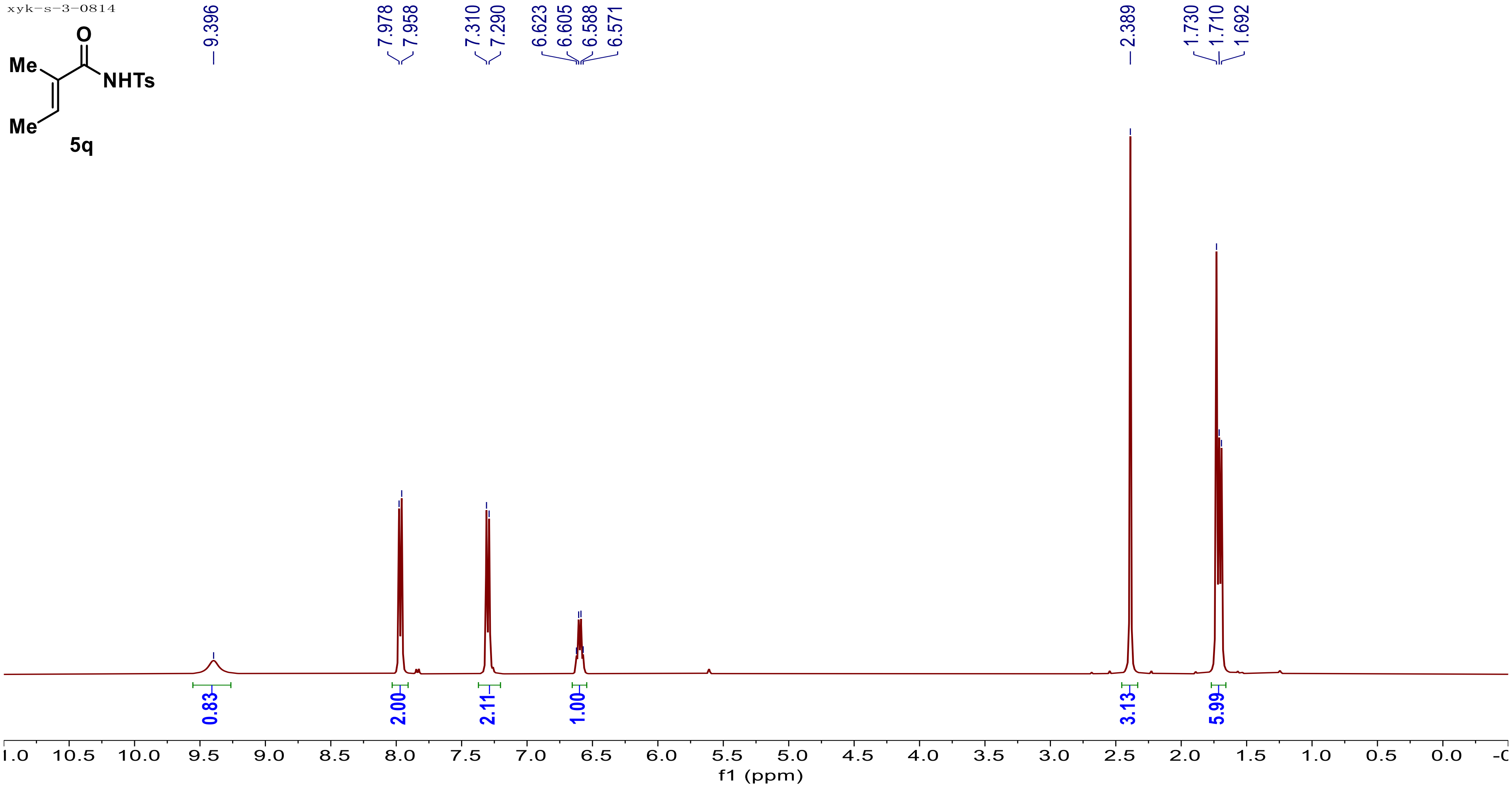

5q

^{1}H NMR Spectrum of 5r at 25 ºC ($CDCl_3$)

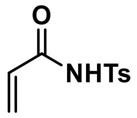

5r

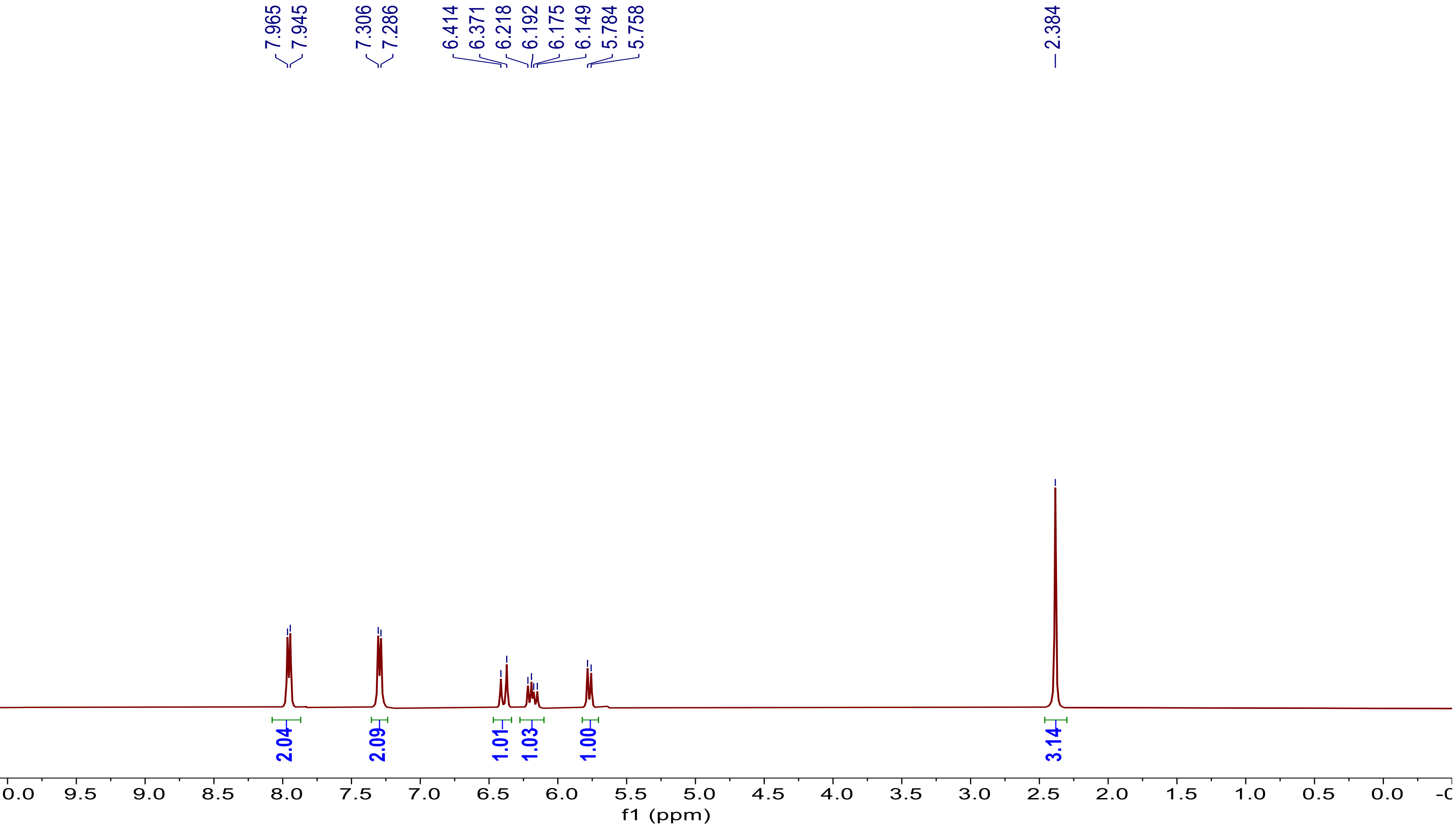

^{1}H NMR Spectrum of 5s at 25 ºC ($CDCl_3$)

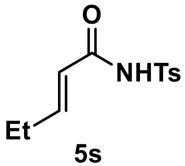

5s

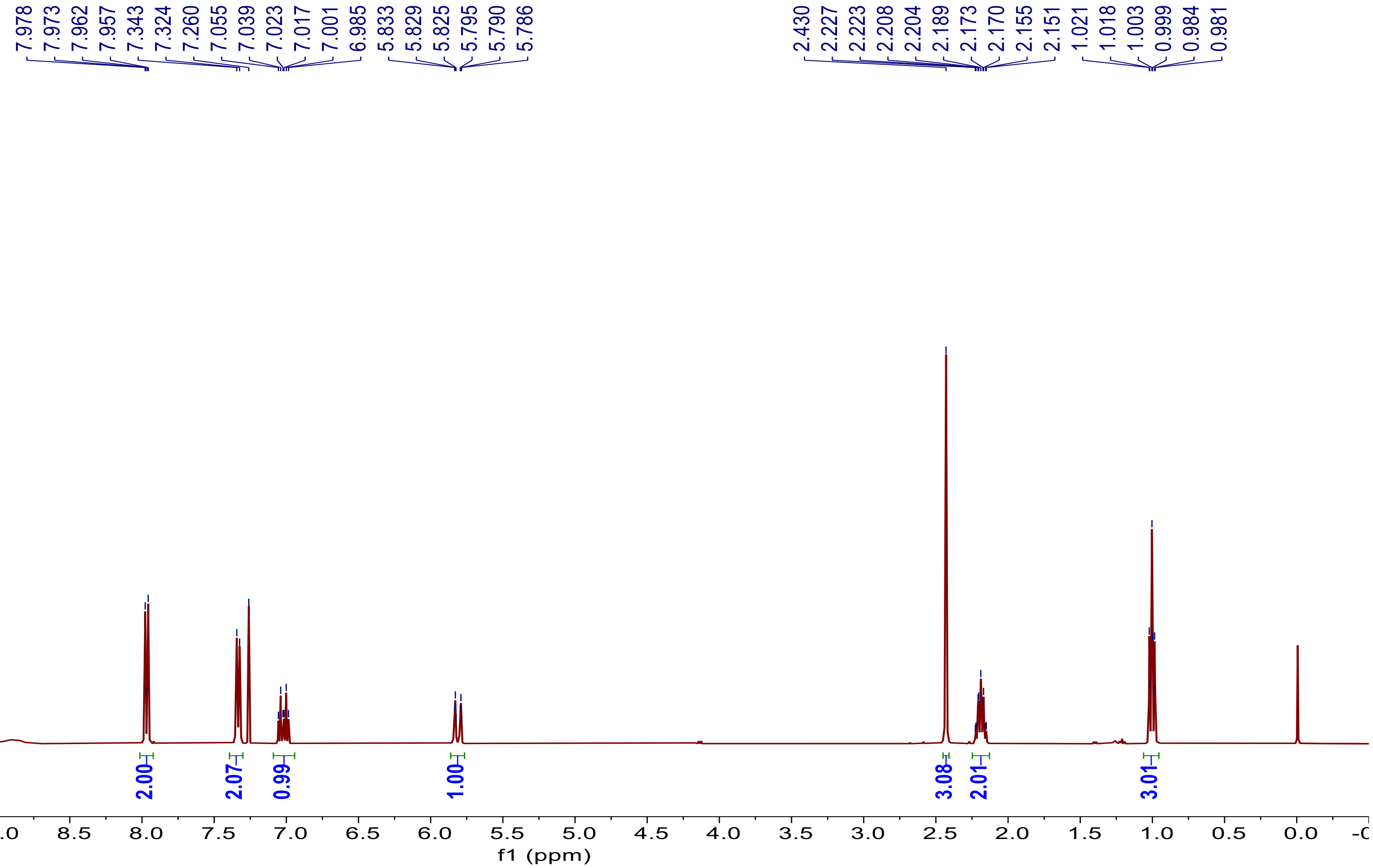

^{13}C NMR Spectrum of 5s at 25 ºC ($CDCl_3$)

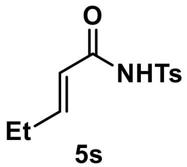

5s

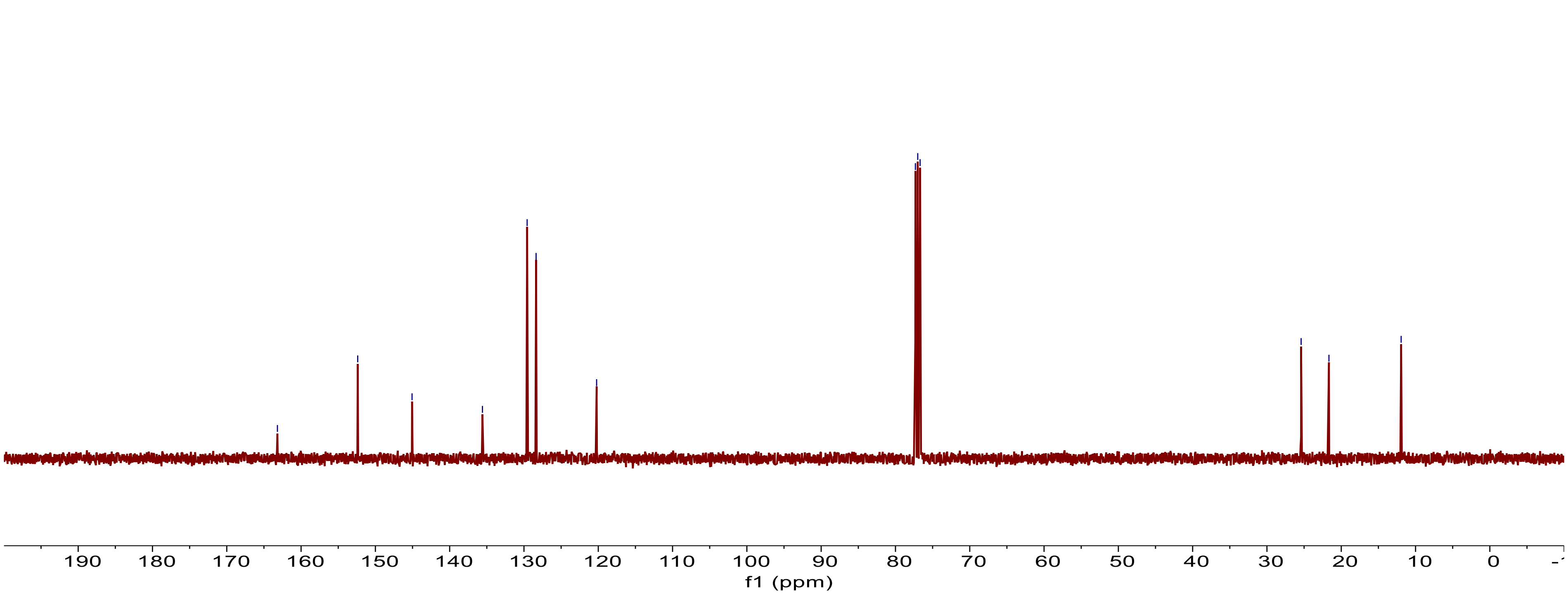

^{1}H NMR Spectrum of 5t at 25 ºC ($CDCl_3$)

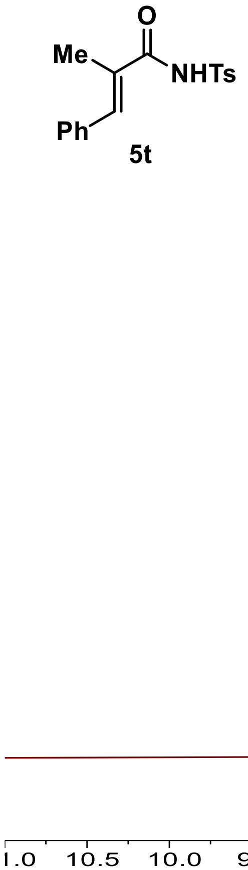

5t

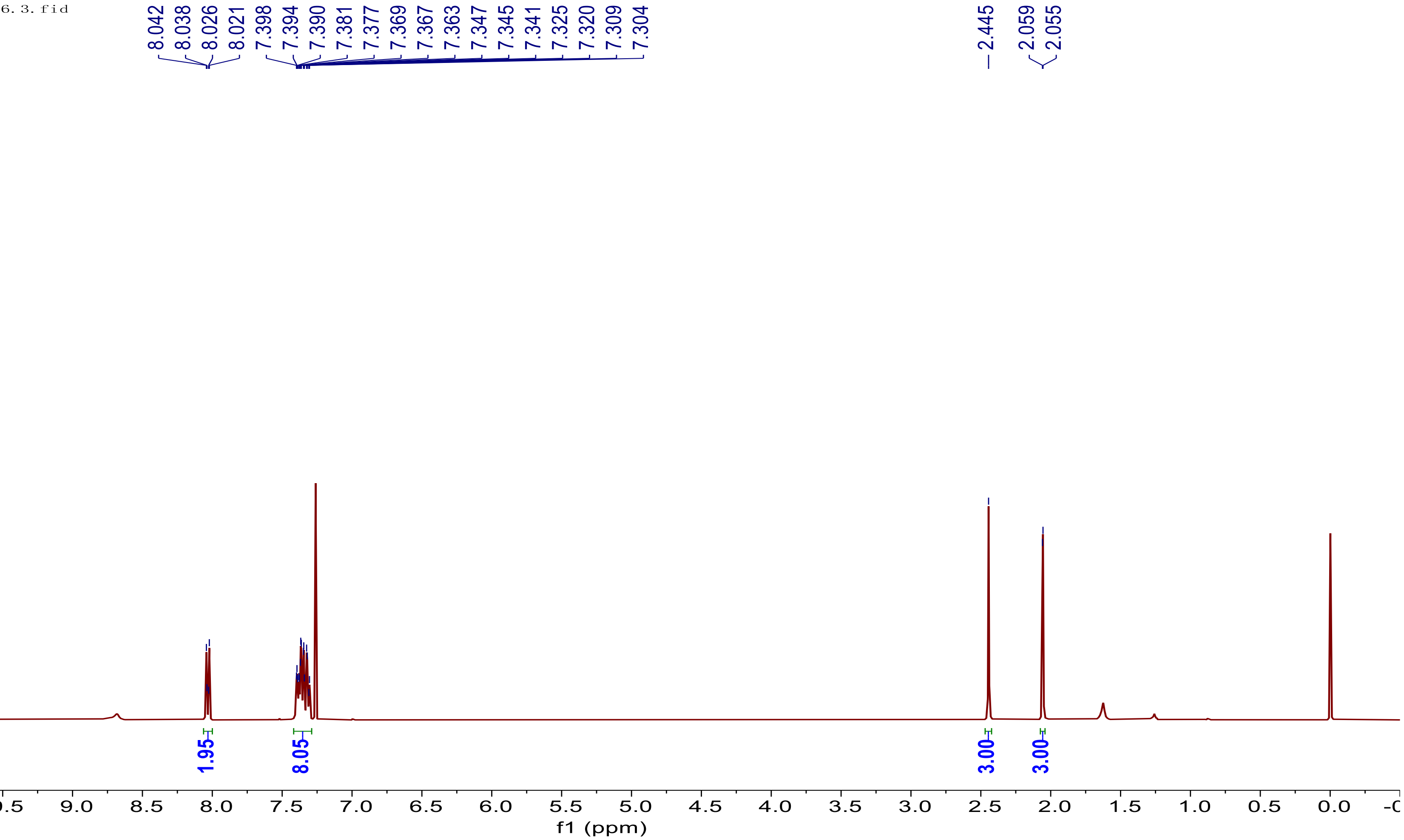

^{1}H NMR Spectrum of 5u at 25 °C ($CDCl_3$)

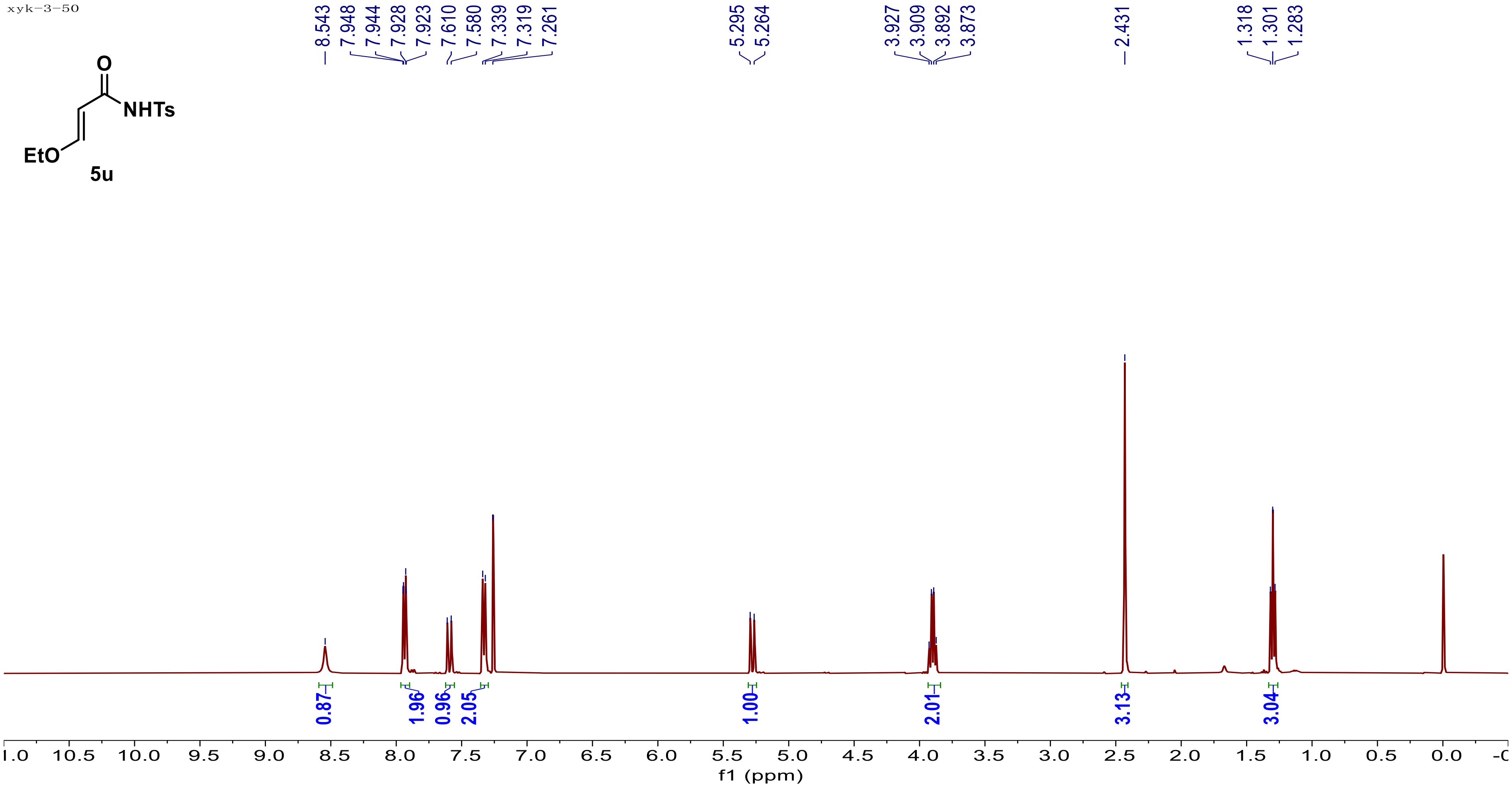

^{1}H NMR Spectrum of 6a at 25 °C ($CDCl_3$)

xyk-4-70-H-0626

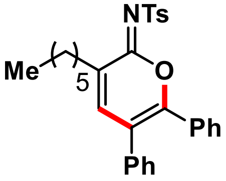

6a

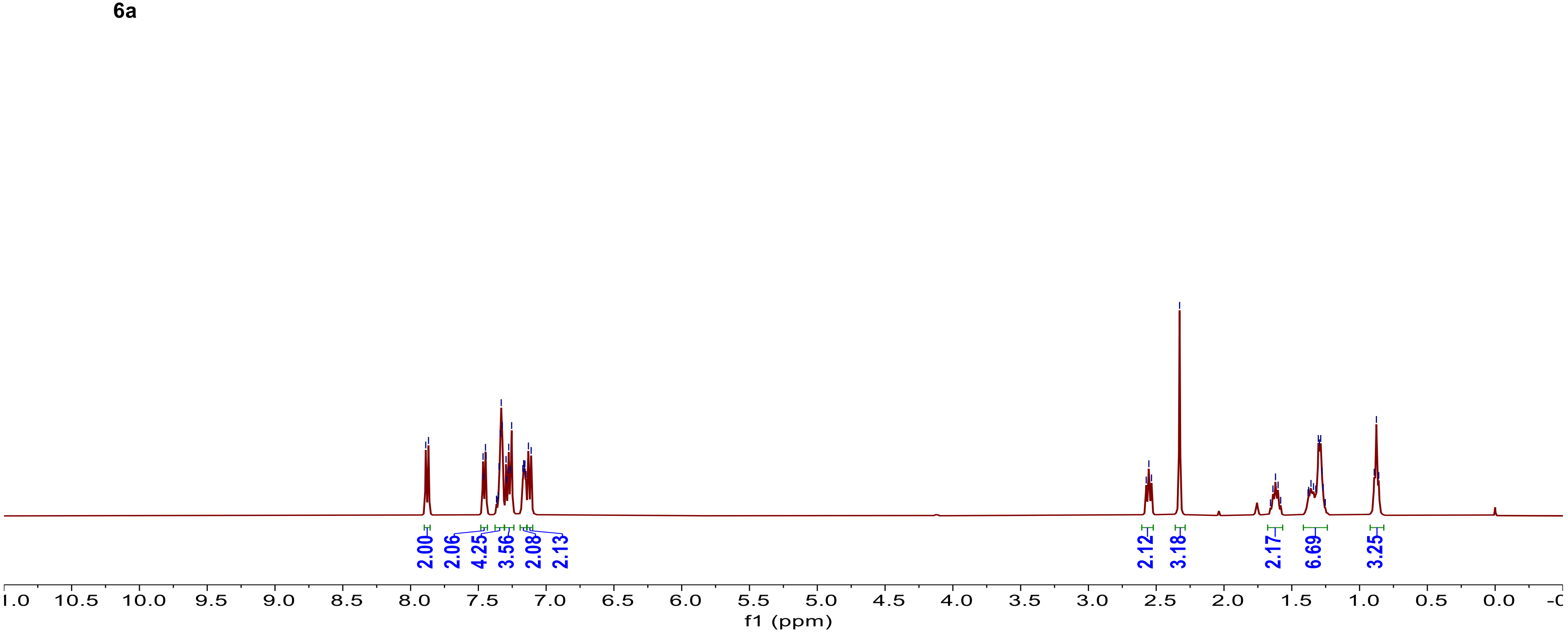

^{13}C NMR Spectrum of 6a at 25 ºC ($CDCl_3$)

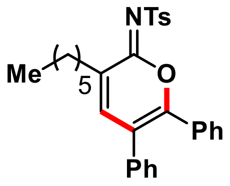

6a

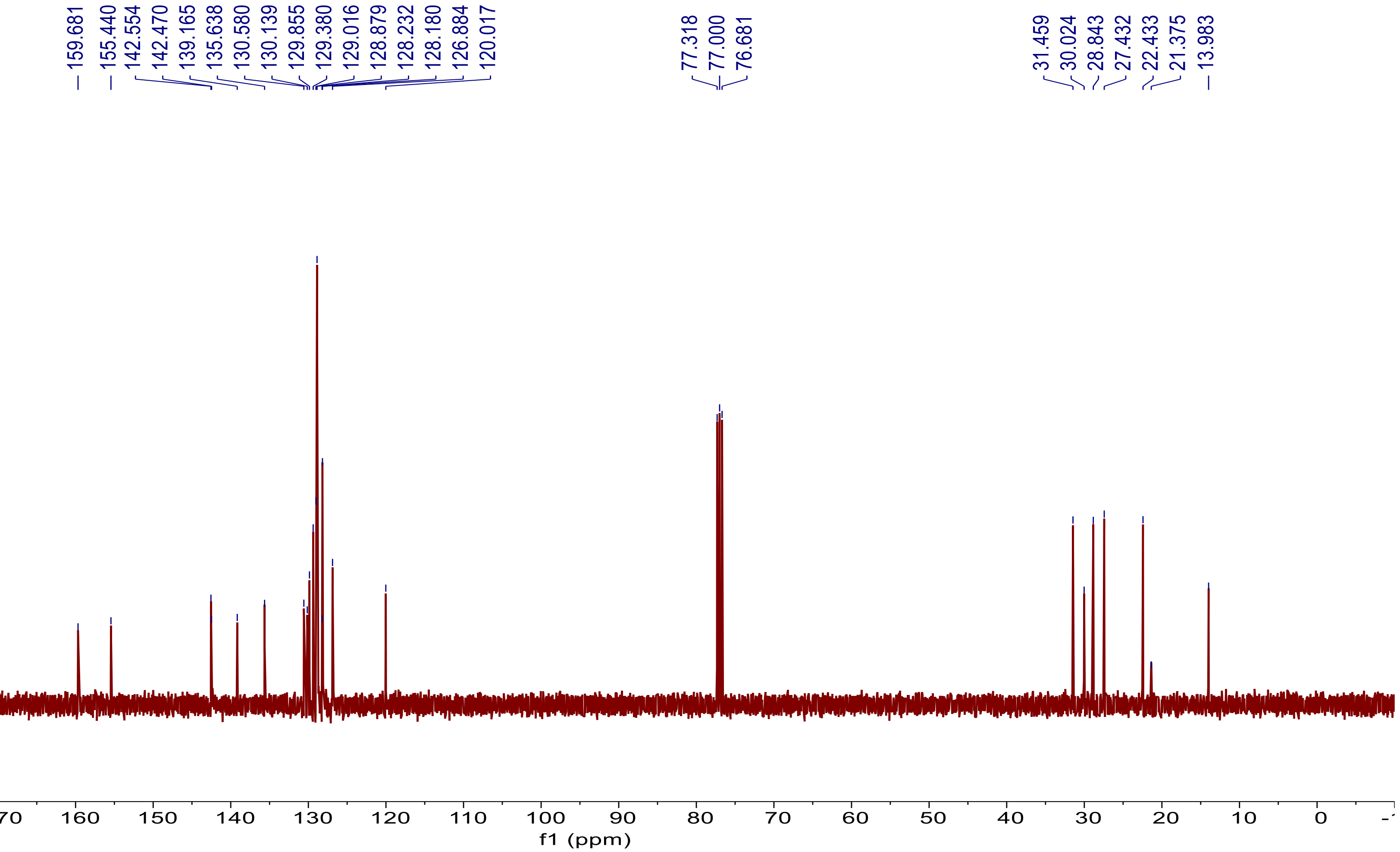

^{1}H NMR Spectrum of 6b at 25 ºC ($CDCl_3$)

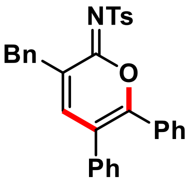

6b

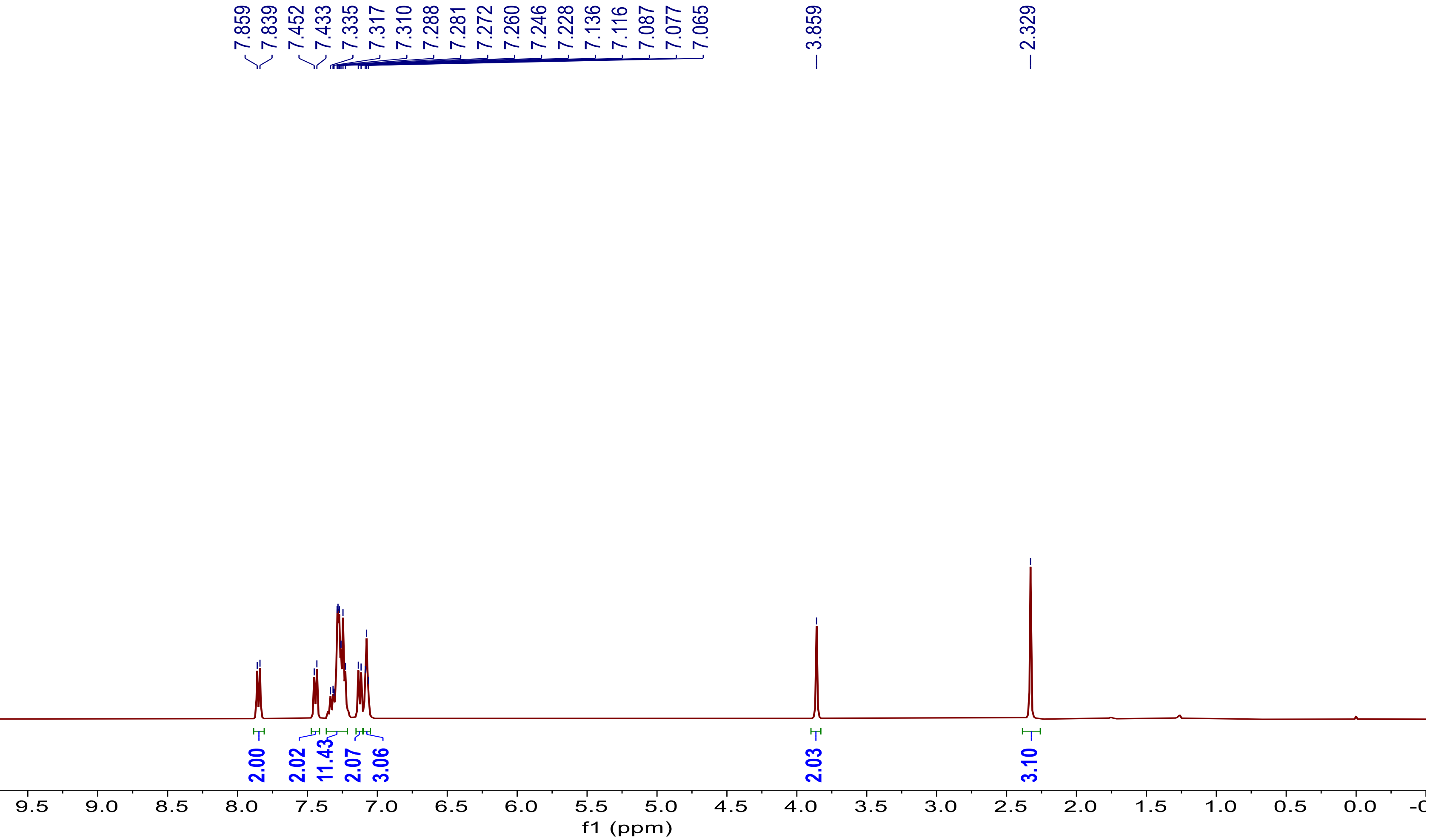

^{13}C NMR Spectrum of 6b at 25 ºC ($CDCl_3$)

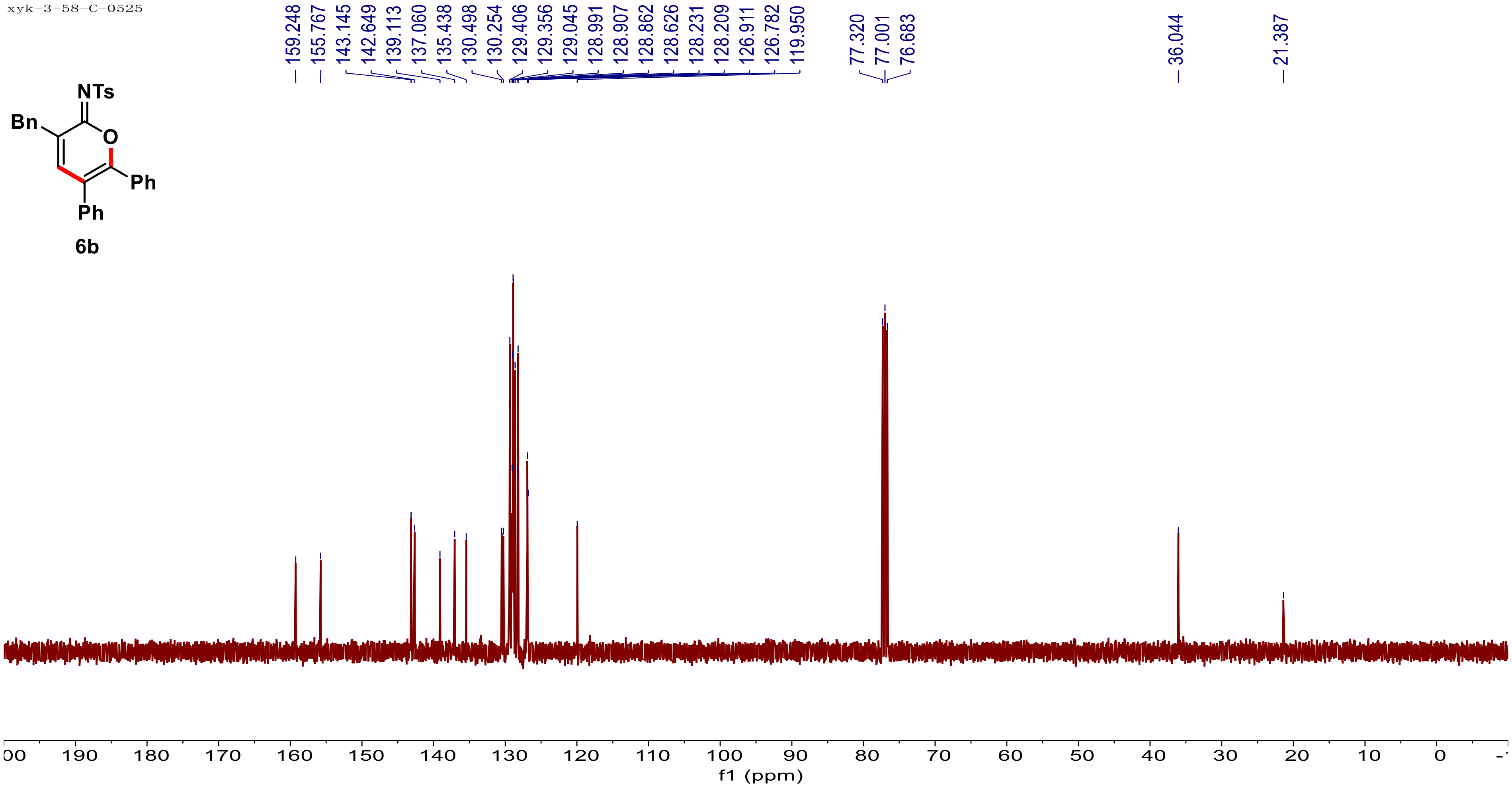

^{1}H NMR Spectrum of 6c at 25 ºC ($CDCl_3$)

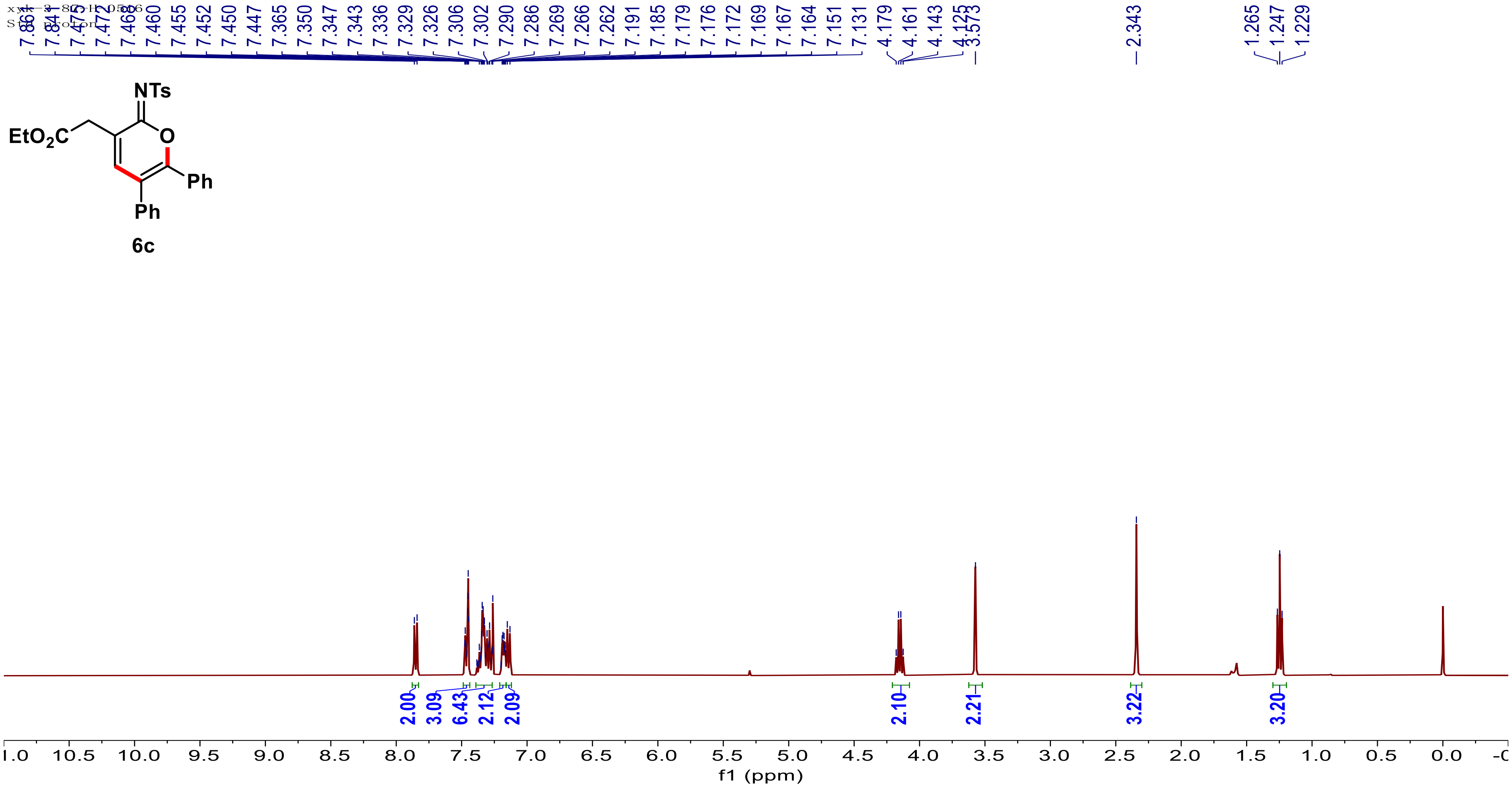

^{13}C NMR Spectrum of 6c at 25 ºC ($CDCl_3$)

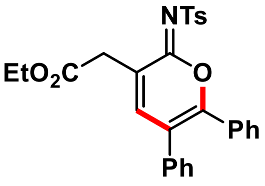

6c

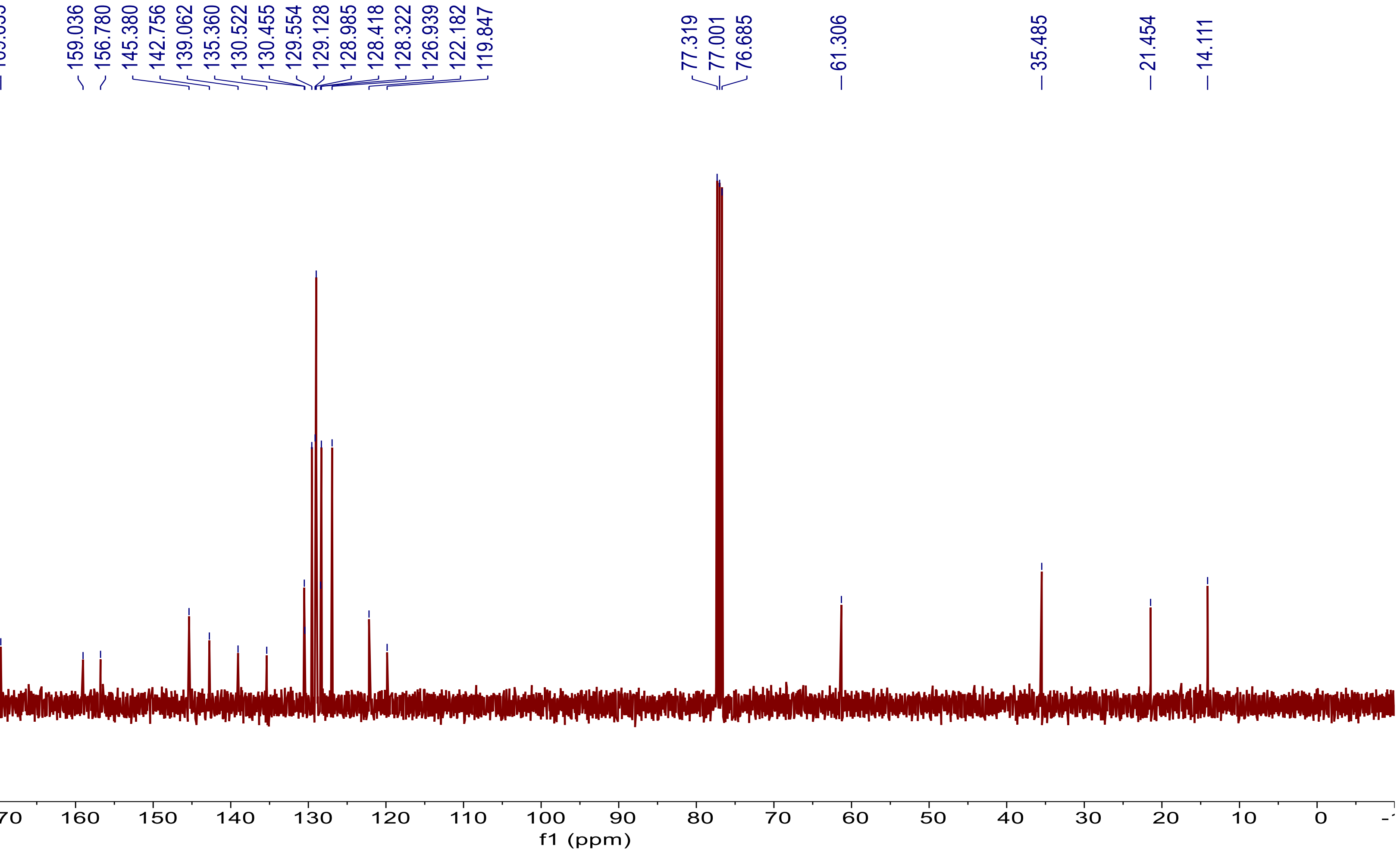

^{1}H NMR Spectrum of 6d at 25 °C ($CDCl_3$)

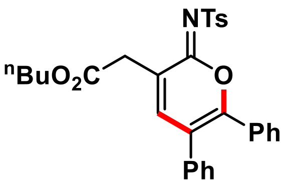

6d

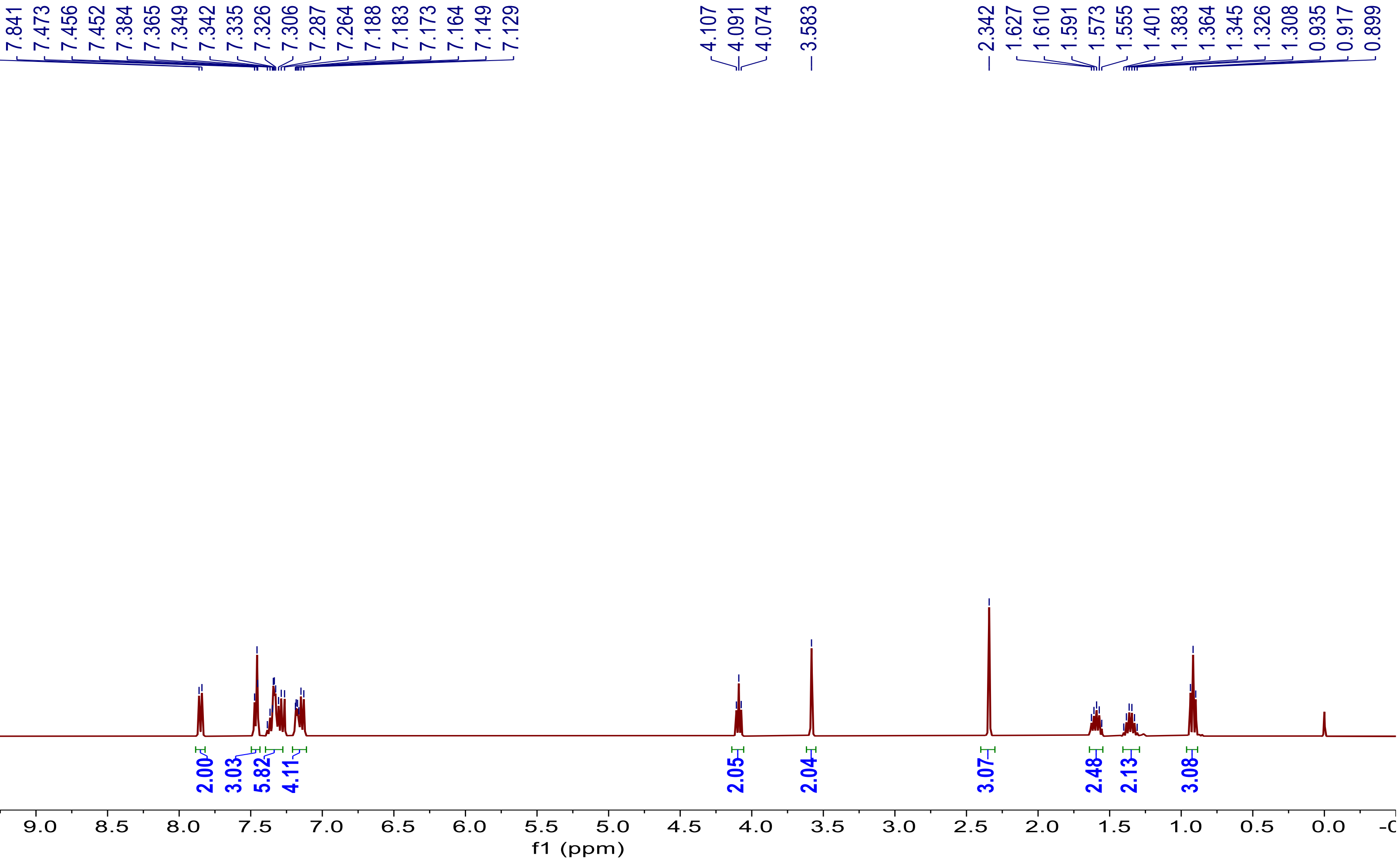

^{13}C NMR Spectrum of 6d at 25 °C ($CDCl_3$)

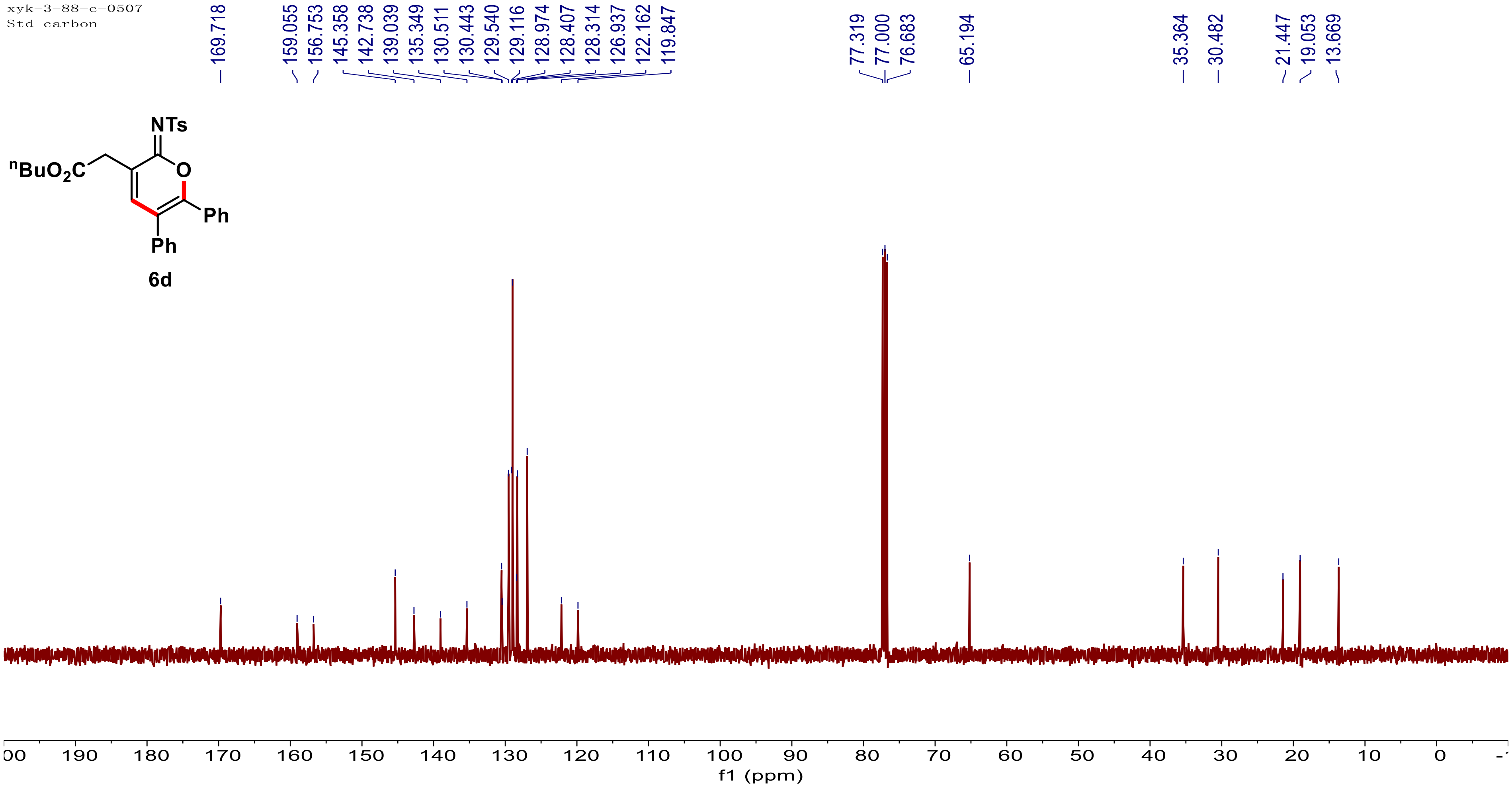

6d

^{1}H NMR Spectrum of 6e at 25 ºC ($CDCl_3$)

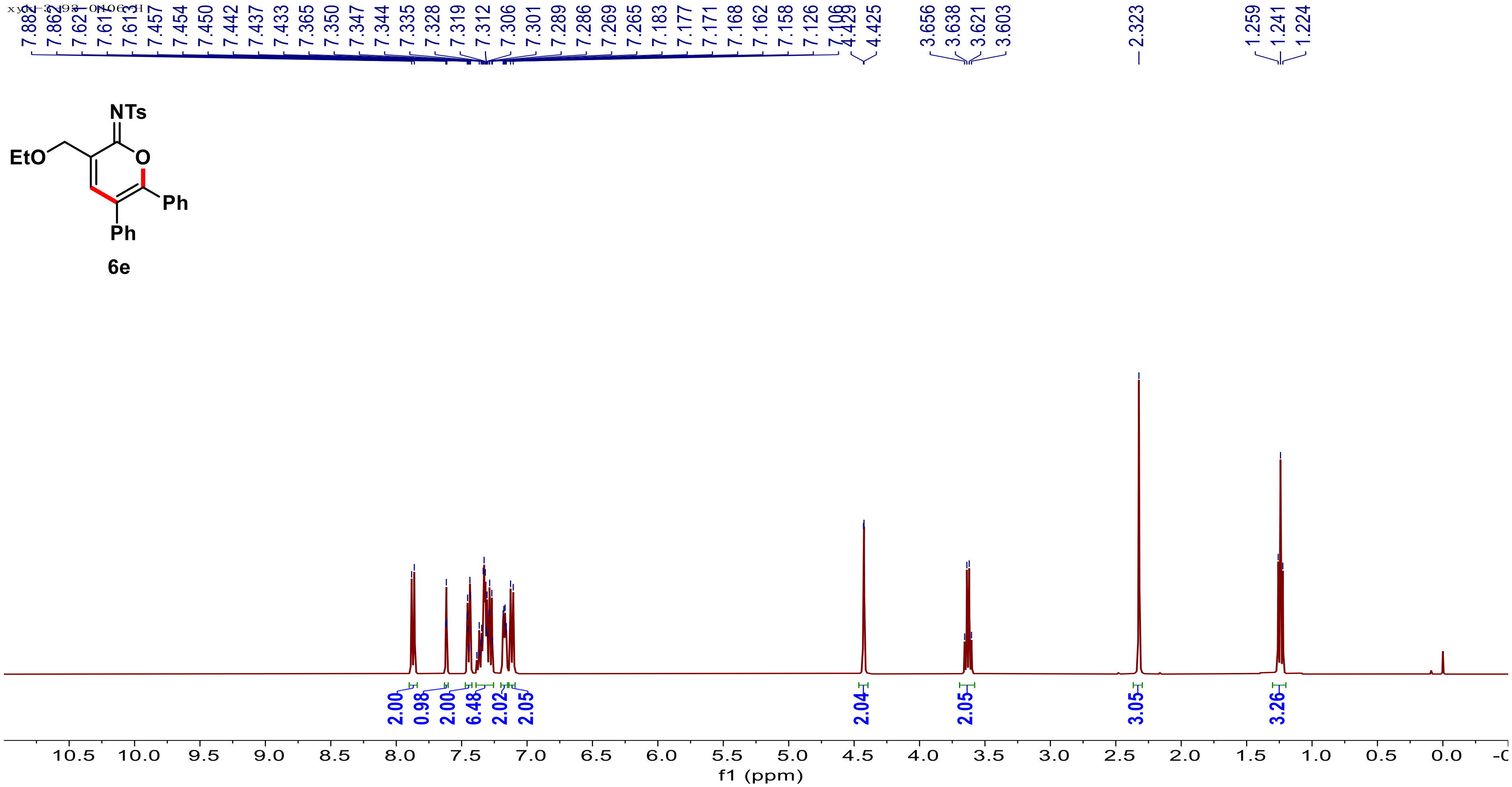

^{13}C NMR Spectrum of 6e at 25 ºC ($CDCl_3$)

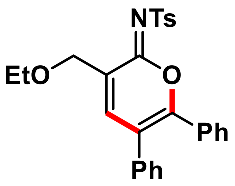

6e

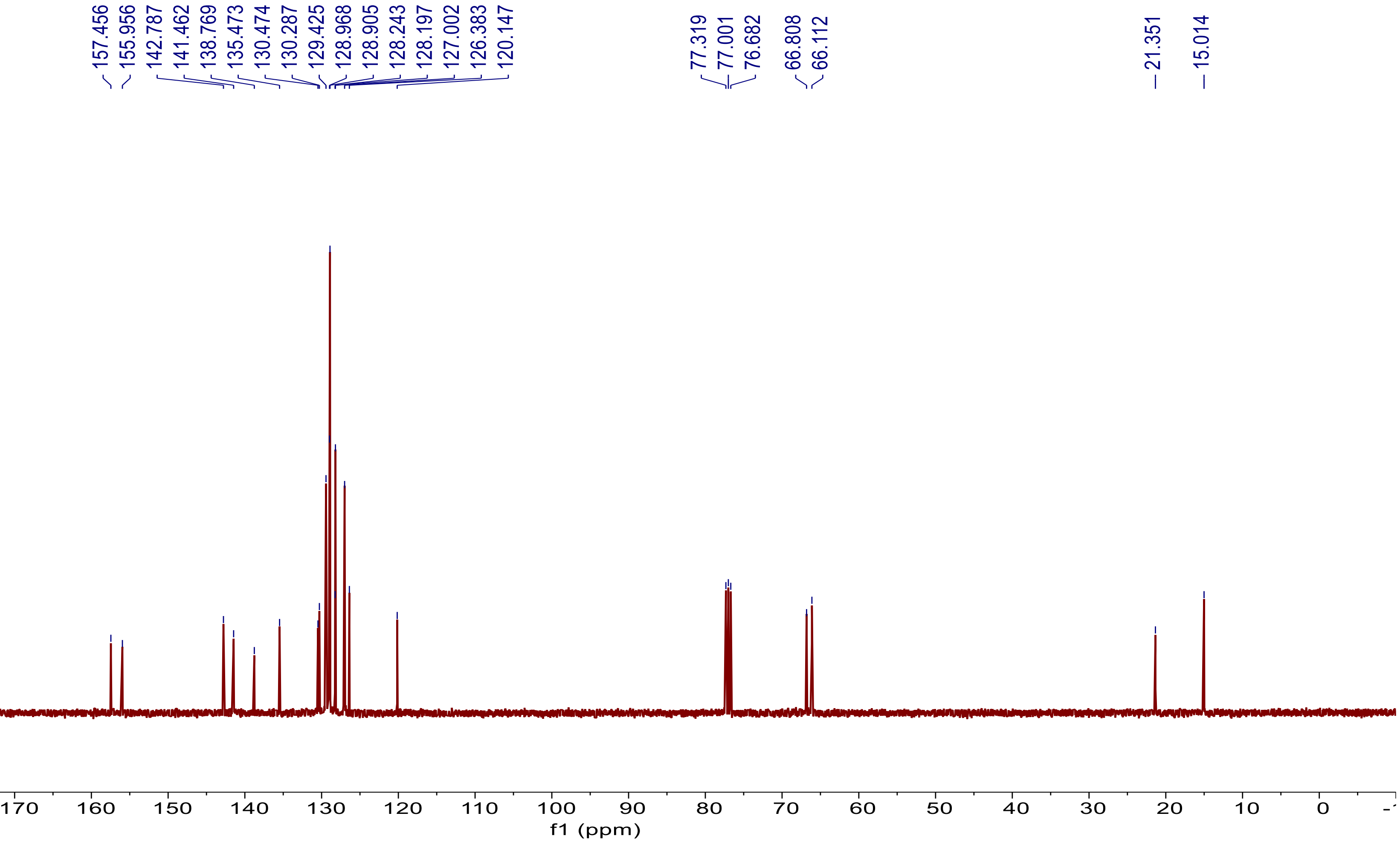

^{1}H NMR Spectrum of 6f at 25 ºC ($CDCl_3$)

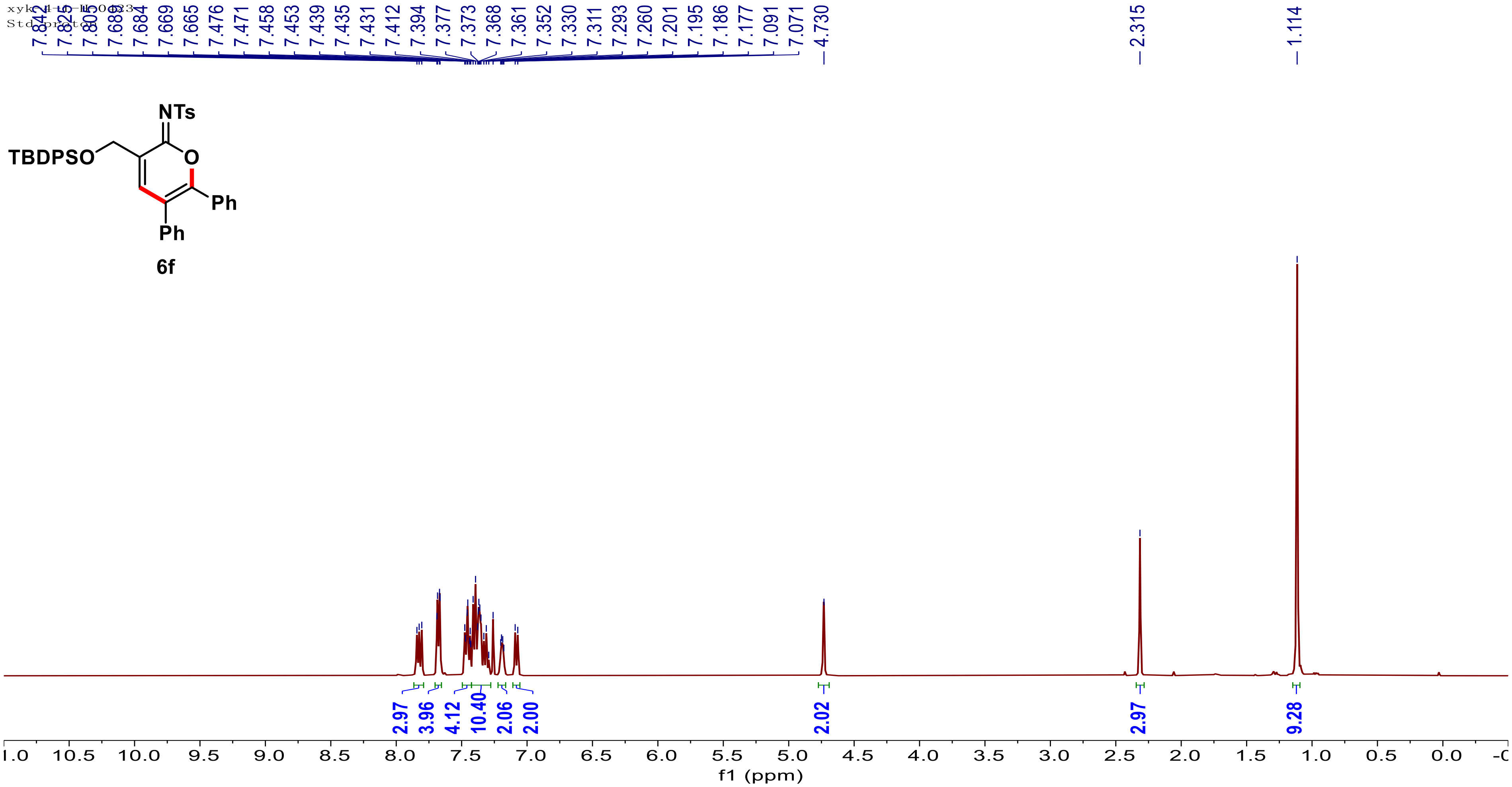

^{13}C NMR Spectrum of 6f at 25 °C ($CDCl_3$)

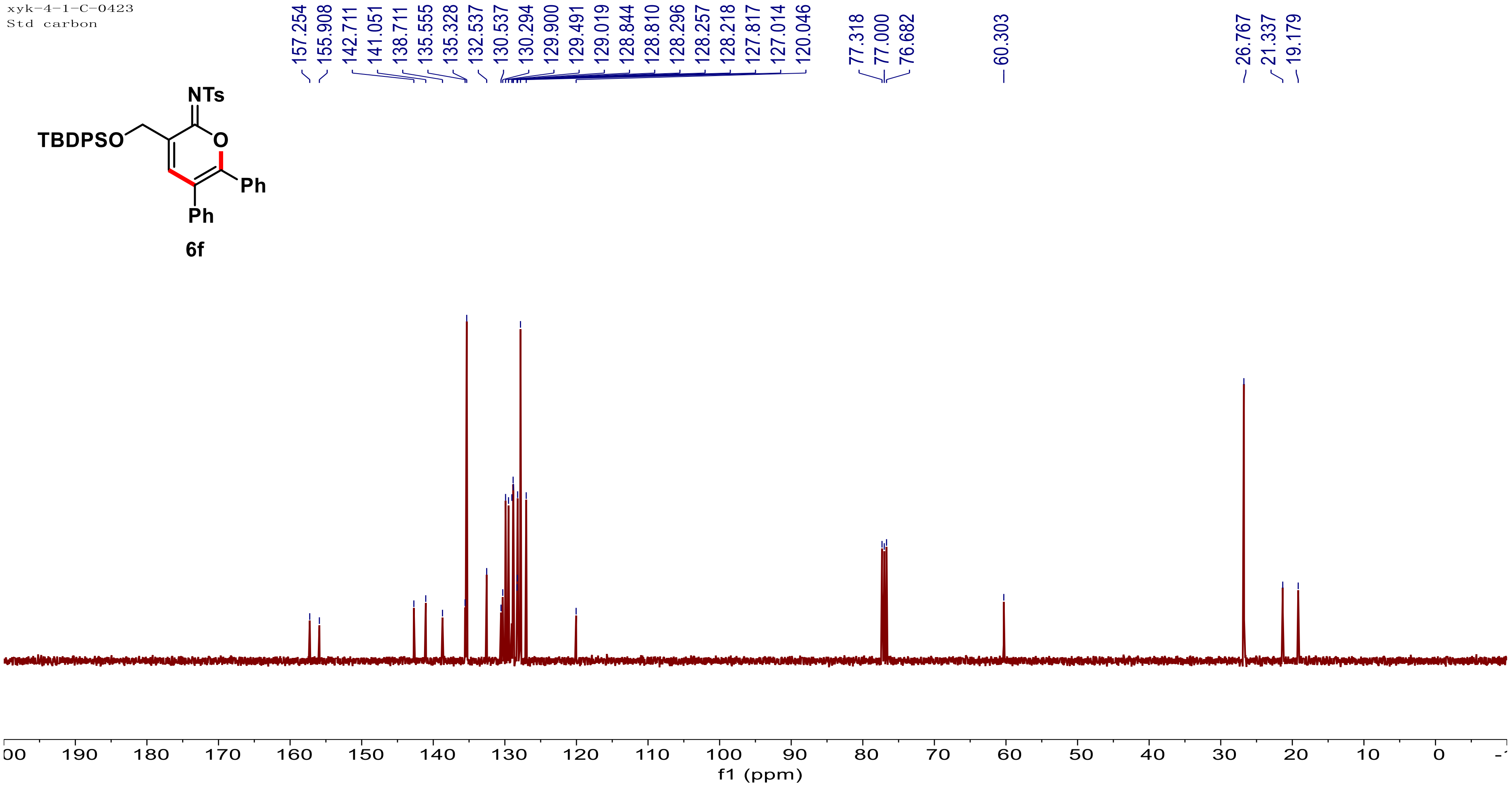

^{1}H NMR Spectrum of 6g at 25 ºC (CDCl$_3$)

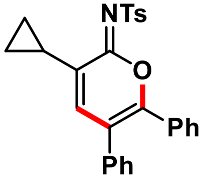

6g

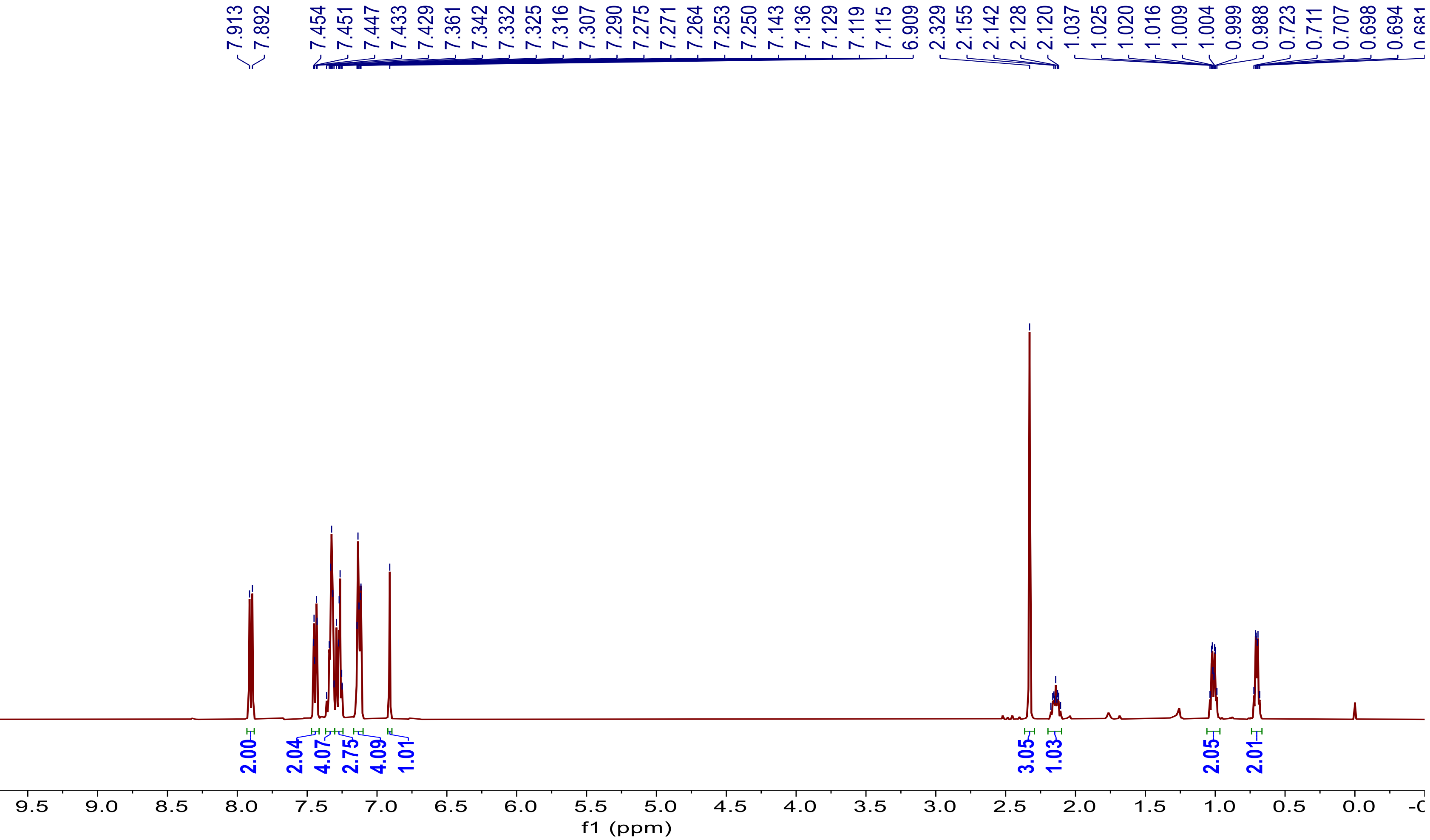

^{13}C NMR Spectrum of 6g at 25 ºC ($CDCl_3$)

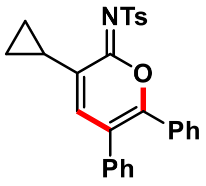

6g

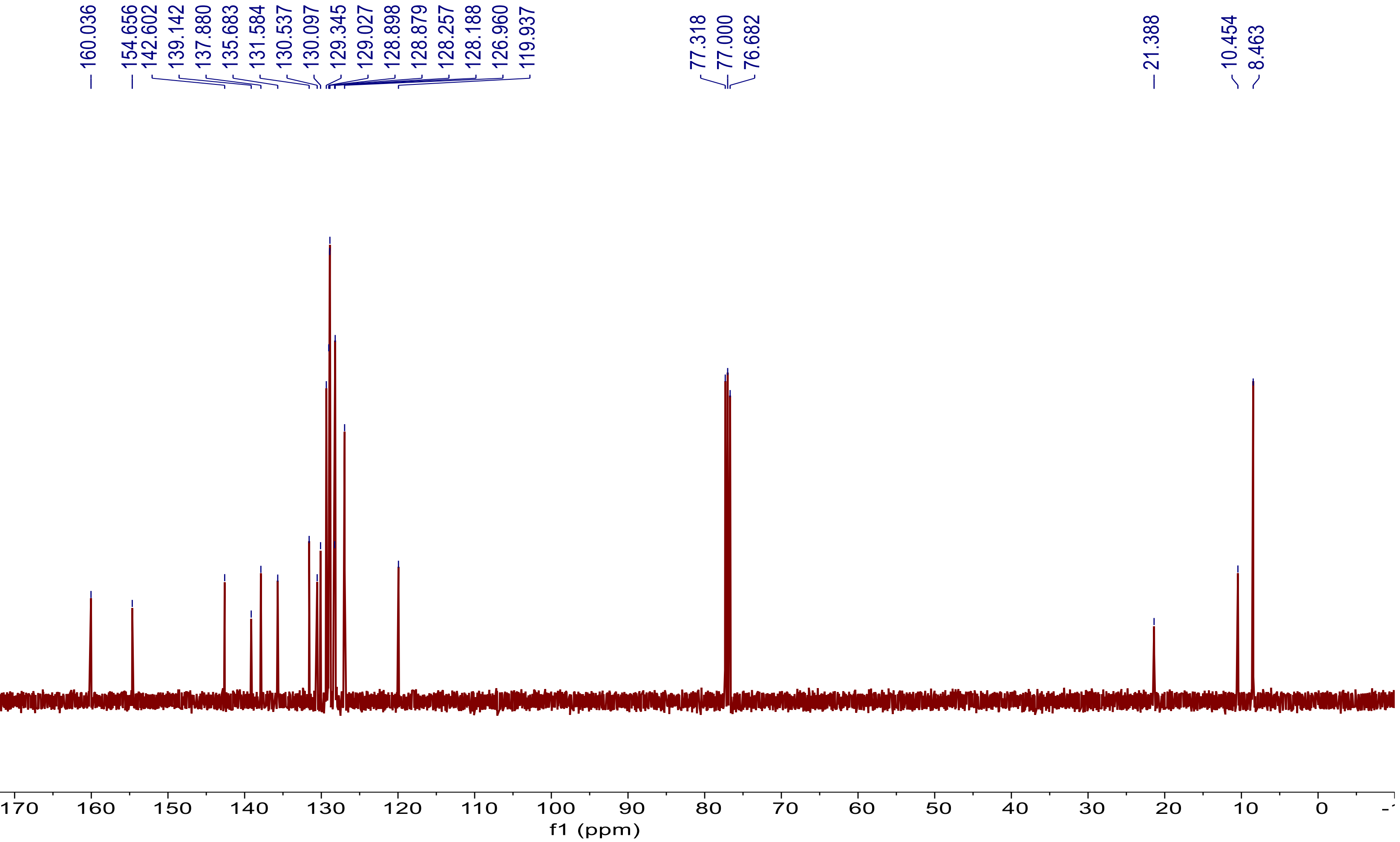

^{1}H NMR Spectrum of 6h at 25 °C ($CDCl_3$)

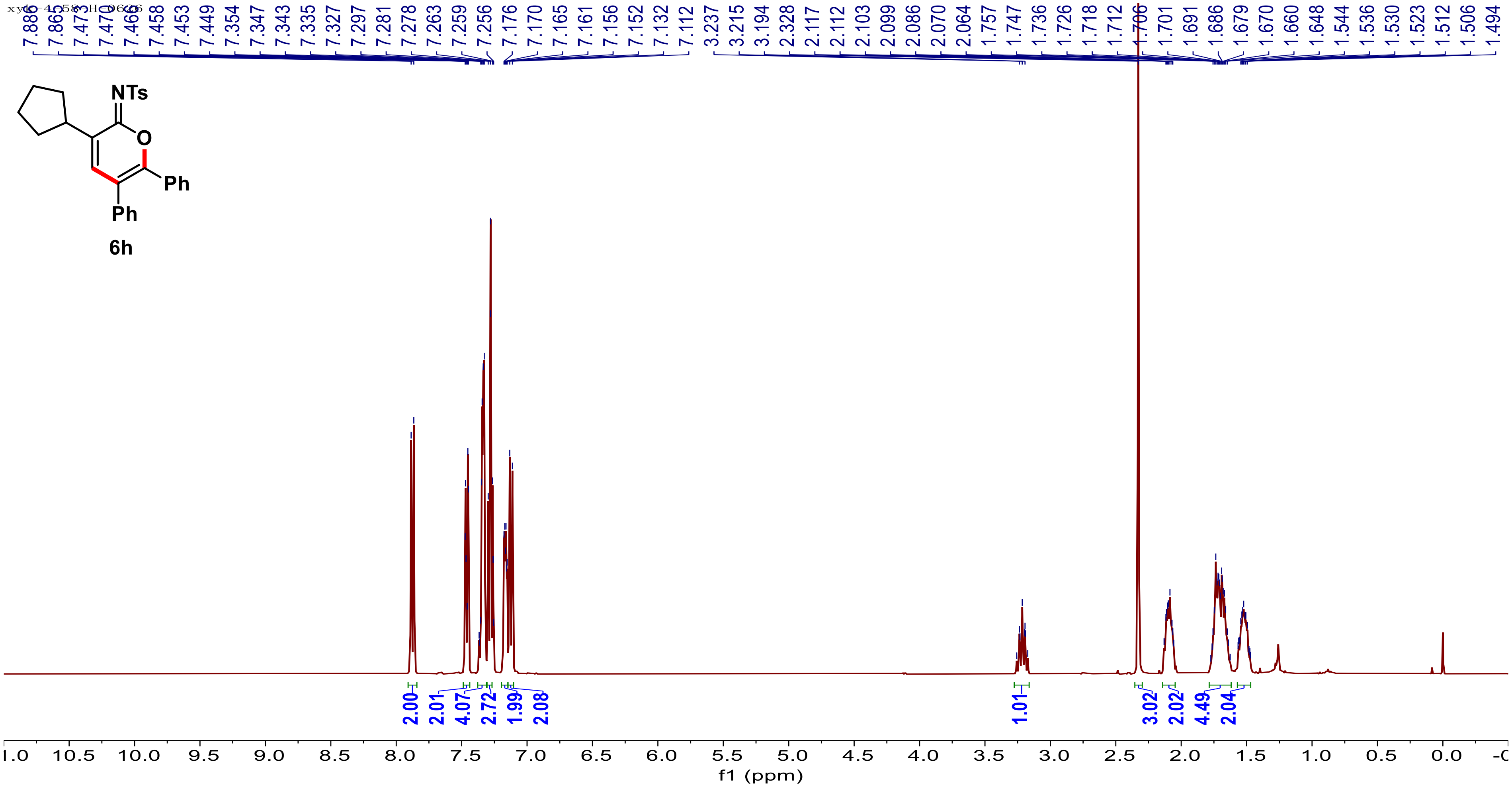

^{13}C NMR Spectrum of 6h at 25 ºC ($CDCl_3$)

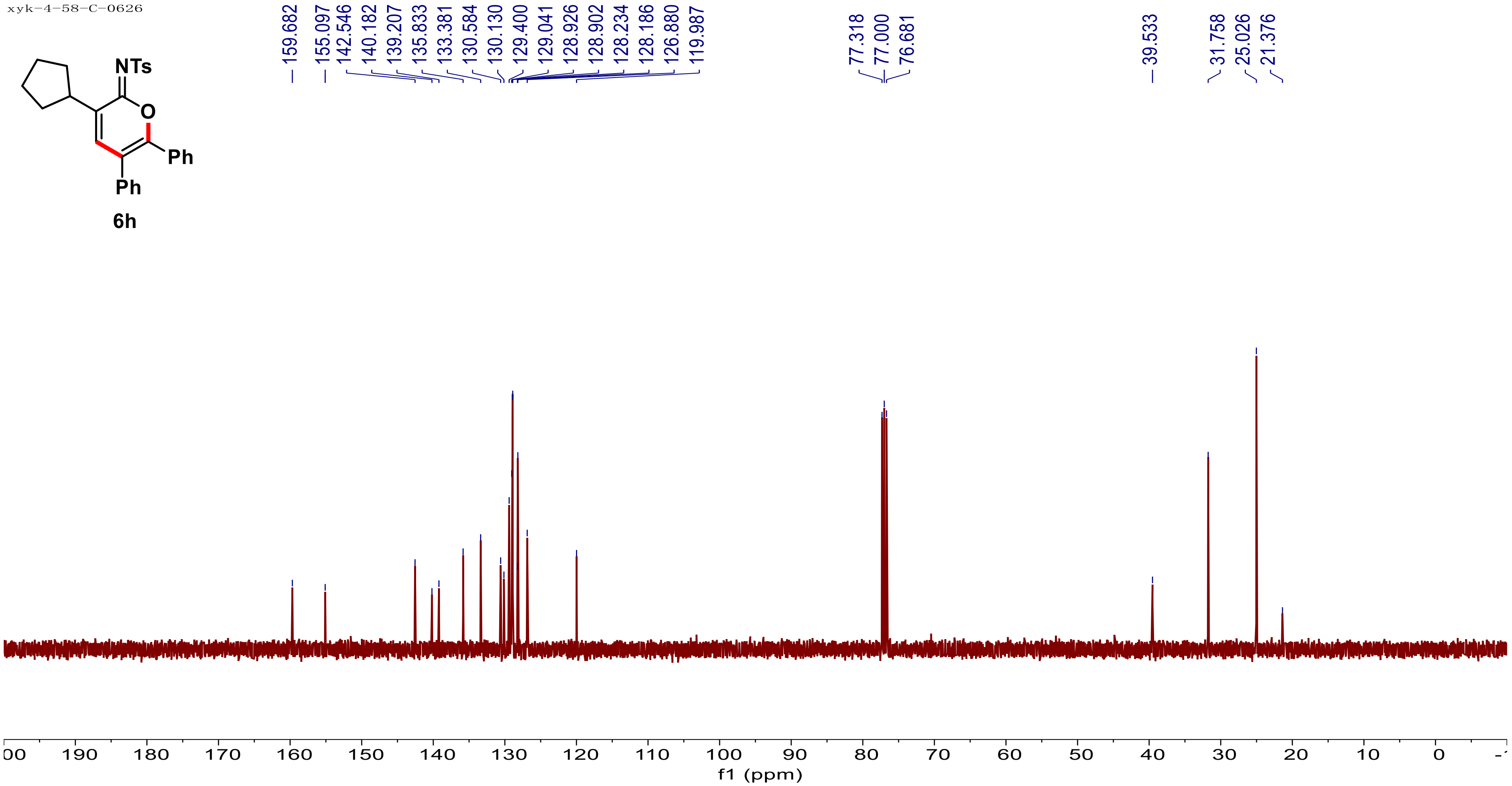

^{1}H NMR Spectrum of 6i at 25 °C ($CDCl_3$)

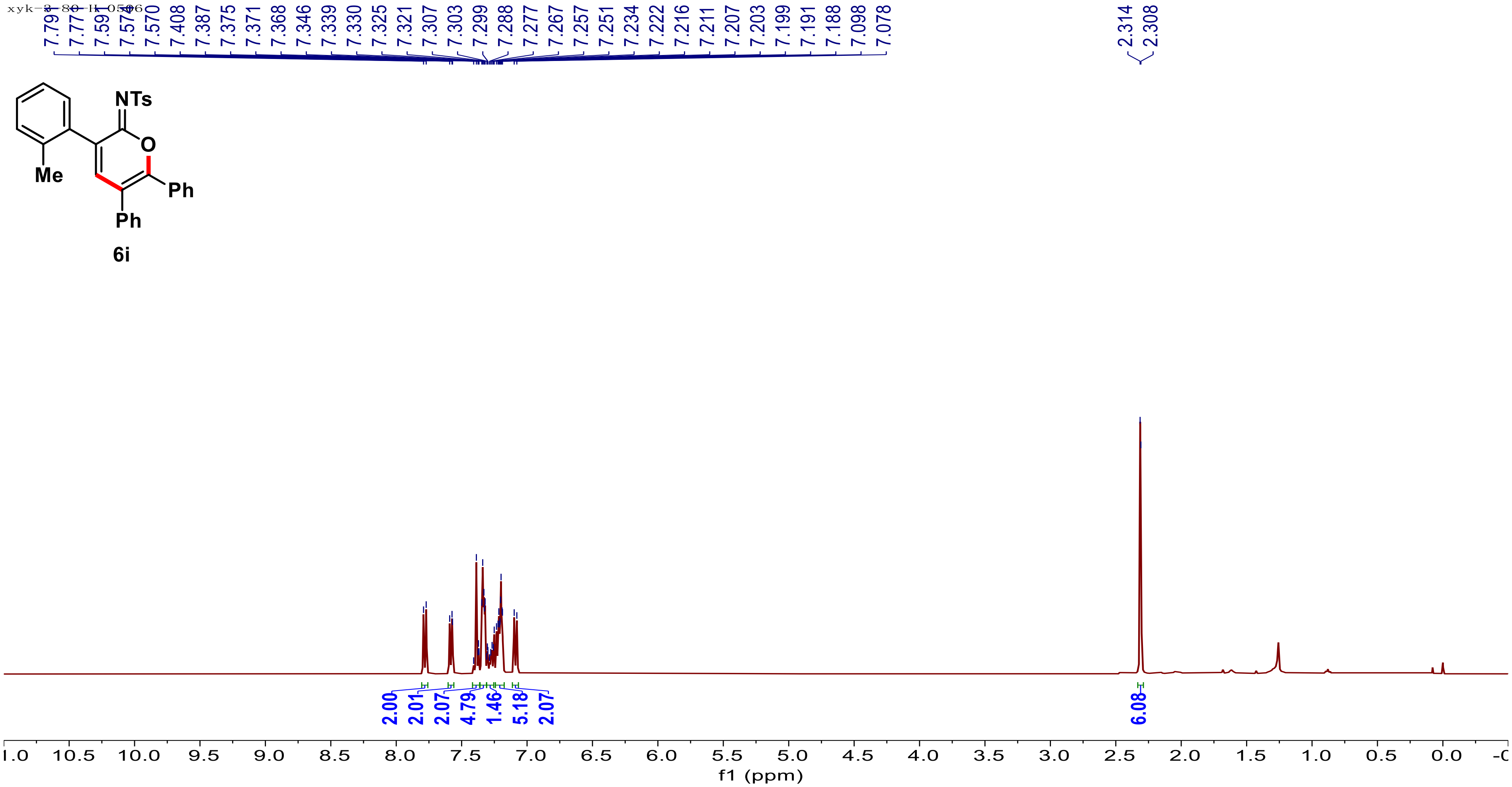

^{13}C NMR Spectrum of 6i at 25 °C (CDCl$_3$)

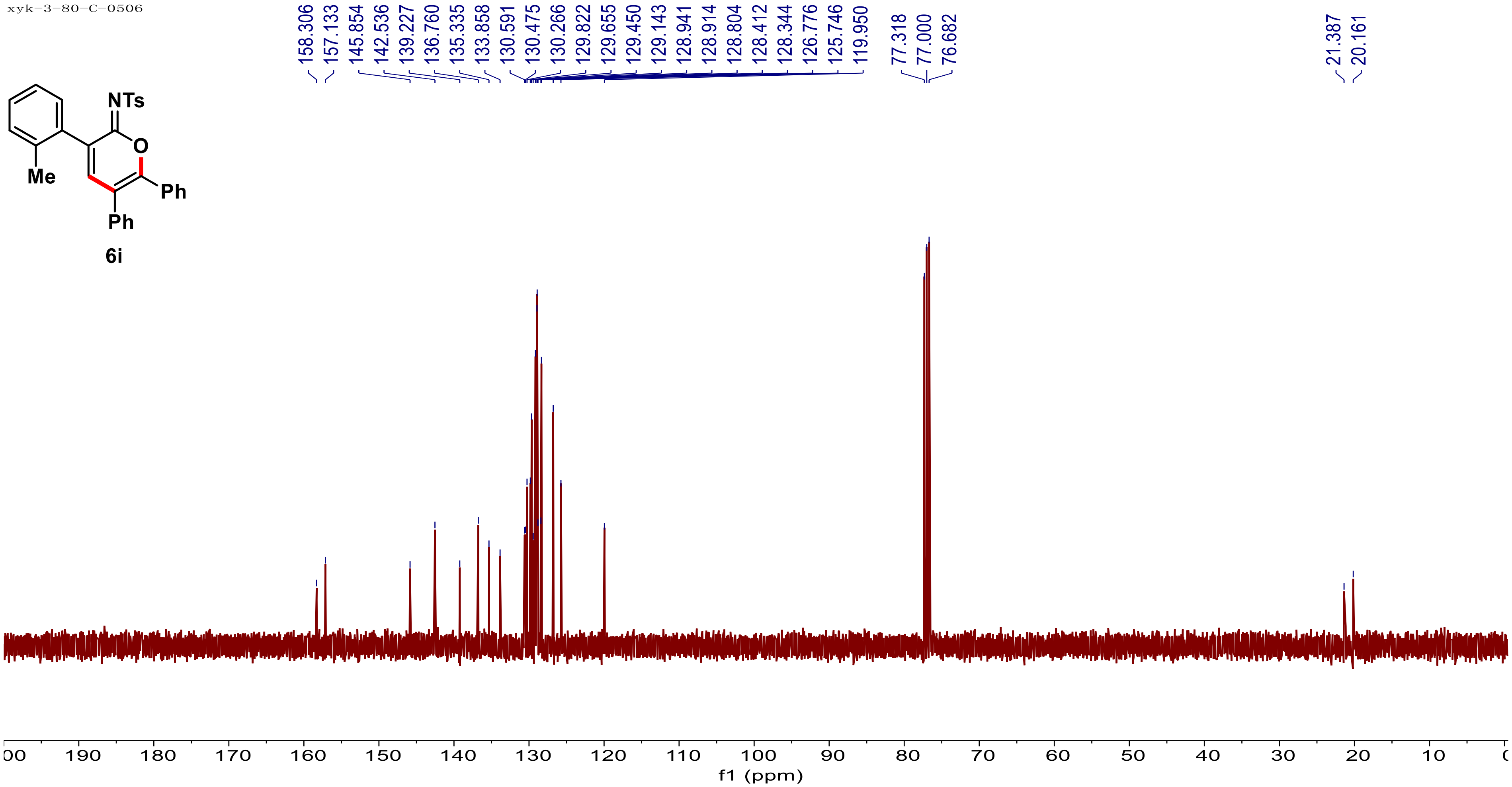

^{1}H NMR Spectrum of 6j at 25 ºC ($CDCl_3$)

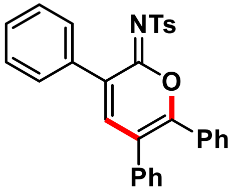

6j

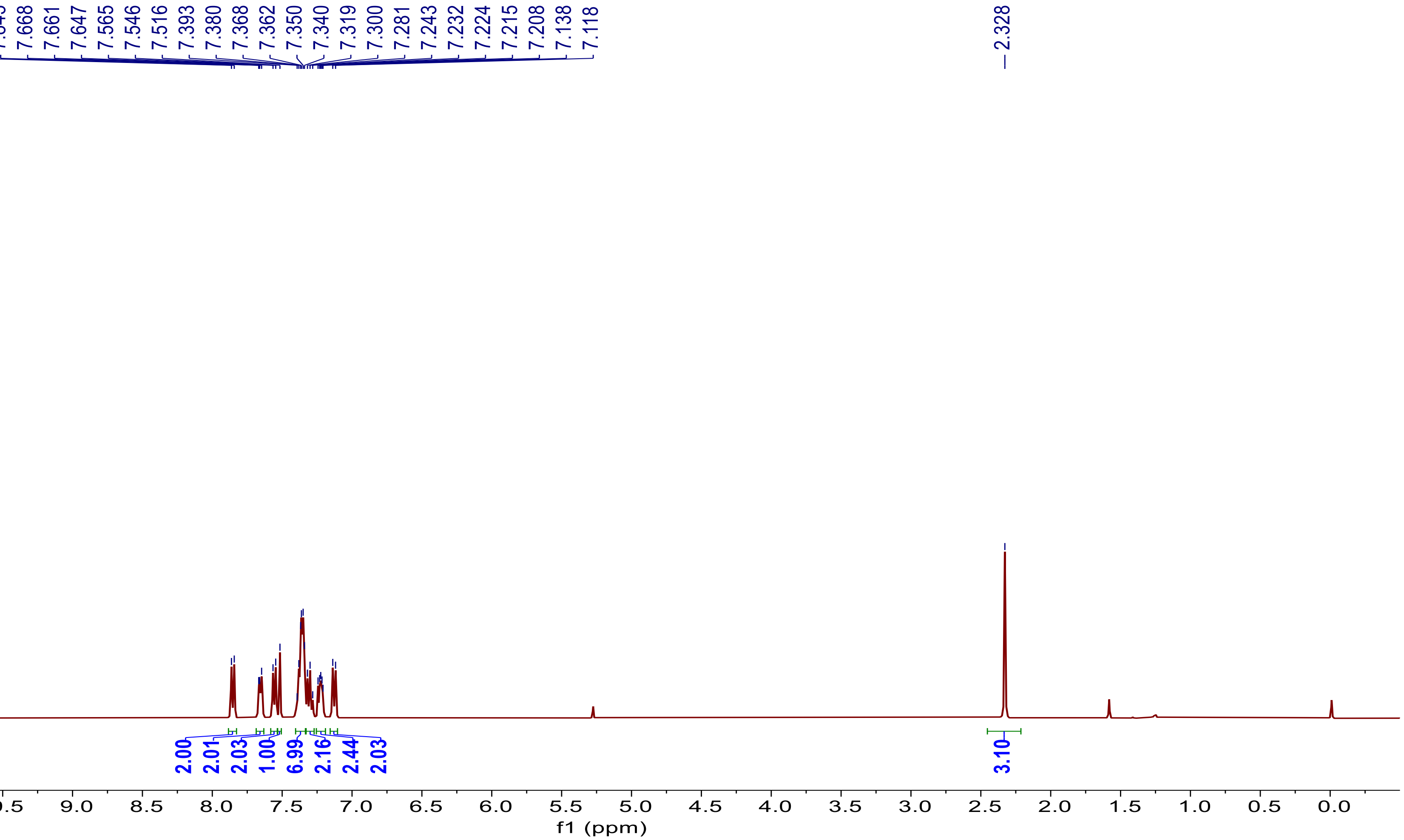

^{13}C NMR Spectrum of 6j at 25 °C ($CDCl_3$)

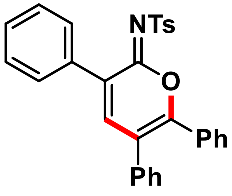

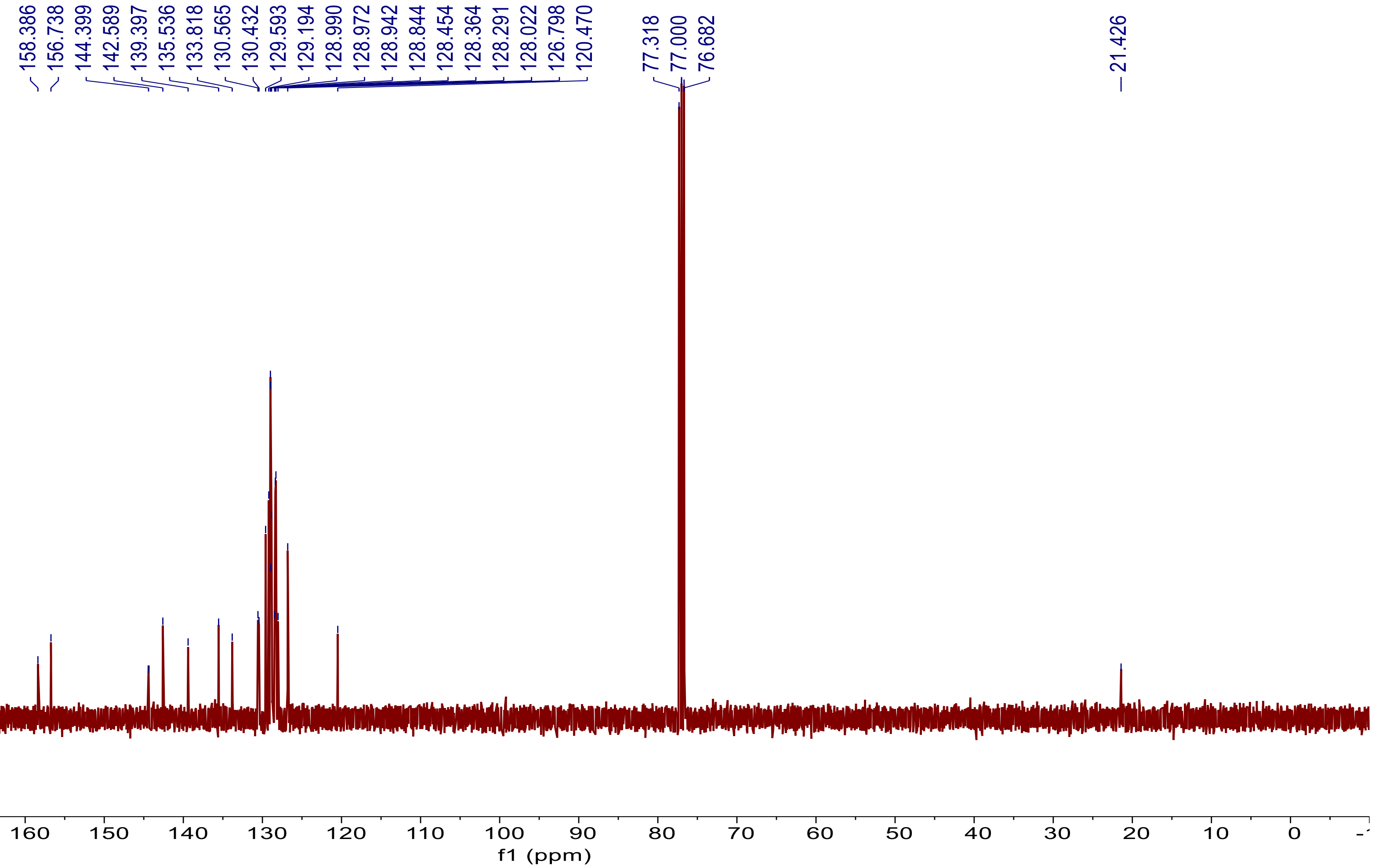

^{1}H NMR Spectrum of 6k at 25 °C ($CDCl_3$)

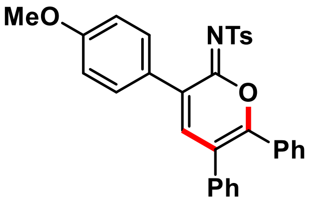

6k

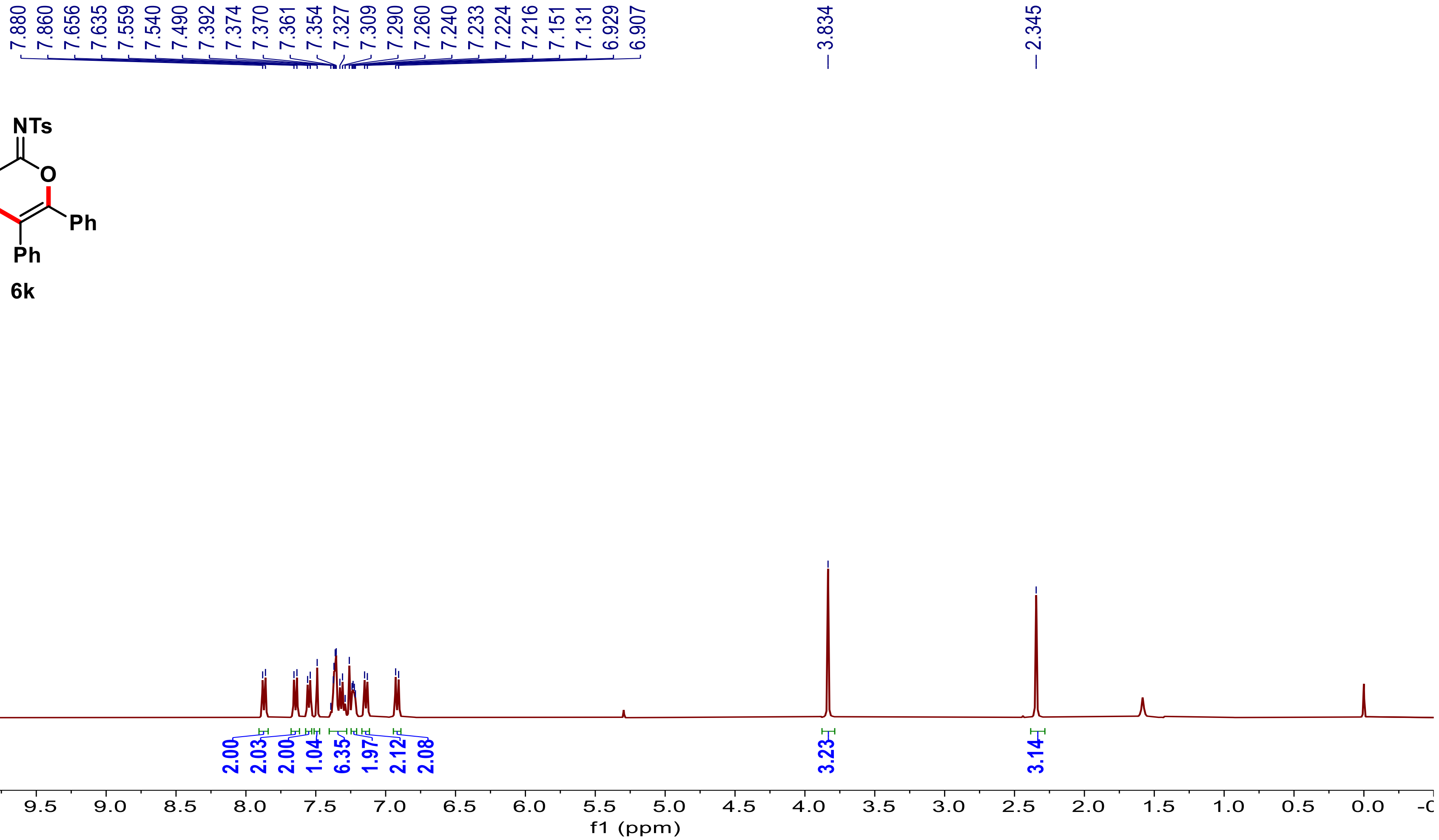

^{13}C NMR Spectrum of 6k at 25 ºC ($CDCl_3$)

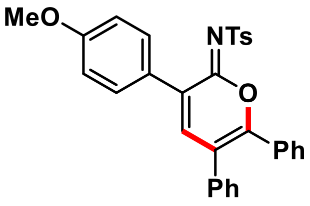

6k

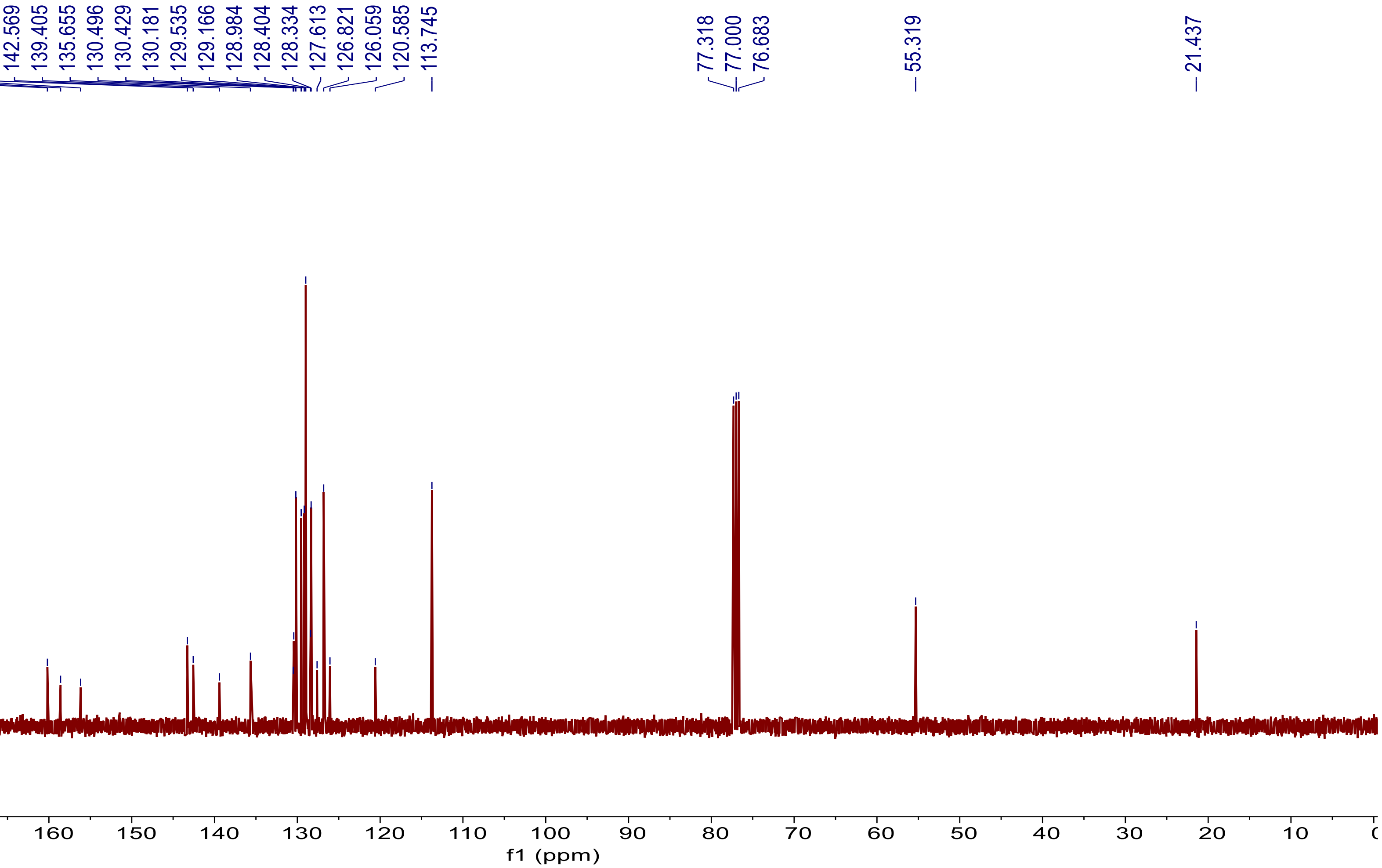

^{1}H NMR Spectrum of 6l at 25 ºC ($CDCl_3$)

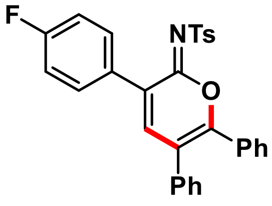

6l

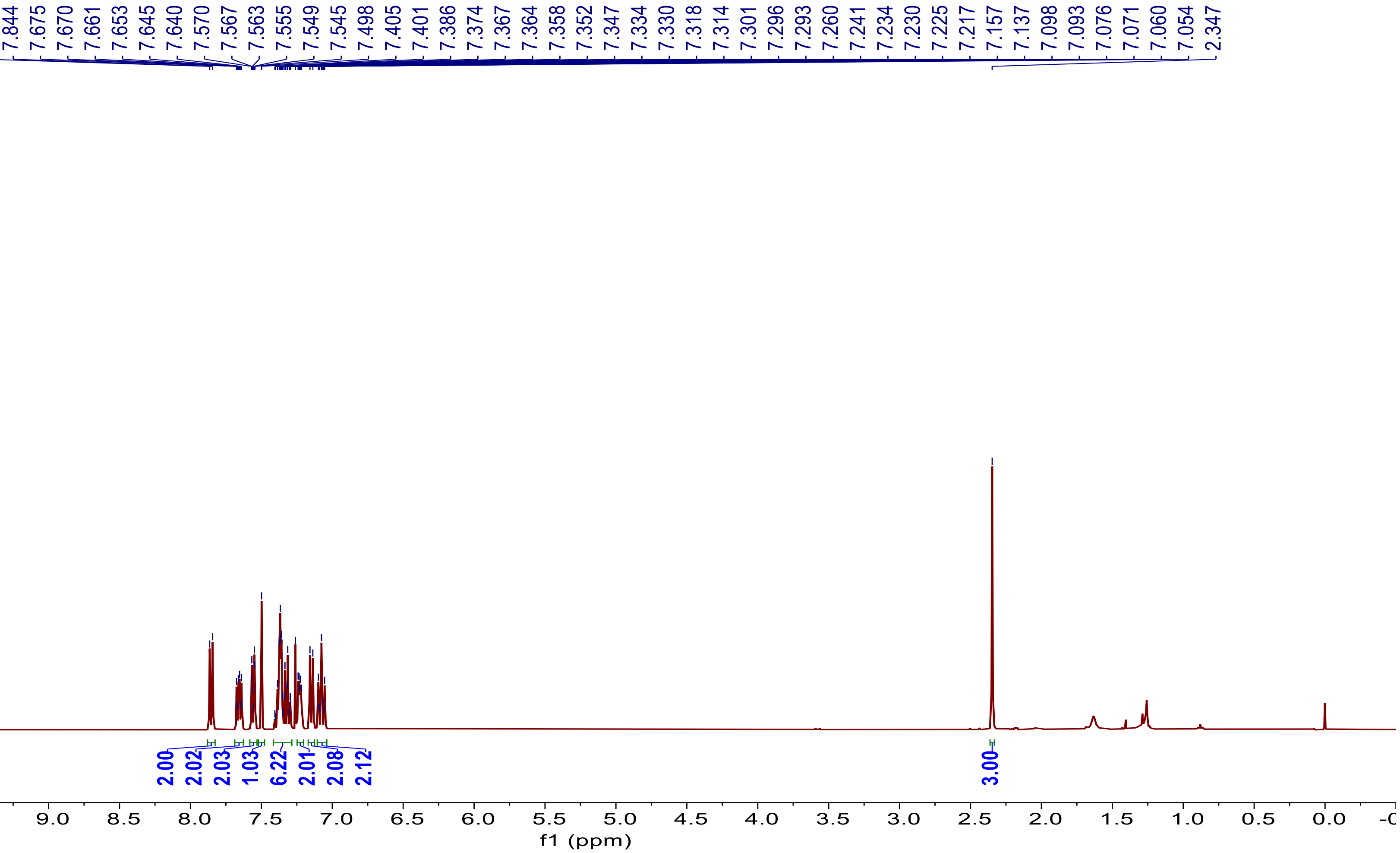

^{13}C NMR Spectrum of 6l at 25 °C ($CDCl_3$)

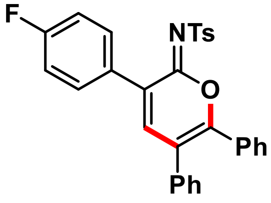

6l

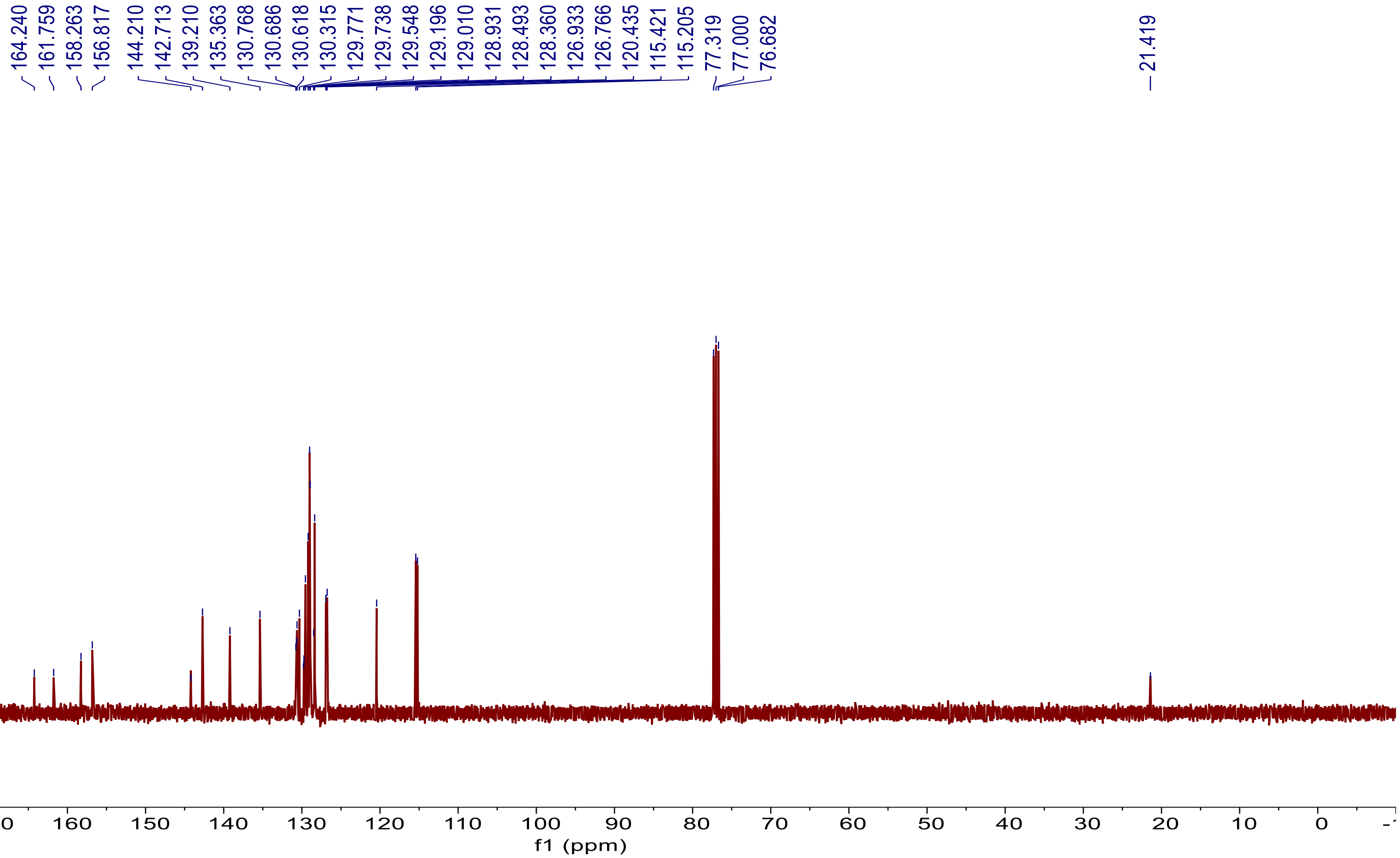

^{19}F NMR Spectrum of 6l at 25 ºC ($CDCl_3$)

xyk-3-100-F-0527
STANDARD PROTON PARAMETERS

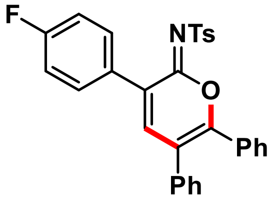

6l

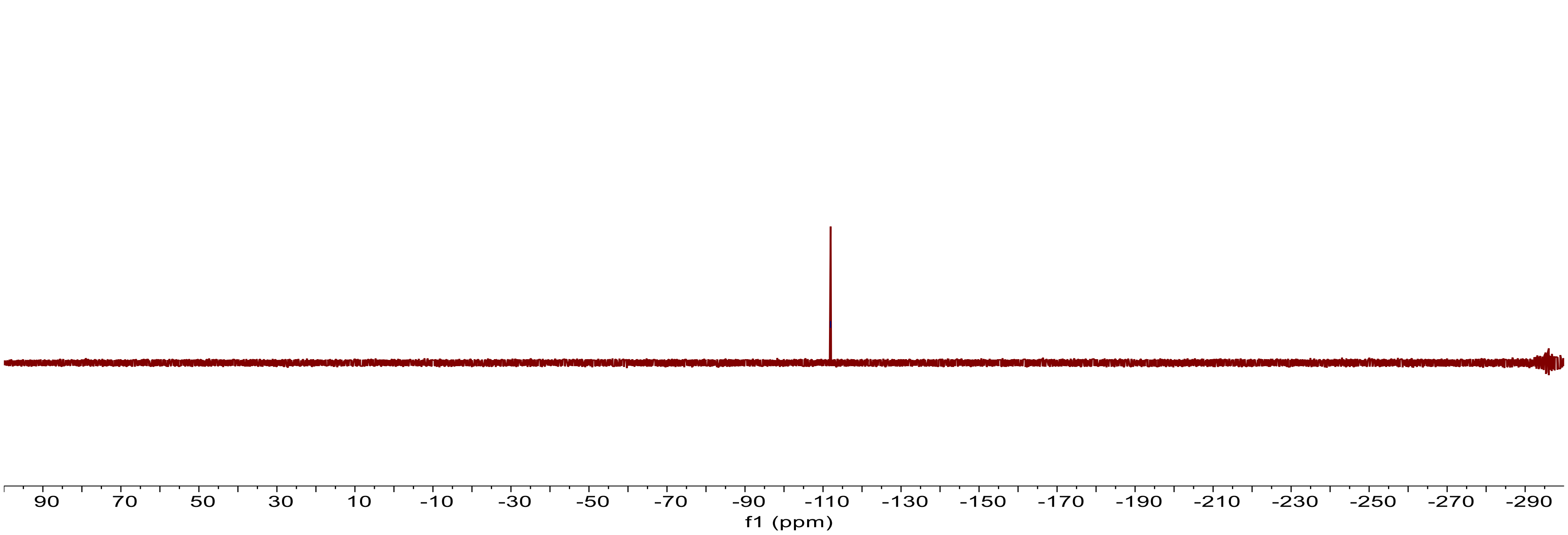

^{1}H NMR Spectrum of 6m at 25 ºC ($CDCl_3$)

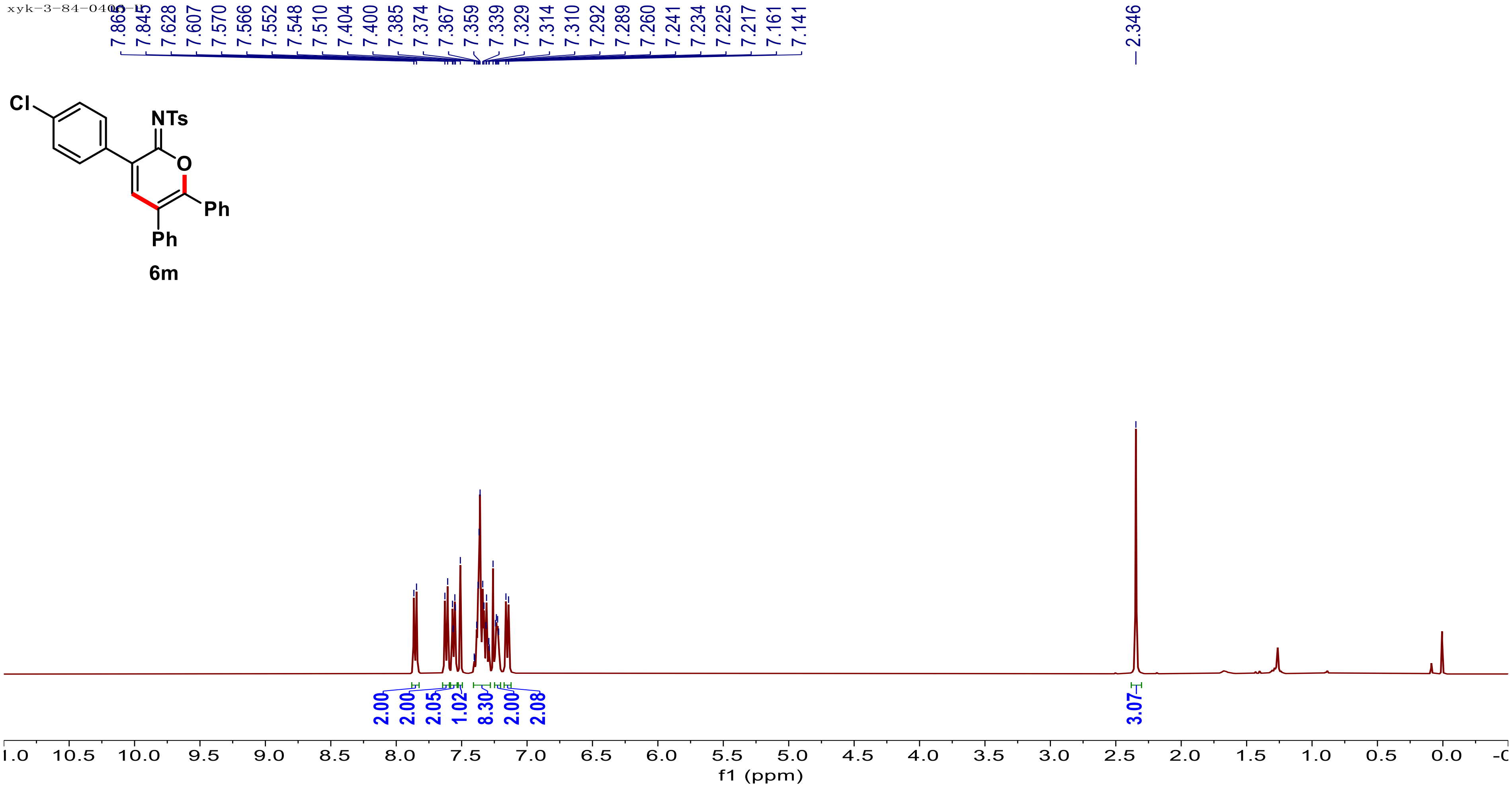

^{13}C NMR Spectrum of 6m at 25 ^{o}C ($CDCl_3$)

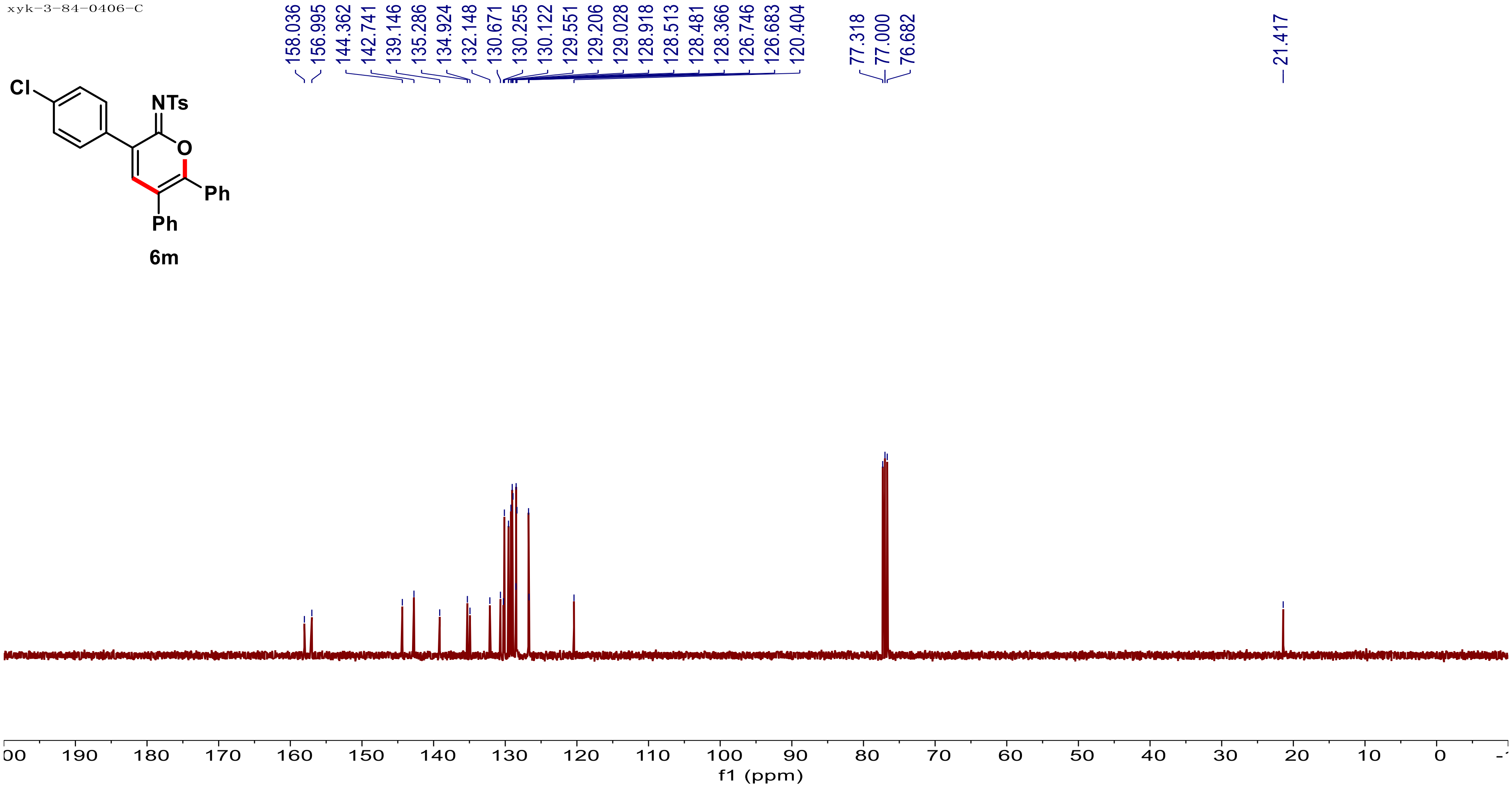

^{1}H NMR Spectrum of 6n at 25 °C ($CDCl_3$)

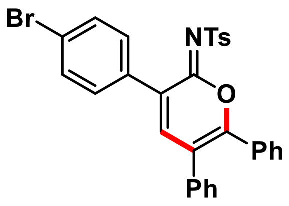

6n

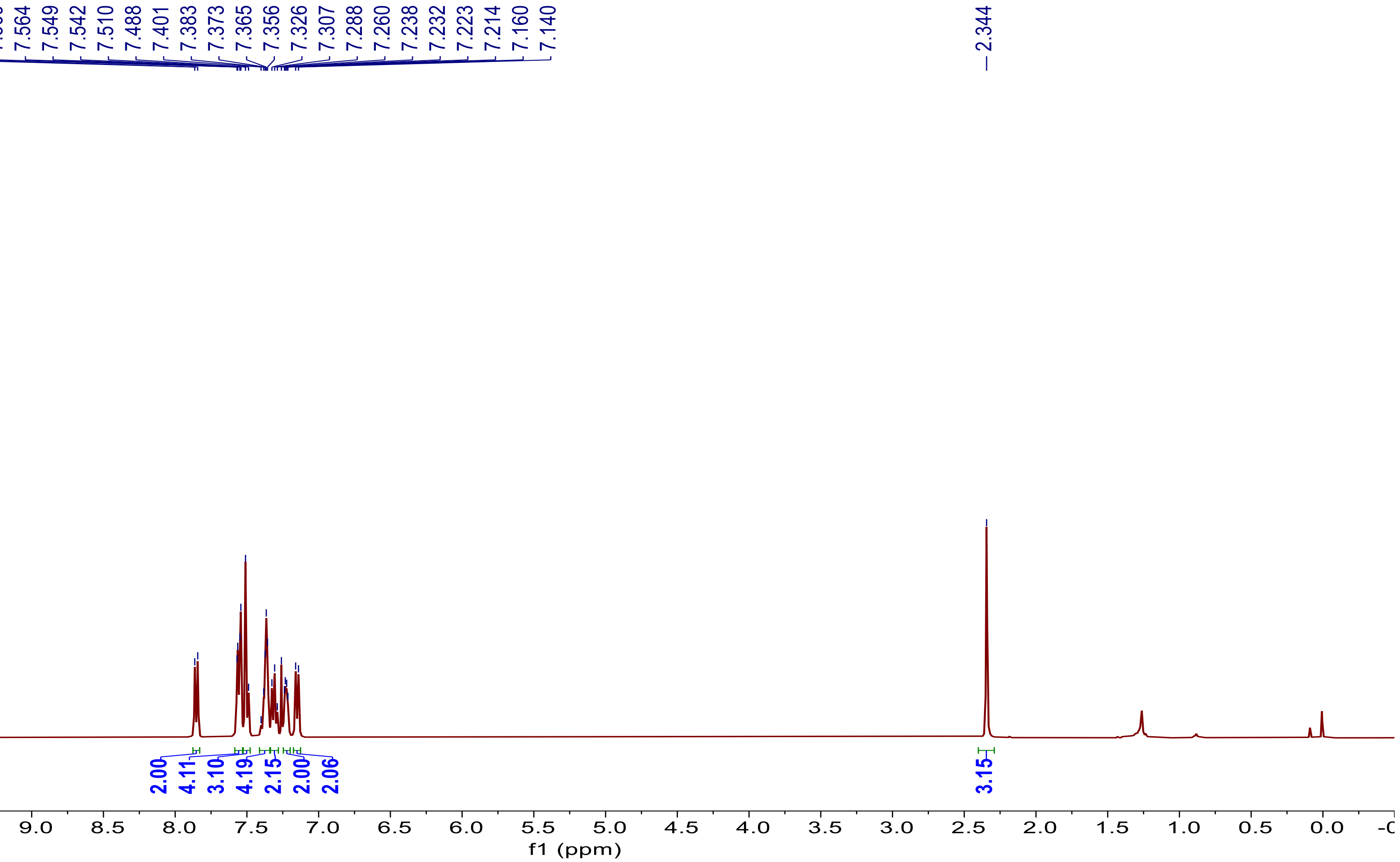

^{13}C NMR Spectrum of 6n at 25 °C (CDCl$_3$)

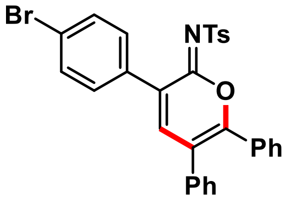

6n

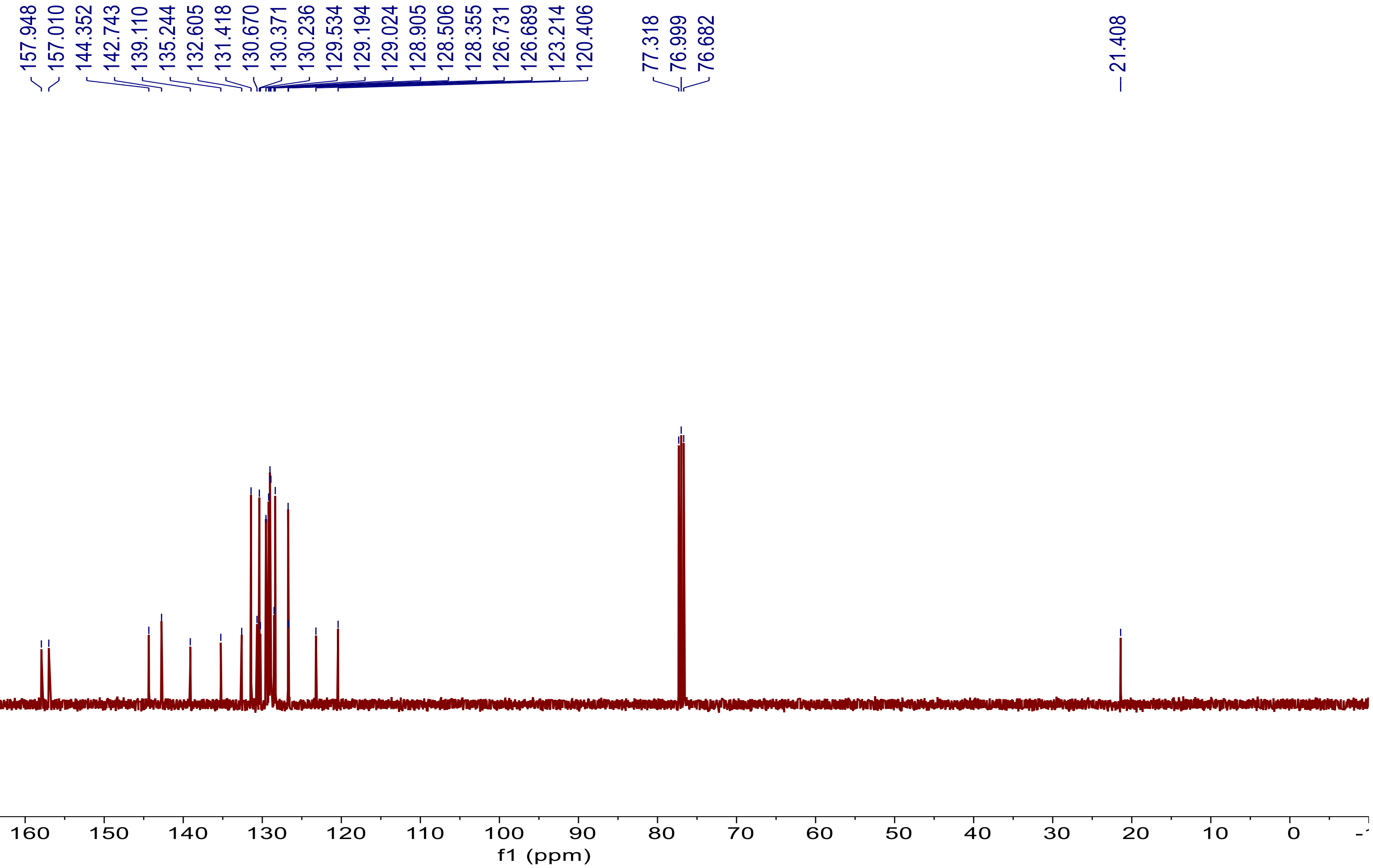

^{1}H NMR Spectrum of 6o at 25 °C (CDCl$_3$)

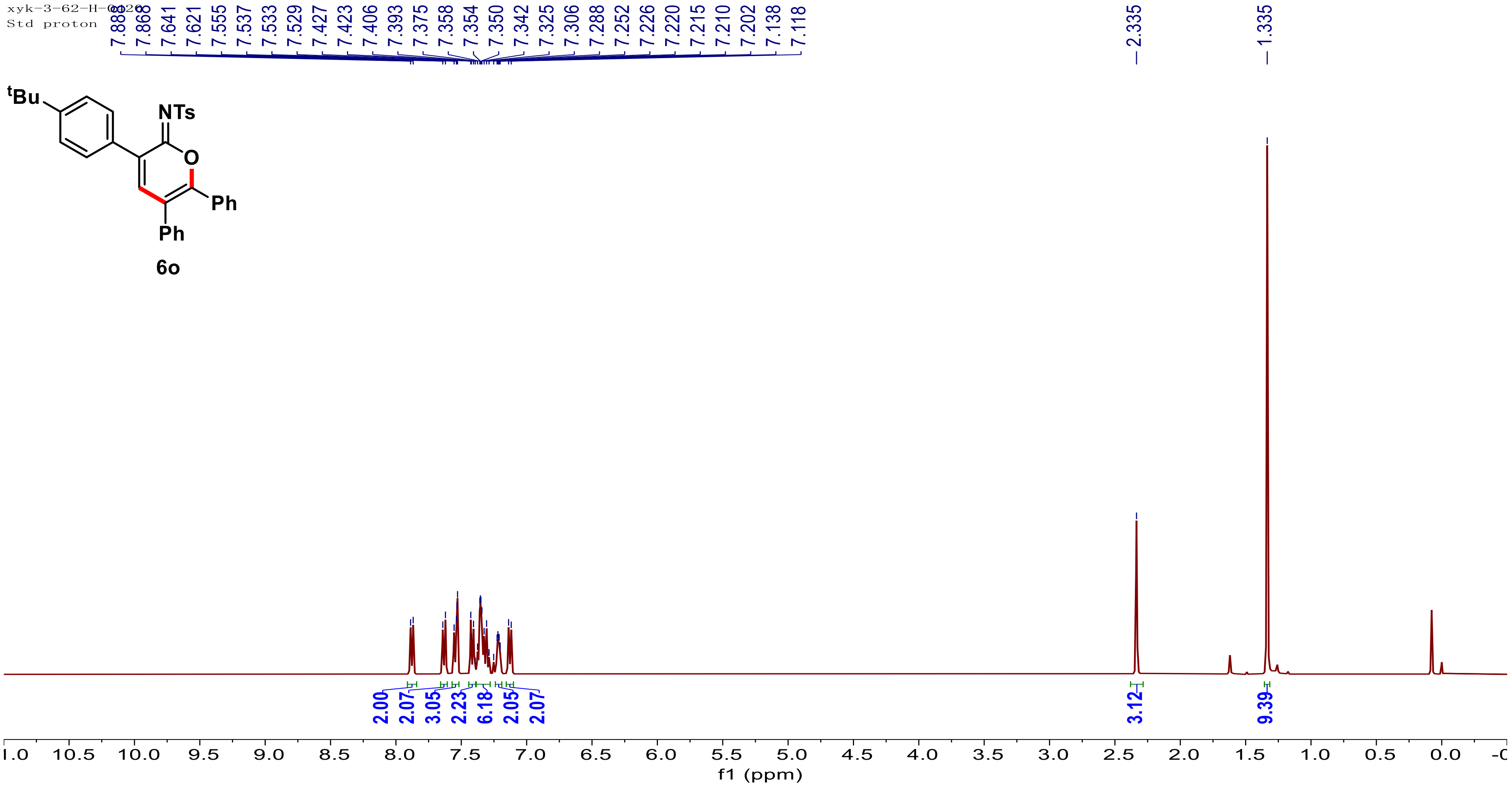

^{13}C NMR Spectrum of 6o at 25 ºC (CDCl$_3$)

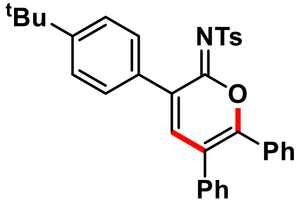

6o

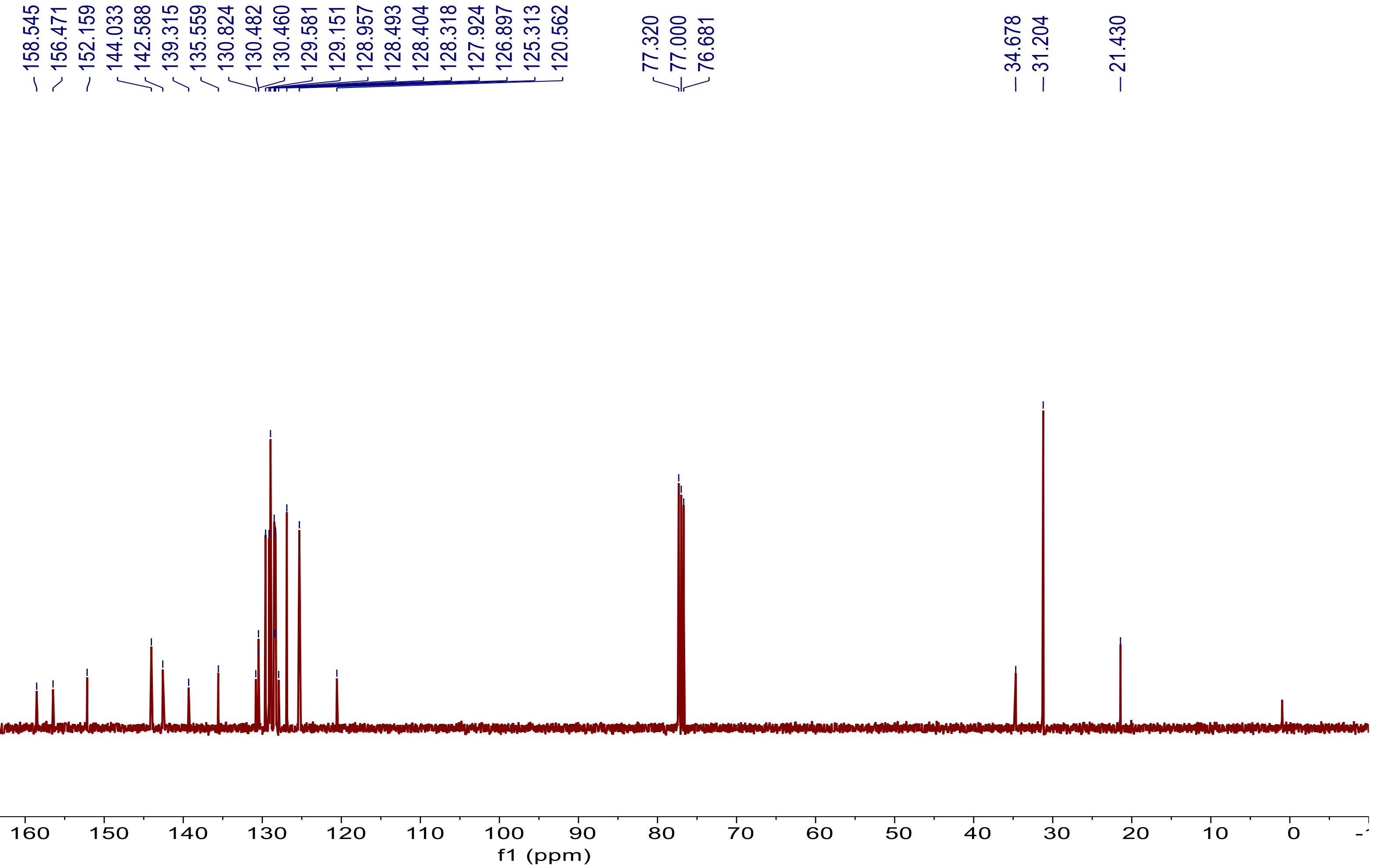

^{1}H NMR Spectrum of 6p at 25 ºC ($CDCl_3$)

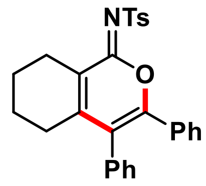

6p

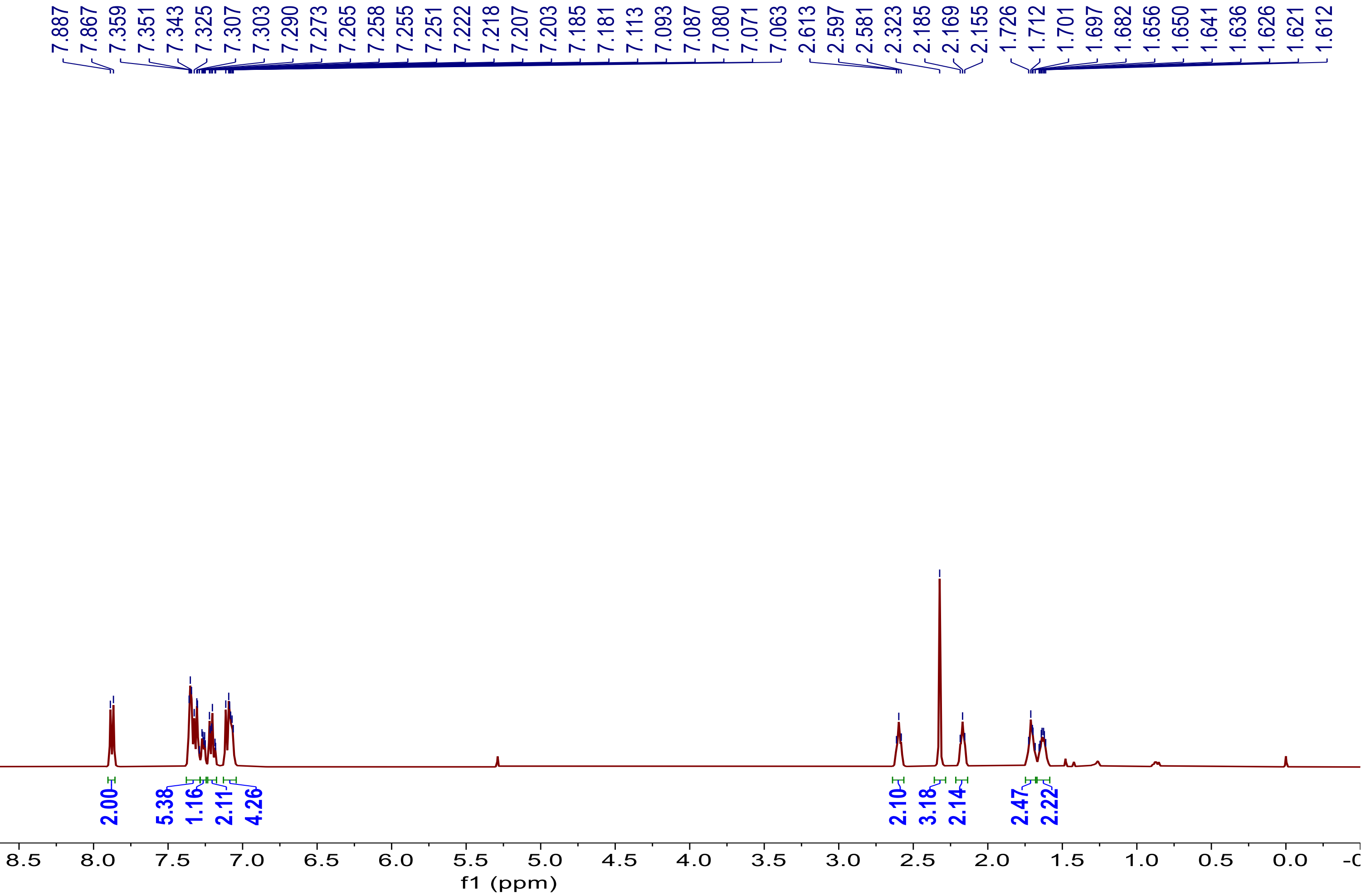

^{13}C NMR Spectrum of 6p at 25 ^{o}C ($CDCl_3$)

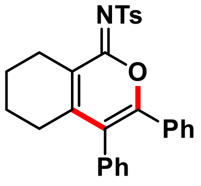

6p

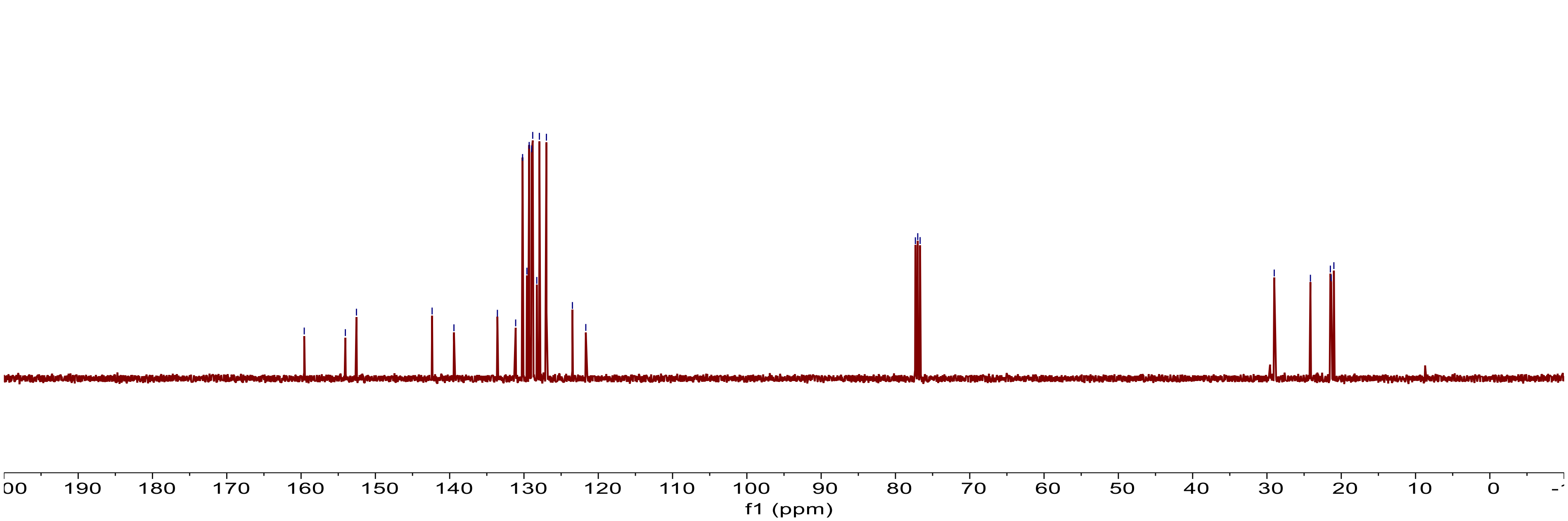

^{1}H NMR Spectrum of 6q at 25 ºC ($CDCl_3$)

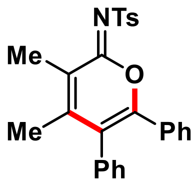

6q

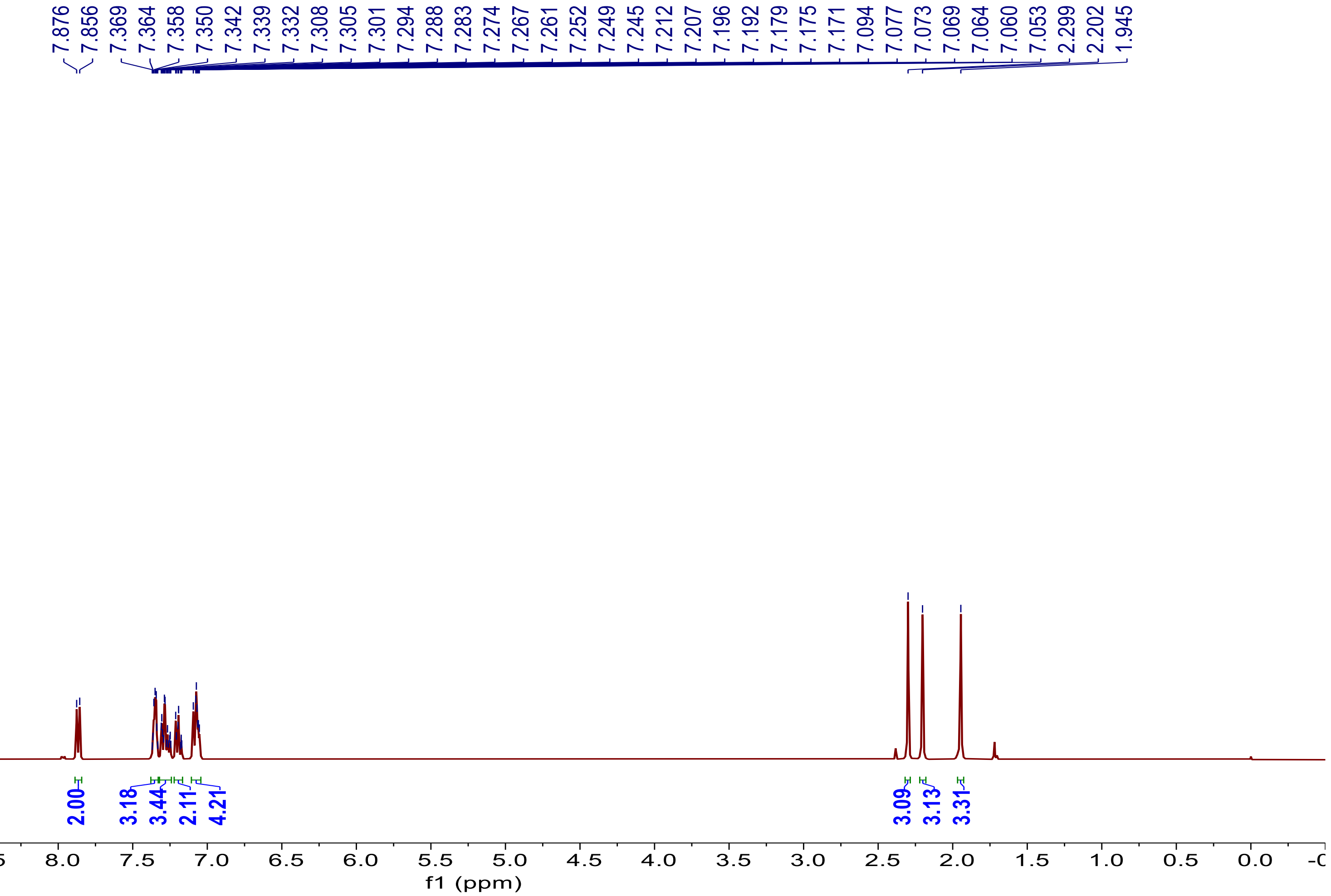

^{13}C NMR Spectrum of 6q at 25 °C ($CDCl_3$)

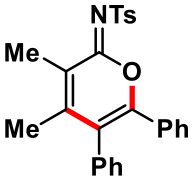

6q

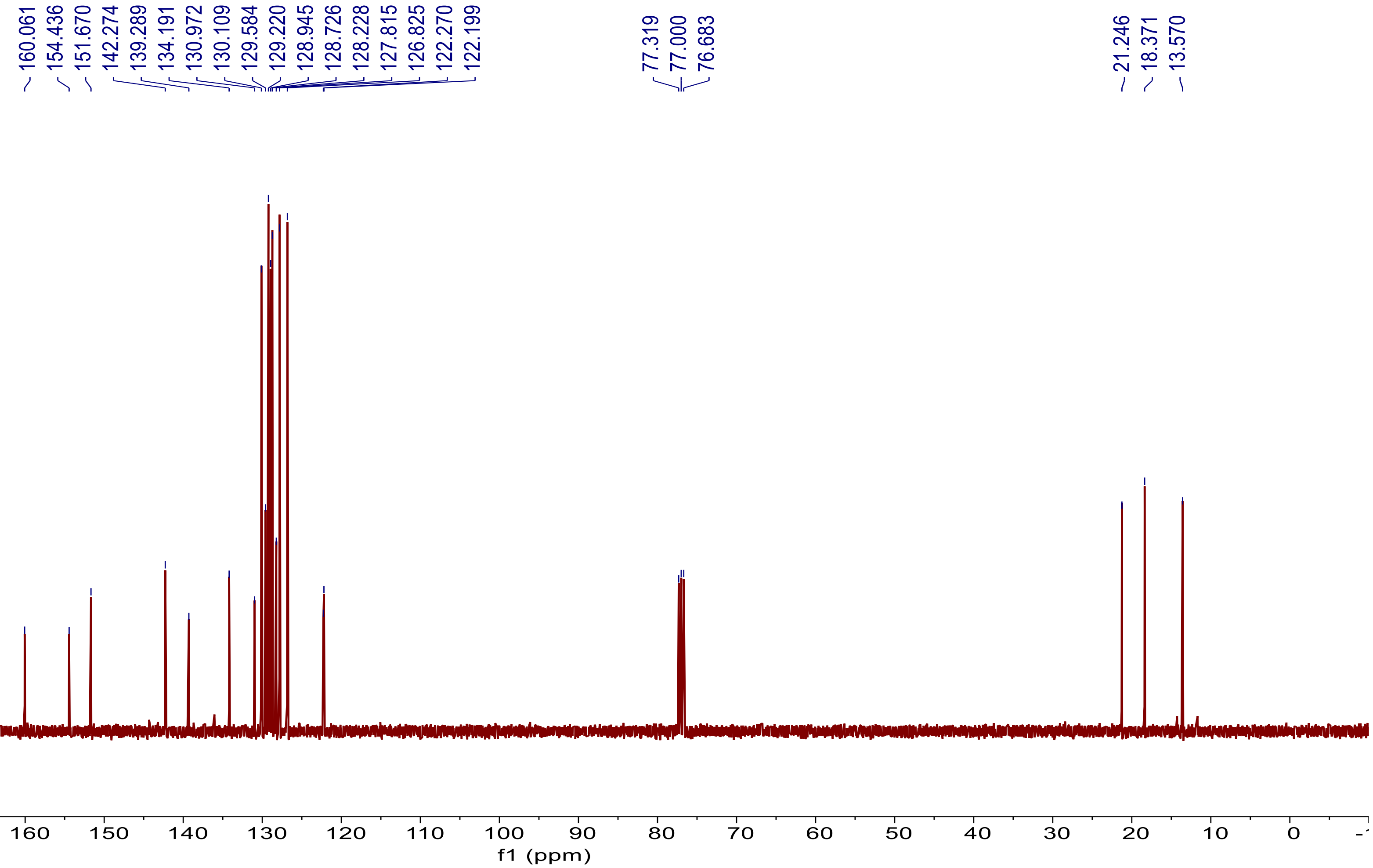

^{1}H NMR Spectrum of 6r at 25 ºC ($CDCl_3$)

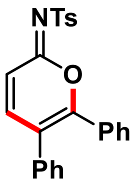

6r

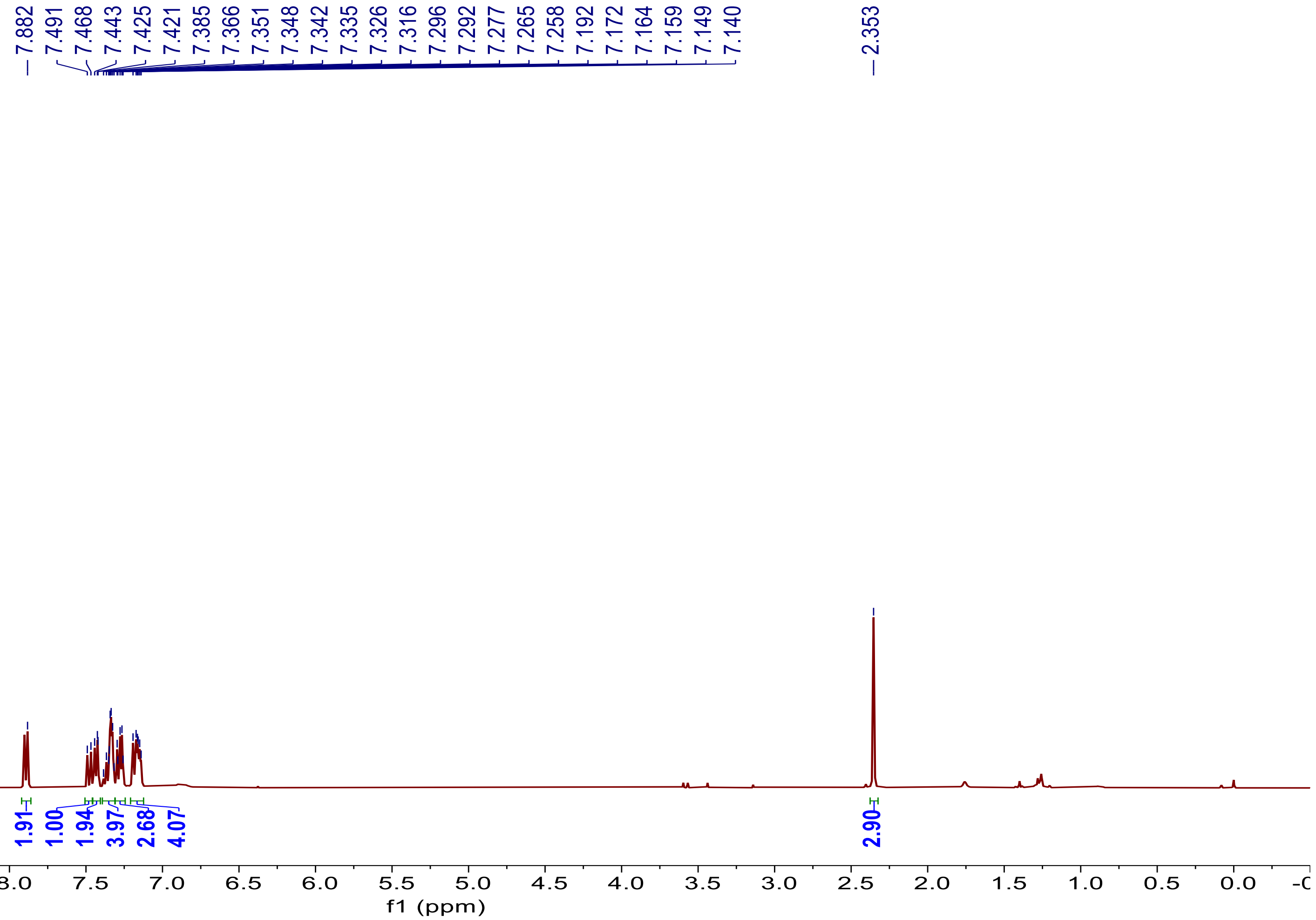

^{13}C NMR Spectrum of 6r at 25 ºC ($CDCl_3$)

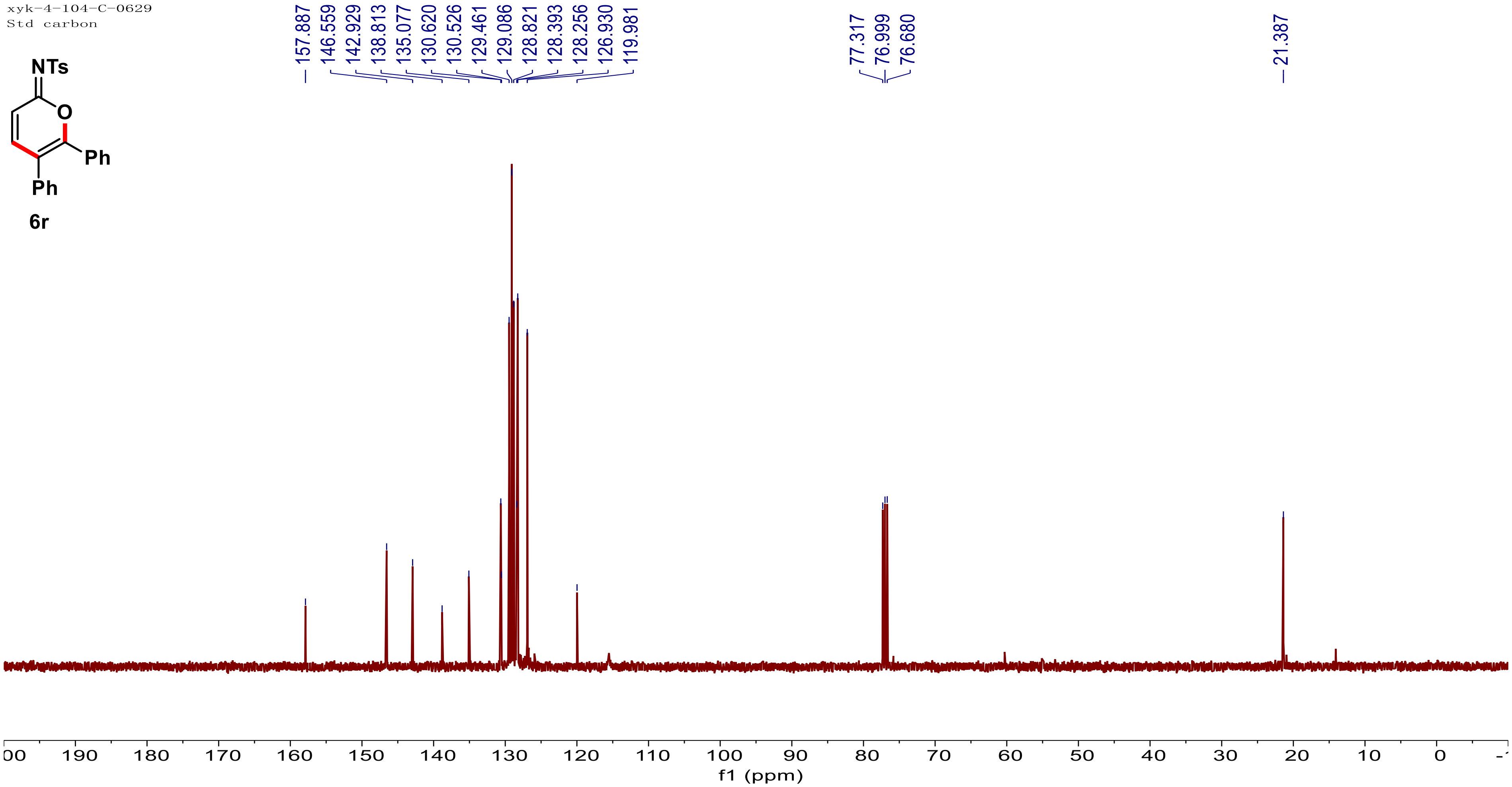

^{1}H NMR Spectrum of 7a at 25 °C ($CDCl_3$)

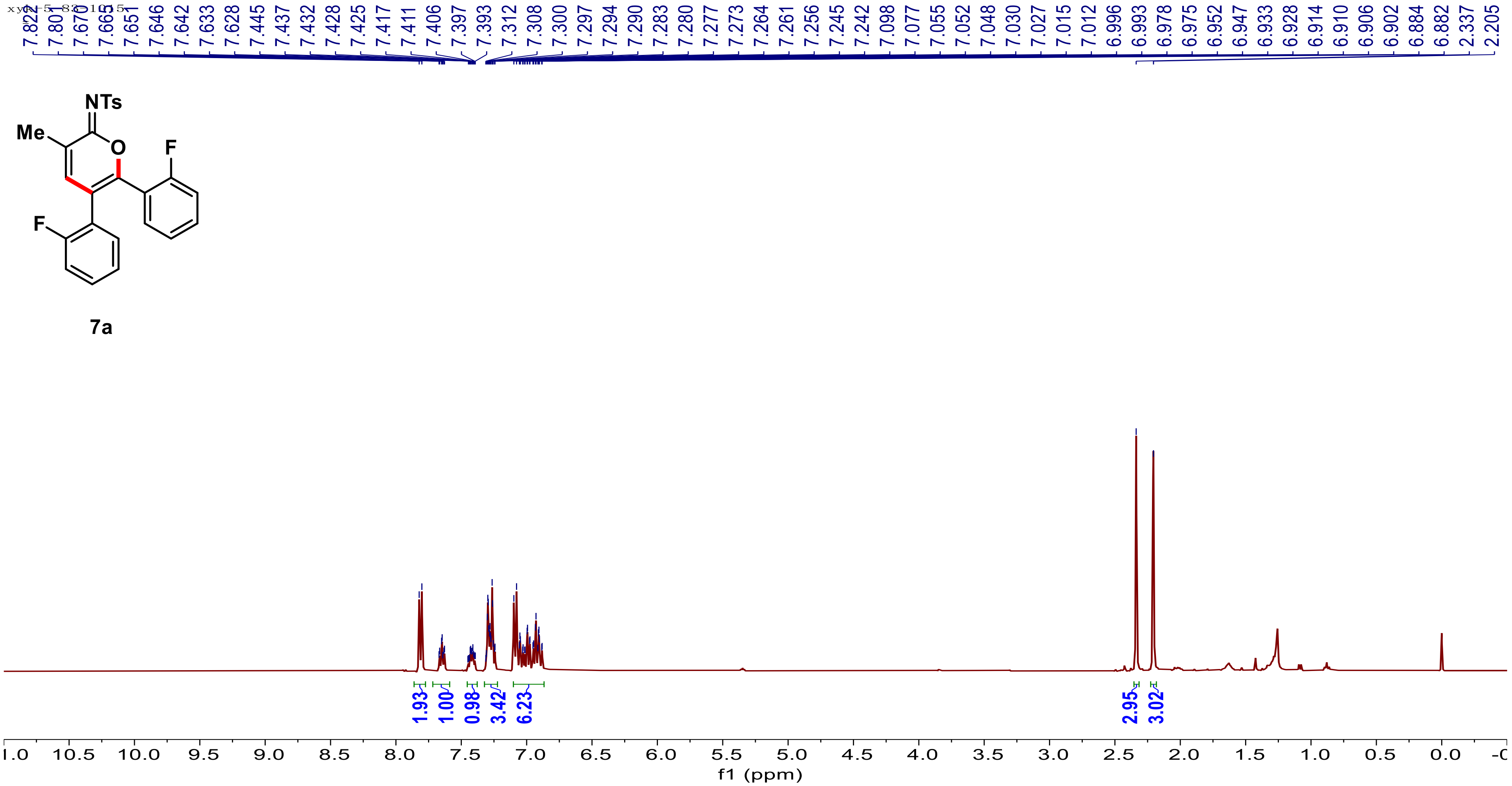

^{13}C NMR Spectrum of 7a at 25 °C ($CDCl_3$)

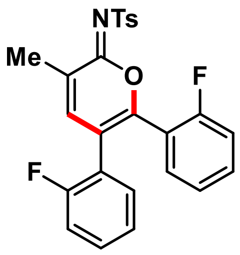

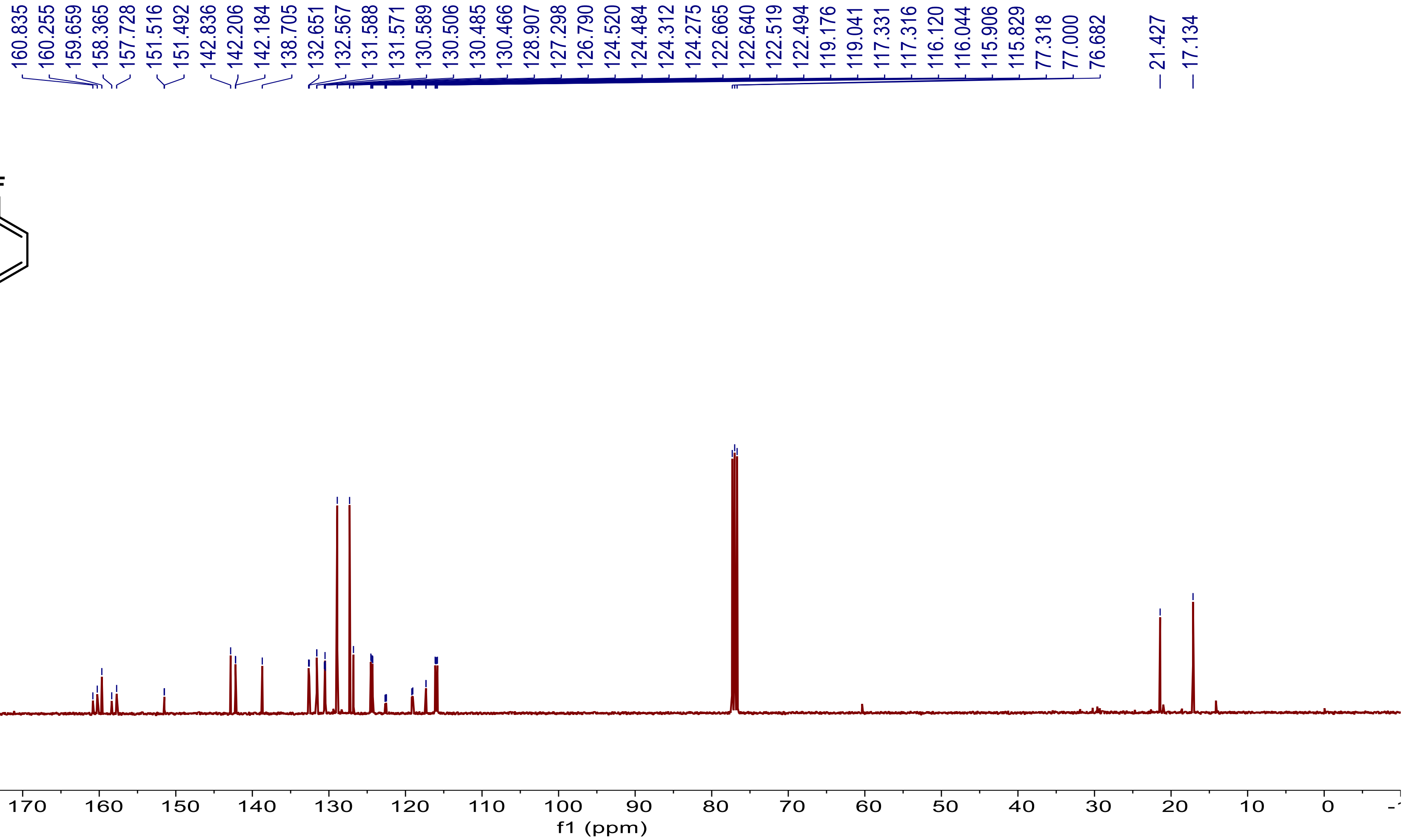

^{19}F NMR Spectrum of 7a at 25 °C ($CDCl_3$)

xyk-5-83-F-1015
STANDARD PROTON PARAMETERS
^{19}F NMR (376 MHz, $CDCl_3$) δ -110.27, -114.97.

-110.266
-114.972

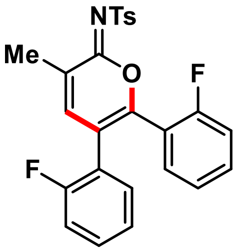

7a

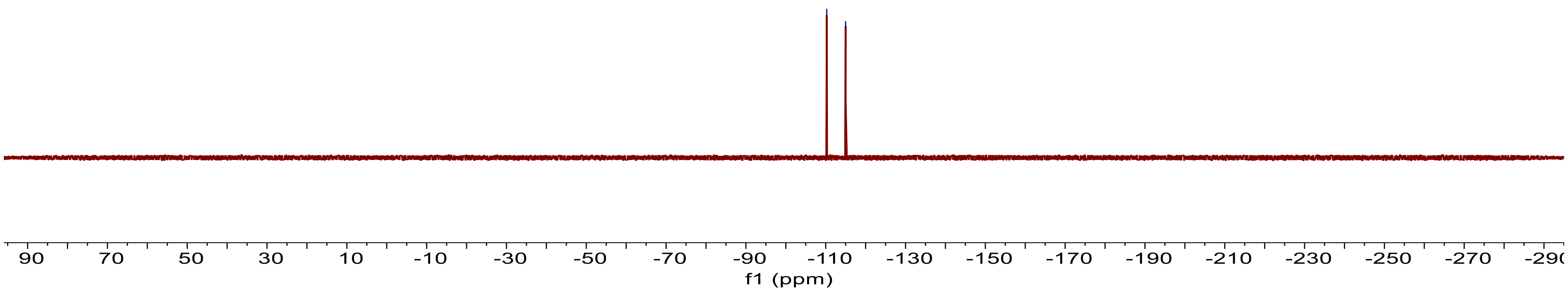

^{1}H NMR Spectrum of 7b at 25 ºC ($CDCl_3$)

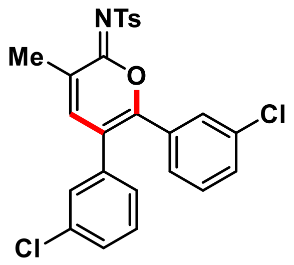

7b

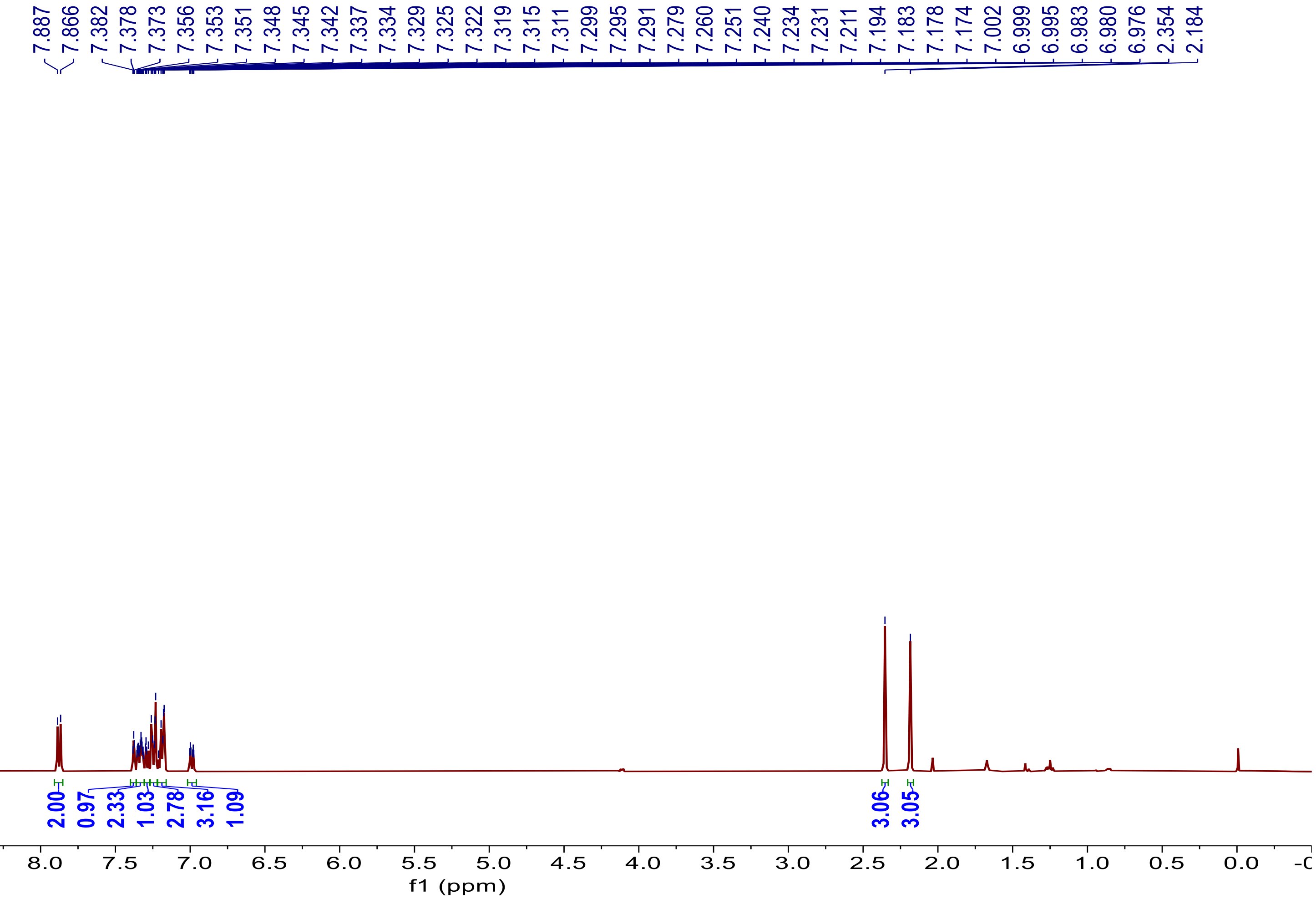

^{13}C NMR Spectrum of 7b at 25 ºC ($CDCl_3$)

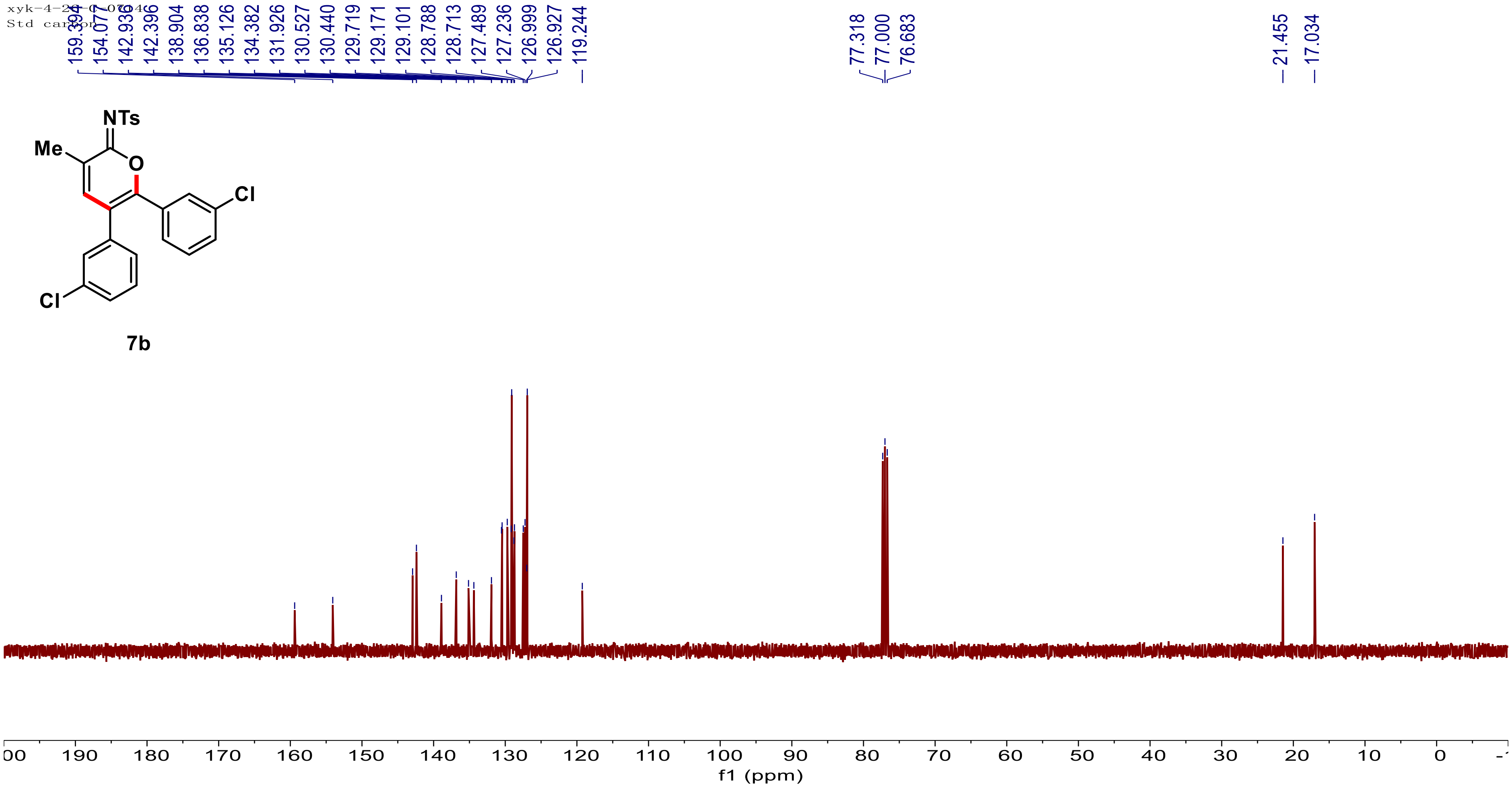

^{1}H NMR Spectrum of 7c at 25 ºC ($CDCl_3$)

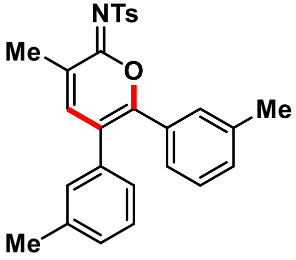

7c

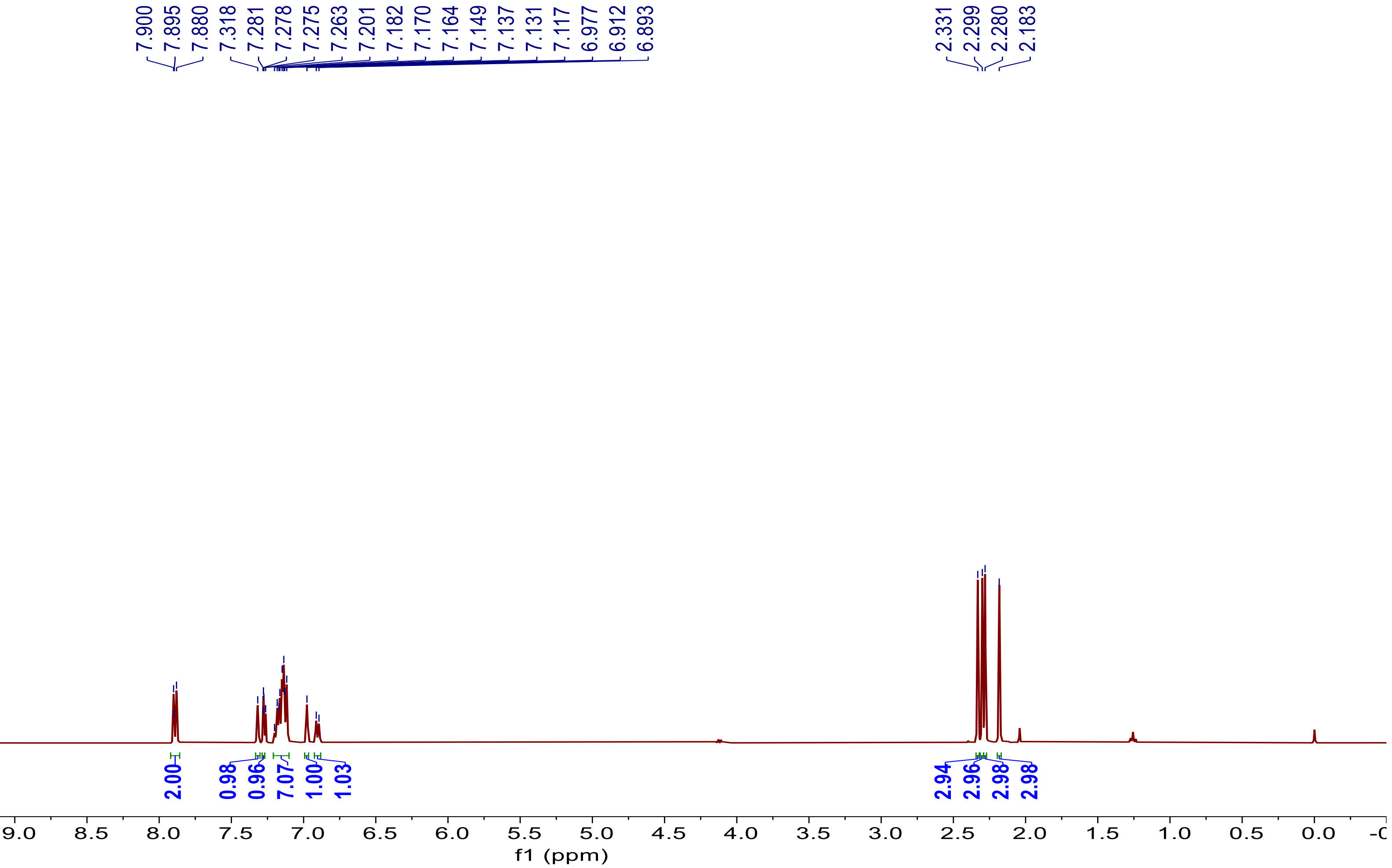

^{13}C NMR Spectrum of 7c at 25 ºC ($CDCl_3$)

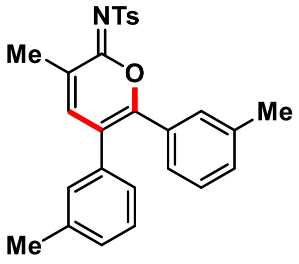

7c

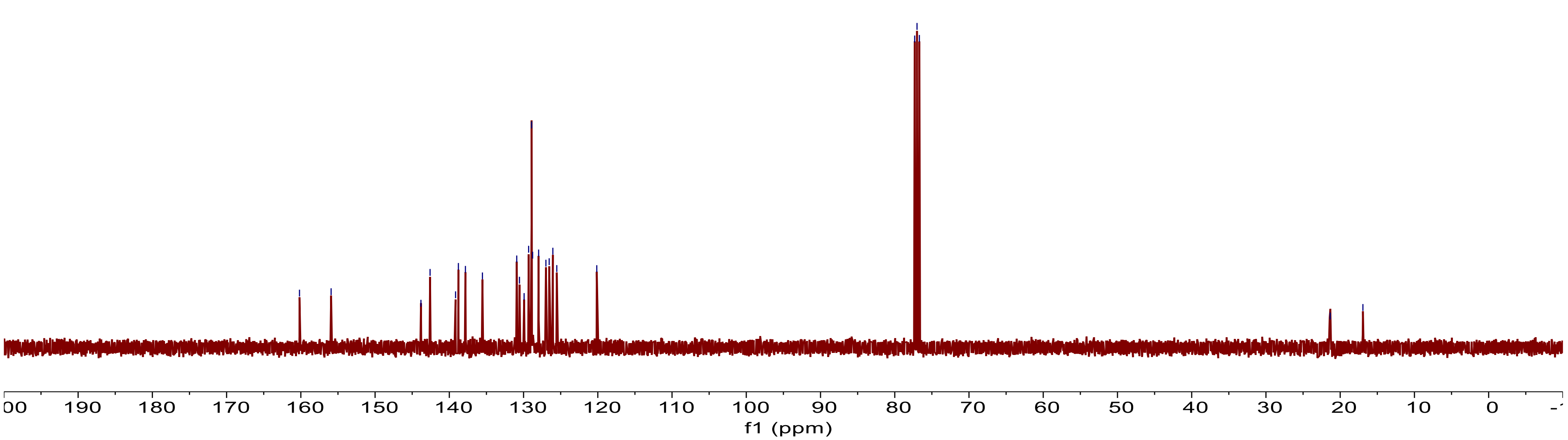

^{1}H NMR Spectrum of 7d at 25 ºC ($CDCl_3$)

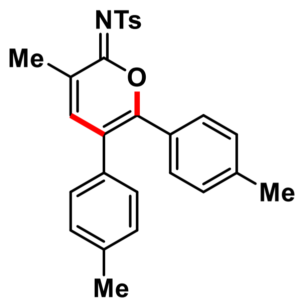

7d

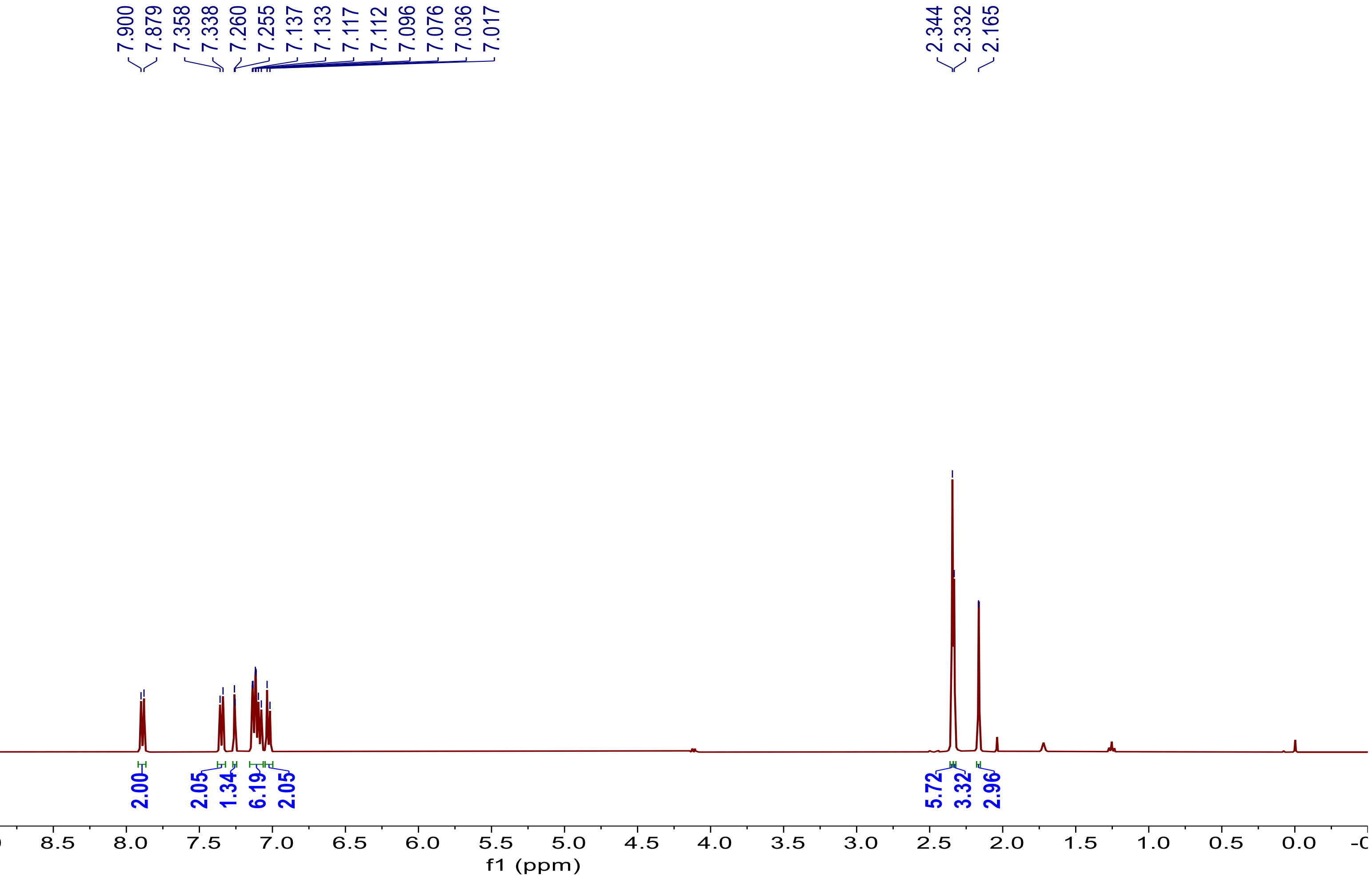

^{13}C NMR Spectrum of 7d at 25 ºC ($CDCl_3$)

xyk-4-6-C-0627

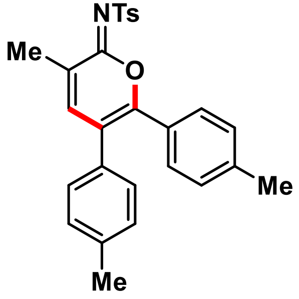

7d

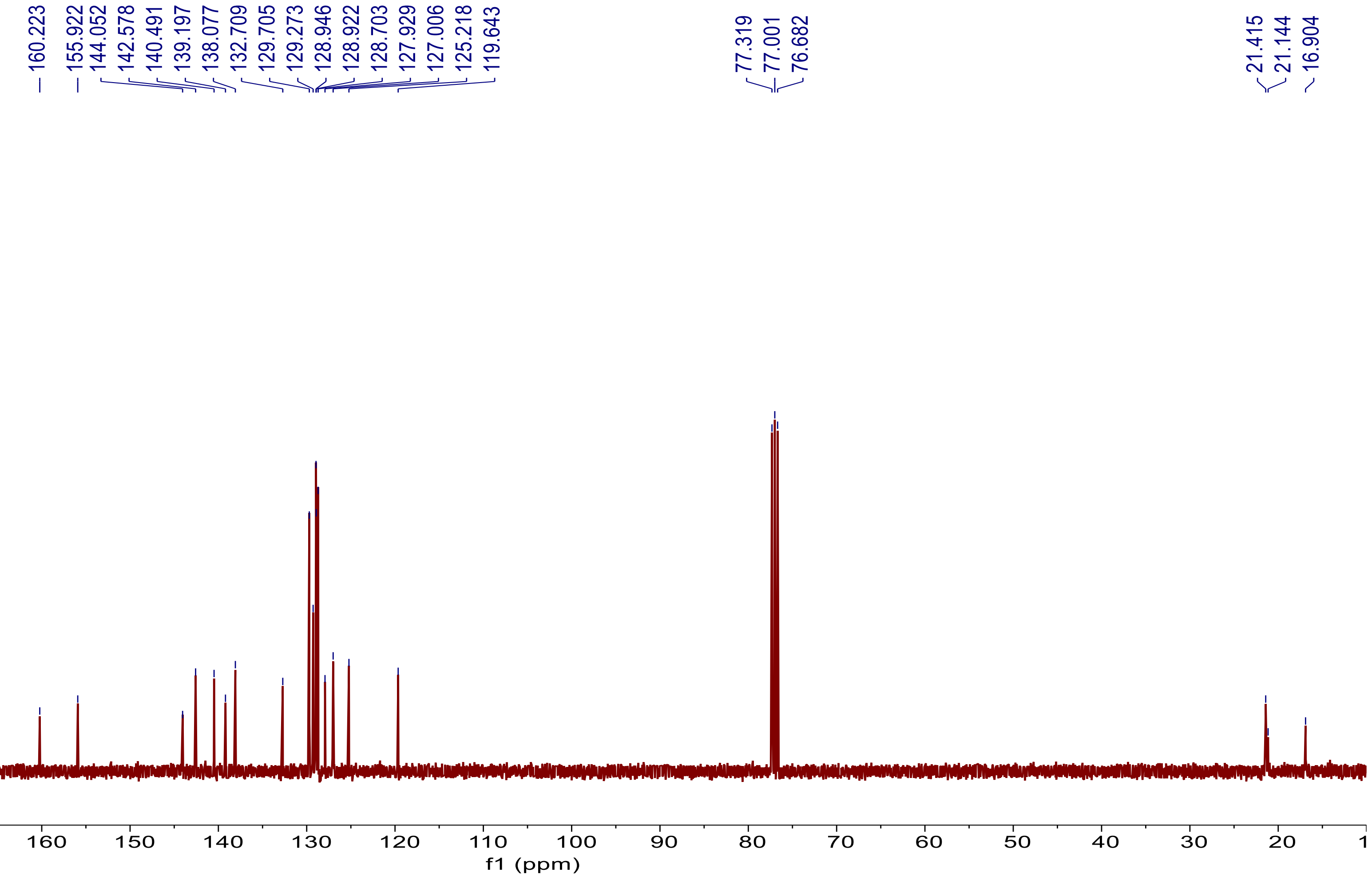

^{1}H NMR Spectrum of 7e at 25 ^{o}C ($CDCl_3$)

xyk-4-22-H-0627

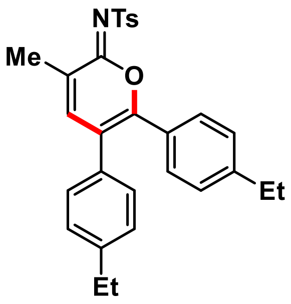

7e

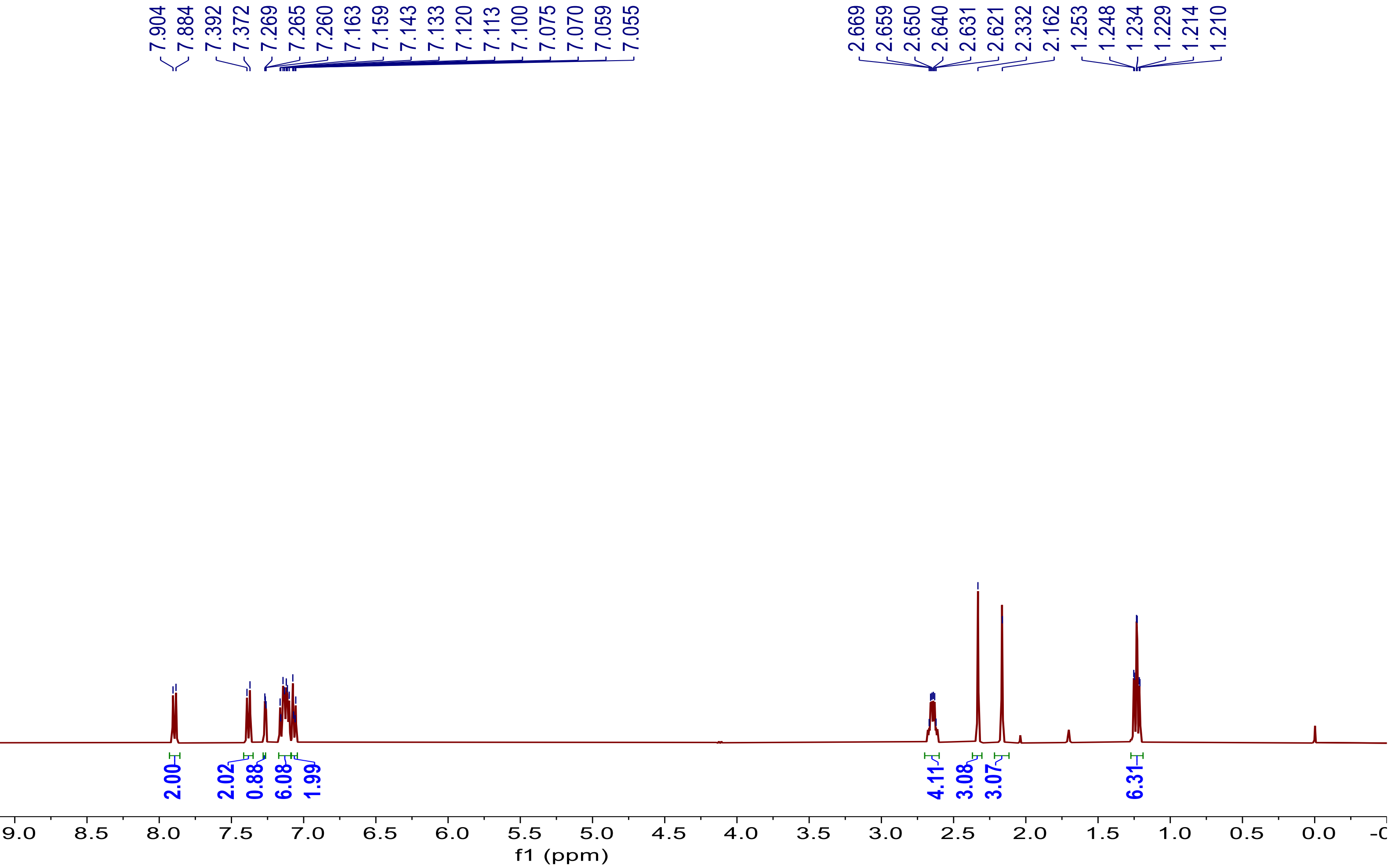

^{13}C NMR Spectrum of 7e at 25 ºC ($CDCl_3$)

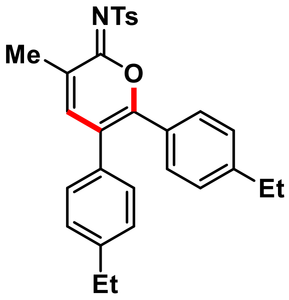

7e

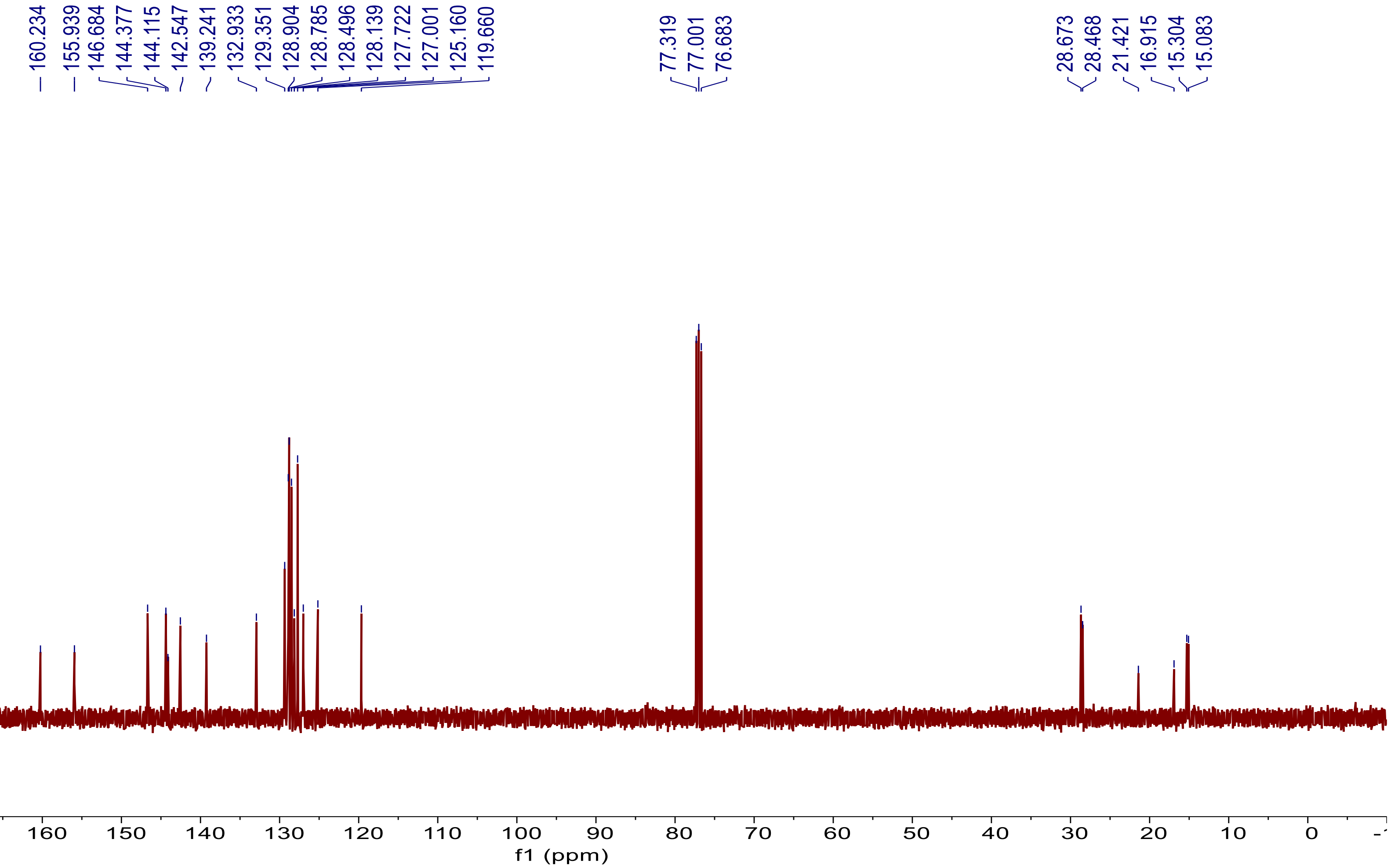

^{1}H NMR Spectrum of 7f at 25 ºC ($CDCl_3$)

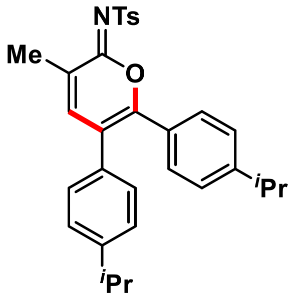

7f

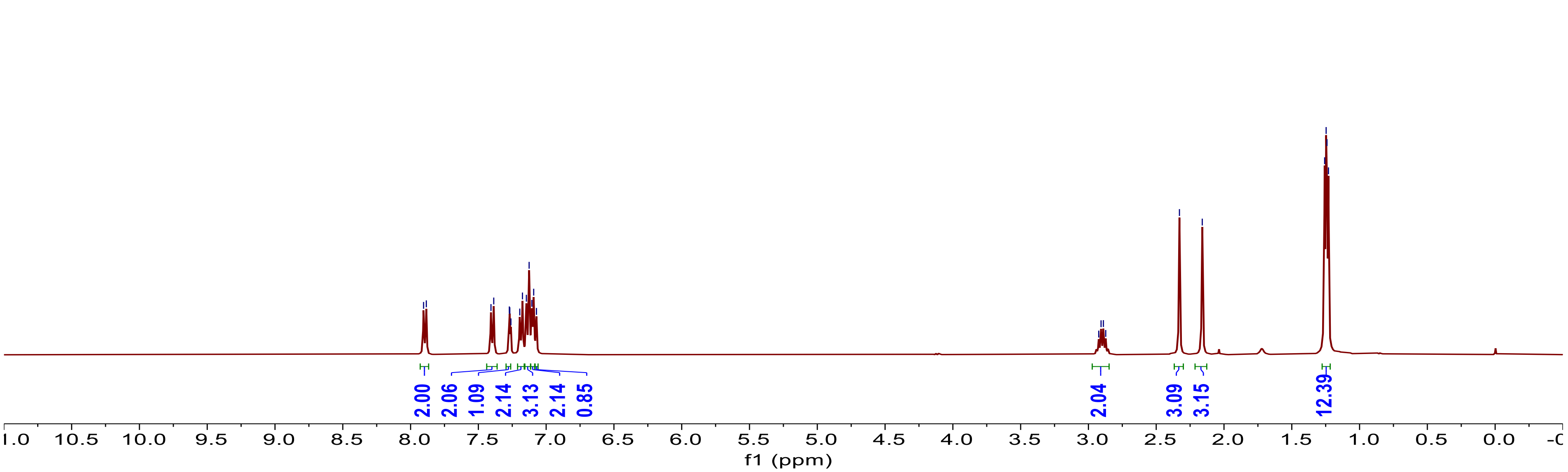

^{13}C NMR Spectrum of 7f at 25 °C ($CDCl_3$)

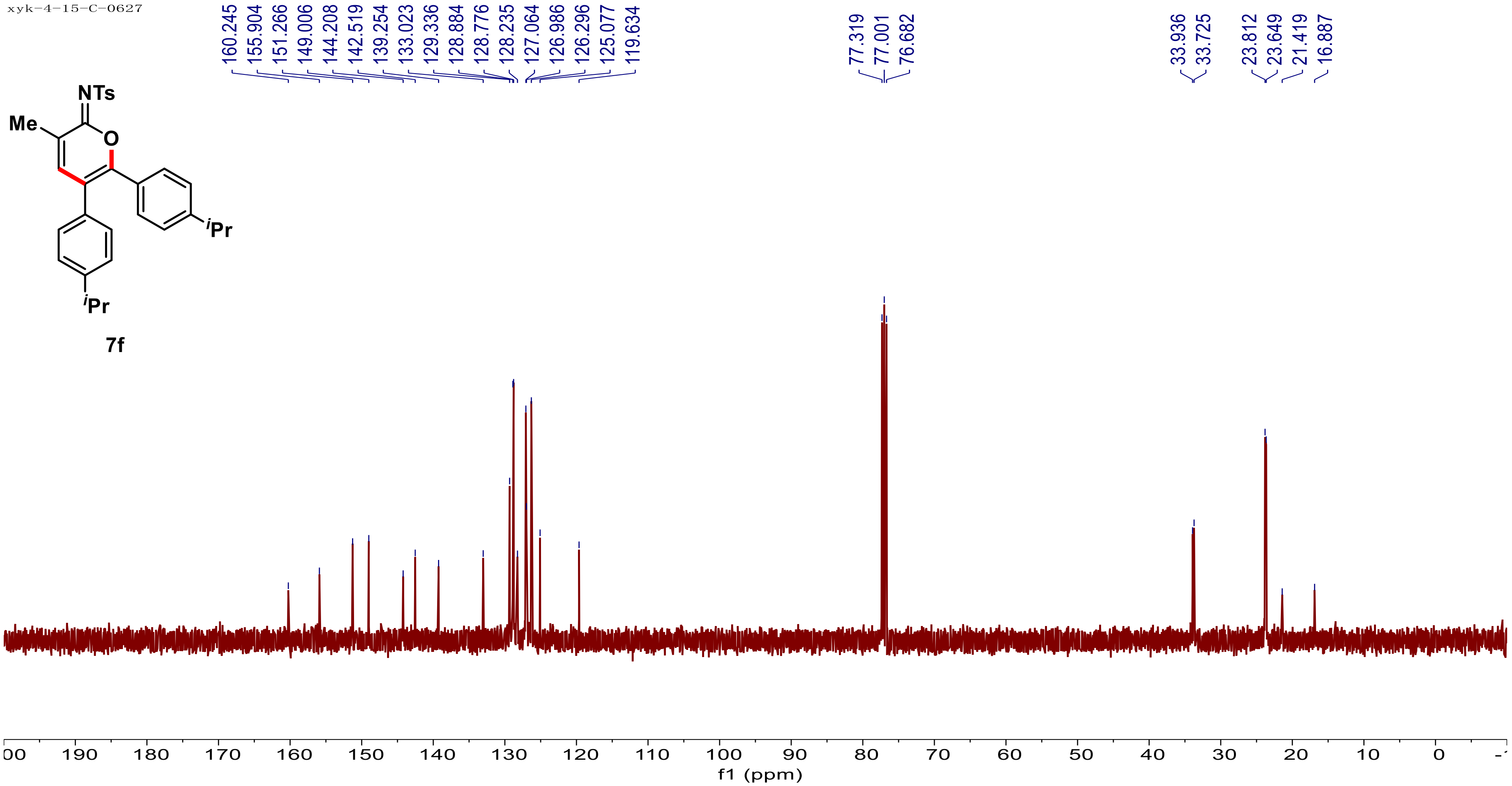

^{1}H NMR Spectrum of 7g at 25 °C ($CDCl_3$)

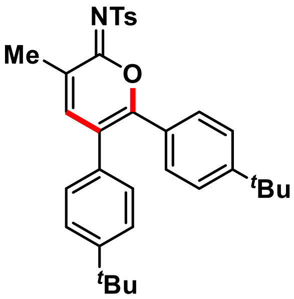

7g

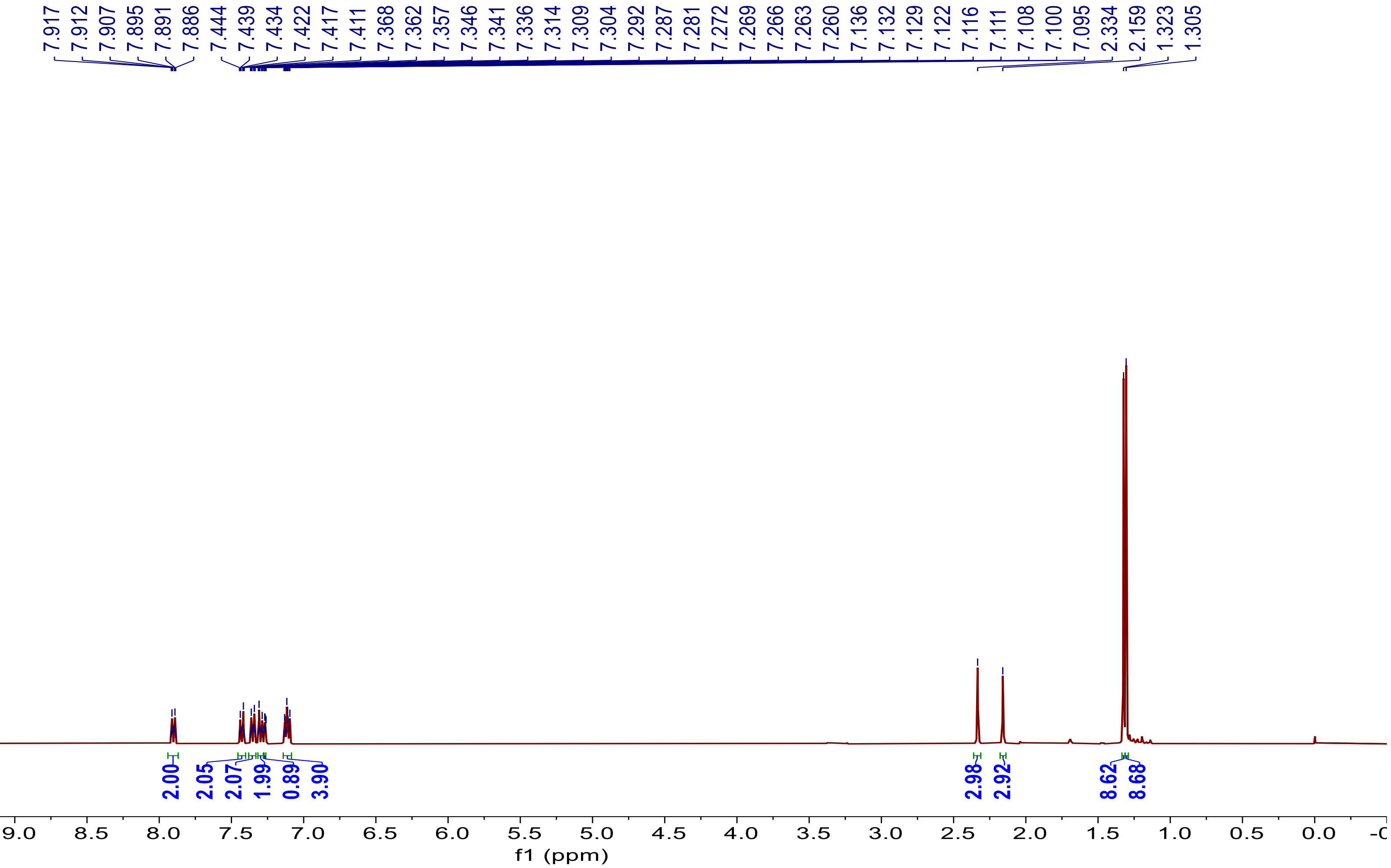

^{13}C NMR Spectrum of 7g at 25 ºC ($CDCl_3$)

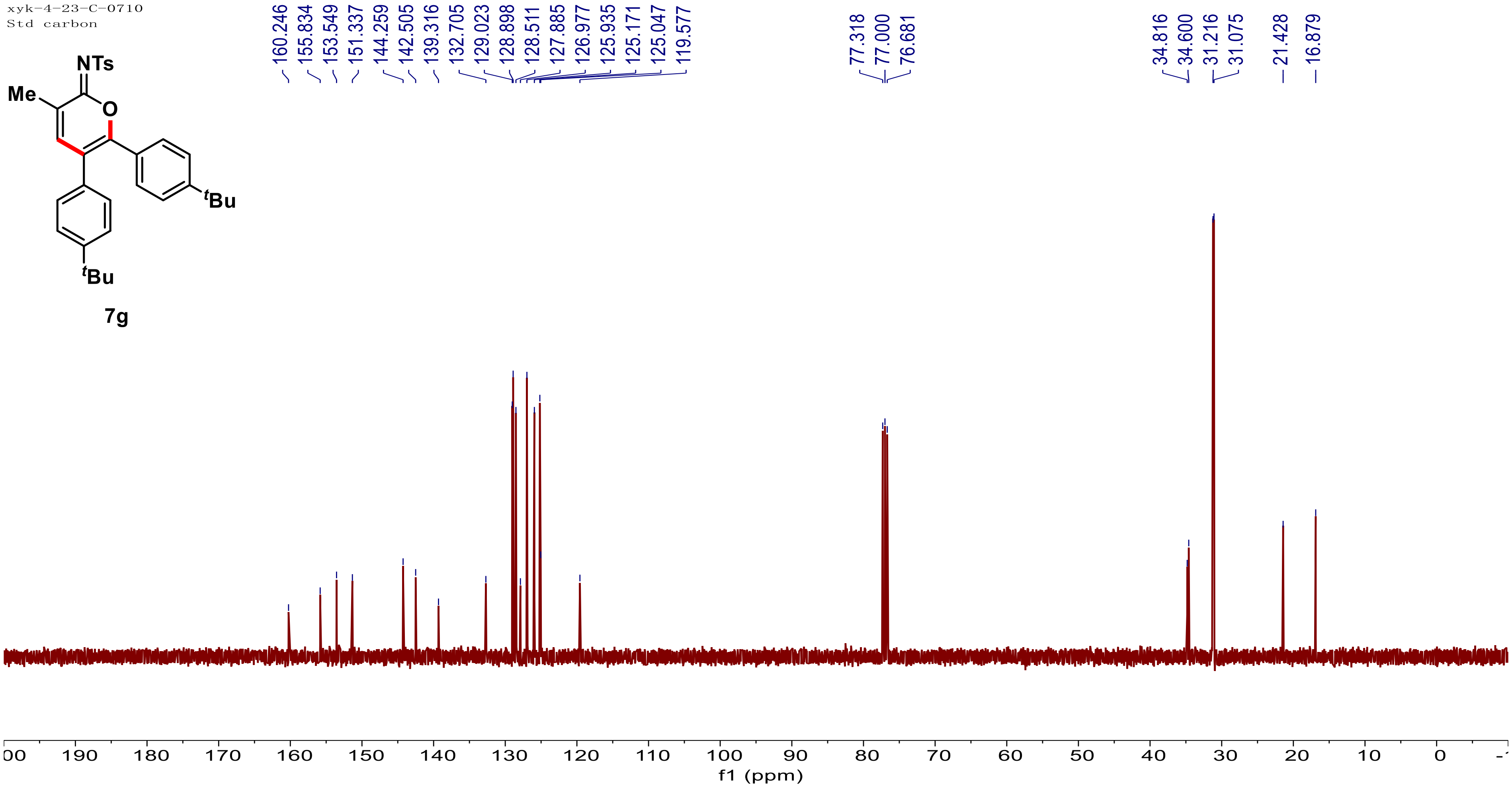

^{1}H NMR Spectrum of 7h at 25 ºC ($CDCl_3$)

xyk-4-21-H-0711

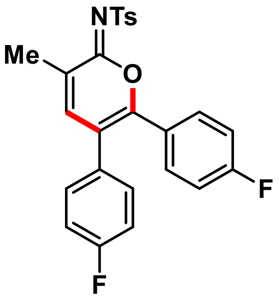

7h

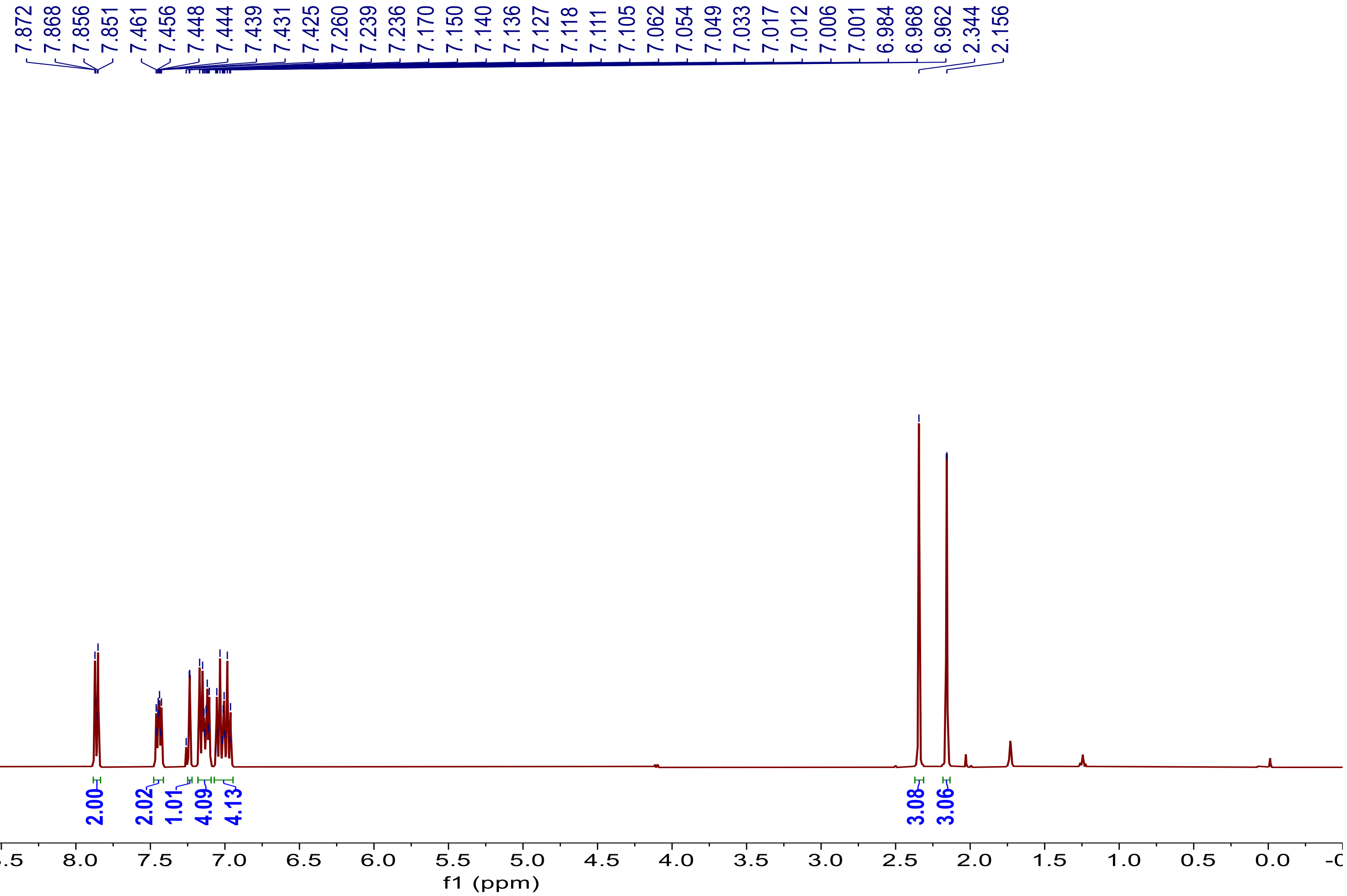

^{13}C NMR Spectrum of 7h at 25 °C ($CDCl_3$)

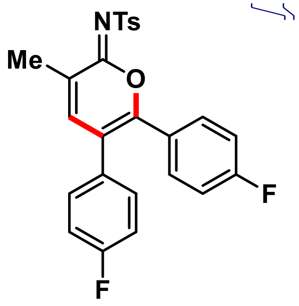

7h

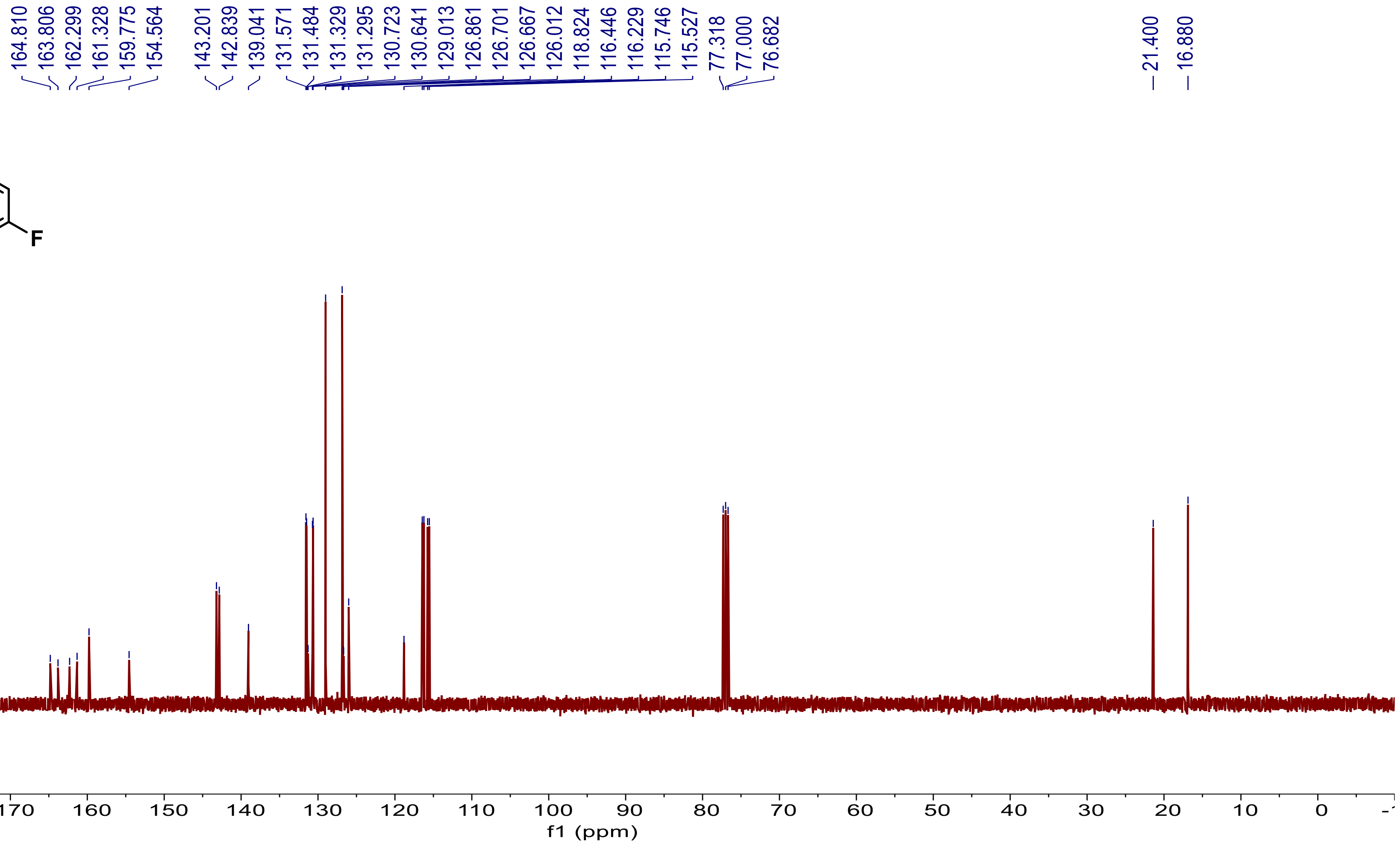

^{19}F NMR Spectrum of 7h at 25 ºC ($CDCl_3$)

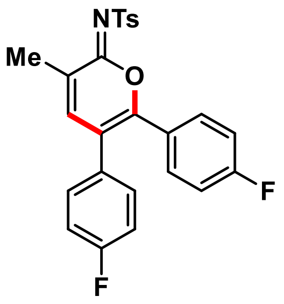

7h

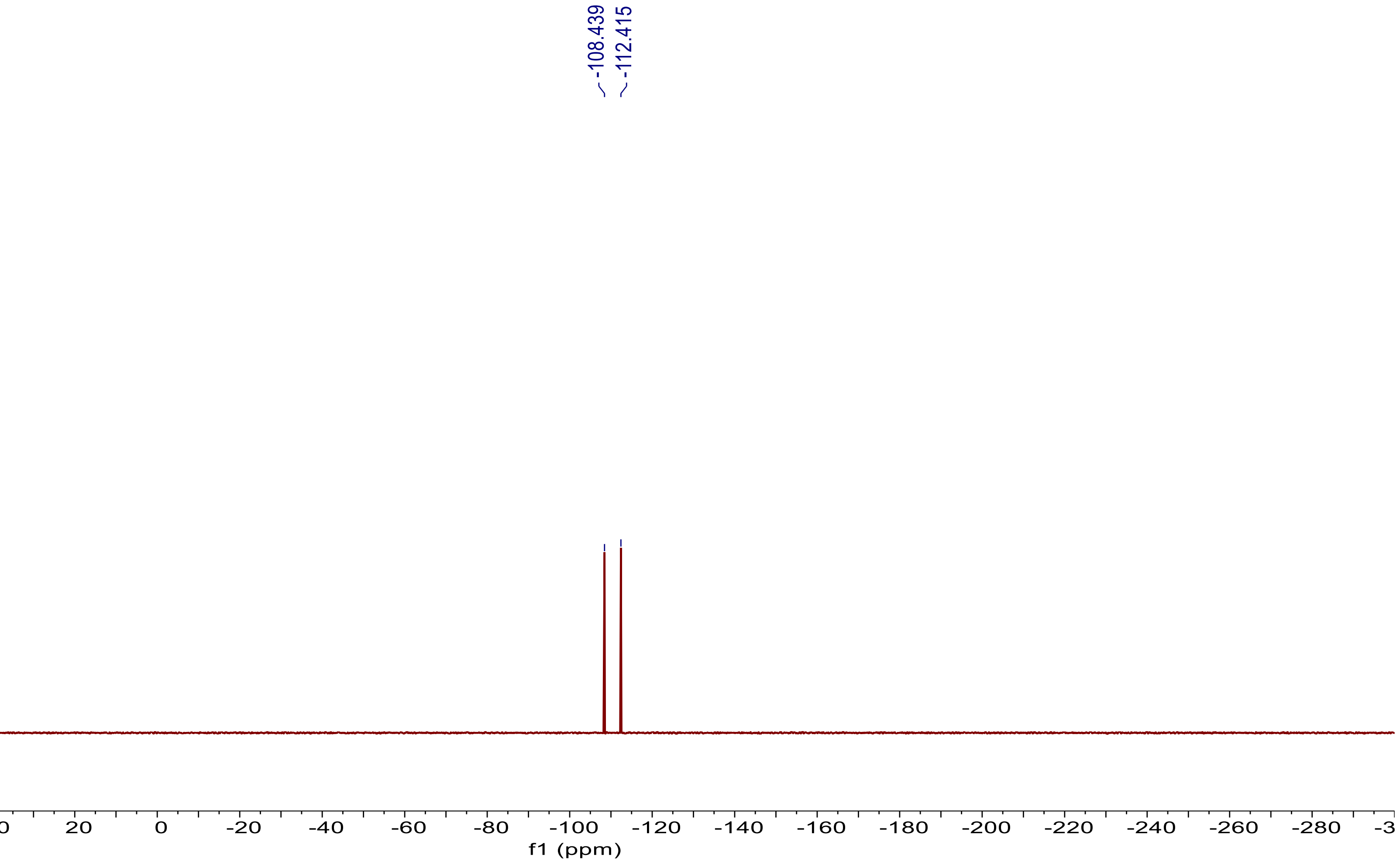

^{1}H NMR Spectrum of 7i at 25 ºC ($CDCl_3$)

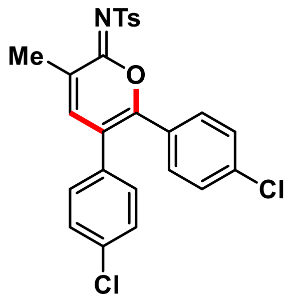

7i

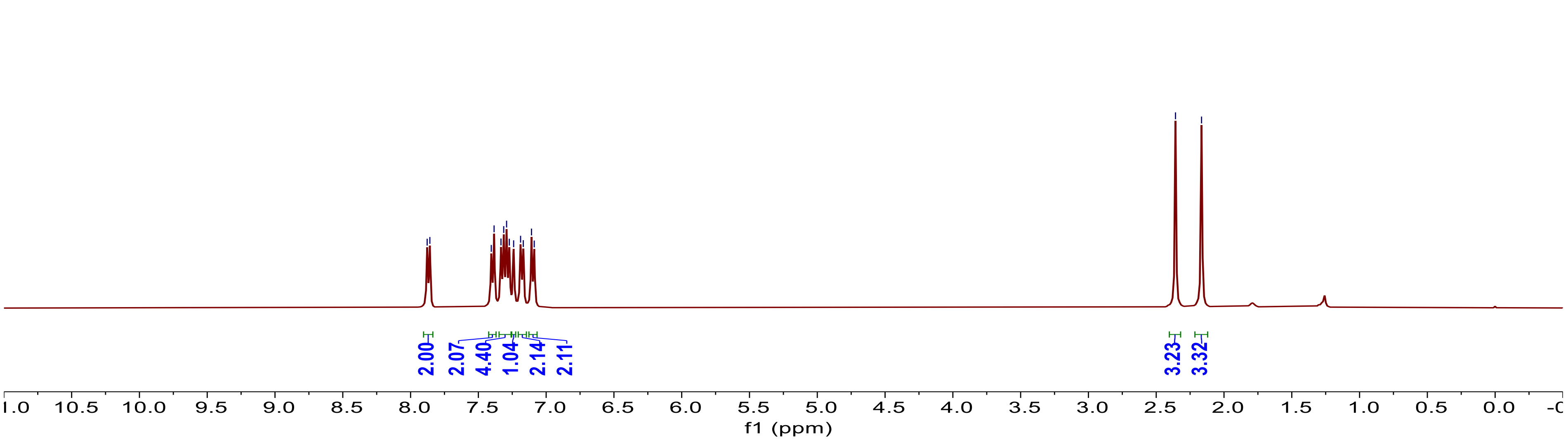

^{13}C NMR Spectrum of 7i at 25 °C ($CDCl_3$)

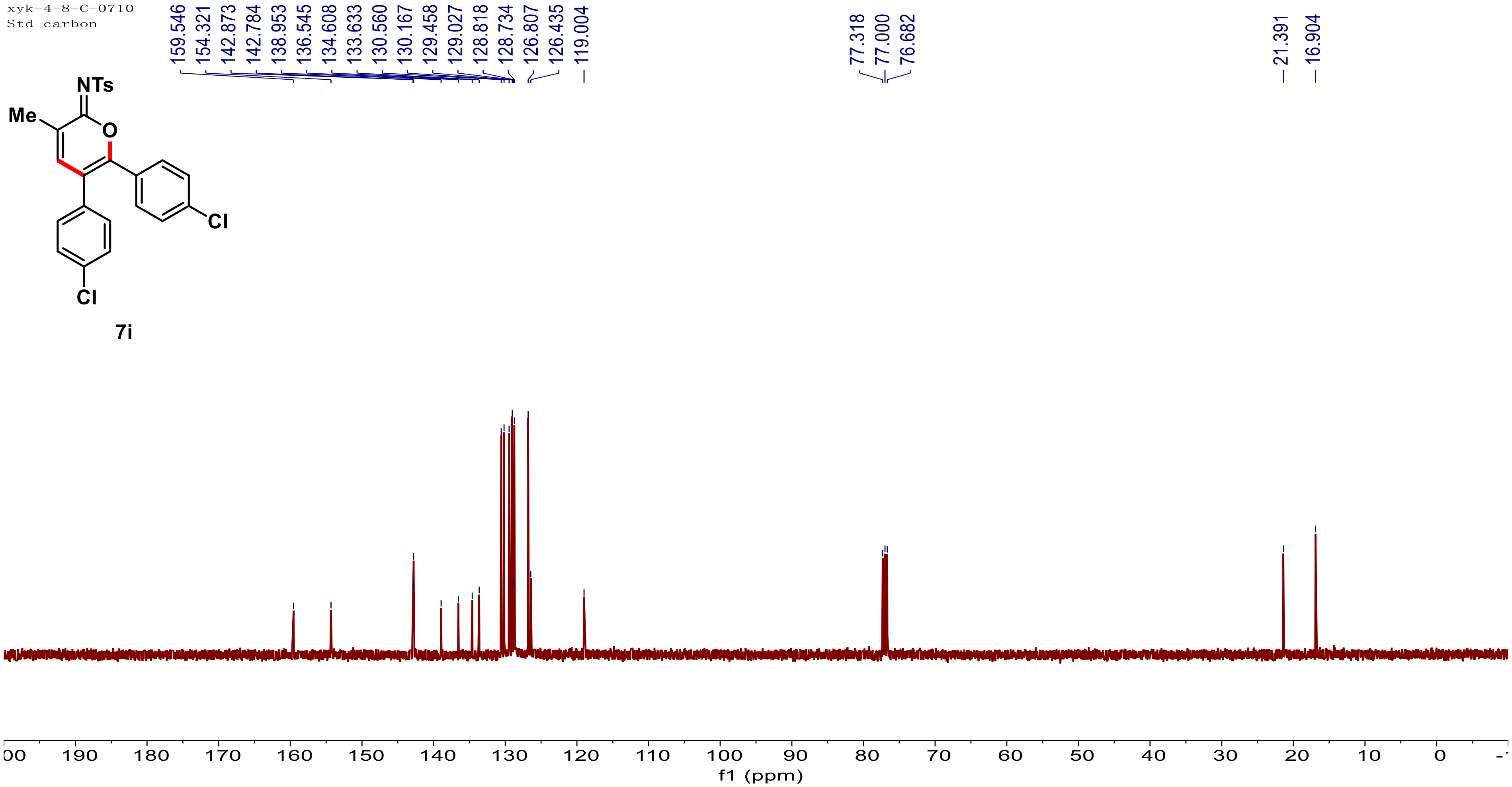

1H NMR Spectrum of 7j at 25 ºC ($CDCl_3$)

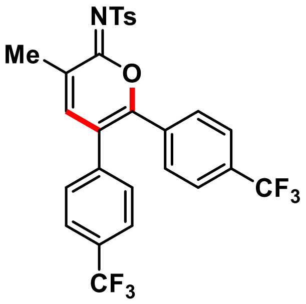

7j

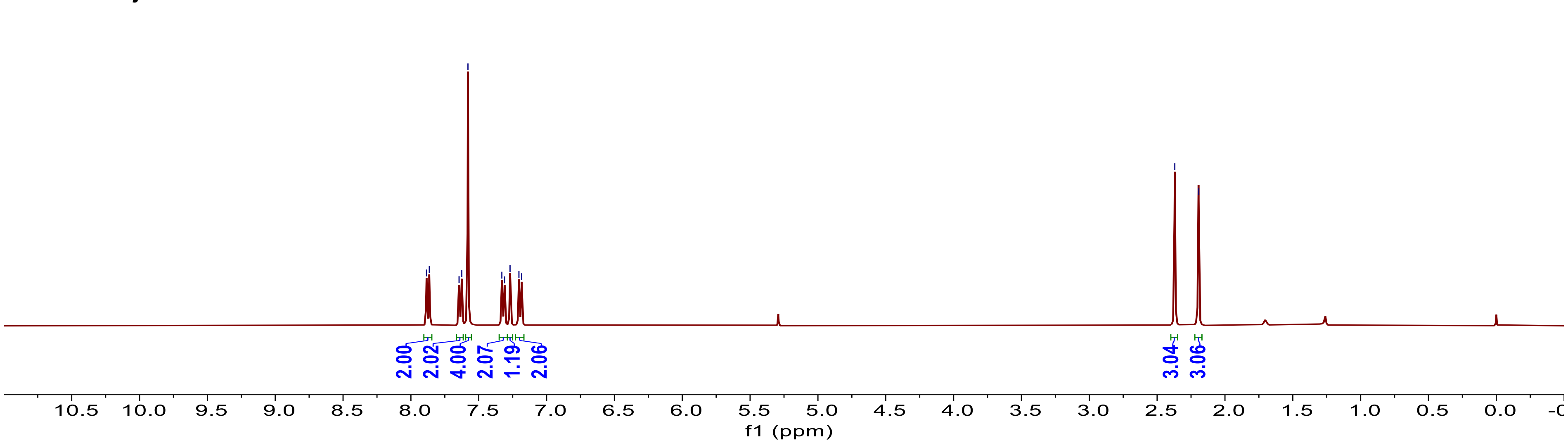

^{13}C NMR Spectrum of 7j at 25 °C ($CDCl_3$)

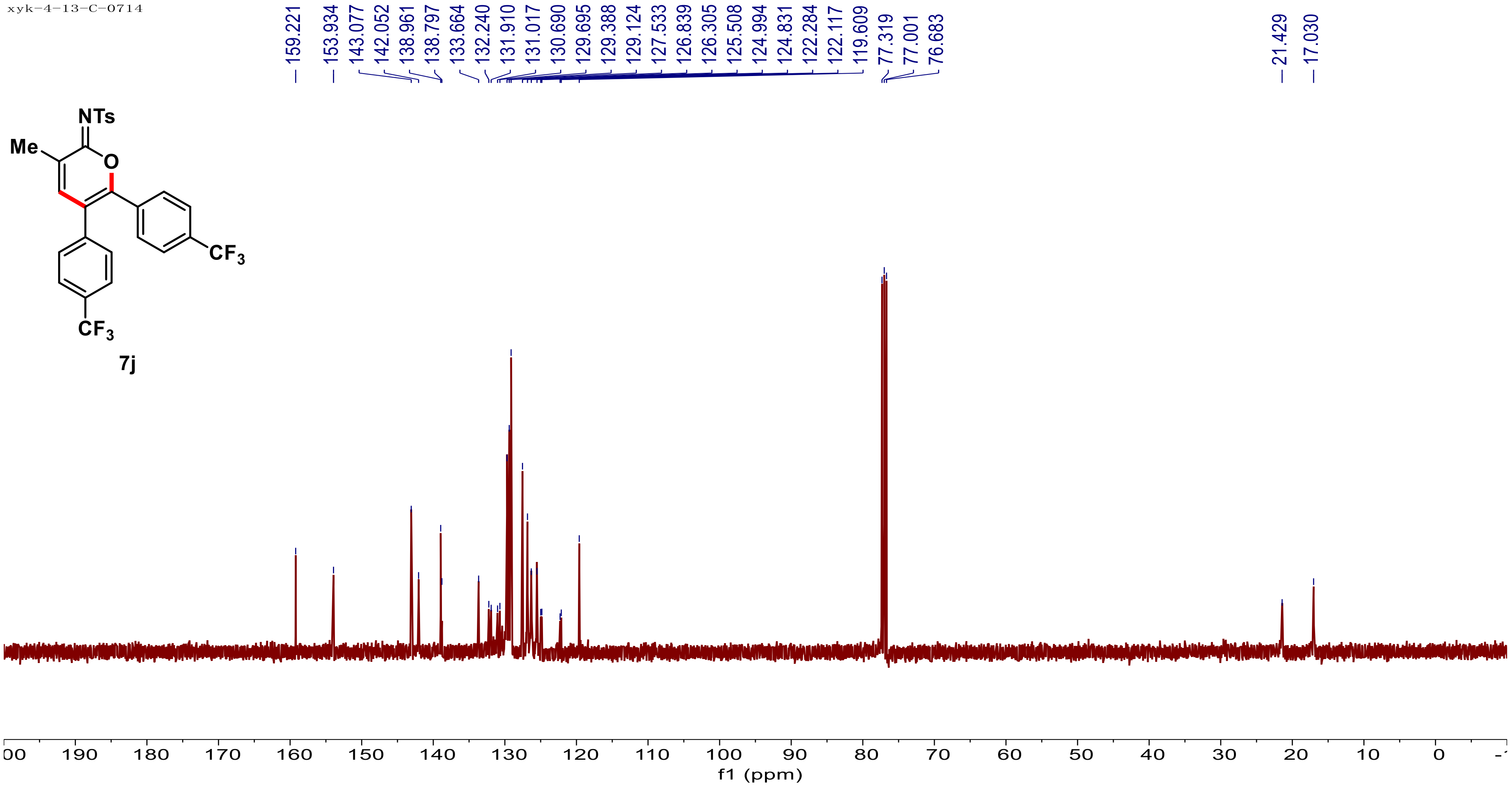

^{19}F NMR Spectrum of 3ak at 25 ^{o}C ($CDCl_3$)

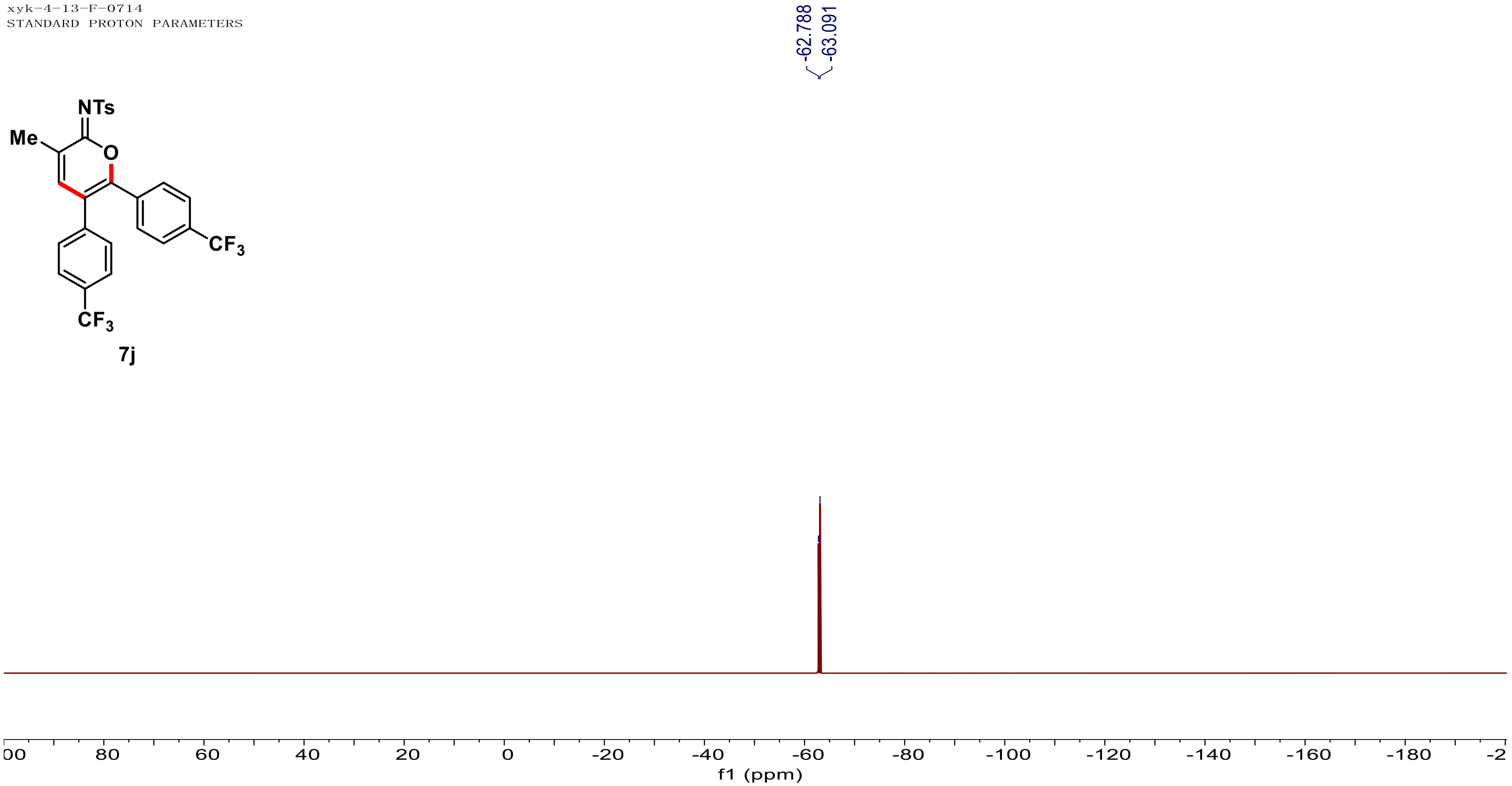

^{1}H NMR Spectrum of 7k at 25 ºC ($CDCl_3$)

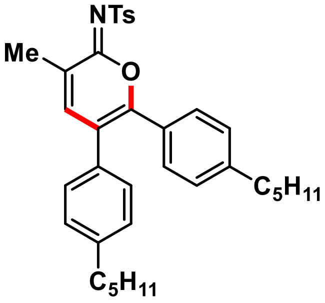

7k

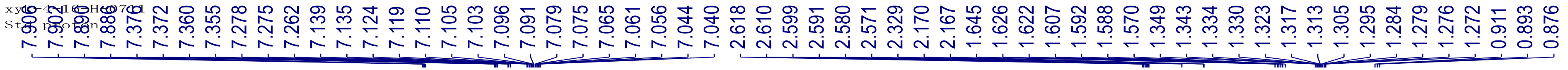
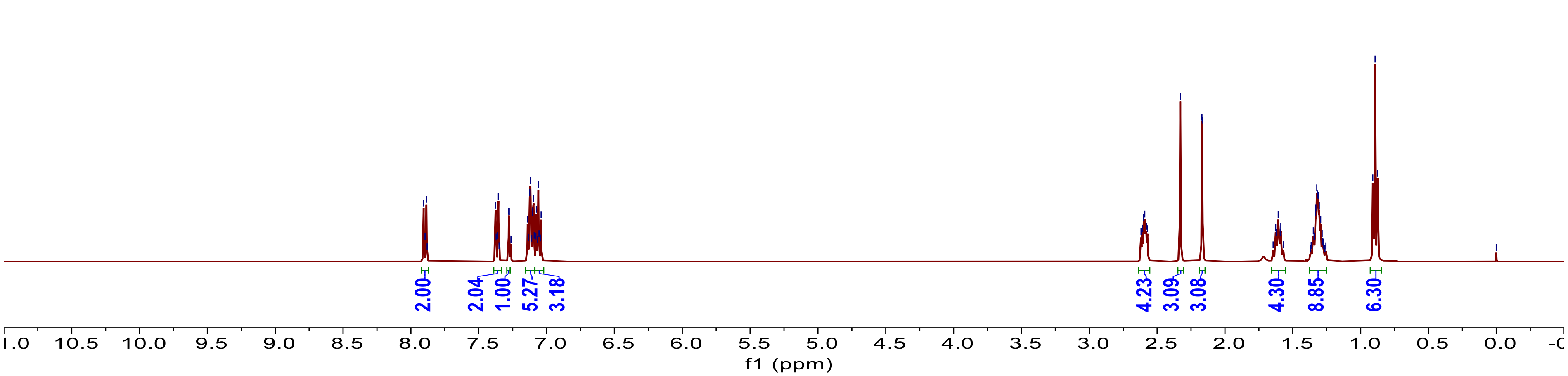

^{13}C NMR Spectrum of 7k at 25 °C ($CDCl_3$)

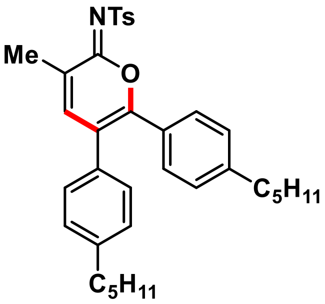

7k

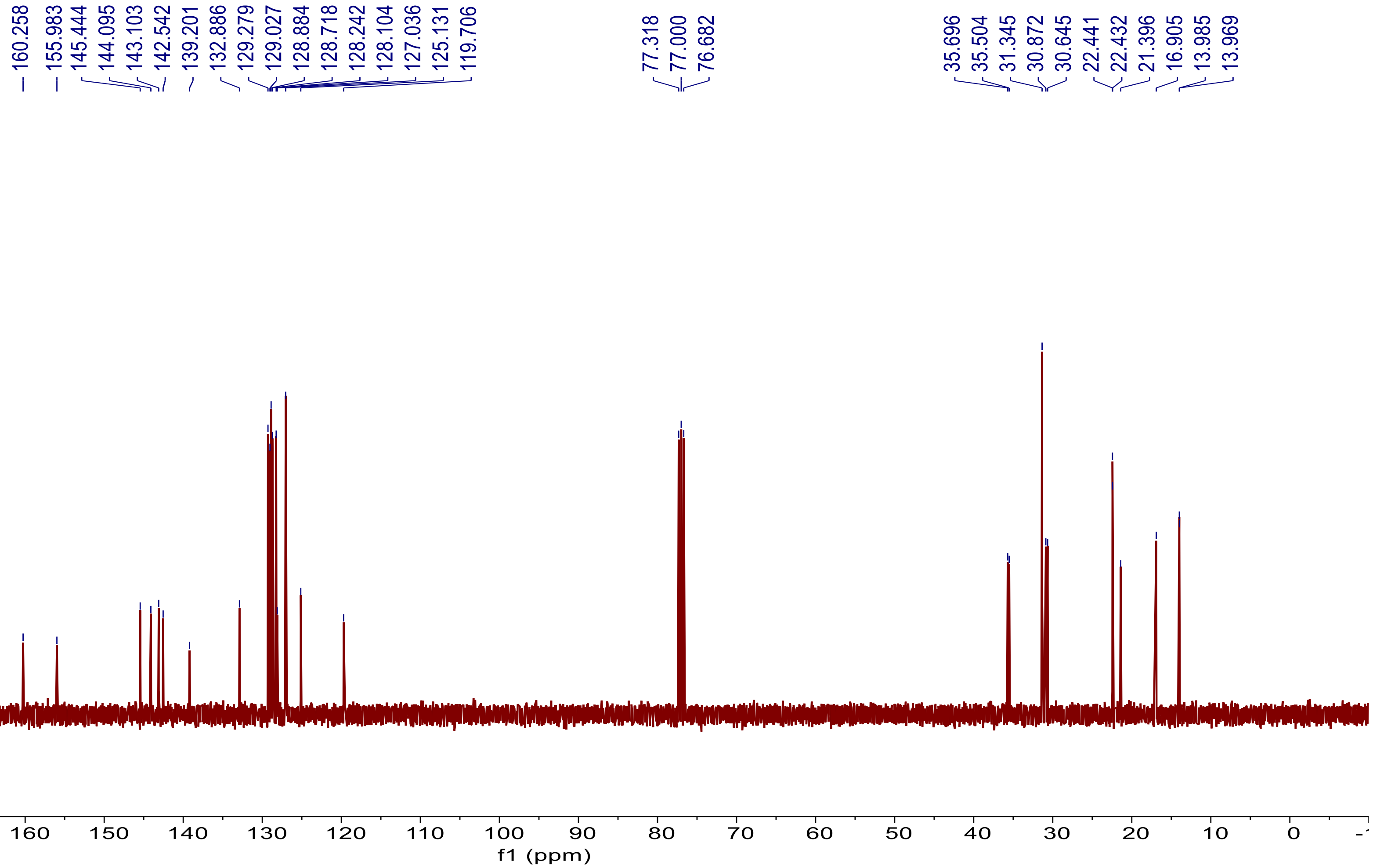

^{1}H NMR Spectrum of 7l at 25 ºC ($CDCl_3$)

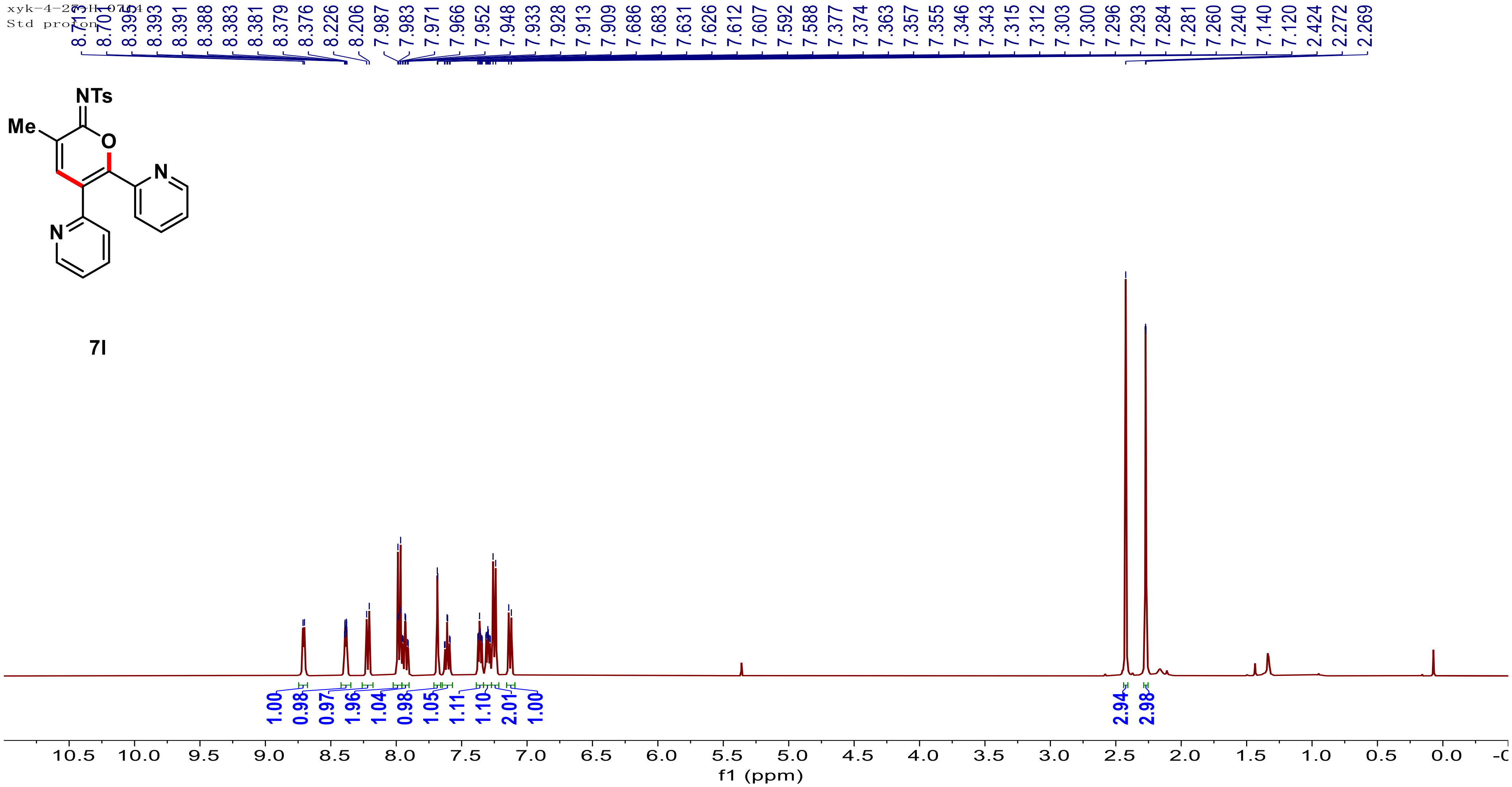

7l

^{13}C NMR Spectrum of 7l at 25 °C ($CDCl_3$)

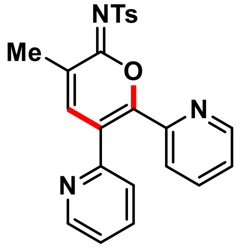

7l

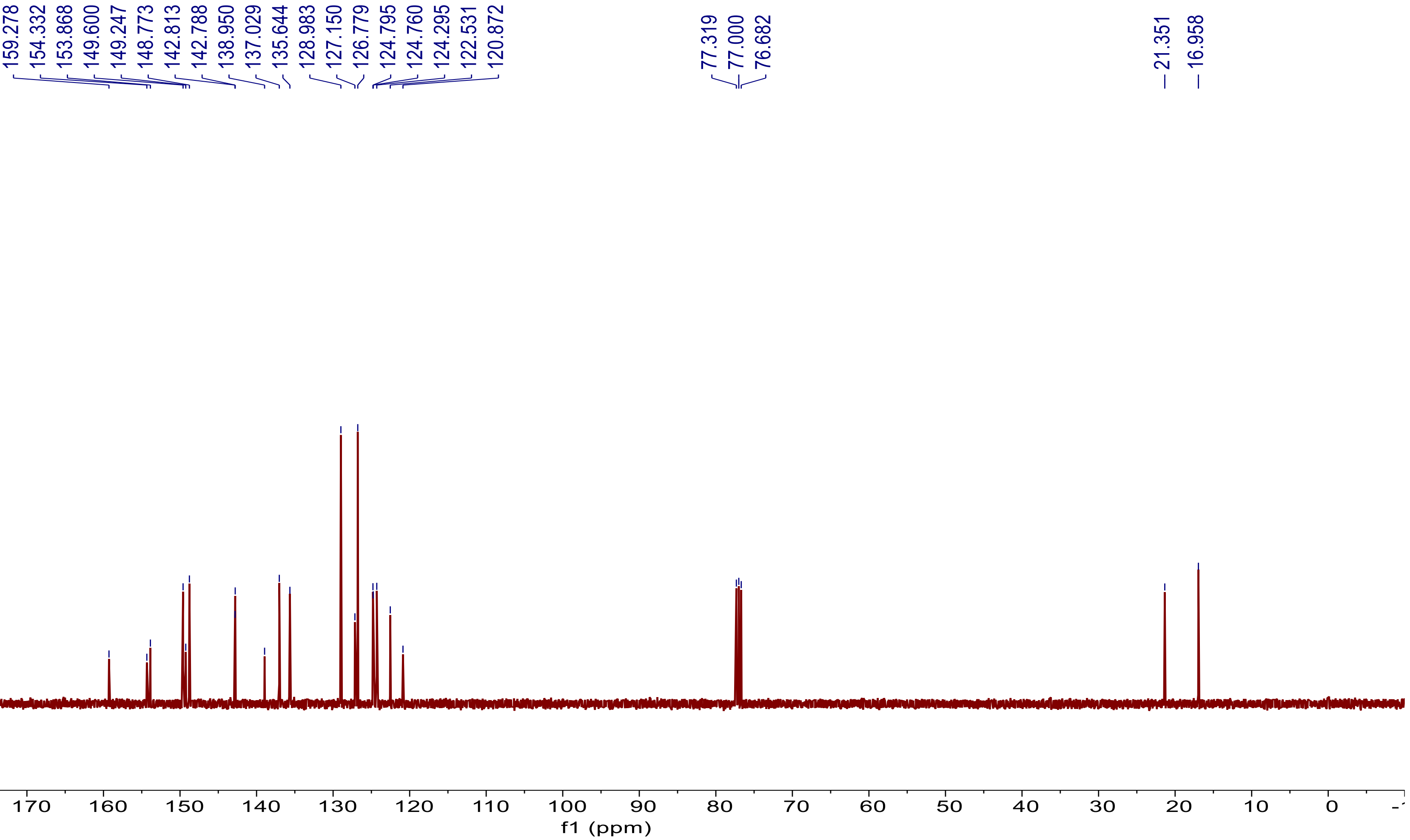

^{1}H NMR Spectrum of 7m at 25 ºC ($CDCl_3$)

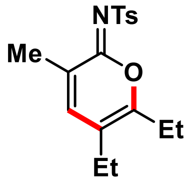

7m

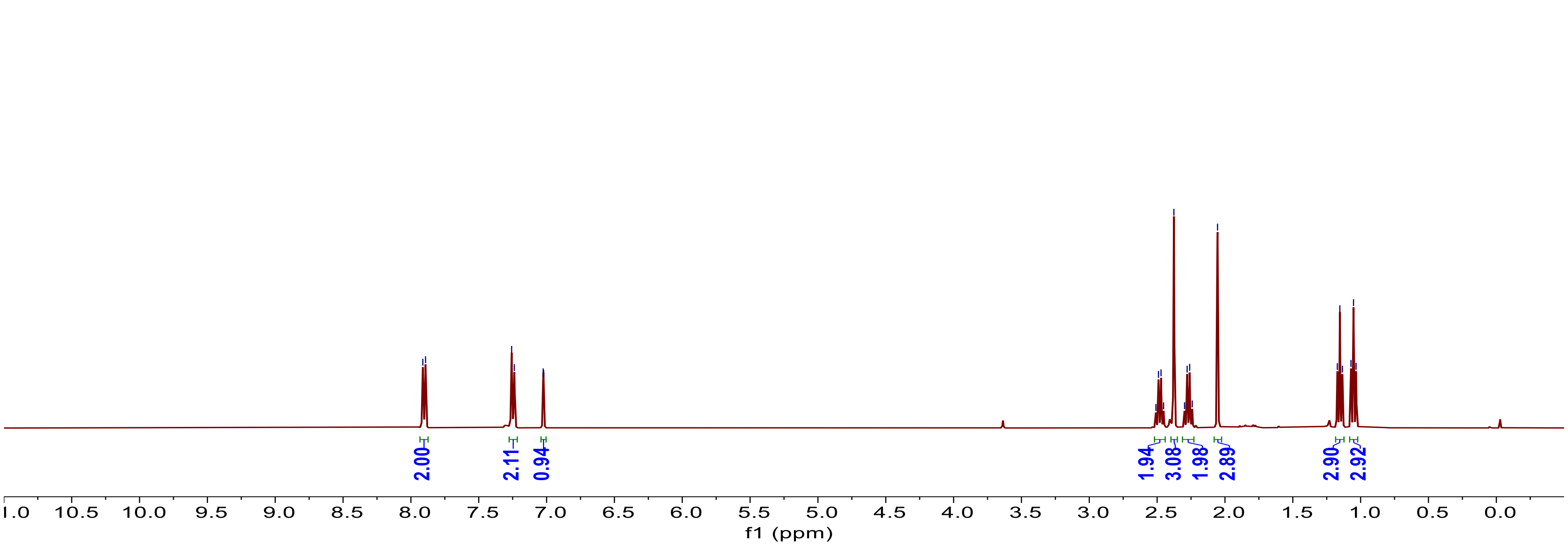

^{13}C NMR Spectrum of 7m at 25 ºC ($CDCl_3$)

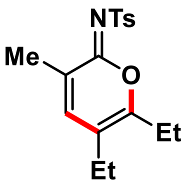

7m

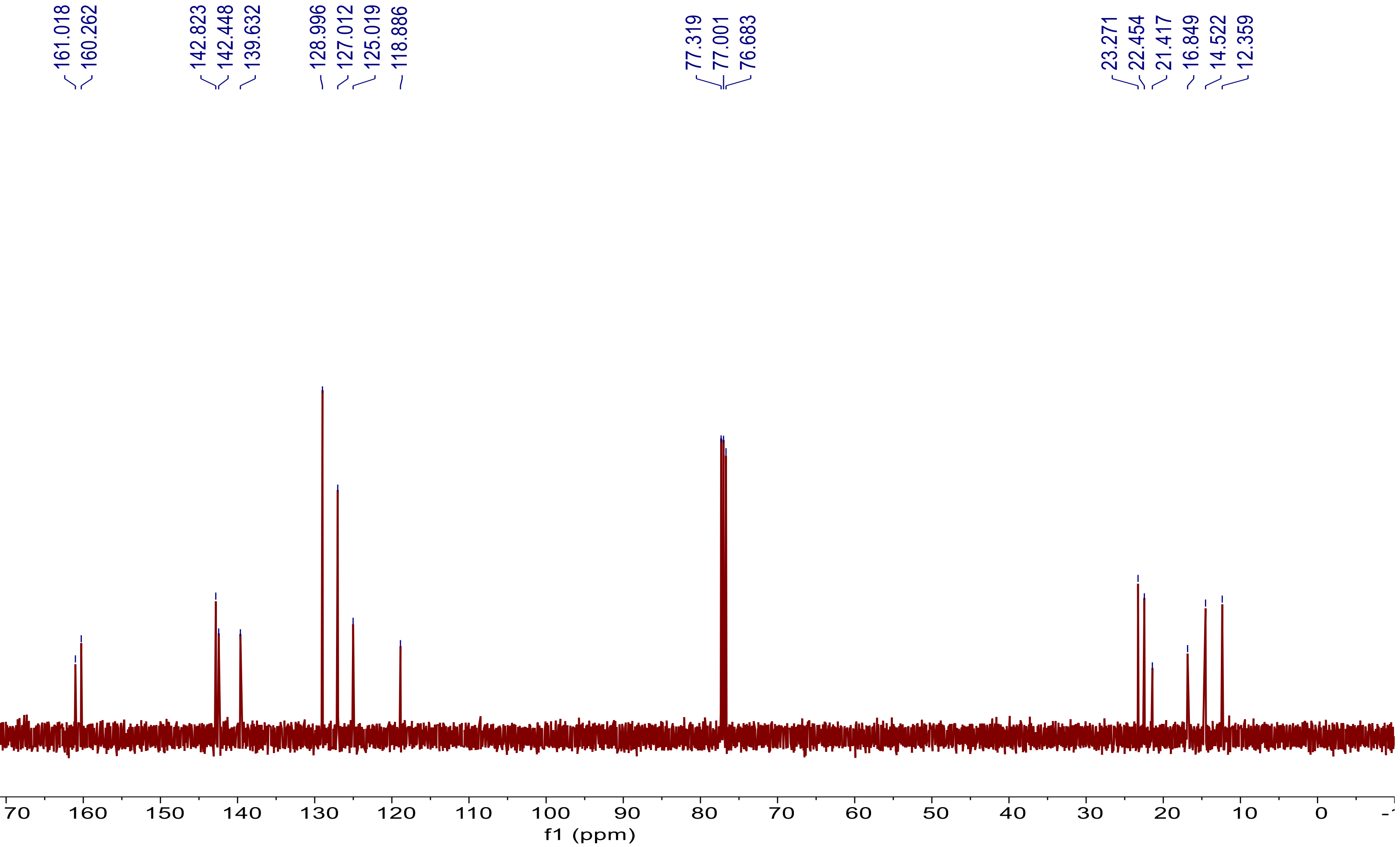

^{1}H NMR Spectrum of 7n at 25 ºC ($CDCl_3$)

xyk-4-19-H-0716
Std proton

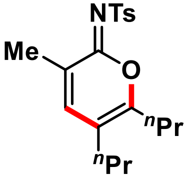

7n

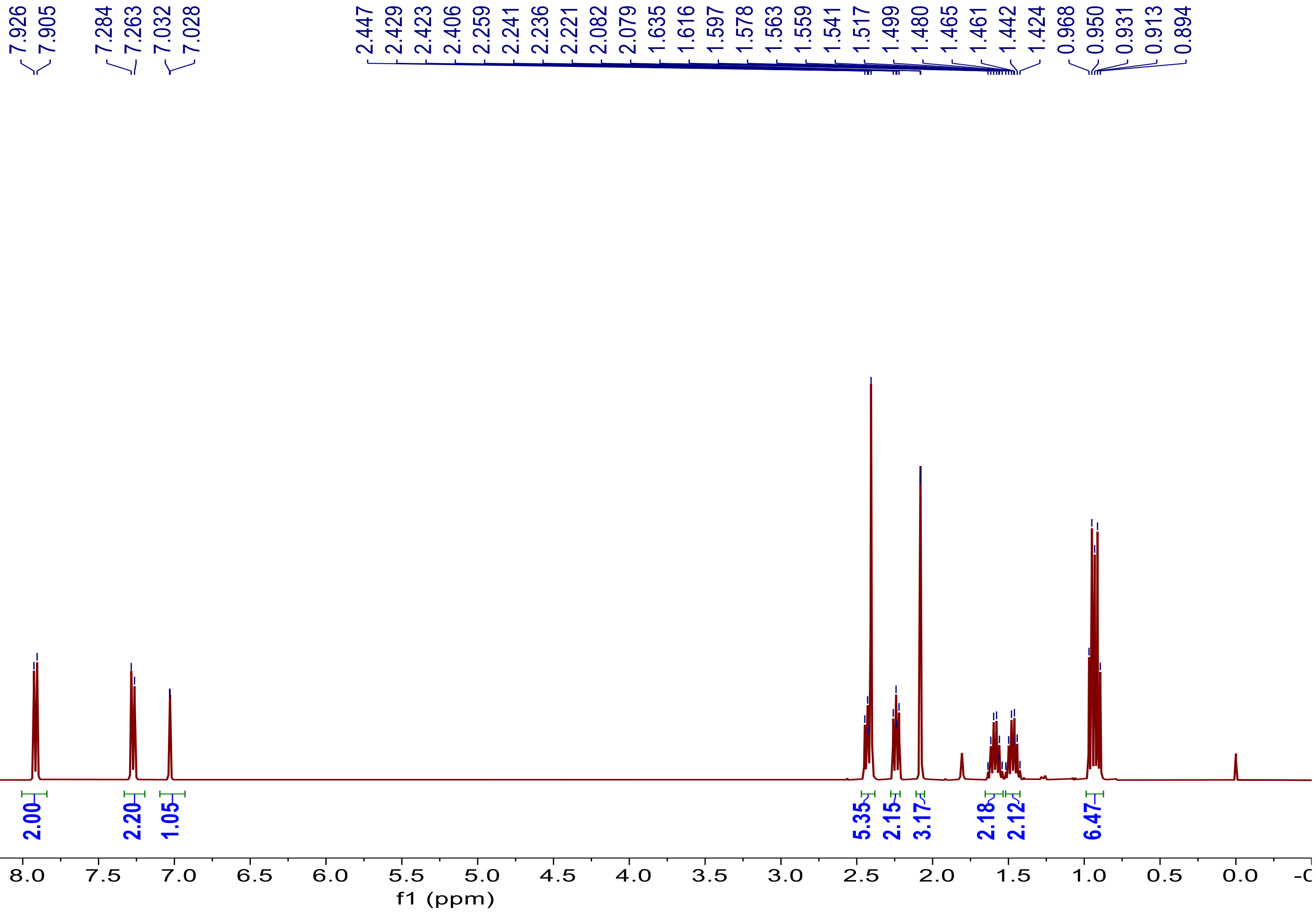

^{13}C NMR Spectrum of 7n at 25 °C ($CDCl_3$)

xyk-4-19-C-0716
Std carbon

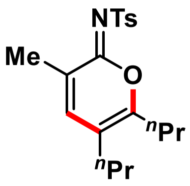

7n

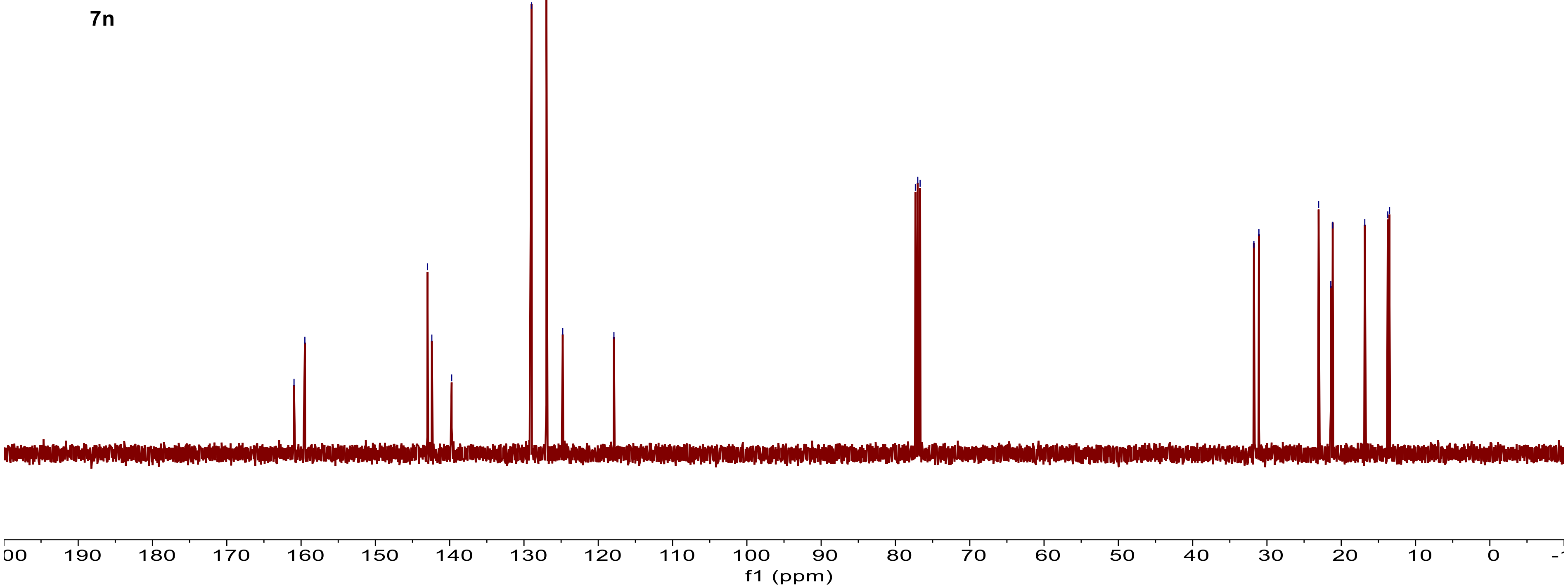

^{1}H NMR Spectrum of 7o at 25 ºC ($CDCl_3$)

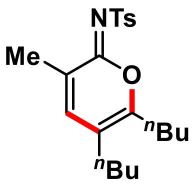

7o

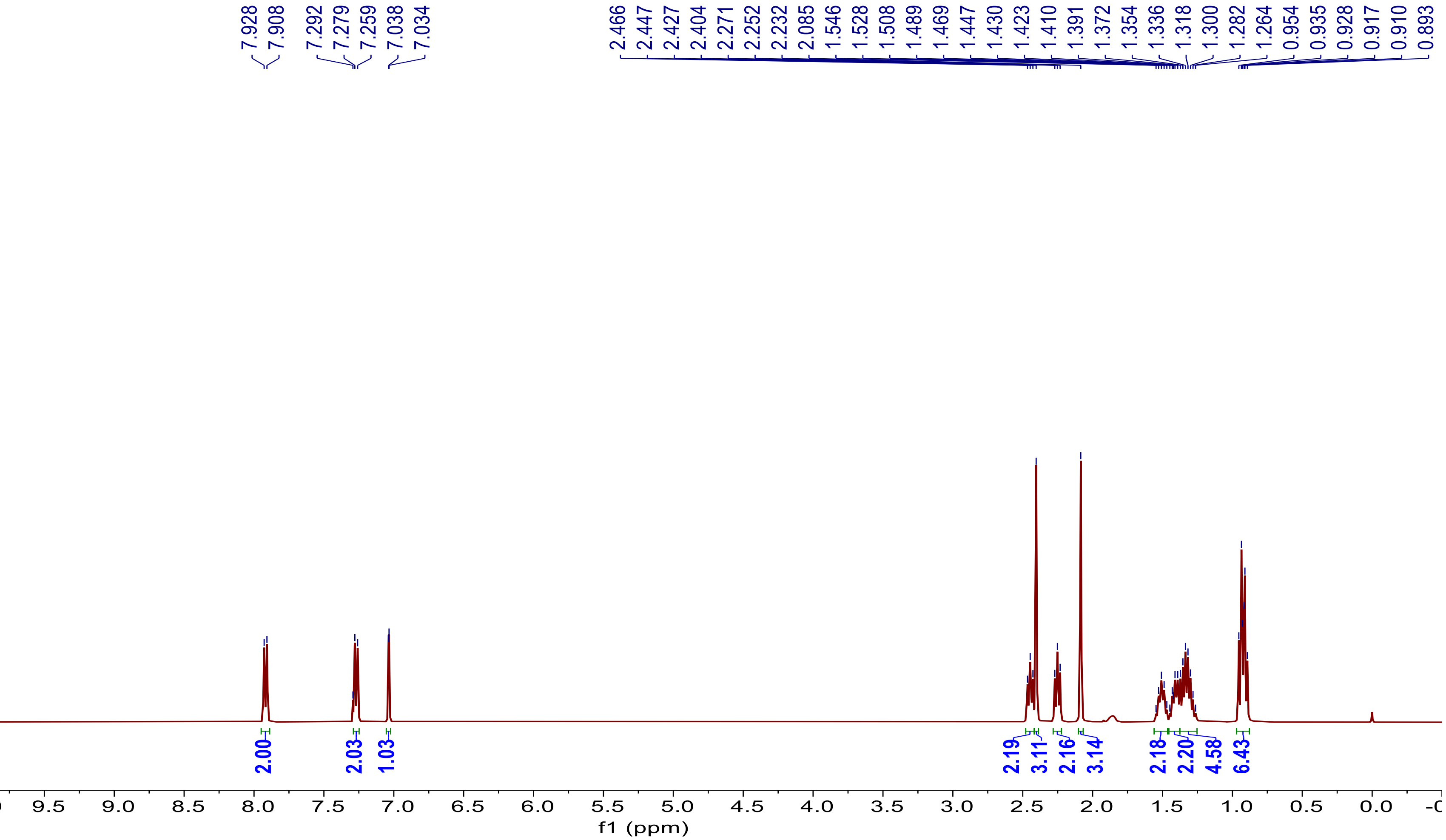

^{13}C NMR Spectrum of 7o at 25 ºC ($CDCl_3$)

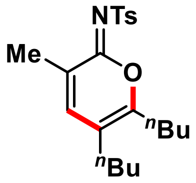

7o

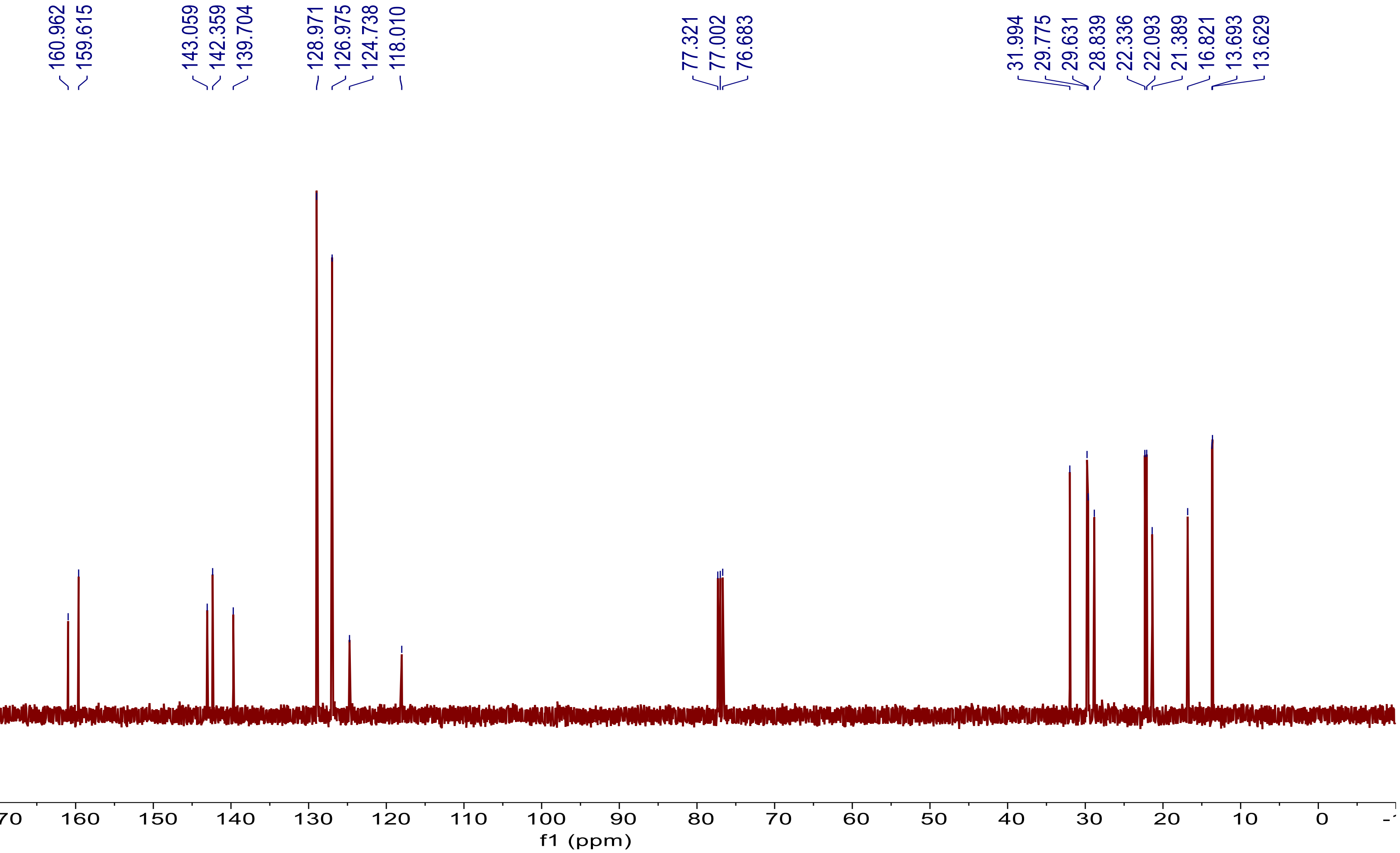

^{1}H NMR Spectrum of 7p at 25 ^{o}C ($CDCl_3$)

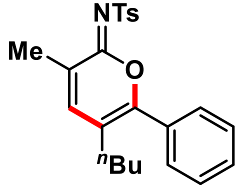

7p

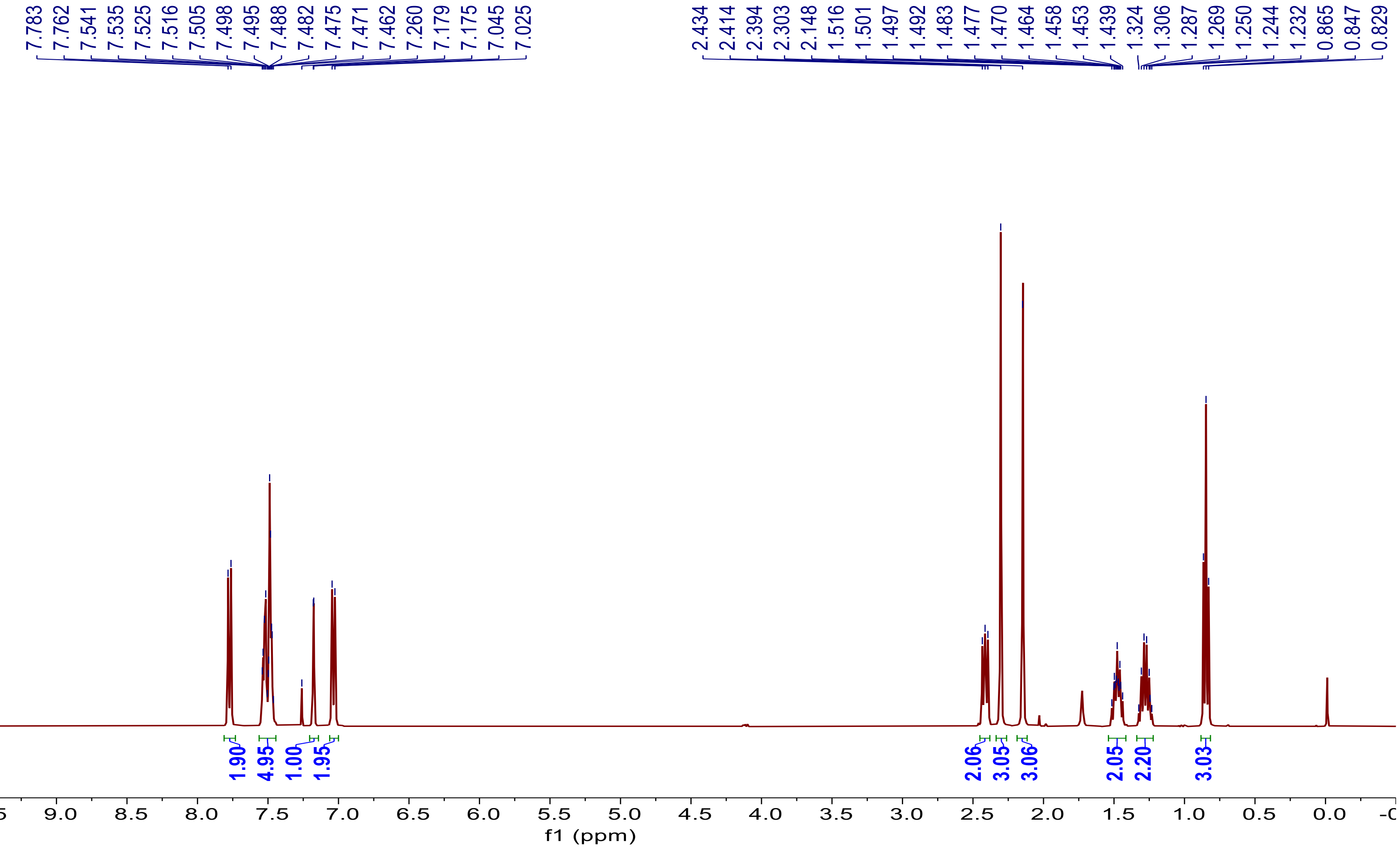

^{13}C NMR Spectrum of 7p at 25 ºC ($CDCl_3$)

xyk-4-71-C-0714
Std carbon

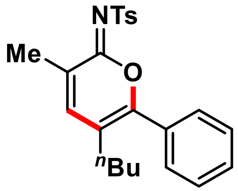

7p

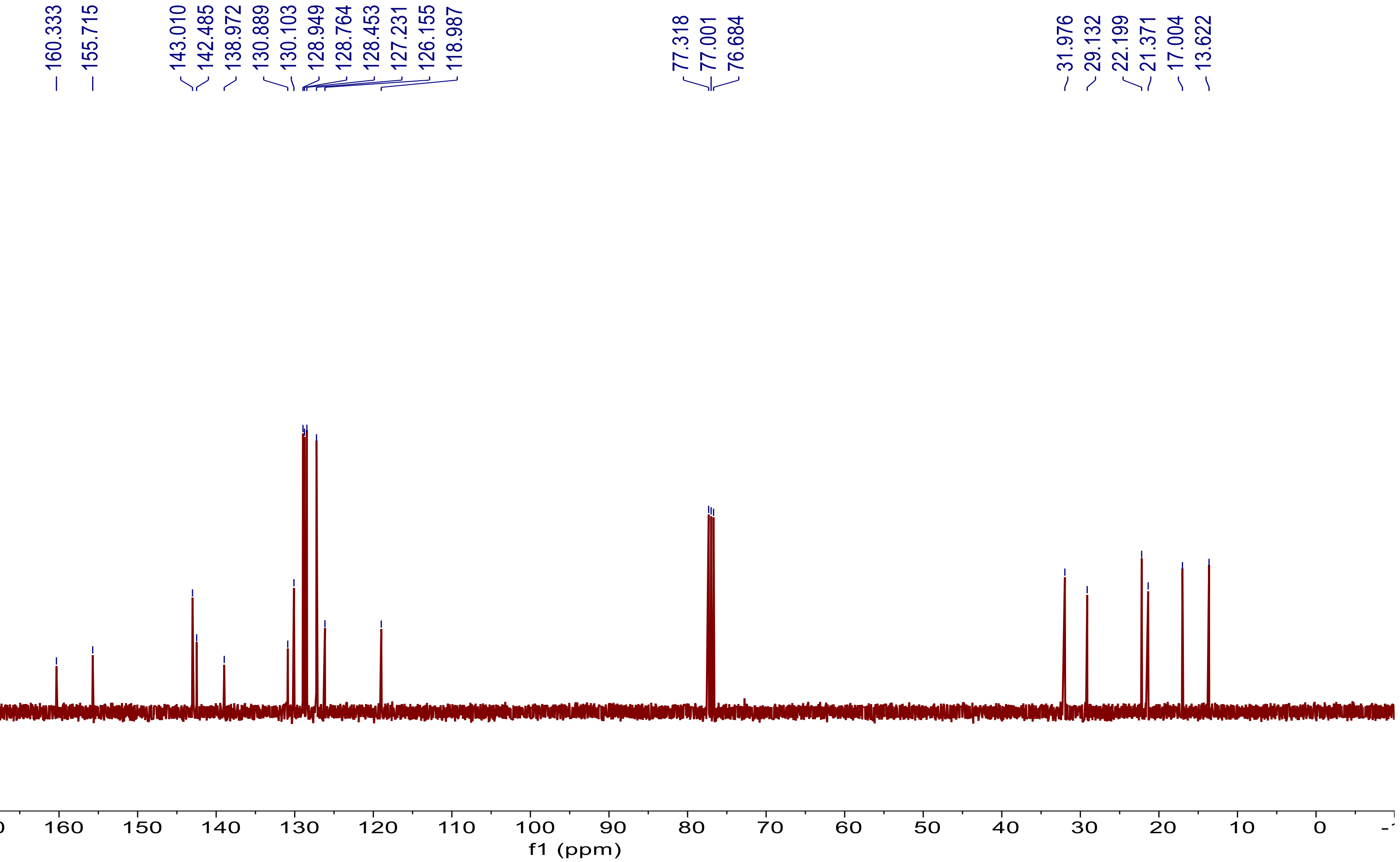

^{1}H NMR Spectrum of 7q at 25 ºC ($CDCl_3$)

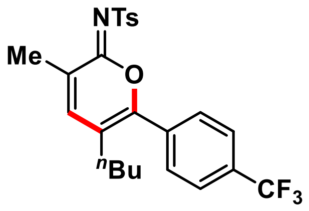

7q

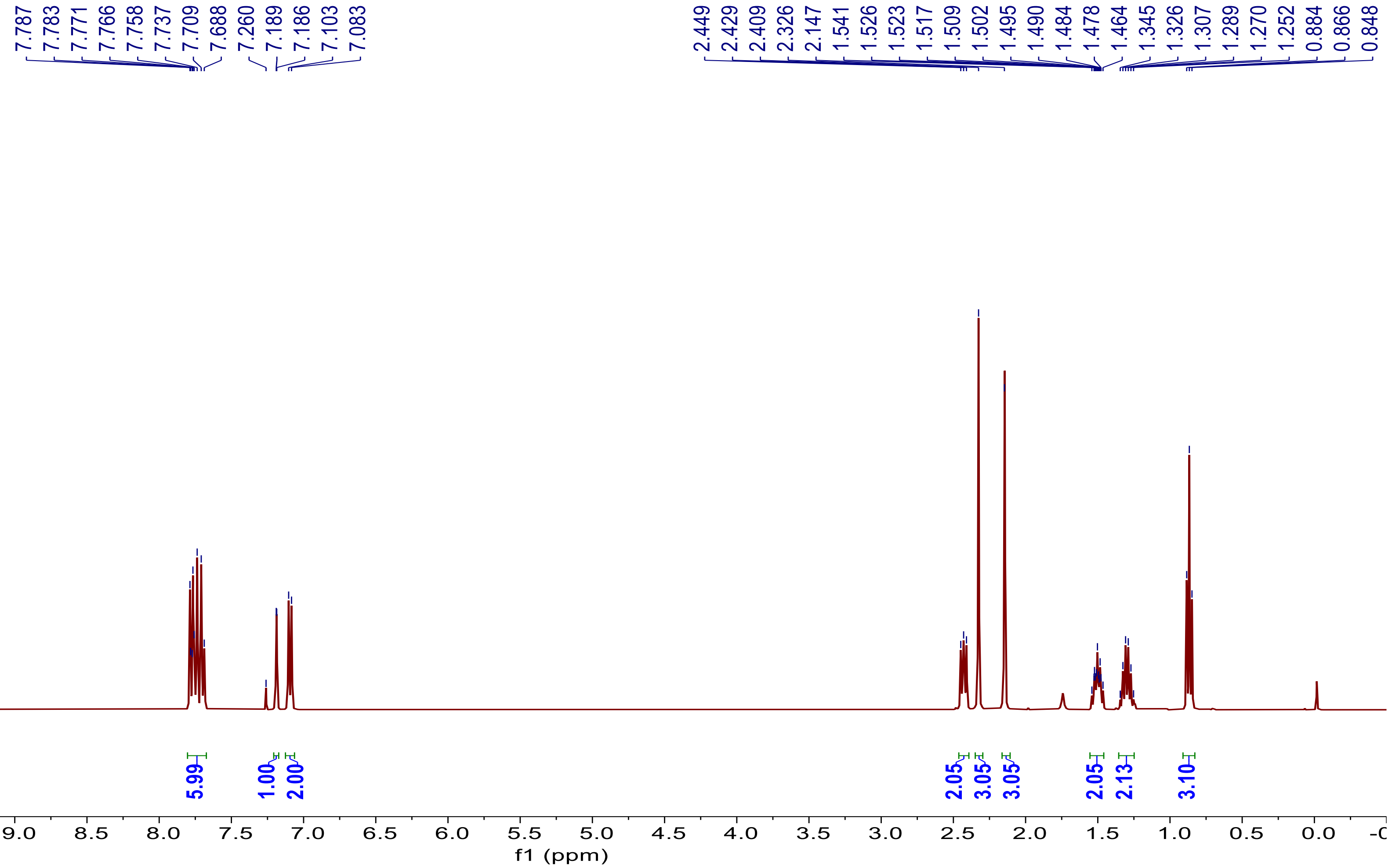

^{13}C NMR Spectrum of 7q at 25 °C ($CDCl_3$)

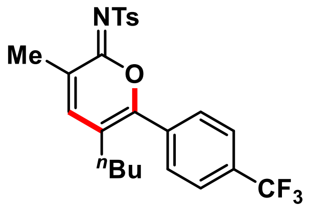

7q

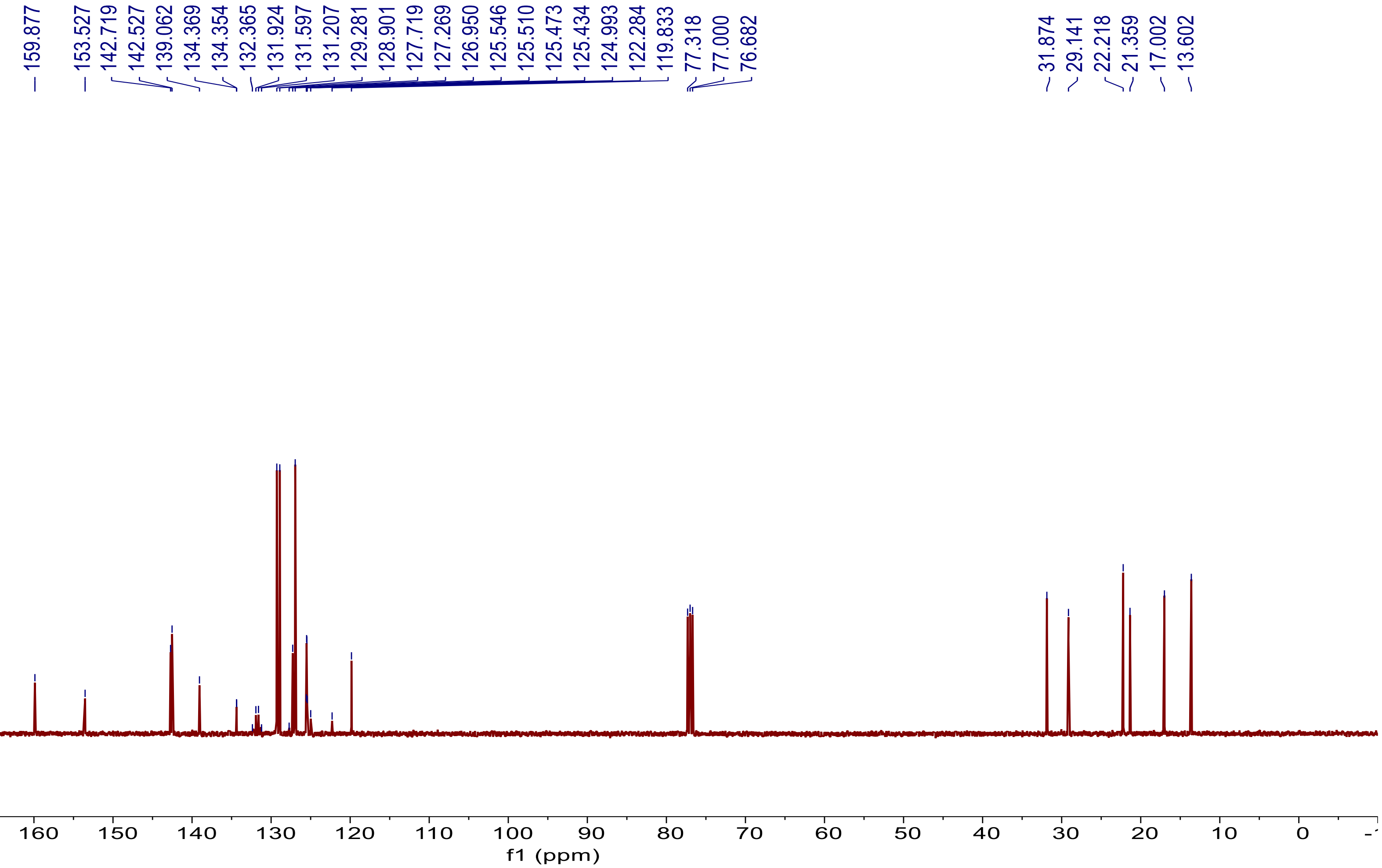

^{19}F NMR Spectrum of 7q at 25 ºC ($CDCl_3$)

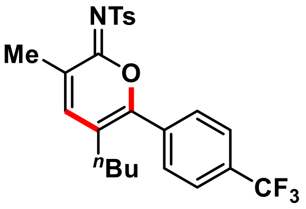

7q

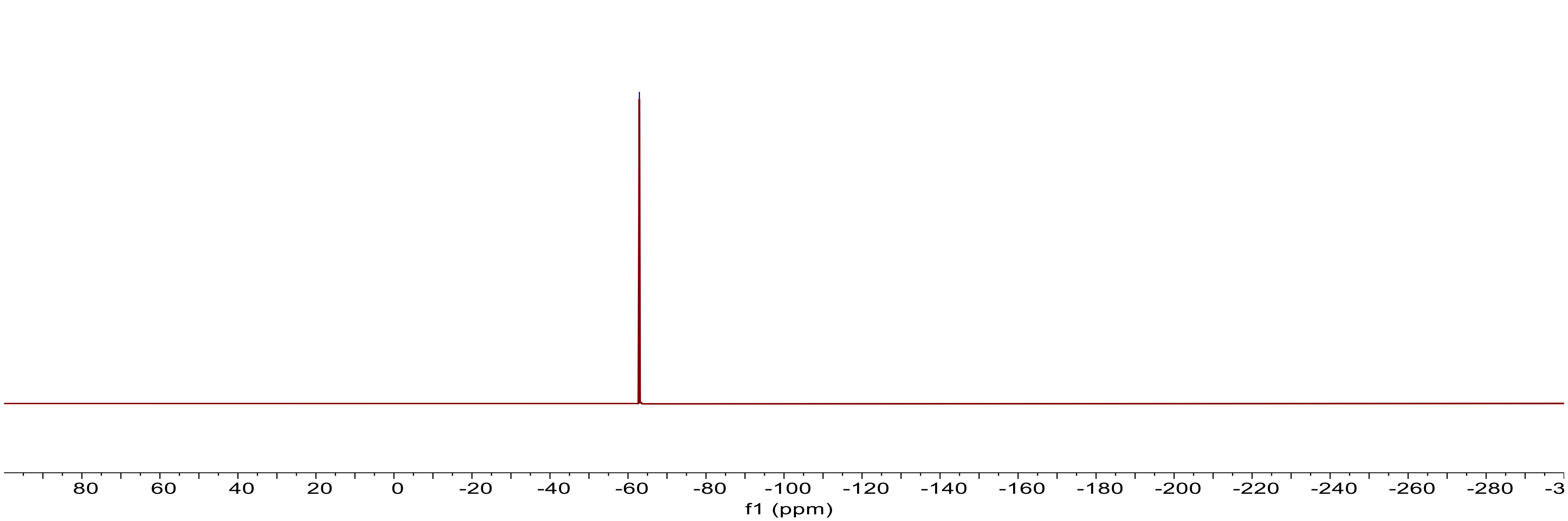

^{1}H NMR Spectrum of 7r at 25 ºC ($CDCl_3$)

xyk-4-43-H-0514

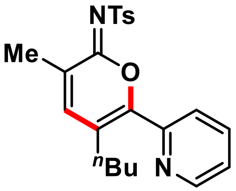

7r

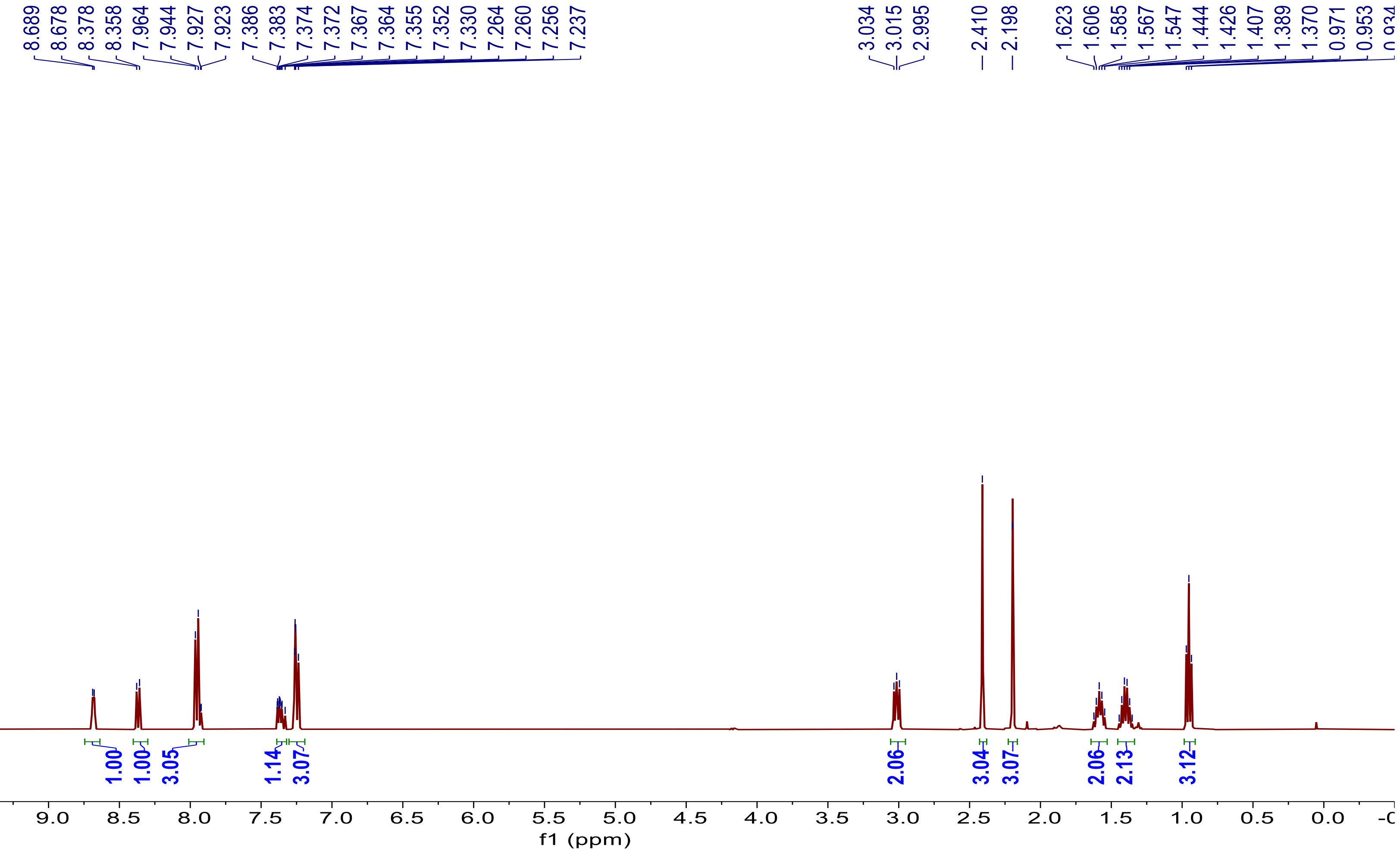

^{13}C NMR Spectrum of 7r at 25 °C ($CDCl_3$)

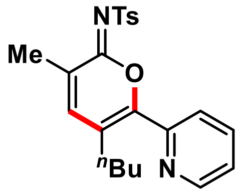

7r

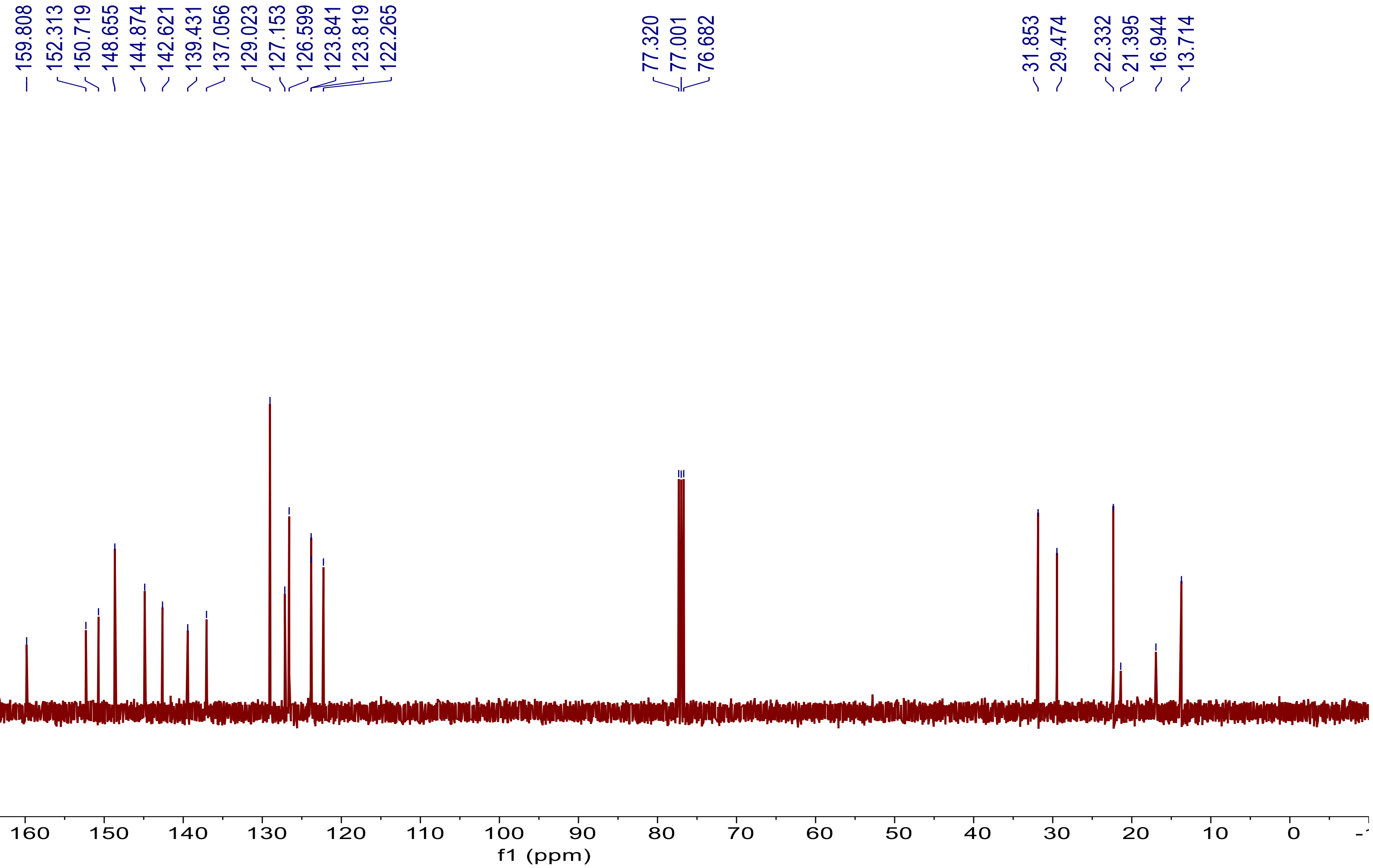

^{1}H NMR Spectrum of 7s at 25 ºC ($CDCl_3$)

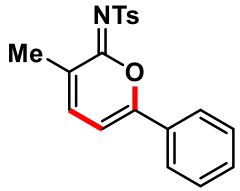

7s

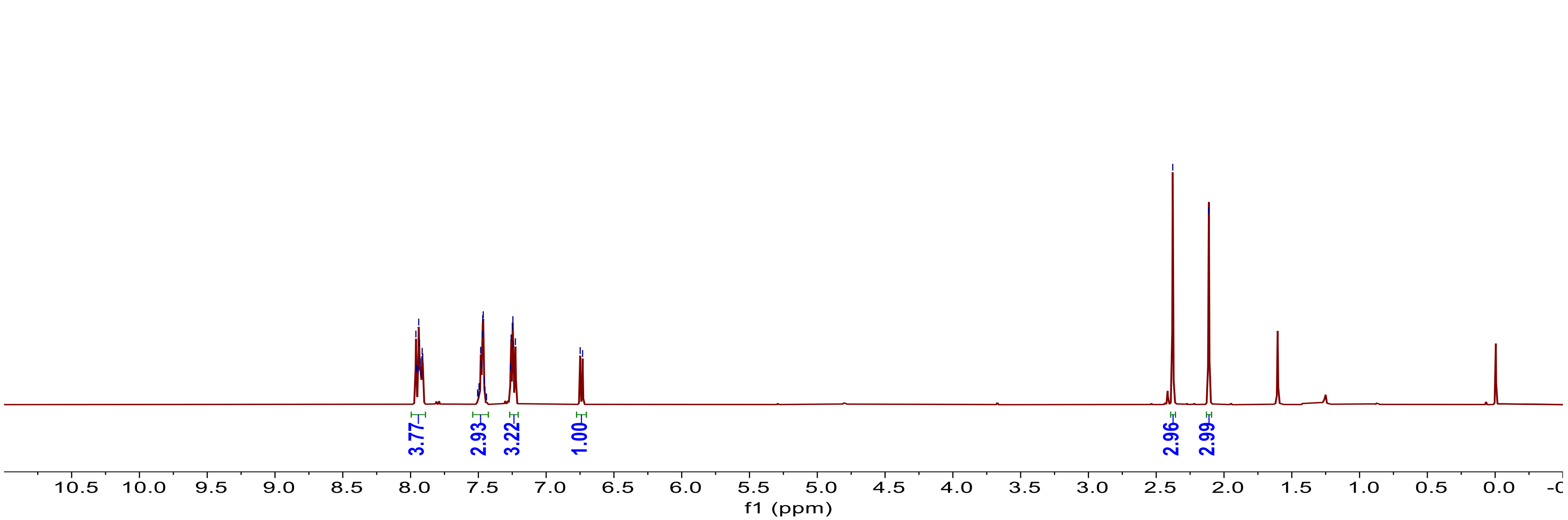

^{13}C NMR Spectrum of 7s at 25 °C ($CDCl_3$)

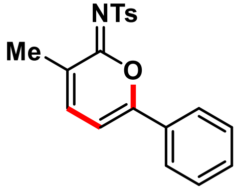

7s

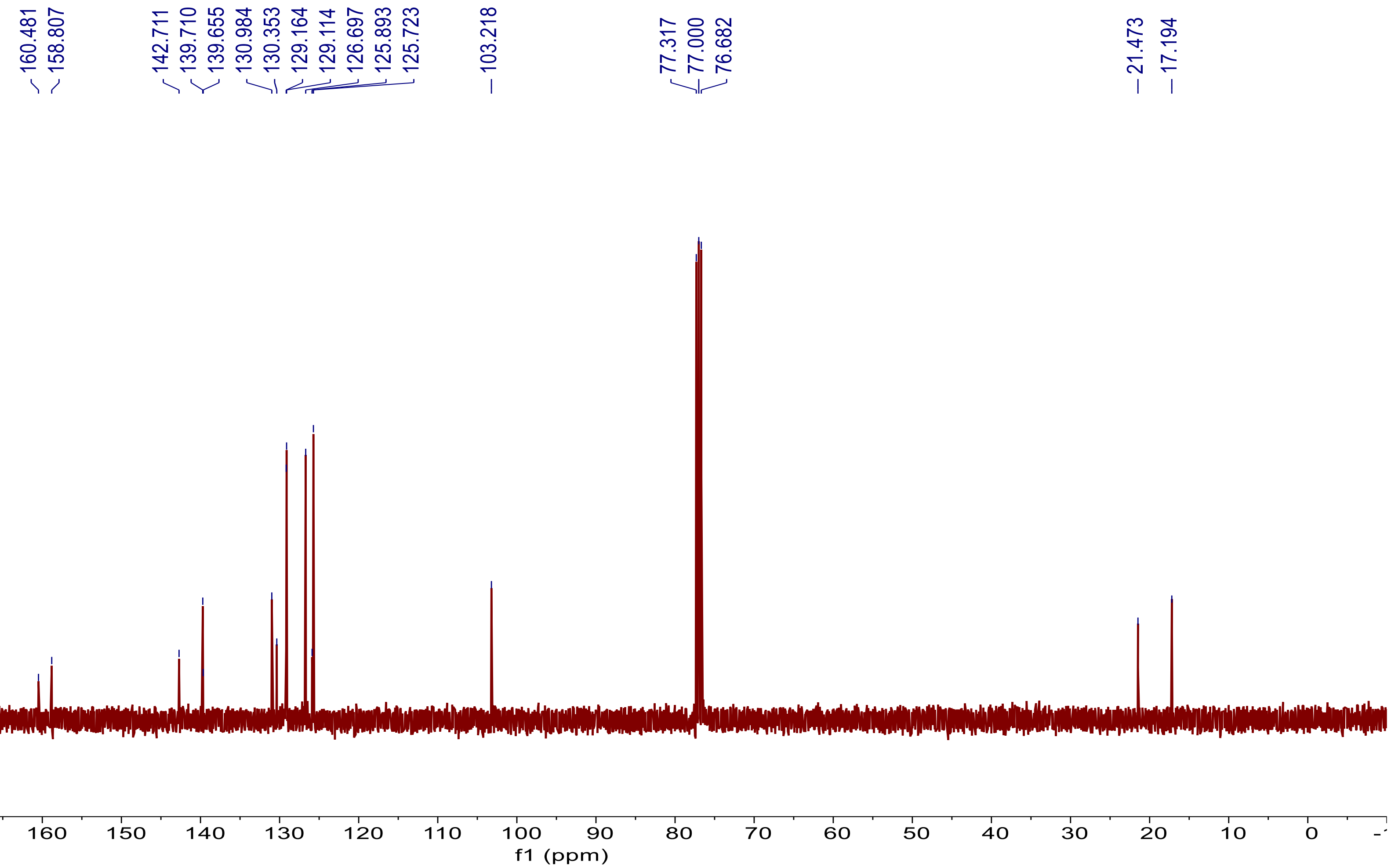

^{1}H NMR Spectrum of 7t at 25 ºC ($CDCl_3$)

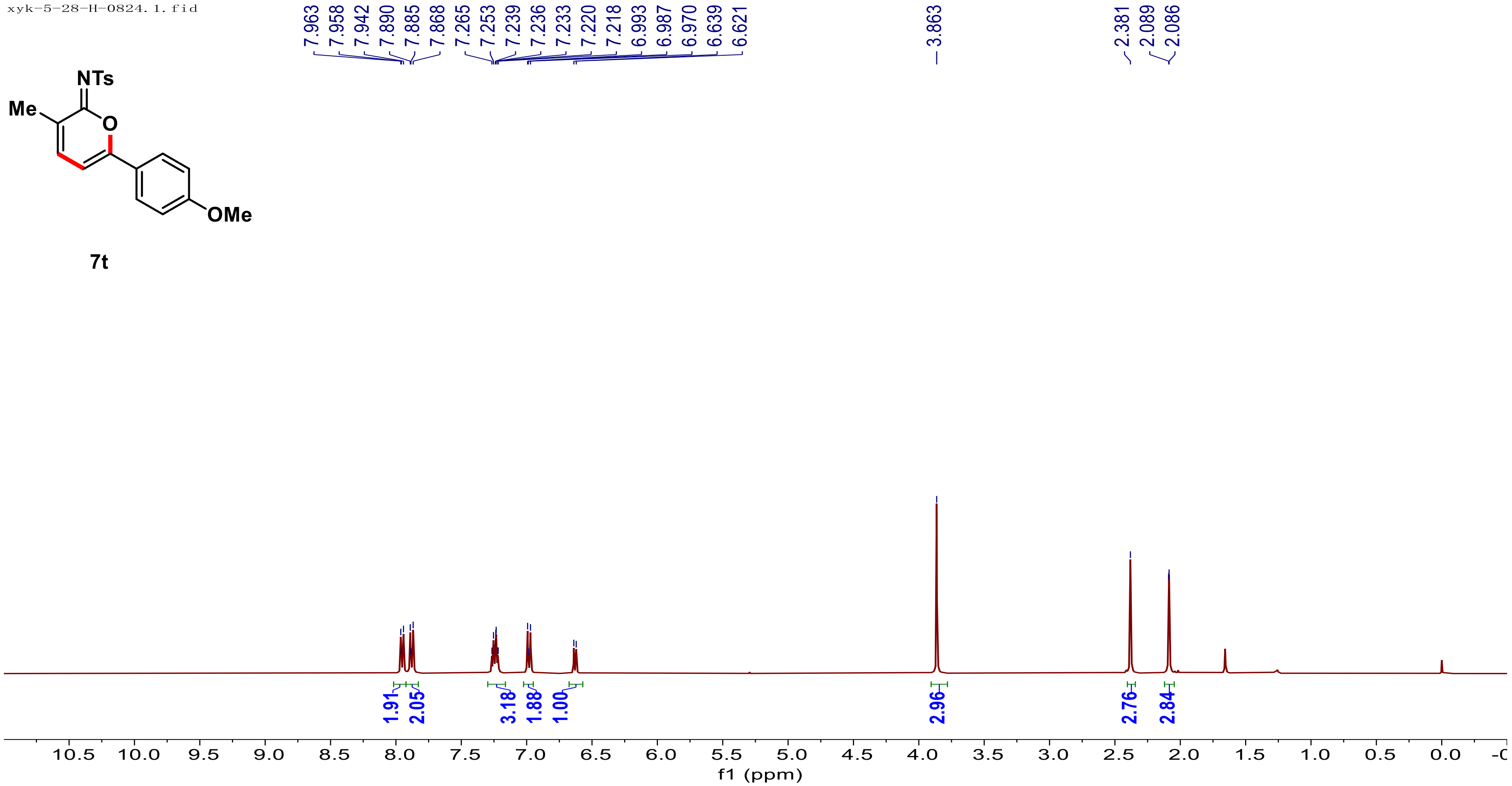

^{13}C NMR Spectrum of 7t at 25 ºC ($CDCl_3$)

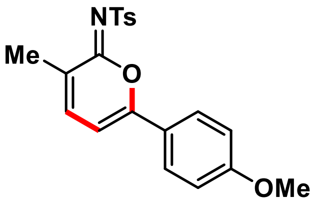

7t

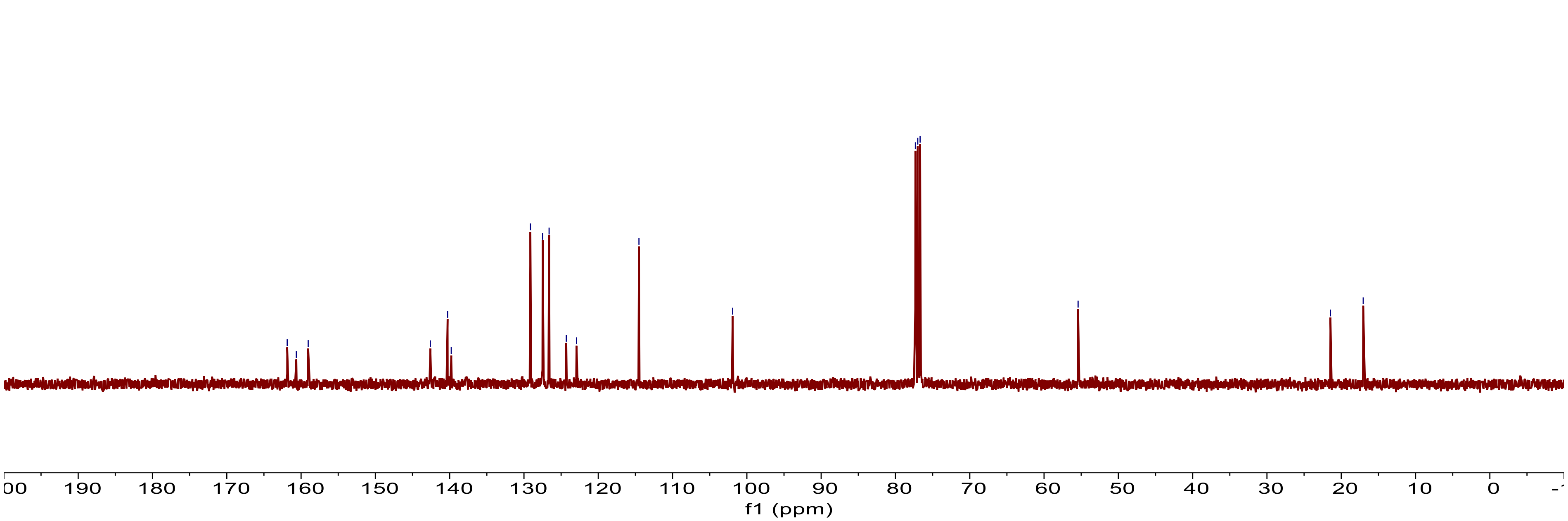

^{1}H NMR Spectrum of 7u at 25 ºC ($CDCl_3$)

xyk-5-40-H-0829
Std proton

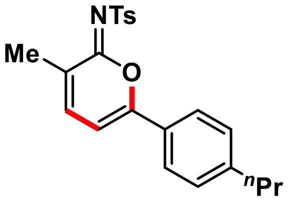

7u

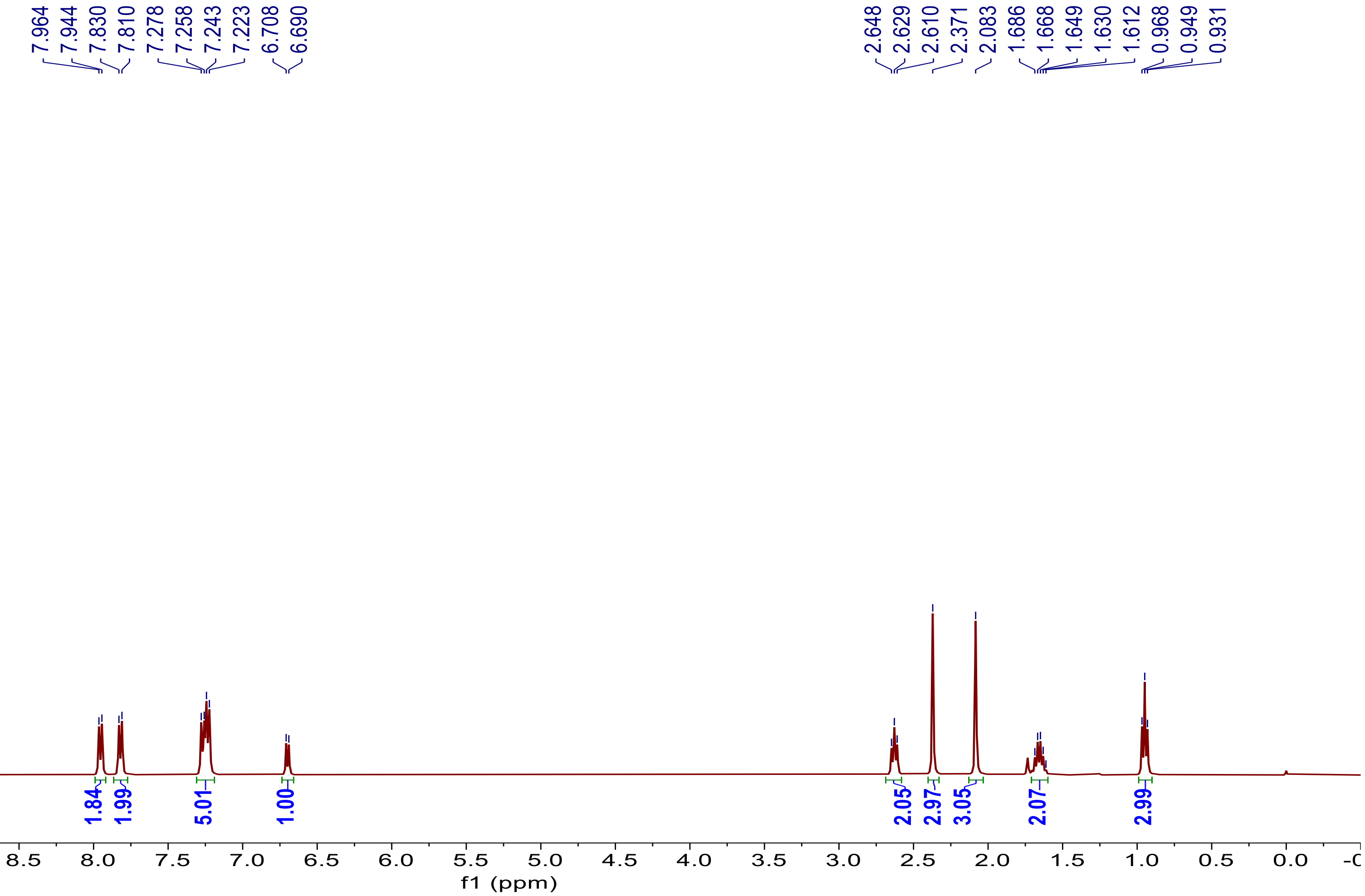

^{13}C NMR Spectrum of 7u at 25 ºC ($CDCl_3$)

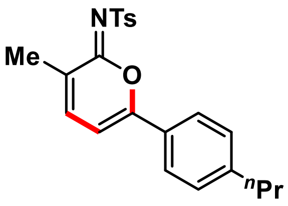

7u

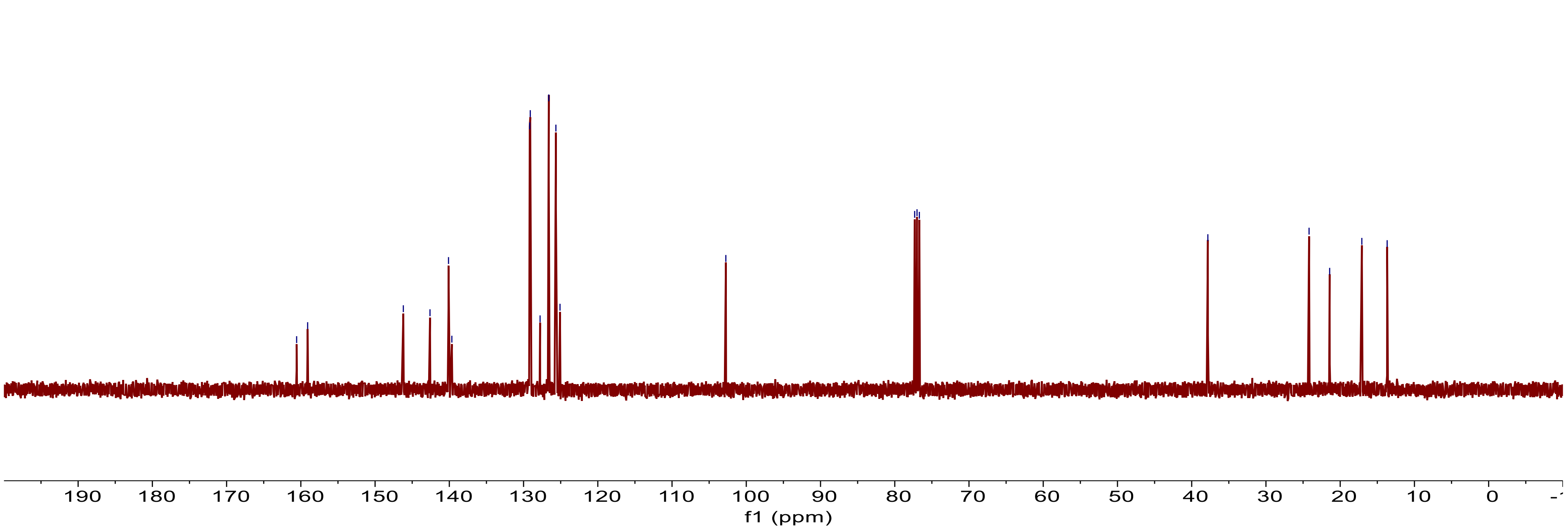

^{1}H NMR Spectrum of 7v at 25 ºC ($CDCl_3$)

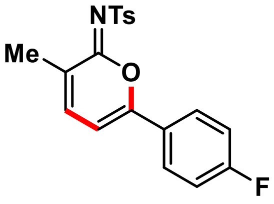

7v

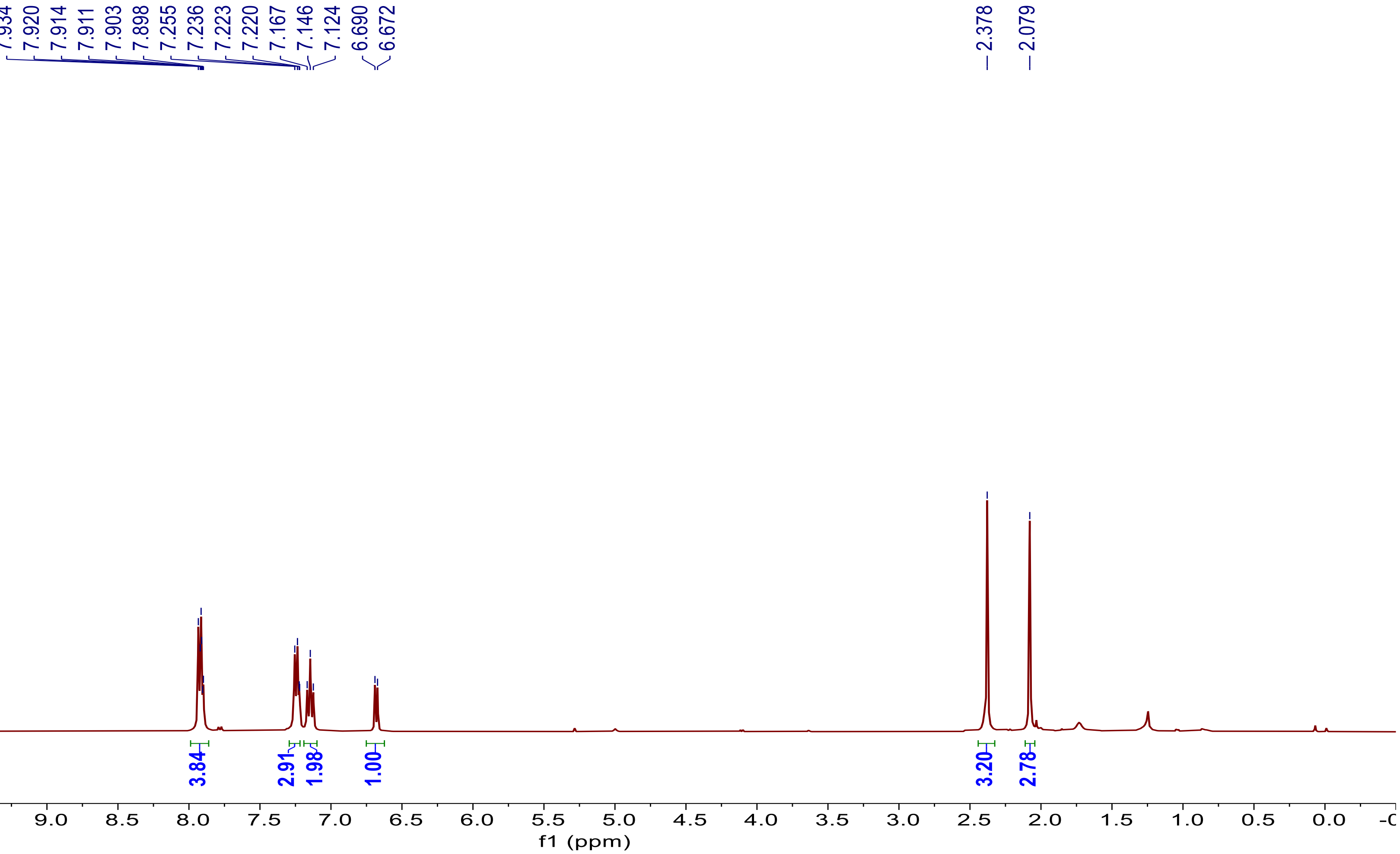

^{13}C NMR Spectrum of 7v at 25 ^{o}C ($CDCl_3$)

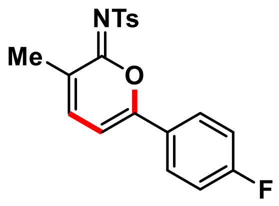

7v

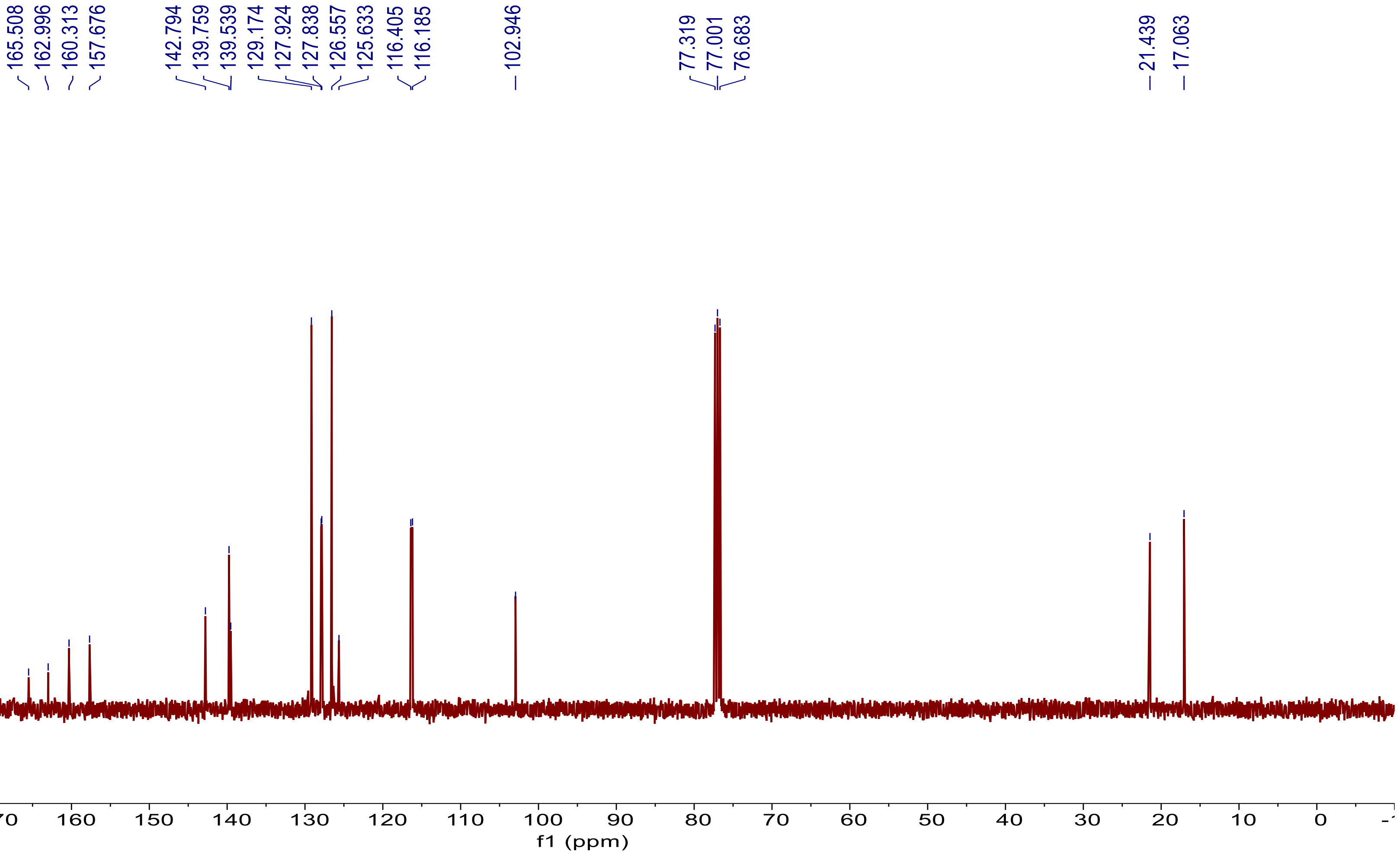

^{19}F NMR Spectrum of 7v at 25 ºC ($CDCl_3$)

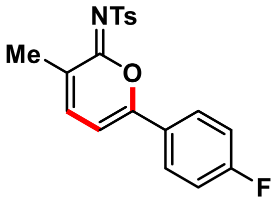

7v

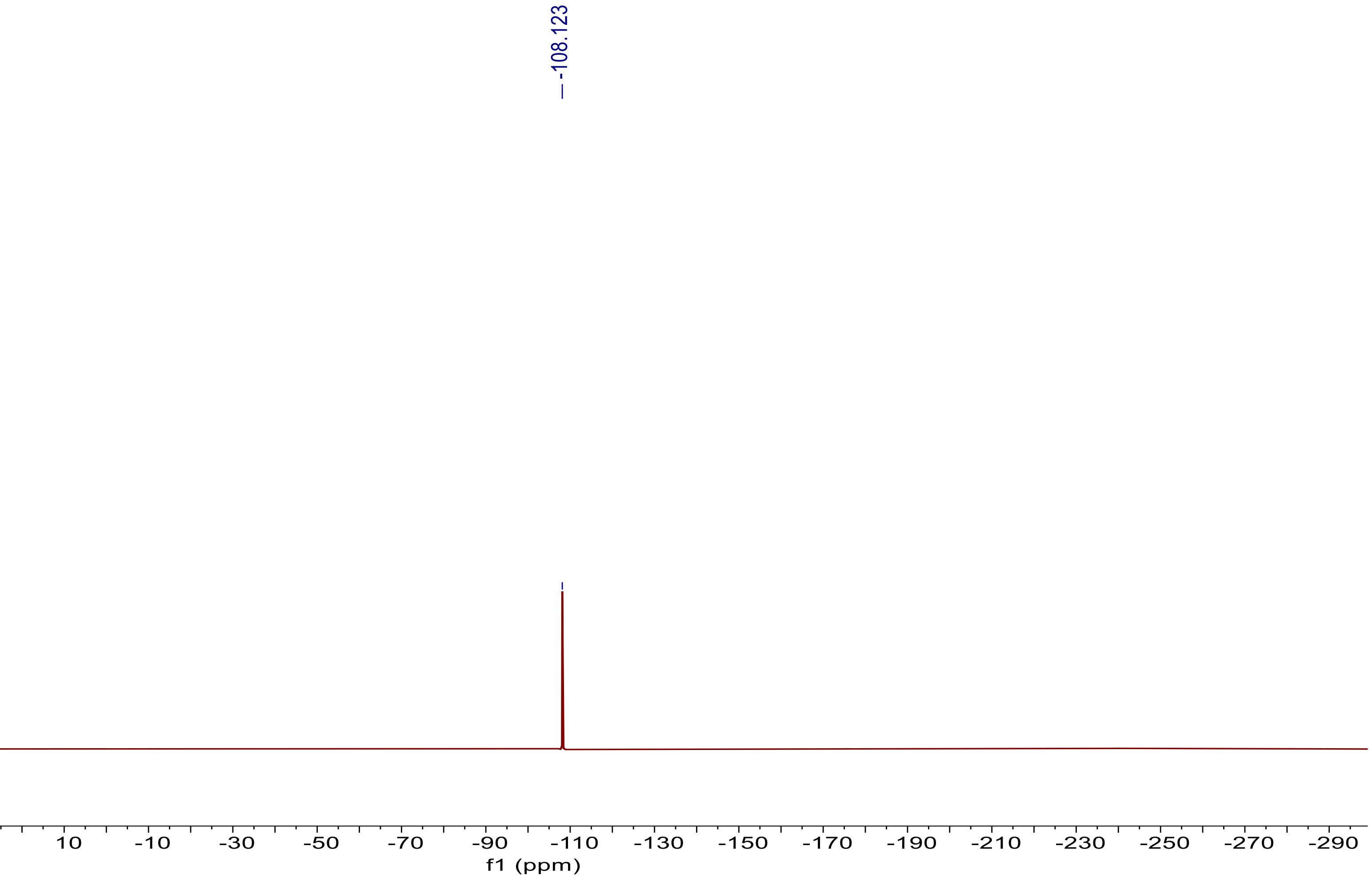

^{1}H NMR Spectrum of 7w at 25 ºC ($CDCl_3$)

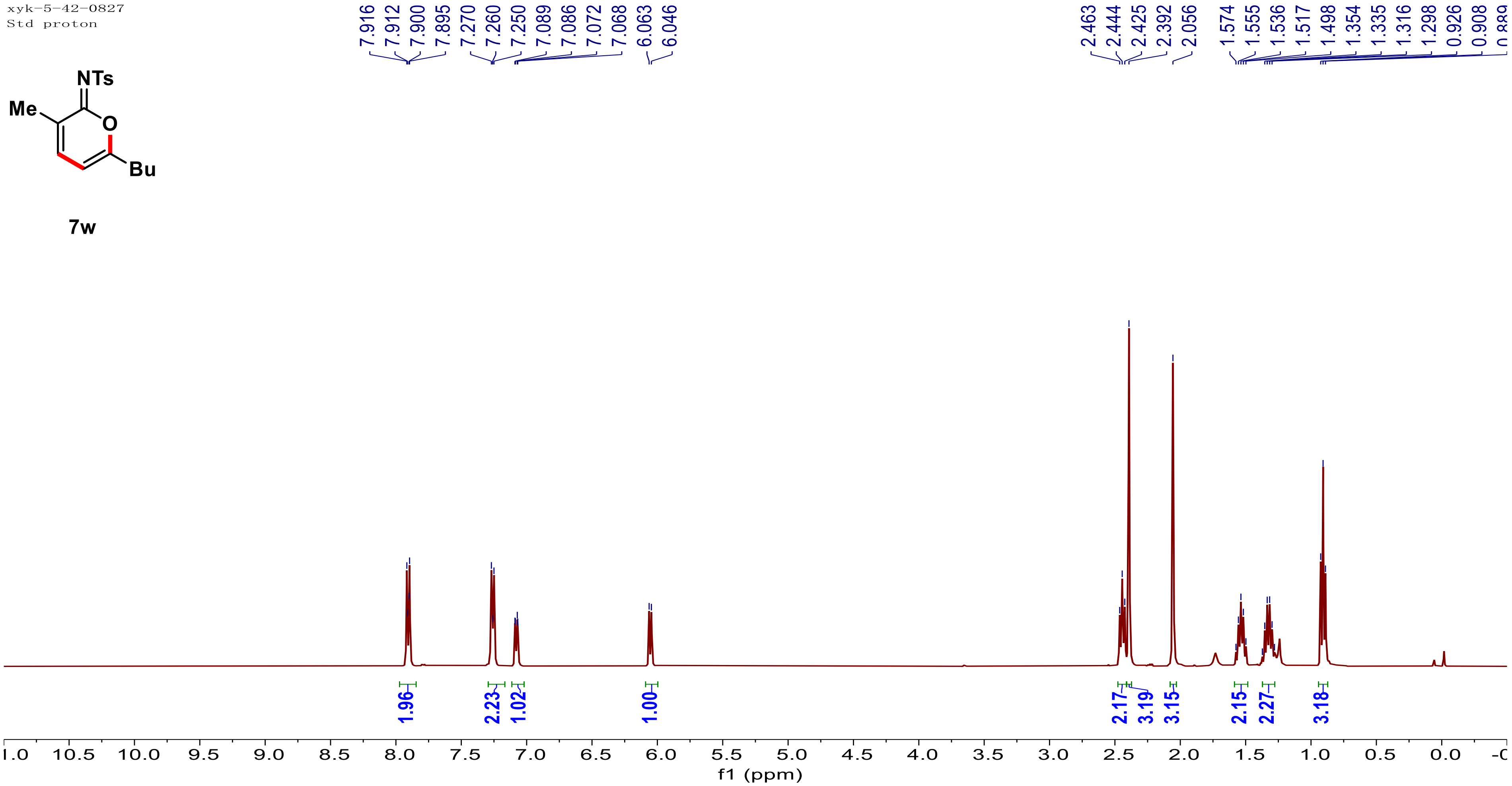

^{13}C NMR Spectrum of 7w at 25 ºC ($CDCl_3$)

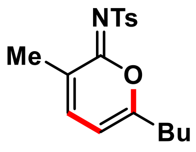

7w

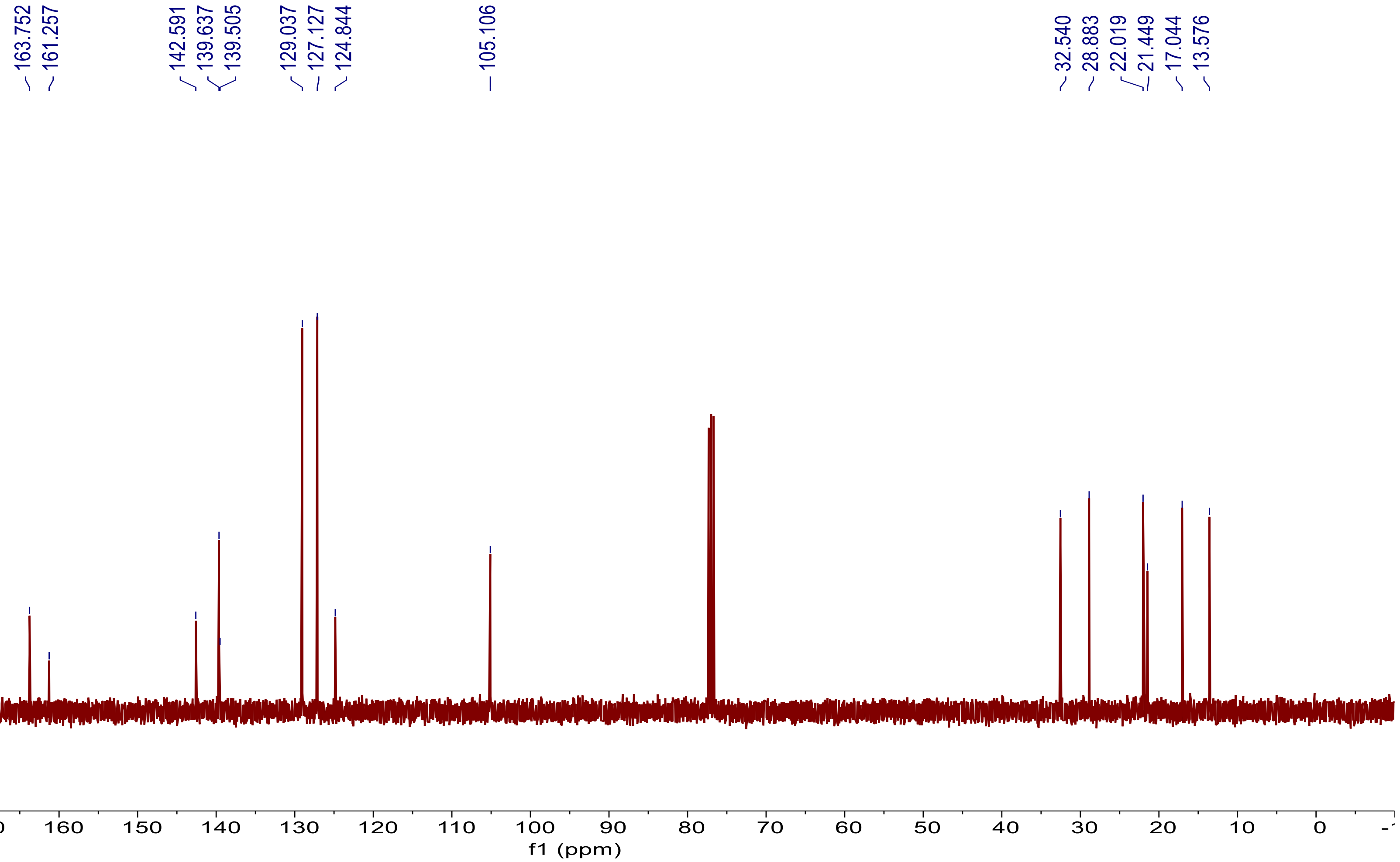

^{1}H NMR Spectrum of 7x at 25 ºC ($CDCl_3$)

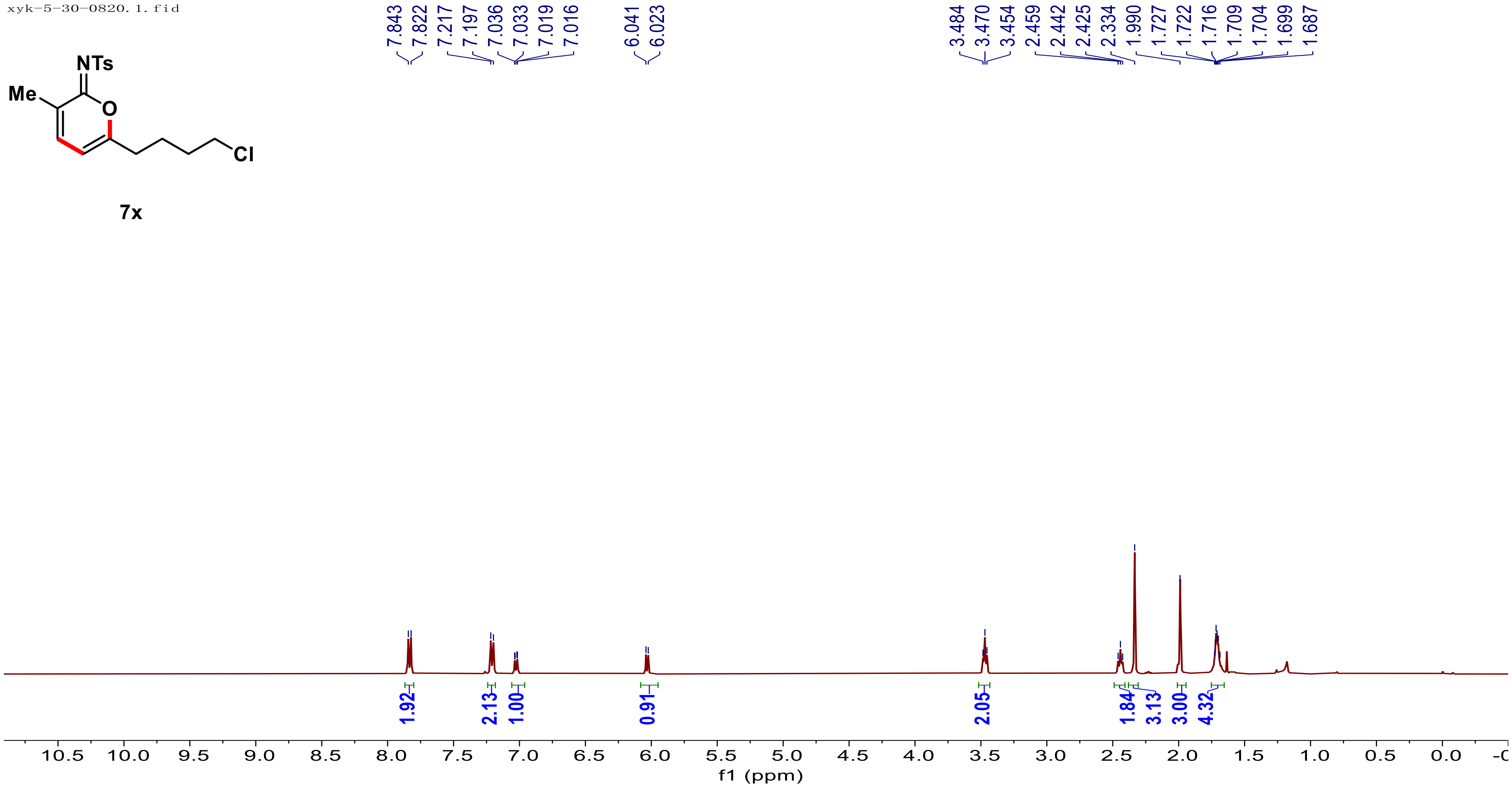

^{13}C NMR Spectrum of 7x at 25 °C ($CDCl_3$)

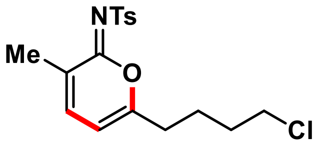

7x

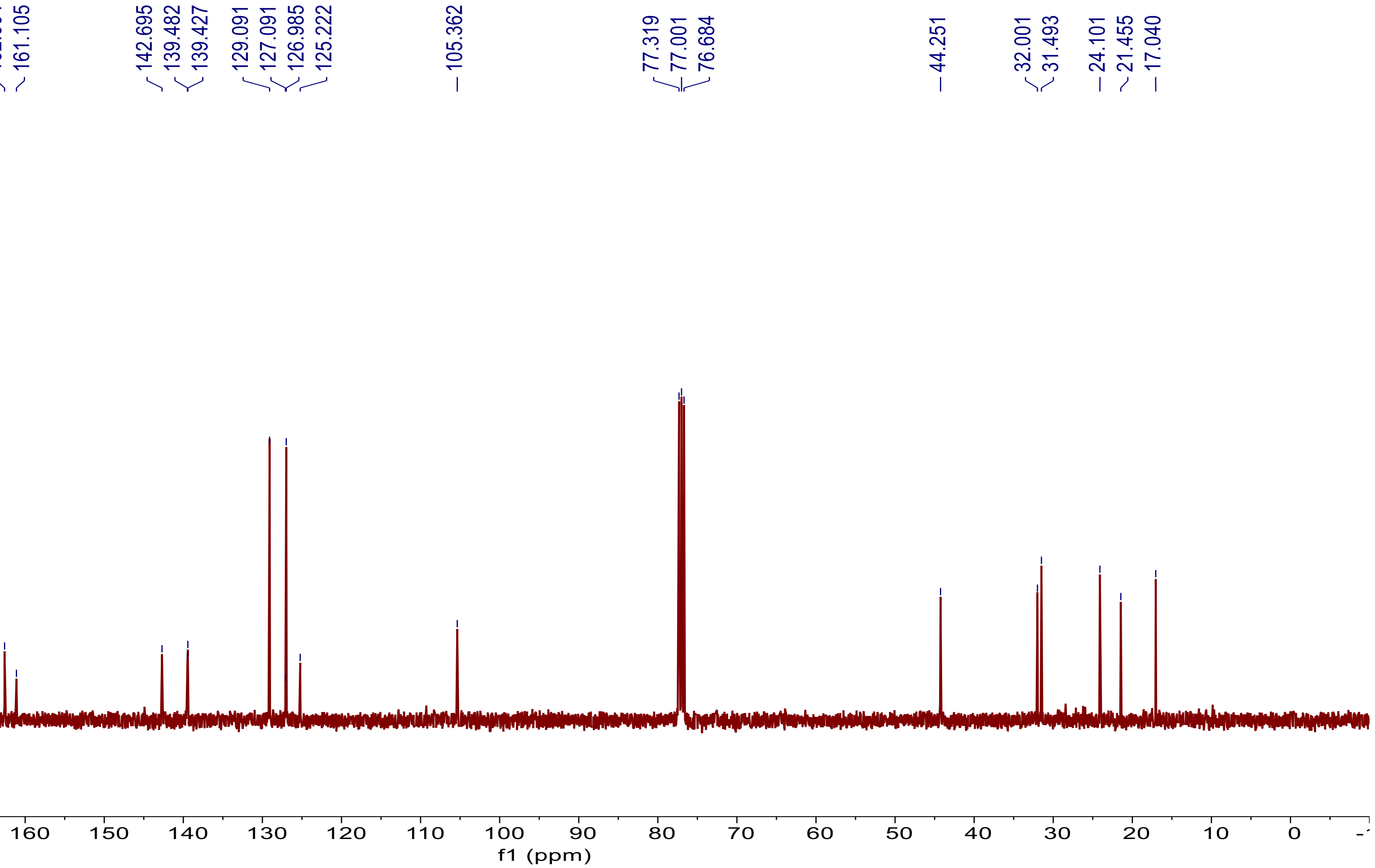

^{1}H NMR Spectrum of 8a at 25 °C ($CDCl_3$)

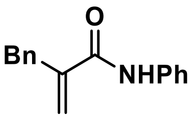

8a

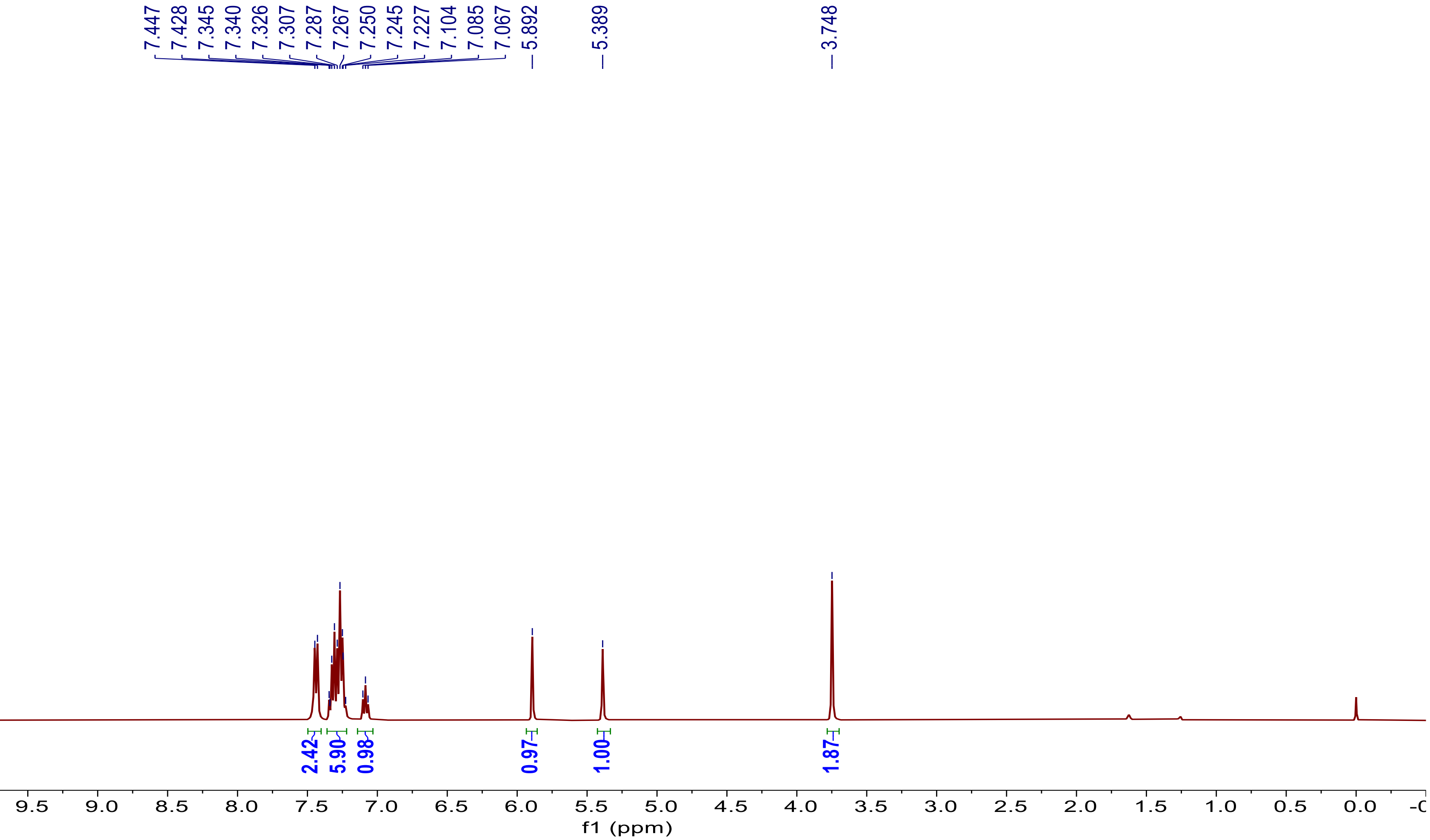

^{1}H NMR Spectrum of 8b at 25 °C ($CDCl_3$)

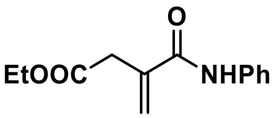

8b

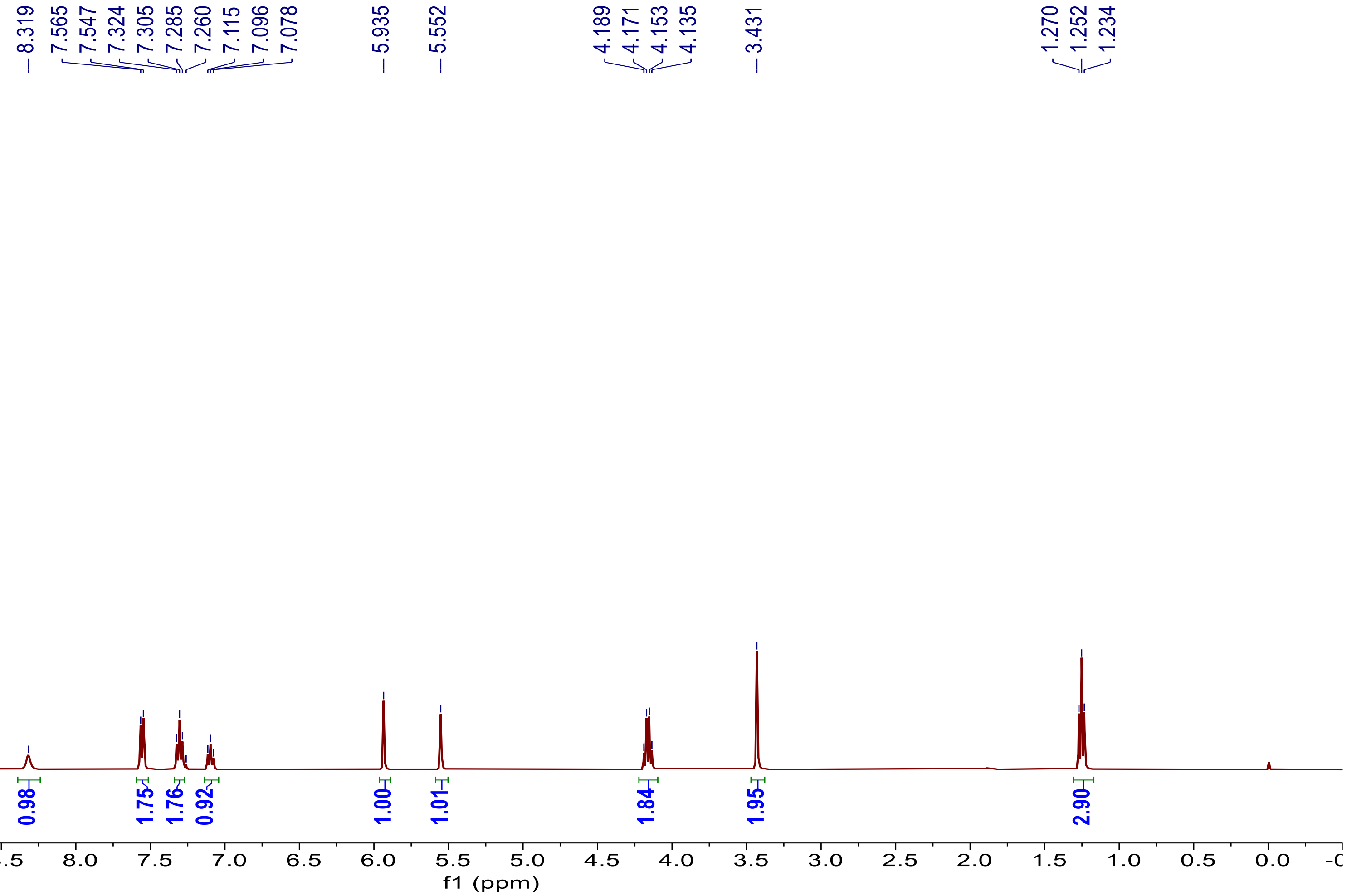

^{13}C NMR Spectrum of 8b at 25 ºC ($CDCl_3$)

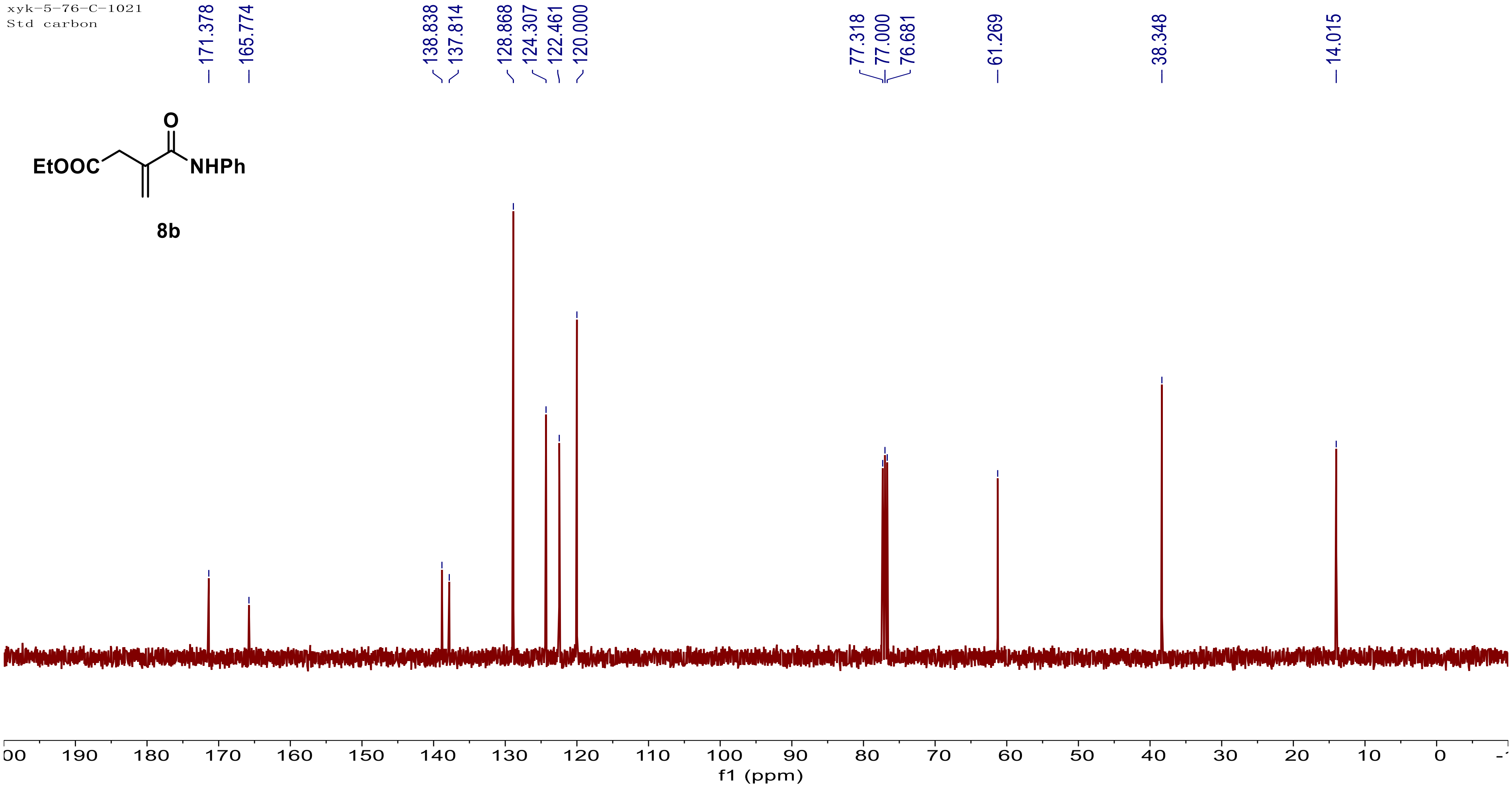

^{1}H NMR Spectrum of 8c at 25 ºC ($CDCl_3$)

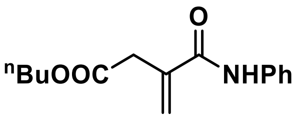

8c

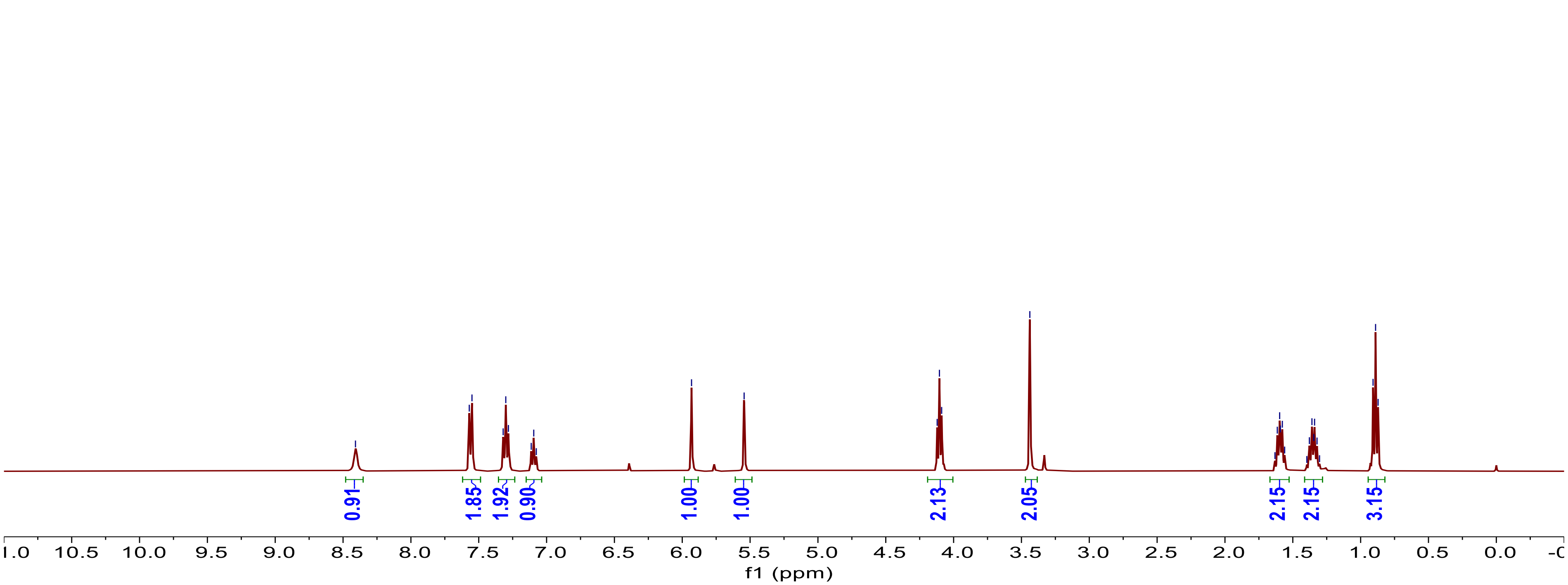

^{13}C NMR Spectrum of 8c at 25 ºC ($CDCl_3$)

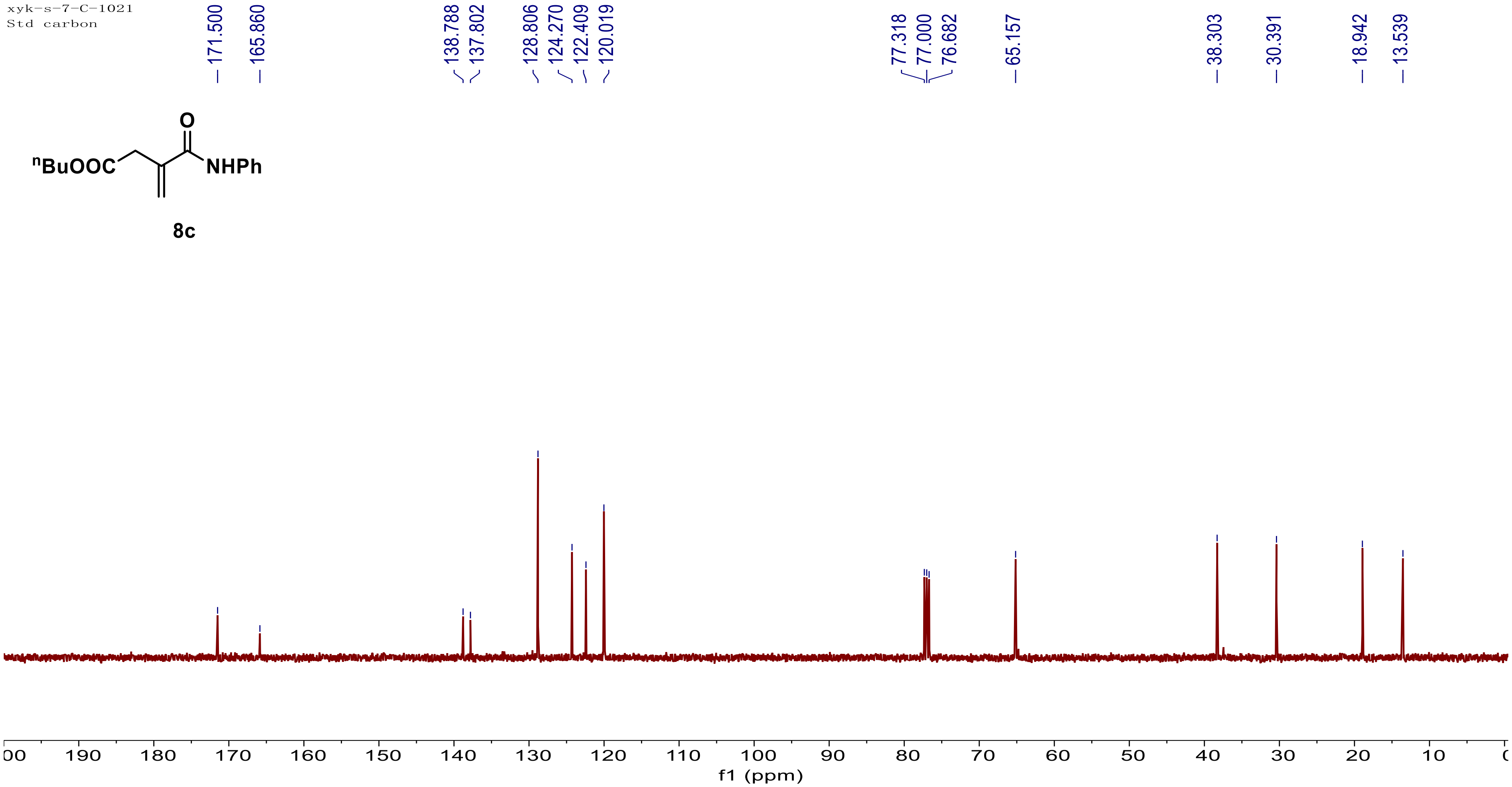

^{1}H NMR Spectrum of 8d at 25 °C ($CDCl_3$)

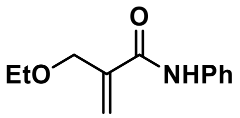

8d

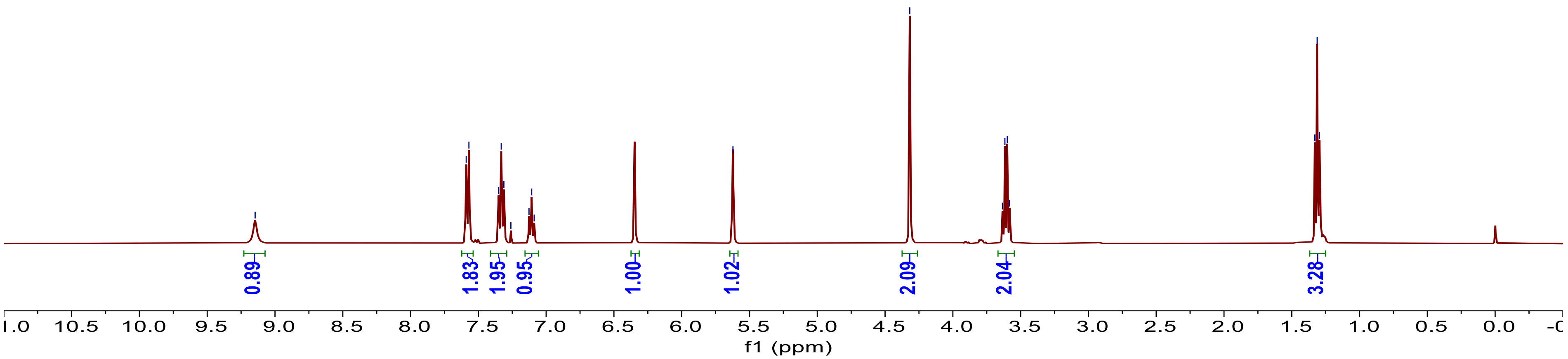

^{13}C NMR Spectrum of 8d at 25 ºC ($CDCl_3$)

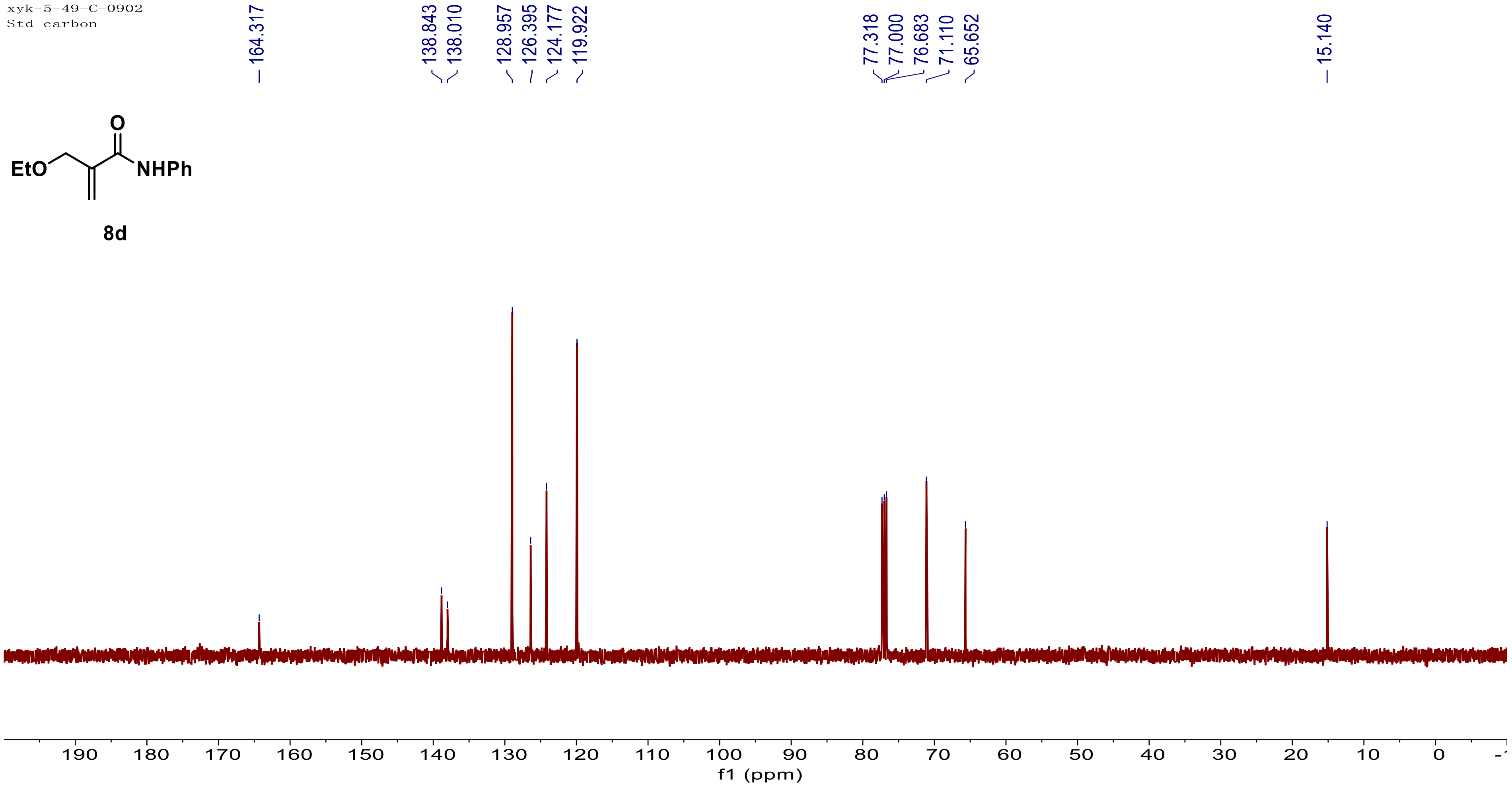

^{1}H NMR Spectrum of 8e at 25 ºC ($CDCl_3$)

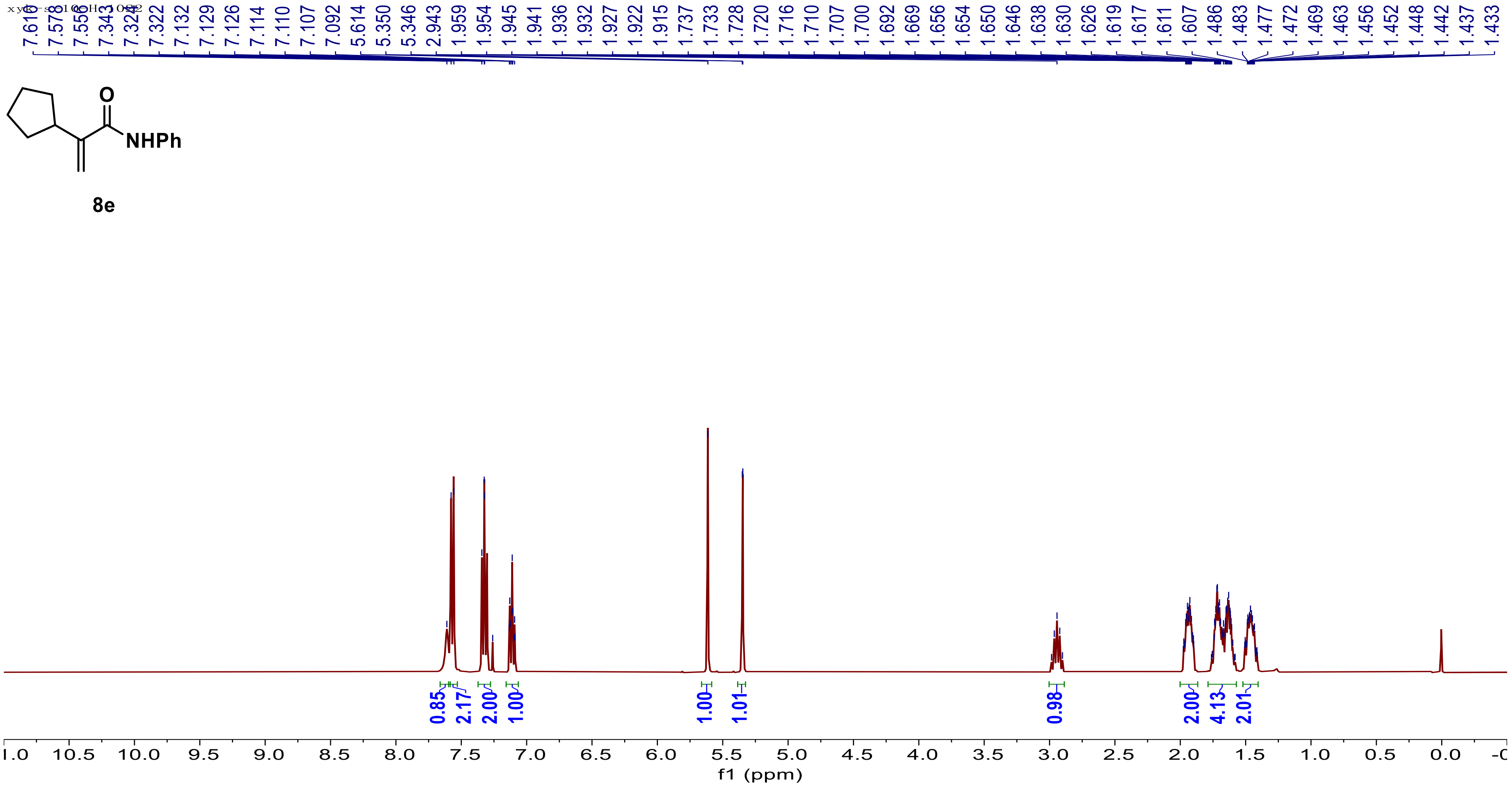

^{13}C NMR Spectrum of 8e at 25 ºC ($CDCl_3$)

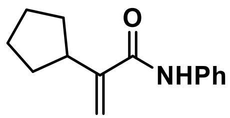

8e

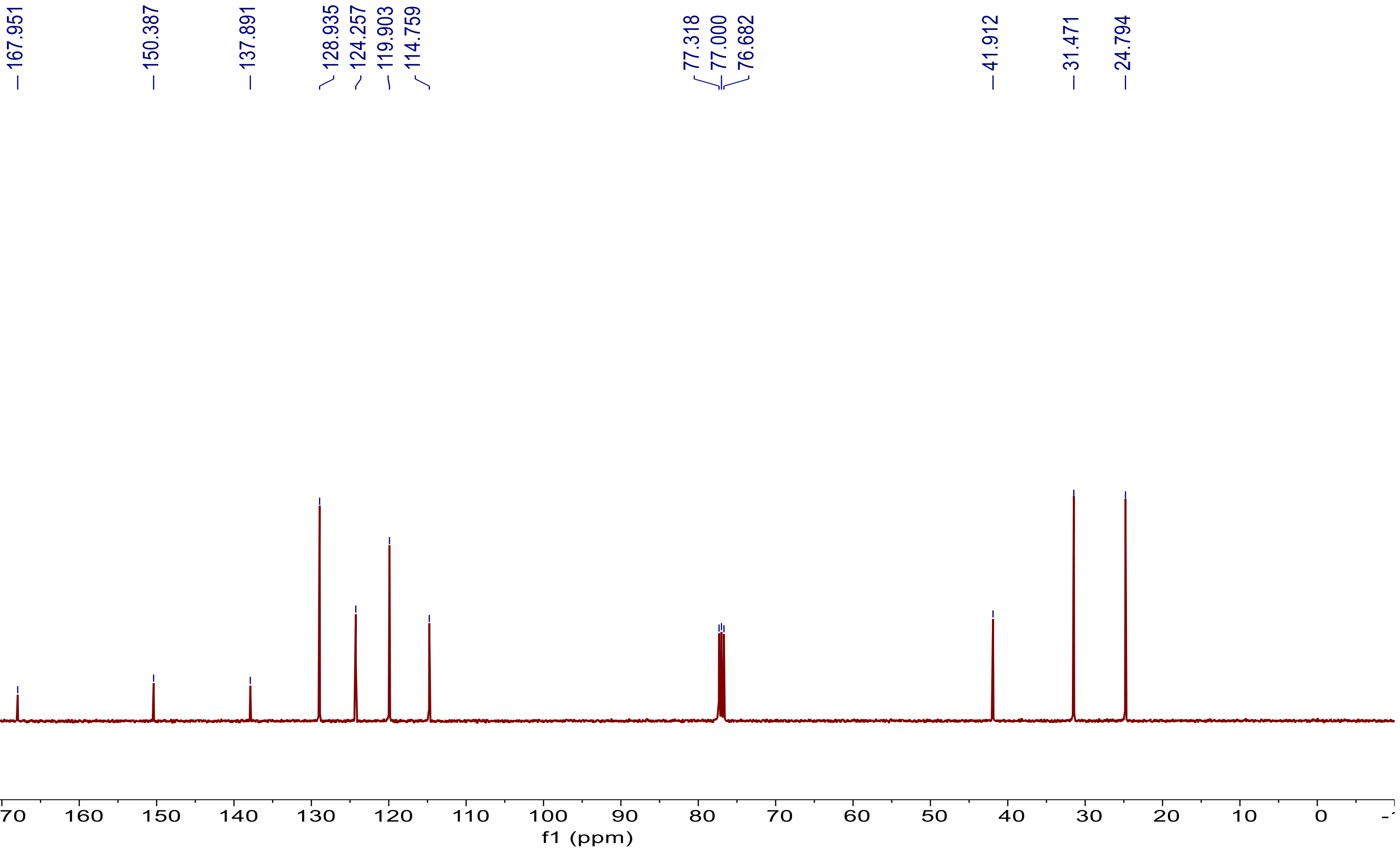

^{1}H NMR Spectrum of 8f at 25 ºC ($CDCl_3$)

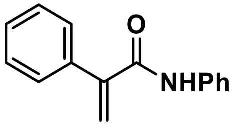

8f

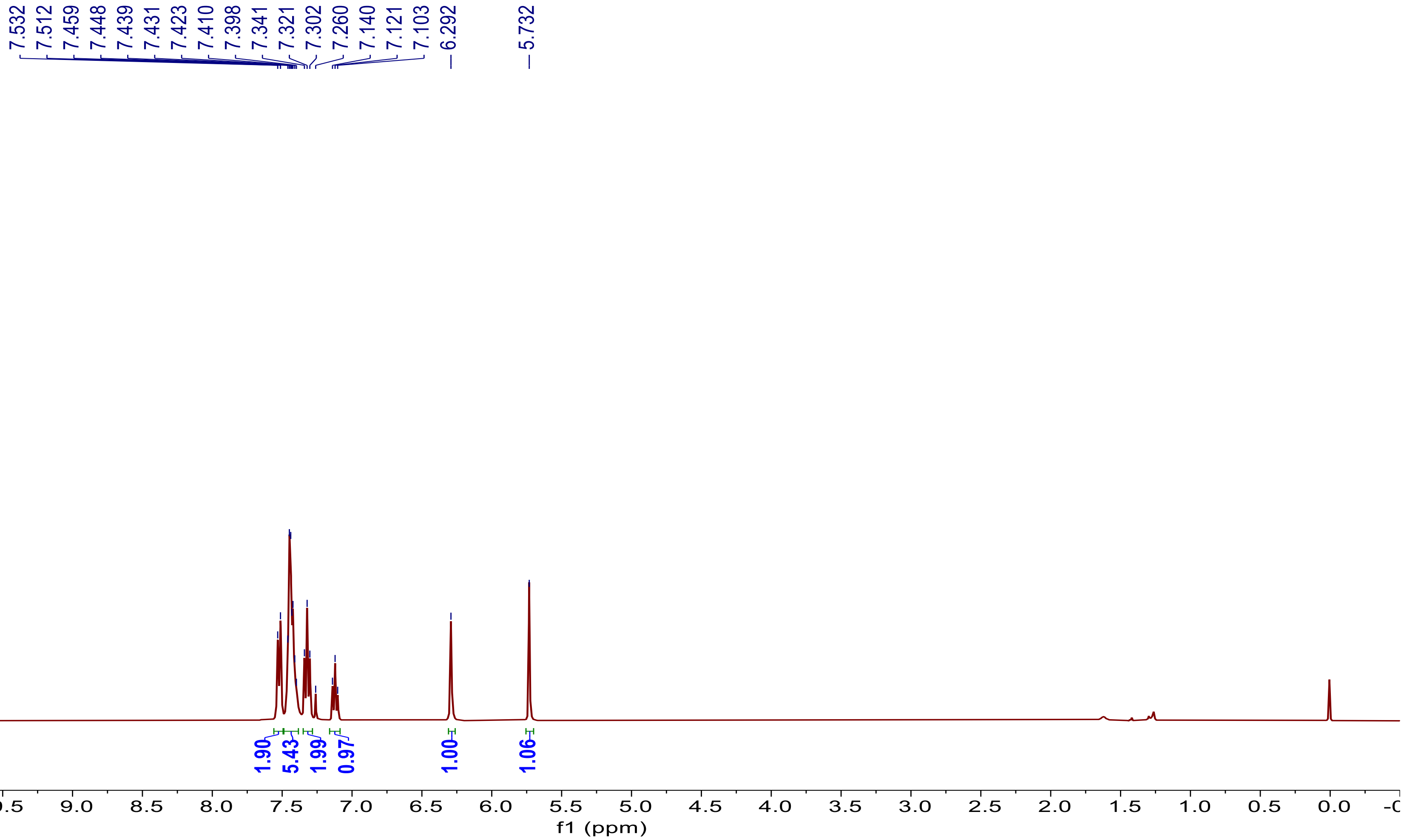

H NMR Spectrum of 9a at 25 °C ($CDCl_3$)

xyk-5-74-0922
Std proton

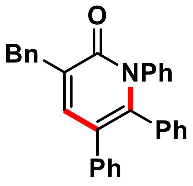

9a

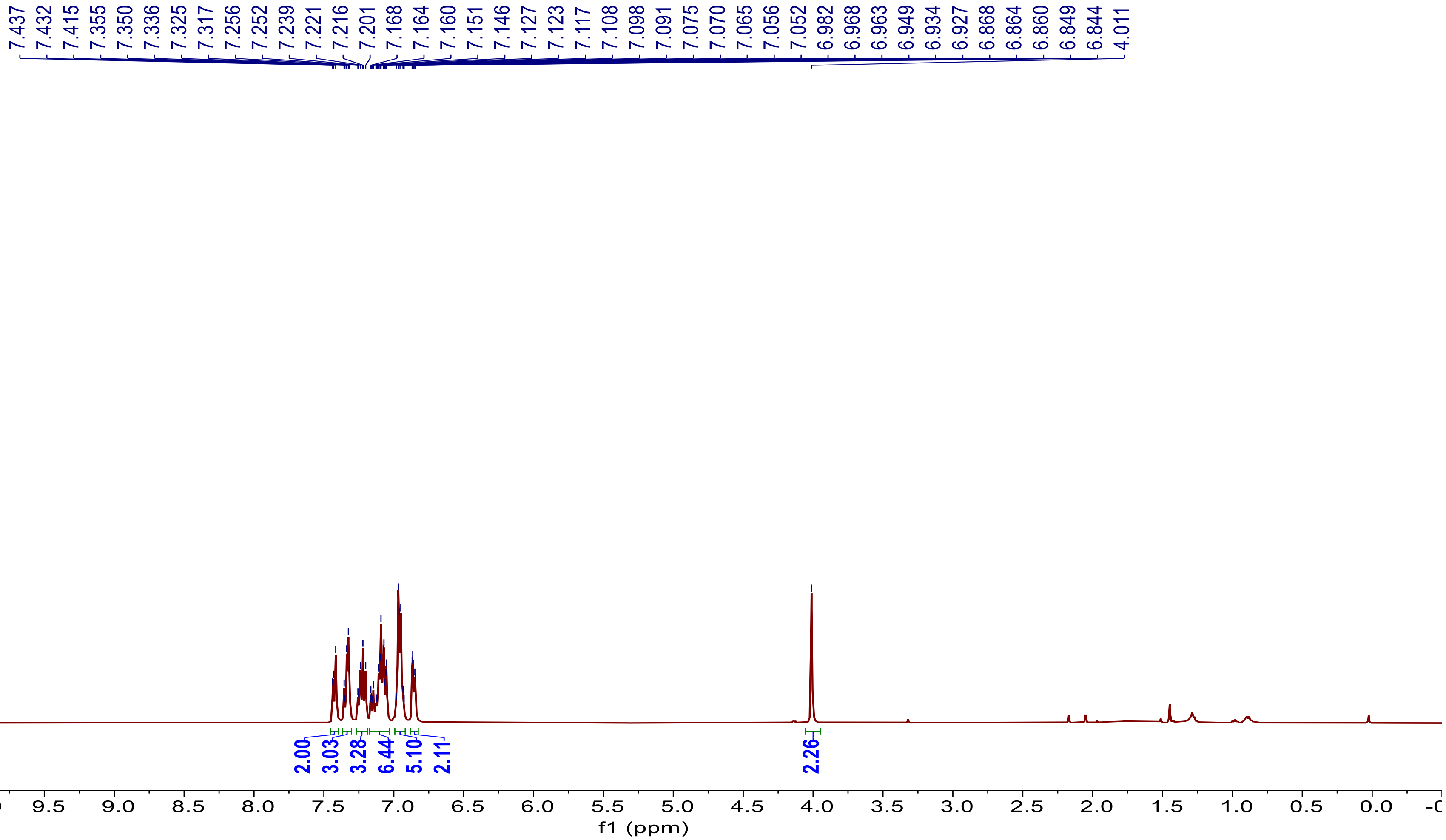

^{13}C NMR Spectrum of 9a at 25 °C ($CDCl_3$)

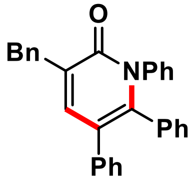

9a

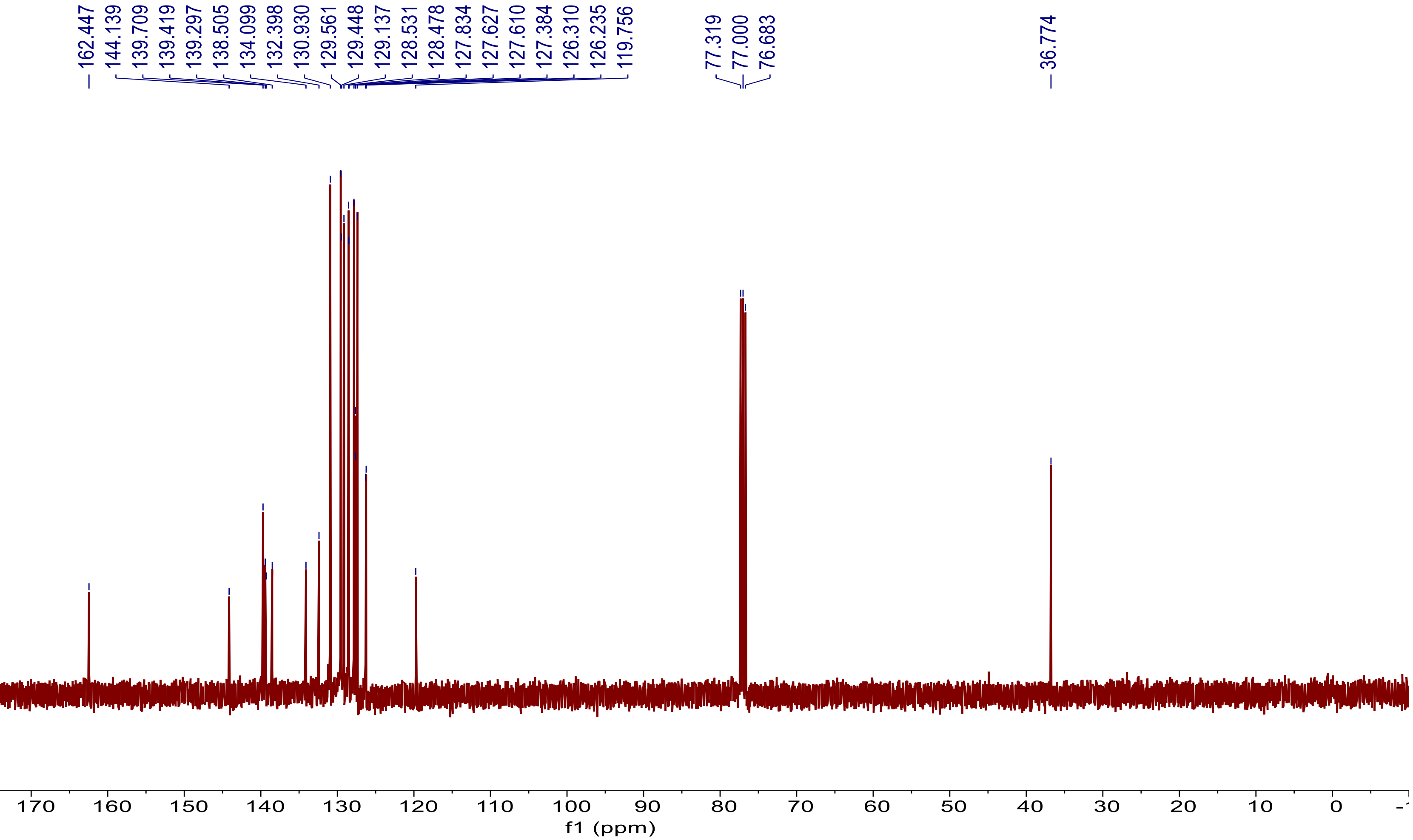

^{1}H NMR Spectrum of 9b at 25 ^{o}C ($CDCl_3$)

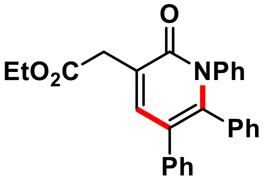

9b

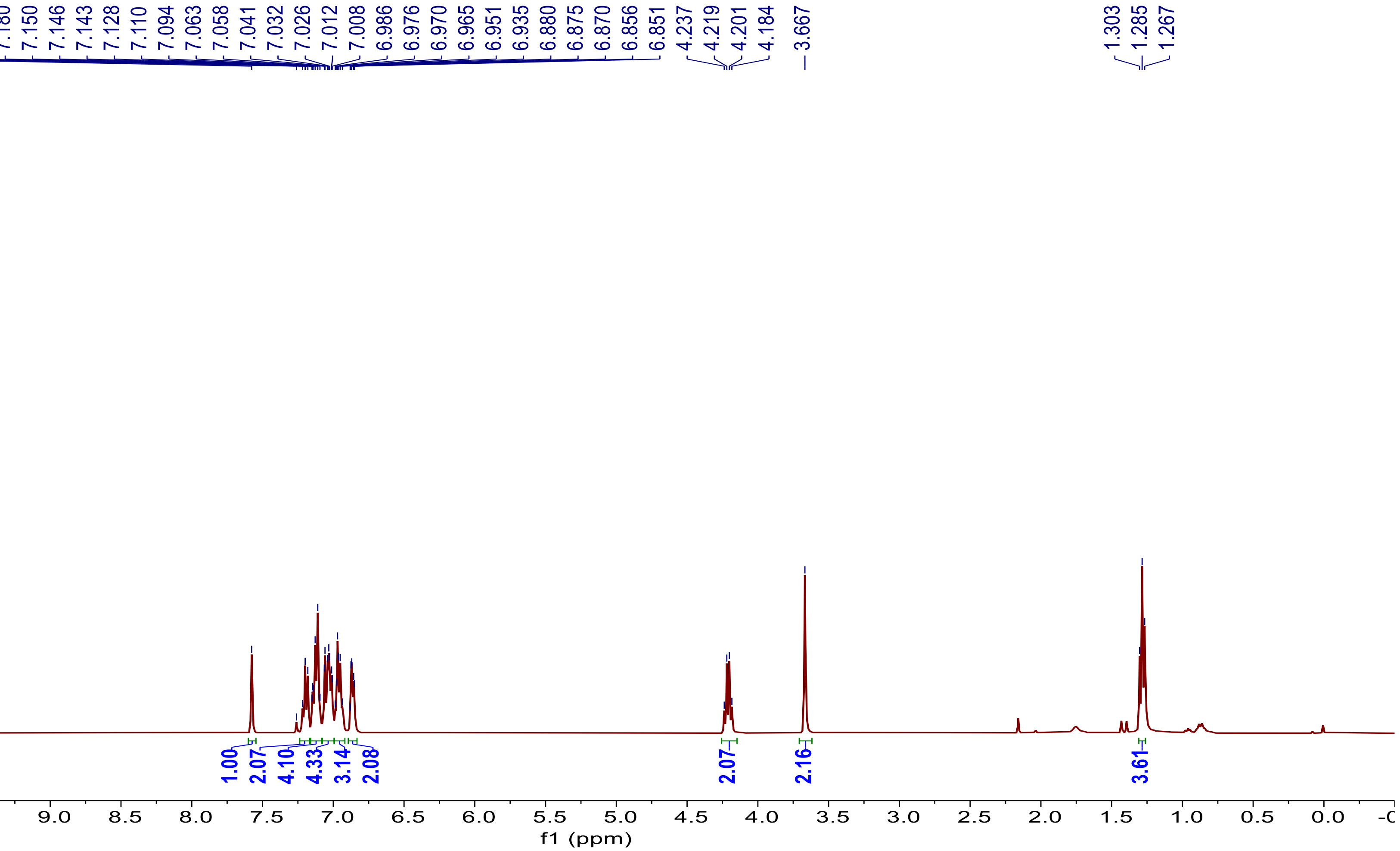

^{13}C NMR Spectrum of 9b at 25 °C ($CDCl_3$)

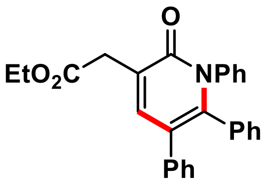

9b

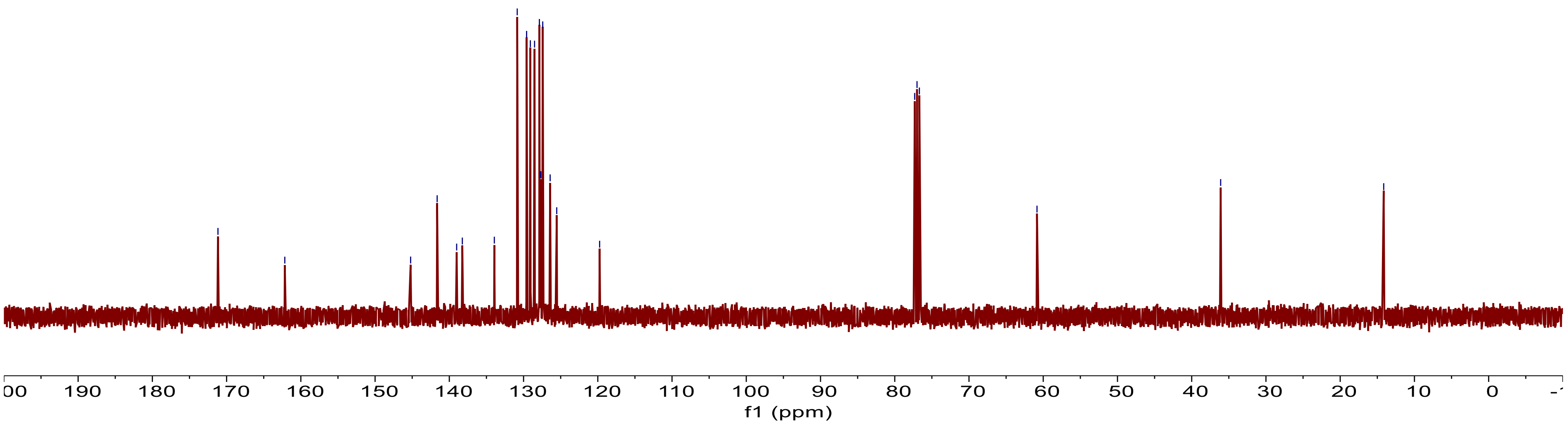

^{1}H NMR Spectrum of 9c at 25 °C ($CDCl_3$)

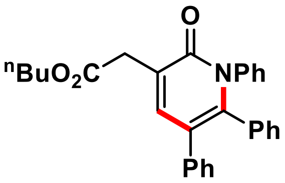

9c

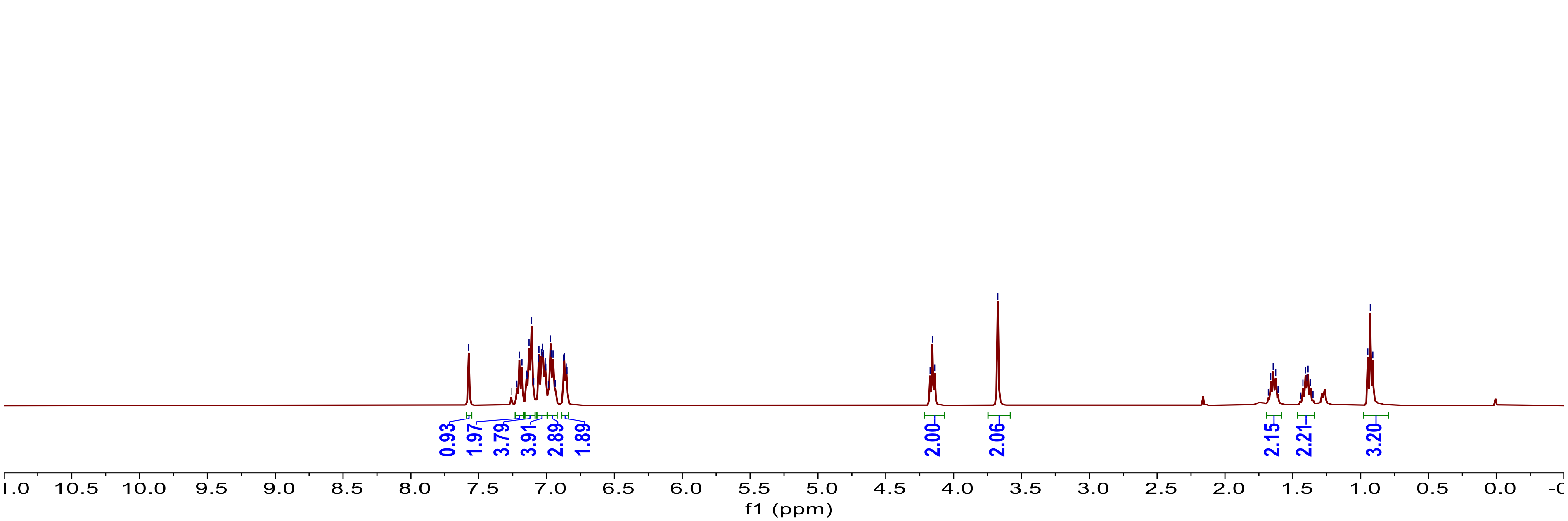

^{13}C NMR Spectrum of 9c at 25 ºC ($CDCl_3$)

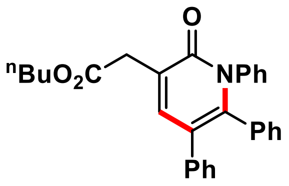

9c

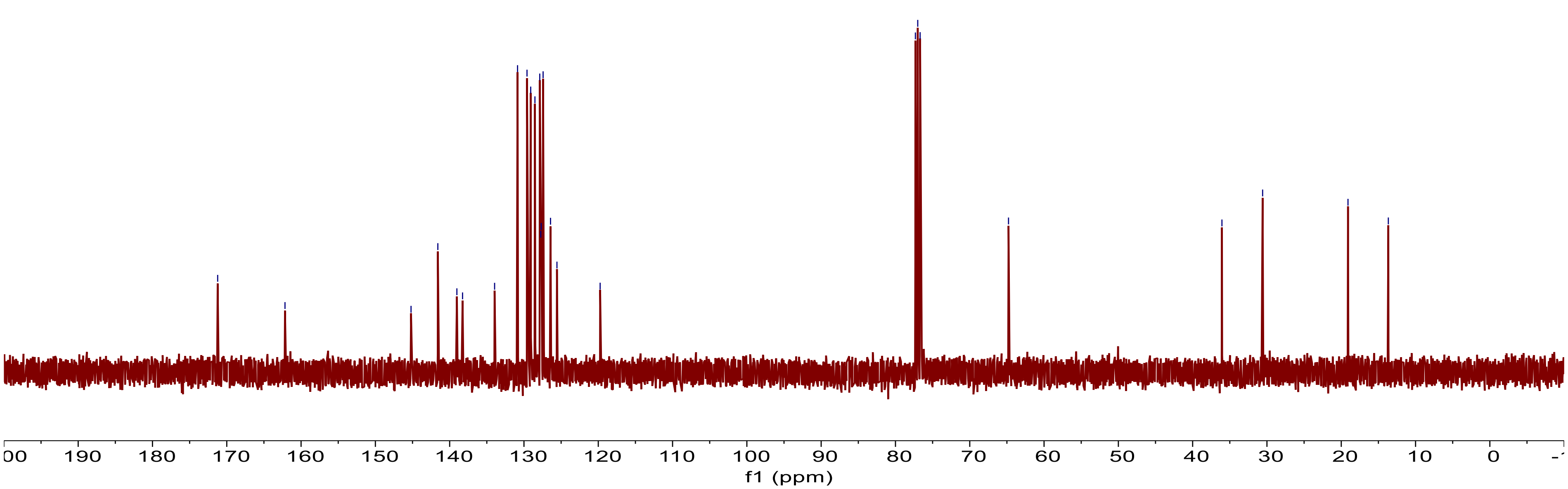

^{1}H NMR Spectrum of 9d at 25 ^{o}C ($CDCl_3$)

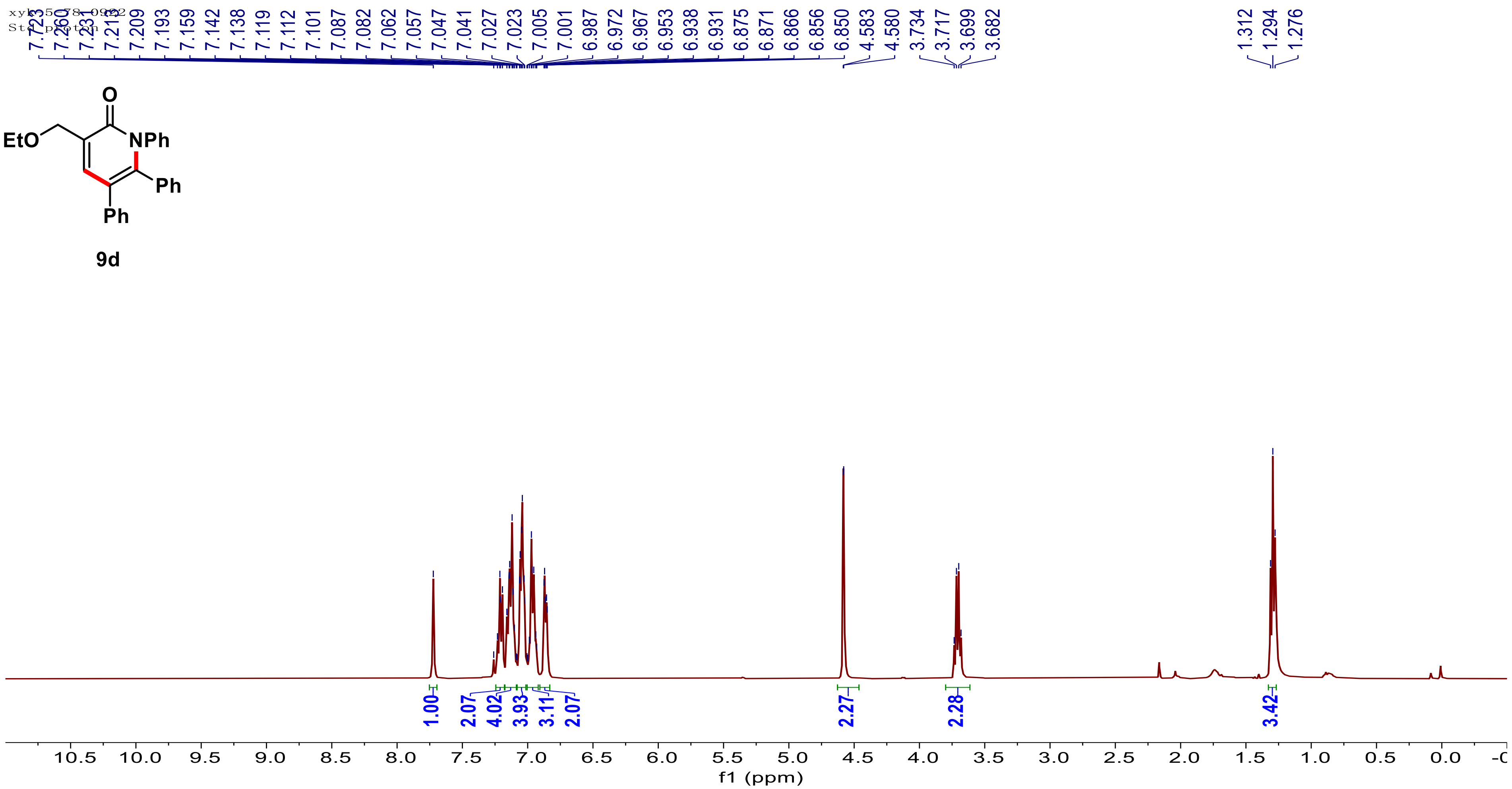

^{13}C NMR Spectrum of 9d at 25 °C ($CDCl_3$)

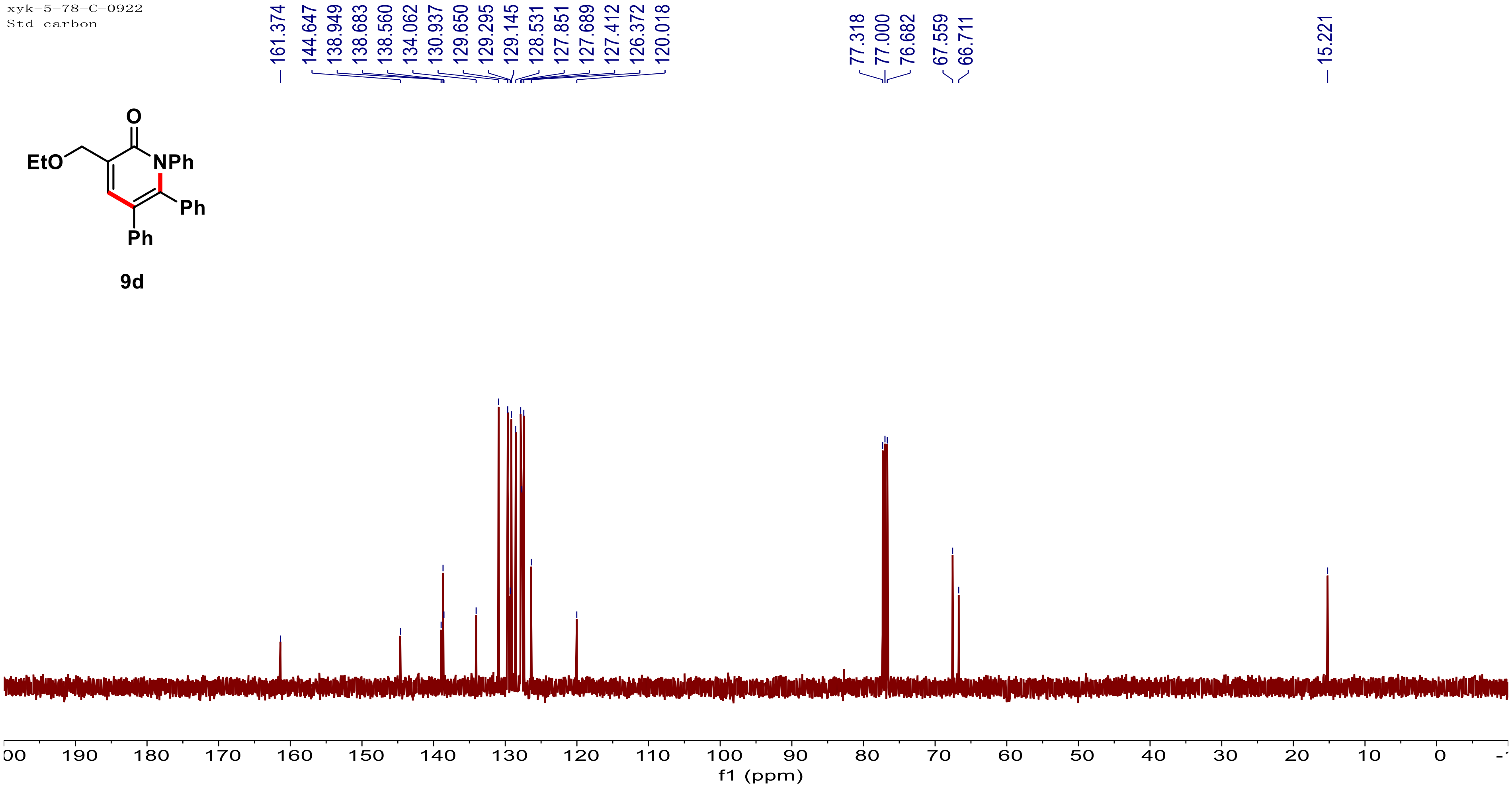

^{1}H NMR Spectrum of 9e at 25 ^{o}C ($CDCl_3$)

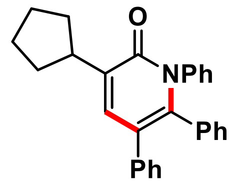

9e

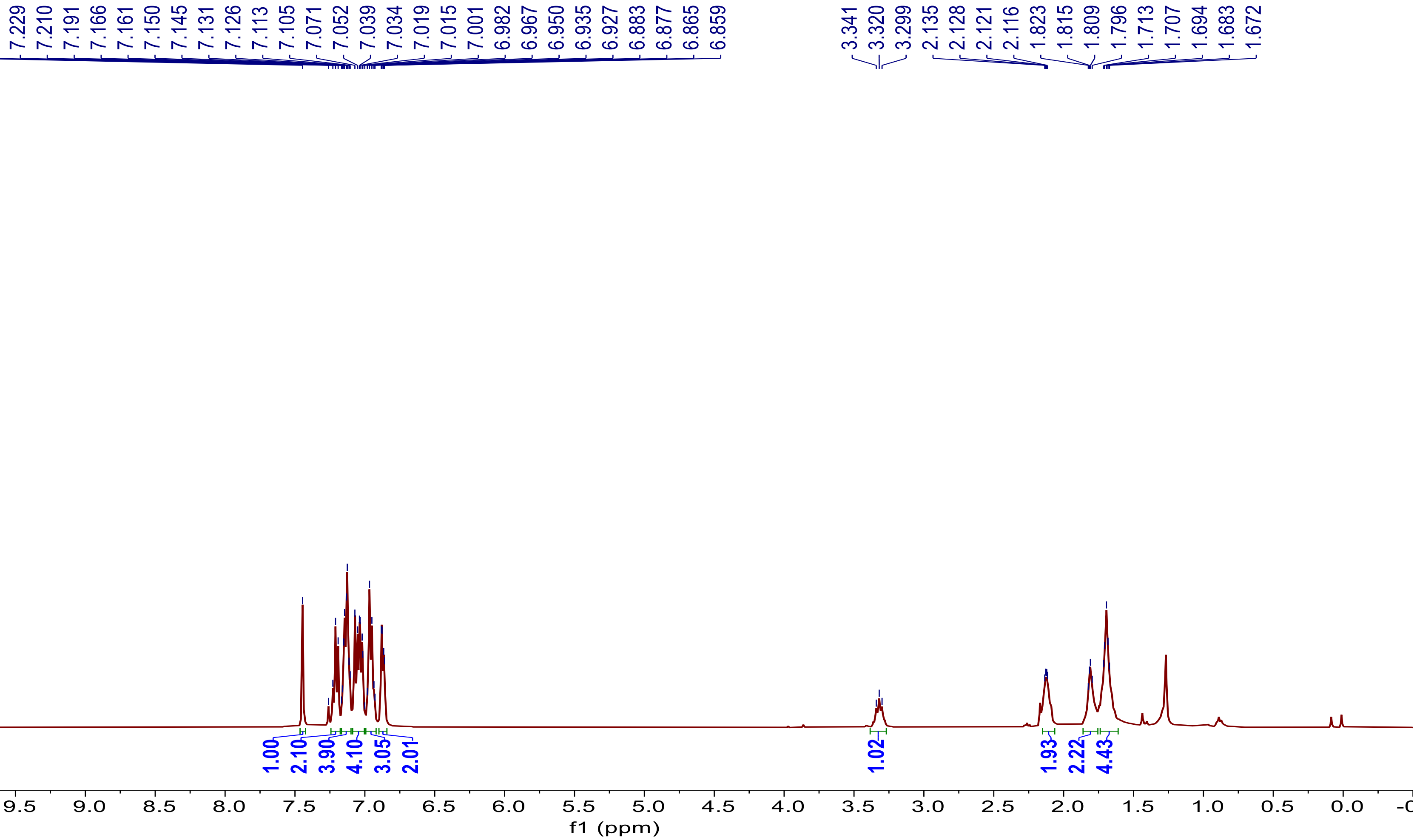

^{13}C NMR Spectrum of 9e at 25 ºC ($CDCl_3$)

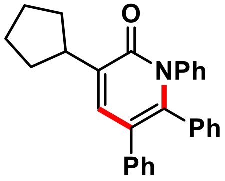

9e

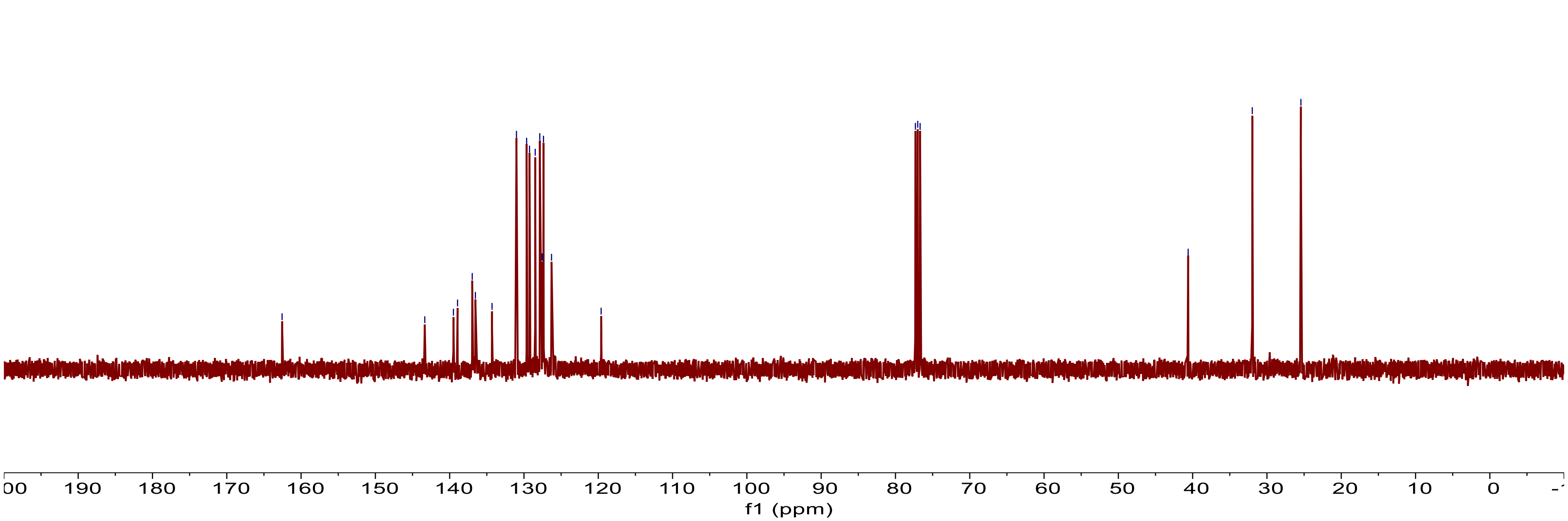

^{1}H NMR Spectrum of 9f at 25 ºC ($CDCl_3$)

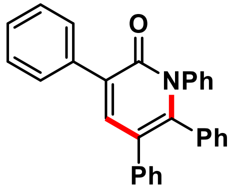

9f

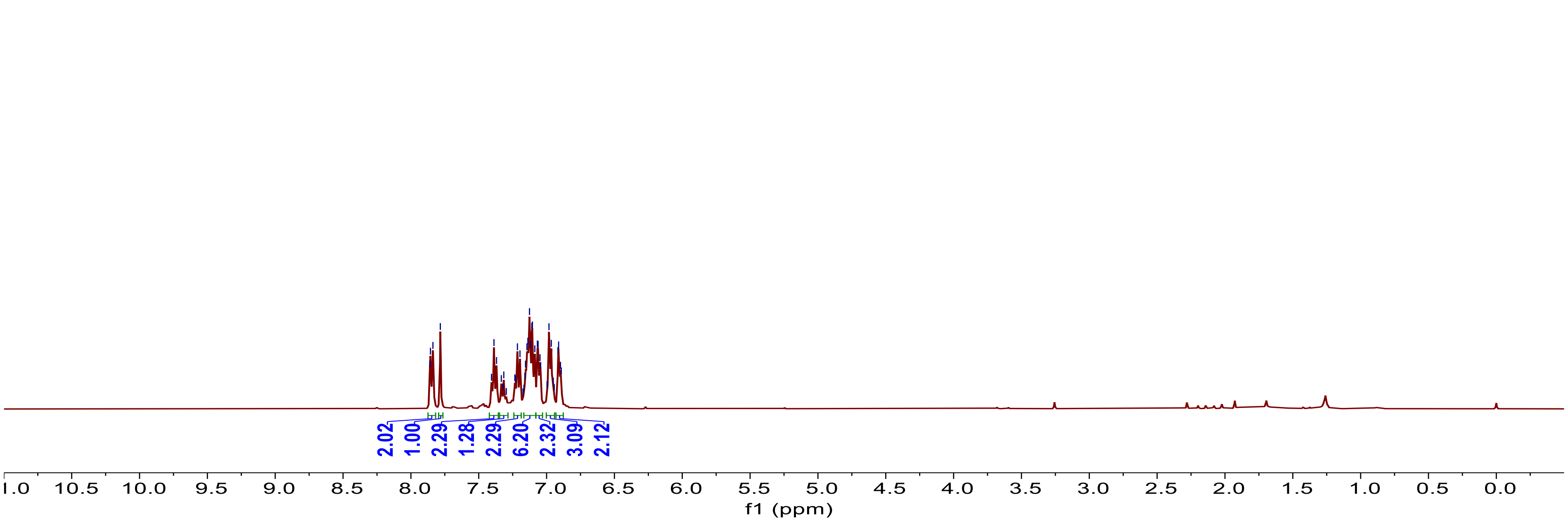

^{13}C NMR Spectrum of 9f at 25 ºC ($CDCl_3$)

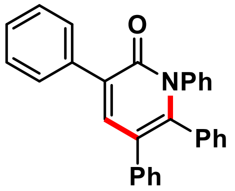

9f

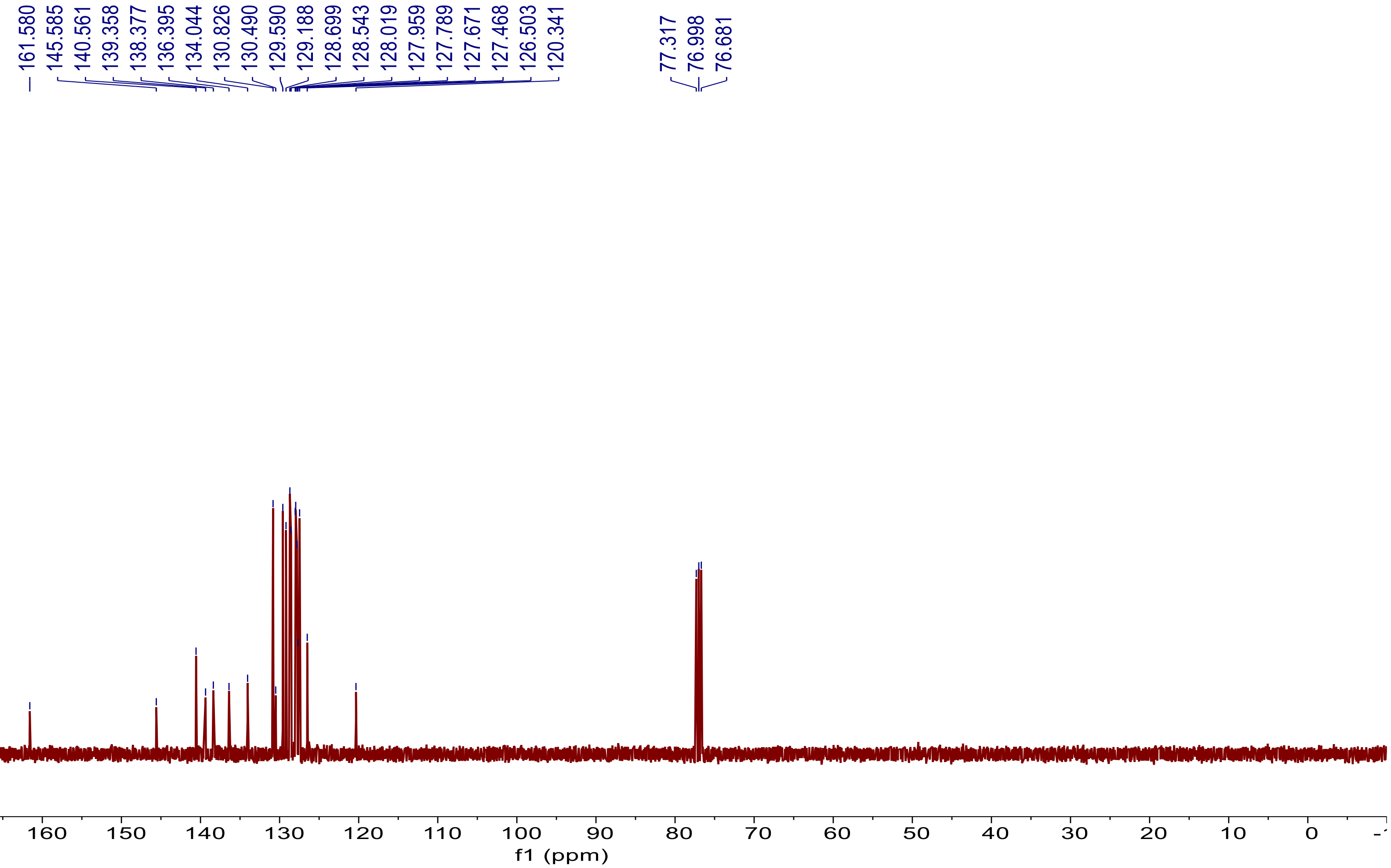

^{1}H NMR Spectrum of 9g at 25 ºC ($CDCl_3$)

xyk-5-53-0902
Std proton

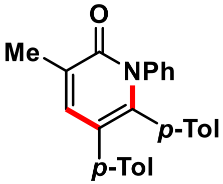

9g

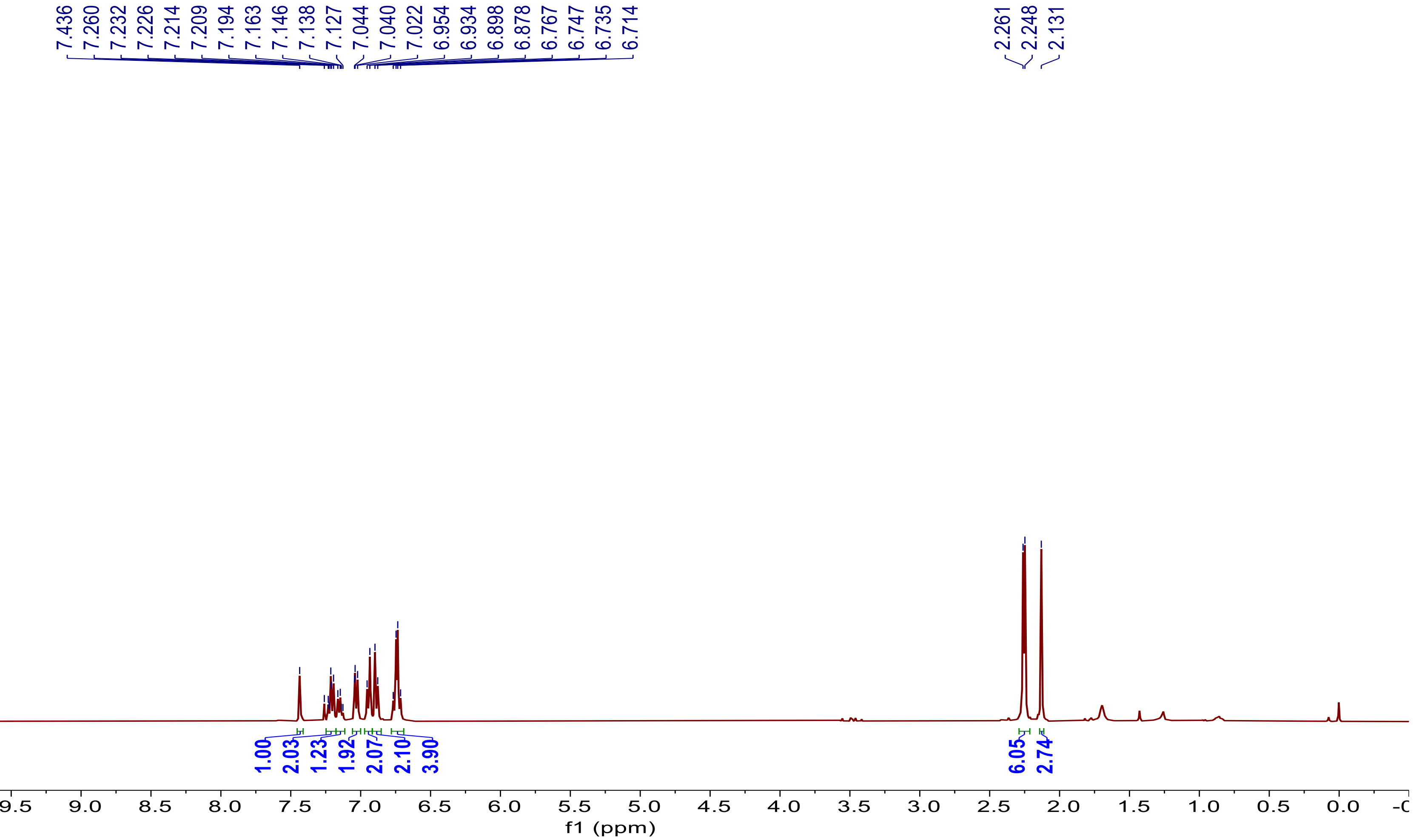

^{13}C NMR Spectrum of 9g at 25 °C ($CDCl_3$)

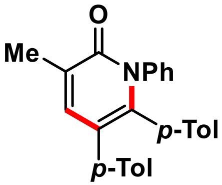

9g

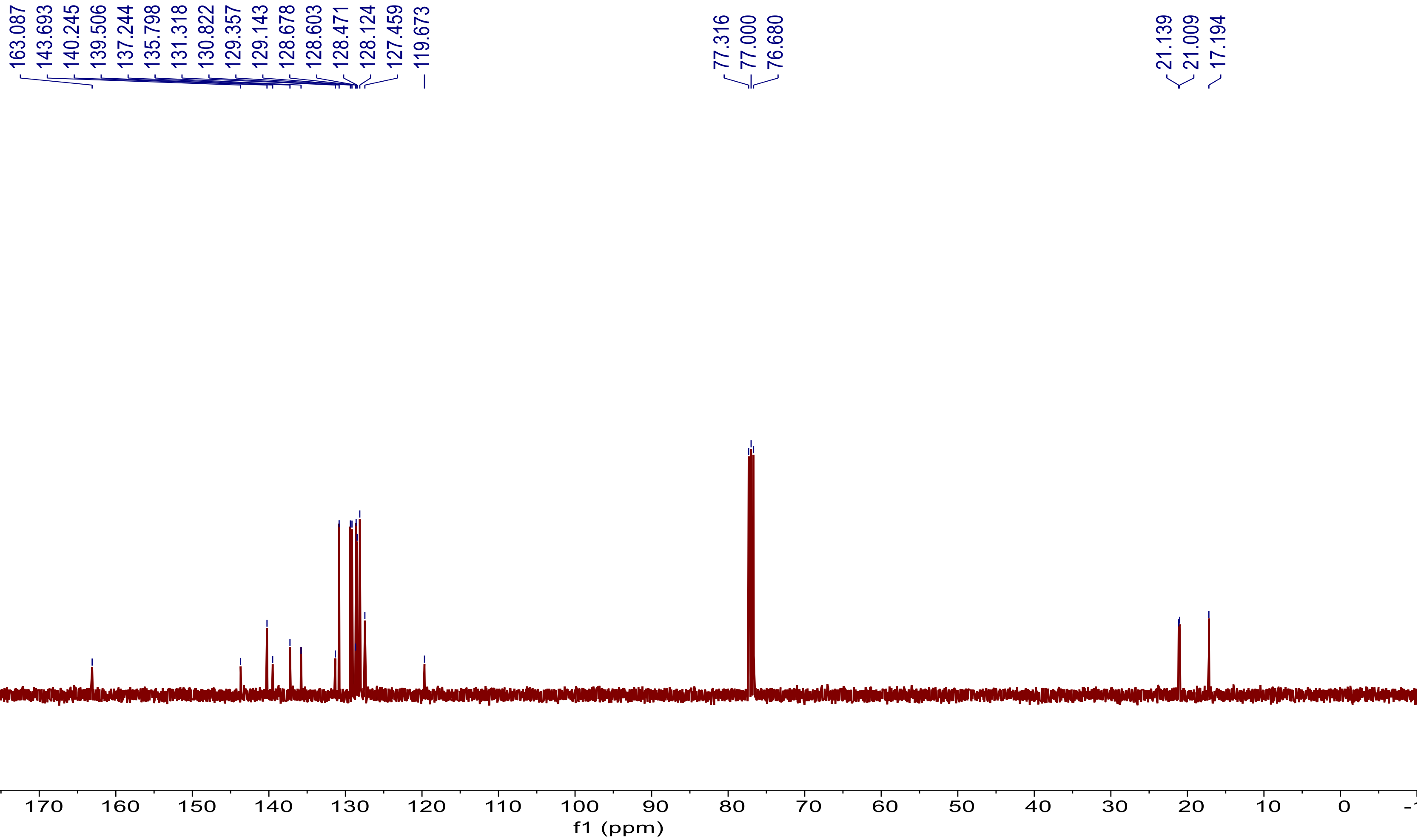

^{1}H NMR Spectrum of 9h at 25 °C ($CDCl_3$)

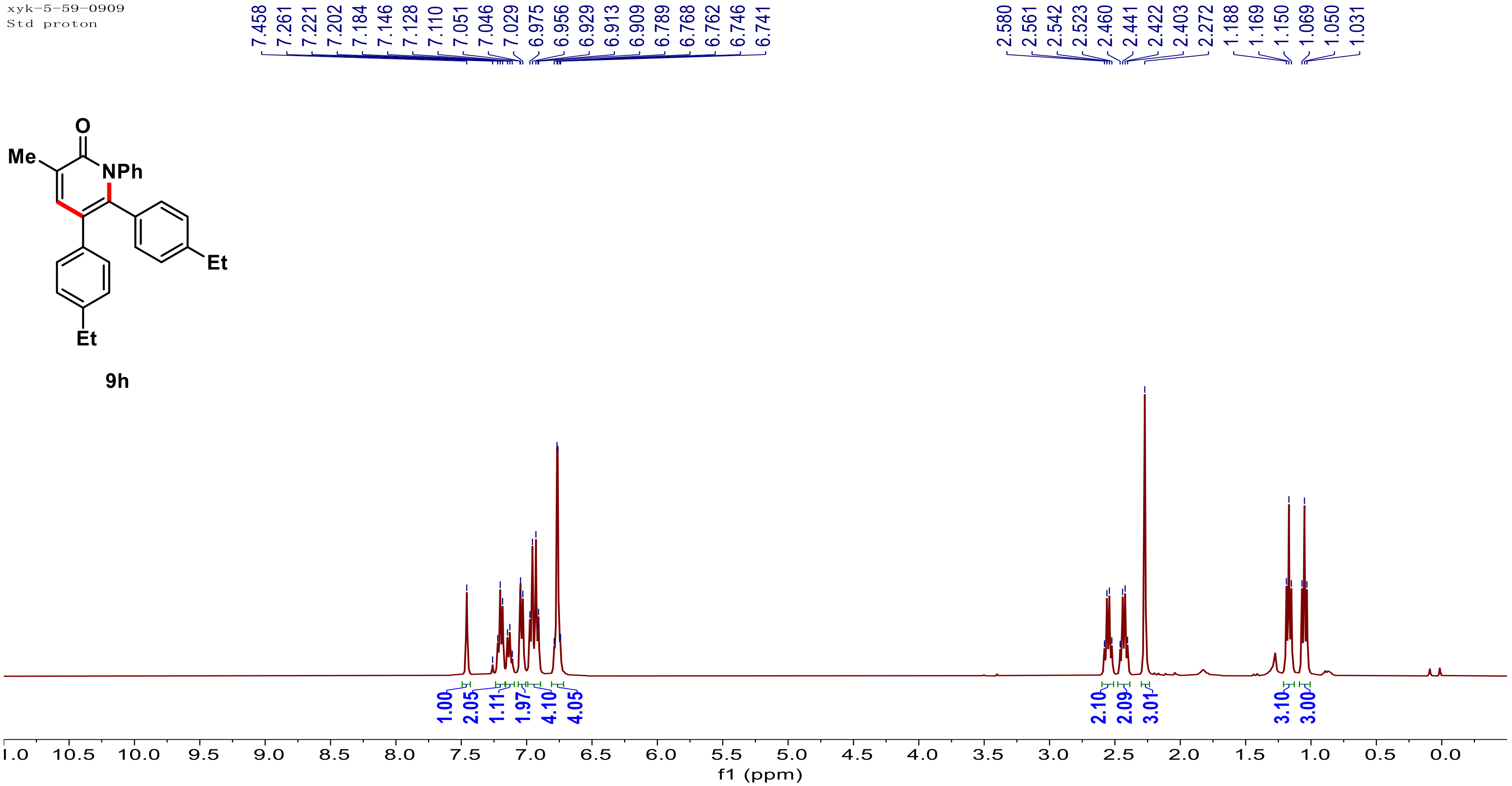

^{13}C NMR Spectrum of 9h at 25 ºC ($CDCl_3$)

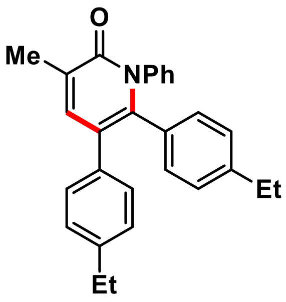

9h

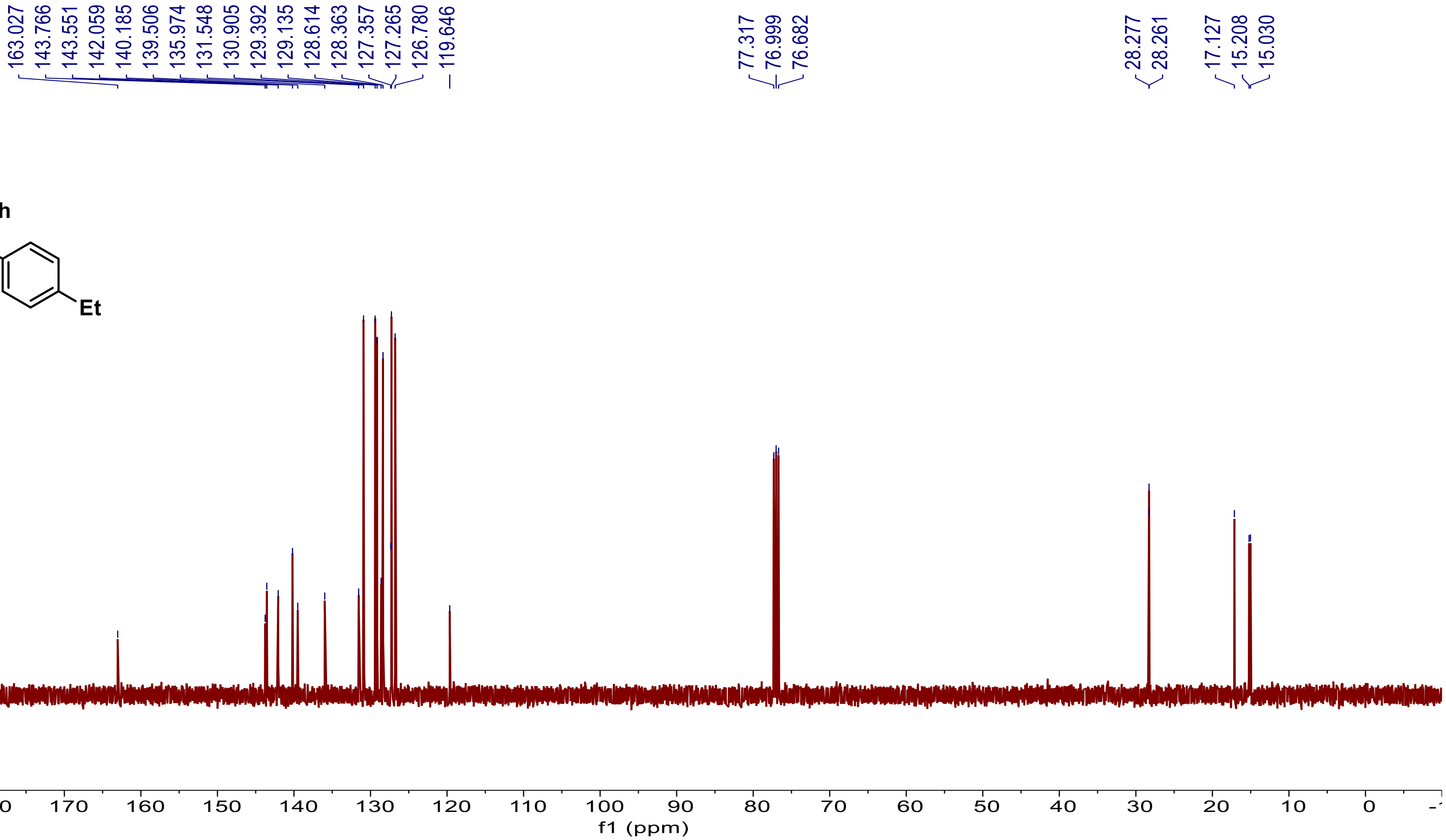

^{1}H NMR Spectrum of 9i at 25 ºC ($CDCl_3$)

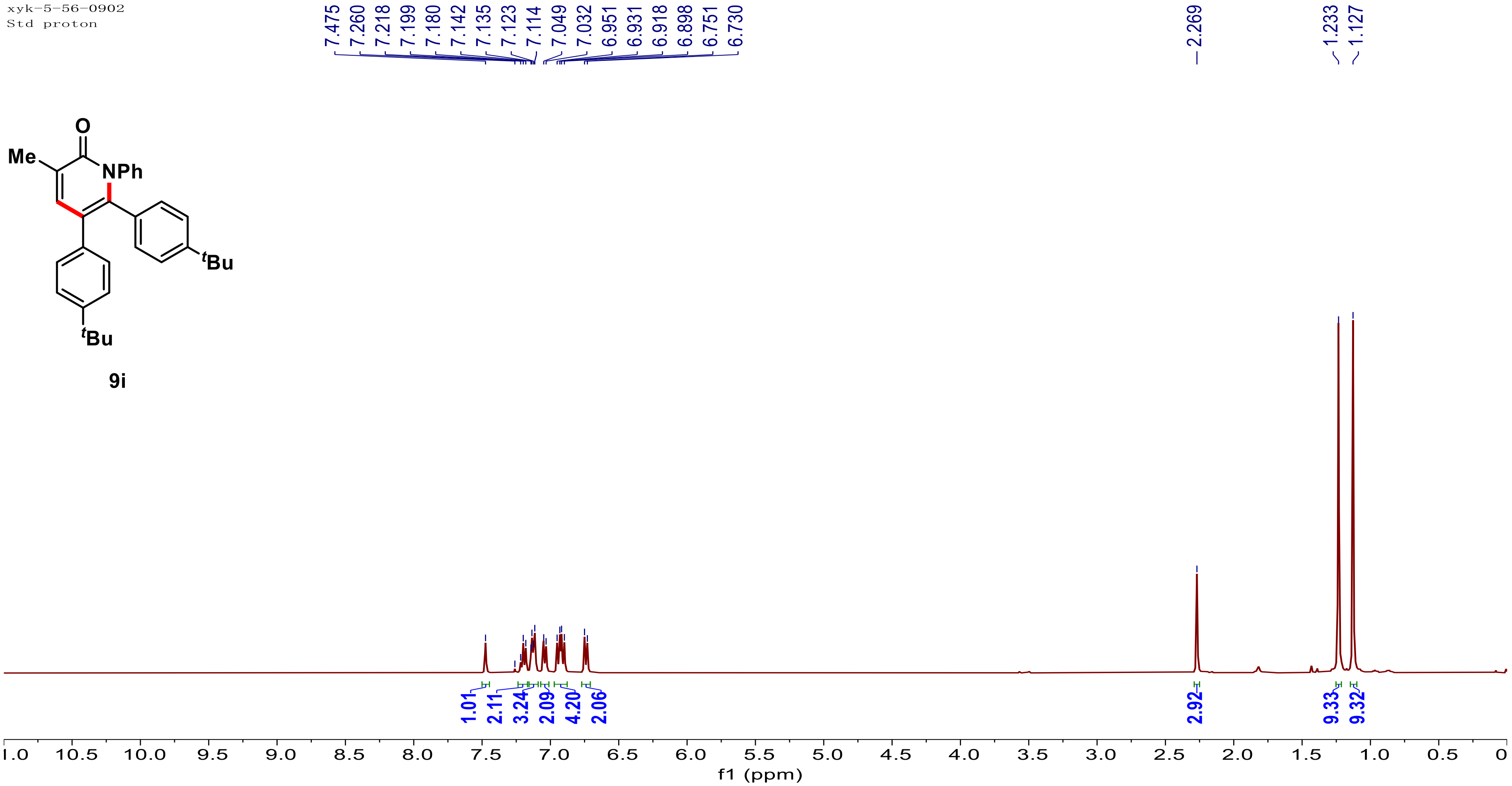

^{13}C NMR Spectrum of 9i at 25 °C ($CDCl_3$)

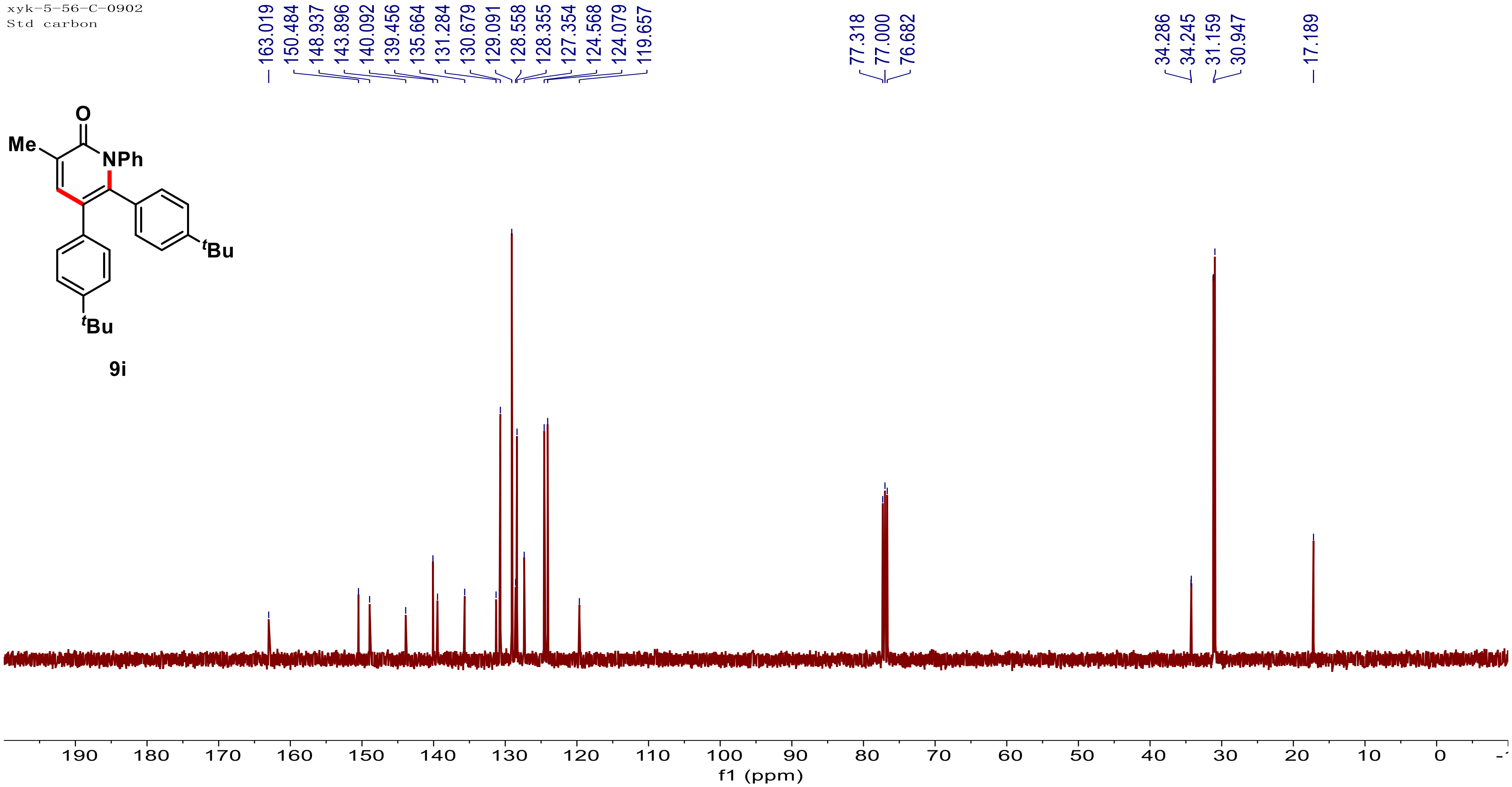

9i

^{1}H NMR Spectrum of 9j at 25 ºC ($CDCl_3$)

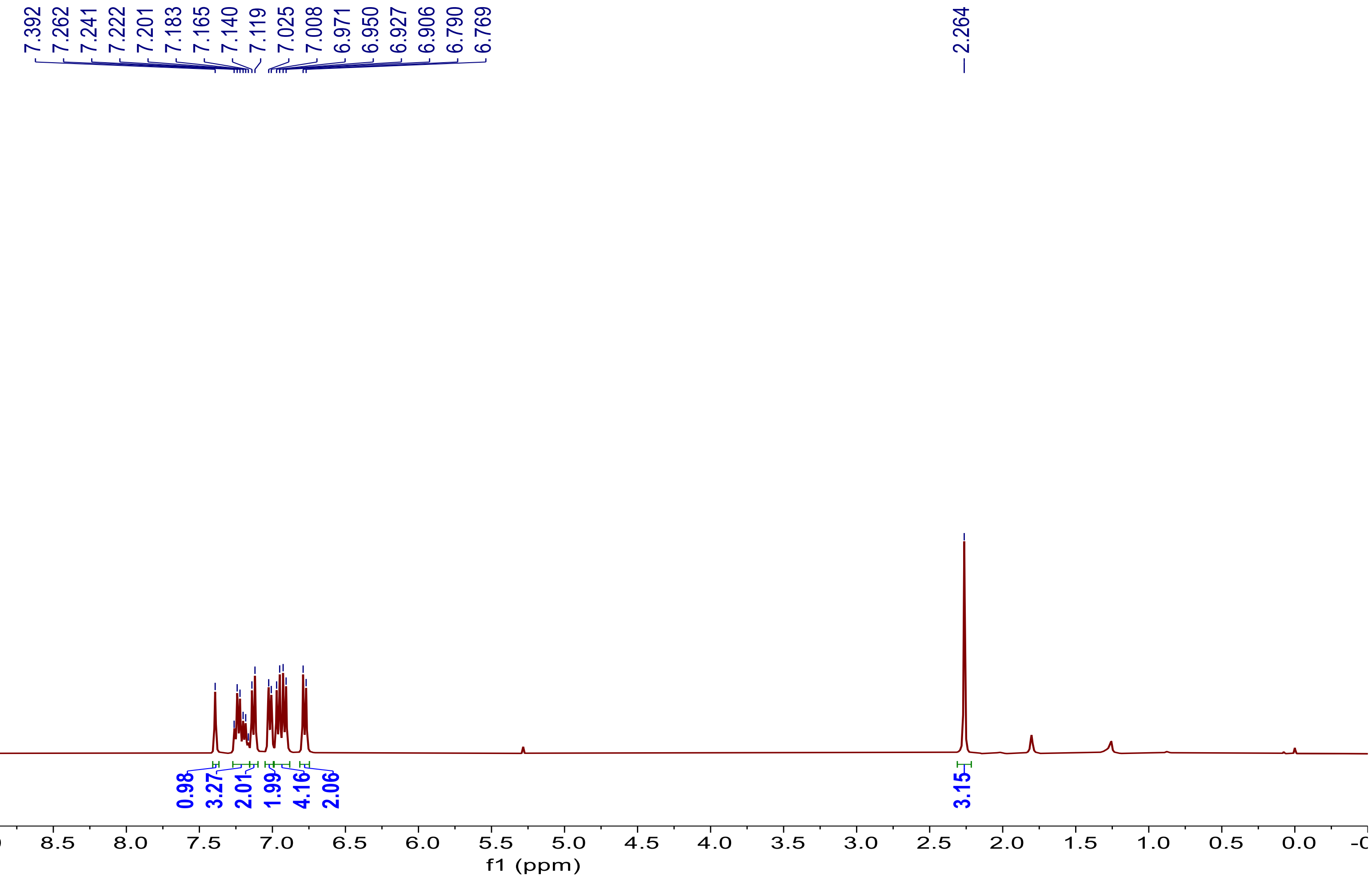

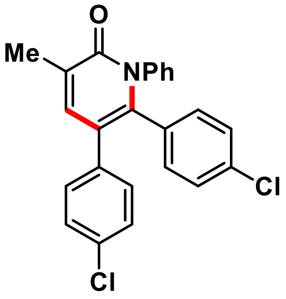

9j

^{13}C NMR Spectrum of 9j at 25 °C ($CDCl_3$)

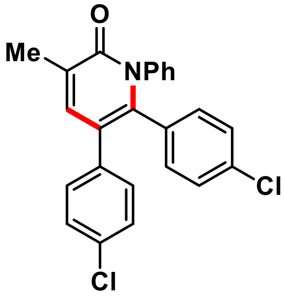

9j

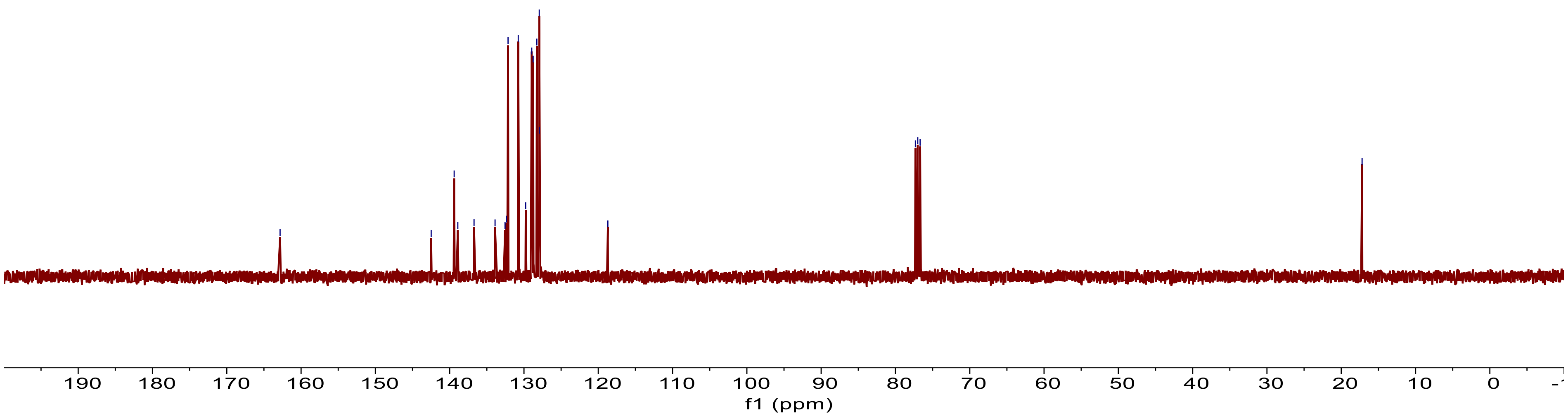

^{1}H NMR Spectrum of 9k at 25 ^{o}C ($CDCl_3$)

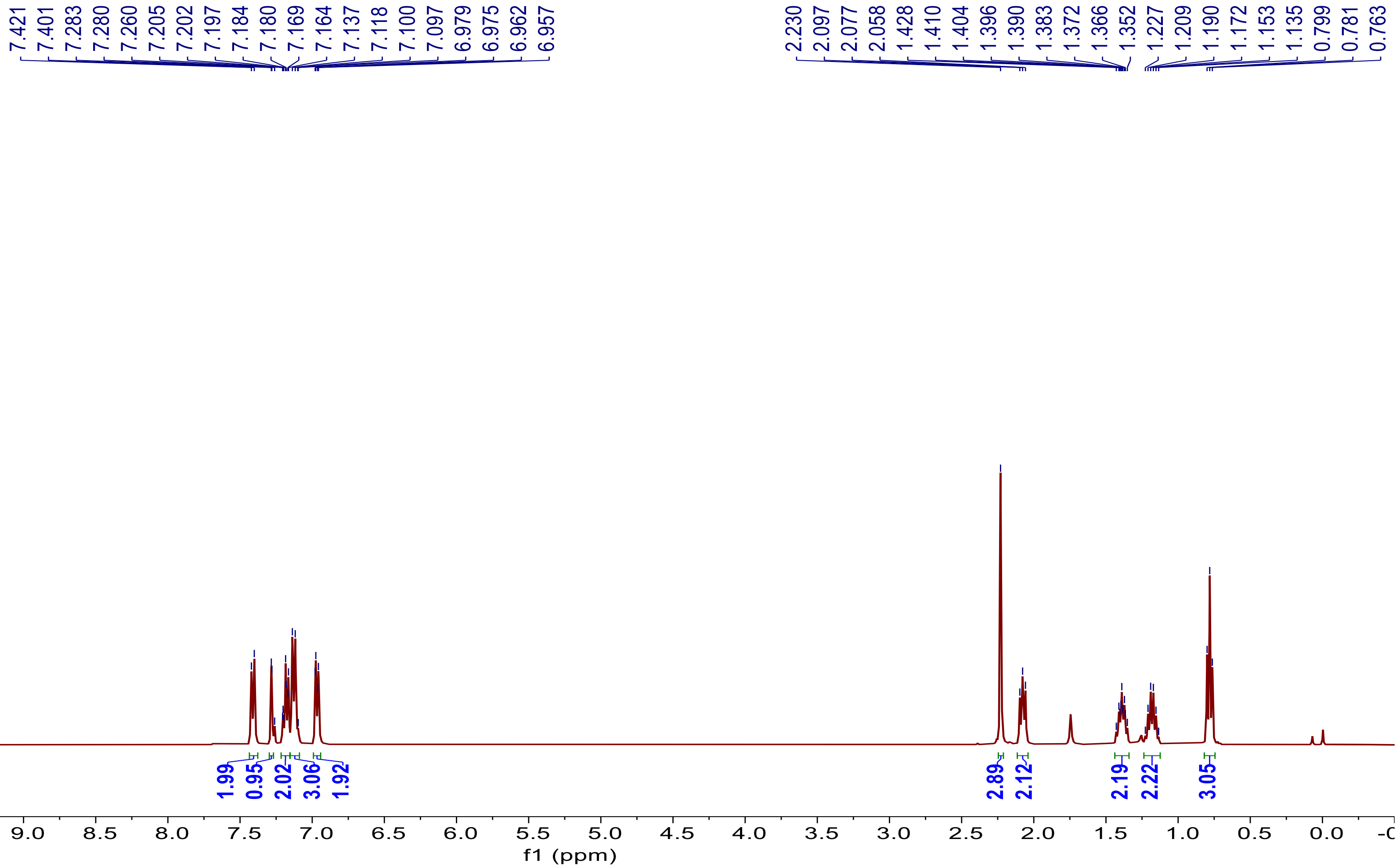

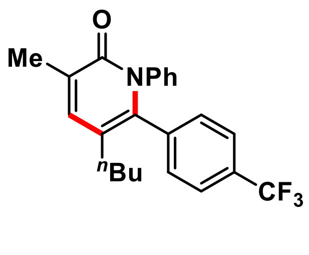

9k

^{13}C NMR Spectrum of 9k at 25 °C ($CDCl_3$)

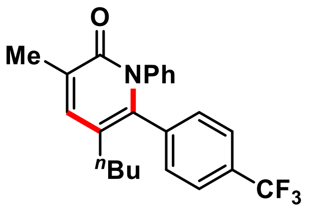

9k

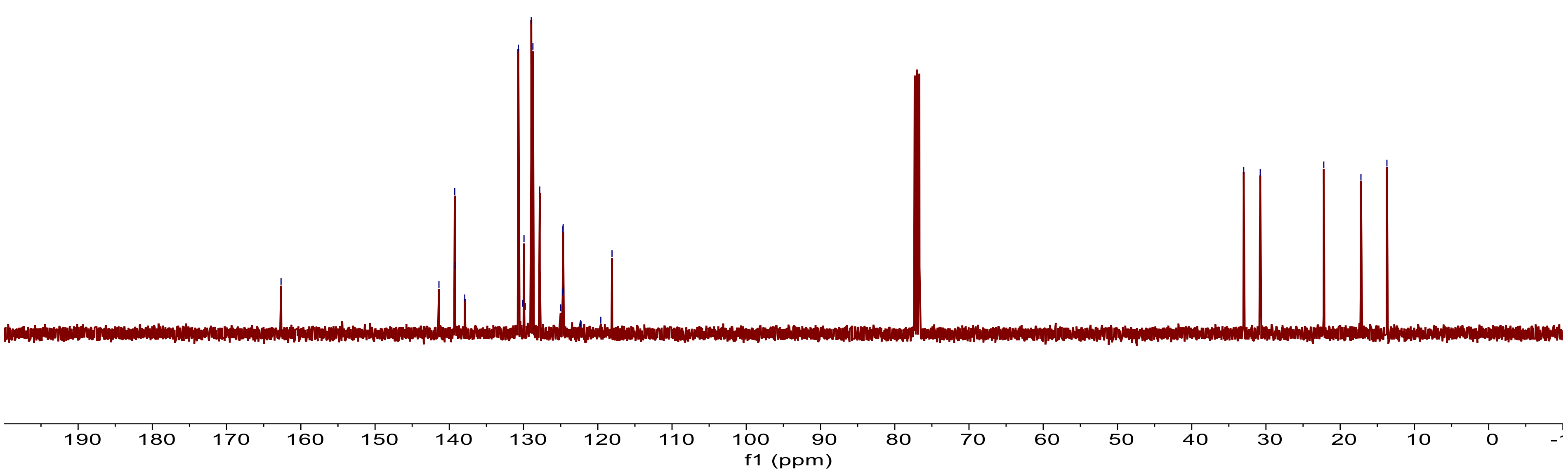

^{19}F NMR Spectrum of 9k at 25 °C ($CDCl_3$)

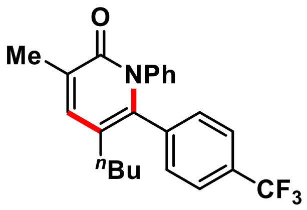

9k

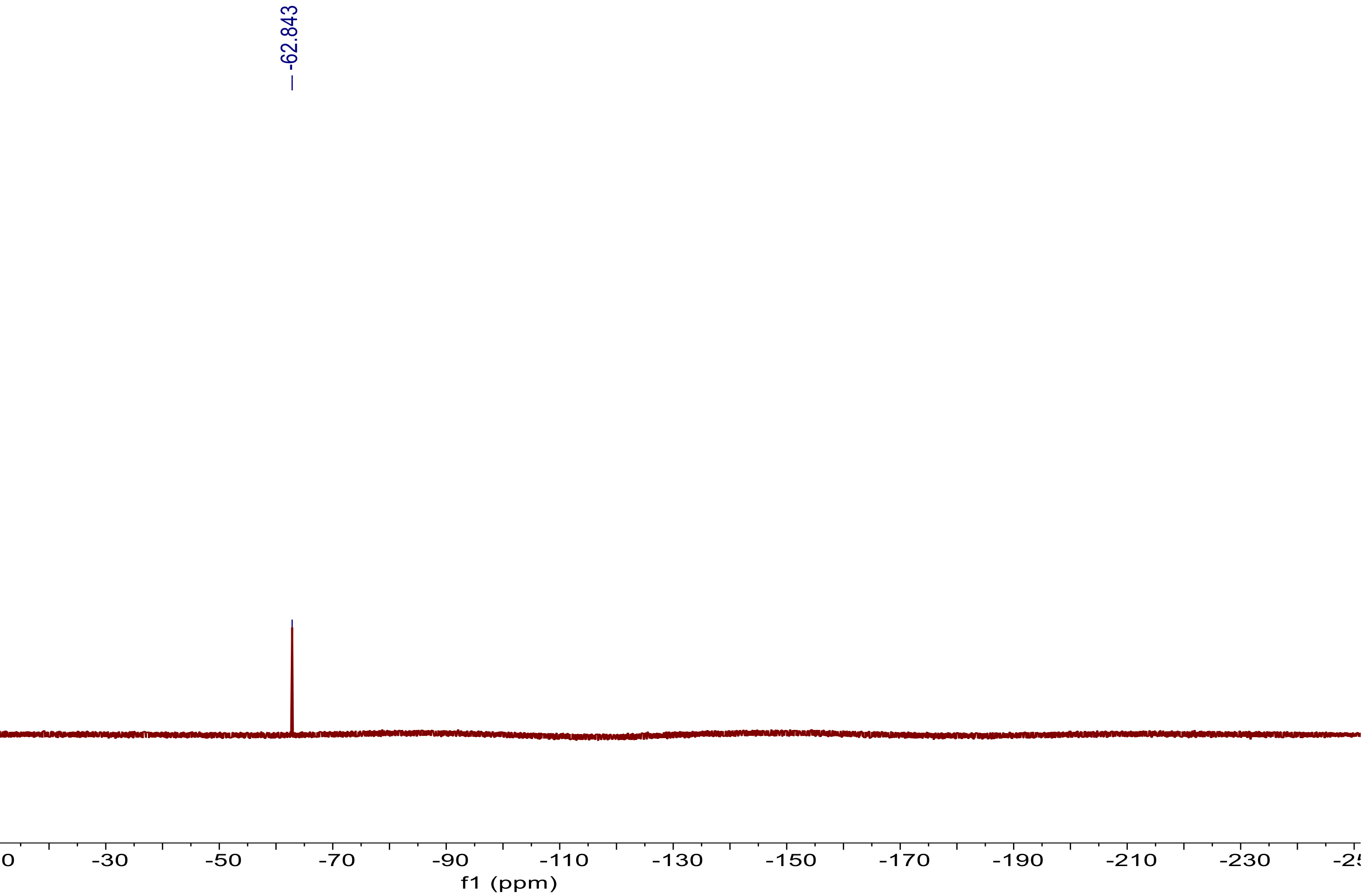

^{1}H NMR Spectrum of 9l at 25 ºC ($CDCl_3$)

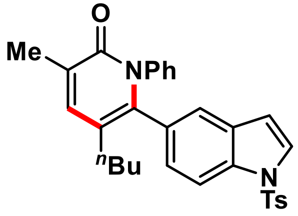

9l

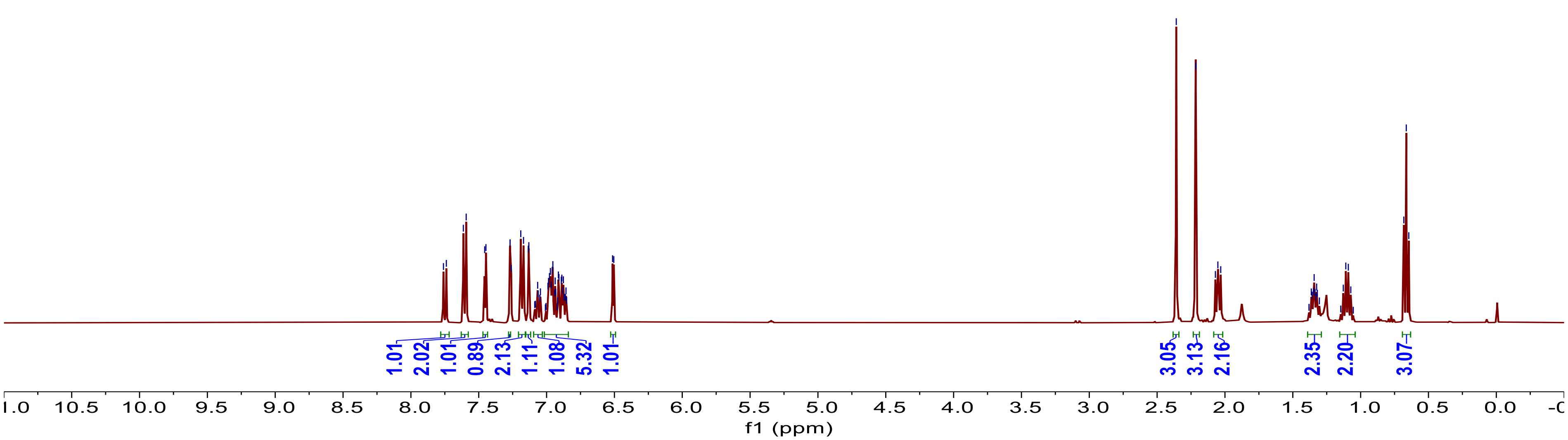

^{13}C NMR Spectrum of 9l at 25 °C ($CDCl_3$)

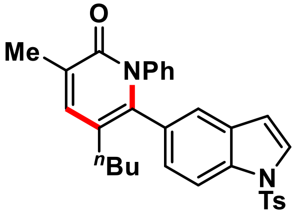

9l

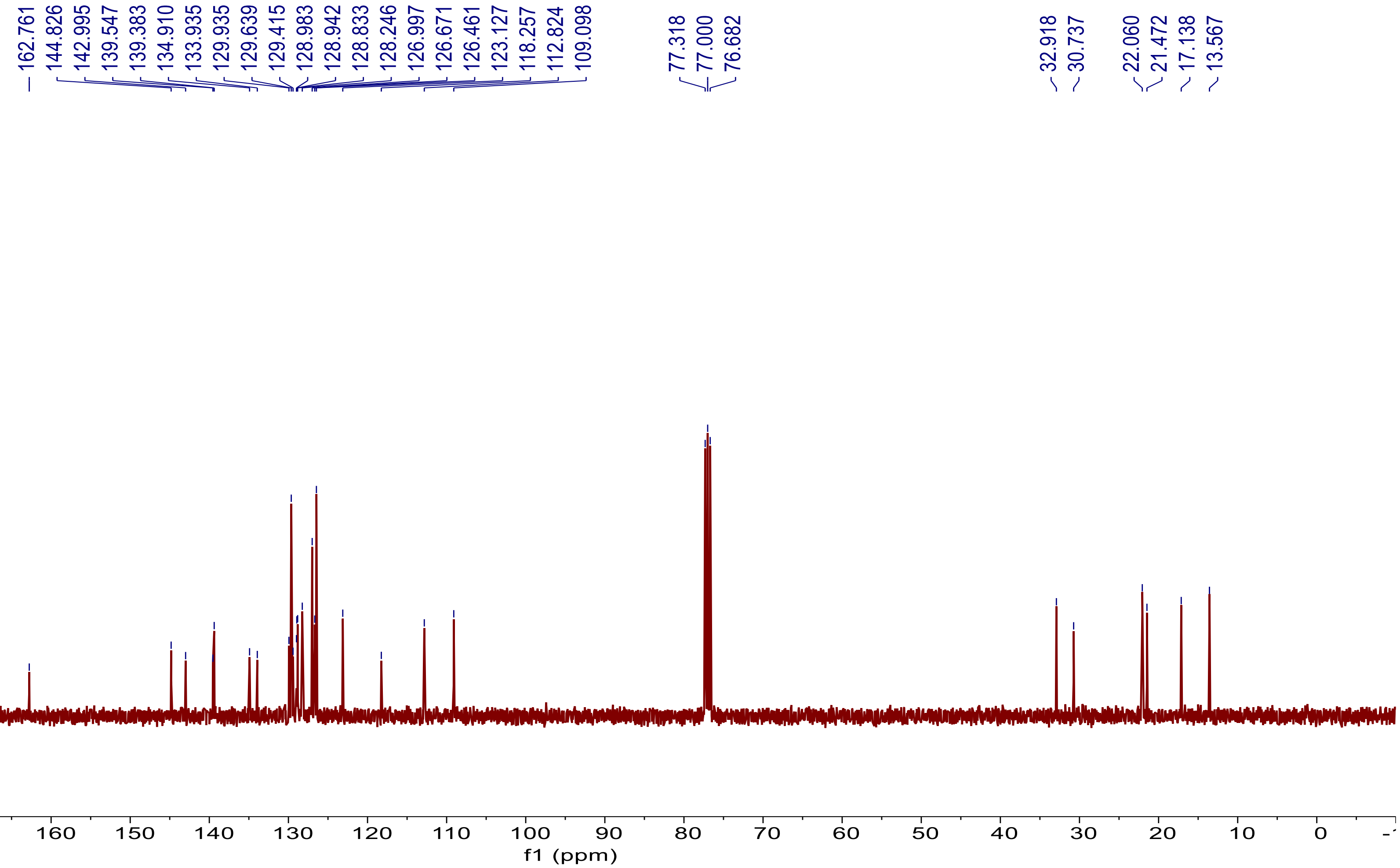

^{1}H NMR Spectrum of 10 at 25 °C (CDCl$_3$)

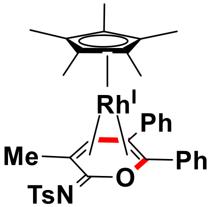

10

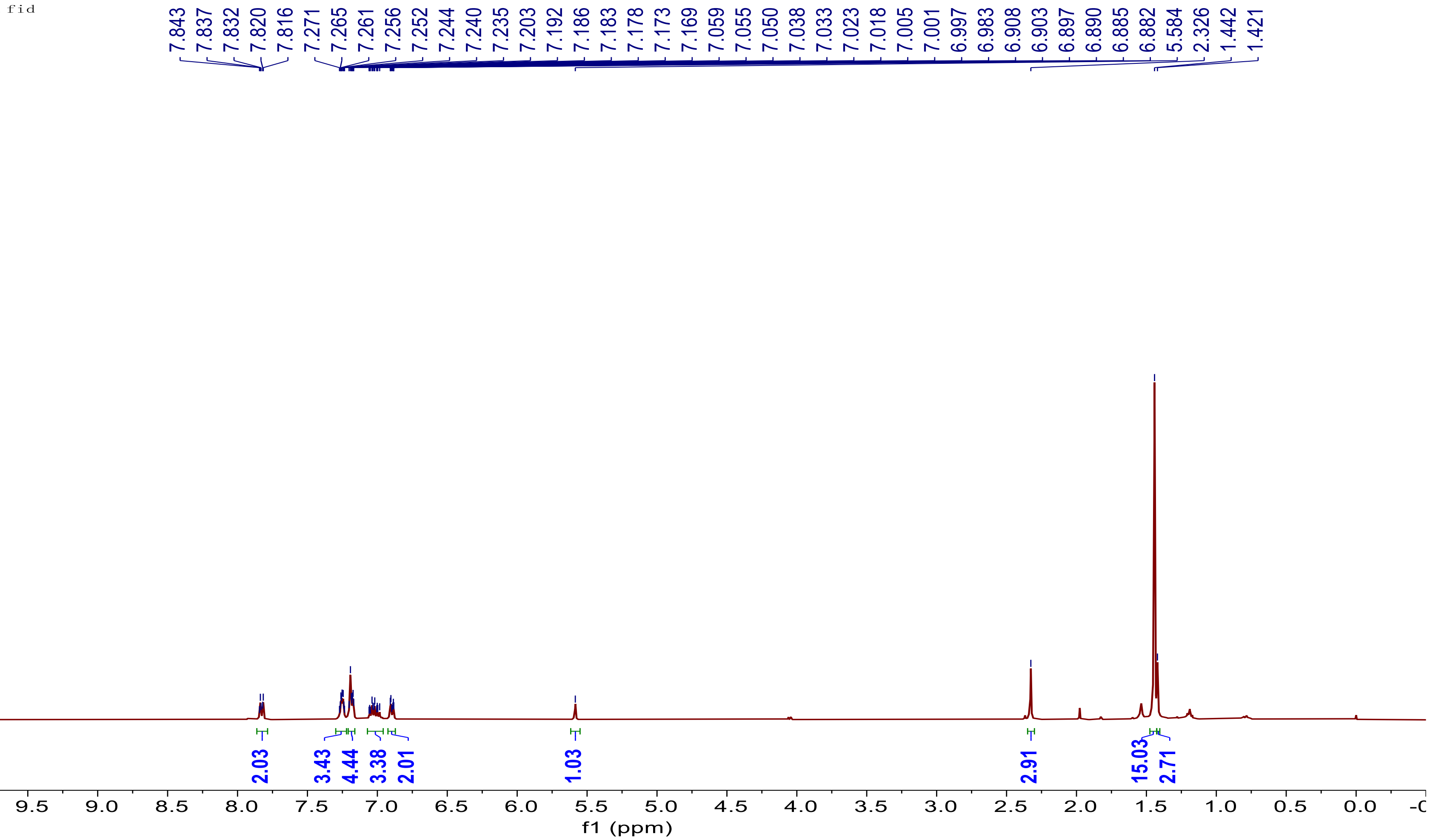

^{13}C NMR Spectrum of 10 at 25 ^{o}C ($CDCl_3$)

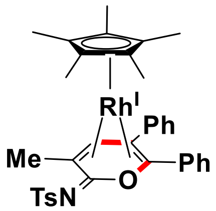

10

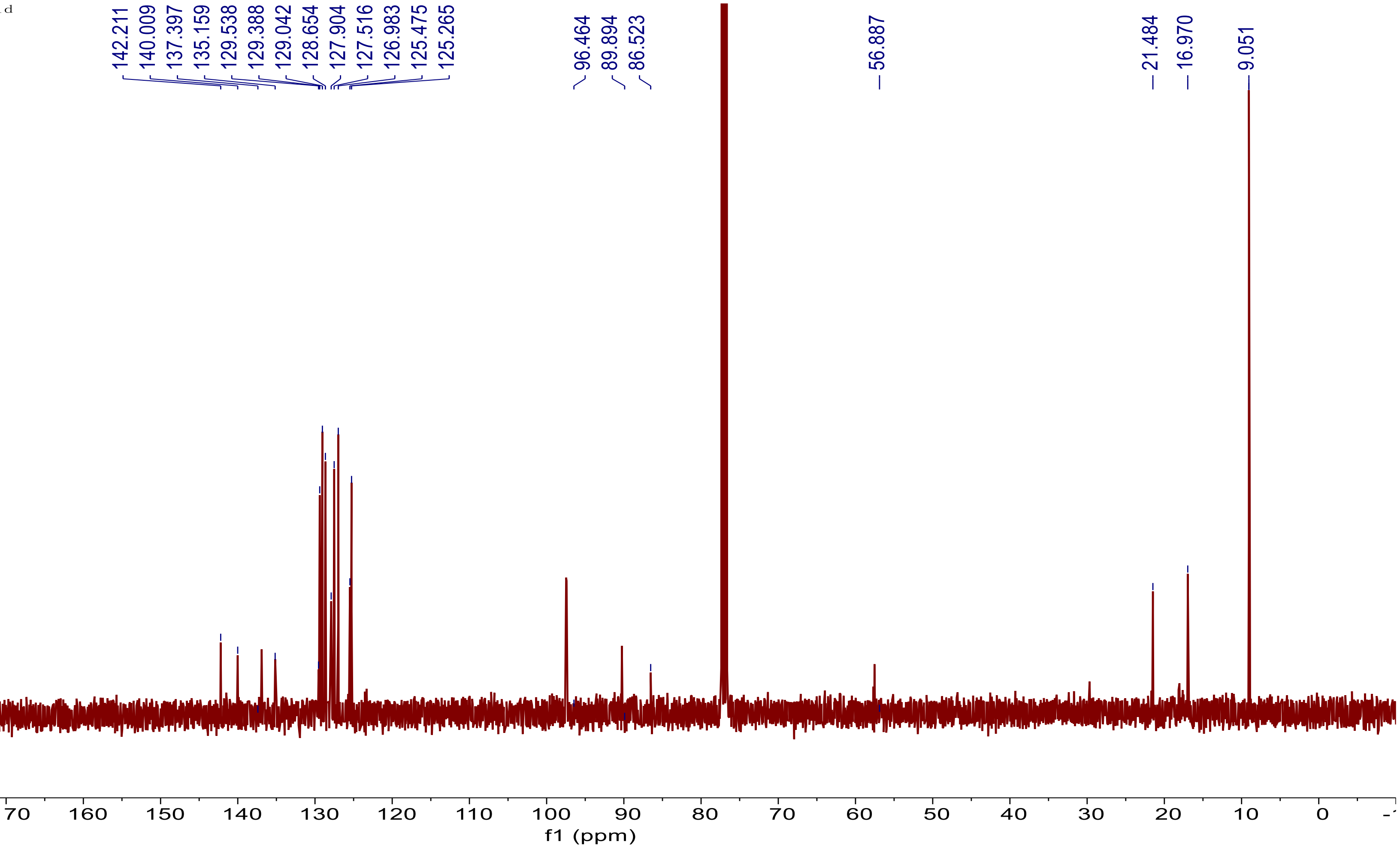

^{1}H NMR Spectrum of 11 at 25 °C ($CDCl_3$)

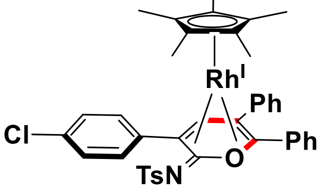

11

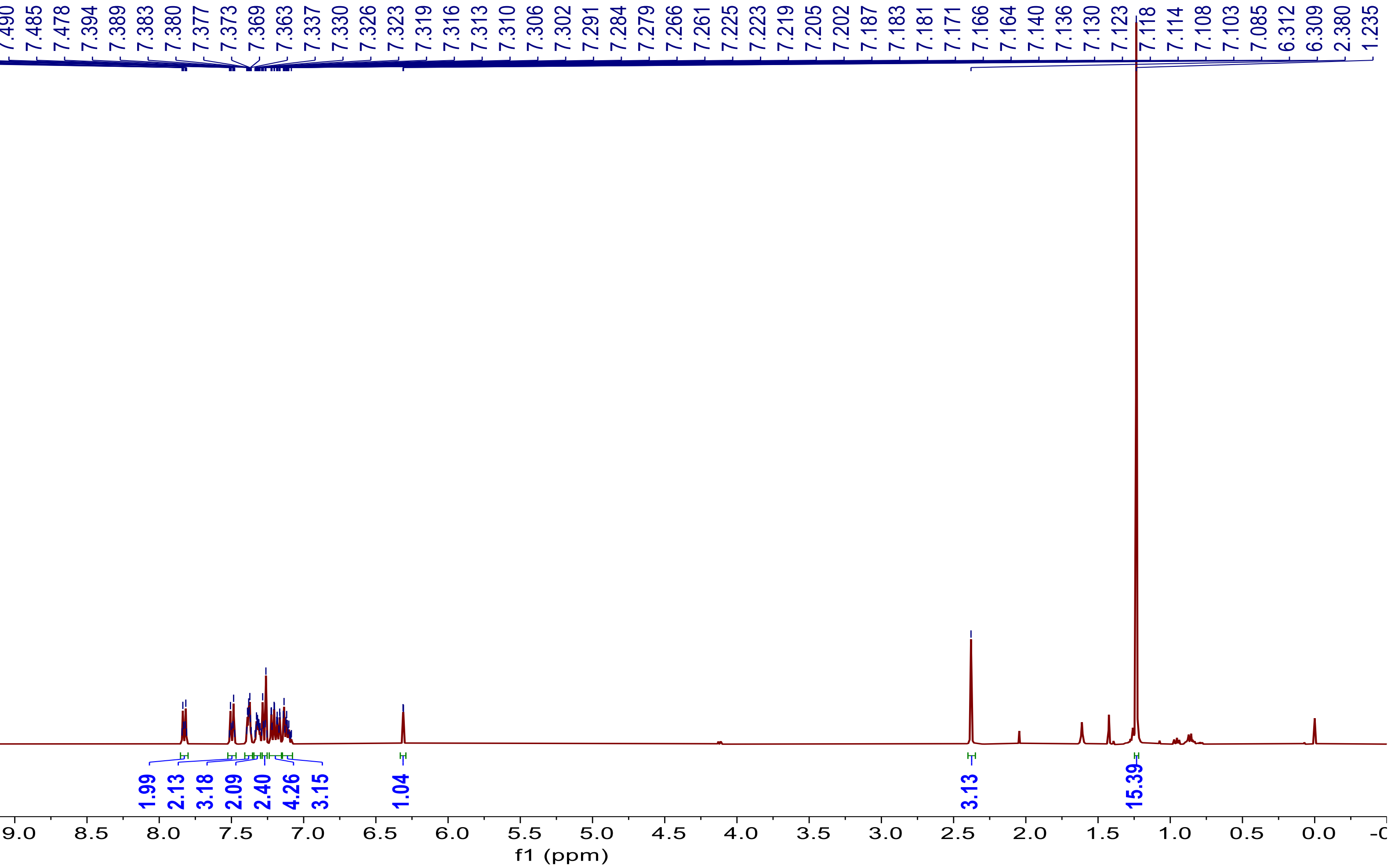

^{13}C NMR Spectrum of 11 at 25 ºC ($CDCl_3$)

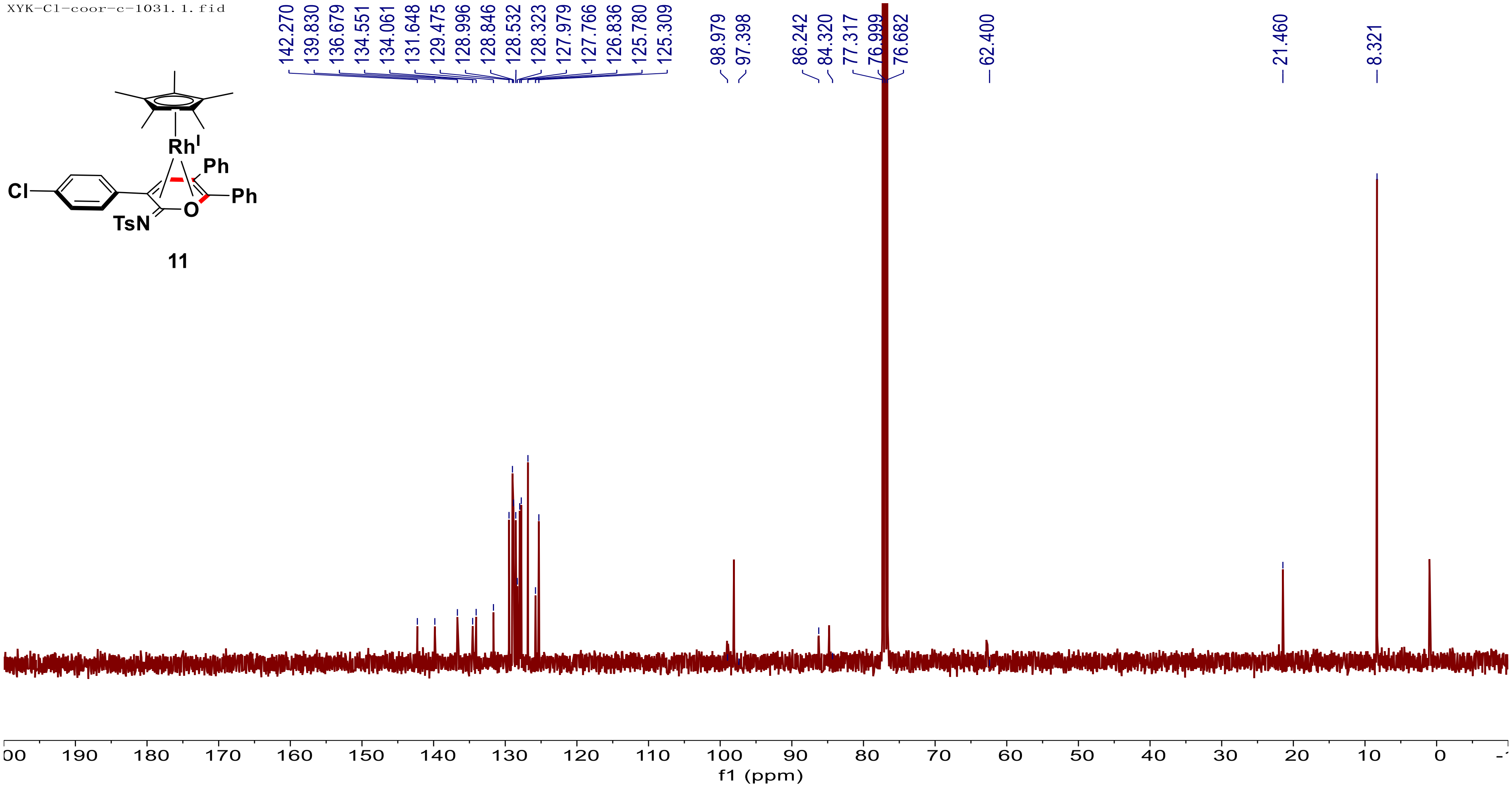

^{1}H NMR Spectrum of 12 at 25 °C ($CDCl_3$)

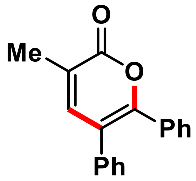

3aa

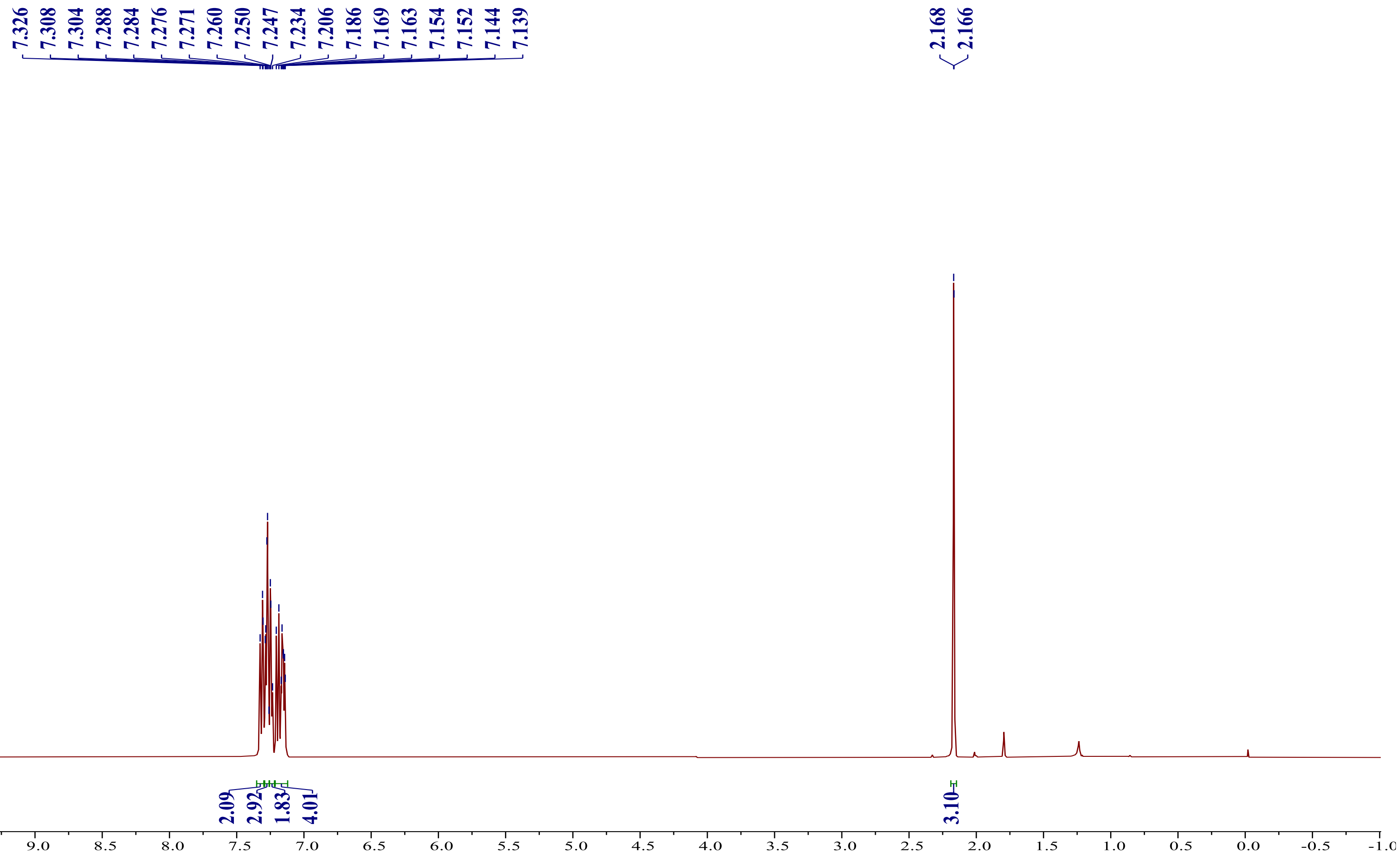

^{13}C NMR Spectrum of 3aa at 25 ºC ($CDCl_3$)

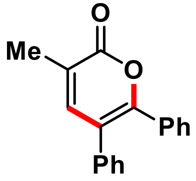

3aa

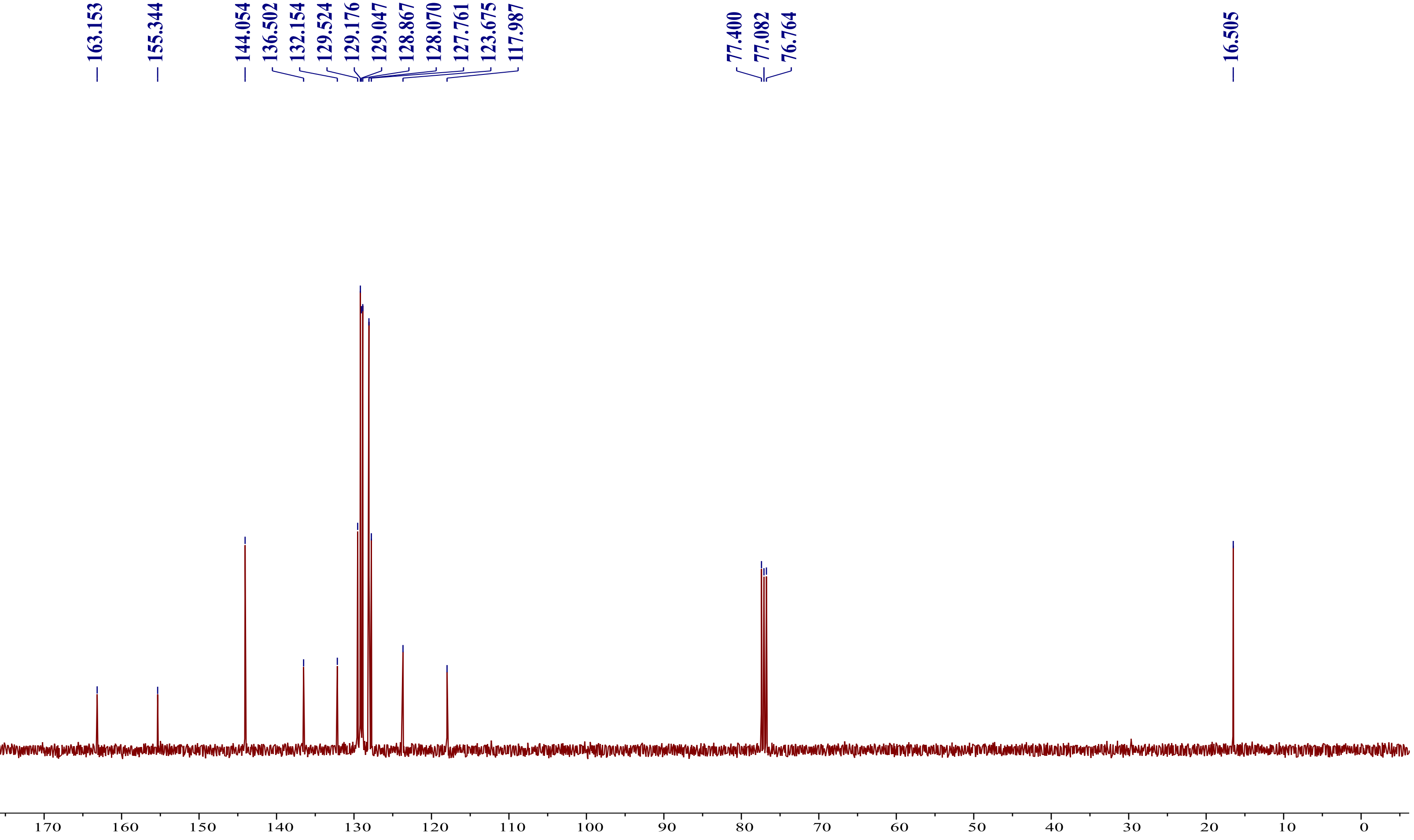

21. Supplementary References

1. Zhang, X., Yu, X., Ji, D., Yamamoto, Y., Almansour, A. I., Arumugam, N., Kumar, R. S. & Bao, M. Rhodium-Catalyzed Oxidative Benzannulation of N-Adamantyl-1-naphthylamines with Internal Alkynes via Dual C–H Bond Activation: Synthesis of Substituted Anthracenes. *Org. Lett.* **18**, 4246-4249 (2016).
2. Mochida, S., Hirano, K., Satoh, T. & Miura, M. Synthesis of functionalized α-pyrone and butenolide derivatives by rhodium-catalyzed oxidative coupling of substituted acrylic acids with alkynes and alkenes. *J. Org. Chem.* **74**, 6295-6298 (2009).
3. Sashuk, V., Ignatowska, J. & Grela, K. A fine-tuned molybdenum hexacarbonyl/phenol initiator for alkyne metathesis. *J. Org. Chem.* **69**, 7748-7751 (2004).
4. Archambeau, A. & Rovis, T. Rhodium (III)-Catalyzed Allylic C(sp^3)-H Activation of Alkenyl Sulfonamides: Unexpected Formation of Azabicycles. *Angew. Chem. Int. Ed.* **54**, 13337-13340 (2015).
5. Itoh, M., Shimizu, M., Hirano, K., Satoh, T. & Miura, M. Rhodium-catalyzed decarboxylative and dehydrogenative coupling of maleic acids with alkynes and alkenes. *J. Org. Chem.* **78**, 11427-11432 (2013).
6. Xu, H.-J., Kang, Y.-S., Shi, H., Zhang, P., Chen, Y.-K., Zhang, B., Liu, Z.-Q., Zhao, J., Sun, W.-Y., Yu, J.-Q. & Lu, Y. Rh (III)-Catalyzed

meta-C–H Alkenylation with Alkynes. *J. Am. Chem. Soc.* **141**, 76-79 (2018).

7. Matsuda, T. & Suzuki, K. Rhodium (III)-catalysed decarbonylative coupling of maleic anhydrides with alkynes. *RSC Adv.* **4**, 37138-37141 (2014).

8. Kandasamy, M., Huang, Y.-H., Ganesan, B., Senadi, G. C. & Lin, W.-Y. In situ generation of alkynylzinc and its subsequent cross-coupling reaction in a flow reactor. *Eur. J. Org. Chem.* 4349-4356 (2019).

9. Nan, J., Zuo, Z., Luo, L., Bai, L., Zheng, H., Yuan, Y., Liu, J., Luan, X. & Wang, Y. Ru^{II}-Catalyzed Vinylative Dearomatization of Naphthols via a $C(sp^2)$−H Bond Activation Approach. *J. Am. Chem. Soc.* **135**, 17306−17309 (2013).

10. Song, S.-J., Lu, P., Liu, H., Cai, S.-H., Feng, C. & Loh, T.-P. Switchable C–H Functionalization of N-Tosyl Acrylamides with Acryloylsilanes. *Org. Lett.* **19**, 2869-2872 (**2017**).

11. Jiang, B., Zhao, M., Li, S.-S., Xu, Y.-H. & Loh, T.-P. Macrolide Synthesis through Intramolecular Oxidative Cross-Coupling of Alkenes. *Angew. Chem. Int. Ed.* **57**, 555-559 (2018).

12. Wu, H. M., Yang, B., Zhu, L., Lu, R. H., Li, G. G. & Lu, H. J. High-Valent Palladium-Promoted Formal Wagner–Meerwein Rearrangement. *Org. Lett.* **18**, 5804-5807 (2016).

13. Youn, S. W., Ko, T. Y., Kim, H. Y. & Kim, Y. A.

Pd(II)/Cu(II)-Catalyzed Regio- and Stereoselective Synthesis of *(E)*-3-Arylmethyleneisoindolin-1-ones Using Air as the Terminal Oxidant. *Org. Lett.* **20**, 7869-7874 (2018).

14. Zhang, J. & Loh, T.-P. Ruthenium- and rhodium-catalyzed cross-coupling reaction of acrylamides with alkenes: efficient access to *(Z,E)*-dienamides. *Chem. Commun.* **48**, 11232 – 11234 (2012).

15. Raghunathan, R., Kumarasamy, E., Lyer, A., Ugrinov, A. & Sivaguru, J. Intramolecular Paternò–Büchi reaction of atropisomeric α-oxoamides in solution and in the solid-state. *Chem. Commun.* **49**, 8713 – 8715 (2013).

16. Ackermann, L., Lygin, A. V. & Hofmann, N. Ruthenium-Catalyzed Oxidative Synthesis of 2-Pyridones through C–H/N–H Bond Functionalizations. *Org. Lett.* **13**, 3278-3281 (2011).

17. Wu, T., Mu, X. & Liu, G.-S. Palladium‐Catalyzed Oxidative Arylalkylation of Activated Alkenes: Dual C–H Bond Cleavage of an Arene and Acetonitrile. *Angew. Chem. Int. Ed.* **50**, 12578-12581 (2011).

18. Li, X. H., Sun, B., Zhou, J. D., Jin, C. & Yu, C. M. Regioselective Acetoxylation of Terminal Olefins Using a Palladium(II)–Thiadiazole Catalyst. *Eur. J. Org. Chem.* 2635-2638 (2019).

19. BAYER, DE 2002065, 1971. [chem, Abstr. 73,76735]

20. Liu, C., Fang, Y., Wang, S.-Y. & Ji, S.-J. Highly Regioselective

Rh^{III}-Catalyzed Thiolation of N-Tosyl Acrylamides: General Access to *(Z)*-β-Alkenyl Sulfides. *Org. Lett.* **20**, 6112-6116 (2018).

21. Xie, W. J., Yang, J., Wang, B. Q. & Li, B. Regioselective Ortho Olefination of Aryl Sulfonamide via Rhodium-Catalyzed Direct C–H Bond Activation. *J. Org. Chem.* **17**, 8278-8287 (2014).
22. Li, Y.-M., Sun, M., Wang, H.-L., Tian, Q.-P. & Yang, S.-D. Direct Annulations toward Phosphorylated Oxindoles: Silver - Catalyzed Carbon - Phosphorus Functionalization of Alkenes. *Angew. Chem. Int. Ed.* **52**, 3972-3976 (2013).
23. Li, Y., Alper, H. & Yu, Z. K. Palladium-Catalyzed Regiospecific Aminocarbonylation of Alkynes in the Ionic Liquid [bmim][Tf_2N^-]. *Org. Lett.* **8**, 5199-5201 (2006).
24. Su, Y., Zhao, M., Han, K. L., Song, G. Y. & Li, X. W. Synthesis of 2-Pyridones and Iminoesters via Rh(III)-Catalyzed Oxidative Coupling between Acrylamides and Alkynes. *Org. Lett.* **12**, 5462-5465 (2010).
25. Boyer, A. Rhodium(II)-Catalyzed Stereocontrolled Synthesis of Dihydrofuran-3-imines from 1-Tosyl-1,2,3-triazoles. *Org. Lett.* **16**, 1660-1663 (2014).
26. Imanieh, H., Macleod, D., Quayle, P. & D. M. Gareth. The use of β-(tri-n-butylstannyl)acrylamides as β-lithioacrylamide synthons. *Tetrahedron. Lett.* **30**, 2693-2696 (1989).

27. Frisch M. J., Trucks G. W., Schlegel H. B., Scuseria G. E., Robb M. A., Cheeseman J. R., Scalmani G., Barone V., Mennucci B., Petersson G. A., Nakatsuji H., Caricato M., Li X., Hratchian H. P., Izmaylov A. F., Bloino J., Zheng G., Sonnenberg J. L., Hada M.,Ehara M., Toyota K., Fukuda R., Hasegawa J., Ishida M., Nakajima T., Honda Y., Kitao O., Nakai H., Vreven T., Montgomery J. A. Jr., Peralta J. E., Ogliaro F., Bearpark M., Heyd J. J., Brothers E., Kudin K. N., Staroverov V. N., Kobayashi R., Normand J., Raghavachari K., Rendell A., Burant J. C., Iyengar S. S., Tomasi J., Cossi M., Rega N., Millam J. M., Klene M., Knox J. E., Cross J. B., Bakken V., Adamo C., Jaramillo J., Gomperts R., Stratmann R. E., Yazyev O., Austin A. J., Cammi R., Pomelli C., Ochterski J. W., Martin R. L., Morokuma K., Zakrzewski V. G., Voth G. A., Salvador P., Dannenberg J. J., Dapprich S., Daniels A. D., Farkas O., Foresman J. B., Ortiz J. V., Cioslowski J. & Fox D. J., Gaussian 09 revision D.01, Gaussian Inc.: Wallingford CT 2013.
28. Becke, A. D. Density‐functional thermochemistry. III. The role of exact exchange. *J. Chem. Phys*. **98**, 5648-5652 (1993).
29. Lee, C., Yang, W. & Parr, R. G. Development of the Colle-Salvetti correlation-energy formula into a functional of the electron density. *Phys. Rev. B: Condens. Matter Mater. Phys*. **37**, 785-789 (1988).
30. Grimme, S., Ehrlich, S. & Goerigk, L. Effect of the damping function in dispersion corrected density functional theory. *J. Comp. Chem*. **32**,

1456-1465 (2011).

31. Hay, P. J. & Wadt, W. R. Ab initio effective core potentials for molecular calculations. Potentials for the transition metal atoms Sc to Hg. *J. Chem. Phys*. **82**, 270-283 (1985).

32. Wadt, W. R. & Hay, P. J. Ab initio effective core potentials for molecular calculations. Potentials for K to Au including the outermost core orbitals. *J. Chem. Phys*. **82**, 284-298 (1985).

33. Hay, P. J. & Wadt, W. R. Ab initio effective core potentials for molecular calculations. Potentials for K to Au including the outermost core orbitals. *J. Chem. Phys.* **82**, 299-310 (1985).

34. Weigend, F. & Ahlrichs, R. Balanced basis sets of split valence, triple zeta valence and quadruple zeta valence quality for H to Rn: Design and assessment of accuracy. *Phys. Chem. Chem. Phys.* **7**, 3297-3305 (2005).

35. Weigend, F. Accurate Coulomb-fitting basis sets for H to Rn. *Phys. Chem. Chem. Phys.* **8**, 1057-1065 (2006).

36. Marenich, A. V., Cramer, C. J. & Truhlar D. G. Universal Solvation Model Based on Solute Electron Density and on a Continuum Model of the Solvent Defined by the Bulk Dielectric Constant and Atomic Surface Tensions. *J. Phys. Chem. B.* **113**, 6378-6396 (2009).

37. Bickelhaupt, F. M. & Houk, K. N. Analyzing Reaction Rates with the Distortion/Interaction-Activation Strain Model. *Angew. Chem., Int. Ed*.

56, 10070-10086 (2017).

38. Fernandez, I. & and Bickelhaupt, F. M. The activation strain model and molecular orbital theory: understanding and designing chemical reactions. *Chem. Soc. Rev.* **43**, 4953-4967 (2014).
