# Supplementary material for: Divergent rhodium-catalyzed electrochemical vinylic C–H annulation of acrylamides with alkynes
Source: Nat Commun. 2021 Feb 10;12:930. doi: 10.1038/s41467-021-21190-8 (PMC7876044; doi:10.1038/s41467-021-21190-8)
Supplement: Supplementary file 3 — Description of Additional Supplementary Files. [file 41467_2021_21190_MOESM3_ESM.pdf]

Description of Additional Supplementary Files

File Name: Supplementary Data 1
Description: Cartesian Coordinates
