# Supplementary material for: Divergent rhodium-catalyzed electrochemical vinylic C–H annulation of acrylamides with alkynes
Source: Nat Commun. 2021 Feb 10;12:930. doi: 10.1038/s41467-021-21190-8 (PMC7876044; doi:10.1038/s41467-021-21190-8)
Supplement: Supplementary file 4 — Supplementary Data 1. [file 41467_2021_21190_MOESM4_ESM.zip › 280688_2_data_set_5228410_qmq2gz.docx]

**Cartesian coordinates of the structures**

**Cp^*^Rh(OAc)_2_**

Rh 0.01556300 0.18542300 -0.09101500

C 1.97102400 0.38429500 0.93656300

C 2.17092400 0.51376200 -0.46191500

C 1.39307100 -0.93328100 1.18613500

C 1.69167800 -0.70502700 -1.10320600

C 1.27706500 -1.61549100 -0.06764200

C -1.51911900 2.20635800 -0.02618000

O -0.82026600 1.94793000 -1.05715800

O -1.33430800 1.50903800 1.02540900

C -2.54774200 -1.44211100 -0.22260500

O -3.40404900 -2.07218200 -0.82656400

O -1.60958000 -0.77448800 -0.86940600

C -2.57668300 3.27385500 -0.06278300

H -3.51841200 2.81644100 -0.38806900

H -2.72586100 3.69987500 0.93203100

H -2.30878300 4.05300300 -0.77994900

C -2.52014500 -1.40430700 1.30203000

H -1.59782600 -1.86134400 1.67736500

H -2.53786700 -0.37127800 1.65932200

H -3.37609200 -1.95770400 1.69269000

C 2.27810200 1.38960100 2.00067000

H 1.48171500 1.42067100 2.74973900

H 3.21434000 1.13695300 2.51517100

H 2.38118100 2.39385800 1.58356800

C 1.08997900 -1.47225100 2.54739700

H 2.02232700 -1.68940100 3.08478100

H 0.52309000 -0.74868500 3.14075700

H 0.50837600 -2.39461200 2.49695800

C 0.72628500 -2.98395100 -0.30903900

H 1.52110400 -3.65968800 -0.64747900

H 0.28451100 -3.40513700 0.59634900

H -0.05259700 -2.95304100 -1.07661300

C 2.69728200 1.70331800 -1.19800800

H 2.78658700 2.57365700 -0.54450000

H 3.68868000 1.48567600 -1.61473300

H 2.03404800 1.97189100 -2.02577500

C 1.72229400 -0.99937200 -2.56836800

H 0.89347600 -1.65379700 -2.84967400

H 1.63717300 -0.08140100 -3.15580000

H 2.66204800 -1.49472900 -2.84700500

**1a**

C -2.64581500 0.06301100 0.11843500

O -3.24084600 -0.97057300 0.35279800

N -1.24881400 0.12670000 0.02559400

H -0.85617600 0.86463200 -0.54981000

C -3.35135300 1.37552700 -0.07321200

C -2.75227500 2.54046800 0.20312900

H -3.28176000 3.48309100 0.09665400

H -1.73621200 2.59548900 0.58245800

C -4.78387500 1.25080600 -0.51238000

H -4.85308000 0.76399300 -1.49231300

H -5.33513500 0.61688400 0.18918800

H -5.26449400 2.23086900 -0.56988400

S -0.28499100 -1.30879900 0.04448000

O -0.47647400 -1.94827700 1.34020200

O -0.46437300 -2.03787100 -1.21079700

C 1.31161700 -0.50856900 0.00532400

C 1.85018700 -0.01075600 1.19319600

C 1.99279200 -0.39643800 -1.20545400

C 3.08913200 0.61994600 1.15548500

H 1.30549600 -0.12770300 2.12388300

C 3.23313600 0.23876300 -1.22204400

H 1.55777300 -0.81103500 -2.10840900

C 3.79798400 0.75353200 -0.04831100

H 3.51840500 1.01158400 2.07385700

H 3.77388100 0.33083900 -2.16003900

C 5.15517200 1.41038900 -0.06827700

H 5.93674000 0.69701100 0.22311100

H 5.40567000 1.78264800 -1.06613000

H 5.20378700 2.24927500 0.63362300

**1b**

C -1.45042900 0.36903400 -0.05324900

O -1.33576200 1.58610100 -0.15038400

N -0.39262100 -0.50299600 0.06180200

H -0.63881800 -1.46382300 0.25379300

C -2.80841800 -0.28328700 -0.04150600

C -3.02351100 -1.47559500 -0.61095800

H -4.01608700 -1.91685800 -0.63618500

H -2.23589200 -2.03535200 -1.10817400

C -3.89759500 0.55863300 0.56281100

H -3.70537300 0.74524900 1.62656000

H -3.92130200 1.53737400 0.07371100

H -4.87398300 0.07750200 0.46139700

C 0.98838200 -0.22750200 0.03992400

C 1.51429200 1.05230200 -0.19010900

C 1.85952200 -1.30690200 0.25254500

C 2.89750500 1.22729700 -0.20328400

H 0.84099900 1.88218700 -0.34714500

C 3.23704700 -1.11447000 0.23637900

H 1.45138300 -2.29998900 0.42989500

C 3.76635800 0.15658200 0.00755000

H 3.29694700 2.22186600 -0.38148500

H 3.89632500 -1.96167200 0.40282700

H 4.84132500 0.30912600 -0.00536000

**2a**

C -0.60777000 -0.00003100 -0.00009700

C 0.60777000 0.00001200 -0.00008800

C 2.03053600 0.00000500 -0.00003200

C 2.74619500 -1.21316600 0.00012700

C 2.74620200 1.21317200 -0.00015100

C 4.13750700 -1.20829900 0.00016200

H 2.19673100 -2.14906600 0.00021100

C 4.13751400 1.20829700 -0.00009300

H 2.19674400 2.14907400 -0.00027600

C 4.83815800 -0.00000400 0.00005900

H 4.67802400 -2.15061300 0.00028800

H 4.67803600 2.15060700 -0.00018500

H 5.92432400 -0.00000700 0.00009600

C -2.03053600 -0.00001500 -0.00004400

C -2.74621300 -1.21317600 -0.00015300

C -2.74618400 1.21316200 0.00012500

C -4.13752500 -1.20828800 -0.00009100

H -2.19676300 -2.14908300 -0.00027600

C -4.13749600 1.20830800 0.00016400

H -2.19671200 2.14905700 0.00021000

C -4.83815800 0.00001800 0.00006200

H -4.67805500 -2.15059400 -0.00018000

H -4.67800500 2.15062700 0.00029300

H -5.92432400 0.00003100 0.00010200

**int1**

Rh 0.39647200 1.13578700 -0.55679700

C 1.51726800 2.99839200 -0.01037600

C 0.29350900 2.93380100 0.73727300

C 1.16693400 3.04573000 -1.39771500

C -0.81248800 3.09052900 -0.20774300

C -0.28235300 3.18432600 -1.50538300

C -0.97445400 -1.19437900 -1.19857400

O -1.51385100 -2.26720000 -1.42447800

C -0.08583100 -0.48325500 -2.20860000

C 1.28261300 -0.43430400 -2.03022700

H 1.87317800 0.00493400 -2.83569400

C -0.75569100 -0.07098600 -3.48943100

H -1.08885200 -0.97563900 -4.01244900

H -1.64505600 0.53979200 -3.30353300

H -0.07332100 0.47995400 -4.14407800

N -1.05771800 -0.36004800 -0.12227300

C 1.75736500 -0.21772200 0.19737900

C 2.01058900 -1.01496800 -0.85390200

C 2.36144800 -0.16721500 1.52478300

C 1.59163100 -0.02723800 2.69199900

C 3.76569100 -0.20690900 1.64413900

C 2.20924000 0.04567400 3.93983800

H 0.51342300 0.02521700 2.61867600

C 4.37765700 -0.12354600 2.89148800

H 4.36534200 -0.30888900 0.74505700

C 3.60076500 0.00136100 4.04674100

H 1.59624200 0.14006600 4.83182800

H 5.46156400 -0.15456600 2.96276100

H 4.07735400 0.06549000 5.02094300

C 2.80279900 -2.24933400 -0.97572200

C 3.06096000 -3.06682300 0.13987000

C 3.29046700 -2.65734400 -2.22865700

C 3.80489000 -4.23451700 0.00514000

H 2.65643100 -2.78929800 1.10628100

C 4.03597100 -3.82781900 -2.36098200

H 3.08505000 -2.05362900 -3.10844800

C 4.30172000 -4.61953100 -1.24365300

H 3.98682900 -4.85563000 0.87785600

H 4.40619300 -4.12217800 -3.33938200

H 4.87932800 -5.53391800 -1.34527400

C 2.12679800 3.14569000 -2.54351000

H 1.71548300 2.69461600 -3.45141800

H 2.35051300 4.19666100 -2.77127700

H 3.07183700 2.64559300 -2.31564000

C 2.89915800 3.04005800 0.55995300

H 3.19273000 4.07893100 0.76100200

H 2.96520200 2.48198500 1.49551700

H 3.63128800 2.61262100 -0.13030200

C -1.03849000 3.40034600 -2.77834100

H -1.04657500 4.46392700 -3.05276300

H -0.59177000 2.85420400 -3.61403200

H -2.07914100 3.07703300 -2.68588200

C -2.25167200 3.11677900 0.19463500

H -2.53173400 4.11262000 0.56343800

H -2.90907800 2.86818400 -0.64265100

H -2.42608700 2.39466300 0.99528200

C 0.16018600 2.99264300 2.22522400

H 1.05564600 2.60885400 2.71774300

H 0.01236200 4.03222900 2.54985000

H -0.68739900 2.39557400 2.56482100

S -1.79299900 -0.81071500 1.31027700

O -1.21588400 -2.05233100 1.83390200

O -1.76535800 0.40204300 2.15181600

C -3.50227200 -1.11387700 0.87722000

C -3.87800500 -2.36577800 0.38712500

C -4.43885800 -0.09641000 1.05306800

C -5.21272900 -2.58496600 0.06001300

H -3.13023300 -3.13562200 0.24989900

C -5.77054400 -0.33563000 0.71899700

H -4.12865900 0.85753500 1.46273200

C -6.17667300 -1.57882700 0.21845300

H -5.51277500 -3.55553400 -0.32682800

H -6.50671500 0.45244400 0.85579500

C -7.62322300 -1.84389200 -0.11909500

H -7.71770900 -2.40548800 -1.05501900

H -8.10736500 -2.43962000 0.66562900

H -8.18826000 -0.91221200 -0.22158200

**TS2**

Rh -1.46331800 -0.69935800 -0.44148500

C -2.99822500 -1.46896300 1.10173400

C -2.66393300 -2.57573300 0.22423700

C -3.57765400 -0.43634000 0.31256700

C -3.04385200 -2.22866200 -1.09254400

C -3.54236100 -0.86372100 -1.07123600

C 0.77354700 -1.21529700 -1.04599900

O 1.38085700 -2.27642600 -1.15267000

C -0.08258800 -0.59745400 -2.12436000

C 0.27421200 0.80320200 -2.36161200

H 0.76944500 1.07545200 -3.29422000

C -0.22556400 -1.46819800 -3.35045900

H 0.67451300 -1.40572000 -3.97886100

H -0.35245000 -2.51623400 -3.07728900

H -1.07935600 -1.14920700 -3.95860400

N 0.63365300 -0.47493700 0.16063300

C -0.51922600 1.07841700 -0.18464300

C 0.18476500 1.66931500 -1.31806700

C -0.95299900 1.90514700 0.95065700

C -1.07260600 1.45570300 2.27623900

C -1.45463200 3.18788500 0.63875300

C -1.62767600 2.27097600 3.25763800

H -0.69554000 0.48364000 2.54711400

C -2.04723100 3.98292700 1.61704700

H -1.39027400 3.55602800 -0.37804700

C -2.12483700 3.53649700 2.93593400

H -1.67615600 1.90868200 4.28065100

H -2.43328600 4.96124400 1.34527900

H -2.56626900 4.16531500 3.70372300

C 0.93926800 2.94116700 -1.25573300

C 1.68785200 3.26572500 -0.11103300

C 0.95742400 3.82449100 -2.34527100

C 2.43608300 4.44000700 -0.06586100

H 1.67783200 2.59389600 0.74200100

C 1.70748000 4.99889800 -2.29716600

H 0.36722800 3.59143700 -3.22772400

C 2.44966100 5.31202000 -1.15701500

H 3.01075600 4.67432400 0.82598500

H 1.70586000 5.67359800 -3.14901000

H 3.03052000 6.22908200 -1.11770100

C -2.02327400 -3.85057200 0.67991400

H -2.73898700 -4.47233000 1.23468700

H -1.65526600 -4.43569500 -0.16674000

H -1.17153400 -3.64351500 1.33418600

C -2.81293500 -1.49515900 2.58743100

H -3.54103900 -2.17249500 3.05415100

H -1.81257500 -1.85395800 2.85040000

H -2.94902200 -0.50536000 3.02724100

C -4.14907200 0.85641900 0.79958900

H -5.23772200 0.77225600 0.91742700

H -3.72445600 1.14892600 1.76114100

H -3.95123600 1.66942900 0.09604300

C -4.14695700 -0.13325100 -2.23009900

H -5.20882400 -0.38840500 -2.35405600

H -4.07749100 0.94904700 -2.08992700

H -3.63329400 -0.38093300 -3.16354600

C -2.99678900 -3.11517900 -2.29653700

H -2.19246700 -3.85227500 -2.22704200

H -3.94325400 -3.66365300 -2.39756900

H -2.85016800 -2.54361000 -3.21563200

S 1.42189600 -1.06546100 1.54743800

O 1.46130500 0.05740400 2.49274700

O 0.81735000 -2.33635500 1.97153900

C 3.09043200 -1.33836300 0.98343300

C 3.57374700 -2.63601600 0.83938100

C 3.88434000 -0.22633100 0.70442200

C 4.88810000 -2.81668800 0.42021800

H 2.91916300 -3.47566700 1.03735200

C 5.19154000 -0.42859800 0.27001300

H 3.48484200 0.77550900 0.81787400

C 5.71324400 -1.72160900 0.12738400

H 5.27701800 -3.82469700 0.30251200

H 5.81712700 0.42976000 0.04000400

C 7.14056100 -1.93253500 -0.31301600

H 7.23956200 -2.83399800 -0.92667600

H 7.51033600 -1.08127100 -0.89325500

H 7.80473500 -2.05330200 0.55295400

**int3**

Rh -1.62552600 -0.86122100 -0.10256700

C -3.03112600 -0.67421300 1.70305800

C -1.89478200 -1.49412000 2.05660000

C -3.76377500 -1.34949800 0.67504400

C -1.90272400 -2.65032600 1.21641800

C -3.05668000 -2.54840600 0.34773800

C 0.74201000 -1.37892300 -1.63344800

O 1.50342300 -2.28532000 -1.92659800

C -0.67087800 -1.30780700 -2.02833600

C -1.32522100 -0.02742900 -2.05712500

H -2.12961600 0.17078600 -2.75824900

C -1.15949700 -2.42726600 -2.90994300

H -0.66326200 -2.39045600 -3.88764800

H -0.92095200 -3.39901100 -2.46968400

H -2.24059200 -2.36379700 -3.06132500

N 1.17009000 -0.25180000 -0.85178300

S 2.42087000 0.70936800 -1.59434200

O 2.50754000 0.34385000 -3.00909100

O 2.15862100 2.09689300 -1.19505700

C 3.89497000 0.16155200 -0.75381800

C 4.32099500 -1.15971300 -0.90709800

C 4.58610400 1.06634700 0.04786400

C 5.46893500 -1.56799000 -0.23649900

H 3.73707100 -1.84907100 -1.50749100

C 5.73730900 0.63520600 0.70619400

H 4.21537900 2.07860100 0.15865000

C 6.19587800 -0.68055400 0.57301600

H 5.80775700 -2.59575600 -0.33924000

H 6.28447100 1.33163800 1.33601800

C 7.45257700 -1.13878800 1.27057700

H 7.31981900 -2.12782900 1.72295700

H 8.28747200 -1.21696900 0.56233800

H 7.75163200 -0.44036200 2.05805300

C 0.09140000 0.47943200 -0.20379400

C -0.96602500 0.95638900 -1.08478900

C 0.45414300 1.05842800 1.10948000

C 1.60724000 0.60014300 1.77106200

C -0.34119000 2.01343300 1.76840800

C 1.97087900 1.09938200 3.01958400

H 2.21489500 -0.16187700 1.30098000

C 0.02588500 2.51272700 3.01610200

H -1.25487100 2.36417300 1.31103500

C 1.18802200 2.06662800 3.65007700

H 2.87325600 0.72728500 3.49708100

H -0.60397900 3.25855900 3.49387400

H 1.47326200 2.46301900 4.62027000

C -1.63547600 2.27672700 -1.06581700

C -3.03359300 2.37796600 -1.07369400

C -0.86193300 3.44692700 -1.08108900

C -3.65291300 3.62711400 -1.08639400

H -3.62645000 1.46857700 -1.05350000

C -1.48512300 4.69383600 -1.09438300

H 0.21942200 3.36201700 -1.08103900

C -2.87873500 4.78936600 -1.09352900

H -4.73755100 3.69314400 -1.08525300

H -0.87860200 5.59505000 -1.10597800

H -3.35906400 5.76377400 -1.09852200

C -0.92523700 -1.22160800 3.16139900

H -1.26608500 -1.69374000 4.09281300

H 0.06688000 -1.61593400 2.92869700

H -0.81314000 -0.15248300 3.34665000

C -0.94651800 -3.80248500 1.27269400

H 0.01607000 -3.49646900 1.69111300

H -1.33936600 -4.61647900 1.89769800

H -0.75446900 -4.21169900 0.27637000

C -3.45524300 0.59648700 2.37422100

H -4.28748100 0.41264400 3.06704500

H -2.63627100 1.03632200 2.94569300

H -3.78944100 1.34340300 1.64583100

C -5.06383500 -0.89659800 0.08497600

H -5.91478000 -1.29176700 0.65722000

H -5.14810600 0.19422600 0.09361500

H -5.18007900 -1.23682000 -0.94842300

C -3.51089600 -3.60230300 -0.61306400

H -2.66615900 -4.15723300 -1.02773800

H -4.16509400 -4.32669400 -0.10892200

H -4.07421300 -3.17487800 -1.44769200

**int4**

Rh -0.53625300 -1.23833900 -0.42067800

C -0.74883600 -2.72004000 -2.08946400

C -2.05571700 -2.73363000 -1.41393200

C 0.23553500 -3.19239900 -1.18160900

C -1.85131300 -3.11976200 -0.08903100

C -0.41215900 -3.34599700 0.10046700

C -0.38808300 -0.08219200 2.51389700

O -0.55158600 0.80983600 3.34500200

C 0.63783200 -1.14016800 2.79953500

C 1.74967400 -1.30775300 2.06831100

H 2.51388500 -1.97911400 2.46391900

C 0.39892300 -1.85666800 4.10312500

H 0.34588500 -1.13159900 4.92242000

H -0.56142800 -2.39001100 4.09628800

H 1.19519800 -2.57812100 4.31032900

N -1.13539000 -0.22166500 1.35824200

C 1.29242700 -0.37249900 -0.20487400

C 2.11999300 -0.56468300 0.85046800

C 1.64501500 0.56657100 -1.28998100

C 1.82395300 1.92999900 -0.99508300

C 1.80774900 0.15246900 -2.62195300

C 2.15717000 2.84256700 -1.99276000

H 1.70319100 2.26059100 0.03057500

C 2.15817200 1.06047600 -3.61939100

H 1.67242500 -0.89416600 -2.86586700

C 2.32872200 2.41307800 -3.31063300

H 2.28712600 3.89153000 -1.73999400

H 2.29594500 0.71360700 -4.64026900

H 2.59487900 3.12278100 -4.08876300

C 3.51172600 -0.01750700 0.89712300

C 4.40561200 -0.15803900 -0.17555300

C 3.96187400 0.63058300 2.05855000

C 5.69910400 0.35356100 -0.09910400

H 4.07652800 -0.66672100 -1.07535300

C 5.25524300 1.14622600 2.13550400

H 3.28042700 0.73896800 2.89781500

C 6.12948600 1.01074900 1.05582000

H 6.37499700 0.23474800 -0.94182900

H 5.57967700 1.65453300 3.03968800

H 7.13849600 1.40940900 1.11484100

C -3.33485000 -2.32816400 -2.07110200

H -3.69986700 -3.11943000 -2.73948500

H -4.10862600 -2.11214000 -1.33274100

H -3.18640600 -1.42650600 -2.67347200

C -2.86399600 -3.21893300 1.00610600

H -3.79004000 -2.70606400 0.74088100

H -3.09524600 -4.26887600 1.22876400

H -2.48675500 -2.75542800 1.92231100

C -0.58784600 -2.39389700 -3.54052500

H 0.45056200 -2.48236500 -3.86692600

H -1.19099900 -3.07763300 -4.15124100

H -0.92887800 -1.37406300 -3.74464500

C 1.67703100 -3.49508400 -1.44813400

H 2.32043800 -3.01689400 -0.70389600

H 1.85298700 -4.57771300 -1.40930900

H 1.98848600 -3.14153400 -2.43390400

C 0.18108100 -4.04388200 1.28057700

H -0.29013800 -3.73160700 2.21285800

H 0.03020800 -5.12811500 1.17477700

H 1.25230300 -3.85679200 1.36362000

C -1.21954100 1.66902200 -2.31200800

H -2.11054800 2.08251800 -2.79832300

H -0.41987800 1.56468500 -3.04238900

H -0.89535700 2.34082800 -1.51629700

O -1.51958100 0.35897500 -1.81331800

H -2.33026800 0.39704500 -1.24810700

S -2.58810800 0.61192000 1.32194300

O -3.30851100 0.10038600 0.11554600

O -3.32032700 0.51744700 2.58442500

C -2.21799200 2.31984500 0.90138200

C -0.99297200 2.91303300 1.20753000

C -3.19634700 3.02452900 0.19808200

C -0.74711900 4.21502600 0.77963600

H -0.25413300 2.37098800 1.78104200

C -2.93567700 4.33007300 -0.21272900

H -4.13905200 2.54609700 -0.04354900

C -1.70599400 4.94153900 0.06015100

H 0.21042500 4.67556500 1.00988300

H -3.69552200 4.87840600 -0.76338200

C -1.40608000 6.33981600 -0.41981000

H -0.67401600 6.32608100 -1.23746600

H -2.30715900 6.83891200 -0.78859100

H -0.98164900 6.95343200 0.38274200

**TS5**

Rh -1.95919100 0.07331500 0.06994800

C -3.58515400 1.14928100 0.97223300

C -4.09230900 -0.23757600 1.00288300

C -3.52061600 1.56914800 -0.40506000

C -4.17992800 -0.68950300 -0.30902100

C -3.76352700 0.41105600 -1.19762000

C 0.39595700 -2.37976100 -1.15325400

O 1.30192600 -2.95231300 -1.78066400

C -0.67259600 -1.73728700 -2.00806400

C -0.69594700 -0.40369300 -2.24351000

H -1.34475700 -0.04436700 -3.04222500

C -1.49067000 -2.71614700 -2.80293900

H -0.80964200 -3.35535700 -3.37548300

H -2.06182700 -3.37870100 -2.14121400

H -2.17599100 -2.21841100 -3.49647500

N 0.20251700 -2.22249300 0.16605000

C -0.27568000 1.11676600 -0.43951800

C 0.18155400 0.63337100 -1.60864800

C 0.41406100 2.03871900 0.46859800

C 1.72479300 1.74676700 0.89189700

C -0.19653200 3.20628500 0.95329700

C 2.39237600 2.59807900 1.76800600

H 2.20820900 0.84824800 0.52963900

C 0.47280900 4.05809500 1.83093300

H -1.19518200 3.45527500 0.61777300

C 1.77070900 3.75454600 2.24599400

H 3.39935500 2.34686700 2.08444200

H -0.01756800 4.96048000 2.18668300

H 2.29367900 4.41379500 2.93299200

C 1.43025400 1.02429200 -2.30931000

C 1.90628700 2.34669400 -2.28819100

C 2.17577000 0.05198100 -2.99834500

C 3.10792400 2.68000700 -2.90698200

H 1.33056200 3.11172100 -1.77878500

C 3.37919100 0.39059200 -3.61717500

H 1.83629700 -0.97855400 -3.01150000

C 3.85402400 1.70199700 -3.57027400

H 3.46017300 3.70769900 -2.87746100

H 3.94846100 -0.37834600 -4.13258300

H 4.79238700 1.96329500 -4.05178100

C -4.31861800 -1.00548200 2.26355300

H -5.05118500 -0.49986300 2.90415400

H -4.68428900 -2.01409400 2.05907200

H -3.37806600 -1.09075300 2.82020100

C -4.60185100 -2.04249500 -0.78565300

H -4.52699800 -2.78956600 0.00777800

H -5.64249700 -2.02219300 -1.13638400

H -3.98428800 -2.38067300 -1.62125500

C -3.49722000 2.02495800 2.18029800

H -2.76524600 2.82396400 2.04953100

H -4.47352900 2.48482900 2.38689100

H -3.21178400 1.44571500 3.06138300

C -3.31345900 2.95311000 -0.94057100

H -2.47203300 2.99446700 -1.63812800

H -4.21237500 3.28636800 -1.47468800

H -3.12609900 3.66852500 -0.13759100

C -3.92423800 0.40724600 -2.68389300

H -3.68795900 -0.56655800 -3.11709100

H -4.96797100 0.63756800 -2.94021600

H -3.29133300 1.16070900 -3.15914000

C -0.43476700 0.21837000 2.98232500

H -0.54926100 -0.24426600 3.96751700

H -0.86085600 1.22083500 2.98190000

H 0.62299800 0.27931900 2.72786000

O -1.15133600 -0.56699200 2.01937000

H -0.63381900 -1.40441400 1.89539200

S 1.27480100 -2.87910200 1.23908700

O 0.63327100 -2.56647000 2.54956800

O 1.65571500 -4.27115100 0.97984800

C 2.73576000 -1.82912400 1.16722600

C 3.42071800 -1.64108400 -0.03719200

C 3.14961600 -1.18279100 2.33060800

C 4.48738500 -0.74812300 -0.07558800

H 3.09642900 -2.16972600 -0.92543700

C 4.24558300 -0.32079100 2.28099300

H 2.60631100 -1.35154100 3.25324500

C 4.91340000 -0.07087600 1.07673800

H 4.99013900 -0.56424400 -1.02137200

H 4.57258000 0.18123000 3.18846800

C 6.03907700 0.93083100 1.00144700

H 5.70320200 1.85624100 0.51502500

H 6.41139100 1.19314100 1.99695700

H 6.88012800 0.54588900 0.41419500

**int6**

Rh 2.05809100 -0.02653300 0.03305000

C 3.68155700 1.03363800 -1.03410500

C 4.17661900 -0.34375700 -0.91247500

C 3.61916700 1.58893100 0.28451300

C 4.32475100 -0.64736500 0.44492100

C 3.89303100 0.52551600 1.20318900

C -0.46716900 -2.29017100 0.93119900

O -1.25781300 -2.69693100 1.79035700

C 0.79902600 -1.68407900 1.53165300

C 0.81202300 -0.36420800 1.93908700

H 1.47596900 -0.08872600 2.75789200

C 1.72593200 -2.70508400 2.13537800

H 1.13524100 -3.38418500 2.76003800

H 2.21112500 -3.30883100 1.36156800

H 2.49529900 -2.23406800 2.75231400

N -0.55122300 -2.31730000 -0.40637200

C 0.33391100 1.09764400 0.25930200

C -0.15174800 0.67064000 1.43788800

C -0.27772700 1.98544500 -0.72974700

C -1.57648400 1.69827200 -1.19311800

C 0.37601300 3.11611600 -1.24298800

C -2.18889800 2.51586800 -2.13809400

H -2.09365400 0.83114700 -0.80276100

C -0.23966300 3.93572600 -2.18815500

H 1.36139500 3.36599000 -0.87526200

C -1.52393000 3.63567900 -2.64445300

H -3.18788900 2.26851400 -2.48083600

H 0.28344300 4.81085400 -2.56450400

H -2.00351100 4.26901100 -3.38529600

C -1.35311100 1.05804400 2.19667200

C -1.98794100 2.29909300 2.00587800

C -1.89530900 0.16313500 3.13557300

C -3.14220800 2.62113600 2.71331200

H -1.57301500 3.00948600 1.30041800

C -3.04912000 0.49250100 3.84608200

H -1.44360000 -0.81500300 3.26339400

C -3.68035600 1.71964300 3.63693900

H -3.61918900 3.58414000 2.55116300

H -3.46016100 -0.21882200 4.55707300

H -4.58051100 1.97468000 4.18932200

C 4.39622800 -1.26298600 -2.06998000

H 5.02685600 -0.79306800 -2.83327200

H 4.87801500 -2.19142000 -1.75437100

H 3.43383700 -1.51974000 -2.52875200

C 4.86979800 -1.91927900 1.01882500

H 4.48408100 -2.80052100 0.49845500

H 5.96457400 -1.93828300 0.93799800

H 4.62178000 -2.02426200 2.07664800

C 3.57021700 1.76857300 -2.33389900

H 2.77990500 2.52126700 -2.31216700

H 4.51614500 2.27178700 -2.57667300

H 3.34607200 1.07877600 -3.15138400

C 3.39888400 3.01800300 0.67975900

H 2.51835400 3.13563800 1.31852500

H 4.26775700 3.39130700 1.23621700

H 3.27394300 3.65728900 -0.19656700

C 4.00640500 0.69198200 2.68646200

H 3.80031800 -0.24032500 3.21894600

H 5.02374900 1.00579600 2.95717300

H 3.31438500 1.45417400 3.05515300

C 0.58624100 -0.40251400 -2.75921800

H 0.63712600 -1.07233100 -3.62298300

H 1.10628100 0.53290000 -2.97355100

H -0.46140200 -0.18629900 -2.54325300

O 1.21615800 -1.05110200 -1.64933400

H 0.47387500 -1.68252900 -1.19878400

S -1.88461000 -2.95759600 -1.19630400

O -1.50485200 -2.92779600 -2.61841100

O -2.34430700 -4.21012400 -0.59278800

C -3.13991800 -1.69009000 -0.97396300

C -3.56241900 -1.32642500 0.30879600

C -3.64746200 -1.05017300 -2.10243900

C -4.44800300 -0.26398800 0.45196400

H -3.17027400 -1.84712600 1.17362600

C -4.57090600 -0.01566300 -1.94051800

H -3.30550800 -1.35557700 -3.08486300

C -4.95868200 0.41265700 -0.66603400

H -4.73184900 0.05733200 1.44976800

H -4.97601300 0.48183300 -2.81859100

C -5.87187200 1.59851000 -0.48100500

H -5.33311100 2.42464100 0.00033000

H -6.26143800 1.96070000 -1.43758200

H -6.72496300 1.35228600 0.16187100

**int7**

Rh -1.35673100 -0.84818300 0.26435800

C -1.37872500 -1.16524600 2.45696300

C -0.38612200 -2.04118000 1.90391800

C -2.67518300 -1.58884000 1.95513700

C -1.06382600 -2.96621800 1.03053100

C -2.47693600 -2.69745100 1.08467000

C 0.58248200 -0.83184500 -1.98991500

O 0.93428800 0.26952000 -2.43799900

C -0.91484900 -1.18207800 -1.99860300

C -1.93982400 -0.24532900 -1.79497900

H -2.94278400 -0.46090100 -2.15774100

C -1.25605300 -2.48076400 -2.67874200

H -0.99050000 -2.39921400 -3.74200600

H -0.65296000 -3.29164200 -2.27089600

H -2.32076000 -2.72512400 -2.60528900

N 1.26689200 -1.84960100 -1.45846300

C -0.61215500 0.89451000 -0.18190000

C -1.73961500 0.96054200 -1.02379200

C 0.25573200 1.91383500 0.32079600

C 0.26605500 3.20687600 -0.25044100

C 1.13243500 1.63689800 1.38743000

C 1.07218900 4.20202500 0.28506500

H -0.36681100 3.41207300 -1.10592000

C 1.92950200 2.63800000 1.92654800

H 1.19457100 0.62164100 1.75325300

C 1.88771400 3.92671500 1.38845700

H 1.06815700 5.19527700 -0.15401400

H 2.60313600 2.41015000 2.74542500

H 2.50908100 4.71053600 1.81173000

C -2.75182900 2.03804200 -1.03089500

C -3.05508700 2.74700000 0.14303500

C -3.42549100 2.36357200 -2.21680900

C -4.01157400 3.75706700 0.12896100

H -2.53651600 2.49472800 1.06276100

C -4.38240900 3.37869500 -2.22923600

H -3.17743300 1.83615300 -3.13312900

C -4.67899700 4.07588300 -1.05779400

H -4.24079500 4.29543900 1.04409200

H -4.89138800 3.62721700 -3.15590000

H -5.42494200 4.86517900 -1.06749100

C -3.98336700 -0.95638800 2.31259800

H -4.74645900 -1.16348900 1.55776000

H -4.35241200 -1.33937700 3.27349100

H -3.88902600 0.12980600 2.40388300

C -1.16953000 -0.12814900 3.51715200

H -1.29441400 -0.57140200 4.51405200

H -0.16946000 0.30591000 3.46867600

H -1.89106200 0.68893500 3.42622700

C -3.54106600 -3.46403900 0.35993400

H -3.88443000 -4.32008300 0.95547400

H -4.41298800 -2.83872000 0.14724900

H -3.16932700 -3.85393400 -0.59091900

C -0.38029800 -4.08683900 0.31895000

H -0.04528000 -4.83491200 1.04983600

H -1.05138300 -4.58685900 -0.38296500

H 0.49903600 -3.72245400 -0.22364200

C 1.07052400 -2.12189100 2.22971600

H 1.44815000 -1.19443900 2.66604600

H 1.23802000 -2.92048000 2.96632700

H 1.66522000 -2.36186600 1.34400300

S 2.90614100 -1.89317500 -1.39560800

O 3.19997600 -2.99701100 -0.45487700

O 3.50757900 -1.92356700 -2.73813200

C 3.53130000 -0.40586300 -0.58481500

C 3.57914100 0.81213100 -1.26861000

C 4.08083800 -0.52487200 0.69069300

C 4.17460200 1.91013800 -0.65661600

H 3.13645900 0.88751400 -2.25247500

C 4.67818200 0.58538700 1.28856500

H 4.06449800 -1.48977000 1.18396600

C 4.74167600 1.81345300 0.62211500

H 4.20417600 2.86178000 -1.18176800

H 5.11759700 0.49083500 2.27929100

C 5.41703600 3.00710900 1.25014300

H 6.40113700 3.18930700 0.79948100

H 4.82461500 3.91809800 1.10769600

H 5.57010300 2.86349200 2.32499300

**TS8**

Rh -1.54951100 -0.83115500 -0.14958400

C -2.61390300 -1.58933000 1.64134800

C -1.37810500 -2.31682600 1.54410500

C -3.43476400 -1.94154700 0.49789300

C -1.40472800 -3.04566000 0.30789900

C -2.68769100 -2.83115900 -0.32278500

C 1.16024300 -0.31550400 -1.10602700

O 1.36509600 0.85040800 -0.70867600

C -0.10107900 -0.63028500 -1.91120100

C -1.19376300 0.25529300 -2.03443500

H -1.87906500 0.13906300 -2.87083200

C 0.02161000 -1.72616400 -2.94258300

H 0.76841300 -1.43638200 -3.69322800

H 0.38608800 -2.64676000 -2.48796900

H -0.92932200 -1.91240300 -3.45217300

N 1.88401400 -1.42186400 -0.91139300

S 3.01223200 -1.42629600 0.29515200

O 2.42591400 -0.91335300 1.55713600

O 3.57228000 -2.78645900 0.30463800

C 4.34522400 -0.31413300 -0.15593200

C 4.24044300 1.05269900 0.10407900

C 5.49086800 -0.84861300 -0.74405400

C 5.30118700 1.88664700 -0.24034100

H 3.33720000 1.44217100 0.55511900

C 6.54112700 0.00182600 -1.08417800

H 5.55391600 -1.91858400 -0.90769600

C 6.46093700 1.37917800 -0.84250500

H 5.23000500 2.95253200 -0.03655300

H 7.44006900 -0.41034400 -1.53651600

C 7.58852100 2.29881300 -1.24437000

H 7.70635000 3.12249000 -0.53241800

H 8.54032600 1.76173500 -1.30785700

H 7.40125500 2.74728100 -2.22988700

C -0.85183600 0.96013400 0.19920000

C -1.53887300 1.21018100 -1.01021400

C -0.37282700 1.78826500 1.26174400

C -0.43480200 3.19725000 1.18368800

C 0.23201700 1.18695100 2.38205800

C 0.06065100 3.97490800 2.22129100

H -0.87312600 3.66388700 0.30860700

C 0.74026800 1.97284300 3.41077100

H 0.35910100 0.11452900 2.39128700

C 0.64618400 3.36443600 3.33774400

H 0.00476300 5.05787200 2.16059800

H 1.22799000 1.50059400 4.25781200

H 1.04360100 3.97787900 4.14159500

C -2.62576500 2.19234300 -1.20544700

C -3.48399100 2.52545400 -0.14387800

C -2.82098800 2.79976100 -2.45374800

C -4.51240400 3.44374400 -0.32974600

H -3.33653000 2.05498600 0.82366300

C -3.85345300 3.72009000 -2.63791100

H -2.14703900 2.56618900 -3.27251800

C -4.70178000 4.04340700 -1.57872300

H -5.17097400 3.69033300 0.49815800

H -3.98969600 4.18839000 -3.60838400

H -5.50540900 4.75959300 -1.72275500

C -4.81819800 -1.43066800 0.23756200

H -5.56141000 -1.99800200 0.81348600

H -4.91572800 -0.37741400 0.51872700

H -5.08217900 -1.51651200 -0.81989100

C -3.04053200 -0.74711200 2.80454300

H -2.20669800 -0.15735800 3.19520800

H -3.83939200 -0.05476800 2.52301500

H -3.41925300 -1.37507800 3.62177200

C -3.14272400 -3.47074300 -1.59972000

H -3.58198800 -4.45902300 -1.40979800

H -3.89928500 -2.86520600 -2.10725400

H -2.30846400 -3.60988500 -2.29194100

C -0.30849500 -3.95448300 -0.15063700

H -0.14823600 -4.74951600 0.58872100

H -0.55225500 -4.42983300 -1.10385500

H 0.63700100 -3.41067100 -0.26515800

C -0.30339200 -2.42982800 2.57946000

H -0.50937500 -1.78044100 3.43449600

H -0.25880800 -3.46211200 2.95208300

H 0.68487300 -2.17124000 2.18647800

**int9**

Rh -1.37812300 -0.89599000 -0.21682900

C -2.61539200 -1.70984300 1.52822300

C -2.21950900 -2.87621000 0.76264700

C -3.39997000 -0.86509000 0.68901400

C -2.70317200 -2.73658400 -0.55341700

C -3.39772500 -1.45020300 -0.63661900

C 0.67679500 -0.67768000 -1.27559100

O 0.73871900 0.15720000 -0.16431900

C -0.43882900 -0.37303300 -2.16598500

C -0.64689700 1.08265600 -2.36223500

H -0.64700500 1.49775700 -3.36817900

C -0.61467600 -1.27183400 -3.36434600

H 0.07522300 -0.98722700 -4.17166900

H -0.40755100 -2.31301800 -3.11335000

H -1.63353500 -1.19258700 -3.76014900

N 1.50498900 -1.67777700 -1.42186900

S 2.32352200 -2.27184800 -0.09464800

O 1.52651100 -2.13903900 1.14823200

O 2.79705000 -3.60372400 -0.48088400

C 3.76019100 -1.21166500 0.04955100

C 3.72301000 -0.06386000 0.84089300

C 4.90397600 -1.54541000 -0.67657600

C 4.84708900 0.75679700 0.89643800

H 2.82961700 0.18246900 1.39920100

C 6.01810100 -0.71298400 -0.60984700

H 4.91111700 -2.45004900 -1.27421200

C 6.00804400 0.44782100 0.17525500

H 4.82380100 1.65254300 1.51216800

H 6.91114400 -0.96839000 -1.17467200

C 7.23041400 1.32780200 0.26594600

H 6.95981900 2.36895700 0.46965800

H 7.89360600 0.99776000 1.07649300

H 7.81195400 1.30100700 -0.66135800

C -0.47529600 0.98479600 -0.03651800

C -0.64081300 1.84767900 -1.25323100

C -0.58877900 1.47914500 1.34779400

C -1.50925900 2.49418200 1.66662100

C 0.15018300 0.88693100 2.39060300

C -1.67091500 2.91611800 2.98483300

H -2.09436000 2.95482600 0.87938000

C -0.01092300 1.32165900 3.70367600

H 0.81739200 0.06235200 2.16735600

C -0.91865400 2.33850400 4.01017100

H -2.38344700 3.70523600 3.20853300

H 0.57013400 0.85363700 4.49350500

H -1.04163900 2.67442800 5.03570800

C -0.61037600 3.31977500 -1.25651900

C -1.46525900 4.04158700 -2.10618200

C 0.27864200 4.03579900 -0.43598300

C -1.42541100 5.43413600 -2.14457400

H -2.17224200 3.49762900 -2.72659600

C 0.31581400 5.42738600 -0.47460100

H 0.94714600 3.49479200 0.22565100

C -0.53534700 6.13387200 -1.32758400

H -2.09689700 5.97369700 -2.80695400

H 1.01448700 5.96235300 0.16270500

H -0.50773400 7.21944500 -1.35214500

C -4.11968300 0.37626800 1.10496500

H -5.10659000 0.12714300 1.51780200

H -3.56370500 0.92176400 1.87124900

H -4.27230700 1.05206100 0.25891900

C -4.18016200 -0.95578900 -1.81114500

H -5.20434500 -1.35323700 -1.79253500

H -4.24148000 0.13578500 -1.81327000

H -3.71999200 -1.26705600 -2.75243100

C -2.56284600 -3.72589000 -1.66810000

H -3.37106600 -4.46898600 -1.63279000

H -2.60899200 -3.23818500 -2.64432600

H -1.61153900 -4.26167800 -1.60959300

C -1.35347500 -3.97372000 1.29627800

H -1.81368400 -4.43586500 2.17858400

H -1.19203500 -4.75508700 0.55014500

H -0.37086600 -3.58525200 1.58733400

C -2.31036200 -1.49280400 2.97668800

H -3.03961400 -2.01576800 3.61115900

H -1.31663800 -1.87460300 3.22748600

H -2.33329600 -0.43246100 3.23845900

**TS10**

C 1.40068800 -2.99421200 0.18211900

C 1.28825300 -3.13639100 -1.24585700

C 0.12762900 -2.51597800 0.64393600

C -0.11134000 -2.91953500 -1.62636300

C -0.82229500 -2.55323000 -0.47846500

C 2.35458300 -3.62754900 -2.16984200

H 2.28416500 -4.71828500 -2.28387900

H 2.25431800 -3.18352000 -3.16428000

H 3.35077300 -3.39267600 -1.78738500

C 2.57157000 -3.35728900 1.03567300

H 2.64550700 -2.71161600 1.91301300

H 2.47047900 -4.39414000 1.38275800

H 3.51247800 -3.27327700 0.48880600

C -0.62834400 -3.02373300 -3.02635900

H -0.65995300 -4.06999500 -3.35719800

H -1.63551200 -2.61061700 -3.11107200

H 0.01443700 -2.47973200 -3.72712400

C -2.26501300 -2.19237400 -0.36162100

H -2.69491200 -2.62717600 0.54594800

H -2.39937000 -1.10450800 -0.28305200

H -2.83531600 -2.55054000 -1.22259000

C -0.22734600 -2.16779300 2.04773800

H -0.66275000 -3.04832800 2.54341300

H 0.66074000 -1.86896100 2.60977700

H -0.96081100 -1.35608800 2.09558000

C -0.75784200 1.12823500 -0.04941100

O 0.03400000 1.27846900 0.89884100

C -0.22656800 0.90004200 -1.47751000

C 1.09843400 1.06975000 -1.82090400

H 1.33369800 1.08961400 -2.88640400

C 2.25446900 1.26784200 -0.88731600

C 2.46179800 0.13778900 -0.16380900

C 2.98700400 2.53543600 -0.90601300

C 2.39573800 3.65282700 -1.52503000

C 4.25883600 2.68606500 -0.31914600

C 3.05017200 4.88078400 -1.55719100

H 1.40435400 3.55733000 -1.95743800

C 4.90915300 3.91447900 -0.35323300

H 4.73254400 1.83888200 0.16055200

C 4.31044800 5.01593000 -0.97236300

H 2.57299700 5.73431500 -2.02990100

H 5.88902100 4.01493300 0.10484800

H 4.82207400 5.97390200 -0.99397900

C 3.37227000 -0.14938100 0.94079700

C 4.63157000 -0.73941300 0.74997000

C 2.92959500 0.12363800 2.24877100

C 5.43884300 -1.04758900 1.84495700

H 4.97166800 -0.94888000 -0.26100800

C 3.74126300 -0.19054500 3.33762900

H 1.95292100 0.58014900 2.37915500

C 4.99477000 -0.77832100 3.14142300

H 6.41412200 -1.49966000 1.68596500

H 3.39447000 0.02730600 4.34398200

H 5.62273400 -1.02161700 3.99366900

C -1.24072200 0.70376000 -2.57802700

H -1.89798200 -0.14076300 -2.36480300

H -1.88863500 1.58372400 -2.63874200

H -0.75062700 0.54638700 -3.54496300

N -2.09962300 1.09934000 -0.04578700

S -2.84924100 1.20921000 1.42641000

O -2.52164900 0.05283000 2.29694700

O -2.79467800 2.56231900 2.00066500

C -4.53216400 0.93444200 0.86127700

C -5.18617800 1.93395000 0.13881700

C -5.17490000 -0.26173700 1.16125400

C -6.48881200 1.71762100 -0.29722700

H -4.67297100 2.86600100 -0.07225500

C -6.48369300 -0.46525600 0.71935500

H -4.64876900 -1.01169900 1.74115400

C -7.15794500 0.51599900 -0.01416400

H -7.00137900 2.49254200 -0.86270800

H -6.99005500 -1.39874300 0.95314300

C -8.57999600 0.30617300 -0.47495000

H -8.89451300 -0.73375300 -0.34201200

H -9.27707000 0.93784400 0.09061900

H -8.69989900 0.56396200 -1.53380100

Rh 0.99216200 -1.05956900 -0.73722100

**int11**

Rh -1.86944000 -0.00700700 -0.20747200

C -3.36869100 1.59548800 0.23625200

C -3.01200300 0.87762900 1.42713900

C -3.94753600 0.64977500 -0.67042600

C -3.53838200 -0.48500800 1.30471100

C -4.12581700 -0.61770200 0.03537600

C -0.07743200 -1.99374100 -0.30200500

O -0.35370800 -1.20479900 0.68464000

C -0.90745800 -1.69084200 -1.52863000

C -0.74082500 -0.48996200 -2.19380500

H -1.31214400 -0.33934700 -3.11047300

C -1.75293900 -2.81025300 -2.06752200

H -1.08751800 -3.59375000 -2.44817800

H -2.36437900 -3.26582700 -1.28342900

H -2.40273100 -2.46891300 -2.87918900

N 0.76942500 -2.97586500 -0.37761300

C -0.22880800 1.13804900 -0.67219600

C 0.24720400 0.57009900 -1.78711900

C 0.28469800 2.14550300 0.24666700

C 0.20233500 3.51970000 -0.04208700

C 0.84191100 1.74068800 1.47312800

C 0.66668500 4.46622700 0.87146100

H -0.22303000 3.83535400 -0.99052900

C 1.30763700 2.69045100 2.37876700

H 0.91029800 0.68151000 1.69192200

C 1.21886900 4.05485900 2.08658900

H 0.59972500 5.52409400 0.63259600

H 1.74809200 2.36039000 3.31501100

H 1.58063700 4.79133500 2.79845400

C 1.52833700 0.73419400 -2.49110300

C 2.31295300 1.89435600 -2.36674100

C 2.02331200 -0.32755200 -3.26822900

C 3.55614900 1.97878300 -2.98801100

H 1.95096000 2.72488600 -1.77302400

C 3.27037800 -0.24242000 -3.88463600

H 1.44163200 -1.24073600 -3.35448400

C 4.04403500 0.91130900 -3.74692900

H 4.14834000 2.88340500 -2.87854700

H 3.64065700 -1.08212600 -4.46583800

H 5.01621900 0.97895100 -4.22648100

C -4.48183000 0.94919300 -2.03715000

H -4.37992600 0.08595700 -2.70118600

H -5.54870500 1.20620800 -1.99143800

H -3.95436500 1.78956400 -2.49554300

C -3.19213000 3.06093100 0.00327300

H -4.06139200 3.61043500 0.38844000

H -2.30283300 3.44331700 0.50861500

H -3.09769600 3.29077600 -1.06151500

C -4.81065000 -1.82219700 -0.53059100

H -5.90075300 -1.73136800 -0.43389000

H -4.59059000 -1.94893100 -1.59469600

H -4.50722400 -2.73764300 -0.01652700

C -3.35014700 -1.54183500 2.34778400

H -3.77533400 -1.22329500 3.30695700

H -3.82783400 -2.48136300 2.06040800

H -2.28288500 -1.74250500 2.50071900

C -2.40400000 1.44934900 2.66907800

H -1.77906300 2.31721400 2.44700500

H -3.18518300 1.76048200 3.37622400

H -1.77328400 0.70929600 3.16971400

S 1.73034600 -3.30678300 0.96786000

O 0.91644700 -3.56700800 2.16635100

O 2.69059700 -4.31786700 0.51880800

C 2.61844100 -1.76863700 1.22586200

C 2.77380300 -1.27930600 2.51997500

C 3.18111900 -1.10393100 0.13557200

C 3.50344400 -0.10681100 2.71880700

H 2.31842600 -1.81136500 3.34806800

C 3.88536700 0.07694700 0.34752200

H 3.05700300 -1.49378800 -0.86801500

C 4.05635100 0.59350300 1.63953200

H 3.63495700 0.27717600 3.72778400

H 4.29814300 0.60756500 -0.50516300

C 4.77638700 1.90137900 1.84919600

H 5.15449600 1.99279000 2.87272400

H 5.62208400 2.00818300 1.16149300

H 4.09720400 2.74510100 1.67032700

**int12**

Rh -0.85746400 -0.86519500 0.03694000

C -1.46333500 -1.34290300 -2.10933300

C -2.53870600 -0.62391200 -1.48166600

C -1.28384000 -2.56008600 -1.39117200

C -3.10471200 -1.48073000 -0.44661100

C -2.34333900 -2.65820500 -0.39105600

C -0.01268000 0.00568400 2.45445500

O 0.54102400 0.45422800 3.45315700

C 0.17746400 -1.43724100 1.99100800

C 1.03497700 -1.75619100 0.94046700

H 1.22421900 -2.81476900 0.75281800

C -0.40949400 -2.49165600 2.88782700

H 0.06031300 -2.40487200 3.87519500

H -1.48490200 -2.34007300 3.02613000

H -0.23805000 -3.50124500 2.50332900

N -0.90071800 0.56249600 1.59843900

C 0.90242600 0.11399300 -0.38152200

C 1.80002900 -0.74454500 0.15292200

C 1.05436000 1.44968300 -0.94788300

C 2.22966800 2.20204100 -0.73593100

C -0.01458300 2.08299200 -1.60204200

C 2.33171400 3.51058800 -1.19491000

H 3.05427000 1.76228800 -0.18832900

C 0.08548500 3.39325300 -2.05951100

H -0.94401600 1.54535200 -1.70366600

C 1.26336200 4.11401700 -1.86439400

H 3.24574900 4.06982200 -1.01377000

H -0.76741900 3.85867500 -2.54525800

H 1.34313500 5.14063400 -2.21041100

C 3.26423000 -0.85633400 0.05191800

C 3.94711400 -0.54743500 -1.13802800

C 4.00342600 -1.34429600 1.14240800

C 5.32746400 -0.70072300 -1.22448800

H 3.38131800 -0.18432700 -1.99061900

C 5.38621100 -1.50002300 1.05345100

H 3.48629600 -1.57741100 2.06934200

C 6.05389800 -1.17778900 -0.12896300

H 5.83905000 -0.45619300 -2.15147400

H 5.94300300 -1.86765200 1.91102900

H 7.13111400 -1.29910700 -0.19917000

C -0.29785100 -3.63607600 -1.73403700

H -0.08314900 -4.27384700 -0.87173900

H -0.68085000 -4.28362300 -2.53460800

H 0.64990400 -3.20962800 -2.07582500

C -0.67483300 -0.93114200 -3.31554500

H -1.04313100 -1.45042500 -4.21027600

H -0.73748600 0.14308900 -3.49700200

H 0.38400100 -1.17952200 -3.19793300

C -2.57323000 -3.84556000 0.49084100

H -3.17492500 -4.60466500 -0.02675500

H -1.63012200 -4.32010900 0.77609200

H -3.10005400 -3.57438500 1.40951200

C -4.31106100 -1.14053100 0.37161200

H -5.22605900 -1.20508200 -0.23287600

H -4.42559100 -1.82001200 1.22015400

H -4.25151100 -0.12332000 0.76822800

C -3.20844200 0.61654200 -1.99744000

H -2.72544200 0.98218100 -2.90706400

H -4.25609600 0.40270300 -2.24568300

H -3.20074200 1.42931800 -1.26554300

C -1.13037300 1.94246800 1.48916500

C -2.38672600 2.38357400 1.04913600

C -0.12781100 2.89552700 1.73871000

C -2.62965100 3.73646700 0.82031400

H -3.16629000 1.64574900 0.89663000

C -0.37715400 4.24544700 1.51197300

H 0.83851700 2.56304500 2.09470900

C -1.62132900 4.67549400 1.04245100

H -3.60928300 4.05683900 0.47449300

H 0.41485200 4.96796900 1.68868000

H -1.80398800 5.73069300 0.86025900

**TS13**

Rh 1.17882300 -0.56832500 -0.14171300

C 2.53445700 -0.16177400 1.68921600

C 3.42945200 -0.27139900 0.56238800

C 1.84241100 -1.41122500 1.82178900

C 3.27291600 -1.55800600 -0.00873400

C 2.25835400 -2.27026700 0.74517300

C 0.38419600 0.34680400 -2.34615100

O 0.70054700 0.84573100 -3.41895400

C 0.36987300 -1.16772200 -2.08688600

C -0.97875200 -1.63967500 -1.83324500

H -1.46711500 -2.29718200 -2.55438700

C 1.17186200 -1.94857100 -3.10549500

H 0.65189100 -1.98557600 -4.07251800

H 2.14340600 -1.48233800 -3.28197000

H 1.33131000 -2.97853700 -2.76642800

N 0.16462800 1.01073600 -1.15627800

C -0.82397400 -0.33927900 0.09884400

C -1.67414800 -1.08772700 -0.78559600

C -1.33172400 0.26484700 1.33635600

C -0.85919300 1.48333000 1.85575700

C -2.24398200 -0.47798500 2.11744300

C -1.30862400 1.95737100 3.08518600

H -0.14533500 2.06285600 1.29059600

C -2.66225400 -0.01617600 3.36261200

H -2.61076400 -1.42883900 1.75049600

C -2.20766100 1.21013300 3.84973200

H -0.94347300 2.91307700 3.45104400

H -3.35393900 -0.61531800 3.94805400

H -2.54555500 1.57715700 4.81456200

C -3.15634300 -1.12446300 -0.72541200

C -3.89751300 0.05771900 -0.56765100

C -3.84697200 -2.33872800 -0.85184100

C -5.28961100 0.02264900 -0.53479600

H -3.37380300 1.00382000 -0.47090800

C -5.24098500 -2.37235300 -0.82161400

H -3.28222000 -3.26100800 -0.96116700

C -5.96807500 -1.19192900 -0.66050700

H -5.84684200 0.94758600 -0.41410100

H -5.75870400 -3.32290200 -0.91775700

H -7.05366300 -1.21762800 -0.63313100

C 4.39758700 0.77702900 0.10280700

H 5.40050400 0.59216500 0.51151500

H 4.48331000 0.79630200 -0.98765700

H 4.09528000 1.77573400 0.42907400

C 2.45515300 0.98883400 2.64425400

H 3.20509800 0.89253100 3.44182700

H 2.63337000 1.94422100 2.14166000

H 1.47166700 1.04705400 3.11622200

C 0.90387600 -1.77583600 2.92892800

H 1.46255300 -2.17342600 3.78705900

H 0.32903100 -0.91313300 3.27361600

H 0.19027800 -2.54174600 2.61310000

C 1.86688900 -3.70433000 0.55449200

H 2.51501800 -4.37828100 1.13210500

H 0.83524400 -3.87753800 0.87413100

H 1.93912800 -3.99818900 -0.49692500

C 4.07017800 -2.12140800 -1.14296100

H 4.40943100 -1.33741400 -1.82624300

H 4.96175000 -2.64201400 -0.76662700

H 3.49064700 -2.84255300 -1.72387800

C 0.17006600 2.41300500 -1.08809100

C -0.90527900 3.14140400 -1.61485500

C 1.22785300 3.09221300 -0.47076200

C -0.91698500 4.53130300 -1.52155300

H -1.71877700 2.60439300 -2.09243000

C 1.20674400 4.48203900 -0.37185500

H 2.05330600 2.50972200 -0.07748000

C 0.13345500 5.20695600 -0.89502500

H -1.75264400 5.08962400 -1.93429300

H 2.03143000 5.00024100 0.11013400

H 0.11588000 6.29003100 -0.81632300

**int14**

Rh -1.24123100 -0.52675500 -0.21401200

C -2.00506200 -0.85417100 1.90484400

C -2.36416200 -1.96887200 1.08937600

C -2.68400300 0.31626300 1.39468800

C -3.27214800 -1.48332900 0.07133900

C -3.49695600 -0.08788400 0.29284700

C 1.02919500 -2.01396600 -1.37272200

O 1.49670700 -3.09956200 -1.69161400

C -0.27910400 -1.52609400 -1.88570500

C -0.41198000 -0.11513600 -2.13643400

H -1.00075000 0.26945100 -2.96380700

C -1.03201500 -2.51190500 -2.74120900

H -0.44227300 -2.79266300 -3.62197700

H -1.22770300 -3.43355200 -2.18597300

H -1.98631900 -2.09254800 -3.07183400

N 1.67771700 -1.08511300 -0.55309600

C 0.86279600 0.04439600 -0.12496800

C 0.16976900 0.77793700 -1.18427800

C 1.21889400 0.59730900 1.20541400

C 0.83246300 1.88260200 1.62841100

C 1.93108600 -0.20049100 2.12243100

C 1.15945400 2.34998500 2.90036100

H 0.26579100 2.52409000 0.96960700

C 2.25723600 0.26989300 3.39118300

H 2.22761100 -1.20150000 1.83667100

C 1.87930600 1.55207100 3.79110800

H 0.84866800 3.34993500 3.19147900

H 2.80951400 -0.37400200 4.07001400

H 2.13777800 1.92208600 4.77897400

C 0.01816000 2.24467400 -1.36182200

C -1.25251400 2.83386300 -1.42159200

C 1.14729500 3.05995300 -1.52198000

C -1.39248600 4.20707500 -1.61678200

H -2.12402500 2.20069100 -1.29355200

C 1.00863100 4.43378500 -1.71451500

H 2.13458200 2.61116300 -1.50301200

C -0.26096300 5.01317100 -1.75783400

H -2.38469200 4.64863200 -1.65321400

H 1.89404700 5.05193400 -1.83261400

H -0.36765100 6.08443900 -1.90202400

C 3.10070300 -1.01342100 -0.52235000

C 3.87732700 -2.18114300 -0.46243600

C 3.74379100 0.23309000 -0.51605300

C 5.26784100 -2.08935000 -0.42473200

H 3.38658300 -3.14321700 -0.46408700

C 5.13317600 0.31090400 -0.46218600

H 3.15765500 1.14238300 -0.52423900

C 5.90702700 -0.84907800 -0.42191300

H 5.85409200 -3.00346300 -0.38494700

H 5.60925000 1.28763900 -0.45335700

H 6.99055700 -0.78683900 -0.38143000

C -4.00004300 -2.33145700 -0.92549100

H -3.43372400 -3.22905700 -1.18417900

H -4.96711800 -2.65899200 -0.51951200

H -4.20128400 -1.78380600 -1.85130300

C -1.94412900 -3.39375400 1.28880800

H -2.65434200 -3.93645500 1.92807400

H -1.88237700 -3.92726400 0.33561400

H -0.95868800 -3.45174000 1.75944700

C -1.16188500 -0.90066700 3.13838100

H -1.78498400 -1.12502300 4.01521900

H -0.39098800 -1.67257000 3.07220600

H -0.65717100 0.04922000 3.32125200

C -2.64409900 1.68417500 2.00272000

H -3.47123100 1.82456800 2.71215100

H -1.70981900 1.84772800 2.54297800

H -2.72557100 2.46564700 1.24057500

C -4.45309600 0.77735700 -0.47041400

H -5.47353800 0.68241500 -0.07351100

H -4.18007600 1.83463200 -0.40342400

H -4.49111200 0.50559400 -1.53011400

**int15**

Rh -0.65559800 -0.83528700 -0.29374700

C -0.78366500 -1.49010600 -2.44421600

C -1.87823000 -0.58675300 -2.19819800

C -0.97560100 -2.62797400 -1.60289600

C -2.80447100 -1.24630300 -1.29099100

C -2.25152200 -2.47854700 -0.90886300

C -0.19225200 0.03215000 2.23128300

O 0.21537400 0.50088000 3.28649700

C 0.03460400 -1.42413700 1.82433900

C 1.04006000 -1.77204800 0.92770400

H 1.22670900 -2.83636200 0.77854700

C -0.71212000 -2.45375800 2.62787000

H -0.35565300 -2.40192400 3.66481900

H -1.78505300 -2.24051900 2.63891400

H -0.53933000 -3.46672700 2.25143300

N -0.96031700 0.58744800 1.25011800

C 1.18212800 0.11040700 -0.37715500

C 1.95127000 -0.78194700 0.28607000

C 1.48160000 1.45648300 -0.84762200

C 2.54215900 2.19605000 -0.28077600

C 0.64893700 2.10804500 -1.76900300

C 2.76750600 3.51635700 -0.65293500

H 3.17145700 1.73330400 0.47033500

C 0.87046800 3.43129700 -2.13877800

H -0.19838800 1.56732800 -2.15767700

C 1.93503700 4.14259200 -1.58548200

H 3.58592000 4.06769900 -0.19777500

H 0.19694500 3.91297400 -2.84203900

H 2.10501300 5.17933100 -1.86164200

C 3.40880800 -0.93079500 0.41854200

C 4.28256600 -0.55048500 -0.61642000

C 3.95242000 -1.52161000 1.57153700

C 5.65522900 -0.73729100 -0.49050000

H 3.87005800 -0.10622400 -1.51682700

C 5.32841100 -1.71145000 1.69458800

H 3.28855500 -1.81139400 2.38168800

C 6.18537700 -1.31926700 0.66554300

H 6.31529700 -0.43699800 -1.29977400

H 5.73121300 -2.16129800 2.59790500

H 7.25734400 -1.46745800 0.76007200

C -0.11159400 -3.85203400 -1.57283500

H -0.19356600 -4.37463100 -0.61580600

H -0.41331300 -4.55765400 -2.35859600

H 0.94156200 -3.60461600 -1.73374700

C 0.33684200 -1.29529600 -3.42065900

H 0.08718700 -1.73752900 -4.39439900

H 0.55384700 -0.23574500 -3.57525400

H 1.25764900 -1.76665600 -3.06576100

C -2.88931600 -3.46923600 0.01594900

H -3.57966300 -4.12856500 -0.52822800

H -2.14567300 -4.10724200 0.50191700

H -3.45598100 -2.95801100 0.79985300

C -4.15116500 -0.72178900 -0.89689800

H -4.93943700 -1.33515200 -1.35380200

H -4.29364700 -0.73940600 0.18802500

H -4.29346200 0.30431200 -1.24569500

C -2.23622500 0.63197800 -2.99808300

H -1.44336800 0.90086400 -3.69988700

H -3.14158900 0.43849700 -3.58831000

H -2.43563100 1.50018200 -2.36195100

C -1.16355500 1.96957700 1.07911800

C -0.26528800 2.93955600 1.55101900

C -2.28820700 2.37951700 0.34705500

C -0.48134700 4.28388200 1.25903300

H 0.59340900 2.62915500 2.13081900

C -2.49810900 3.72626500 0.06191700

H -2.99005200 1.62461500 0.01091500

C -1.58915600 4.68693800 0.50987200

H 0.23261600 5.02302800 1.61158200

H -3.37410100 4.02523600 -0.50812500

H -1.74477800 5.73778600 0.28332100

C -3.61299100 0.21963600 3.32713900

H -4.44151500 -0.20640300 3.89997300

H -2.72208500 0.26188900 3.96836100

H -3.87656400 1.24606800 3.03544900

O -3.40517300 -0.61415800 2.19266800

H -2.62105600 -0.24918000 1.72822100

**TS16**

Rh 0.42756400 -0.49453800 -0.60506000

C 0.87697900 -2.69824100 -0.99156200

C 2.18435100 -2.06863300 -0.84833500

C 0.23379000 -2.10209100 -2.11045200

C 2.35214500 -1.09129500 -1.85165600

C 1.11830300 -1.05287100 -2.60362300

C 0.42611800 1.95897500 0.98342400

O -0.60383600 2.40043000 1.49202500

C 0.49854200 1.87820000 -0.54556400

C -0.61926800 1.43464500 -1.24430500

H -0.61528300 1.55197400 -2.32804300

C 1.61892200 2.52302600 -1.32222800

H 1.61221800 3.60515300 -1.14255000

H 2.60215100 2.16237900 -1.02341500

H 1.48345800 2.36293100 -2.39687700

N 1.41139400 1.31703300 1.67785600

C -1.54473700 -0.27508800 -0.06972400

C -1.86154700 0.91627000 -0.59827400

C -2.31138900 -1.08447300 0.87271400

C -2.80707900 -0.44418500 2.02692700

C -2.56867700 -2.45194300 0.70167000

C -3.54651200 -1.15645700 2.96669200

H -2.58378700 0.60809200 2.17151300

C -3.30766800 -3.16320600 1.64577800

H -2.19592500 -2.95138000 -0.18294000

C -3.79878700 -2.51896400 2.78325300

H -3.92038100 -0.64777700 3.85105100

H -3.50129000 -4.22169100 1.49255000

H -4.37142900 -3.07397900 3.52089900

C -3.12797500 1.66051400 -0.62440200

C -4.36649500 0.99934800 -0.67624300

C -3.10795400 3.06435200 -0.61698700

C -5.55352900 1.72602100 -0.71436600

H -4.38892500 -0.08568800 -0.68654000

C -4.29729900 3.78866300 -0.65851500

H -2.15395700 3.57356800 -0.52774900

C -5.52405200 3.12314400 -0.70870700

H -6.50453900 1.20173100 -0.75432000

H -4.26758800 4.87472800 -0.64035300

H -6.45132400 3.68848600 -0.74061400

C 3.17158000 -2.47757900 0.18856200

H 3.52608300 -3.49662400 -0.01878100

H 4.03344300 -1.80984400 0.21657200

H 2.68924100 -2.47424500 1.17042400

C 3.58151800 -0.26946000 -2.09298400

H 4.32830400 -0.83925000 -2.66199700

H 3.35332100 0.63155700 -2.66685600

H 4.04401300 0.04463900 -1.15236500

C 0.44333900 -3.87949100 -0.18201300

H -0.50768900 -4.28472600 -0.53219700

H 1.19026300 -4.68081500 -0.24865500

H 0.33272600 -3.61245400 0.87228300

C -1.04807300 -2.50051400 -2.77541600

H -1.71458000 -1.64294900 -2.90322100

H -0.84708000 -2.92545200 -3.76751800

H -1.58524300 -3.25199800 -2.19389500

C 0.88208000 -0.27767100 -3.86046100

H 1.39773900 0.68515100 -3.84857800

H 1.26344500 -0.84539300 -4.72039600

H -0.18286100 -0.09824600 -4.02844400

C 2.75708500 1.56342500 1.54353900

C 3.28469100 2.84858900 1.26377500

C 3.68801200 0.52839700 1.80449200

C 4.65709800 3.05866200 1.16609100

H 2.59589400 3.68007100 1.14527700

C 5.05683200 0.74916700 1.70602000

H 3.30333400 -0.44453900 2.08499300

C 5.55949500 2.01136000 1.36845200

H 5.02595500 4.05697100 0.94245100

H 5.74261200 -0.07199900 1.90362500

H 6.62956100 2.18026100 1.29483700

C 0.10933200 -1.65916500 3.09477900

H 0.77256500 -2.01455500 3.89722400

H -0.55637000 -2.47528500 2.79917000

H -0.50781900 -0.84255600 3.48136900

O 0.86555000 -1.24880200 1.96319300

H 1.06084200 -0.26289800 2.04093700

**int17**

Rh -0.07268200 -0.83263600 0.15641000

C -0.04716000 -3.03752600 -0.02717600

C -1.47718700 -2.76183100 0.17044600

C 0.64519300 -2.67938200 1.17191100

C -1.63048000 -2.14331700 1.41285100

C -0.29608800 -1.98395800 2.00440800

C -1.32950400 1.96693200 -0.86070100

O -0.24186700 2.16195500 -1.51975700

C -1.12114500 1.51791000 0.58727700

C 0.14340600 1.30613200 1.10264600

H 0.18791100 1.15722700 2.18266000

C -2.25522100 1.59329100 1.58314900

H -2.70843600 2.58980100 1.53100300

H -3.05751800 0.88738200 1.38118300

H -1.88746800 1.43507700 2.60248200

N -2.50805600 2.15493300 -1.38123600

C 1.76340400 0.09344000 0.04985900

C 1.49551500 1.34192400 0.45164900

C 2.97251700 -0.57362500 -0.41218200

C 3.01035000 -1.28853300 -1.62392600

C 4.12492900 -0.56543000 0.39794400

C 4.16113400 -1.97677200 -2.00441100

H 2.12908200 -1.27407900 -2.25420100

C 5.27346500 -1.25254000 0.01263900

H 4.10449600 -0.00800700 1.32966500

C 5.29511300 -1.96789300 -1.18728800

H 4.17622000 -2.51745500 -2.94720500

H 6.15195100 -1.23390200 0.65191300

H 6.18924300 -2.50752400 -1.48605400

C 2.29931400 2.57182300 0.42731300

C 3.47541300 2.67853400 -0.33555500

C 1.88028500 3.68855200 1.16782600

C 4.21392300 3.85753200 -0.33626100

H 3.79865900 1.84048000 -0.94135500

C 2.62230400 4.86787100 1.16777900

H 0.95738900 3.63423100 1.73816500

C 3.79616300 4.95678900 0.41920700

H 5.11631600 3.92302800 -0.93815400

H 2.27826700 5.72023000 1.74728800

H 4.37434700 5.87648400 0.41385300

C -2.56053800 -3.15883300 -0.78141300

H -2.69172200 -4.24932700 -0.77914400

H -3.51673400 -2.70644100 -0.51016000

H -2.32900400 -2.86412000 -1.80870600

C -2.91247700 -1.74616800 2.07047100

H -3.34134300 -2.60438600 2.60558200

H -2.75735900 -0.95223200 2.80360900

H -3.65445900 -1.39345700 1.35037600

C 0.52088800 -3.80622500 -1.17788600

H 1.58507700 -3.59788000 -1.30814800

H 0.39997600 -4.88609500 -1.01544500

H 0.01159100 -3.55509500 -2.11246900

C 2.06241600 -3.00383000 1.51520200

H 2.51449700 -2.23326400 2.14371700

H 2.09623200 -3.95268900 2.06768900

H 2.68493200 -3.10793300 0.62516800

C -0.02122900 -1.47335200 3.38521700

H -0.69890000 -0.65814400 3.65209500

H -0.15319500 -2.27007000 4.12966300

H 1.00333000 -1.10103000 3.46984000

C -3.72909800 1.70506300 -0.92934400

C -4.77566600 2.61384400 -0.66317900

C -4.02716000 0.32491100 -0.84304500

C -6.03799700 2.16373800 -0.28798600

H -4.56642800 3.67563800 -0.75281500

C -5.29863300 -0.11691200 -0.48300800

H -3.23937100 -0.38745100 -1.06472100

C -6.31463500 0.79616000 -0.19092600

H -6.81960100 2.88902600 -0.07468200

H -5.50239100 -1.18554400 -0.43646800

H -7.30518300 0.45068700 0.08957400

C -0.90344000 -0.59611500 -2.95591900

H -1.89416100 -0.19286700 -2.72812900

H -0.94757800 -1.68692300 -3.02201700

H -0.57832900 -0.19980900 -3.92429900

O 0.05970600 -0.24373700 -1.96574000

H 0.06557500 0.81153700 -1.88270300

**int18**

Rh 1.63804800 -0.32204300 -0.32522700

C 2.86771600 -0.48771500 1.49300200

C 2.65426400 0.89491900 1.18307900

C 3.64013300 -1.05063100 0.40755800

C 3.37999200 1.19446300 -0.04809200

C 3.98031200 -0.00028900 -0.51588800

C -0.28875000 1.89493800 -1.24180500

O 0.33178500 2.87823800 -0.76137300

C 0.52811800 0.90541600 -2.08593200

C 0.17944900 -0.42991900 -2.13491500

H 0.48139400 -1.04895100 -2.98041100

C 1.43028100 1.50506500 -3.12808700

H 0.80781700 1.91111200 -3.93749700

H 1.98992700 2.34247600 -2.71411400

H 2.12011300 0.77085200 -3.55619200

N -1.56260400 1.53498900 -1.19099900

C -0.16990200 -0.84991500 0.14225600

C -0.73125200 -0.99676900 -1.11252900

C -2.48266000 2.12672600 -0.33783700

C -2.23317700 3.19502400 0.55842900

C -3.77226000 1.54543400 -0.32386000

C -3.22812800 3.62560000 1.43592600

H -1.25896100 3.66392500 0.54464600

C -4.75448000 1.98371400 0.55569700

H -3.96126000 0.72387800 -1.00824300

C -4.48981500 3.02675200 1.45004600

H -3.01237200 4.44510700 2.11848500

H -5.73209500 1.50711900 0.54980500

H -5.25592900 3.36968500 2.14031000

C -1.94623100 -1.70807700 -1.51657700

C -2.55400100 -1.34746400 -2.73368100

C -2.54279900 -2.70640300 -0.72745300

C -3.72335900 -1.97295300 -3.15324600

H -2.12982600 -0.52877000 -3.30487800

C -3.71277400 -3.33038500 -1.15137600

H -2.08992400 -2.99095000 0.21401300

C -4.30480900 -2.96897500 -2.36385500

H -4.19165000 -1.67370100 -4.08641800

H -4.16505000 -4.10045600 -0.53308100

H -5.22060800 -3.45503200 -2.68832300

C -0.73326800 -1.10601000 1.46717300

C -0.50362000 -2.32117300 2.13565800

C -1.47764100 -0.10380800 2.11357300

C -1.02184900 -2.53802400 3.41127900

H 0.08273100 -3.09232000 1.64254000

C -1.98476500 -0.32334000 3.39334200

H -1.65917300 0.83723900 1.60955800

C -1.76250300 -1.53809700 4.04621400

H -0.84373800 -3.48622000 3.91144000

H -2.56445300 0.46029300 3.87259900

H -2.16377200 -1.70602100 5.04165000

C 1.92487500 1.89727000 2.01930500

H 2.62818900 2.45209500 2.65645400

H 1.38918800 2.60319900 1.38116100

H 1.19458700 1.40614300 2.66805400

C 3.50917400 2.57008700 -0.61154800

H 2.52138000 3.03428600 -0.70347200

H 4.11873000 3.18513000 0.06473500

H 4.00068000 2.56508300 -1.58734700

C 4.81462600 -0.15918300 -1.75051800

H 4.57593200 0.60756100 -2.49230200

H 5.88591300 -0.07788200 -1.52263700

H 4.65398000 -1.13538500 -2.21887500

C 4.09344400 -2.47223300 0.31432300

H 4.19235600 -2.79397600 -0.72631600

H 5.07493100 -2.59448800 0.79347300

H 3.39415400 -3.14690700 0.81525900

C 2.49729300 -1.16921000 2.77100700

H 3.28084600 -1.00832500 3.52376800

H 1.55760000 -0.78648900 3.17356100

H 2.38034300 -2.24724300 2.63776500

**TS-S1**

Rh 2.03199800 0.09770700 0.07106000

C 2.44285800 -1.76656000 -1.03425100

C 2.17618600 -2.11018100 0.31870200

C 3.61121000 -0.88650600 -1.04472600

C 3.12673100 -1.40943200 1.16775400

C 4.05825200 -0.71923700 0.30593100

C 0.43359300 1.67782800 -2.06788000

O 1.56613100 1.30967100 -1.64897100

O -0.66014000 1.05278500 -1.90196400

C 1.79685900 2.90712800 1.01562100

O 0.66981000 2.99257900 0.53704000

O 2.59066800 1.86358000 0.98240000

C 0.37474300 2.98328500 -2.82362100

H -0.51542500 3.03731000 -3.45314100

H 0.33491100 3.77445300 -2.06633800

H 1.28080400 3.12380500 -3.41768300

C 2.44307200 4.08085500 1.75076200

H 3.37662300 4.36612900 1.25359500

H 1.75898300 4.93163300 1.76643800

H 2.69476600 3.78976200 2.77633500

C -0.83051300 -0.31800600 0.98424200

O 0.24928100 0.24977300 1.26845800

N -1.02924700 -0.76558900 -0.27591400

H -0.68241600 0.05882800 -1.13006900

C -1.85578300 -0.34807600 2.07772000

C -2.17046900 -1.47553700 2.71756300

H -2.87270300 -1.45909500 3.54713500

H -1.77184400 -2.43927000 2.42401800

C -2.40830300 1.01544300 2.40827400

H -1.59437900 1.73146900 2.56280200

H -3.01667600 1.39103100 1.57636800

H -3.03461700 0.97138700 3.30385200

S -2.33769600 -1.69790500 -0.79059100

O -2.11888800 -1.81359400 -2.23372400

O -2.43393500 -2.90864800 0.03475900

C -3.76852900 -0.67653100 -0.48933900

C -3.83515100 0.58242500 -1.09470700

C -4.77094200 -1.12790400 0.36375200

C -4.93234800 1.39367000 -0.82568100

H -3.03107700 0.92678100 -1.73727400

C -5.86442700 -0.30011200 0.61561400

H -4.67952400 -2.10312700 0.82724300

C -5.96158400 0.96717200 0.02920000

H -4.99043000 2.37857100 -1.28171100

H -6.65075700 -0.64131100 1.28366800

C -7.14845700 1.85882200 0.29684400

H -7.79607400 1.92611100 -0.58653200

H -7.75493000 1.48073400 1.12523000

H -6.83212300 2.87869600 0.54308700

C 3.21159000 -1.49254600 2.65837000

H 2.21916000 -1.61208600 3.10086500

H 3.82881100 -2.34474100 2.97294800

H 3.65099800 -0.58036600 3.07010400

C 5.17662700 0.14595100 0.78960900

H 4.76118100 1.05728900 1.23915300

H 5.76986500 -0.37952700 1.54532100

H 5.84024700 0.43864500 -0.02707900

C 4.22078600 -0.30593000 -2.27890800

H 4.99402100 0.42547800 -2.03457400

H 4.66961800 -1.09590300 -2.89359700

H 3.45663900 0.20401600 -2.87219900

C 1.68222300 -2.20540600 -2.24486400

H 1.69330800 -1.42155500 -3.00638100

H 2.12868100 -3.10884000 -2.68072500

H 0.63651400 -2.41529300 -2.00988900

C 1.10543400 -3.02342800 0.81790200

H 0.69669300 -2.67036100 1.76832900

H 0.28090200 -3.12467000 0.11274900

H 1.53283300 -4.01949700 0.99382400

**int-S2**

Rh 1.18716100 -0.33491000 -0.26816200

C 2.06062300 -1.94041300 0.91632100

C 2.65124100 -0.70559500 1.35180200

C 2.41466400 -2.14244800 -0.48318200

C 3.31338700 -0.11101400 0.22454600

C 3.17519000 -1.01884200 -0.90466400

C -0.61110900 -0.08286000 -2.05501700

O -0.63374300 -1.04183900 -1.21699400

O 0.44060800 0.63809400 -2.10884500

C -1.82852800 0.25682600 -2.85990800

H -2.38419200 1.00819400 -2.28588800

H -1.55173400 0.68902400 -3.82427700

H -2.46410100 -0.62127100 -2.98854600

C -0.36346400 2.25621500 0.02470300

O -1.49617300 2.64484800 -0.24772500

C 0.81561300 3.10536900 -0.35368700

C 1.78968800 3.30081700 0.54182900

H 2.59519000 4.00850700 0.36051200

H 1.77833800 2.78244400 1.49449700

C 0.71287900 3.83197400 -1.66458000

H 0.68667200 3.11178100 -2.48896300

H -0.22161900 4.40128400 -1.70077600

H 1.55512900 4.51582000 -1.80795600

N -0.06312600 1.08637700 0.72395600

S -1.22941000 0.53067800 1.79852500

O -0.63183000 -0.66947600 2.41869700

O -1.63043300 1.65106700 2.65601400

C -2.69417100 -0.04088700 0.93401900

C -2.83778900 -1.40036600 0.66650900

C -3.68299400 0.87226700 0.56612400

C -3.97809900 -1.84646300 0.00274600

H -2.06171600 -2.08997800 0.97256800

C -4.81514800 0.40949000 -0.09910900

H -3.54936100 1.92300900 0.78395300

C -4.97960100 -0.95148900 -0.39348100

H -4.09179200 -2.90607700 -0.21254500

H -5.58643900 1.11697600 -0.39352200

C -6.22473700 -1.44199000 -1.09089600

H -6.05200700 -2.39903900 -1.59377000

H -7.04438300 -1.59073100 -0.37551700

H -6.57491100 -0.72147900 -1.83793700

C 4.15128800 1.12527400 0.20761300

H 5.21381600 0.85388300 0.14766000

H 3.91322000 1.75673300 -0.65236600

H 4.00473700 1.72193100 1.10807000

C 2.54656300 -0.14181200 2.73019600

H 3.25077300 -0.65415700 3.39849100

H 2.77959400 0.92471200 2.74240400

H 1.53249800 -0.27366100 3.11412200

C 1.31896200 -2.91854800 1.76531900

H 2.02920200 -3.59458600 2.26093000

H 0.72355800 -2.40449700 2.51895400

H 0.64352800 -3.52838900 1.15922600

C 1.99397300 -3.31882400 -1.30447100

H 2.54866100 -4.21586000 -1.00193200

H 0.92670200 -3.51967800 -1.17430600

H 2.17504700 -3.14958900 -2.36819200

C 3.72351600 -0.75036000 -2.26864000

H 4.80597500 -0.93173500 -2.28557700

H 3.25851600 -1.38829500 -3.02297200

H 3.55219200 0.29068900 -2.55685000

**TS-S3**

Rh 1.35314900 -0.23679200 -0.20214600

C 2.51816900 -1.08721600 1.51472200

C 2.57481500 0.33390500 1.59572700

C 3.12951000 -1.48698800 0.25317900

C 3.15656800 0.83426400 0.36972500

C 3.56200500 -0.31283000 -0.42444700

C -0.69359200 -2.03063600 -1.47581300

O -0.68778700 -1.23123600 -2.46004300

O 0.01508300 -1.89976400 -0.43325500

C -1.62654000 -3.22045400 -1.55223500

H -1.44946100 -3.90615600 -0.72209100

H -2.65719300 -2.85472800 -1.51059100

H -1.49219700 -3.73469200 -2.50816100

C -0.55700800 1.98162900 -0.79118300

O -1.46106100 2.81232900 -0.75865000

C 0.41936300 1.90868400 -1.93765000

C 1.03989400 0.71735900 -2.13457700

H 1.77565500 0.63284200 -2.93692000

H 0.20786400 -0.27611200 -2.18712900

C 0.57397300 3.14677600 -2.76046700

H -0.41134400 3.51656400 -3.06312200

H 1.02818700 3.94744600 -2.16012800

H 1.19343600 2.97087200 -3.64404300

N -0.29073800 1.00486800 0.14147000

S -1.25255700 0.85509000 1.51080800

O -0.69246600 -0.28256500 2.25853200

O -1.38903300 2.15026500 2.18455400

C -2.84550300 0.34024000 0.89015500

C -3.12431300 -1.02455900 0.82052900

C -3.77726400 1.29473700 0.48130800

C -4.36159000 -1.43506600 0.32884100

H -2.38104100 -1.73886300 1.15529900

C -5.00603000 0.86217100 -0.01126800

H -3.52743500 2.34618300 0.54054100

C -5.31574700 -0.50203900 -0.09928600

H -4.59537400 -2.49612400 0.28540800

H -5.73968400 1.59746700 -0.33183500

C -6.64048400 -0.95427600 -0.66253200

H -6.88987100 -1.96870700 -0.33543200

H -7.45350400 -0.28725400 -0.35664100

H -6.61948300 -0.95766000 -1.76027900

C 4.29753000 -0.24913400 -1.72413600

H 4.02018500 0.63999600 -2.29647800

H 5.37966400 -0.20288600 -1.54552300

H 4.09587300 -1.12573700 -2.34495100

C 3.26938000 -2.90433100 -0.20461000

H 3.59765800 -2.95955800 -1.24534200

H 4.00192500 -3.44037100 0.41179500

H 2.31179100 -3.42795300 -0.12367800

C 1.95844400 -2.02110400 2.53799600

H 1.59654200 -2.93783900 2.06549000

H 2.72632000 -2.29782500 3.27291700

H 1.11551300 -1.56160000 3.05553100

C 2.09469000 1.18610000 2.72510600

H 1.38439000 0.64930400 3.35269400

H 2.94969900 1.49972100 3.33897200

H 1.59230000 2.08520200 2.36004400

C 3.44874800 2.27099200 0.07013900

H 2.65896800 2.91738400 0.46241500

H 4.39722500 2.58359000 0.52646400

H 3.51977700 2.44603400 -1.00630500

**TS-S4**

Rh 2.11483700 -0.00756700 0.09205300

C 3.32475300 -1.18243400 -1.36586800

C 1.97684600 -1.63717100 -1.41637800

C 3.82110500 -1.37123400 -0.01102100

C 1.61549600 -2.11828700 -0.08940300

C 2.77889600 -2.00331000 0.74995300

C 2.81703300 2.89673200 0.18045500

O 3.00286900 1.83523700 -0.49080400

C 3.33097800 4.19145300 -0.41070900

H 4.18162000 4.00260900 -1.06861500

H 2.52594600 4.63821100 -1.00554900

H 3.60027700 4.89206200 0.38229800

C -0.57708800 0.65485600 0.69153000

O 0.22987800 0.84064800 -0.30128900

C 0.04555800 0.46394600 2.04068800

C 1.40252300 0.57902100 2.07570500

H 1.92340400 0.34006800 3.00424500

C -0.83661200 0.11282700 3.19693700

H -1.58335000 0.89898600 3.35607600

H -1.40617600 -0.79862900 2.97837400

H -0.25648100 -0.02729600 4.11342000

N -1.88393200 0.54512900 0.61770100

C 2.87742300 -2.44579000 2.17402900

H 1.90779900 -2.39715500 2.67510000

H 3.22900800 -3.48460600 2.22044300

H 3.58279200 -1.82869800 2.73660500

C 5.21377400 -1.06239200 0.43986300

H 5.26760200 -0.95356000 1.52624500

H 5.90889700 -1.86055400 0.14672300

H 5.56541700 -0.12903400 -0.00884900

C 4.10783600 -0.53371900 -2.46169400

H 4.42906500 0.46799400 -2.15661100

H 5.00110400 -1.12406100 -2.69980100

H 3.51204900 -0.43226000 -3.37082000

C 1.03751400 -1.58509600 -2.57659000

H 1.42737600 -0.95319400 -3.37754400

H 0.88493600 -2.59472000 -2.98046800

H 0.06075400 -1.19221200 -2.27666800

C 0.29218000 -2.71283800 0.27743900

H -0.52165400 -2.23094500 -0.27224700

H 0.27363900 -3.78540300 0.04239700

H 0.09433000 -2.60166800 1.34709700

O 2.21420700 2.94992200 1.29265000

H 1.82773700 1.70153700 1.59173800

S -2.53543300 0.42443300 -0.91979700

O -2.46576000 1.67776900 -1.68390000

O -2.06184000 -0.83284300 -1.54813400

C -4.24856100 0.16103100 -0.47299600

C -5.14861800 1.21768900 -0.57315700

C -4.66541500 -1.10075900 -0.04913900

C -6.48415700 1.00632800 -0.23091400

H -4.80045400 2.18175800 -0.92675300

C -6.00053300 -1.29539400 0.29055900

H -3.95144800 -1.91579900 -0.00425400

C -6.92805400 -0.24568200 0.21021800

H -7.19362500 1.82597300 -0.31197600

H -6.33352900 -2.27771200 0.61698000

C -8.36758800 -0.46217100 0.60818200

H -9.02258200 0.29689800 0.16918500

H -8.72709600 -1.44718800 0.29098300

H -8.48715500 -0.40918400 1.69838100

**int-S5**

Rh 1.47301500 0.25593500 -0.27305200

C 2.35832800 -1.92111600 -0.42761400

C 2.47834800 -1.49458700 0.89358500

C 3.02318000 -0.92400700 -1.28597800

C 3.19946400 -0.20906700 0.88246500

C 3.64729200 0.05495200 -0.46180600

C -0.87580200 1.91973400 0.27949100

O -1.92916200 2.32153600 0.75118600

C 0.15454600 2.81614500 -0.34002000

C 1.31495900 2.22401600 -0.66820300

H 2.13951400 2.79621400 -1.09901100

C -0.20695800 4.26411200 -0.50410800

H -1.06559400 4.38001300 -1.17717000

H -0.50586600 4.69939800 0.45658700

H 0.63419400 4.83558900 -0.90833800

N -0.46645300 0.57838200 0.23895300

S -1.35078600 -0.60002700 1.04549800

O -0.75690700 -1.87717200 0.60733800

O -1.42625100 -0.31374100 2.48498000

C -2.99303600 -0.52813900 0.34709200

C -3.24483100 -1.21408700 -0.83986300

C -3.99293300 0.20210900 0.98803900

C -4.52189400 -1.15889100 -1.39469400

H -2.45235900 -1.78725800 -1.30792200

C -5.26388900 0.23917600 0.42338300

H -3.76243100 0.73734900 1.90010300

C -5.54737900 -0.43522300 -0.77330300

H -4.72719900 -1.68884400 -2.32126000

H -6.04933300 0.80797500 0.91459500

C -6.93571200 -0.39622200 -1.36319800

H -6.93603100 -0.72067700 -2.40841100

H -7.61737400 -1.05659100 -0.81143000

H -7.35890200 0.61346400 -1.31956300

C 1.63609100 -3.12329900 -0.94303600

H 2.34664100 -3.89211400 -1.27504900

H 0.98149000 -3.54489300 -0.18082900

H 1.00585000 -2.86178100 -1.79894700

C 1.93387900 -2.12814400 2.13254100

H 1.33046600 -1.41772300 2.70623400

H 1.28783300 -2.97286400 1.89660300

H 2.75520100 -2.47246100 2.77506500

C 3.57919000 0.55136400 2.11062700

H 4.47842500 0.11281400 2.56552400

H 3.79009000 1.59863300 1.88223400

H 2.77776400 0.51783600 2.85330100

C 3.13640100 -1.04267900 -2.77129700

H 3.38802300 -0.08517700 -3.23347400

H 3.92078500 -1.76480100 -3.03662700

H 2.19957700 -1.39843700 -3.20976000

C 4.54798600 1.16684400 -0.89846800

H 5.59885400 0.85217000 -0.86344900

H 4.32717400 1.48186100 -1.92222000

H 4.44047600 2.04208200 -0.25195500

**int-S6**

Rh 2.28818900 0.32894700 -0.45747800

C 4.35313100 -0.09281400 0.29570600

C 3.80906200 -1.42110500 -0.04093600

C 3.55011600 0.47977300 1.31315300

C 2.64561500 -1.62872200 0.70459200

C 2.40622900 -0.41132400 1.49307700

C -0.41030600 0.90845800 -0.94252800

O 0.41316100 -0.08906700 -1.12112300

C 0.21669000 2.25095400 -0.81535100

C 1.53864600 2.18954300 -0.54195600

H 2.11705000 3.09014400 -0.32518700

C -0.62839900 3.48822900 -0.90564900

H -1.07769600 3.58470700 -1.90171200

H -1.45907500 3.43519100 -0.19284300

H -0.03510100 4.38523800 -0.70201000

N -1.70187800 0.77605000 -0.78143500

S -2.24386900 -0.80473800 -0.57571200

O -2.27837500 -1.55860700 -1.83749700

O -1.59741900 -1.40862700 0.61305800

C -3.93693000 -0.45324600 -0.11241400

C -4.93222700 -0.48739300 -1.08571500

C -4.23950500 -0.15391500 1.21511000

C -6.24782300 -0.20645600 -0.72044000

H -4.67124100 -0.73770800 -2.10800300

C -5.55799600 0.12548200 1.56387500

H -3.44898300 -0.15019900 1.95735500

C -6.58013900 0.10326100 0.60434200

H -7.02996000 -0.22982900 -1.47516600

H -5.80130400 0.36182300 2.59696400

C -8.01127300 0.37520600 0.99901400

H -8.59324500 0.75832700 0.15459800

H -8.50517200 -0.54146800 1.34769400

H -8.06992900 1.10526400 1.81326600

C 1.68142200 -2.77048700 0.64640000

H 1.95110100 -3.48297100 -0.13655100

H 1.66315900 -3.30678300 1.60372400

H 0.66528300 -2.41091400 0.44567300

C 1.27779000 -0.24802100 2.45875500

H 1.48978500 -0.80452700 3.38247000

H 1.12904400 0.80228700 2.72018700

H 0.34633800 -0.63332900 2.03267200

C 3.79567500 1.75052000 2.06268800

H 4.31985300 1.54629100 3.00527700

H 4.40822500 2.44489600 1.48142400

H 2.85576100 2.25385900 2.30296300

C 5.62872700 0.45767400 -0.25948200

H 6.49617000 -0.00363700 0.23190800

H 5.71424100 0.25645600 -1.33167400

H 5.69534100 1.53855200 -0.11276700

C 4.41515200 -2.33222600 -1.06072700

H 5.36586100 -2.74427600 -0.69750600

H 3.75159400 -3.16705200 -1.29557300

H 4.62858200 -1.79942900 -1.99385200

**int-S7**

Rh 1.20646500 -0.62514900 -0.10605000

C 2.65759900 -0.98727100 -1.84125400

C 1.55228500 -1.84298100 -2.16627700

C 3.22612400 -1.41611400 -0.58875200

C 1.40017900 -2.76824700 -1.09752400

C 2.41957000 -2.50949500 -0.10869300

C -0.61386400 -0.82370700 2.26941600

O -1.56071500 -1.05498000 3.01732400

C 0.73656500 -0.44772800 2.76615900

C 1.68907300 -0.31617300 1.83977000

H 2.71610600 -0.04687300 2.07909400

C 0.91019700 -0.27809300 4.24948900

H 0.24454700 0.50646600 4.62981900

H 0.63665200 -1.19804100 4.77850900

H 1.94358600 -0.01771000 4.49992200

N -0.64182300 -0.87383800 0.88202200

S -1.94148400 -1.60743200 0.12104700

O -1.66128700 -1.48422600 -1.32271100

O -2.16634200 -2.94883300 0.68030200

C -3.40258700 -0.62424400 0.44911400

C -3.84715600 0.25641400 -0.53355500

C -4.11317600 -0.79721700 1.63942500

C -5.00689300 0.99567000 -0.30746200

H -3.30273600 0.34604900 -1.46335400

C -5.26874200 -0.05047800 1.84505300

H -3.74754800 -1.48982000 2.38450500

C -5.72958500 0.86041100 0.88258900

H -5.35305100 1.68513900 -1.07274800

H -5.82668500 -0.17824500 2.76948100

C -6.96823500 1.68385500 1.13714000

H -7.37120700 2.09922800 0.20791800

H -7.75444300 1.08708900 1.61277200

H -6.75169900 2.52531700 1.80862300

C 0.38712200 1.32057300 -0.75072200

C 1.40001300 1.58550600 -0.06006400

C -0.78152400 1.75117900 -1.47305000

C -1.56586500 2.78799100 -0.93671100

C -1.11217900 1.21953400 -2.72782700

C -2.64347800 3.29597800 -1.65655700

H -1.31928500 3.18215200 0.04351800

C -2.18836800 1.73480300 -3.44532900

H -0.52879600 0.39993700 -3.12112300

C -2.95491400 2.77610300 -2.91493600

H -3.24476300 4.09393700 -1.23109500

H -2.43575900 1.31596700 -4.41639300

H -3.79656500 3.17279700 -3.47549700

C 2.46123600 2.37798200 0.50422500

C 3.47174900 2.88856400 -0.33174400

C 2.49990700 2.67404500 1.87918800

C 4.50470000 3.65792300 0.19864500

H 3.42587800 2.68879000 -1.39618400

C 3.53505000 3.44600600 2.40111900

H 1.71529300 2.29296900 2.52121000

C 4.54374000 3.93363900 1.56719000

H 5.27666100 4.04744400 -0.45887300

H 3.55206500 3.66942600 3.46392500

H 5.35055300 4.53244700 1.97953400

C 0.75124900 -1.83968000 -3.43107600

H 0.98184300 -2.72422200 -4.03952200

H -0.31858000 -1.85161900 -3.20673700

H 0.97552000 -0.95905700 -4.03982300

C 0.42383000 -3.89723500 -1.04303200

H -0.40118600 -3.74060400 -1.73757300

H 0.93589000 -4.83419800 -1.30377000

H -0.01229100 -4.01285600 -0.04874900

C 3.19424800 0.09756700 -2.72156300

H 3.66057400 -0.33310000 -3.61667600

H 2.39992100 0.77441100 -3.05206100

H 3.95159200 0.68862900 -2.20347100

C 4.47708800 -0.88325100 0.03996100

H 5.36809600 -1.29000600 -0.45539200

H 4.52612200 0.20838300 -0.01861700

H 4.53430300 -1.16004500 1.09574800

C 2.65526000 -3.34591600 1.11051800

H 1.70544400 -3.64981000 1.55904400

H 3.21697300 -4.25521300 0.85825000

H 3.21800700 -2.79884400 1.87053700

**int-S8**

Rh -1.43398000 0.27390800 -0.66189200

C -3.14578700 0.10453100 -2.13148500

C -2.44959400 -1.17425500 -2.19659700

C -2.24612100 1.12848500 -2.57715000

C -1.11876300 -0.92822700 -2.59026900

C -0.96363700 0.51370800 -2.78528900

C 1.18819400 0.96699800 0.28162100

O 0.51764100 -0.07960600 -0.10401800

C 0.39160100 2.18613700 0.50275400

C -0.89709400 2.06245200 0.13466300

H -1.60232200 2.88655100 0.23207500

C 1.04413900 3.42225200 1.05596200

H 1.45567000 3.23086600 2.05401500

H 1.88502300 3.73158200 0.42480000

H 0.32766700 4.24700400 1.12532200

N 2.49396400 0.96976200 0.43449100

S 3.26216800 -0.43275600 -0.05503200

O 2.96632700 -1.57600300 0.83281500

O 3.12616500 -0.62546500 -1.51773400

C 4.95620900 0.05045500 0.25658000

C 5.46076600 -0.02828400 1.55499800

C 5.75055100 0.49201800 -0.79679300

C 6.77650500 0.35465300 1.79454500

H 4.82688700 -0.39056400 2.35694100

C 7.06917300 0.86870700 -0.54094900

H 5.33440800 0.53059000 -1.79725100

C 7.59986700 0.80777200 0.75252800

H 7.17572500 0.29919500 2.80450700

H 7.69616800 1.21432800 -1.35904600

C 9.03270000 1.19641500 1.02449800

H 9.11314200 1.82417900 1.91922600

H 9.65748200 0.31002500 1.19506600

H 9.46423100 1.74762700 0.18326600

C -1.94622700 -0.99761000 1.10699400

C -2.59728300 0.06630500 1.21423700

C -1.51624400 -2.36082900 1.23776100

C -0.17864800 -2.75011500 1.03896300

C -2.48339400 -3.33337500 1.56541500

C 0.17230000 -4.09214200 1.16017300

H 0.57160200 -2.01025500 0.79181500

C -2.11762200 -4.67091900 1.68347200

H -3.51366500 -3.02644800 1.71954300

C -0.78918400 -5.05456100 1.47771100

H 1.20974000 -4.37389800 1.00809300

H -2.86947600 -5.41424000 1.93349300

H -0.50644600 -6.09964400 1.56874200

C -3.47988900 0.99449700 1.86817800

C -3.81571900 0.78712900 3.21962000

C -4.04262500 2.08580400 1.18768100

C -4.69355800 1.65283300 3.86529900

H -3.37630300 -0.05227800 3.74926000

C -4.92270200 2.94686000 1.83736400

H -3.77955500 2.24014600 0.14839200

C -5.25045700 2.73482800 3.17804800

H -4.94183800 1.48493300 4.90933400

H -5.35161300 3.78642900 1.29760600

H -5.93341100 3.40946500 3.68592300

C -3.07005000 -2.49373000 -1.86265000

H -3.74710800 -2.40474800 -1.00804400

H -3.65385600 -2.87606400 -2.71035100

H -2.31509300 -3.23928300 -1.60473600

C -4.60889700 0.24777100 -1.84403100

H -5.20277800 -0.13440200 -2.68481500

H -4.89603500 -0.31653300 -0.95183100

H -4.89158900 1.29021200 -1.68289400

C -2.56611100 2.57581700 -2.78431700

H -2.79302300 2.77294700 -3.83994400

H -3.43482500 2.88513500 -2.19637300

H -1.72381200 3.21175500 -2.49854900

C 0.29715900 1.17441100 -3.24816500

H 0.40500200 1.08517200 -4.33736300

H 0.30186900 2.23725900 -2.99363800

H 1.17235600 0.71014600 -2.78370900

C 0.00088700 -1.91105400 -2.71737800

H 0.24397400 -2.07922600 -3.77468200

H 0.90430700 -1.54040300 -2.22172900

H -0.25958200 -2.87352100 -2.27070100

**TS-S9**

Rh -1.23092900 0.76556300 -0.08403600

C -2.64936700 0.99813900 -1.84858500

C -1.68752200 2.04011300 -1.97441600

C -3.34443600 1.17788300 -0.59325000

C -1.77576200 2.85977400 -0.78146400

C -2.83672600 2.36974300 0.03880300

C 0.66419400 0.88558400 2.26130400

O 1.61812300 1.09270600 3.01126700

C -0.62651000 0.33096000 2.74983100

C -1.53255700 -0.06283200 1.82577100

H -2.52356600 -0.39606700 2.12211400

C -0.82400000 0.26579700 4.23488800

H -0.01542700 -0.31475300 4.69545500

H -0.76985700 1.26693200 4.67955500

H -1.78695400 -0.18525100 4.49441700

N 0.61852700 1.12366600 0.90133500

S 1.92407500 1.86413200 0.15396300

O 1.59919700 1.85391500 -1.28531500

O 2.21011800 3.15242700 0.79709700

C 3.33608900 0.78578700 0.37261500

C 3.64984700 -0.12419400 -0.63533900

C 4.11848100 0.88451900 1.52543700

C 4.74762000 -0.96663800 -0.47096900

H 3.05466000 -0.15982100 -1.53702400

C 5.21363800 0.03810700 1.66754400

H 3.85459900 1.60102000 2.29100200

C 5.53967700 -0.90444000 0.68095900

H 4.98913300 -1.67948300 -1.25488900

H 5.82792100 0.10948300 2.56186000

C 6.71110300 -1.83681800 0.86909700

H 7.00092100 -2.31032300 -0.07424300

H 7.58419900 -1.30735000 1.26680700

H 6.46810300 -2.63712700 1.58043200

C -0.29582600 -1.03746100 -0.48059300

C -1.06782700 -1.41900400 0.48720800

C 0.62761800 -1.54420000 -1.45401600

C 1.31585800 -2.74776200 -1.19650000

C 0.86686800 -0.86861300 -2.66290400

C 2.20925500 -3.25976400 -2.13085900

H 1.14933400 -3.25812000 -0.25329700

C 1.75371000 -1.39309100 -3.59875000

H 0.38596500 0.08399500 -2.82933600

C 2.43027900 -2.58772100 -3.33724200

H 2.74064500 -4.18260700 -1.91612300

H 1.93414200 -0.85667700 -4.52591600

H 3.13184900 -2.98792300 -4.06348400

C -1.92026500 -2.56792000 0.78339900

C -2.14153900 -3.02745400 2.09162800

C -2.50543600 -3.26422700 -0.29100600

C -2.93259200 -4.15195900 2.31890900

H -1.67319100 -2.50896900 2.92093300

C -3.29648200 -4.38634700 -0.05932600

H -2.32046600 -2.91543300 -1.30195800

C -3.51653800 -4.83026700 1.24704800

H -3.08969200 -4.50225900 3.33498400

H -3.74283200 -4.91378600 -0.89754100

H -4.13624100 -5.70374700 1.42805900

C -3.31104600 2.96242100 1.32948700

H -3.61866600 2.18794400 2.03872800

H -2.52016900 3.54824900 1.80534400

H -4.16969700 3.62780300 1.16891200

C -0.93748000 4.07160800 -0.52611700

H -1.41150800 4.95790600 -0.96960900

H -0.80662400 4.25476100 0.54268800

H 0.05562900 3.95597400 -0.96244700

C -0.83832400 2.35991900 -3.16442900

H -1.18942200 3.28409300 -3.64284800

H 0.20399700 2.49930300 -2.87009500

H -0.88871700 1.56576600 -3.91457500

C -2.92714700 -0.08859000 -2.84158200

H -2.02512400 -0.36677600 -3.39216500

H -3.30366900 -0.98712000 -2.34509600

H -3.68569700 0.22891500 -3.56904400

C -4.47150200 0.32906800 -0.08972600

H -5.42609100 0.64574700 -0.52992300

H -4.32292800 -0.72671900 -0.33511400

H -4.56926900 0.40719500 0.99675900

**TS-S10**

Rh -1.54006000 -0.69402900 -0.62961300

C -3.00473900 -2.30586000 0.01346900

C -1.72718200 -2.86303500 -0.26773000

C -3.46605000 -1.64877000 -1.18775400

C -1.44172700 -2.65609200 -1.69330900

C -2.52672500 -1.94393000 -2.25761100

C 1.17625600 0.33145100 -0.80314500

O 0.50112600 -0.57381600 -0.16985000

C 0.40565300 1.17305500 -1.73938800

C -0.94925600 1.09413900 -1.62304300

H -1.58299100 1.62624400 -2.32786300

C 1.13105300 2.03068400 -2.73408500

H 1.80587600 2.72101900 -2.21312800

H 1.76555200 1.42209900 -3.38884400

H 0.43227800 2.60621200 -3.34924800

N 2.47300100 0.51589200 -0.67184400

S 3.22965900 -0.60103500 0.32374800

O 2.83995400 -0.43057300 1.73829200

O 3.18186400 -1.95377200 -0.26907100

C 4.91503700 -0.01506400 0.17689800

C 5.38382100 0.94732300 1.06889900

C 5.74008200 -0.53393700 -0.81837700

C 6.69547600 1.40106900 0.95010200

H 4.72571100 1.32143300 1.84532300

C 7.04979700 -0.07038500 -0.92418000

H 5.35501900 -1.29514400 -1.48788500

C 7.54659000 0.90059500 -0.04441800

H 7.06748700 2.15266600 1.64220100

H 7.69928500 -0.47070900 -1.69890600

C 8.97714100 1.37175600 -0.14310400

H 9.34642800 1.31834200 -1.17255700

H 9.08303800 2.40494200 0.20397000

H 9.64038100 0.75109800 0.47400800

C -1.57771800 0.58558200 0.98698100

C -1.74788500 1.46804900 0.06058500

C -1.52912500 0.20882900 2.37848200

C -2.61540600 0.47585000 3.23122900

C -0.40392900 -0.47643400 2.87821800

C -2.58408900 0.05421300 4.55905100

H -3.47657000 1.01236300 2.84495000

C -0.38250800 -0.88509400 4.20940800

H 0.44328200 -0.66200700 2.22587000

C -1.47009800 -0.63033700 5.04994200

H -3.42911900 0.26113100 5.20995000

H 0.49371400 -1.40222800 4.58927200

H -1.44776500 -0.95869500 6.08535800

C -2.47385100 2.72461200 -0.13465500

C -3.81282100 2.81220200 0.28206200

C -1.85870900 3.85237700 -0.69921600

C -4.51848000 4.00521200 0.14319000

H -4.28913400 1.93258200 0.70415300

C -2.56469200 5.04664000 -0.82838100

H -0.82294300 3.78646000 -1.01460100

C -3.89554400 5.12540300 -0.41188500

H -5.55369500 4.06124300 0.46760300

H -2.07511600 5.91800200 -1.25360100

H -4.44503800 6.05623000 -0.51867300

C -0.18493100 -3.11619600 -2.36352400

H 0.69579500 -2.81799300 -1.78448700

H -0.17178500 -4.20984900 -2.45609100

H -0.08929700 -2.69226200 -3.36635400

C -0.80574900 -3.56506100 0.68043800

H -0.86854800 -4.65390500 0.55369000

H 0.23089600 -3.26091400 0.50641200

H -1.04930900 -3.32683600 1.71874900

C -3.71113900 -2.32342400 1.33237100

H -4.38201900 -3.18958200 1.40081500

H -3.00501700 -2.37379700 2.16431400

H -4.31542400 -1.42308100 1.47383500

C -4.77718400 -0.94407600 -1.35367000

H -5.56669600 -1.64403300 -1.65905300

H -5.09493500 -0.47369700 -0.41892800

H -4.71437600 -0.16186100 -2.11556800

C -2.67687500 -1.50723700 -3.68195500

H -3.32450800 -2.19683000 -4.23911600

H -3.12382700 -0.51043400 -3.74642000

H -1.70998200 -1.47096200 -4.18997000

**TS-S11**

Rh 1.34277600 -0.15135300 -0.06410700

C 1.31673900 -1.87289600 -1.43590800

C 0.90990400 -2.32025300 -0.15299900

C 2.67106900 -1.33322300 -1.31874100

C 1.98298300 -2.02894600 0.78933200

C 3.09570600 -1.50007700 0.03916200

C 0.07971800 2.06632000 -1.86138900

O 1.04430000 1.28493400 -1.64971700

O -1.14049900 1.85250400 -1.55781800

C 1.92160700 2.52058200 1.11670900

O 0.86374700 2.96907300 0.68848100

O 2.39581500 1.31103100 0.93557300

C 0.37955200 3.38041900 -2.53705800

H -0.49835800 3.76741700 -3.05785500

H 0.65801800 4.07530800 -1.73690100

H 1.22706700 3.27826600 -3.21850700

C 2.86555900 3.37237300 1.96522800

H 3.86064200 3.40570000 1.50868800

H 2.46742700 4.38454200 2.06008300

H 2.97669100 2.92644200 2.95977000

C -1.44881800 0.02199400 1.14678600

O -0.20463000 0.21201100 1.35430400

N -1.97217900 0.06304700 -0.06536000

H -1.37631200 0.92606500 -0.90089100

C -2.21812200 -0.30192000 2.39894500

C -3.07411100 -1.32833600 2.43970800

H -3.57372500 -1.60220000 3.36543700

H -3.31688800 -1.91463600 1.56016300

C -1.86781200 0.53516600 3.60063000

H -0.79390400 0.48132600 3.80315000

H -2.09812400 1.59221100 3.41652000

H -2.42302500 0.20605100 4.48397100

C 1.95064100 -2.32335300 2.25503800

H 0.99564200 -2.00542300 2.68349400

H 2.07697600 -3.39628000 2.44997700

H 2.74304300 -1.78243200 2.77775800

C 4.38270500 -1.02847900 0.63429000

H 4.22509700 -0.04611500 1.09969200

H 4.73682800 -1.72683000 1.39866400

H 5.15998900 -0.92843300 -0.12713800

C 3.45279200 -0.75264100 -2.45325500

H 4.38629500 -0.30609600 -2.10352600

H 3.69463500 -1.52147000 -3.19779100

H 2.87200100 0.03472700 -2.94366700

C 0.51830300 -1.90358100 -2.69899200

H 0.69347200 -0.99608500 -3.28269700

H 0.79600700 -2.76761300 -3.31661200

H -0.55179400 -1.96233800 -2.48672700

C -0.38932400 -2.96518500 0.20447000

H -0.68539300 -2.71798100 1.22623700

H -1.19182900 -2.64321400 -0.46097200

H -0.29643300 -4.05699800 0.13454000

C -3.36399300 -0.01085800 -0.32157600

C -3.82846200 -0.95199600 -1.24847500

C -4.27537500 0.88973100 0.24810800

C -5.18315100 -1.01653400 -1.57498800

H -3.11477500 -1.63036800 -1.70789600

C -5.62738600 0.82289100 -0.07907900

H -3.91019900 1.64029900 0.94194000

C -6.08944400 -0.13279600 -0.98768400

H -5.52922300 -1.75645800 -2.29173000

H -6.32288500 1.52568800 0.37165200

H -7.14417500 -0.18036700 -1.24265700

**int-S12**

Rh -0.89100900 0.24279800 0.11774200

C -1.88970800 -1.36462700 -0.96807000

C -1.94071500 -0.15909500 -1.77585100

C -2.65456200 -1.11817400 0.23232400

C -2.65207600 0.83283400 -1.04150600

C -3.09523900 0.23922700 0.21202600

C 0.46694200 -1.49423400 2.09795500

O 0.11356600 -0.26372000 1.86786700

O 0.20236200 -2.47985800 1.40485600

C 1.31125700 -1.65554900 3.35984800

H 0.93266800 -2.49733700 3.94733700

H 2.33626400 -1.90104900 3.05921800

H 1.32220200 -0.74747000 3.96631700

C 1.09837400 1.86054400 0.12846300

O -0.04036000 2.21700300 0.59398900

C 2.27934900 2.73997200 0.34531600

C 3.27484400 2.78373700 -0.54826200

H 4.12274700 3.44898100 -0.40952600

H 3.27740500 2.16104800 -1.43625900

C 2.22669100 3.59614300 1.58287400

H 1.33496800 4.23112100 1.57362200

H 2.15534000 2.97174500 2.48159900

H 3.11668900 4.22738600 1.66048000

N 1.07924200 0.71238900 -0.54403400

C -2.91312900 -2.11802500 1.31193100

H -3.74815500 -2.76999500 1.02230000

H -2.02452500 -2.72721400 1.48313600

H -3.17960300 -1.62651300 2.25089300

C -3.91483800 0.94020200 1.24854300

H -4.98086000 0.91657700 0.98436100

H -3.80092000 0.47266200 2.22945600

H -3.61737700 1.98880700 1.34085400

C -1.26832800 -2.66627900 -1.35783400

H -0.84673700 -3.15282500 -0.47762900

H -2.01132300 -3.32544200 -1.82733200

H -0.45179300 -2.51459700 -2.06936300

C -1.33497000 0.00989700 -3.13258200

H -0.52077700 -0.70065500 -3.28718600

H -2.08650300 -0.16784000 -3.91262500

H -0.93300800 1.01706700 -3.26910500

C -2.91443500 2.24614500 -1.45535800

H -3.96960300 2.37856200 -1.72725200

H -2.68453300 2.93943300 -0.64086800

H -2.30463100 2.52914200 -2.31640700

C 2.15575300 -0.16814500 -0.73144200

C 3.03333800 -0.49273400 0.31614700

C 2.31789500 -0.79205100 -1.97592800

C 4.05533500 -1.41450900 0.11212700

H 2.88670100 -0.02761100 1.28408300

C 3.34063500 -1.71861000 -2.17283000

H 1.64050600 -0.52909600 -2.78183100

C 4.21420700 -2.03380900 -1.13071500

H 4.72595600 -1.66029600 0.93118600

H 3.45652900 -2.19351600 -3.14335000

H 5.00883200 -2.75828900 -1.28302300

**TS-S13**

Rh -0.72071200 -0.10286100 0.07321700

C -0.84898600 -1.94026900 -1.22646000

C -0.33183100 -0.87989800 -2.01475500

C -2.18781800 -1.56193100 -0.77858700

C -1.29858900 0.20044000 -2.00148400

C -2.47981200 -0.27538100 -1.30139300

C -0.40504300 -0.57372600 3.03028900

O -0.69781400 0.64938000 3.18961600

O -0.40265300 -1.17646300 1.91488800

C 0.00681600 -1.36675200 4.25175200

H -0.44288500 -0.94642500 5.15321300

H -0.26648100 -2.41767900 4.13476100

H 1.09729500 -1.30493200 4.34755200

C 0.95823800 2.28328200 0.33001900

O 1.85389100 3.12510500 0.19440500

C -0.47102900 2.71662500 0.53785700

C -1.36702000 1.76590400 0.91404100

H -2.42367100 2.03216400 0.99239300

H -0.98684000 1.14880200 2.00311400

C -0.76046600 4.16446500 0.30973300

H -0.09633700 4.77915500 0.92690200

H -0.53236200 4.43433400 -0.73022400

H -1.80343600 4.41015500 0.52751000

N 1.08525400 0.92189300 0.32535200

C -3.75847300 0.48516000 -1.15238300

H -3.57467400 1.55973200 -1.07098200

H -4.40248500 0.32409200 -2.02674800

H -4.31310400 0.17107100 -0.26437400

C -3.07082100 -2.43138900 0.06003500

H -3.93638500 -1.87994800 0.43542700

H -3.44011500 -3.28878300 -0.51748000

H -2.51985700 -2.81889700 0.92271900

C -0.17523000 -3.23388700 -0.89638800

H -0.36227200 -3.50951500 0.14601100

H -0.54867100 -4.04700800 -1.53287700

H 0.90614900 -3.16109800 -1.03202900

C 0.98939300 -0.84402900 -2.71163400

H 1.71701800 -1.50508200 -2.23738000

H 0.86717200 -1.15590000 -3.75738600

H 1.41623800 0.16126500 -2.70683600

C -1.17403100 1.48859500 -2.75392100

H -0.14142700 1.84868100 -2.74274400

H -1.47730600 1.36770300 -3.80236000

H -1.80023900 2.26703600 -2.31041800

C 2.30099900 0.26705200 0.06686500

C 3.36667700 0.85322100 -0.64690900

C 2.43379200 -1.07416000 0.48127300

C 4.50159700 0.10347700 -0.95397400

H 3.29538100 1.89131800 -0.93826200

C 3.57212100 -1.81072000 0.16616700

H 1.62689000 -1.52393800 1.04748400

C 4.61493900 -1.23109900 -0.56180700

H 5.30744100 0.57425800 -1.51166600

H 3.64676000 -2.84347300 0.49859800

H 5.50352900 -1.80564500 -0.80741800

**TS-S14**

Rh 1.14917300 0.03640800 0.00293300

C 2.11964200 -1.08892000 -1.69197200

C 0.91618200 -1.74802200 -1.33148800

C 2.99202100 -1.06615600 -0.52316700

C 1.00862700 -2.12177200 0.07196000

C 2.32710300 -1.75852300 0.53627400

C 1.46044100 3.01993200 -0.09513900

O 1.65098400 1.96457500 -0.77031600

C 1.69708300 4.34565200 -0.78872800

H 2.38071400 4.22273900 -1.63098900

H 0.73683900 4.71296900 -1.16890400

H 2.08335500 5.08116700 -0.07957500

C -1.46309400 0.39425000 1.09252200

O -0.85744400 0.58582800 -0.06245100

C -0.56657100 0.33843800 2.27738200

C 0.75333200 0.60941800 2.03467000

H 1.47298700 0.47251200 2.84428100

C -1.14226000 -0.02048400 3.61117300

H -1.93652700 0.68281400 3.88219700

H -1.62101900 -1.00601900 3.56339800

H -0.37411300 -0.02339900 4.39008600

N -2.73268700 0.19599700 1.29168500

C 2.88096900 -2.04358200 1.89528900

H 2.09579500 -2.03082200 2.65527900

H 3.35025900 -3.03574400 1.91363900

H 3.63839700 -1.30914100 2.18081800

C 4.37476600 -0.49403700 -0.50700300

H 4.71800700 -0.30871000 0.51406100

H 5.08977700 -1.17757200 -0.98415400

H 4.40658600 0.45600600 -1.04854800

C 2.45967200 -0.44687700 -2.99915800

H 2.73192600 0.60295900 -2.84668900

H 3.30869300 -0.95357300 -3.47495300

H 1.61434900 -0.47442500 -3.69017300

C -0.31133900 -1.94572700 -2.16336800

H -0.17373800 -1.57115400 -3.18022000

H -0.56768100 -3.01022700 -2.22289000

H -1.16186600 -1.41498000 -1.72021300

C -0.04059600 -2.87765800 0.82575000

H -1.03710000 -2.48220900 0.60759900

H -0.03190600 -3.94164600 0.55503400

H 0.11622800 -2.80463400 1.90475300

C -3.67819500 0.14354400 0.26769800

C -3.49786500 0.55257000 -1.07436600

C -4.94118000 -0.36482400 0.63964500

C -4.54220300 0.43412600 -1.99110900

H -2.54439100 0.96291200 -1.37550400

C -5.97299700 -0.48658800 -0.28344300

H -5.07604400 -0.66275800 1.67496700

C -5.77911300 -0.08847500 -1.60989800

H -4.38505700 0.75719700 -3.01758100

H -6.93331900 -0.88784100 0.03041200

H -6.58436300 -0.17867900 -2.33388800

O 1.06566000 3.05014400 1.10936100

H 0.89906100 1.78986100 1.51009900

**int-S15**

Rh 0.72730100 0.24148900 -0.24775100

C 1.19549800 -2.06687100 -0.41238200

C 0.69207800 -1.79970600 0.86321900

C 2.38457500 -1.22572900 -0.62345300

C 1.53130400 -0.75008600 1.45560100

C 2.64659200 -0.50320200 0.56694200

C -1.25864500 2.35165300 -0.34652500

O -2.32951100 2.94710000 -0.32039000

C 0.07348400 3.04102300 -0.30315400

C 1.13114100 2.21922500 -0.23256800

H 2.15306400 2.60060100 -0.16857400

C 0.08069200 4.54196900 -0.33234600

H -0.36255400 4.91778200 -1.26301600

H -0.52547300 4.95148300 0.48446900

H 1.09999900 4.93201500 -0.24876100

N -1.12519300 0.96374500 -0.43135800

C 0.64690300 -3.01507900 -1.43162800

H 1.26792000 -3.91810000 -1.50125400

H -0.37216600 -3.32019800 -1.18487700

H 0.62654000 -2.55757700 -2.42642600

C -0.52660800 -2.36586800 1.51910100

H -1.20544900 -1.57043900 1.84559100

H -1.09182600 -3.00388400 0.83805700

H -0.25285300 -2.95798000 2.40184100

C 1.37551000 -0.22817900 2.84759700

H 1.83520300 -0.91747100 3.56980400

H 1.85141000 0.74873500 2.96172700

H 0.31908900 -0.12338900 3.10941100

C 3.22977700 -1.25840900 -1.85697700

H 3.86390900 -0.37143500 -1.92989100

H 3.88333700 -2.14181100 -1.85678000

H 2.61299000 -1.30607600 -2.75952400

C 3.81626300 0.38625900 0.85012100

H 4.61659100 -0.16823700 1.35698800

H 4.23093100 0.80574100 -0.07079900

H 3.53067300 1.22293800 1.49384700

C -2.23829700 0.11961500 -0.27006000

C -2.35033600 -1.00687800 -1.10184600

C -3.19706600 0.30869000 0.74127800

C -3.37235500 -1.93473900 -0.91587200

H -1.62137900 -1.13228300 -1.89673000

C -4.22079300 -0.62067200 0.91930700

H -3.13211200 1.18670200 1.37267200

C -4.31251300 -1.74933000 0.10071700

H -3.44237800 -2.79777900 -1.57340400

H -4.95216500 -0.46227800 1.70754500

H -5.11263200 -2.46918700 0.24705700

**int-S16**

Rh 1.09824100 0.22528800 -0.13815300

C 3.10589700 -0.29611100 -0.86640700

C 2.44462900 -1.60236800 -0.72124400

C 3.20451800 0.30774700 0.41792100

C 2.06781100 -1.75520500 0.61628800

C 2.45694300 -0.53492500 1.32698400

C -1.58806900 1.14941700 -0.09770300

O -0.91245400 0.00606200 -0.09683100

C -0.75078800 2.37044300 -0.11031000

C 0.58513600 2.13839400 -0.09016200

H 1.30210300 2.96199400 -0.05676900

C -1.39842500 3.72565200 -0.13904000

H -2.03443600 3.83375700 -1.02483000

H -2.05365600 3.86168300 0.72868100

H -0.64388700 4.51928300 -0.14443900

N -2.87870100 1.28010700 -0.08720900

C 1.30774400 -2.88300000 1.24156600

H 0.97023800 -3.60245200 0.49196700

H 1.92859800 -3.42042600 1.96986600

H 0.42155300 -2.51308300 1.76791800

C 2.29566500 -0.32302000 2.79744400

H 3.11098200 -0.81786900 3.34408500

H 2.31396000 0.73917600 3.05217600

H 1.35109600 -0.74181800 3.15447100

C 3.92158800 1.57247200 0.77269900

H 4.94905000 1.36166900 1.09653200

H 3.97306300 2.25385300 -0.08091800

H 3.41510700 2.09960200 1.58570200

C 3.70520900 0.20699500 -2.14110900

H 4.67674000 -0.27149800 -2.32712200

H 3.05906000 -0.01260500 -2.99594900

H 3.86284900 1.28767100 -2.10825700

C 2.20159000 -2.54303300 -1.85852200

H 3.13898300 -3.01614900 -2.18025900

H 1.50059100 -3.33451000 -1.58424300

H 1.78782300 -2.01632000 -2.72466100

C -3.79434300 0.23026000 -0.06497400

C -3.50969900 -1.15517100 -0.10255400

C -5.14770600 0.62598300 0.00170300

C -4.54653300 -2.08474600 -0.06716400

H -2.47910000 -1.47565300 -0.15939300

C -6.17562800 -0.30902000 0.03811600

H -5.35518000 1.69121700 0.02616900

C -5.88020300 -1.67470000 0.00420200

H -4.30958000 -3.14576200 -0.09719400

H -7.20863600 0.02493500 0.09153600

H -6.67965300 -2.41026500 0.03053200

**int-S17**

Rh 0.37357700 -0.65084100 -0.12236200

C 1.06326200 -1.28375600 -2.24084300

C -0.35257000 -1.52643600 -2.25114800

C 1.67583300 -2.10383000 -1.23398300

C -0.63003000 -2.45614500 -1.21060600

C 0.61566200 -2.81409100 -0.56678000

C -0.73693300 -0.18400800 2.58020900

O -1.44126500 0.16697200 3.53417700

C 0.70300500 -0.53219500 2.74738100

C 1.36946600 -0.82041100 1.62800500

H 2.42393600 -1.09176000 1.60140100

C 1.26066400 -0.53666300 4.14255100

H 1.13428500 0.44851400 4.60776100

H 0.71796400 -1.24632000 4.77795400

H 2.32365500 -0.79973000 4.14648900

N -1.15179800 -0.30836300 1.27426500

C 0.32753300 1.62517700 -0.31312200

C 1.50652800 1.27438400 -0.10837500

C -0.83505000 2.44025000 -0.49219300

C -1.63227800 2.79169700 0.61245900

C -1.20861300 2.86052600 -1.78151200

C -2.78713900 3.54210900 0.41892200

H -1.36336700 2.43673100 1.60038000

C -2.36783200 3.60900100 -1.96304700

H -0.58649300 2.58950100 -2.62948000

C -3.16203100 3.94665000 -0.86429100

H -3.40820600 3.79372100 1.27304600

H -2.65366300 3.92724800 -2.96156100

H -4.07227700 4.52125800 -1.00875200

C 2.92347700 1.43924900 0.09916500

C 3.78677100 1.59433400 -1.00000100

C 3.46083500 1.47514600 1.39891500

C 5.15627300 1.75838300 -0.80400700

H 3.37260700 1.60110900 -2.00131100

C 4.83087800 1.64106400 1.58604500

H 2.79367700 1.37383500 2.24556800

C 5.68432300 1.77495200 0.48862100

H 5.81071000 1.88012700 -1.66245200

H 5.23234400 1.67009800 2.59492600

H 6.75228400 1.90193600 0.63993300

C -1.31384900 -0.94612100 -3.24292400

H -1.18324000 -1.40575100 -4.23214100

H -2.34891100 -1.10519700 -2.93482700

H -1.16726500 0.13312300 -3.36387000

C -1.95236000 -3.06000000 -0.85843100

H -2.78474300 -2.50658200 -1.29332300

H -1.99387900 -4.09717700 -1.21742000

H -2.10474900 -3.07280000 0.22405300

C 1.76629300 -0.38891300 -3.21218300

H 1.65096700 -0.77042400 -4.23460000

H 1.34959400 0.62446000 -3.19086900

H 2.83401800 -0.32380700 -2.99643900

C 3.14519200 -2.24148500 -0.97677600

H 3.60234500 -2.96073100 -1.66894600

H 3.66458600 -1.28532000 -1.08998900

H 3.33561200 -2.59898000 0.03884200

C 0.76017200 -3.86244400 0.49248200

H -0.07697300 -3.82343800 1.19452100

H 0.78537500 -4.86710100 0.04910700

H 1.67808100 -3.72376300 1.06857200

C -2.51157500 -0.31165700 0.92222400

C -2.90213200 0.04234800 -0.38148000

C -3.52478200 -0.72442400 1.81478500

C -4.23278700 -0.01111400 -0.78484400

H -2.14865500 0.37110700 -1.08013600

C -4.85365100 -0.78297100 1.40034900

H -3.26045800 -0.97768400 2.83159700

C -5.22625300 -0.42997800 0.10149900

H -4.48975300 0.28759600 -1.79857600

H -5.60913400 -1.11168500 2.10998100

H -6.26631600 -0.47327700 -0.20882500

**int-S18**

Rh 0.71820000 -0.19670900 0.74047900

C 2.10414800 -1.87032800 1.38432900

C 0.77831300 -2.47436100 1.34916400

C 2.10598100 -0.83696400 2.37295200

C -0.04374400 -1.76104800 2.24474000

C 0.75538200 -0.70428400 2.85704700

C -1.07060800 2.06623700 0.55913200

O -1.10441500 0.74362700 0.65202600

C 0.27403400 2.65397100 0.73416400

C 1.26475600 1.75096000 0.81831200

H 2.31645900 2.02588300 0.89915200

C 0.43816800 4.14818300 0.73934300

H 0.00587200 4.57986300 -0.17103300

H -0.10090100 4.60113100 1.57976100

H 1.49433900 4.43060100 0.80408100

N -2.10510800 2.79536800 0.28902700

C 0.29089200 -0.61627400 -1.42341800

C 1.36630900 0.01039300 -1.44573900

C -0.82622000 -1.41956100 -1.84278600

C -2.13790700 -1.15584900 -1.40812000

C -0.58668600 -2.50734600 -2.70492600

C -3.18479000 -1.96935800 -1.83328100

H -2.31192500 -0.32524800 -0.73758000

C -1.64123600 -3.31626800 -3.11966600

H 0.42691100 -2.70516700 -3.04073900

C -2.94251100 -3.05067200 -2.68445800

H -4.19230400 -1.74265500 -1.49738700

H -1.44731100 -4.15415400 -3.78347600

H -3.76328300 -3.68282200 -3.01189700

C 2.55452500 0.68955700 -1.87846300

C 3.71749500 -0.04719800 -2.16739000

C 2.57414400 2.08893200 -2.02415200

C 4.87649200 0.60330400 -2.58360600

H 3.69789200 -1.12697000 -2.06748600

C 3.73740900 2.73016200 -2.44174200

H 1.67657400 2.65270300 -1.80207400

C 4.89180500 1.99347600 -2.71816600

H 5.76783200 0.02372700 -2.80670200

H 3.74140900 3.81058900 -2.55345600

H 5.79664100 2.49926400 -3.04244800

C 0.38334400 -3.63383300 0.48934600

H 0.65574700 -4.58562700 0.96446600

H -0.69165900 -3.64299400 0.29616000

H 0.88225600 -3.59569800 -0.48272900

C -1.51518600 -1.92283800 2.46595300

H -1.90796100 -2.79167600 1.93320800

H -1.74116500 -2.04313000 3.53209600

H -2.04543900 -1.03416300 2.10173900

C 3.29307200 -2.36382100 0.62144400

H 3.81424200 -3.15668500 1.17455300

H 2.99337200 -2.77924700 -0.34499500

H 4.00820100 -1.55898200 0.43199900

C 3.28964700 -0.03558300 2.81584400

H 3.79344900 -0.52082900 3.66189800

H 4.02098500 0.07382000 2.00980500

H 2.99216900 0.96717400 3.13371700

C 0.25430600 0.23526400 3.90884700

H -0.73120700 0.62191800 3.63324200

H 0.16538300 -0.26744100 4.88104600

H 0.92374000 1.09047100 4.02740400

C -3.32629600 2.13637400 0.06337200

C -3.94499100 2.25570600 -1.19163100

C -3.97208300 1.37519000 1.05543900

C -5.14859300 1.60458900 -1.45911000

H -3.45178000 2.85103600 -1.95419700

C -5.17914100 0.73350000 0.78752700

H -3.50670800 1.28805000 2.03237200

C -5.77285500 0.83528800 -0.47436400

H -5.60093500 1.69868200 -2.44326200

H -5.65866600 0.14684300 1.56771900

H -6.71439800 0.33406800 -0.68154100

**TS-S19**

Rh -0.15126100 -0.67386200 -0.41936900

C 0.10047800 -0.59890300 -2.69675600

C -1.29748800 -0.46020800 -2.42692300

C 0.53165000 -1.87310700 -2.18573700

C -1.72381400 -1.65518000 -1.73453200

C -0.61148800 -2.54650800 -1.62331500

C -0.86419300 -1.04496800 2.45847600

O -1.42181400 -1.06523800 3.56282700

C 0.39778900 -1.79861200 2.21570200

C 1.03654800 -1.59918600 1.04310800

H 1.90320200 -2.18876600 0.75537000

C 0.83848000 -2.75232500 3.28505900

H 0.95945900 -2.21605700 4.23414800

H 0.07035000 -3.51360700 3.46715800

H 1.77843200 -3.24952700 3.02479900

N -1.29928600 -0.39224300 1.33494400

C 0.77881800 1.07807400 0.22029200

C 1.67266700 0.22644800 0.60034300

C 0.58675200 2.50099400 0.12673100

C 1.13742300 3.33972500 1.11690000

C -0.11053300 3.08970900 -0.94275500

C 0.99826800 4.72092500 1.02772300

H 1.66291400 2.88899600 1.95261500

C -0.24126300 4.47270800 -1.03021200

H -0.54761100 2.44837900 -1.69779400

C 0.31081600 5.29495100 -0.04565900

H 1.42377100 5.35337400 1.80176200

H -0.78161400 4.90964700 -1.86544900

H 0.19993700 6.37344900 -0.10887200

C 3.12850000 0.12090400 0.62399800

C 3.81973400 -0.53620400 1.65437000

C 3.86392500 0.73987200 -0.40382300

C 5.21247200 -0.57392400 1.65479200

H 3.25590800 -0.99794700 2.45724100

C 5.25585400 0.69634000 -0.40164200

H 3.32634600 1.26012000 -1.19020600

C 5.93366400 0.03556100 0.62566900

H 5.73652600 -1.07743800 2.46207300

H 5.81184300 1.17738100 -1.20136700

H 7.01927800 -0.00047400 0.62628400

C -0.63246400 -3.91651700 -1.01955400

H -1.38264300 -3.98348100 -0.22673200

H -0.86885600 -4.68142100 -1.77120400

H 0.33403300 -4.17249400 -0.57625000

C -3.12687300 -1.93059800 -1.29634600

H -3.69723600 -2.38661400 -2.11689800

H -3.15325100 -2.61608100 -0.44579500

H -3.63741800 -1.01343600 -0.99560000

C -2.21990800 0.61899500 -2.90286200

H -2.85623400 0.25029100 -3.71870800

H -2.87900700 0.96454400 -2.10128300

H -1.66818700 1.48134300 -3.28684500

C 0.98242900 0.36810100 -3.42614200

H 0.54662000 1.36906700 -3.45847800

H 1.96102200 0.44907200 -2.94357500

H 1.15105800 0.04061800 -4.46042000

C 1.92017200 -2.42608900 -2.29248100

H 2.08469600 -2.89220800 -3.27297700

H 2.67344200 -1.64301400 -2.16353300

H 2.10247300 -3.18997700 -1.53138200

C -2.57958900 0.17972300 1.28063100

C -3.68855700 -0.38887200 1.94159400

C -2.79978200 1.31218700 0.47886600

C -4.96087300 0.15324200 1.77840400

H -3.53496100 -1.24167800 2.58874100

C -4.07605800 1.84999000 0.32341300

H -1.95442500 1.76936400 -0.01411100

C -5.17143900 1.27082800 0.96620100

H -5.80032800 -0.30761000 2.29322500

H -4.20847100 2.73197300 -0.29891500

H -6.16751300 1.68717200 0.84612900

**TS-S20**

Rh 0.58113500 0.84938900 -0.56235200

C 1.45467300 2.70438700 0.42197900

C 0.18943100 3.00186500 -0.15368800

C 2.31670100 2.24144100 -0.63899000

C 0.29734300 2.83213400 -1.60542200

C 1.60968400 2.39786100 -1.89920000

C -1.59796800 -0.93370100 -1.33894400

O -1.40664300 0.17374700 -0.65065800

C -0.41523600 -1.40969200 -2.09220300

C 0.79198900 -0.93598700 -1.68810400

H 1.70357900 -1.19444800 -2.22021000

C -0.60715700 -2.36523100 -3.23316900

H -1.19803400 -3.22301800 -2.89344900

H -1.18484200 -1.90043900 -4.04142800

H 0.35085400 -2.70895600 -3.63691300

N -2.69381600 -1.62960000 -1.42058300

C 0.63847700 -0.46880000 1.01148500

C 1.25984400 -1.22213500 0.16977500

C 0.19634700 -0.22967700 2.36525900

C 1.05803500 -0.43082100 3.45759900

C -1.10526500 0.25469000 2.59416900

C 0.62977700 -0.14035100 4.75183700

H 2.05853500 -0.81336900 3.28174200

C -1.52652100 0.53627400 3.89072500

H -1.76594200 0.37862100 1.74305600

C -0.66008000 0.34769000 4.97182400

H 1.30427200 -0.29502500 5.58948400

H -2.53679400 0.89869300 4.05896100

H -0.99082600 0.57391600 5.98156500

C 2.32270200 -2.22388200 0.13129200

C 3.50214900 -1.99541400 0.86123900

C 2.18603500 -3.42133300 -0.58825400

C 4.51758700 -2.94918300 0.87909000

H 3.61186700 -1.05997200 1.40153300

C 3.19963400 -4.37638400 -0.56069500

H 1.27465200 -3.59696900 -1.14921300

C 4.36793500 -4.14237000 0.16887600

H 5.42541300 -2.76119000 1.44535100

H 3.07786700 -5.30619000 -1.10883800

H 5.15872500 -4.88689200 0.18328700

C -0.83507600 3.05956200 -2.55670200

H -1.71247500 2.48114700 -2.24755500

H -1.11997700 4.11898800 -2.58566600

H -0.57323400 2.75248900 -3.57214600

C -1.04298000 3.47122900 0.55407900

H -1.15831500 4.56021300 0.47022200

H -1.93305200 3.00443400 0.12239300

H -1.01483900 3.21284400 1.61565100

C 1.81219600 2.78729700 1.87341500

H 2.25728400 3.76261900 2.10951800

H 0.93455200 2.65144200 2.50995000

H 2.53487200 2.01546100 2.15224700

C 3.75339700 1.84225100 -0.49567500

H 4.42083400 2.70269100 -0.64021400

H 3.95424300 1.43152000 0.49775900

H 4.02964500 1.08144100 -1.23166400

C 2.18210200 2.09211000 -3.24857100

H 2.78766800 2.92967100 -3.61917200

H 2.82815300 1.20939000 -3.21447600

H 1.39487800 1.89639000 -3.98102600

C -3.87595000 -1.29544400 -0.76025400

C -4.87174100 -2.29554300 -0.75848100

C -4.18079100 -0.06675200 -0.12846200

C -6.10175500 -2.09623100 -0.14239400

H -4.63887400 -3.23286900 -1.25460300

C -5.42117800 0.12862100 0.47803100

H -3.44052100 0.72049700 -0.12831600

C -6.38693600 -0.87896600 0.48373000

H -6.84420100 -2.89047600 -0.15281200

H -5.63350100 1.08339500 0.95454900

H -7.34813500 -0.71839900 0.96447600

**int-S21**

Rh -1.38494000 -0.29794900 -0.29635200

C -3.27714400 0.68771000 0.40215600

C -2.90792900 -0.38529400 1.28252400

C -3.50299900 0.11826700 -0.89145700

C -3.06607700 -1.63904700 0.54666100

C -3.44526000 -1.33483400 -0.77318600

C 0.91361000 -1.56179600 -0.81498300

O 0.27498000 -1.44440600 0.32983700

C 0.09223500 -0.98208800 -1.94607400

C -0.06748000 0.39130400 -2.05161700

H -0.61760300 0.77672900 -2.91095300

C -0.37804300 -1.93742600 -3.00638300

H 0.50111100 -2.32534800 -3.53414200

H -0.89582300 -2.79596400 -2.56789000

H -1.04058400 -1.45082800 -3.72942100

N 2.04567300 -2.10572900 -1.06946700

C -0.05413400 1.21953000 0.06810400

C 0.57936100 1.36584800 -1.10451700

C 0.11861700 1.76787500 1.40773000

C -0.22648900 3.09667800 1.71420600

C 0.59439000 0.93559900 2.43978100

C -0.09278700 3.58303800 3.01504800

H -0.59270700 3.74283600 0.92170800

C 0.73642100 1.43115400 3.73335700

H 0.83767300 -0.09406100 2.20001800

C 0.39054300 2.75318600 4.02877000

H -0.36301700 4.61231900 3.23506000

H 1.11566400 0.78075500 4.51692900

H 0.49812800 3.13384800 5.04045600

C 1.73597000 2.17346200 -1.51983600

C 2.31764500 3.14840700 -0.68811600

C 2.30223200 1.95921400 -2.78834900

C 3.42193700 3.87855400 -1.11650000

H 1.90569400 3.32869100 0.29719200

C 3.40579000 2.69434300 -3.21621400

H 1.88405500 1.19401800 -3.43580000

C 3.97091100 3.65992900 -2.38301600

H 3.85771700 4.62409400 -0.45674800

H 3.82862900 2.50608800 -4.19926700

H 4.83241700 4.23321300 -2.71342600

C -3.92223200 0.86124000 -2.12314200

H -3.55292300 0.36900900 -3.02783300

H -5.01700700 0.91043100 -2.19814900

H -3.54127400 1.88596400 -2.11957300

C -3.41714100 2.12873200 0.77555700

H -4.44100000 2.33674000 1.11396500

H -2.73491200 2.40066300 1.58395400

H -3.20670300 2.78486100 -0.07375800

C -3.73867100 -2.29489100 -1.88386700

H -4.82115300 -2.43250600 -2.00814500

H -3.34638100 -1.93551900 -2.84003200

H -3.29895800 -3.27761200 -1.69437100

C -2.76256700 -2.98436200 1.12863100

H -3.38241700 -3.17812100 2.01232200

H -2.94178500 -3.78524100 0.40720800

H -1.71105700 -3.03707300 1.43476200

C -2.62484000 -0.28106700 2.74799400

H -2.20627500 0.69430700 3.00525000

H -3.54239800 -0.42585500 3.33498900

H -1.90252800 -1.03985500 3.06169900

C 2.88500100 -2.52380600 -0.02048500

C 3.43106600 -3.81518400 -0.05471800

C 3.27078900 -1.65431500 1.01612100

C 4.31647100 -4.23761700 0.93633000

H 3.14786000 -4.47596500 -0.86879200

C 4.16431900 -2.07813400 1.99611400

H 2.87282000 -0.64471800 1.03124500

C 4.68877600 -3.37352500 1.96774600

H 4.72370800 -5.24494200 0.89703700

H 4.45484400 -1.39089200 2.78709900

H 5.38520900 -3.70111600 2.73458300

**MeOH**

O -0.74911100 -0.12251500 -0.00000200

H -1.13352900 0.76659100 0.00005800

C 0.66226900 0.01949800 -0.00000300

H 1.03660100 0.54307400 0.89355600

H 1.07973900 -0.99102500 -0.00080500

H 1.03646700 0.54449700 -0.89277100

**HOAc**

C -0.09214600 0.12597400 -0.00012400

O -0.64529600 1.20206400 0.00002800

O -0.77786000 -1.04691100 0.00000400

H -1.72245900 -0.80296600 0.00015300

C 1.39644200 -0.10976200 0.00000200

H 1.68279300 -0.69076100 0.88257800

H 1.68275500 -0.69282300 -0.88120500

H 1.91638000 0.84805100 -0.00104200

**S22 (Me)**

Rh -0.78787800 0.29825000 0.14394700

C -1.67408600 2.27730800 -0.89736700

C -2.75085900 1.42545300 -0.53877100

C -0.82530400 1.53165400 -1.78650400

C -2.65259500 0.19455300 -1.30168200

C -1.48620200 0.27143300 -2.09236800

C 1.36358100 -3.02943200 1.92597400

C 0.91960100 -1.77807500 1.72889800

H 0.50937700 -1.24493300 2.58143200

C 1.27658400 -3.73505100 3.24626500

H 0.69180800 -4.66014600 3.15555800

H 2.27036400 -4.02327500 3.61655200

H 0.78964900 -3.10939900 4.00049800

N -1.77392600 -0.49079500 1.74413300

C 1.01590900 0.42940400 0.63407900

C 0.97168800 -0.98992800 0.48166900

C 2.05427400 1.39343800 0.82709800

C 1.76262200 2.77280200 0.90132400

C 3.39491700 0.97242800 0.98804600

C 2.77891900 3.70083700 1.08625500

H 0.72788400 3.08235700 0.82386600

C 4.40706100 1.90522300 1.18354000

H 3.61638100 -0.08965200 0.95990100

C 4.10380300 3.26997500 1.22411000

H 2.54462700 4.76027600 1.13588300

H 5.43366900 1.57262200 1.30581100

H 4.89686100 3.99675000 1.37471400

C 1.41830400 -1.64078100 -0.78386400

C 2.35373200 -1.04080500 -1.64246500

C 0.86280000 -2.87392000 -1.16964500

C 2.73679700 -1.66204600 -2.83245900

H 2.78718700 -0.08262100 -1.37851800

C 1.24787400 -3.49491100 -2.35492900

H 0.10986700 -3.32985600 -0.53554900

C 2.19164600 -2.89487100 -3.19276000

H 3.46758600 -1.17987800 -3.47630500

H 0.80251300 -4.44707600 -2.63020700

H 2.49210900 -3.37924800 -4.11722800

C -3.87568000 1.74472300 0.39314100

H -3.65965600 2.62896800 0.99911500

H -4.80235800 1.93723500 -0.16414700

H -4.06261000 0.90450300 1.06944200

C -1.50206800 3.70582100 -0.47472000

H -2.22092400 4.35570200 -0.99246200

H -1.66212000 3.84066300 0.60037100

H -0.50298700 4.07676600 -0.71736200

C 0.39161700 2.05292900 -2.49109800

H 0.11504800 2.61067200 -3.39620200

H 0.97386000 2.72358100 -1.85181100

H 1.04848200 1.23327100 -2.79378100

C -1.01165800 -0.72949200 -3.09524000

H -1.36850300 -0.46448200 -4.10035800

H 0.07838500 -0.77884600 -3.13386500

H -1.37761600 -1.73288100 -2.86567500

C -3.65797700 -0.91380500 -1.25777900

H -3.94859400 -1.13981000 -0.22713200

H -4.57006300 -0.64641700 -1.80902700

H -3.26061200 -1.83173300 -1.69962400

H 1.80101600 -3.58863800 1.10186300

C -2.05112200 -1.81413600 1.83612200

O -2.44410400 -2.40685800 2.84086700

H -1.90292700 -2.34070100 0.87314500

C -1.87889600 0.29790400 2.96054000

H -0.94189000 0.27303900 3.54015100

H -2.08443900 1.34209800 2.70590100

H -2.67825200 -0.09156200 3.59906800

**S22 (Ph)**

Rh -0.22568300 -0.25207500 -0.44862400

C -0.96585400 0.48871500 -2.59657900

C -1.65256200 -0.71187700 -2.26247700

C 0.43661800 0.18857200 -2.58297600

C -0.69512100 -1.79699900 -2.17589500

C 0.58939600 -1.24922400 -2.38714600

C 1.42018000 -1.09398000 3.48773600

C 0.78537500 -0.45644700 2.49115700

H -0.20466300 -0.05278700 2.68309400

C 0.82725900 -1.29572600 4.85062400

H 0.74475400 -2.36458100 5.09036100

H 1.45578900 -0.84592700 5.63169000

H -0.17393500 -0.85833100 4.91998800

N -1.65584800 -0.97069700 0.86652200

C 0.79083500 1.00229900 0.50906100

C 1.31048300 -0.16554100 1.14271300

C 1.11749300 2.39177200 0.51823900

C 0.40960200 3.31456600 -0.28370000

C 2.12781900 2.87689800 1.38240600

C 0.72625200 4.66613300 -0.25191000

H -0.38981900 2.94248100 -0.91165700

C 2.43364900 4.23204300 1.41639400

H 2.65144500 2.17254100 2.02079200

C 1.74107700 5.12787000 0.59470200

H 0.17899900 5.36659000 -0.87603500

H 3.21030200 4.59538800 2.08288200

H 1.98366100 6.18622200 0.62214900

C 2.60118500 -0.76483100 0.70003300

C 3.59931700 -0.00442200 0.06921800

C 2.82536600 -2.14328300 0.86618800

C 4.78751100 -0.59712100 -0.36151900

H 3.44838800 1.05847900 -0.08426400

C 4.01167700 -2.73351400 0.43846900

H 2.04740900 -2.74970100 1.31824700

C 5.00287400 -1.96305500 -0.17522100

H 5.54707900 0.01418900 -0.84146000

H 4.15965600 -3.80103700 0.57711100

H 5.92827000 -2.42365900 -0.50838400

C -3.13633800 -0.86735800 -2.15929600

H -3.61681600 0.05550100 -1.82660500

H -3.55715500 -1.13969900 -3.13740900

H -3.40712900 -1.65208700 -1.44956100

C -1.62556000 1.79461100 -2.92197800

H -2.17354700 1.72978600 -3.87159700

H -2.34931500 2.08459100 -2.15145300

H -0.89291600 2.59914000 -3.02728900

C 1.54978100 1.11863100 -2.96458900

H 1.69738400 1.12617900 -4.05296000

H 1.34594200 2.14555700 -2.64837800

H 2.49318300 0.81262900 -2.50442400

C 1.88306600 -1.99369400 -2.45382100

H 2.11524600 -2.26596000 -3.49289500

H 2.71430500 -1.39586500 -2.07425600

H 1.84793000 -2.91480700 -1.86752700

C -1.04271100 -3.23442100 -1.94003200

H -1.81553800 -3.34134300 -1.17265900

H -1.42231000 -3.70646900 -2.85654600

H -0.17047300 -3.80637900 -1.61113700

H 2.41812900 -1.49676000 3.32758900

C -1.48378100 -2.25432600 1.29887100

O -2.28783200 -2.98057900 1.87927600

H -0.46559100 -2.61387800 1.06308700

C -2.85350300 -0.25035300 1.02332200

C -4.10064900 -0.84875000 1.30552200

C -2.81745400 1.14830300 0.84344900

C -5.25470100 -0.07006000 1.36404900

H -4.14043200 -1.91377800 1.48060900

C -3.97924300 1.91397300 0.90219700

H -1.86066400 1.62081500 0.65419400

C -5.21224500 1.31017300 1.15483800

H -6.20447800 -0.55595500 1.57359200

H -3.91588300 2.98988900 0.75765800

H -6.12045400 1.90447000 1.19940700

**S22 (*p*-OMe-C_6_H_4_)**

Rh 0.22379100 -0.35412400 -0.46457700

C -0.44372600 0.16686500 -2.70390700

C -0.99645300 -1.09323200 -2.34561300

C 0.98056200 0.05369200 -2.57515400

C 0.08134300 -2.03757300 -2.12210500

C 1.29853600 -1.33907700 -2.27983500

C 1.67995700 -0.72756300 3.61770100

C 1.03382200 -0.24902700 2.54240100

H -0.01558400 0.01286000 2.64401600

C 1.02512400 -0.93968200 4.95035800

H 1.07945200 -1.99439100 5.25286700

H 1.52449800 -0.36024500 5.73922600

H -0.03080700 -0.65155100 4.92764100

N -1.18715900 -1.18961200 0.79592600

C 0.97862300 1.08466600 0.47391100

C 1.60874600 0.04048000 1.21409200

C 1.09748400 2.50526900 0.41439900

C 0.32130700 3.26443200 -0.48958900

C 1.96291700 3.18506100 1.30439600

C 0.43368900 4.64781200 -0.52867200

H -0.36790200 2.74080800 -1.13964400

C 2.06420800 4.57044400 1.26613600

H 2.53875500 2.60486100 2.01829800

C 1.30759200 5.30430800 0.34591900

H -0.16471800 5.22122900 -1.23066400

H 2.73103400 5.08346300 1.95291300

H 1.39044300 6.38690700 0.31776100

C 3.00253400 -0.38207200 0.89750100

C 3.91760200 0.48484200 0.27767400

C 3.41869500 -1.69740200 1.17059100

C 5.20819900 0.05848900 -0.04016800

H 3.61848000 1.50053700 0.04371400

C 4.70697600 -2.12159400 0.85544900

H 2.71251900 -2.38972700 1.61653200

C 5.61204400 -1.24503200 0.25116400

H 5.89900500 0.75053500 -0.51434800

H 5.00321500 -3.14384000 1.07460800

H 6.61655100 -1.57689900 0.00542200

C -2.45233700 -1.43486000 -2.34418600

H -3.06979100 -0.56990900 -2.09119800

H -2.75625500 -1.79457200 -3.33735100

H -2.67788000 -2.22128500 -1.62096100

C -1.23516800 1.35746900 -3.15601900

H -1.71256200 1.16429800 -4.12608200

H -2.03465400 1.60609000 -2.44818500

H -0.59886300 2.23799700 -3.27982900

C 1.99039600 1.10158100 -2.93960800

H 2.19351600 1.09009300 -4.01906000

H 1.64555000 2.10572600 -2.67600200

H 2.93851200 0.93174700 -2.42270900

C 2.67795300 -1.90804700 -2.20904700

H 3.04741200 -2.14660500 -3.21617600

H 3.38084000 -1.20636900 -1.75561500

H 2.70297500 -2.82651200 -1.61831100

C -0.10089300 -3.49174300 -1.81329600

H -0.90298700 -3.65189700 -1.08607000

H -0.35777300 -4.06137800 -2.71689400

H 0.81135100 -3.92688500 -1.39572200

H 2.73470500 -0.98524200 3.54881200

C -0.88336200 -2.40918600 1.32481600

O -1.63473800 -3.20737100 1.88347400

H 0.19063800 -2.63728300 1.19897500

C -2.48570600 -0.64450100 0.80027800

C -3.65854600 -1.39394100 1.00839600

C -2.63460900 0.73232600 0.52692200

C -4.91859800 -0.80082700 0.91085800

H -3.57100200 -2.44226700 1.25404300

C -3.88614300 1.32258200 0.42305400

H -1.74377800 1.33297800 0.38674800

C -5.04516300 0.55814400 0.60668200

H -5.79480400 -1.41810200 1.07394300

H -3.98900100 2.38233300 0.21008500

O -6.23364600 1.22625000 0.47733400

C -7.42455400 0.48446500 0.65912100

H -8.24576600 1.18895200 0.51280700

H -7.51011200 -0.32977400 -0.07360700

H -7.48691700 0.05975700 1.67030300

**S22 (Ms)**

Rh -0.29030700 -0.42059400 -0.48422700

C -0.93015200 -0.12385800 -2.76437200

C -1.54626700 -1.30157800 -2.25131700

C 0.48287600 -0.29956600 -2.62042800

C -0.52202700 -2.27350600 -1.92109200

C 0.72920800 -1.66501600 -2.15813300

C 1.15281000 -0.55739300 3.59592200

C 0.57370300 -0.05305800 2.49349400

H -0.41384800 0.38580200 2.59596500

C 0.46576900 -0.58733300 4.92931600

H 0.40888700 -1.61321700 5.31809600

H 1.01449900 0.00266700 5.67679700

H -0.55446500 -0.19898000 4.85728900

N -1.86945700 -0.82799900 0.83148500

C 0.56428400 1.05246700 0.29930400

C 1.15053800 0.05925700 1.13931300

C 0.69958200 2.45473300 0.09244500

C -0.19621100 3.12727500 -0.76862100

C 1.67748900 3.19940900 0.79248600

C -0.07939500 4.49695100 -0.96394400

H -0.99464600 2.55497700 -1.22296000

C 1.78651200 4.56944600 0.59235200

H 2.33290400 2.68459500 1.48743900

C 0.91591100 5.21648500 -0.29245200

H -0.77263500 5.01253700 -1.62164400

H 2.54006800 5.13909800 1.12779000

H 1.00103900 6.28900900 -0.44309400

C 2.51215500 -0.47338400 0.85194800

C 3.47091400 0.27333200 0.14728900

C 2.85321800 -1.77977100 1.24520700

C 4.73190300 -0.25618300 -0.13073200

H 3.23027000 1.27593600 -0.18835300

C 4.11315900 -2.30631200 0.97185900

H 2.10916600 -2.38661700 1.74963100

C 5.06347400 -1.54599900 0.28584500

H 5.45680300 0.34535800 -0.67259700

H 4.35052200 -3.31863300 1.28741200

H 6.04575200 -1.95691800 0.07214200

C -3.01818900 -1.55036000 -2.16842400

H -3.57022500 -0.61151000 -2.10061600

H -3.36509200 -2.10117300 -3.05355300

H -3.26197600 -2.14910000 -1.28614200

C -1.65445000 1.05740900 -3.33293000

H -2.10335000 0.81286400 -4.30531600

H -2.45737000 1.38060700 -2.66339600

H -0.97750500 1.90142600 -3.49105400

C 1.54252400 0.65369500 -3.08603000

H 1.75723400 0.50961100 -4.15342600

H 1.23291800 1.69341200 -2.94547700

H 2.47595100 0.50676700 -2.53656500

C 2.07401200 -2.30437200 -2.03945000

H 2.37697600 -2.73448300 -3.00433500

H 2.84076800 -1.58672100 -1.74199400

H 2.07356400 -3.10952500 -1.30109000

C -0.77602400 -3.66714900 -1.43314900

H -1.61593900 -3.69481900 -0.73127700

H -1.01913500 -4.34778800 -2.26078900

H 0.09942400 -4.07291500 -0.91757800

H 2.15982100 -0.96655800 3.55583600

C -1.76394200 -1.77553200 1.82060000

O -2.45709200 -1.90197100 2.82138800

H -0.94745600 -2.48191300 1.58593500

S -2.90860000 0.48634200 1.03575700

O -2.46705500 1.32962600 2.15878700

O -3.04691200 1.12360000 -0.28774300

C -4.50613200 -0.23323600 1.44648900

H -4.81303400 -0.87066400 0.61538500

H -4.38843900 -0.80551600 2.36510600

H -5.19889600 0.60246500 1.56788600

**S22 (Ts)**

Rh 1.50522200 -0.52476900 0.07846600

C 3.46726100 0.25416100 0.64773400

C 3.76796100 -1.11215500 0.17590300

C 2.66408500 0.15375000 1.82131200

C 3.10617400 -2.02025300 1.00162200

C 2.32811100 -1.23732200 1.96950000

C -1.31595600 -1.26503000 1.79147200

C -1.55281600 0.05478600 1.73261500

H -2.34193700 0.42759700 2.38484400

C -2.03809900 -2.18793400 2.72828900

H -2.55925800 -2.97573100 2.17243200

H -1.34591800 -2.69275600 3.41740800

H -2.77828900 -1.64926300 3.33102800

N 0.37888400 -1.50914600 -1.31600600

C 0.18897600 1.02782700 0.16771100

C -0.97530800 1.09639100 0.86118700

C 0.82397400 2.18511100 -0.50260800

C 1.01835200 3.39213400 0.20083600

C 1.35152700 2.09369100 -1.80553100

C 1.71240100 4.45681800 -0.36998100

H 0.59913000 3.49220300 1.19598100

C 2.03971800 3.16280300 -2.37644400

H 1.17682400 1.19102100 -2.37649800

C 2.23326200 4.34687200 -1.66196700

H 1.84248900 5.37638100 0.19474000

H 2.42149300 3.07049000 -3.38991500

H 2.77327300 5.17708300 -2.10843000

C -1.82046000 2.33108700 0.75931300

C -2.13370200 2.87263700 -0.49867900

C -2.33049000 2.96651200 1.90177700

C -2.92704800 4.01250700 -0.60732700

H -1.74062100 2.39218100 -1.38845400

C -3.12066800 4.11134600 1.79394100

H -2.08931900 2.57380300 2.88609900

C -3.42459600 4.63835600 0.53801100

H -3.15847800 4.41191100 -1.59104900

H -3.49611900 4.59314400 2.69301800

H -4.04295400 5.52760900 0.45252700

C 4.62266700 -1.40428700 -1.01492600

H 5.67457600 -1.17238300 -0.80305100

H 4.55827300 -2.45411900 -1.30977800

H 4.32129100 -0.79250500 -1.87151900

C 4.10278400 1.48717300 0.09461900

H 5.12715700 1.58227700 0.48122300

H 4.15565600 1.45292300 -0.99578400

H 3.54543300 2.38474000 0.36513800

C 2.23577700 1.25852500 2.73336000

H 2.88726600 1.30082400 3.61595200

H 2.28278400 2.22432600 2.22795700

H 1.20665300 1.11008000 3.07067000

C 1.55238400 -1.83594400 3.09291600

H 2.23021200 -2.04928000 3.93182000

H 0.76964500 -1.16088700 3.44223800

H 1.08510000 -2.77652200 2.79256600

C 3.13187600 -3.51394500 0.95817500

H 3.67074800 -3.88072900 0.08128000

H 3.63150400 -3.90986000 1.85181700

H 2.12323300 -3.93470200 0.91939200

H -0.60517300 -1.72798800 1.11343400

C 0.84944800 -2.84304500 -1.45501200

O 0.48307100 -3.77391500 -0.76768500

H 1.64635000 -2.97058400 -2.20947000

S -0.91644000 -1.14401000 -2.30896400

O -0.90328500 0.28889600 -2.62344800

O -0.86796400 -2.12971300 -3.40293400

C -2.39945200 -1.45644000 -1.36341200

C -3.26156000 -0.40211400 -1.08262900

C -2.68364600 -2.75464700 -0.93326400

C -4.40939000 -0.64394400 -0.33080500

H -3.02719500 0.59308100 -1.43332800

C -3.84199200 -2.98028400 -0.19795100

H -2.00416900 -3.56805500 -1.15830500

C -4.71290300 -1.92948700 0.12889600

H -5.07370300 0.18323300 -0.09537600

H -4.07193900 -3.98854200 0.13817700

C -5.92873200 -2.18322100 0.98449300

H -6.66057300 -1.37492400 0.89143100

H -6.42107000 -3.12367500 0.71363600

H -5.65060600 -2.25637100 2.04430500

**S22 (*p*-Cl-C_6_H_4_SO_2_)**

Rh 0.76777800 0.04258100 -0.68044100

C 0.74446700 1.64336900 -2.45263300

C -0.18531900 0.61189300 -2.75322700

C 2.02427500 1.01458400 -2.29769800

C 0.52622000 -0.64121500 -2.92747900

C 1.88971700 -0.39349600 -2.67193700

C 1.22380300 -2.56496000 2.74359700

C 1.01067200 -1.42395600 2.06726500

H 0.12528000 -0.84741400 2.31647600

C 0.26088300 -3.08758900 3.76806500

H -0.08118200 -4.09697700 3.50227500

H 0.72962200 -3.16317400 4.75917200

H -0.62106400 -2.44497800 3.84370600

N -1.10570400 -0.60224400 0.02443000

C 1.68475200 0.60242700 0.85680300

C 1.87882300 -0.79757300 1.05143000

C 2.12632000 1.80711200 1.47476300

C 1.58804100 3.04745300 1.06511300

C 3.05021400 1.77433200 2.54549600

C 2.00294100 4.22376200 1.67501700

H 0.81981800 3.04433300 0.30280100

C 3.45940000 2.95550800 3.15090200

H 3.42650100 0.81534600 2.88640200

C 2.94433400 4.18008100 2.71009900

H 1.58326300 5.17501100 1.36206200

H 4.17097500 2.92823700 3.97062900

H 3.26294700 5.10162600 3.18903100

C 3.13950800 -1.46617600 0.62372100

C 4.35984500 -0.77655100 0.53863100

C 3.11969400 -2.82156500 0.24855200

C 5.52245200 -1.42317600 0.11585400

H 4.40272800 0.27390900 0.80427100

C 4.28070600 -3.46794800 -0.16753200

H 2.17676700 -3.35751400 0.26672300

C 5.49143800 -2.77383500 -0.23296200

H 6.45474700 -0.86735800 0.06358500

H 4.23803300 -4.51575600 -0.45208100

H 6.39651400 -3.27791300 -0.55871800

C -1.65184500 0.78642100 -2.97709700

H -1.99439500 1.74809600 -2.59292100

H -1.88472700 0.73518400 -4.04946100

H -2.22084300 -0.00054200 -2.47407400

C 0.41465000 3.09883100 -2.31972200

H 0.16905400 3.53778500 -3.29620700

H -0.44644200 3.24613900 -1.66011800

H 1.25854200 3.66260300 -1.91368400

C 3.32760500 1.71621900 -2.05797100

H 3.75303400 2.08233800 -3.00199200

H 3.20321900 2.57535300 -1.39256100

H 4.05941400 1.04493300 -1.60186900

C 3.02036700 -1.35839000 -2.81745000

H 3.44441900 -1.29618500 -3.82944000

H 3.82484800 -1.15343600 -2.10894000

H 2.69376300 -2.38815400 -2.65572600

C -0.10697200 -1.93618900 -3.33435600

H -1.07334300 -2.07648900 -2.83882300

H -0.28725100 -1.97354400 -4.41767800

H 0.52708400 -2.78903900 -3.07508500

H 2.11650400 -3.15927700 2.56183300

C -1.31551600 -1.92101200 0.35242900

O -2.14893900 -2.38323000 1.11726400

H -0.61389200 -2.56634000 -0.20664300

S -1.96424400 0.60099200 0.83762700

O -1.70537600 0.54070500 2.28474800

O -1.67120400 1.85479300 0.11900800

C -3.70439000 0.25861200 0.56971400

C -4.38841100 -0.59266300 1.43774800

C -4.35769000 0.89708500 -0.48426200

C -5.74646200 -0.82455200 1.23395400

H -3.85612000 -1.07566700 2.24531900

C -5.71512500 0.66367100 -0.69409500

H -3.81033900 1.58190900 -1.11945400

C -6.39259000 -0.19911300 0.16721500

H -6.29910300 -1.48452300 1.89291800

H -6.24389300 1.14897400 -1.50667600

Cl -8.10255100 -0.49707900 -0.09318100

**23**

Rh -0.90054900 0.08009300 0.31272900

C -2.18729400 1.74408600 -0.50303400

C -3.03997900 0.69856800 -0.01972900

C -1.36232800 1.17812500 -1.53783500

C -2.84586600 -0.47566900 -0.85360500

C -1.80840300 -0.19252400 -1.77095800

C -1.03115600 -2.13249700 1.61347200

C -0.02851600 -1.24732500 1.92330900

H -0.03407500 -0.75740500 2.89275000

C -2.13273700 -2.50033700 2.55314600

H -3.10460500 -2.49938300 2.05016600

H -1.97399800 -3.52460400 2.91738400

H -2.17537300 -1.82917100 3.41516300

C 0.93104800 0.56996200 0.81360100

C 1.00355800 -0.82553100 0.93196800

C 1.89542500 1.61417800 0.66964000

C 1.47979700 2.94673100 0.45639100

C 3.28006700 1.34586500 0.79942100

C 2.41141700 3.96934900 0.33448200

H 0.41834700 3.15624200 0.42165600

C 4.20705400 2.37097700 0.67083600

H 3.60940700 0.32984700 0.98767700

C 3.77697000 3.68196100 0.43238100

H 2.08098200 4.99102000 0.17526800

H 5.26697300 2.15573200 0.76255400

H 4.50602300 4.48083200 0.33768700

C 1.85829700 -1.77351400 0.20174400

C 2.08541000 -3.06556400 0.70253200

C 2.43853100 -1.40563000 -1.02699600

C 2.87541200 -3.96851900 -0.00810200

H 1.66010500 -3.35331900 1.65885200

C 3.22543900 -2.30857900 -1.73230400

H 2.26332400 -0.40928500 -1.42129700

C 3.44520900 -3.59444200 -1.22550700

H 3.05112300 -4.96175200 0.39334200

H 3.66640400 -2.01498500 -2.68004500

H 4.05988200 -4.29834000 -1.77779600

C -4.07424000 0.84591300 1.05062400

H -3.80692800 1.63796700 1.75436700

H -5.04759100 1.10247200 0.61152900

H -4.20456300 -0.08048000 1.61685200

C -2.23458800 3.17477000 -0.06385700

H -3.17065600 3.63737800 -0.40176700

H -2.19445000 3.27188400 1.02482600

H -1.41406200 3.75389700 -0.49183900

C -0.38701400 1.90596400 -2.40747600

H -0.91427400 2.37958500 -3.24566100

H 0.14934200 2.68607800 -1.86394200

H 0.35377800 1.22189200 -2.82981800

C -1.23981700 -1.11216400 -2.80397700

H -1.57697400 -0.82400900 -3.80780600

H -0.14523700 -1.08608500 -2.80227900

H -1.54993800 -2.14571100 -2.63309100

C -3.65195100 -1.73428300 -0.78364400

H -4.08852700 -1.88078900 0.20677300

H -4.48530600 -1.68733500 -1.49617200

H -3.05894600 -2.61863700 -1.03312400

H -0.95394500 -2.69696800 0.68583900

**24 (Me)**

C -0.76728500 0.46513000 -0.00002900

O -1.33404000 -0.66324200 0.00003800

N 0.51313200 0.79271800 0.00013900

H -1.42517400 1.38001800 -0.00034600

C 1.37160400 -0.37764600 -0.00010200

H 2.43107500 -0.06626100 -0.00314800

H 1.22662200 -1.03934400 0.87863000

H 1.22196500 -1.04239900 -0.87562000

**24 (Ph)**

C 2.54492500 -0.31240100 0.00001200

O 2.75956000 0.92085700 0.00006900

N 1.42135800 -1.04195300 -0.00007900

C 0.18030200 -0.44125900 0.00003700

C -0.11673200 0.95372100 -0.00006100

C -0.94228500 -1.31485500 0.00005100

C -1.43342400 1.40890400 0.00003300

H 0.71859900 1.64111700 -0.00023500

C -2.25068800 -0.84823500 -0.00000900

H -0.73070200 -2.38182100 0.00008800

C -2.51863200 0.52644000 0.00000200

H -1.61771300 2.48429400 -0.00000300

H -3.07512000 -1.56226000 0.00001600

H -3.54226500 0.89697200 -0.00009000

H 3.44042500 -0.98538100 -0.00016800

**24 (*p*-OMe-C_6_H_4_)**

N 2.44211300 0.88724700 -0.15430500

C 3.42538500 -0.02186000 -0.18006300

O 3.43172100 -1.27003400 -0.08303500

H 4.41460700 0.48409800 -0.31836800

C 1.12657200 0.50702700 0.00753100

C 0.16362400 1.55360700 0.01158100

C 0.61237000 -0.81297700 0.17695800

C -1.19801700 1.31889200 0.16107700

H 0.54064100 2.56633000 -0.10854800

C -0.75338800 -1.04325300 0.32923000

H 1.32372000 -1.62778200 0.18473500

C -1.66470100 0.01284100 0.32478100

H -1.91270800 2.14027400 0.16400900

H -1.12820700 -2.05716600 0.46503900

O -3.03699700 -0.22820200 0.49024700

C -3.68892600 -0.50951100 -0.73130900

H -3.27441200 -1.40561700 -1.21846500

H -4.74802400 -0.68317800 -0.50473000

H -3.60570800 0.32960800 -1.43995700

**24 (Ms)**

N -0.59236700 1.01863400 0.00040300

S 0.53917200 -0.16738600 0.00005800

O 0.62846200 -0.94196400 1.26319100

O 0.62752100 -0.94256900 -1.26269400

C 2.00266600 0.90635700 -0.00069500

H 1.99072300 1.52886000 -0.89721700

H 1.99053400 1.53032300 0.89480900

H 2.87838800 0.25190200 -0.00007700

C -1.89295700 0.65380500 0.00003400

O -2.43962400 -0.45287800 -0.00018200

H -2.52896200 1.57496600 0.00018600

**24 (Ts)**

C 2.27700900 1.52081100 -0.95729800

O 1.71193800 2.32253700 -0.20059100

N 2.32616000 0.17205100 -0.98006000

S 1.61398200 -0.67301500 0.22457800

O 1.75143900 -2.10529900 -0.11941300

O 2.01950400 -0.26874200 1.58900300

C -0.16667100 -0.34446300 0.12224300

C -0.70468200 0.82857700 0.65634800

C -0.99491900 -1.28435200 -0.48741200

C -2.07671700 1.05163500 0.56683300

H -0.03375300 1.55782200 1.09258600

C -2.36770500 -1.04884200 -0.56818100

H -0.54874700 -2.19037000 -0.88306100

C -2.92893500 0.11940600 -0.04115900

H -2.49718500 1.96947800 0.97507500

H -3.01425000 -1.78356300 -1.04582400

C -4.41890800 0.36131000 -0.10172500

H -4.64687700 1.41318300 -0.31197200

H -4.89153200 -0.24810000 -0.88032800

H -4.90862800 0.11104000 0.85009000

H 2.86024700 1.92192300 -1.81929800

**24 (*p*-Cl-C_6_H_4_SO_2_)**

N -2.19262200 -0.92263900 -0.56648600

S -1.72584200 0.39852400 0.26415400

O -2.00674500 1.69989700 -0.38911100

O -1.96747600 0.32073200 1.72481200

C -3.52045500 -1.13000400 -0.73978600

O -4.50115000 -0.47910000 -0.37655300

H -3.66954700 -2.07137200 -1.32177200

C 0.06430600 0.16909400 0.08175800

C 0.84873300 1.25414000 -0.29797400

C 0.65938300 -1.05441700 0.39339100

C 2.23764800 1.12688000 -0.37139900

H 0.35387700 2.18787800 -0.54330800

C 2.04290400 -1.19778000 0.32180400

H 0.02803800 -1.89210000 0.66803300

C 2.81447000 -0.09970300 -0.05862800

H 2.86177500 1.96279800 -0.67005400

H 2.52062400 -2.14396300 0.55381100

Cl 4.57577100 -0.27662600 -0.15270300
